# Intramolecular N–O Bond Formation for the Synthesis of *N*‑Alkyl and *N*‑Aryl Isoxazolidines

**DOI:** 10.1021/jacs.5c06061

**Published:** 2025-06-05

**Authors:** Margaret Solecki, David Crich

**Affiliations:** † Department of Chemistry, 1355 University of Georgia, 302 East Campus Road, Athens, Georgia 30602, United States; ‡ Department of Pharmaceutical and Biomedical Sciences, University of Georgia, 250 West Green Street, Athens, Georgia 30602, United States; § Complex Carbohydrate Research Center, University of Georgia, 315 Riverbend Road, Athens, Georgia 30602, United States

## Abstract

We describe the synthesis of a series of readily assembled, variously substituted 3-(4-trifluoromethyl-2-nitrobenzene­sulfonamido)­alkyl silylperoxides and their conversion to the corresponding isoxazolidines in moderate to high yield by intramolecular N–O bond formation on removal of the sulfonamide protecting group. Cyclization with N–O bond formation was dependent on steric hindrance of the electrophilic silylperoxides, with primary systems cyclizing directly during the course of sulfonamide cleavage with thiolate anions. Secondary silyl peroxides, on the other hand, were best cyclized by warming in the presence of hexafluoroisopropanol after desulfonylation, while tertiary silylperoxides required deprotonation of the intermediate amine. Tertiary benzylic peroxides underwent cyclization by a novel mechanism, with the formation of intermediate trifluoromethyl­nitrophenyl peroxides, during the course of fluoride-mediated desilylation. The cyclization was extended to include the formation of a simple oxazine, but extrapolation to the formation of an oxazepine was foiled by competing Kornblum DeLaMare fragmentation at the level of the intermediate aminoperoxide.

## Graphical abstract

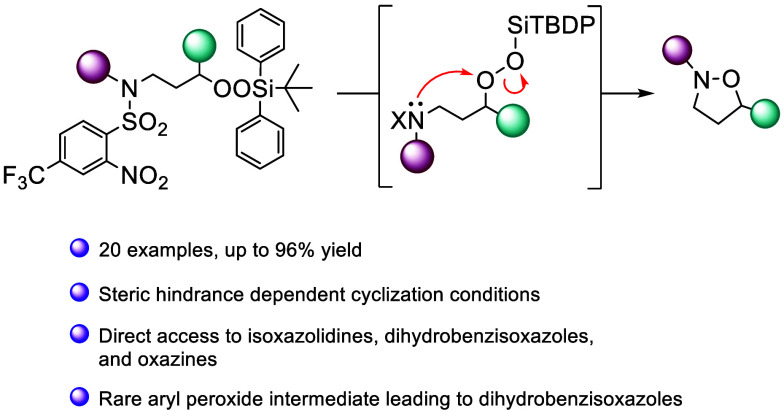

## Introduction

*N*,*N*,*O*-Trisubstituted hydroxylamines, or hydroxalogs, are convenient bioisosteres of tertiary amines, ethers, and even hydrocarbon moieties with the ability to modulate multiple physical and biological properties without molecular weight creep.^1–5^ When incorporated into five- and six-membered rings, where they are known as tetrahydroisoxazoles, or isoxazolidines, and tetrahydro-1,2-oxazines, they are found in multiple natural products^6–9^ and have an established history in medicinal chemistry^10^ that continues with the recent disclosure of a brain-penetrant non-small cell lung cancer therapeutic ORIC-114.^11^

Acyclic and exocyclic hydroxalogs can be assembled by a variety of methods^12^ but most convergently by direct N–O bond formation (Scheme 1a).^13–15^ The synthesis of endocyclic hydroxalogs, however, is largely based on cycloaddition methodologies and on the cyclization of unsaturated hydroxylamines^10,16–21^ and suffers from the limitations inherent to such processes. The synthesis of the more highly oxidized isoxazoles and isoxazolones and related heterocycles by N–O bond formation has been occasionally reported,^22,23^ but to our knowledge, the direct formation of isoxazolidines and their congeners by N–O bond formation is an unknown transformation.^24^ With this in mind, we undertook the development of an intramolecular version (Scheme 1b) of our intermolecular N–O bond formation route to acyclic trisubstituted hydroxylations (Scheme 1a) and report here on the reduction of this concept to practice.

**Scheme 1:**
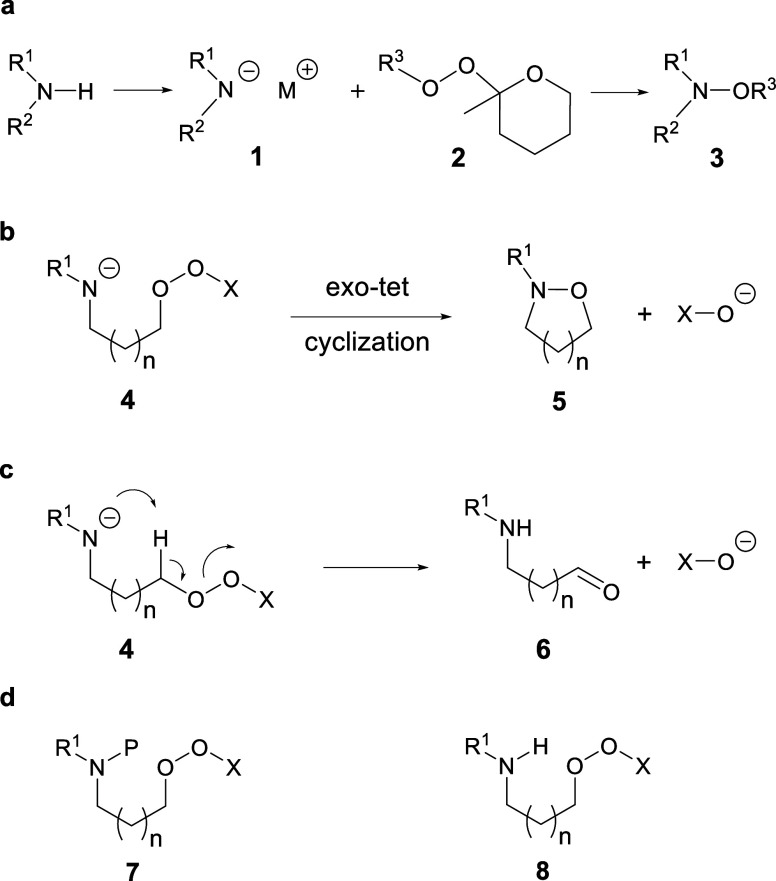
a) Existing Intermolecular N–O–Bond Formation, b) the Desired Intramolecular Version, c) Competing Kornblum DeLaMare Elimination, and d) the Required Precursors

## Results and Discussion

The intermolecular N–O bond-forming reaction, based on earlier work by the Dussault and Herzon laboratories on ether formation by the reaction of carbanions with tetrahydropyranyl peroxides,^25,26^ relies on the reaction of metalated secondary amines with alkyl 2-methyltetra­hydropyranyl peroxides (Scheme 1a) and is straightforward in execution. The desired intramolecular version (Scheme 1b), proceeding via an *exo*-*tet* mode cyclization,^27^ was expected to be similarly facile given Dussault’s earlier synthesis of cyclic ethers by the intramolecular reaction of carbanions with peroxides,^28,29^ provided that competing Kornblum DeLaMare elimination (Scheme 1c) could be avoided. Dussault’s cyclization of carbanions onto alkyl *tert*-butyl peroxides, with the expulsion of *tert*-butoxide as a leaving group,^28,29^ suggested that the tetrahydropyranyl peroxides would not be required for the intramolecular N–O bond-forming process and that either *tert*-butyl or silyl peroxides would suffice. The crux of the problem, therefore, was determined to be the identification of a suitable amine protecting group strategy that would enable the synthesis of a protected amino peroxide **7** and its deprotection to amino peroxide **8** without affecting the acid and base labile peroxide unit (Scheme 1d). Based on Dussault’s demonstration of the moderate stability of various peroxides toward thiophenate,^30^ we selected the thiolate-cleavable nitrobenzenesulfonamides^31^ with the expectation that they would also be stable to the conditions of the Mukaiyama silyl peroxidation reaction.^32^

We assembled a series of mono- and dinitrobenzenesulfonyl-protected *N*-(1-naphthylmethyl)-*N*-(3-butenyl)­amines **9**–**13** (Supporting Information) and subjected them to Mukaiyama silyl hydroperoxidation by stirring with triethylsilane and catalytic Co­(acac)_2_ under oxygen to give the triethylsilyl peroxides **14**–**19**. To afford a series of more tractable peroxides,^33^ the triethylsilyl group was removed with tetrabutylammonium fluoride and the *tert*-butyldiphenylsilyl group was installed with *tert*-butyldiphenylsilyl chloride, giving **20**–**24** in good yields (Scheme 2). Attempts to access the *tert*-butyldiphenylsilyl peroxides directly from the alkenes employing *tert*-butyldiphenylsilane^34^ in the silyl hydroperoxidation reaction were unsuccessful.

**Scheme 2:**
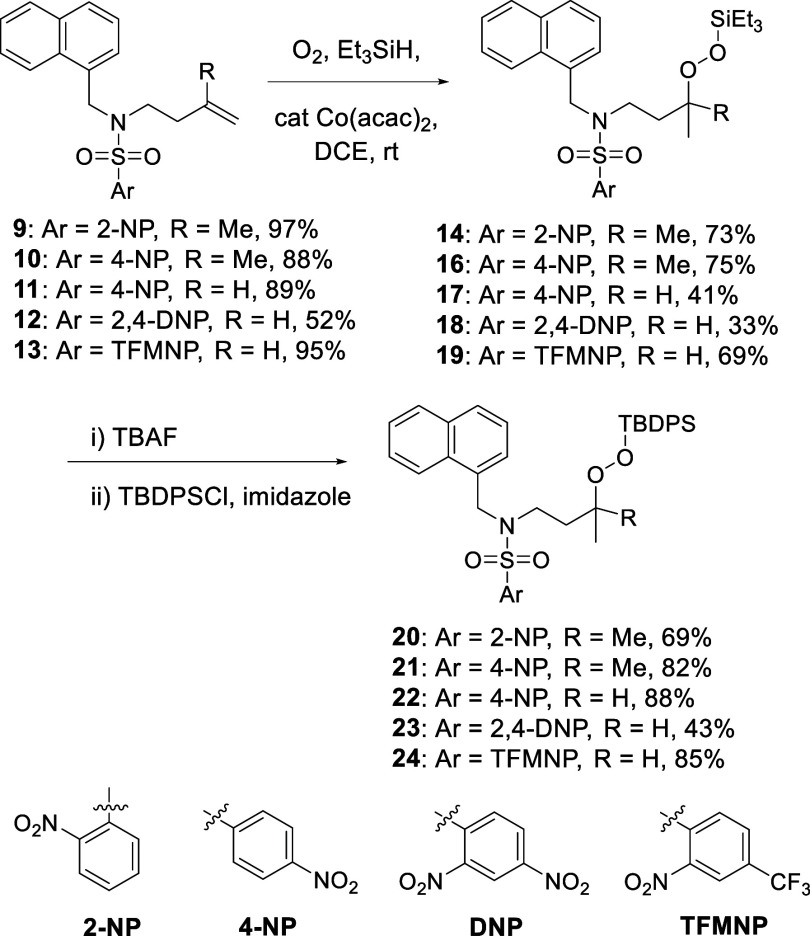
Synthesis of *N*-Sulfonyl Silylperoxyamines for the Exploration of Desulfonylation

We then turned to the exploration of conditions for sulfonamide cleavage employing either thiophenol or a variety of water-soluble aliphatic thiols in the presence of various bases (Table 1). Working first with the tertiary triethylsilyl protected peroxides **14**–**19** and monitoring reaction progress by ESI mass spectrometry, it was determined that none of the conditions employed afforded the simple desulfonylation product or the desired isoxazolidine **25** in any significant quantity but rather gave the aminoalcohol **26** arising from desulfonylation with concomitant reduction of the peroxide (Table 1, entries 1–4). Turning to the secondary *tert*-butyldiphenylsilyl peroxides **22** and **23**, exposure of 4-nitrophenyl sulfonamide **22** to thiophenol and potassium *tert*-butoxide in THF at room temperature gave a 94% isolated yield of the aminoketone **27** by Kornblum DeLaMare elimination (Table 1, entry 5). Comparable results were observed with sulfonamide **22** on treatment with 2-mercaptoethanol and DBU in DMF at room temperature, albeit the desired isoxazolidine **29** was isolated from the reaction mixture in 4% yield (Table 1, entry 6). Moving to the dinitrophenyl sulfonamide **23** and retaining the 2-mercaptoethanol and DBU under DMF conditions, it was possible to avoid the formation of the amino ketone **27** and to obtain an improved 28% yield of the isoxazolidine **29**; however, the major product under these conditions was **30** in which Kornblum DeLaMare elimination was coupled with substitution of one of the nitro groups in the sulfonamide by the thiolate (Table 1, entry 7). When the latter reaction was conducted in acetonitrile instead of DMF, the isoxazolidine **29** was isolated in 22% yield, but nucleophilic aromatic substitution of one of the nitro groups to give **31** again predominated, albeit fragmentation of the peroxide to the ketone could be avoided (Table 1, entry 8). This matrix of results demonstrated that given sufficiently rapid desulfonylation, the desired N–O bond-forming isoxazolidine synthesis was possible and may become the major product in the absence of competing nitro group substitution. Nuclear Overhauser effect studies between the *S*-methylene group and the aromatic ring identified the locus of the substitution reaction in **30** and **31** and indicated the need to replace the 4-nitro group in the 2,4-dinitrophenylsulfonamide moiety by an alternative electron-withdrawing group. We considered the 2,6-bis­(trifluoromethyl) and 2,4,6-tris­(trifluoromethyl)­phenylsulfonamides advocated by the Yokoshima and Maulide laboratories^35,36^ but opted first to try the commercially available 2-nitro-4-trifluoromethyl­phenylsulfonyl group, here dubbed the TFMNP group, as earlier work from our laboratory suggested it would be cleanly and rapidly removed.^37^ Accordingly, we prepared the TFMNP precursor **13** and the protected secondary triethylsilyl and *tert*-butyldiphenylsilyl peroxides **19** and **24** (Supporting Information) and exposed the latter to 2-mercpatoethanol and DBU in acetonitrile at 0 °C. ESIMS monitoring of the reaction progress revealed the clean formation of an intermediate amino silylperoxide **32**, with only trace amounts of the isoxazolidine **29**. Chromatographic separation of **32** over silica gel and subsequent concentration of the fractions under vacuum on a rotary evaporator at 35 °C resulted in slow cyclization and, after further chromatographic purification, isolation of the isoxazolidine **29** in 72% yield (Table 1, entry 9). Control experiments determined that the cyclization of **32** to **29** did not arise from heating on the rotary evaporator or from exposure to silica gel but rather from the act of concentration of the chromatographic fractions. This suggested an autocatalytic concentration-dependent intermolecular component to the cyclization, leading us to propose that a minor amount of *tert*-butyldiphenylsilanol **33**, with an estimated p*K*_a_ of 12,^38^ generated during an initial slow cyclization to **29** was serving as an acid and catalyzing further cyclization during the concentration (Scheme 3). To test this hypothesis, the crude reaction mixture from the desulfonylation step was diluted with dichloromethane, washed with brine, and finally treated with hexafluoroisopropanol (p*K*_a_ 9.3),^39–42^ which was previously known to promote hydrogen peroxide-mediated oxidations,^43–46^ and heated to 35 °C for 2 h, resulting in smooth cyclization to isoxazolidine **29** and its isolation in 91% yield after chromatographic purification (Table 1, entry 10, Scheme 3).

**Scheme 3:**
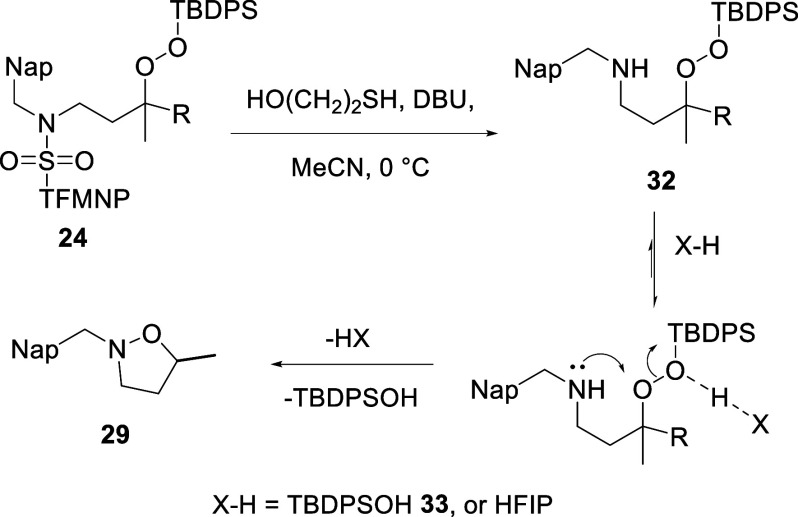
Proposed Mechanism for Silanol and Hexafluoroisopropanol Catalysis of Cyclization

**Table 1:**
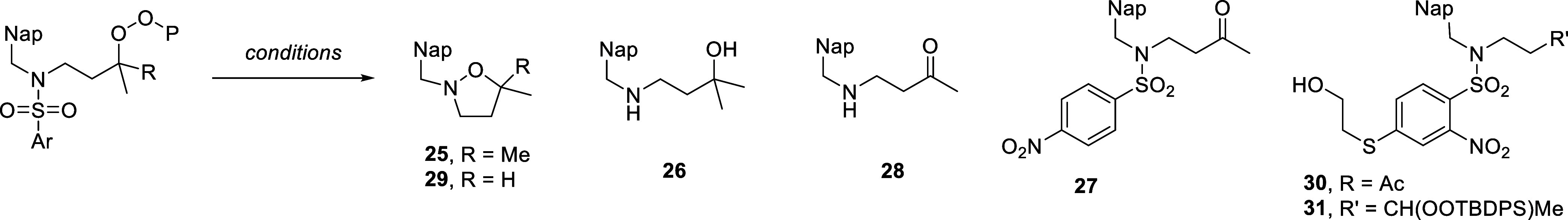
Exploration of the Sulfonamide Cleavage Conditions and Influence of the Silyl Group

**Entry**	**Subs**	**Ar**	**P**	**R**	**Conditions**	**25** or **29** (yield)	**26** or **28** (yield)	**27**, **30**, or **31** (yield)
**1**	**14**	2-NP	TES	Me	PhSH, K_2_CO_3_, DMF, RT, 3 h	0%	**26** ^a^	na
**2**	**16**	4-NP	TES	Me	PhSH, Cs_2_CO_3_, DMF, RT, 3 h	0%	**26** ^a^	na
**3**	**16**	4-NP	TES	Me	HSCH_2_COOH, Et_3_N, DCM, RT, 1 h	0%	**26** ^a^	na
**4**	**16**	4-NP	TES	Me	HS(CH_2_)_2_OH, DBU, DMF, 0 °C, 4 h	0%	**26**, 87%	na
**5**	**22**	4-NP	TBDPS	H	PhSH, KO^*t*^Bu, THF, RT 4 h	0%	na	**27**, 94%
**6**	**22**	4-NP	TBDPS	H	HS(CH_2_)_2_OH, DBU, DMF, RT, 4 h	4%	na	**27**, 90%
**7**	**23**	DNP	TBDPS	H	HS(CH_2_)_2_OH, DBU, DMF, 0 °C, 5 min	28%	**28**, 17%	**30**, 51%
**8**	**23**	DNP	TBDPS	H	HS(CH_2_)_2_OH, DBU, MeCN, 0 °C, 5 min	22%	na	**31**, 48%
**9**	**24**	TFMNP	TBDPS	H	i) HS(CH_2_)_2_OH, DBU, MeCN, 0 °C, 5 min, ii) SiO_2_ chromatography, iii) concentration	72%	na	na
**10**	**24**	TFMNP	TBDPS	H	i) HS(CH_2_)_2_OH, DBU, MeCN, 0 °C, 1 min, ii) HFIP, 35 °C, 2 h	91%	na	na

a Not isolated; major product by ESIMS.

With conditions for amino silyl peroxide generation and cyclization in hand, we turned to the reaction scope, preparing first a number of substrates by minor variations of the methods employed in Scheme 2 or by nucleophilic substitution methods for the formation of the primary silyl peroxides (Supporting Information). Further examples of cyclization onto secondary *tert*-butyldiphenylsilyl peroxides proceeded smoothly and demonstrated that the nitrogen substituent could be either benzylic, alkyl, or, interestingly in view of the reduced nucleophilicity of anilines vis à vis amines, even aryl (Table 2, entries 1–4). Attention was then shifted to substitution at the 2-position of the 3-amino peroxide substrate (Table 2, entry 5) and to unsubstituted primary peroxides (Table 2, entries 6 and 7), all of which cyclized directly in high yield in the course of the desulfonylation without the need for heating in the presence of HFIP, thereby demonstrating the sensitivity of the N–O bond-forming reaction to steric hindrance. Next we turned to cyclization onto tertiary peroxides, which were problematic in the intermolecular reaction and forced us to resort to the more electrophilic peroxyesters rather than the tetrahydropyranyl peroxides.^13,14^ In the event, these tertiary systems required deprotonation of the deprotected amines, using ethylmagnesium bromide, after which the cyclizations proceeded in mostly excellent yield at room temperature independent of the nature of the nitrogen substituent (Table 2, entries 8–10). Two diastereomeric 3-substituted secondary aminoperoxides **51** and **52** cyclized after deprotection and exposure to HFIP to give the corresponding isoxazolidines **53** and **54** without loss of stereochemical integrity, albeit in only moderate yield (Table 2, entries 11 and 12). We presume that the moderate yield in these examples is due to steric hindrance, consistent with the influence of substitution in the intermolecular variant of the reaction,^13,14^ but cannot exclude the possibility that the additional substitution disfavors the conformation required for cyclization.

**Table 2:**
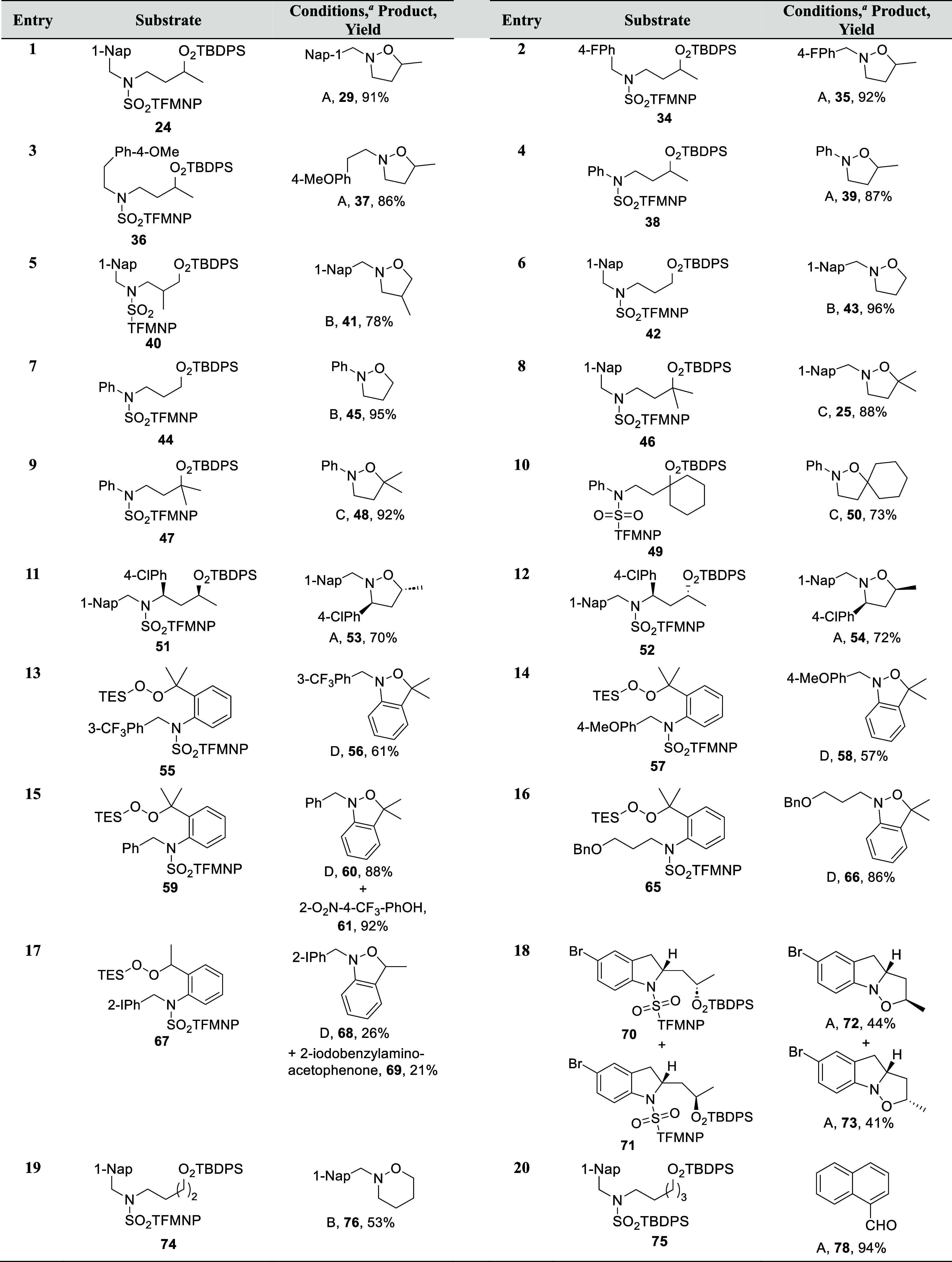
Scope of the Cyclization Reactions

a A: i) HS­(CH_2_)_2_OH, DBU, MeCN, −20 °C, 1 min, ii) HFIP, 35 °C, 2 h. B: HS­(CH_2_)_2_OH, DBU, MeCN, −20 °C, 1 min. C: i) HS­(CH_2_)_2_OH, DBU, MeCN, −20 °C, 1 min, ii) EtMgBr, THF, 0 °C to RT. D: TBAF, THF, 0 °C, 10 min.

We then turned to the formation of the *N*-alkyl-1,3-dihydro-2,1-benzisoxazoles, which were all but unknown until our recent description of them.^47^ To our surprise, attempted exchange of the initial triethylsilyl group, installed in the course of the peroxidation reaction, for the *tert*-butyldiphenylsilyl group by treatment with tetrabutylammonium fluoride followed by resilylation with the more bulky silyl chloride, as conducted successfully for all other classes of substrate (Scheme 2), resulted in direct cyclization to the targeted isoxazolidines in moderate to high yield (Table 2, entries 13–16). Benzo-fused isoxazolidine **58** (Table 2, entry 14) was crystalline, and its X-ray structure provided final and unambiguous proof of N–O bond formation (CCDC 2451510 and Supporting Information) and underlined the pyramidal nature of its sp^3^-hybridized nitrogen atom consistent with expectation and earlier work.^47^ Closer investigation of Table 2, entry 15 revealed that the cyclization of **59** to **60** took place during the desilylation reaction and was accompanied by the stoichiometric formation of 2-nitro-4-trifluoromethylphenol **61**. These observations led us to propose a mechanism involving transfer of the TFMNP group from the sulfonamide to the initial peroxide **62**, with the loss of sulfur dioxide, by intramolecular nucleophilic aromatic substitution. This TFMNP group migration generates a more reactive electrophilic peroxide **64** that undergoes spontaneous cyclization (Scheme 4) to generate products **60** and **61** in the observed 1:1 ratio. The eight-membered cyclic Meisenheimer complex **63** leading to the activated peroxide **64** is favored by the highly conformationally restricted system and by the limited degree of transannular strain in unsaturated medium-sized rings. Aryl peroxides are extremely rare^48^ owing to the weakness of their O–O bond,^49^ whose homolytic cleavage affords a persistent phenoxy radical, but have been previously accessed by comparable nucleophilic aromatic substitution reactions employing hydrogen peroxide as a nucleophile.^50–54^ Application of the same protocol to the secondary benzylic peroxide **67** also gave the desired benzo-fused heterocyclic product **68** in moderate yield but was accompanied by the Kornblum DeLaMare elimination product **69** (Table 2, entry 17) consistent with literature observations on the ease of the Kornblum DeLaMare reaction of benzylic peroxides.^26^ Cyclization of the diastereomeric indoline-based secondary peroxides **70** and **71** was next examined and was found to give the corresponding diastereomerically pure fused tricyclic systems **72** and **73** featuring a bridgehead nitrogen atom with moderate yield following exposure of the crude amines to HFIP (Table 2, entry 18).

**Scheme 4:**
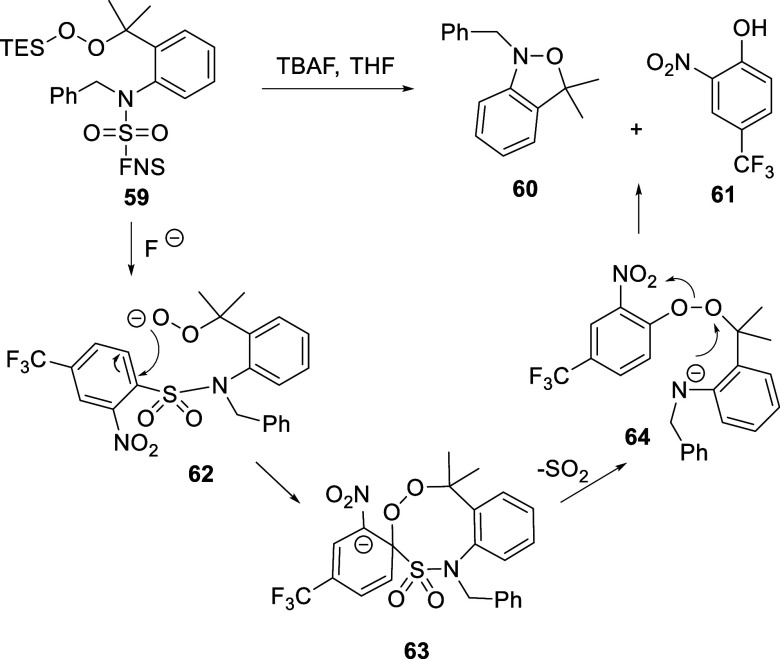
Proposed Mechanism for the Spontaneous Cyclization of Conformationally Restricted Substrates

Finally, we briefly attempted the extrapolation of the N–O bonding reaction to the synthesis of 1,2-oxazines and oxazepines, preparing first the homologous substrates **74** and **75** in the standard manner (Supporting Information). Treatment of **74** with 2-mercaptoethanol and DBU in MeCN in the standard manner directly afforded the 1,2-oxazine **76** in 53% yield (Table 2, entry 19), with the reduced yield with respect to the immediately lower homologue **43** (Table 2, entry 6) arising from the slower formation of the six-membered ring, and the consequently greater possibility of competing reaction modes. Deprotection of **75** under the same conditions did not afford the anticipated 1,2-oxazepine **77**. An examination of the crude reaction mixture by ESIMS revealed a base peak with *m*/*z* 224.1422 corresponding to a molecular formula of C_16_H_18_N, while workup led to the isolation of 1-naphthaldehyde **78** in 94% yield (Table 2, entry 20). We interpret these findings in terms of Kornblum DeLaMare elimination by the intramolecular base in amino peroxide **79** outcompeting the desired cyclization and giving rise to an amino aldehyde that dehydrates to give the iminium ion **80** and, after tautomerization, the iminium ion **81**. Both **80** and **81** are consistent with the observed base peak in the mass spectrum of the reaction mixture, and the hydrolysis of **81** yields piperidine and the isolated aldehyde **78** (Scheme 5).

**Scheme 5:**
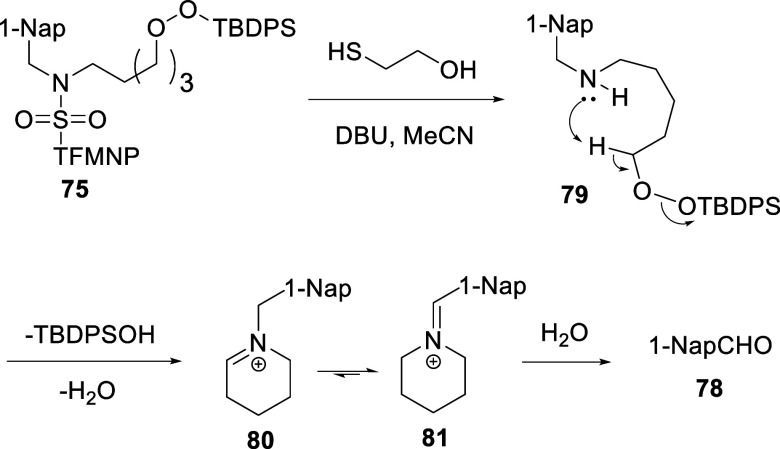
Proposed Mechanism for the Decomposition of **75** on Deprotection

Finally, given our interest in using hydroxalogs as stereoadaptive isosteres of tertiary chiral centers,^1,47,55^ we determined the barrier to pyramidal inversion at nitrogen in a representative isoxazolidine, **25**. Using the usual variable-temperature NMR methods, we find this barrier to be approximately 14 kcal·mol^–1^, consistent with literature values.^56–59^

## Conclusions

Overall, we describe a novel method for intramolecular N–O bond formation by *exo*-*tet* mode nucleophilic attack of secondary amines on silyl peroxides permitting access to a broad range of *N*-substituted isoxazolidines, many of which could not be readily accessed by the existing cycloaddition or cyclization methodologies. The precursor *N*-sulfonyl silylperoxyamines are readily assembled by classical methods in conjunction with the Mukaiyama silyl hydroperoxylation reaction, and the amines are released for cyclization by treatment of the sulfonamides with thiolate anions. Extension to the formation of a homologous 1,2-oxazine was demonstrated, albeit in only modest yield, while further extrapolation to the formation of a 1,2-oxazepine was thwarted by competing Kornblum DeLaMare elimination.

## Supplementary Material



## Data Availability

The data underlying this study are available in the published article and its Supporting Information.

## Notes

Full experimental details, copies of NMR spectra, and details of the crystal structure of compound **58** (CCDC 2451510) (PDF)
