# Supplementary material for: Intramolecular N–O Bond Formation for the Synthesis of N‑Alkyl and N‑Aryl Isoxazolidines
Source: J Am Chem Soc. 2025 Jun 5;147(24):21053–9. doi: 10.1021/jacs.5c06061 (PMC12186478; doi:10.1021/jacs.5c06061)
Supplement: Supplementary file 1 [file ja5c06061_si_001.pdf]

SUPPORTING INFORMATION

for

Intramolecular N–O-Bond Formation for the Synthesis of *N*-Alkyl and *N*-Aryl Isoxazolidines

Margaret Solecki[a,b] and David Crich.[a,b,c*]

[a]Department of Chemistry, University of Georgia, 140 Cedar Street, GA 30602, USA.

[b]Department of Pharmaceutical and Biomedical Sciences, University of Georgia, 250 West Green Street, Athens, GA 30602, USA.

[c]Complex Carbohydrate Research Center, University of Georgia, 315 Riverbend Road, Athens, GA, 30602, USA.

*Correspondence to: david.crich@uga.edu

Table of Contents

**General Experimental**	**S9**
**General Procedures**	**S10**
**Synthesis and Characterization of Substrates for Protecting Group Screen**	**S20**
**Protecting Group Screen**	**S30**
**Synthesis and Characterization of Substrates**	**S38**
**Synthesis and Characterization of Cyclic Hydroxylamines**	**S110**
**Attempted formation of Oxazepanes**	**S137**
**Determination of Barrier to Inversion by VT NMR**	**S138**
**Spectra**	**S139**
**Spectra for VT NMR Study**	**S423**
**X-ray data for compound 58**	**S427**
**References**	**S437**

	**Experimental**	**Spectra**
Bis(2,2,6,6-tetramethyl-3,5-heptanedionato)cobalt(II) (Co(thd)2) (**S10**)	S14	-
N-(Naphthalen-1-ylmethyl)-4-nitrobenzenesulfonamide (**S1**)	S20	S139-140
N-(3-Methylbut-3-en-1-yl)-*N*-(naphthalen-1-ylmethyl)-4-nitrobenzenesulfonamide (**10**)	S21	S141-142
N-(3-Methyl-3-((triethylsilylperoxy)butyl)-*N*-(naphthalene-1-ylmethyl)-4-nitrobenzenesulfonamide (**16**)	S22	S143-144
2-Methyl-4-((naphthalen-1-ylmethyl)amino)butan-2-ol (**26**)	S31	S145-146
N-(But-3-en-1-yl)-*N*-(naphthalen-1-ylmethyl)-4-nitrobenzenesulfonamide (**11**)	S23	S147-148
N-(Naphthalene-1-ylmethyl)-4-nitro-*N*-(3-(triethylsilylperoxy)butyl)benzenesulfonamide (**17**)	S24	S149-150
N-(3-(*tert*-Butyldiphenylsilylperoxy)butyl)-*N*-(naphthalene-1-ylmethyl)-4-nitrobenzenesulfonamide (**22**)	S25	S151-152

N-(Naphthalen-1-ylmethyl)-4-nitro-*N*-(3-oxobutyl)benzenesulfonamide (**27**)	S32	S153-154
N-(Naphthalen-1-ylmethyl)-2, 4-dinitrobenzenesulfonamide (**S2**)	S26	S155-156
N-(But-3-en-1-yl)-*N*-(naphthalen-1-ylmethyl)-2, 4-dinitro-4-benzenesulfonamide (**12**)	S27	S157-158
N-(Naphthalene-1-ylmethyl)-2, 4-dinitro-*N*-(3-(triethylsilylperoxy)butyl)-benzenesulfonamide (**18**)	S28	S159-160
N-(3-(*tert*-Butyldiphenylsilylperoxy)butyl)-*N*-(naphthalene-1-ylmethyl)-2, 4-dinitrobenzenesulfonamide (**23**)	S29	S161-162
4-((Naphthalen-1-ylmethyl)amino)butan-2-one (**28**)	S34	S163
4-(2-Hydroxyethylthio)-*N*-(naphthalen-1-ylmethyl)-2-nitro-*N*-(3-oxobutyl)benzenesulfonamide (**30**)	S34	S164-166
N-(3-(*tert*-Butyldiphenylsilylperoxy)butyl)-4-(2-hydroxyethylthio)-*N*-(naphthalen-1-ylmethyl)-2-nitrobenzenesulfonamide (**31**)	S37	S167
N-(Naphthalen-1-ylmethyl)-2-nitro-4-(trifluoromethyl)benzenesulfonamide (**S3**)	S38	S168-169
N-(But-3-en-1-yl)-*N*-(naphthalen-1-ylmethyl)-2-nitro-4-(trifluoromethyl)benzenesulfonamide (**13**)	S39	S170-171
N-(Naphthalen-1-ylmethyl)-2-nitro-*N*-(3-(triethylsilylperoxy)butyl)-4-(trifluoromethyl)benzenesulfonamide (**19**)	S40	S172-173
N-(3-(*tert*-Butyldiphenylsilylperoxy)butyl)-*N*-(naphthalen-1-ylmethyl)-2-nitro-4-(trifluoromethyl)benzenesulfonamide (**24**)	S41	S174-175
5-Methyl-2-(naphthalen-1-ylmethyl)isoxazolidine **(29)**	S110	S176-181
N-(4-Fluorobenzyl)-2-nitro-4-(trifluoromethyl)benzenesulfonamide (**S4**)	S42	S182-183
N-(But-3-en-1-yl)-*N*-(4-fluorobenzyl)-2-nitro-4-(trifluoromethyl)benzenesulfonamide (**4a**)	S43	S184-186
N-(4-Fluorobenzyl)-2-nitro-*N*-(3-(triethylsilylperoxy)butyl)-4-(trifluoromethyl)benzenesulfonamide (**4b**)	S44	S187-188
N-(3-(*tert*-Butyldiphenylsilylperoxy)butyl)-*N*-(4-fluorobenzyl-2-nitro-4-(trifluoromethyl)benzenesulfonamide (**34**)	S45	S189-190
2-(4-Fluorobenzyl)-5-methylisoxazolidine **(35)**	S111	S191-195
N-(4-Methoxyphenethyl)-2-nitro-4-(trifluoromethyl)benzenesulfonamide (**S5**)	S46	S196-197

N-(But-3-en-1-yl)-*N*-(4-methoxyphenethyl)-2-nitro-4-(trifluoromethyl)benzenesulfonamide (**5a**)	S47	S198-199
N-(4-Methoxyphenethyl)-2-nitro-*N*-(3-(triethylsilylperoxy)butyl)-4-(trifluoromethyl)benzenesulfonamide (**5b**)	S48	S200-201
N-(3-(*tert*-Butyldiphenylsilylperoxy)butyl)-*N*-(4-methoxyphenethyl)-2-nitro-4-(trifluoromethyl)benzenesulfonamide (**36**)	S49	S202-203
2-(Methoxyphenethyl)-5-methylisoxazolidine **(47)**	S112	S204-208
2-Nitro-*N*-phenyl-4-(trifluoromethyl)benzenesulfonamide (**S6**)	S50	S209-210
N-(But-3-en-1-yl)-2-nitro-*N*-phenyl-4-(trifluoromethyl)benzenesulfonamide (**6a**)	S51	S211-212
2-Nitro-*N*-phenyl-*N*-(3-(triethylsilylperoxy)butyl)-4-(trifluoromethyl)benzenesulfonamide (**6b**)	S52	S213-214
N-(3-(*tert*-Butyldiphenylsilylperoxy)butyl)-2-nitro-*N*-phenyl-4-(trifluoromethyl)benzenesulfonamide (**38**)	S53	S215-216
5-Methyl-2-phenylisoxazolidine **(39)**	S114	S217-219
N-(2-Methylallyl)-*N*-(naphthalen-1-ylmethyl)-2-nitro-4-(trifluoromethyl)benzenesulfonamide (**3a**)	S54	S220-221
N-(3-Hydroxy-2-methylpropyl)-*N*-(naphthalen-1-ylmethyl)-2-nitro-4-(trifluoromethyl)benzenesulfonamide (**3b**)	S55	S222-224
N-(3-Iodo-2-methylpropyl)-*N*-(naphthalen-1-ylmethyl)-2-nitro-4-(trifluoromethyl)benzenesulfonamide (**3c**)	S56	S225-227
N-(3-Hydroperoxy-2-methylpropyl)-*N*-(naphthalen-1-ylmethyl)-2-nitro-4-(trifluoromethyl)benzenesulfonamide (**3d**)	S57	S228-230
N-(3-(*tert*-Butyldiphenylsilylperoxy)-2-methylpropyl)-*N*-(naphthalen-1-ylmethyl)-2-nitro-4-(trifluoromethyl)benzenesulfonamide (**40**)	S58	S231-233
4-Methyl-2-(naphthalen-1-ylmethyl)isoxazolidine **(41)**	S115	S234-237
N-Allyl-*N*-(naphthalen-1-ylmethyl)-2-nitro-4-(trifluoromethyl)benzenesulfonamide (**3e**)	S59	S238-239
N-(3-Hydroxypropyl)-*N*-(naphthalen-1-ylmethyl)-2-nitro-4-(trifluoromethyl)benzenesulfonamide (**3f**)	S60	S240-241
N-(3-Iodopropyl)-*N*-(naphthalen-1-ylmethyl)-2-nitro-4-(trifluoromethyl)benzenesulfonamide (**3g**)	S61	S242-243
N-(3-Hydroperoxypropyl)-*N*-(naphthalen-1-ylmethyl)-2-nitro-4-(trifluoromethyl)benzenesulfonamide (**3h**)	S62	S244-245

N-(3-(*tert*-Butyldiphenylsilylperoxy)propyl)-*N*-(naphthalen-1-ylmethyl)-2-nitro-4-(trifluoromethyl)benzenesulfonamide (**42**)	S63	S246-247
2-(Naphthalen-1-ylmethyl)-isoxazolidine **(43)**	S116	S248-251
N-Allyl-2-nitro-*N*-phenyl-4-(trifluoromethyl)benzenesulfonamide (**6c**)	S64	S252-253
N-(3-Hydroxypropyl)-2-nitro-*N*-phenyl-4-(trifluoromethyl)benzenesulfonamide (**6d**)	S65	S254-255
N-(3-Iodopropyl)-2-nitro-*N*-phenyl-4-(trifluoromethyl)benzenesulfonamide (**6e**)	S66	S256-259
N-(3-Hydroperoxypropyl)-2-nitro-*N*-phenyl-4-(trifluoromethyl)benzenesulfonamide (**6f**)	S67	S260-261
N-(3-(*tert*-Butyldiphenylsilylperoxy)propyl)-2-nitro-*N*-4-(trifluoromethyl)benzenesulfonamide (**44**)	S68	S262-263
2-Phenylisoxazolidine **(45)**	S117	S264-266
N-(3-Methylbut-3-en-1-yl)-*N*-(naphthalen-1-ylmethyl)-2-nitro-4-(trifluoromethyl)benzenesulfonamide (**3i**)	S69	S267-268
N-(3-Methyl-3-(triethylsilylperoxy)butyl)-*N*-(naphthalen-1-ylmethyl)-2-nitro-4-(trifluoromethyl)benzenesulfonamide (**3j**)	S70	S269-270
N-(3-(*tert*-Butyldiphenylsilylperoxy)-3-methylbutyl)-*N*-(naphthalen-1-ylmethyl)-2-nitro-4-(trifluoromethyl)benzenesulfonamide (**46**)	S71	S271-272
(3-((*tert*-Butyldiphenylsilyl)peroxy)-3-methy-*N*-(naphthalen-1-ylmethyl)butan-1-amine (**3k**)	S72	S273-274
5,5-Dimethyl-2-(naphthalen-1-ylmethyl)isoxazolidine **(25)**	S118	S275-278
3-Methylbut-3-en-1-yl 4-methylbenzenesulfonate (**S11**)	S73	S279-280
N-(3-Methylbut-3-en-1-yl)-2-nitro-*N*-phenyl-4-(trifluoromethyl)benzenesulfonamide (**6g**)	S74	S281-282
N-(3-Methyl-3-(triethylsilylperoxy)butyl)-2-nitro-*N*-phenyl-4-(trifluoromethyl)benzenesulfonamide (**6h**)	S75	S283-284
N-(3-(*tert*-Butyldiphenylsilylperoxy)-3-methylbutyl)-2-nitro-*N*-phenyl-4-(trifluoromethyl)benzenesulfonamide (**47**)	S76	S285-286
N-(3-((*tert*-Butyldiphenylsilyl)peroxy)-3-methylbutyl)-aniline (**6i**)	S77	S287-288
5,5-Dimethyl-2-phenylisoxazolidine **(48)**	S119	S289-291
2-(Cyclohex-1-en-yl)ethan-1-ol (**S12**)	S78	S292-293
N-(2-(Cyclohex-1-en-1-yl)ethyl)-2-nitro-*N*-phenyl-4-(trifluoromethyl)benzenesulfonamide (**12a**)	S79	S294-295

2-Nitro-*N*-phenyl-*N*-(2-(1-(triethylsilylperoxy)cyclohexyl)ethyl)-4-(trifluoromethyl)benzenesulfonamide (**12b**)	S80	S296-297
N-(2-(1-(*tert*-Butyldiphenylsilylperoxy)cyclohexyl)ethyl)-2-nitro-*N*-phenyl-4-(trifluoromethyl)benzenesulfonamide (**49**)	S81	S298-399
N-(2-(1-((*tert*-Butyldiphenylsilyl)peroxy)cyclohexyl)ethyl)aniline **(12c)**	S82	S300-301
2-Phenyl-1-oxa-2-azaspiro[4.5]decane **(50)**	S120	S302-304
1-(4-Chlorophenyl)but-3-en-1-ol (**S13**)	S83	S305-307
N-(1-(4-Chlorophenyl)but-3-en-1-yl)-*N*-(naphthalen-1-ylmethyl)-2-nitro-4-(trifluoromethyl)benzenesulfonamide (**13a**)	S84	S308-310
anti-*N*-(1-(4-chlorophenyl)-3-(triethylsilylperoxy)butyl)-*N*-(naphthalene-1-ylmethyl)-2-nitro-4-(trifluoromethyl)benzenesulfonamide **(13b)**	S85	S311-312
syn-*N*-(1-(4-chlorophenyl)-3-(triethylsilylperoxy)butyl)-*N*-(naphthalene-1-ylmethyl)-2-nitro-4-(trifluoromethyl)benzenesulfonamide **(13c)**	S85	S320-322
anti-*N*-(3-(*tert*-Butyldiphenylsilylperoxy)butyl)-1-(4-chlorophenyl)butyl)-*N*-(naphthalen-1-ylmethyl-2-nitro-4-(trifluoromethyl)benzenesulfonamide (**51**)	S87	S313-314
syn-*N*-(3-(*tert*-Butyldiphenylsilylperoxy)butyl)-1-(4-chlorophenyl)butyl)-*N*-(naphthalen-1-ylmethyl-2-nitro-4-(trifluoromethyl)benzenesulfonamide (**52**)	S88	S323-324
trans-3-(4-Chlorophenyl)-5-methyl-2-(naphthalene-1-ylmethyl)isoxazolidine **(53)**	S121	S315-319
cis-3-(4-Chlorophenyl)-5-methyl-2-(naphthalene-1-ylmethyl)isoxazolidine **(54)**	S122	S325-330
2-Nitro-*N*-(2-(prop-1-en-2-yl)phenyl)-4-(trifluoromethyl)benzenesulfonamide (**S7**)	S89	S331-332
2-Nitro-*N*-(2-(prop-1-en-2-yl)phenyl)-4-(trifluoromethyl)-*N*-(3-(trifluoromethyl)benzyl)benzenesulfonamide (**7a**)	S90	S333-334
2-Nitro-*N*-(2-(2-(triethylsilylperoxy)propan-2-yl)phenyl)-4-(trifluoromethyl)-*N*-(3-(trifluoromethyl)benzyl)benzenesulfonamide (**55**)	S91	S335-336
3,3-Dimethyl-1-(3-trifluoromethyl)benzyl)-1,3-dihydrobenzo[*c*]isoxazole **(56)**	S123	S337-340
N-(4-Methoxybenzyl)-2-nitro-*N*-(2-(prop-1-en-2-yl)phenyl)-4-(trifluoromethyl)benzenesulfonamide (**7b**)	S92	S341-342

N-(4-Methoxybenzyl)-2-nitro-*N*-(2-(2-(triethylsilylperoxy)propan-2-yl)phenyl)-4-(trifluoromethyl)benzenesulfonamide (**57**)	S93	S343-344
1-(4-Methoxybenzyl)-3,3-dimethyl-1,3-dihydrobenzo[*c*]isoxazole **(58)**	S124	S345-347
N-Benzyl-2-nitro-*N*-(2-(prop-1-en-2-yl)phenyl)-4-(trifluoromethyl)benzenesulfonamide (**7c**)	S94	S348-349
N-Benzyl-2-nitro-*N*-(2-(2-(triethylsilylperoxy)propan-2-yl)phenyl)-4-(trifluoromethyl)benzenesulfonamide (**59**)	S95	S350-351
1-Benzyl-3,3-dimethyl-1,3-dihydrobenzo[*c*]isoxazole **(60)**	S125	S352-354
2-Nitro-4-(trifluoromethyl)phenol (**61**)	S125	S355-359
N-(3-Benzyloxy)propyl)2-nitro-*N*-(2-(prop-1-en-2-yl)phenyl)-4-(trifluoromethyl)benzenesulfonamide (**7d**)	S96	S360-361
N-(3-Benzyloxy)propyl)2-nitro-*N*-(2-(2-(triethylsilylperoxy)propan-2-yl)phenyl)-4-(trifluoromethyl)benzenesulfonamide (**65**)	S97	S362-363
1-(3-(Benzyloxy)propyl)-3,3-dimethyl-1,3-dihydrobenzo[*c*]isoxazole **(66)**	S127	S364-366
2-Nitro-4-(trifluoromethyl)-*N*-(2-styryl)benzenesulfonamide (**S8**)	S98	S367-368
N-(2-Iodobenzyl)-2-nitro-4-(trifluoromethyl)-*N*-(2-vinylphenyl) benzenesulfonamide (**8a**)	S99	S369-370
N-(2-Iodobenzyl)-2-nitro-*N*-(2-(1-(triethylsilylperoxy)ethyl)phenyl)-4-(trifluoromethyl) benzenesulfonamide (**67**)	S100	S371-372
1-(2-Iodobenzyl)-3-methyl-1,3-dihydrobenzo[*c*]isoxazole **(68)**	S128	S373-375
1-(2((2-Iodobenzyl)amino)phenyl)ethan-1-one **(69)**	S128	S376
2-Allyl-5-bromoindoline (**S9**)	S101	S377-381
2-Allyl-5-bromo-1-((2-nitro-4-(trifluoromethyl)phenyl)sulfonyl)indoline (**9a**)	S102	S382-383
5-Bromo-1-((2-nitro-4-(trifluoromethyl)phenyl)sulfonyl)-2-(2-(triethylsilylperoxy)propyl)indoline (**9b**)	S103	S384-385
5-Bromo-2-(2-(*tert*-Butyldiphenylsilylperoxy)propyl)-1-((2-nitro-4-(trifluoromethyl)phenyl)sulfonyl)indoline (**70** and **71**)	S104	S386-388
exo-6-Bromo-2-methyl-2,3,3a,4-tetrahydroisoxazolo[2,3-*a*]indole **(72)**	S129	S389-393
endo-6-Bromo-2-methyl-2,3,3a,4-tetrahydroisoxazolo[2,3-*a*]indole (**73**)	S129	S394-398

N-(4-Hydroxybutyl)-*N*-(naphthalen-1-ylmethyl)-2-nitro-4-(trifluoromethyl)benzenesulfonamide (**3l**)	S106	S399-400
N-(4-Iodobutyl)-*N*-(naphthalen-1-ylmethyl)-2-nitro-4-(trifluoromethyl)benzenesulfonamide (**3m**)	S107	S401-402
N-(4-Hydroperoxybutyl)-*N*-(naphthalen-1-ylmethyl)-2-nitro-4-(trifluoromethyl)benzenesulfonamide (**3n**)	S108	S403-404
N-(4-(*tert*-Butyldiphenylsilylperoxy)butyl)-*N*-(naphthalen-1-ylmethyl)-2-nitro-4-(trifluoromethyl)benzenesulfonamide (**74**)	S109	S405-406
2-(Naphthalen-1-ylmethyl)-1,2-oxazinane **(76)**	S130	S407-410
N-(Naphthalen-1-ylmethyl)-2-nitro-*N*-(pent-4-en-1-yl)-4-(trifluoromethyl)benzenesulfonamide **(3o)**	S132	S411-412
N-(5-Hydzroxypentyl)-*N*-(naphthalen-1-ylmethyl)-2-nitro-4-(trifluoromethyl)benzenesulfonamide **(3p)**	S133	S413-414
N-(5-Iodopentyl)-*N*-(naphthalen-1-ylmethyl)-2-nitro-4-(trifluoromethyl)benzenesulfonamide **(3q)**	S134	S415-416
N-(5-Hydroperoxypentyl)-*N*-(naphthalen-1-ylmethyl)-2-nitro-4-(trifluoromethyl)benzenesulfonamide **(3r)**	S135	S417-418
N-(5-((*tert*-Butyldiphenylsilyl)peroxy)pentyl)-*N*-(naphthalen-1-ylmethyl)-2-nitro-4-(trifluoromethyl)benzenesulfonamide **(75)**	S136	S419-420
1-Naphthaldehyde (**78**)	S137	-
2-((2-Nitro-4-(trifluoromethyl)phenyl)thio)ethan-1-ol (**82**)	-	S421-422

1) General Experimental

Experimental. All reactions were conducted in flame/oven dried glassware capped with rubber septa under an atmosphere of argon or nitrogen unless otherwise stated. Commercially available starting materials were used without purification, unless otherwise stated. All organic solutions were concentrated under reduced pressure on a rotary evaporator and water bath. Flash column chromatography was performed using silica gel (Sorbent Tech Purity Flash Cartridges Granular Silica Gel 60 Å, 40-75µm) on a Teledyne Isco COMBIFLASH® NextGen system, unless otherwise stated. Thin-layer chromatography (TLC) was carried out with 250 µm glass backed silica (XHL) plates with fluorescent indicator (254 nm). TLC plates were visualized by exposure to ultraviolet light (UV) and/or submersion in aqueous potassium permanganate, *p*-anisaldehyde in ethanol or ninhydrin solution followed by heating on a hot plate (120 °C, 10-15 s).

Materials. Commercial solvents and reagents were used as received without further purification.

Instrumentation. ^{1}H and ^{13}C Nuclear magnetic resonance (NMR) spectra of all compounds were obtained at 500 and 126 MHz, respectively in $CDCl_3$ (δ_H 7.26 and δ_C 77.16), or C_6D_6 (δ_H 7.16 and δ_C 77.23). ^{19}F NMR spectra were obtained at 470 MHz and are reported in terms of chemical shift (δ ppm), multiplicity, coupling constant (Hz) and integration The chemical shifts (δ) were calculated with respect to residual solvent peak and are given in ppm. Multiplicities are abbreviated as follows: s (singlet), m (multiplet), br (broad), d (doublet), t (triplet), q (quartet), p (pentet), dd (doublet of doublets) and dq (doublet of quartets). Assignments were made with the help of COSY, NOESY HMBC, and HSQC spectra. High-resolution mass spectra were obtained on a ThermoFisher Orbitrap Q-Exactive using electrospray ionization (ESI).

Hazard statement. Although no safety issues were encountered in the course of this work, all work with peroxides should be performed behind an appropriately sized explosion shield with an awareness of the potential for spontaneous and exothermic decomposition.[1, 2]

2) General Procedures

General procedure 1A: Sulfonamide protection of aliphatic amines[3]

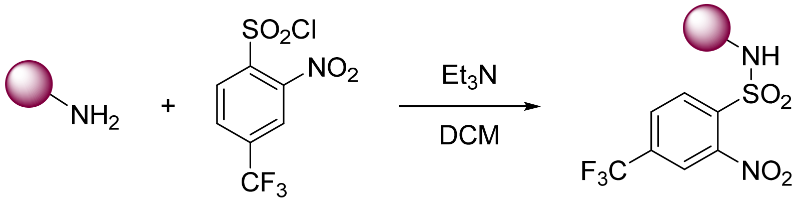

To a stirred solution of primary amine (1 eq) in dry DCM (0.2 M) at room temperature, 2-nitro-4-(trifluoromethyl)benzenesulfonyl chloride (1.2 eq) was added portion-wise, followed by drop-wise addition of triethylamine (1.2 eq). This mixture was stirred for 2-4 h. Upon full consumption of the primary amine, the reaction mixture was diluted with DCM and quenched by the addition of a saturated aqueous solution of NH_4Cl. The resulting biphasic mixture was transferred to a separatory funnel and the layers separated. The aqueous layer was extracted with DCM (3 x 15 mL per mmol) and the organic layers were combined, dried over Na_2SO_4, filtered, and concentrated *in vacuo*. The crude residue was purified by flash column chromatography on silica gel.

General procedure 1B: Sulfonamide protection of aromatic amines

To a stirred solution of aromatic amine (1.5 eq) in dry DCM (0.2 M) at 0 °C, 2-nitro-4-(trifluoromethyl)benzenesulfonyl chloride (1 eq) was added portion-wise over 10 minutes. The mixture was stirred for 10 min, followed by the drop-wise addition of triethylamine (1 eq). This mixture was stirred for 2-4 h. Upon full consumption of 2-nitro-4-(trifluoromethyl)benzenesulfonyl chloride via TLC, the reaction mixture was diluted with DCM and quenched by the addition of a saturated aqueous solution of NH_4Cl. The resulting biphasic mixture was transferred to a separatory funnel and the layers separated. The aqueous layer was

extracted with DCM (3 x 15 mL per mmol) and the organic layers were combined, dried over Na_2SO_4, filtered, and concentrated *in vacuo*. The crude residue was purified by flash column chromatography on silica gel.

General procedure 2A: Base mediated sulfonamide alkylation

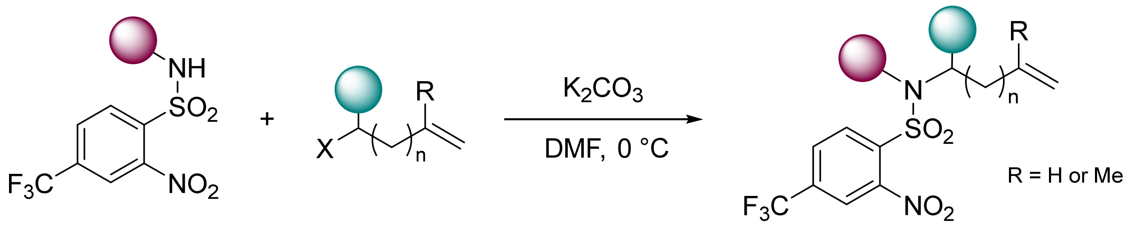

To a stirred solution of sulfonamide (1.0 eq) in dry DMF (0.2 M) was added K_2CO_3 (1.5 eq), followed by the alkyl halide (1.5 eq). The solution was heated to 60 °C and stirred for 8-12 h. After such time, the mixture was quenched with H_2O and extracted with EtOAc (3 x 15 mL per mmol). The combined organics were dried over Na_2SO_4 and concentrated *in vacuo*. The resulting residue was purified by flash column chromatography on silica gel.

General procedure 2B: Sulfonamide-Mitsunobu alkylation

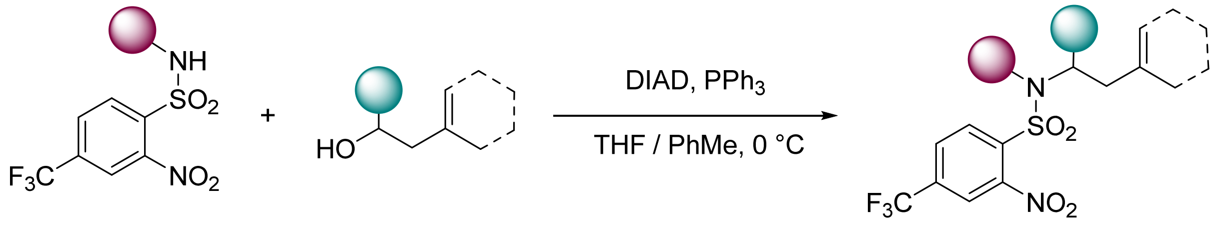

Following the literature protocol,[4] the sulfonamide (1 eq) was suspended in a 1:1 (v:v) ratio of THF and toluene (0.2 M), and cooled to 0 °C, followed by addition of the alcohol (3 eq) and triphenyl phosphine (3 eq). This mixture was stirred for 10 min, at which time diisopropyl azodicarboxylate (3 eq) was added drop-wise over 10 min. The solution was then allowed to warm to room temperature and stir for 12-16 h. Upon full consumption of the sulfonamide (via TLC) the

mixture was quenched with H_2O and extracted with EtOAc (3 x 15 mL per mmol). The combined organic layers were washed with brine, dried over Na_2SO_4, and concentrated *in vacuo*. The crude residue was purified by flash column chromatography on silica gel.

General procedure 3: Hydroboration-oxidation

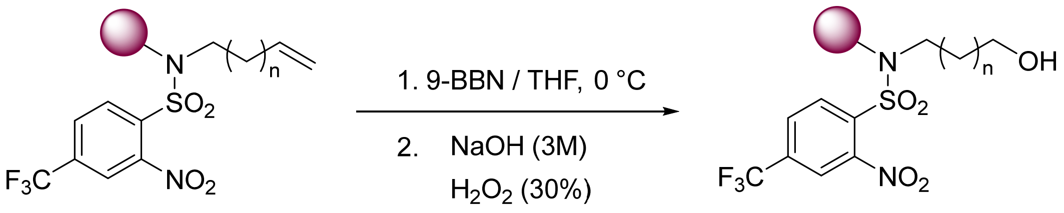

To a solution of alkene (1 eq) in dry THF (0.4 M) at 0 °C was added 9-BBN 0.5 M in THF (2.0 eq) drop-wise over 15-min. The solution was slowly warmed to RT and stirred for 3 h. Upon full consumption of the alkene (via TLC and MS), the mixture was cooled to 0 °C and carefully treated with NaOH (3 M, 1.2 eq) and aq. H_2O_2 (30%, 1.2 eq). The solution was then allowed to warm to RT and stirred for 1 h, after which time it was treated with saturated aqueous $NaHCO_3$. The layers were separated, and the aqueous layer extracted with EtOAc (3 x 15 mL per mmol). The combined organic layers were dried over Na_2SO_4 and concentrated *in vacuo*. The crude residue obtained was purified by flash column chromatography on silica gel.

General procedure 4: Iodination

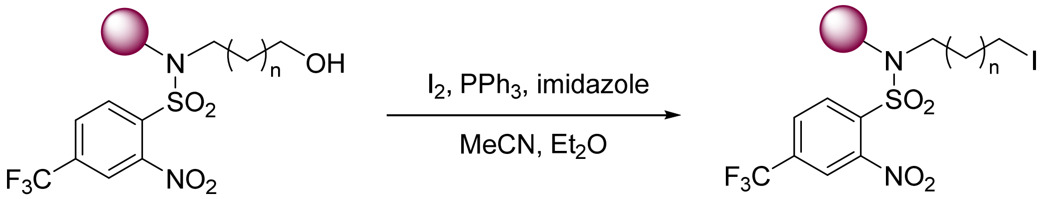

Triphenylphosphine (1.5 eq), imidazole (1.5 eq) and iodine (1.5 eq) were added subsequently to a solution of alcohol (1 eq) in a 1:3 (v:v) mixture of MeCN and Et_2O (0.2 M) at RT. The resulting

mixture was stirred for 1-3 h. Upon full consumption of the alcohol, the reaction was quenched by addition of saturated aqueous $NaHCO_3$. The biphasic mixture was separated, and the aqueous layer extracted with EtOAc (3 x 15 mL per mmol). The combined organic phases were washed with saturated aqueous $Na_2S_2O_3$, dried over Na_2SO_4, and concentrated *in vacuo*. The resulting crude residue was purified by flash column chromatography on silica gel.

General procedure 5: Formation of hydroperoxides from iodides[5]

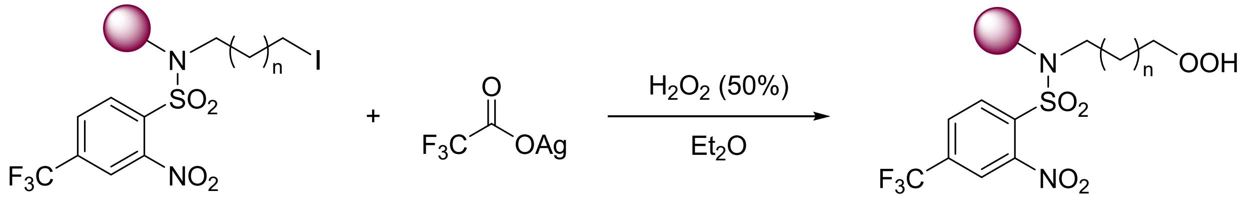

Silver trifluoroacetate (1.2 eq) was added in small portions to an ice-cooled stirred solution of alkyl iodine (1 eq) in diethyl ether (0.2 M) with stirring over 5 min. Aqueous 50% hydrogen peroxide (2 eq) was then added slowly drop-wise and the mixture stirred for 1–3 h. Upon full consumption of the alkyl iodide (via TLC), the mixture was filtered and the filtrate separated into phases. The organic phase was washed with saturated aqueous $NaHCO_3$, washed with brine, dried over Na_2SO_4, and concentrated *in vacuo*. The crude residue was purified by flash column chromatography on silica gel.

General procedure 6A: Alkene silyl peroxidation

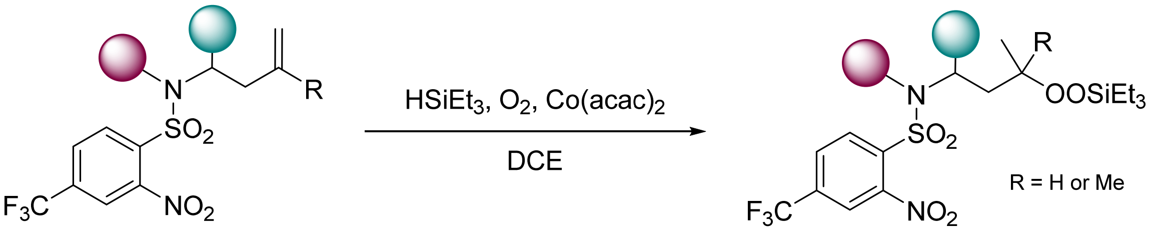

Following the literature protocol,[6] a stirred solution of alkene (1 eq) in DCE (0.2 M) was treated at room temperature with triethylsilane (2.5 eq), followed by Co(acac)$_2$ (10 mol%), after which the vessel was sealed under an oxygen atmosphere (1 atm, balloon) and stirred at RT for 18-24 h. Upon full consumption of the alkene (via TLC and MS), the reaction mixture was concentrated *in vacuo* and directly purified by flash column chromatography on silica gel.

Bis(2,2,6,6-tetramethyl-3,5-heptanedionato)cobalt(II) (Co(thd)$_2$) (S10)

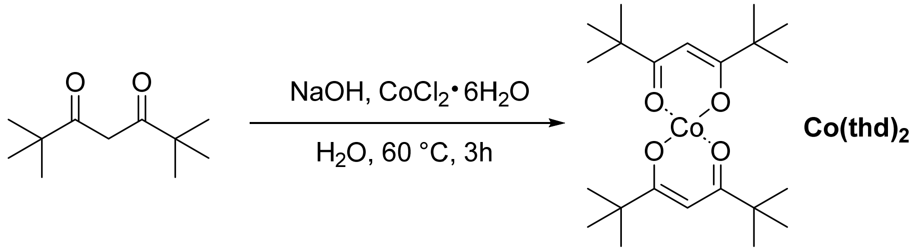

S10

Following a modified literature protocol,[7] a stirred solution of 2,2,6,6-tetramethylheptane-3,5-dione (1.0 mL, 4.8 mmol, 1 eq) in H_2O (50 mL, 0.1 M) was treated with NaOH (192 mg, 4.8 mmol, 1 eq). The mixture was heated to 60 °C and stirred for 10 min. Then $CoCl_2 \cdot 6H_2O$ was added portion wise (570 mg, 2.4 mmol, 0.5 eq) and the mixture stirred for 3 h, during which time the color shifted from royal blue, to lavender, to brown, and finally to dusty pink. The solution was filtered and washed with H_2O (3 x 100 mL) to obtain the title compound **S10** as a pink solid (1.01 g, 2.36 mmol, 49%).

HRMS-ESI (m/z): $[M+H]^+$ calculated for $[C_{22}H_{39}O_4Co]^+$: 426.2175, found 426.2154.

General procedure 6B: Styrene silyl peroxidation

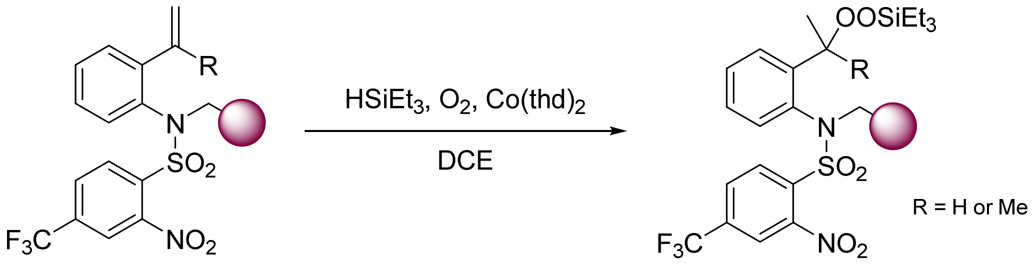

Following the literature protocol,[7] a solution of alkene in DCE (0.2 M) was treated with triethylsilane (2.5 eq), followed by $Co(thd)_2$ (10 mol%), after which the vessel was sealed under an oxygen atmosphere (1 atm, balloon) and stirred at RT for 4-6 h. Upon full consumption of the alkene, the reaction mixture was concentrated under reduced pressure, and directly purified by flash column chromatography on silica gel.

Note: Amino styrenes when exposed to ninhydrin followed by heating on TLC are identifiable as green spots.

General procedure 7: Silyl group exchange

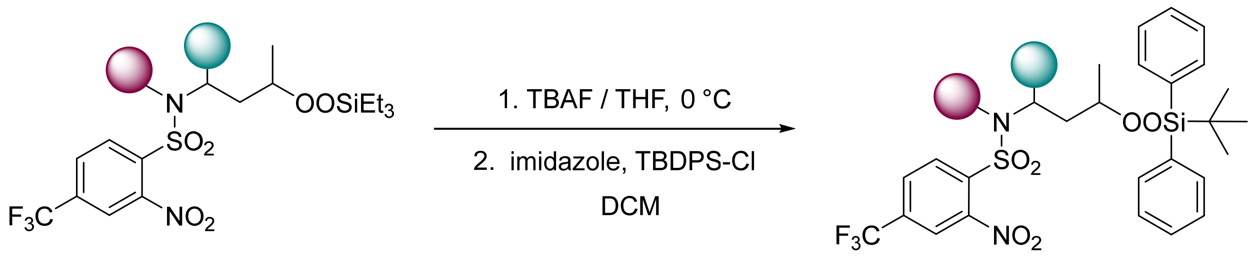

Following the literature protocol,[8] TBAF (1.0 M in THF 1.2 eq) was added slowly to a stirred solution of triethylsilyl peroxide in THF (0.1 M) at 0 °C, followed by stirring for 15 to 30 min. Upon completion, the mixture was quenched with H_2O and extracted with EtOAc (3 x 15 mL per mmol). The combined organic phases were washed with brine, dried over Na_2SO_4 and concentrated *in vacuo*. The residue was then dissolved in DCM (0.2 M), treated with imidazole

(1.5 eq), TBDPS-Cl (1 eq), and stirred for 2-4 h at RT. Upon completion (via TLC and MS), the reaction mixture was quenched with H_2O and extracted with DCM (3 x 15 mL per mmol). The combined organic phases were washed with brine, dried over Na_2SO_4, concentrated *in vacuo* and purified by flash column chromatography on silica gel.

General procedure 8: TBDPS protection of hydroperoxides

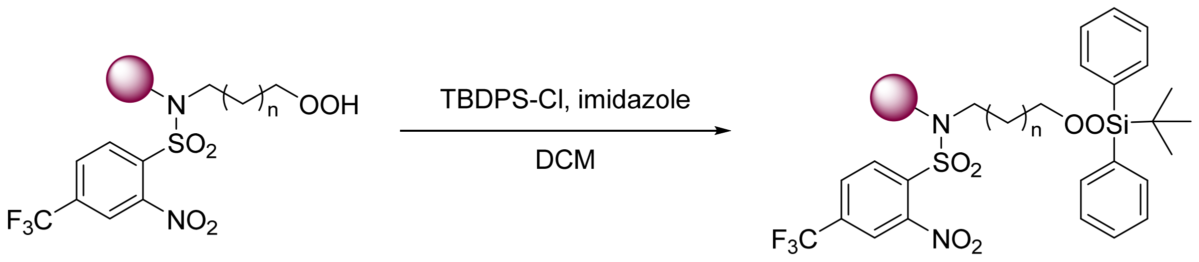

Primary hydroperoxides in DCM (0.2 M) at room temperature, were treated with imidazole (1.5 eq), TBDPS-Cl (1 eq) and stirred for 2-4 h. Upon full consumption of the hydroperoxide (via TLC and MS), the mixture was quenched with H_2O and extracted with DCM (3 x 15 mL per mmol). The combined organic phases were washed with brine, dried over Na_2SO_4 and concentrated *in vacuo* and purified by flash column chromatography on silica gel.

Procedure 9A: Deprotection/cyclization of primary silyl peroxides

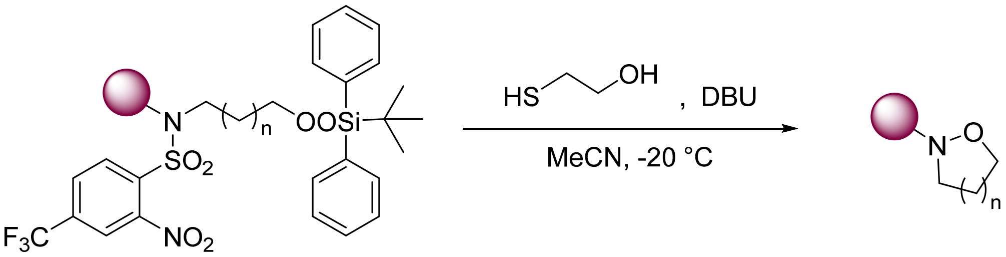

The substrate (1 eq) was stirred in dry MeCN (0.2 M), brought to -20 °C, and treated with a solution of 2-mercaptoethanol (1 eq) and DBU (1 eq) in dry MeCN (1 M). The mixture was stirred for 30 s, and then quenched with H_2O and extracted with DCM (3 x 15 mL per mmol). The combined organic phases were washed with brine, dried over Na_2SO_4 and concentrated *in vacuo* and the crude residue purified by flash column chromatography on silica gel.

Procedure 9B: Deprotection/cyclization of secondary silyl peroxides

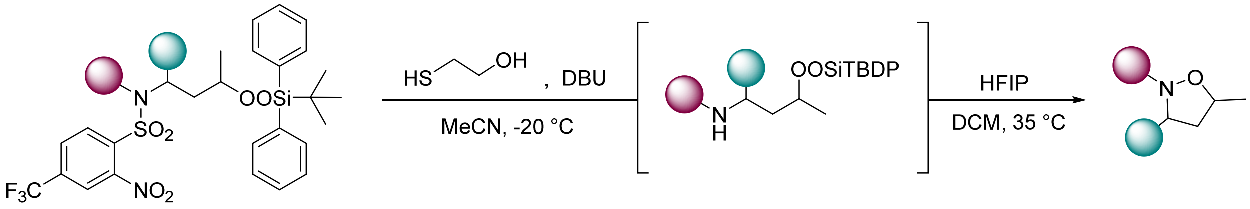

The substrate (1 eq) was stirred in dry MeCN (0.2 M), brought to -20 °C, and treated with a solution of 2-mercaptoethanol (1 eq) and DBU (1 eq) in dry MeCN (1 M). The mixture was stirred for 30 s, and quenched with H_2O and extracted with DCM (3 x 15 mL per mmol). The combined organic phases were washed with brine, dried over Na_2SO_4 and concentrated *in vacuo*. The crude residue was taken up in DCM (0.2 M), treated dropwise with hexafluoroisopropanol (1 eq), and then heated with stirring at 35 °C for 0.5 to 2 h until full cyclization. The crude residue was then concentrated under reduced pressure and purified by flash column chromatography on silica gel.

Procedure 9C: Deprotection/cyclization of benzylic triethylsilyl peroxides

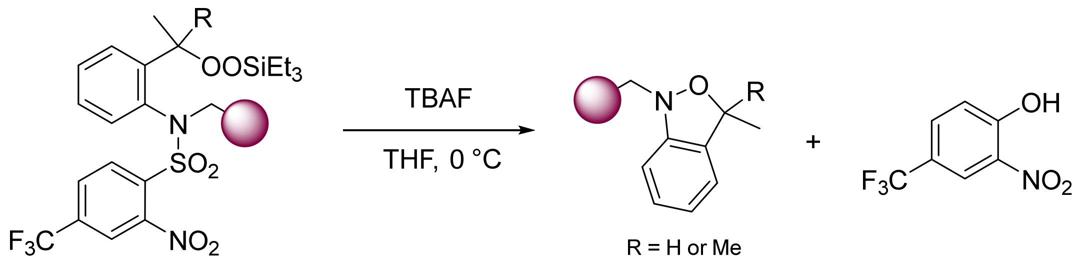

A stirred solution of substrate (1 eq) in THF (0.2 M) at 0 °C was treated with TBAF 1.0 M in THF (1.2 eq) drop-wise over 10 min. Upon full consumption (via TLC and MS), the mixture was quenched with H_2O and extracted with EtOAc (3 x 15 mL per mmol). The combined organic phases were washed with brine, dried over Na_2SO_4 and concentrated *in vacuo* and the products were isolated after purification by flash column chromatography on silica gel.

Note: During purification, all cyclized products co-elute with the by-product (2-nitro-4-(trifluoromethyl)phenol) unless purified with a 1% Et_3N in hexanes and EtOAc gradient. The inclusion of Et_3N in the eluent allows for easy isolation of the cyclized product, while leaving the by-product at the top of the column as a yellow band. When isolation of the by-product is required, basic extraction of the crude reaction mixture transfers the phenol into the aqueous layer from which it can be extracted following acidification, while leaving the cyclized product in the organic layer.

General note on isoxazolidines: All cyclic hydroxylamines synthesized were easily identifiable on TLC when run with a basified solvent gradient (1% Et_3N in hexanes typically). The spots would appear as flat, dark slits as opposed to circular.

Procedure 9D: Desulfonylation of tertiary silyl peroxides

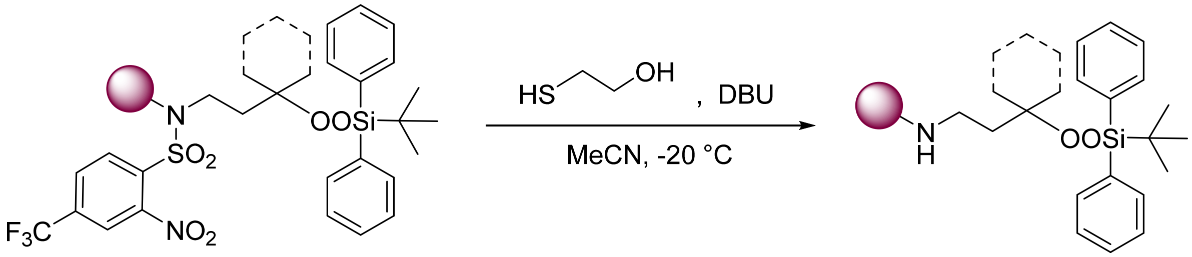

A stirred solution of TBDPS peroxide (1 eq) in dry MeCN (0.2 M) was cooled to -20 °C, treated with a solution of 2-mercaptoethanol (1 eq) and DBU (1 eq) in MeCN (1 M) and stirred for 5 min. The mixture was then quenched with H_2O and extracted with EtOAc (3 x 15 mL per mmol). The combined organic phases were washed with brine, dried over Na_2SO_4, concentrated *in vacuo*, and the crude product was purified by flash column chromatography on silica gel.

Procedure 10: Cyclization of tertiary silyl peroxy amines

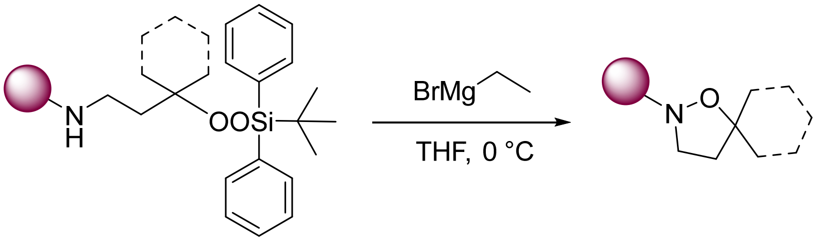

To a stirred solution of substrate (1 eq) in anhydrous THF (0.2 M) under an argon atmosphere at 0 °C was added dropwise EtMgBr (3 M in diethyl ether, 4 eq). The ice bath was removed, and the reaction mixture was allowed to slowly rise to RT and stirred for 1-2 h. After completion, the reaction mixture was quenched with H_2O and diluted in EtOAc. The biphasic mixture was separated, and the organic layer washed with $NaHCO_3$, followed by brine. The organic layer was then dried over Na_2SO_4, concentrated *in vacuo,* and the crude product was purified by flash column chromatography on silica gel.

3) Synthesis and Characterization of Substrates for Protecting Group Screen

***N*-(Naphthalen-1-ylmethyl)-4-nitrobenzenesulfonamide (S1)**

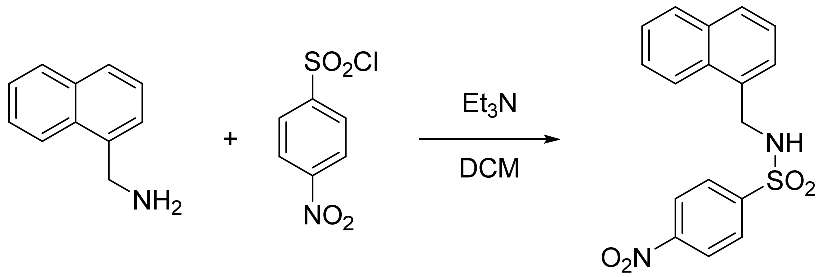

S1

Following general procedure 1A, 1-naphthalylmethylamine (2.0 mL, 13.62 mmol, 1 eq) in dry DCM (27 mL, 0.5 M) was treated with triethylamine (1.45 mL, 16.62 mmol, 1.2 eq) and 4-nitrobenzenesulfonyl chloride (3.32 g, 15 mmol, 1.2 eq). The crude residue was purified by flash column chromatography on silica gel (eluent: 75:25 hexanes: EtOAc) to obtain the title compound **S1** (4.1 g, 12 mmol, 88%) as an off white solid.

TLC: R*f* = 0.17 (80:20 hexanes: EtOAc).

^{1}H NMR (500 MHz, CDCl$_3$): δ 8.15 (d, *J* = 8.2 Hz, 2H, ArH), 7.86 (d, *J* = 7.3 Hz, 2H, ArH), 7.85 (s, 1H, ArH), 7.80 (d, *J* = 6.8 Hz, 1H, ArH), 7.76 (d, *J* = 7.7 Hz, 1H, ArH), 7.52 – 7.47 (m, 2H, ArH), 7.38 – 7.28 (m, 2H, ArH), 4.93 (br t, *J* = 5.8 Hz, 1H, NH), 4.68 (d, *J* = 5.6 Hz, 2H, ArCH$_2$).

^{13}C NMR (126 MHz, CDCl$_3$): δ 145.9, 139.0, 134.0, 131.1, 130.7, 129.6, 129.1, 128.3, 127.7, 127.1, 126.4, 125.3, 124.1, 122.9, 45.9.

HRMS-ESI (m/z): [M+Na]$^+$ calculated for [$C_{17}H_{14}N_2O_4SNa$]$^+$: 365.0572, found 365.0550.

N-(3-Methylbut-3-en-1-yl)-N-(naphthalen-1-ylmethyl)-4-nitrobenzenesulfonamide (10)

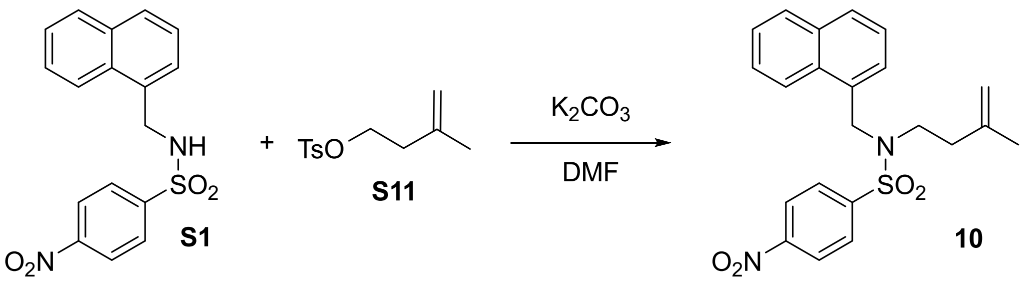

Following general procedure 2A, a stirred solution of **S1** (890 mg, 2.6 mmol, 1.0 eq) in DMF (13 mL, 0.2 M) was treated with K_2CO_3 (538 mg, 3.9 mmol, 1.5 eq), followed by **S11** (936 mg, 3.9 mmol, 1.5 eq). The crude residue was purified by flash column chromatography on silica gel (eluent: 93:7 hexanes: EtOAc) to obtain the title compound **10** (938 mg, 2.3 mmol, 88%) as a white solid.

TLC: R_f = 0.21 (90:10 hexanes: EtOAc).

^{1}H NMR (500 MHz, $CDCl_3$): δ 8.30 (d, J = 16.0 Hz, 2H, ArH), 8.21 (d, J = 17.0 Hz, 1H, ArH), 8.00 (d, J = 15.9 Hz, 2H, ArH), 7.88 – 7.79 (m, 2H, ArH), 7.60 – 7.47 (m, 2H, ArH), 7.43 – 7.33 (m, 2H, ArH), 4.83 (s, 2H, $ArCH_2$), 4.60 (s, 1H, =CH), 4.41 (s, 1H, =CH), 3.23 (t, J = 8.8 Hz, 2H, CH_2), 1.88 (t, J = 8.6 Hz, 2H, CH_2), 1.46 (s, 3H, CH_3).

^{13}C {^{1}H, ^{19}F} NMR (126 MHz, $CDCl_3$): δ 150.0, 145.2, 141.8, 133.9, 131.8, 130.3, 129.6, 128.9, 128.5, 127.9, 126.9, 126.3, 125.1, 124.4, 123.6, 112.4, 51.1, 47.4, 36.9, 22.2.

HRMS-ESI (m/z): $[M+Na]^+$ calculated for $[C_{22}H_{22}N_2O_4SNa]^+$: 433.1198, found 433.1196.

***N*-(3-Methyl-3-((triethylsilylperoxy)butyl)-*N*-(naphthalene-1-ylmethyl)-4-nitrobenzenesulfonamide (16)**

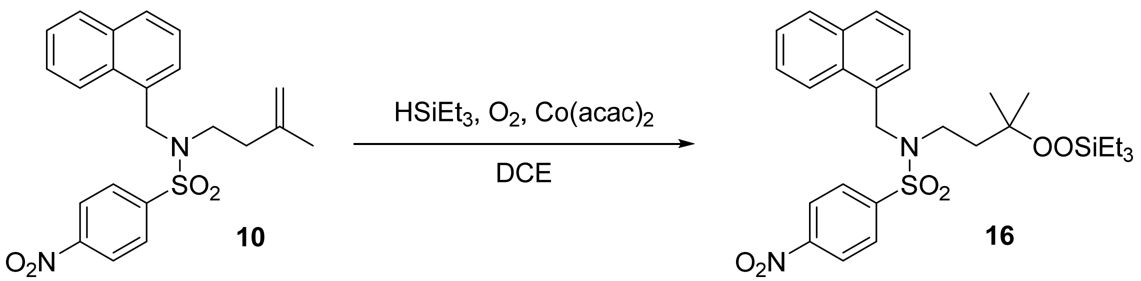

Following general procedure 6A, **10** (900 mg, 2.19 mmol, 1 eq) in DCE (11 mL, 0.2 M) was treated with triethylsilane (0.81 mL, 5.49 mmol, 2.5 eq), and $Co(acac)_2$ (56 mg, 0.22 mmol, 10 mol%) under an atmosphere of oxygen. The crude residue was purified by flash column chromatography on silica gel (eluent: 95:5 hexanes: EtOAc) to obtain the title compound **16** (915 mg, 1.64 mmol, 75%) as a colorless oil.

TLC: R_f = 0.41 (90:10 hexanes: EtOAc).

^{1}H NMR (500 MHz, C_6D_6): δ 8.28 (d, *J* = 8.8 Hz, 2H, ArH), 8.19 (d, *J* = 8.3 Hz, 1H, ArH), 7.98 (d, *J* = 8.8 Hz, 2H, ArH), 7.84 (d, *J* = 9.5 Hz, 1H, ArH), 7.80 (dd, *J* = 6.3, 3.3 Hz, 1H, ArH), 7.58 – 7.47 (m, 2H, ArH), 7.41 – 7.34 (m, 2H, ArH), 4.84 (s, 2H, $ArCH_2$), 3.32 – 3.25 (m, 2H, CH_2), 1.59 – 1.52 (m, 2H, CH_2), 0.95 (s, 6H, $(CH_3)_2$), 0.87 (t, *J* = 7.9 Hz, 9H, $SiCH_2CH_3$), 0.54 (q, *J* = 8.0 Hz, 6H, $SiCH_2CH_3$).

^{13}C NMR (126 MHz, C_6D_6): δ 150.0, 145.6, 133.9, 131.8, 130.4, 129.4, 128.9, 128.5, 127.7, 126.9, 126.3, 125.1, 124.3, 123.6, 80.8, 50.6, 44.2, 36.8, 24.4, 6.8, 3.9.

HRMS-ESI (m/z): $[M+Na]^+$ calculated for $[C_{28}H_{38}N_2O_6SSiNa]^+$: 581.2118, found 581.2104.

***N*-(But-3-en-1-yl)-*N*-(naphthalen-1-ylmethyl)-4-nitrobenzenesulfonamide (11)**

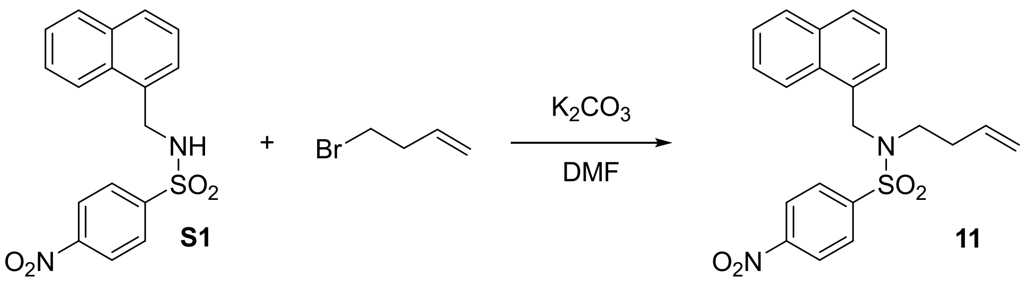

Following general procedure 2A, a stirred solution of **S1** (1.3 g, 3.8 mmol, 1 eq) in DMF (15 mL, 0.2 M) was treated with K_2CO_3 (1.05 g, 7.6 mmol, 2 eq), followed by 5-bromobut-1-ene (0.58 mL, 5.7 mmol, 1.5 eq). The crude residue was purified by flash column chromatography on silica gel (eluent: 92:8 hexanes: EtOAc) to obtain the title compound **11** (1.37 g, 3.38 mmol, 89%) as an iridescent white solid.

TLC: R_f = 0.19 (90:10 hexanes: EtOAc).

^{1}H NMR (500 MHz, $CDCl_3$): δ 8.31 (d, *J* = 8.9 Hz, 2H, ArH), 8.18 (d, *J* = 8.4 Hz, 1H, ArH), 8.00 (d, *J* = 6.9 Hz, 2H, ArH), 7.85 (d, *J* = 7.6 Hz, 1H, ArH), 7.83 (d, *J* = 8.0 Hz, 1H, ArH), 7.59 – 7.47 (m, 2H, ArH), 7.42 – 7.33 (m, 2H, ArH), 5.43 (ddt, *J* = 17.1, 10.2, 6.8 Hz, 1H, C*H*=H2), 4.85 (s,1H, =C*H*), 4.82 (s, 2H, $ArCH_2$), 4.75 (d, *J* = 17.2 Hz, 1H, =C*H*), 3.21 (t, *J* = 15.5 Hz, 2H, CH_2), 1.94 (q, *J* = 6.9 Hz, 2H, CH_2).

^{13}C NMR (126 MHz, $CDCl_3$): δ 150.0, 145.3, 134.1, 134.0, 131.8, 130.4, 129.6, 128.9, 128.5, 127.8, 127.0, 126.4, 125.2, 124.4, 123.6, 117.4, 51.2, 48.2, 33.3.

HRMS-ESI (m/z): $[M+Na]^+$ calculated for $[C_{21}H_{20}N_2O_4SNa]^+$: 419.1041, found 419.1025.

***N*-(Naphthalene-1-ylmethyl)-4-nitro-*N*-(3-(triethylsilylperoxy)butyl)benzenesulfonamide (17)**

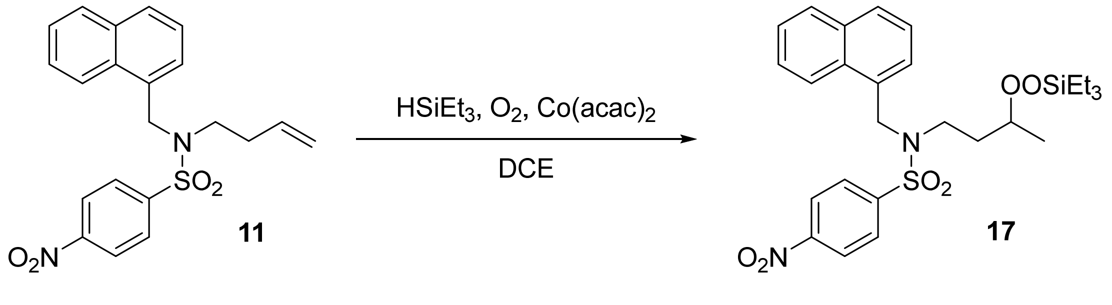

Following general procedure 6A, **11** (497 mg, 1.25 mmol, 1 eq) in DCE (15 mL, 0.2 M) was treated with triethylsilane (0.5 mL, 3.14 mmol, 2.5 eq), and $Co(acac)_2$ (32 mg, 0.13 mmol, 10 mol%) under an atmosphere of oxygen. The crude residue was purified by flash column chromatography on silica gel (eluent: 87:12 hexanes: EtOAc) to obtain the title compound **17** (137 mg, 0.51 mmol, 41%) as a colorless oil.

TLC: R_f = 0.41 (90:10 hexanes: EtOAc).

^{1}H NMR (500 MHz, C_6D_6): δ 8.42 (d, *J* = 8.5 Hz, 1H, ArH), 7.65 (d, *J* = 7.6 Hz, 2H, ArH), 7.59 (d, *J* = 8.2 Hz, 1H, ArH), 7.56 (d, *J* = 7.8 Hz, 1H, ArH), 7.51 (d, *J* = 7.6 Hz, 2H, ArH), 7.35 (t, *J* = 7.4 Hz, 1H, ArH), 7.23 (t, *J* = 7.5 Hz, 1H, ArH), 7.19 (d, *J* = 6.9 Hz, 1H, ArH), 7.13 (d, *J* = 8.2 Hz, 1H, ArH), 4.63 (d, *J* = 14.1 Hz, 1H, ArCH), 4.50 (d, *J* = 14.1 Hz, 1H, ArCH), 3.70 (h, *J* = 5.8 Hz, 1H, CH), 3.22 (ddd, *J* = 15.6, 10.5, 5.4 Hz, 1H, CH_2), 3.15 (ddd, *J* = 15.5, 10.4, 5.5 Hz, 1H, CH_2), 1.54 (ddd, *J* = 17.0, 9.4, 5.8 Hz, 1H, CH_2), 1.40 (ddd, *J* = 15.0, 10.4, 5.1 Hz, 1H, CH_2), 0.93 (t, *J* = 8.0 Hz, 9H, $SiCH_2CH_3$), 0.80 (d, *J* = 6.2 Hz, 3H, CH_3), 0.58 (q, *J* = 7.9 Hz, 6H, $SiCH_2CH_3$).

^{13}C NMR (126 MHz, C_6D_6): δ 149.9, 145.0, 134.4, 132.3, 131.1, 129.6, 129.1, 128.4, 128.3, 127.2, 126.5, 125.2, 124.09, 124.05, 79.2, 51.3, 45.3, 33.6, 18.1, 7.0, 4.1.

HRMS-ESI (m/z): $[M+Na]^+$ calculated for $[C_{23}H_{36}N_2O_6SSiNa]^+$: 567.1961, found 567.1956.

***N*-(3-(*tert*-Butyldiphenylsilylperoxy)butyl)-*N*-(naphthalene-1-ylmethyl)-4-nitrobenzenesulfonamide (22)**

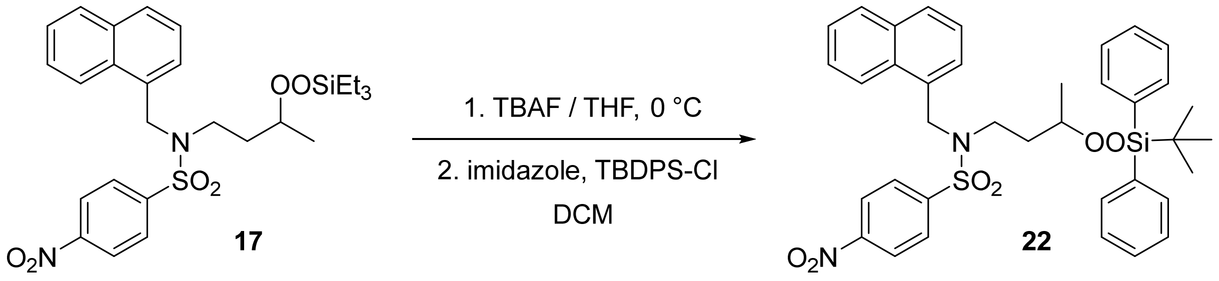

Following general procedure 7, TBAF (1.43 mL, 1.0 M in THF, 1.2 eq) was added to a solution of **17** (650 mg, 1.19 mmol, 1 eq) in THF (6.0 mL, 0.2 M). The resulting crude residue was then dissolved in DCM (6.0 mL, 0.2 M) and treated with imidazole (122 mg, 1.8 mmol, 1.5 eq) and TBDPS-Cl (0.33 mL, 1.19 mmol, 1 eq). The crude product was purified by flash column chromatography on silica gel (eluent: 90:10 hexanes: EtOAc) to obtain the title compound **22** (697 mg, 1.04 mmol, 88% over 2 steps) as a colorless oil.

TLC: R_f = 0.42 (90:10 hexanes: EtOAc).

^{1}H NMR (500 MHz, $CDCl_3$): δ 8.18 (d, *J* = 8.7 Hz, 2H, ArH), 8.15 (d, *J* = 9.5 Hz, 1H, ArH), 7.84 (d, *J* = 8.7 Hz, 2H, ArH), 7.76 (d, *J* = 8.1 Hz, 1H, ArH), 7.66 (d, *J* = 6.7 Hz, 2H, ArH), 7.57 (d, *J* = 6.7 Hz, 2H, ArH), 7.53 – 7.50 (m, 2H, ArH), 7.44 (t, *J* = 7.4 Hz, 1H, ArH), 7.38 – 7.34 (m, 2H, ArH), 7.24 (d, *J* = 6.9 Hz, 1H, ArH), 7.22 – 7.16 (m, 2H, ArH), 4.70 (d, *J* = 14.2 Hz, 1H, ArCH), 4.67 (d, *J* = 14.4 Hz, 1H, ArCH), 3.73 (td, *J* = 6.9, 4.2 Hz, 1H, CH), 3.00 (ddd, *J* = 14.7, 11.3, 5.4 Hz, 1H, CH_2), 2.82 (ddd, *J* = 16.0, 11.0, 5.4 Hz, 1H, CH_2) 1.35 (ddt, *J* = 16.1, 10.6, 5.2 Hz, 2H, CH_2), 1.03 (s, 9H, *t*Bu), 0.90 (d, *J* = 6.1 Hz, 3H, CH_3).

^{13}C NMR (126 MHz, $CDCl_3$): δ 149.9, 145.0, 135.8, 132.8, 132.6, 130.2, 130.0, 129.4, 128.8, 128.5, 127.8, 127.7, 127.6, 126.8, 126.3, 125.1, 124.2, 123.6, 79.3, 50.9, 45.0, 33.3, 27.3, 19.4, 18.2.

HRMS-ESI (m/z): $[M+Na]^+$ calculated for $[C_{37}H_{40}N_2O_6SSiNa]^+$: 691.2274, found 691.2256.

N-(Naphthalen-1-ylmethyl)-2, 4-dinitrobenzenesulfonamide (S2)

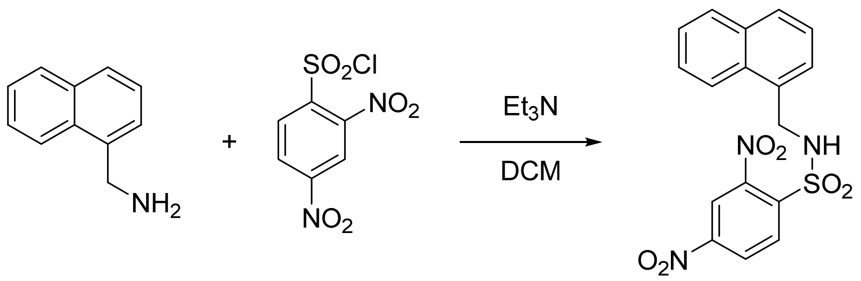

S2

Following general procedure 1A, 1-naphthalylmethylamine (0.77 mL, 5.25 mmol, 1 eq) in dry DCM (26 mL, 0.2 M) with triethylamine (0.8 mL, 5.78 mmol, 1.1 eq) was treated with 2, 4-dinitrobenzenesulfonyl chloride (1.4 g, 5.25 mmol, 1 eq). The crude residue was purified by flash column chromatography on silica gel (eluent: 88:12 hexanes: EtOAc) to obtain the title compound **S2** (1.65 g, 4.8 mmol, 92%) as a yellow solid.

TLC: R_f = 0.30 (80:20 hexanes: EtOAc).

Note: Title compound **S2** can be seen with the naked eye as a bright yellow spot on TLC.

^{1}H NMR (500 MHz, C_6D_6): δ 8.87 (s, 1H), 8.39 (br, 1H, NH), 7.66 (d, *J* = 8.2 Hz, 1H), 7.57 (d, *J* = 8.7 Hz, 2H), 7.53 (d, *J* = 7.3 Hz, 2H), 7.39 – 7.26 (m, 2H), 6.97 (d, *J* = 7.3 Hz, 1H), 5.82 (d, *J* = 9.5 Hz, 1H), 4.01 (s, 2H, $ArCH_2$).

^{13}C NMR (126 MHz, C_6D_6): δ 147.8, 136.8, 134.4, 131.3, 131.1, 129.8, 129.5, 129.1, 126.9, 126.5, 125.6, 124.6, 124.0, 122.6, 113.8, 113.5, 44.8.

HRMS-ESI (m/z): $[M+Na]^+$ calculated for $[C_{17}H_{13}N_3O_6SNa]^+$: 410.0423, found 410.0402.

***N*-(But-3-en-1-yl)-*N*-(naphthalen-1-ylmethyl)-2, 4-dinitro-4-benzenesulfonamide (12)**

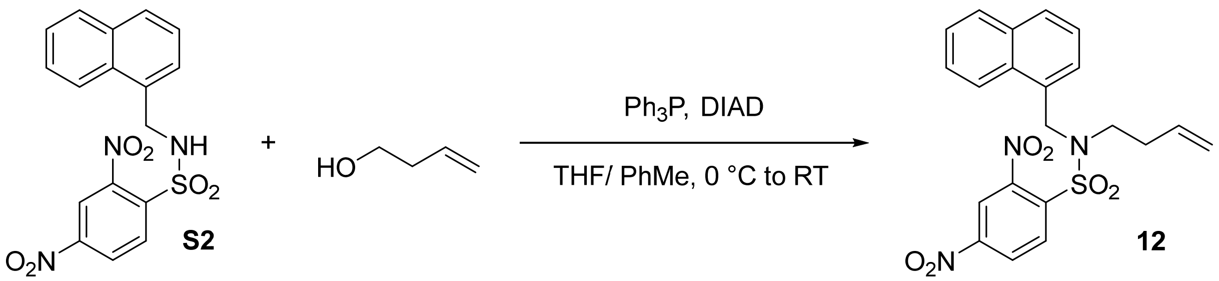

Following general procedure 2B, a solution of **S2** (1.65 g, 4.26 mmol, 1 eq) in a 1:1 mixture of THF and toluene (21 mL, 0.2 M) was treated with but-3-en-1-ol (1.1 mL, 12.78 mmol, 3 eq), triphenyl phosphine (3.35 g, 12.8 mmol, 3 eq) and DIAD (2.51 mL, 12.8 mmol, 3 eq). The crude residue was purified by flash column chromatography on silica gel (eluent: 90:10 hexanes: EtOAc) to obtain the title compound **12** (977 mg, 2.22 mmol, 52%) as a white crystalline solid.

TLC: R_f = 0.36 (90:10 hexanes: EtOAc).

^{1}H NMR (500 MHz, CDCl$_3$): δ 8.25 (s, 1H, ArH), 8.05 (d, *J* = 8.7 Hz, 1H, ArH), 7.97 (d, *J* = 8.2 Hz, 1H, ArH), 7.77 (d, *J* = 7.7 Hz, 1H, ArH), 7.74 (d, *J* = 8.6 Hz, 2H, ArH), 7.50 – 7.42 (m, 2H, ArH), 7.41 – 7.37 (m, 2H, ArH), 5.71 – 5.58 (m, 1H, C*H*=CH$_2$), 5.03 (s, 1H, =C*H*), 5.00 (d, *J* = 3.9 Hz, 1H, =C*H*), 4.98 (s, 2H, ArCH$_2$), 3.61 – 3.52 (m, 2H, CH$_2$), 2.28 (q, *J* = 7.1 Hz, 2H, CH$_2$).

^{13}C NMR (126 MHz, CDCl$_3$): δ 151.2, 139.1, 133.9, 133.8, 132.4, 131.6, 130.2, 129.5, 128.8, 128.4, 127.0, 126.4, 125.34, 125.31, 123.2, 121.8, 119.5, 118.0, 50.9, 49.5, 33.3.

HRMS-ESI (m/z): $[M+Na]^+$ calculated for $[C_{21}H_{19}N_3O_6SNa]^+$: 464.0892, found 464.0892.

***N*-(Naphthalene-1-ylmethyl)-2, 4-dinitro-*N*-(3-(triethylsilylperoxy)butyl)-benzenesulfonamide (18)**

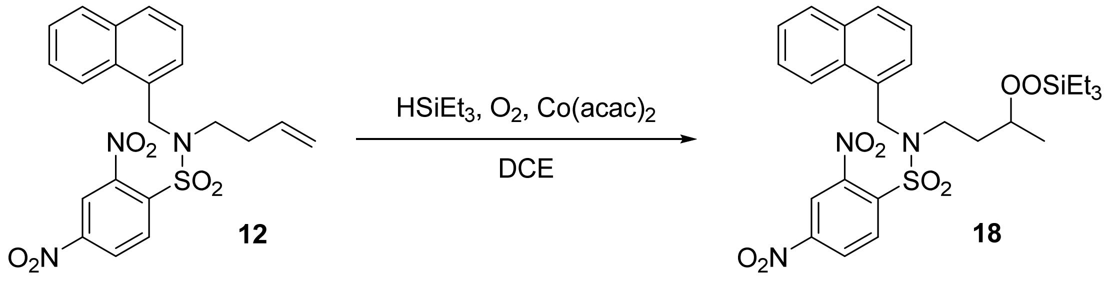

Following general procedure 6A, **12** (700 mg, 1.59 mmol, 1 eq) in DCE (15 mL, 0.2 M) was treated with triethylsilane (1.36 mL, 3.97 mmol, 2.5 eq), and $Co(acac)_2$ (41 mg, 0.16 mmol, 10 mol%) under an atmosphere of oxygen. The crude residue was then purified by flash column chromatography on silica gel (eluent: 90:10 hexanes: EtOAc) to obtain the title compound **18** (310 g, 0.53 mmol, 33%) as a colorless oil.

TLC: R*f* = 0.38 (90:10 hexanes: EtOAc).

^{1}H NMR (500 MHz, $CDCl_3$): δ 8.28 (s, 1H, ArH), 8.07 (d, *J* = 8.7 Hz, 1H, ArH), 8.01 (d, *J* = 7.2 Hz, 1H, ArH), 7.81 – 7.72 (m, 3H, ArH), 7.50 – 7.43 (m, 3H, ArH), 7.39 (t, *J* = 7.6 Hz, 1H), 4.99 (s, 2H, $ArCH_2$), 3.95 (td, *J* = 6.4, 4.8 Hz, 1H, CH), 3.62 (ddd, *J* = 16.3, 10.8, 5.5 Hz, 1H, CH_2), 3.57 (ddd, *J* = 14.5, 10.7, 5.1 Hz, 1H, CH_2), 1.83 (ddd, *J* = 16.5, 12.9, 5.7 Hz, 1H, CH_2), 1.73 (ddt, *J* = 13.6, 10.5, 5.2 Hz, 1H, CH_2), 1.09 (d, *J* = 6.3 Hz, 3H, CH_3), 0.93 (t, *J* = 7.9 Hz, 9H, $SiCH_2CH_3$), 0.62 (q, *J* = 7.9 Hz, 6H, $SiCH_2CH_3$).

^{13}C NMR (126 MHz, $CDCl_3$): δ 149.2, 147.6, 139.0, 133.8, 132.4, 131.6, 130.2, 129.5, 128.7, 128.3, 127.0, 126.4, 125.4, 125.3, 123.2, 119.5, 78.9, 50.6, 46.3, 33.2, 18.3, 6.8, 3.9.

HRMS-ESI (m/z): $[M+Na]^+$ calculated for $[C_{27}H_{35}N_3O_8SSiNa]^+$: 612.1812, found 612.1783.

***N*-(3-(*tert*-Butyldiphenylsilylperoxy)butyl)-*N*-(naphthalene-1-ylmethyl)-2, 4-dinitrobenzenesulfonamide (23)**

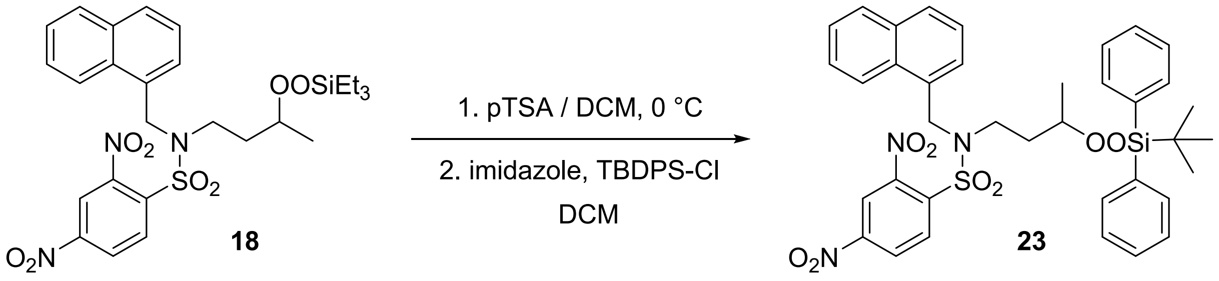

Following a modified general procedure 7, a solution of **18** (200 mg, 0.32 mmol, 1 eq) in DCM (2.0 mL, 0.2 M) was treated with p-toluenesulfonic acid (83 mg, 0.48 mmol, 1.5 eq). Upon completion, the residue was dissolved in DCM (2.0 mL, 0.2 M) and treated with imidazole (33 mg, 0.48 mmol, 1.5 eq) and TBDPS-Cl (0.13 mL, 0.48 mmol, 1 eq). The crude residue was purified by flash column chromatography on silica gel (eluent: 92:8 hexanes: EtOAc) to obtain the title compound **23** (150 mg, 0.21 mmol, 43% over 2 steps) as a colorless oil.

TLC: R_f = 0.40 (90:10 hexanes: EtOAc).

^{1}H NMR (500 MHz, $CDCl_3$): δ 8.25 (s, 1H, ArH), 7.98 – 7.92 (m, 1H, ArH), 7.81 (d, J = 8.7 Hz, 1H, ArH), 7.77 – 7.68 (m, 5H, ArH), 7.64 (d, J = 6.7 Hz, 2H, ArH), 7.59 (d, J = 8.7 Hz, 1H, ArH), 7.48 – 7.44 (m, 4H, ArH), 7.43 (d, J = 10.2 Hz, 2H, ArH), 7.41 – 7.35 (m, 4H, ArH), 7.34 – 7.27 (m, 2H, ArH), 4.87 (s, 2H, $ArCH_2$), 3.89 (q, J = 6.0 Hz, 1H, CH), 3.39 – 3.27 (m, 1H, CH_2), 3.24 – 3.14 (m, 1H, CH_2), 1.65 – 1.58 (m, 2H, CH_2), 1.05 (s, 9H, *t*Bu), 1.03 (d, J = 6.2 Hz, 3H, CH_3).

^{13}C NMR (126 MHz, $CDCl_3$): δ 149.1, 138.8, 135.8, 135.7, 134.9, 133.8, 132.8, 132.6, 131.6, 130.2, 129.8, 129.4, 128.7, 128.1, 127.8, 127.0, 126.3, 125.4, 123.3, 119.4, 79.2, 50.6, 45.9, 33.1, 27.4, 19.5, 18.4.

HRMS-ESI (m/z): $[M+Na]^+$ calculated for $[C_{37}H_{39}N_3O_8SSiNa]^+$: 736.2125, found 736.2102.

4) ***Protecting Group Screen***

Table 1, Entries 1-3: General Experimental for **2-Methyl-4-((naphthalen-1-ylmethyl)amino)butan-2-ol (26)**

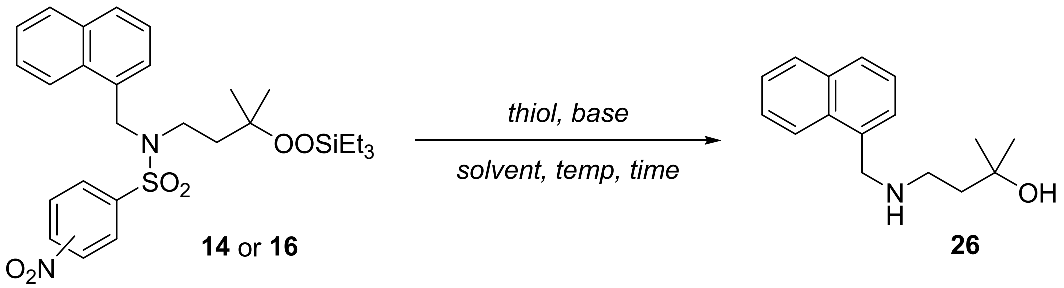

A solution of **14** or **16** (1 eq) in DMF or DCM (0.2 M) was treated with a thiol (1.2 eq) and a base (1.2 eq) with stirring at RT for 1-4 h. The crude mixture was monitored by TLC and ESIMS, and **26** was not isolated.

Entry	Substrate	Ar	Thiol	Base	Solvent	Temp	Time
**1**	14	2-NP	PhSH	K_2CO_3	DMF	RT	3 h
**2**	16	4-NP	PhSH	Cs_2CO_3	DMF	RT	3 h
**3**	16	4-NP	$HSCH_2COOH$	Et_3N	DCM	RT	1 h

2-Methyl-4-((naphthalen-1-ylmethyl)amino)butan-2-ol (26)

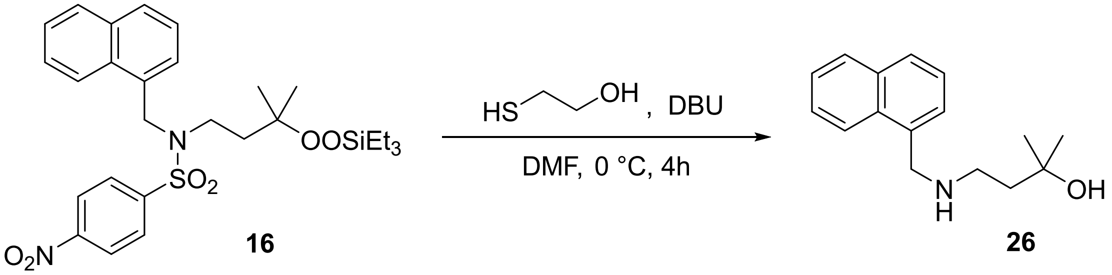

16 **26**

A solution of **16** (350 mg, 0.63 mmol, 1 eq) in DMF (3.0 mL, 0.2 M) was treated with 2-mercaptoethanol (0.73 mL, 0.75 mmol, 1.2 eq) and DBU (0.1 mL, 0.75 mmol, 1.2 eq) with stirring at 0 °C for 4 h. The mixture was quenched with H_2O and extracted with EtOAc (3 x 5 mL). The combined organic phases were washed with brine, dried over Na_2SO_4, concentrated *in vacuo*, and purified by flash column chromatography on silica gel (eluent: 99:1 EtOAc: Et_3N) to obtain the title compound **26** (133 mg, 0.55 mmol, 87%) as a colorless oil.

TLC: R_f = 0.1 (90:10 EtOAc: MeOH).

^{1}H NMR (500 MHz, $CDCl_3$): δ 8.07 (d, *J* = 8.4 Hz, 1H, ArH), 7.86 (d, *J* = 7.7 Hz, 1H, ArH), 7.78 (d, *J* = 8.3 Hz, 1H, ArH), 7.55 (t, *J* = 6.9 Hz, 1H, ArH), 7.49 (t, *J* = 6.9 Hz, 1H, ArH), 7.46 – 7.36 (m, 2H, ArH), 4.24 (s, 2H, $ArCH_2$), 3.02 (t, *J* = 5.9 Hz, 2H, CH_2), 1.65 (t, *J* = 5.9 Hz, 2H, CH_2), 1.21 (s, 6H, $(CH_3)_2$).

^{13}C NMR (126 MHz, $CDCl_3$): δ 135.1, 134.0, 131.8, 128.9, 128.2, 126.5, 126.4, 125.9, 125.4, 123.5, 71.0, 51.6, 46.2, 40.6, 29.7.

HRMS-ESI (m/z): $[M+H]^+$ calculated for $[C_{16}H_{22}NO]^+$: 244.1696, found 244.1693.

N-(Naphthalen-1-ylmethyl)-4-nitro-N-(3-oxobutyl)benzenesulfonamide (27)

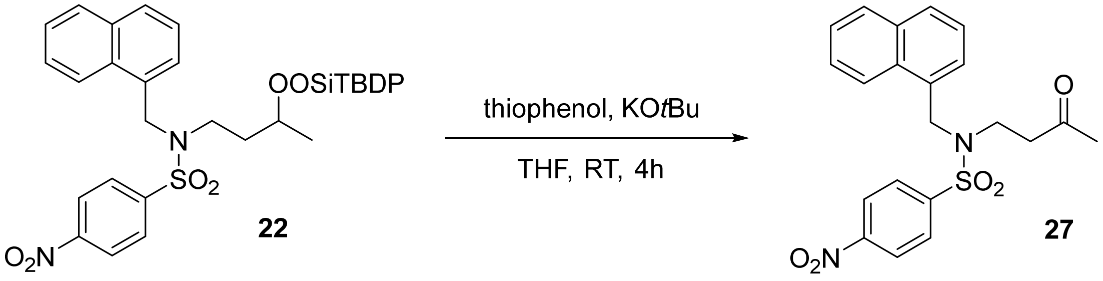

Following a literature protocol,[9] a solution of **22** (152 mg, 0.28 mmol, 1 eq) in THF (2.0 mL, 0.2 M) was treated with thiophenol (0.03 mL, 0.33 mmol, 1.2 eq) and potassium *tert*-butoxide (94 mg, 0.84 mmol, 3 eq) with stirring at RT for 4h. The crude mixture was quenched with H_2O and extracted with EtOAc (3 x 10 mL). The combined organic layers were washed with brine, dried over Na_2SO_4, and concentrated *in vacuo*. The crude residue was then purified by flash column chromatography on silica gel (eluent: 60:40 hexanes: EtOAc) to obtain the title compound **27** (108 mg, 0.26 mmol, 94%) as a white solid.

TLC: R_f = 0.12 (80:20 hexanes: EtOAc).

^{1}H NMR (500 MHz, $CDCl_3$): δ 8.34 (d, J = 8.8 Hz, 2H, ArH), 8.26 (d, J = 8.4 Hz, 1H, ArH), 8.00 (d, J = 8.8 Hz, 2H, ArH), 7.85 (dd, J = 16.3, 8.8 Hz, 2H, ArH), 7.63 – 7.51 (m, 2H, ArH), 7.37 (t, J = 7.6 Hz, 1H, ArH), 7.30 (d, J = 6.9 Hz, 1H, ArH), 4.76 (s, 2H, $ArCH_2$), 3.36 (t, J = 7.4 Hz, 2H, CH_2), 2.31 (t, J = 7.4 Hz, 2H, CH_2), 1.82 (s, 3H, CH_3).

^{13}C NMR (126 MHz, $CDCl_3$): δ 206.4, 150.2, 144.1, 134.0, 131.7, 130.2, 129.8, 128.9, 128.6, 128.2, 127.2, 126.5, 125.2, 124.5, 123.7, 52.5, 43.6, 43.5, 30.0.

HRMS-ESI (m/z): $[M+Na]^+$ calculated for $[C_{21}H_{20}N_2O_5SNa]^+$: 435.0991, found 435.1001.

***N*-(Naphthalen-1-ylmethyl)-4-nitro-*N*-(3-oxobutyl)benzenesulfonamide (27) and 5-Methyl-2-(naphthalen-1-ylmethyl)isoxazolidine (29)**

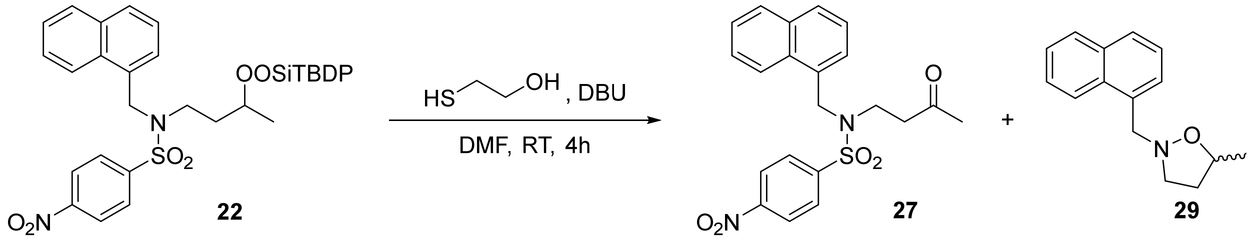

A solution of **22** (150 mg, 0.22 mmol, 1 eq) in DMF (1.0 mL, 0.2 M) was treated with 2-mercaptoethanol (0.18 mL, 0.22 mmol, 1 eq) and DBU (0.03 mL, 0.22 mmol, 1 eq) with stirring at RT for 4h. The crude mixture was quenched with H_2O and extracted with EtOAc (3 x 10 mL). The combined organic layers were washed with brine, dried over Na_2SO_4, and concentrated *in vacuo*. The crude products were purified by flash column chromatography on silica gel (**29:** eluent: 90:10 hexanes: EtOAc. **27:** eluent: 60:40 hexanes: EtOAc) to obtain the title compound **27** (83 mg, 0.2 mmol, 90%) as a white solid with spectra data consistent with the above sample, and **29** (2 mg, 0.01 mmol, 4%) as a colorless oil with spectra data consistent with the sample described below.

4-(2-Hydroxyethylthio)-*N*-(naphthalen-1-ylmethyl)-2-nitro-*N*-(3-oxobutyl)benzenesulfonamide (30), 4-((naphthalen-1-ylmethyl)amino)butan-2-one (28), and 5-Methyl-2-(naphthalen-1-ylmethyl)isoxazolidine (29)

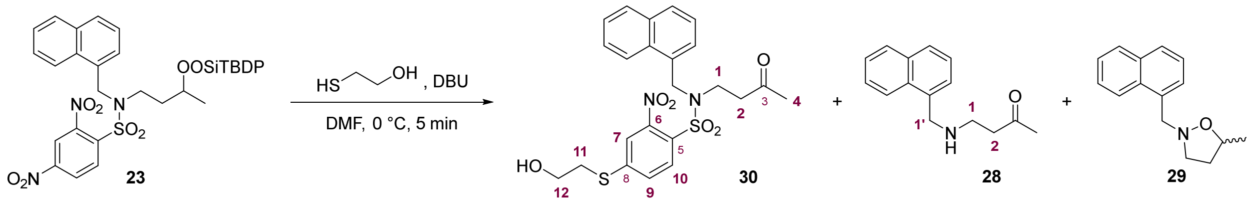

A solution of **23** (170 mg, 0.24 mmol, 1 eq) in DMF (1.0 mL, 0.2 M) at 0 °C was treated with 2-mercaptoethanol (0.18 mL, 0.24 mmol, 1 eq) and DBU (0.06 mL, 0.24 mmol, 1 eq) with stirring for 5 min. The crude products were then purified by flash column chromatography on silica gel (**30:** eluent: 60:40 hexanes: EtOAc. **28**: eluent: 5:95 hexanes: EtOAc. **29:** eluent: 92:8 hexanes: EtOAc) to obtain the title compounds **30** (59 mg, 0.12 mmol, 51%) as a yellow oil, **28** (9 mg, 0.04 mmol, 17%) as a colorless oil, and **29** (15 mg, 0.07 mmol, 28%) as a colorless oil.

<u>Data for 30:</u>

TLC: R_f = 0.23 (70:30 hexanes: EtOAc).

^{1}H NMR (500 MHz, C_6D_6): δ 8.34 (d, J = 8.4 Hz, 1H, ArH), 7.52 (d, J = 8.2 Hz, 1H, ArH), 7.46 (d, J = 8.3 Hz, 1H, ArH), 7.40 (d, J = 8.4 Hz, 1H, ArH), 7.31 – 7.24 (m, 1H, ArH), 7.20 – 7.11 (m, 3H, ArH), 7.05 – 6.99 (m, 1H, ArH^{10}), 6.86 (s, 1H, ArH^7), 6.55 (d, J = 8.4 Hz, 1H, ArH^9), 4.83 (s, 2H, $ArCH_2$), 3.52 (t, J = 7.4 Hz, 2H, $C^{12}H_2$), 3.08 (t, J = 6.0 Hz, 2H, C^1H_2), 2.24 (t, J = 6.1 Hz, 2H, $C^{11}H_2$), 2.01 (t, J = 7.4 Hz, 2H, C^2H_2), 1.28 (br, 1H, OH), 1.16 (s, 3H, C^4H_3).

^{13}C NMR (126 MHz, C_6D_6): δ 206.5, 146.1, 137.8, 136.5, 133.9, 131.6, 131.2, 129.4, 128.8, 128.6, 127.8, 127.0, 126.3, 125.3, 123.6, 121.5, 96.0, 60.7, 51.9, 49.0, 43.0, 35.2, 30.0.

HRMS-ESI (m/z): $[M+Na]^+$ calculated for $[C_{23}H_{24}N_2O_6S_2Na]^+$: 511.0973, found 511.0967.

Data for 28:

TLC: R_f = 0.42 (99:1 DCM: MeOH).

^{1}H NMR (500 MHz, CDCl$_3$): δ 8.11 (d, *J* = 8.4 Hz, 1H), 7.86 (d, *J* = 8.1 Hz, 1H, ArH), 7.76 (d, *J* = 8.0 Hz, 1H, ArH), 7.53 (dd, *J* = 8.9, 6.1 Hz, 1H, ArH), 7.48 (dd, *J* = 8.9, 8.0 Hz, 2H, ArH), 7.42 (d, *J* = 8.1 Hz, 1H, ArH), 4.23 (s, 2H, $C^{1'}H_2$), 2.98 (t, *J* = 6.3 Hz, 2H, C^1H_2), 2.69 (t, *J* = 6.3 Hz, 2H, C^2H_2), 2.14 (s, 3H, C^4H_3).

HRMS-ESI (m/z): $[M+H]^+$ calculated for $[C_{15}H_{18}NO]^+$: 228.1383, found 228.1372.

Data for 29:

TLC: R_f = 0.54 (95:5 hexanes: EtOAc + 1% Et_3N).

^{1}H NMR RT (500 MHz, C_6D_6): δ 8.42 (d, *J* = 8.4 Hz, 1H, ArH^{5n}), 7.66 (d, *J* = 8.1 Hz, 1H, ArH^8), 7.61 (d, *J* = 8.3 Hz, 1H, ArH^1), 7.46 (d, *J* = 6.5 Hz, 1H, ArH^6), 7.35 (t, *J* = 7.8 Hz, 1H, ArH^7), 7.29 – 7.24 (m, 2H, ArH^2 and ArH^{3n}), 4.65 (br, 2H, $C^{2'}H_2$), 4.10 (br, 1H, C^5H), 2.74 (br, 2H, C^3H_2), 1.93 (dt, *J* = 16.6, 7.7 Hz, 1H, C^4H), 1.42 (dt, *J* = 13.6, 6.3 Hz, 1H, C^4H), 1.11 (d, *J* = 6.0 Hz, 3H, $C^{5'}H_3$).

^{1}H NMR 70 °C (500 MHz, C_6D_6): δ 8.38 (d, *J* = 8.5 Hz, 1H, ArH^{5n}), 7.66 (d, *J* = 8.1 Hz, 1H, ArH^8), 7.60 (d, *J* = 8.2 Hz, 1H, ArH^1), 7.47 (d, *J* = 6.9 Hz, 1H, ArH^6), 7.34 (t, *J* = 7.7 Hz, 1H, ArH^7), 7.29 – 7.24 (m, 2H, ArH^2 and ArH^{3n}), 4.35 (br, 1H, $C^{2'}H$), 4.28 (br, 1H, $C^{2'}H$), 4.10 (h, *J* = 6.2 Hz, 1H, C^5H), 2.69 (br, 1H, C^3H), 2.60 (br, 1H, C^3H), 1.98 (dq, *J* = 16.2, 7.6 Hz, 1H, C^4H), 1.42 (td, *J* = 13.6, 6.3 Hz, 1H, C^4H), 1.12 (d, *J* = 6.0 Hz, 3H, $C^{5'}H_3$).

^{13}C NMR RT (126 MHz, C_6D_6): δ 134.4 (C^{10}), 134.1 C^9), 133.0 (C^{4n}), 128.7 (C^8), 128.4 (C^1), 127.6 (C^6), 126.1 (C^7), 125.9 (C^2), 125.5 (C^{3n}), 125.4 (C^{5n}), 73.0 (br, C^5), 60.8 (br, $C^{2'}$), 54.7 (br, C^3), 36.0 (C^4), 20.6 (br, $C^{5'}$).

^{13}C NMR 70 °C (126 MHz, C_6D_6): δ 134.1 (C^a), 133.9 (C^f), 132.7 (C^j), 128.3 (C^e), 128.0 (C^g), 127.1 (C^c), 125.7 (C^d), 125.4 (C^h), 125.1 (C^i), 124.9 (C^b), 72.7 (C^5), 60.5 ($C^{2'}$), 54.4 (C^3), 35.9 (C^4), 20.3 ($C^{5'}$).

HRMS-ESI (m/z): $[M+H]^+$ calculated for $[C_{16}H_{17}NO]^+$: 228.1383, found 228.1380.

***N*-(3-(*tert*-Butyldiphenylsilylperoxy)butyl)-4-(2-hydroxyethylthio)-*N*-(naphthalen-1-ylmethyl)-2-nitrobenzenesulfonamide (31) and 5-Methyl-2-(naphthalen-1-ylmethyl)isoxazolidine (29)**

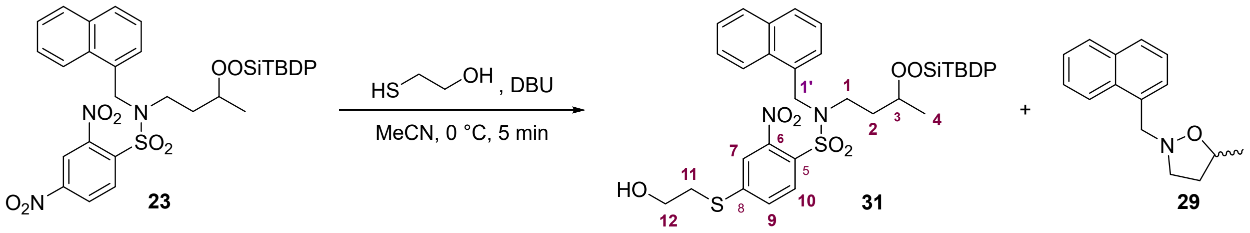

A solution of **23** (83 mg, 0.11 mmol, 1 eq) in MeCN (0.5 mL, 0.2 M) at 0 °C was treated with 2-mercaptoethanol (0.1 mL, 0.11 mmol, 1 eq) and DBU (0.02 mL, 0.11 mmol, 1 eq) with stirring for 5 min. The crude product was then purified by flash column chromatography on silica gel (**31:** eluent: 70:30 hexanes: EtOAc) to obtain the title compound **31 (**41 mg, 0.05 mmol, 48%) as a colorless oil, and **29** (6 mg, 0.02 mmol, 22%) as a colorless oil with spectral data consistent with the sample described above.

Data for 31:

TLC: R_f = 0.42 (70:30 hexanes: EtOAc).

^{1}H NMR (500 MHz, CDCl$_3$): δ 8.07 (dd, *J* = 6.1, 3.3 Hz, 1H, ArH), 7.82 (dd, *J* = 6.3, 3.3 Hz, 1H, ArH), 7.74 (d, *J* = 8.4 Hz, 1H, ArH), 7.64 (d, *J* = 6.9 Hz, 2H, ArH), 7.59 (d, *J* = 7.7 Hz, 2H, ArH), 7.56 (d, *J* = 8.3 Hz, 1H, ArH), 7.48 (dd, *J* = 6.3, 3.1 Hz, 2H, ArH), 7.43 – 7.32 (m, 6H, ArH), 7.30 (d, *J* = 6.7 Hz, 1H, ArH), 7.28 (s, 1H, ArH^7), 7.23 (d, *J* = 7.5 Hz, 1H, ArH), 7.13 (d, *J* = 6.7 Hz, 1H, ArH), 4.88 (d, *J* = 15.2 Hz, 1H, C$^{1'}$H), 4.84 (d, *J* = 15.0 Hz, 1H, C$^{1'}$H), 3.88 (t, *J* = 6.0 Hz, 2H, C^{1}H$_2$), 3.77 (q, *J* = 6.2 Hz, 1H, C^{3}H), 3.19 (t, *J* = 6.0 Hz, 2H, C^{12}H$_2$), 1.35 – 1.27 (m, 2H, C^{2}H$_2$), 1.47 (dd, *J* = 10.5, 5.7 Hz, 2H, C^{11}H$_2$), 1.02 (s, 9H, *t*Bu), 0.93 (d, *J* = 6.3 Hz, 3H, C^{4}H$_3$).

HRMS-ESI (m/z): [M+Na]$^+$ calculated for [$C_{39}H_{44}N_2O_7S_2SiNa$]$^+$: 767.2257, found 767.2205.

5) Synthesis and Characterization of Substrates

***N*-(Naphthalen-1-ylmethyl)-2-nitro-4-(trifluoromethyl)benzenesulfonamide (S3)**

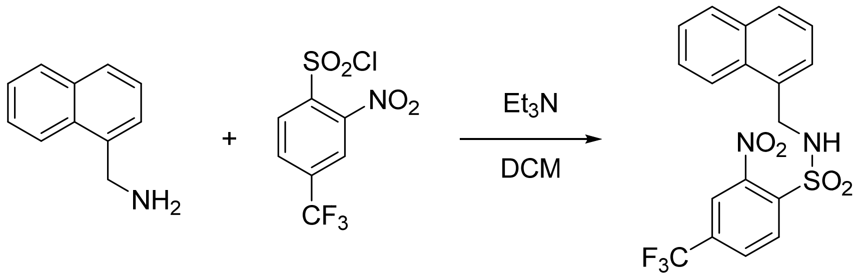

S3

Following general procedure 1A, 1-naphthalylmethylamine (0.52 mL, 3.45 mmol, 1 eq) in dry DCM (0.2 M) with triethylamine (0.3 mL, 4.14 mmol, 1.2 eq) was treated with 2-nitro-4-(trifluoromethyl)benzenesulfonyl chloride (1.0 g, 4.14 mmol, 1.2 eq). The crude residue was purified by flash column chromatography on silica gel (eluent: 75:25 hexanes: EtOAc) to obtain the title compound **S3** (1.4 g, 3.3 mmol, 96%) as a yellow solid.

TLC: R*f* = 0.31 (80:20 hexanes: EtOAc).

^{1}H NMR (500 MHz, CDCl$_3$): δ 7.89 (d, *J* = 8.0 Hz, 1H, ArH), 7.77 (s, 2H, ArH), 7.71 (d, *J* = 7.8 Hz, 1H, ArH), 7.68 (d, *J* = 8.3 Hz, 1H, ArH), 7.57 (d, *J* = 8.3 Hz, 1H, ArH), 7.49 – 7.39 (m, 3H, ArH), 7.32 (t, *J* = 7.7 Hz, 1H, ArH), 6.09 (br, 1H, NH), 4.82 (s, 2H, ArCH$_2$).

^{13}C {^{1}H, ^{19}F} NMR (126 MHz, CDCl$_3$): δ 147.0, 137.3, 135.0, 133.8, 131.5, 131.0, 130.9, 129.5, 129.0, 128.9, 128.3, 127.0, 126.3, 125.1, 122.9, 122.2, 120.9, 46.5.

HRMS-ESI (m/z): [M+Na]$^+$ calculated for [$C_{18}H_{13}F_3N_2O_4SNa$]$^+$: 433.0446, found 433.0430.

N-(But-3-en-1-yl)-N-(naphthalen-1-ylmethyl)-2-nitro-4-(trifluoromethyl)-benzenesulfonamide (13)

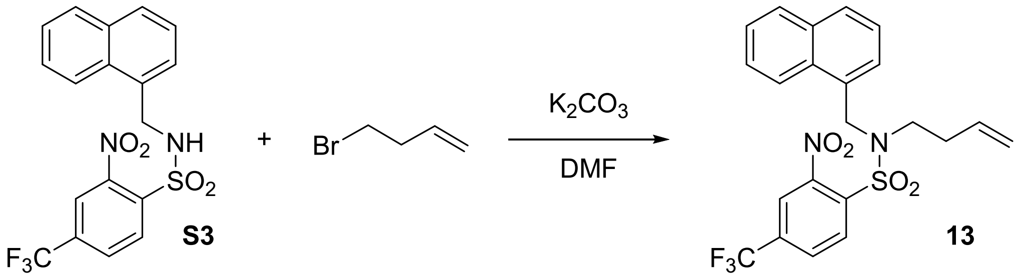

Following general procedure 2A, a stirred solution of **S3** (840 mg, 2.03 mmol, 1 eq) in DMF (10 mL, 0.2 M) was treated with K_2CO_3 (560 mg 4.1 mmol, 2 eq) followed by 4-bromobut-1-ene (0.25 mL, 2.46 mmol, 1.2 eq). The crude residue was purified by flash column chromatography on silica gel (eluent: 90:10 hexanes: EtOAc) to obtain the title compound **13** (895 mg, 1.93 mmol, 95%) as an orange solid.

TLC: R$_f$ = 0.44 (90:10 hexanes: EtOAc).

^{1}H NMR (500 MHz, $CDCl_3$): δ 8.01 (d, J = 8.2 Hz, 1H, ArH), 7.83 – 7.73 (m, 4H, ArH), 7.59 (d, J = 8.4 Hz, 1H, ArH), 7.52 – 7.42 (m, 3H, ArH), 7.41 – 7.36 (m, 1H, ArH), 5.61 (ddt, J = 17.2, 10.5, 6.9 Hz, 1H, =CH), 4.99 (s, 2H, ArCH$_2$), 4.97 (d, J = 8.8 Hz, 2H, CH=CH$_2$), 3.54 – 3.48 (m, 2H, CH$_2$), 2.22 (q, J = 7.2 Hz, 2H, CH$_2$).

^{13}C {^{1}H, ^{19}F} NMR (126 MHz, $CDCl_3$): δ 137.1, 134.0, 133.7, 132.4, 131.9, 131.8, 131.6, 130.3, 129.4, 128.8, 128.0, 127.9, 126.9, 126.2, 125.2, 123.1, 121.4, 121.2, 117.7, 50.6, 48.9, 33.1.

HRMS-ESI (m/z): [M+Na]$^+$ calculated for $[C_{22}H_{19}F_3N_2O_4SNa]^+$: 487.0915, found 487.0900.

***N*-(Naphthalen-1-ylmethyl)-2-nitro-*N*-(3-(triethylsilylperoxy)butyl)-4-(trifluoromethyl)-benzenesulfonamide (19)**

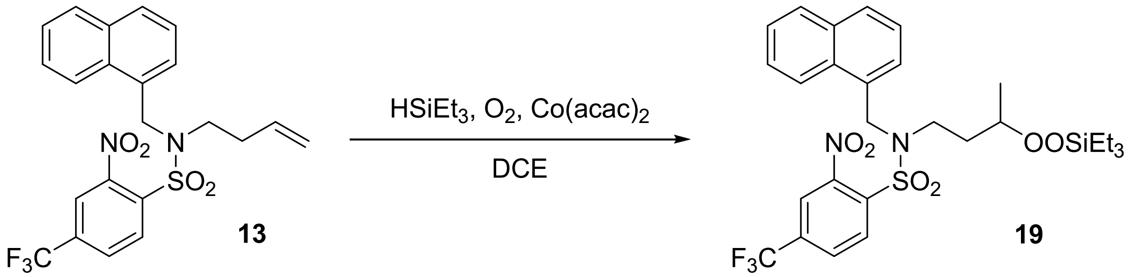

Following general procedure 6A, a stirred solution of **13** (640 mg, 1.38 mmol, 1 eq) in DCE (7.0 mL, 0.2 M) was treated with triethylsilane (0.53 mL, 3.44 mmol, 2.5 eq), and Co(acac)$_2$ (35 mg, 0.14 mmol, 10 mol%) under an atmosphere of oxygen. The crude residue was then purified by flash column chromatography on silica gel (eluent: 92:8 hexanes: EtOAc) to obtain the title compound **19** (560 mg, 0.95 mmol, 69%) as a colorless oil.

TLC: R*f* = 0.41 (90:10 hexanes: EtOAc).

^{1}H NMR (500 MHz, CDCl$_3$): δ 8.05 (d, *J* = 7.4, 2.1 Hz, 1H, ArH), 7.86 – 7.75 (m, 4H, ArH), 7.60 (d, *J* = 8.1, 1.6 Hz, 1H, ArH), 7.49 – 7.46 (m, 3H, ArH), 7.38 (t, *J* = 7.6 Hz, 1H, ArH), 4.72 (d, *J* = 14.9 Hz, 1H, ArCH), 4.69 (d, *J* = 14.9 Hz, 1H, ArCH), 3.92 (q, *J* = 6.0 Hz, 1H, CH), 3.62 – 3.44 (m, 2H, CH$_2$), 1.79 (ddt, *J* = 16.9, 9.5, 5.5 Hz, 1H, CH$_2$), 1.67 (ddt, *J* = 13.5, 10.5, 5.1 Hz, 1H, CH$_2$), 1.07 (d, *J* = 6.2 Hz, 3H, CH$_3$), 0.93 (t, *J* = 8.0 Hz, 9H, SiCH$_2$C*H*$_3$), 0.61 (q, *J* = 8.0 Hz, 6H, SiC*H*$_2$CH$_3$).

^{13}C NMR (126 MHz, CDCl$_3$): δ 147.7, 137.1, 135.1 (q, *J* = 34.7 Hz), 133.8, 131.9, 131.7, 130.4, 129.4, 128.8, 128.1 (q, *J* = 3.6 Hz), 128.0, 126.9, 126.2, 125.2, 123.3, 122.1 (q, *J* = 273.7 Hz), 121.5 (q, *J* = 3.8 Hz), 79.0, 50.5, 45.9, 33.2, 18.3, 6.8, 3.8.

HRMS-ESI (m/z): [M+Na]$^+$ calculated for [$C_{28}H_{35}F_3N_2O_6SSiNa$]$^+$: 612.7362, found 612.1790.

***N*-(3-(*tert*-Butyldiphenylsilylperoxy)butyl)-*N*-(naphthalen-1-ylmethyl)-2-nitro-4-(trifluoromethyl)benzenesulfonamide (24)**

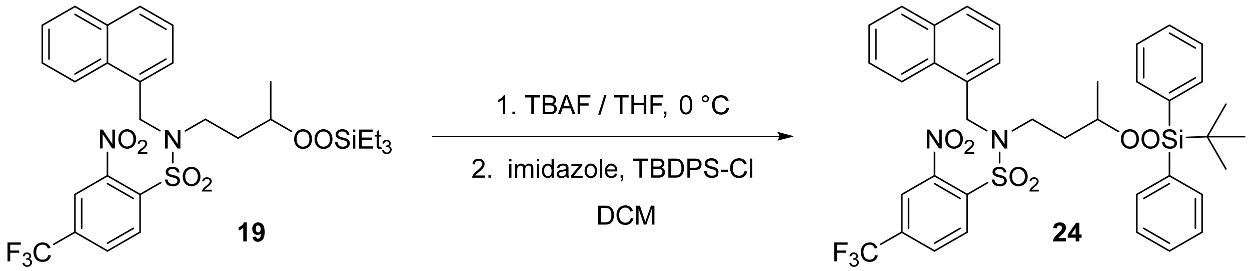

Following general procedure 7, TBAF (1.4 mL, 1.0 M in THF, 1.2 eq) was added drop-wise to a solution of **19** (690 mg, 1.13 mmol, 1 eq) in THF (6.0 mL, 0.2 M). Upon completion, the residue was dissolved in DCM (6.0 mL, 0.2 M) and treated with imidazole (115 mg, 1.7 mmol, 1.5 eq) and TBDPS-Cl (0.3 mL, 1.13 mmol, 1 eq). The crude product was purified by flash column chromatography on silica gel (eluent: 95:5 hexanes: EtOAc) to obtain the title compound **24** (707 mg, 0.96 mmol, 85% over 2 steps) as a colorless oil.

TLC: R_f = 0.33 (90:10 hexanes: acetone).

^{1}H NMR (500 MHz, Toluene-*D*$_8$): δ 8.02 (d, *J* = 8.4 Hz, 1H, ArH), 7.75 (d, *J* = 3.4 Hz, 2H, ArH), 7.68 (d, *J* = 5.5 Hz, 3H, ArH), 7.49 (d, *J* = 8.1 Hz, 1H, ArH), 7.43 (d, *J* = 8.2 Hz, 1H, ArH), 7.29 (d, *J* = 8.2 Hz, 1H, ArH), 7.21 – 7.18 (m, 6H, ArH), 7.17 – 7.12 (m, 4H, ArH), 7.01 (d, *J* = 7.9 Hz, 1H, ArH), 6.67 (d, *J* = 8.4 Hz, 1H, ArH), 4.71 (d, *J* = 4.8 Hz, 2H, $ArCH_2$), 3.75 – 3.67 (m, 1H, CH), 3.20 (ddd, *J* = 16.0, 11.1, 5.3 Hz, 1H, CH_2), 3.07 (ddd, *J* = 15.4, 10.9, 5.2 Hz, 1H, CH_2), 1.44 (ddt, *J* = 30.7, 10.3, 5.0 Hz, 2H, CH_2), 1.10 (s, 9H, *t*Bu), 0.83 (d, *J* = 6.1 Hz, 3H, CH_3).

^{13}C NMR (126 MHz, Toluene-*D*$_8$): δ 147.4, 137.0, 136.4, 135.6, 135.4, 134.7, 133.8 (q, *J* = 34.7 Hz), 132.6, 132.5, 131.6, 130.4, 129.7, 128.8, 127.7, 127.0 (q, *J* = 3.3 Hz), 126.4, 125.7,

124.7, 121.9 (q, J = 273.4 Hz), 120.4 (q, J = 3.6 Hz), 79.1, 50.2, 45.1, 32.8, 26.9, 26.2, 19.0, 17.7.

HRMS-ESI (m/z): $[M+Na]^+$ calculated for $[C_{38}H_{39}F_3N_2O_6SSiNa]^+$: 759.2148, found 759.2131.

***N*-(4-Fluorobenzyl)-2-nitro-4-(trifluoromethyl)benzenesulfonamide (S4)**

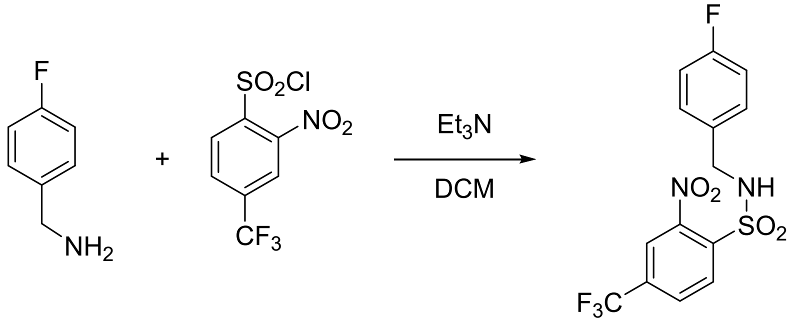

S4

Following general procedure 1A, (4-fluorophenyl)methylamine (0.3 mL, 2.5 mmol, 1 eq) in dry DCM (12 mL, 0.2 M) with triethylamine (0.3 mL, 3 mmol, 1.2 eq) was treated with 2-nitro-4-(trifluoromethyl)benzenesulfonyl chloride (883 mg, 3 mmol, 1.2 eq). The crude residue was purified by flash column chromatography on silica gel (eluent: 83:17 hexanes: EtOAc) to obtain the title compound **S4** (900 mg, 2.4 mmol, 95%) as a colorless oil.

TLC: R_f = 0.39 (80:20 hexanes: EtOAc).

^{1}H NMR (500 MHz, CDCl$_3$): δ 8.10 (d, J = 8.2 Hz, 1H, ArH), 8.06 (s, 1H, ArH), 7.87 (d, J = 8.2 Hz, 1H, ArH), 7.23 – 7.16 (m, 2H, ArH), 6.92 (t, J = 8.5 Hz, 2H, ArH), 5.77 (br, 1H, NH), 4.33 (d, J = 5.6 Hz, 2H, ArCH$_2$).

^{13}C {^{1}H, ^{19}F} NMR (126 MHz, CDCl$_3$): δ 163.7, 137.7, 134.1, 132.1, 131.4, 129.9, 129.8, 129.6, 122.7, 115.9, 115.8, 47.4.

HRMS-ESI (m/z): $[M+Na]^+$ calculated for $[C_{14}H_{10}F_4N_2O_4SNa]^+$: 401.0195, found 401.0179.

***N*-(But-3-en-1-yl)-*N*-(4-fluorobenzyl)-2-nitro-4-(trifluoromethyl)benzenesulfonamide (4a)**

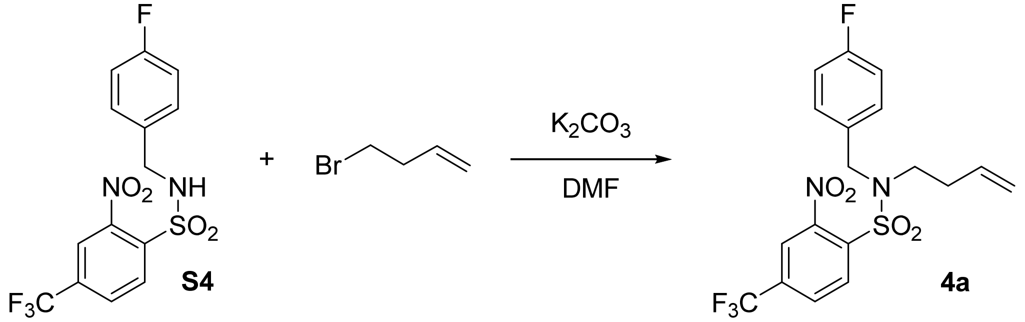

Following general procedure 2A, a stirred solution of **S4** (325 mg, 0.86 mmol, 1.0 eq) in DMF (4.0 ml, 0.2 M) was treated with K_2CO_3 (178 mg, 1.3 mmol, 1.5 eq), followed by 4-bromobut-1-ene (0.13 mL, 1.3 mmol, 1.5 eq). The crude residue was purified by flash column chromatography on silica gel (eluent: 93:7 hexanes: EtOAc) to obtain the title compound **4a** (352 mg, 0.82 mmol, 95%) as a yellow solid.

TLC: R_f = 0.61 (80:20 hexanes: EtOAc).

^{1}H NMR (500 MHz, $CDCl_3$): δ 8.12 (d, *J* = 8.2 Hz, 1H, ArH), 7.91 – 7.84 (m, 2H, ArH), 7.25 (d, *J* = 9.1, Hz, 2H, ArH), 7.00 (d, *J* = 8.6 Hz, 2H, ArH), 5.53 (ddt, *J* = 17.1, 10.2, 6.9 Hz, 1H, C*H*=CH_2), 4.95 (s, 1H, =C*H*), 4.91 (d, *J* = 9.3 Hz, 1H, =C*H*), 4.51 (s, 2H, ArCH_2), 3.34 – 3.27 (m, 2H, CH_2), 2.15 (q, *J* = 7.2 Hz, 2H, CH_2).

^{13}C NMR (126 MHz, $CDCl_3$): δ 162.7 (d, *J* = 247.2 Hz), 148.0, 137.5, 135.7 (q, *J* = 34.7 Hz), 133.9, 132.2, 131.1 (d, *J* = 3.3 Hz), 130.2 (d, *J* = 8.5 Hz), 128.6 (q, *J* = 3.6 Hz), 122.1 (q, *J* = 273.7 Hz), 121.8 (q, *J* = 3.6 Hz), 117.8, 115.9 (d, *J* = 21.7 Hz), 51.0, 47.2, 32.4.

^{19}F NMR (470 MHz, $CDCl_3$): δ -63.14 (s, 3F), -113.57 (ddd, *J* = 8.7, 4.4 Hz, 1F).

HRMS-ESI (m/z): $[M+Na]^+$ calculated for $[C_{18}H_{16}F_4N_2O_4SNa]^+$: 455.0665, found 455.0638.

***N*-(4-Fluorobenzyl)-2-nitro-*N*-(3-(triethylsilylperoxy)butyl)-4-(trifluoromethyl)-benzenesulfonamide (4b)**

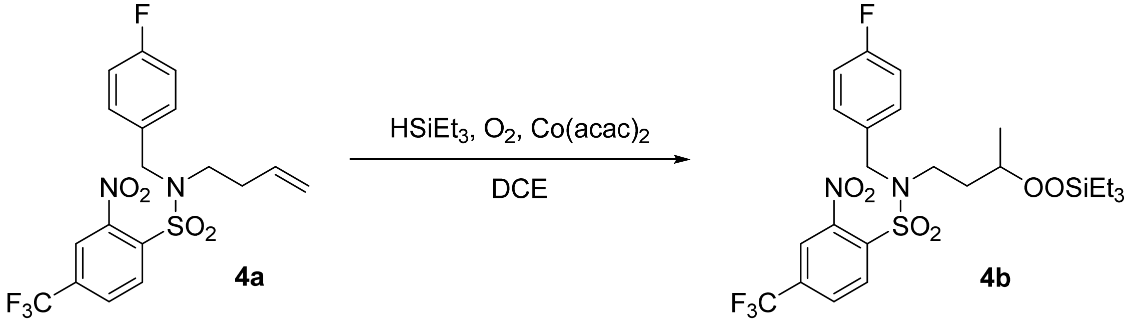

Following general procedure 6A, **4a** (377 mg, 0.87 mmol, 1 eq) in DCE (5.0 mL, 0.2 M) was treated with triethylsilane (0.4 mL, 2.2 mmol, 2.5 eq), and $Co(acac)_2$ (22 mg, 0.1 mmol, 10 mol%) under an atmosphere of oxygen. The crude product was then purified by flash column chromatography on silica gel (eluent: 95:5 hexanes: EtOAc) to obtain the title compound **4b** (309 mg, 0.53 mmol, 61%) as a yellow oil.

TLC: R_f = 0.37 (90:10 hexanes: EtOAc).

^{1}H NMR (500 MHz, $CDCl_3$): δ 8.12 (d, *J* = 8.3 Hz, 1H, ArH), 7.91 – 7.83 (m, 2H, ArH), 7.26 (d, *J* = 8.9, Hz, 2H, ArH), 6.99 (d, *J* = 8.3 Hz, 2H, ArH), 4.50 (d, *J* = 3.9 Hz, 2H, $ArCH_2$), 3.88 (h, *J* = 6.7 Hz, 1H, CH), 3.43 – 3.26 (m, 2H, CH_2), 1.76 – 1.57 (m, 2H, CH_2), 1.07 (d, *J* = 6.2 Hz, 3H, CH_3), 0.92 (t, *J* = 8.0 Hz, 9H, $SiCH_2C\mathit{H}_3$), 0.60 (q, *J* = 7.9 Hz, 6H, $SiC\mathit{H}_2CH_3$).

^{13}C NMR (126 MHz, $CDCl_3$): δ 162.8 (d, *J* = 247.2 Hz), 148.1, 137.5, 135.6 (q, *J* = 34.7 Hz), 132.2, 131.2 (d, *J* =3.29 Hz), 130.3 (d, *J* = 8.1 Hz), 128.6 (q, *J* = 3.6 Hz), 121.8 (q, *J* = 3.9 Hz), 121.3 (q, *J* = 237.5 Hz), 115.9 (d, *J* = 21.7 Hz), 78.8, 51.0, 44.2, 32.6, 18.3, 6.8, 3.9.

HRMS-ESI (m/z): $[M+Na]^+$ calculated for $[C_{24}H_{32}F_4N_2O_6SSiNa]^+$: 603.1584, found 603.1569.

***N*-(3-(*tert*-Butyldiphenylsilylperoxy)butyl)-*N*-(4-fluorobenzyl-2-nitro-4-(trifluoromethyl)-benzenesulfonamide (34)**

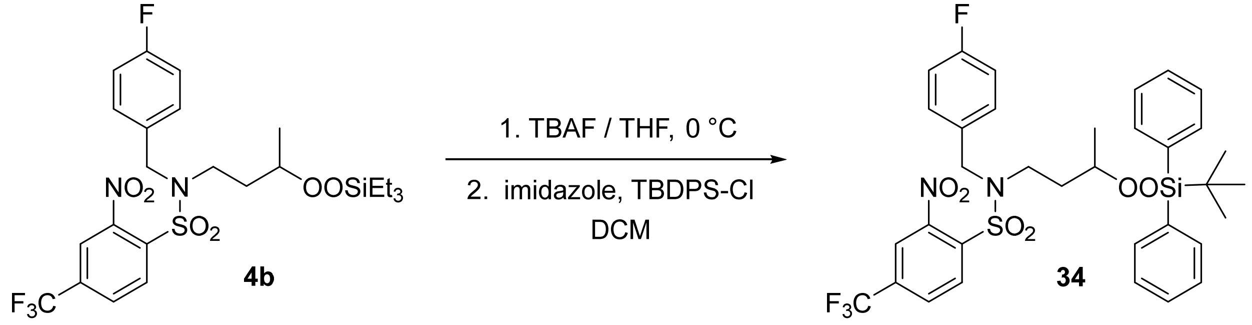

Following general procedure 7, TBAF (0.54 ml, 1.2 eq) was added to a solution of **4b** (260 mg, 0.45 mmol, 1 eq) in THF (3.0 mL, 0.2M). The residue was then dissolved in DCM (3.0 mL, 0.2 M) and treated with imidazole (46 mg, 0.67 mmol, 1.5 eq) and TBDPS-Cl (0.12 mL, 0.45 mmol, 1 eq). The crude residue was purified by flash column chromatography on silica gel (eluent: 57:43 hexanes: DCM) to obtain the title compound **34** (230 mg, 0.33 mmol, 73% over 2 steps) as a colorless oil.

TLC: R_f = 0.39 (50:50 hexanes: DCM).

^{1}H NMR (500 MHz, C_6D_6): δ 7.80 – 7.76 (m, 2H, ArH), 7.74 – 7.70 (m, 2H, ArH), 7.47 (d, *J* = 8.2 Hz, 1H, ArH), 7.24 – 7.17 (m, 6H, ArH), 7.11 (s, 1H, ArH), 6.91 (dd, *J* = 8.1, 5.8 Hz, 2H, ArH), 6.80 (d, *J* = 8.3 Hz, 1H, ArH), 6.63 (t, *J* = 8.7 Hz, 2H, ArH), 4.23 (d, *J* = 15.3 Hz, 1H, ArCH), 4.16 (d, *J* = 15.3 Hz, 1H, ArCH), 3.73 (h, *J* = 6.2 Hz, 1H, CH), 3.11 (ddd, *J* = 16.0, 10.8, 5.6 Hz, 1H, CH), 3.00 (ddd, *J* = 15.6, 10.5, 5.7 Hz, 1H, CH), 1.45 (dtq, *J* = 18.7, 9.1, 5.0 Hz, 2H, CH_2), 1.13 (s, 9H, *t*Bu), 0.85 (d, *J* = 6.1 Hz, CH_3).

^{13}C NMR (126 MHz, C_6D_6): δ 163.6 (d, *J* = 246.6 Hz), 147.8, 135.7 (d, *J* = 7.2 Hz), 135.0, 134.4 (q, *J* = 34.4 Hz), 132.7 (d, *J* = 13.0 Hz), 131.9, 131.4 (q, *J* = 3.0 Hz), 130.2, 130.1 (d, *J* = 8.5 Hz), 129.5, 122.1 (q, *J* = 273.4 Hz), 120.9 (q, *J* = 3.3 Hz), 115.6, 79.1, 50.8, 44.0, 32.5, 27.1, 26.4, 19.2, 17.9.

HRMS-ESI (m/z): $[M+Na]^+$ calculated for $[C_{34}H_{36}F_4N_2O_6SSiNa]^+$: 727.1897, found 727.1887.

***N*-(4-Methoxyphenethyl)-2-nitro-4-(trifluoromethyl)benzenesulfonamide (S5)**

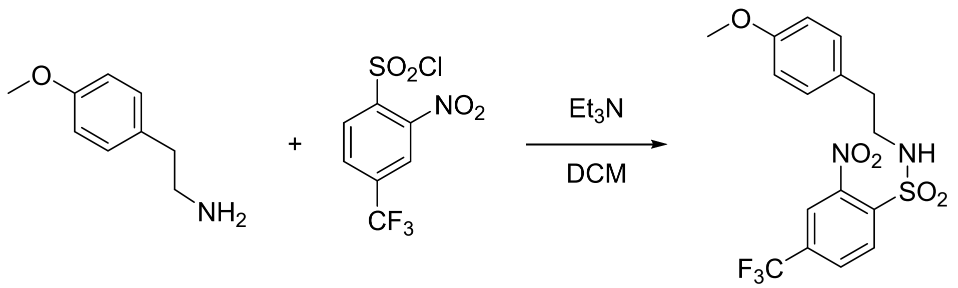

S5

Following general procedure 1A, 2-(4-methoxyphenyl)ethylamine (0.23 mL, 1.57 mmol, 1 eq) in dry DCM (8.0 mL, 0.2 M) with triethylamine (0.25 mL, 1.73 mmol, 1.2 eq) was treated with 2-nitro-4-(trifluoromethyl)benzenesulfonyl chloride (500 mg, 1.73 mmol, 1.2 eq). The crude product was purified by flash column chromatography on silica gel (eluent: 80:20 hexanes: EtOAc) to obtain the title compound **S5** (590 mg, 1.5 mmol, 93%) as a yellow solid.

TLC: R_f = 0.33 (80:20 hexanes: EtOAc).

^{1}H NMR (500 MHz, CDCl$_3$): δ 8.15 (d, *J* = 8.1 Hz, 1H, ArH), 8.04 (s, 1H, ArH), 7.92 (d, *J* = 8.1 Hz, 1H, ArH), 6.98 (d, *J* = 8.3 Hz, 2H, ArH), 6.71 (d, *J* = 8.3 Hz, 2H, ArH), 5.35 (t, *J* = 5.8 Hz, 1H, NH), 3.75 (s, 3H, OMe CH_3), 3.42 (q, *J* = 6.5 Hz, 2H, CH_2), 2.78 (t, *J* = 6.8 Hz, 2H, CH_2).

^{13}C NMR (126 MHz, CDCl$_3$): δ 158.7, 147.9, 137.6, 135.4 (q, *J* = 34.7 Hz), 131.9, 129.8, 129.7 (q, *J* = 3.3 Hz), 129.2, 122.8 (q, *J* = 3.6 Hz), 122.15 (q, *J* = 273.4 Hz), 114.2, 55.3, 45.6, 35.2.

HRMS-ESI (m/z): $[M+Na]^+$ calculated for $[C_{16}H_{15}F_3N_2O_5SNa]^+$: 427.0551, found 427.0527.

N-(But-3-en-1-yl)-N-(4-methoxyphenethyl)-2-nitro-4-(trifluoromethyl)benzenesulfonamide (5a)

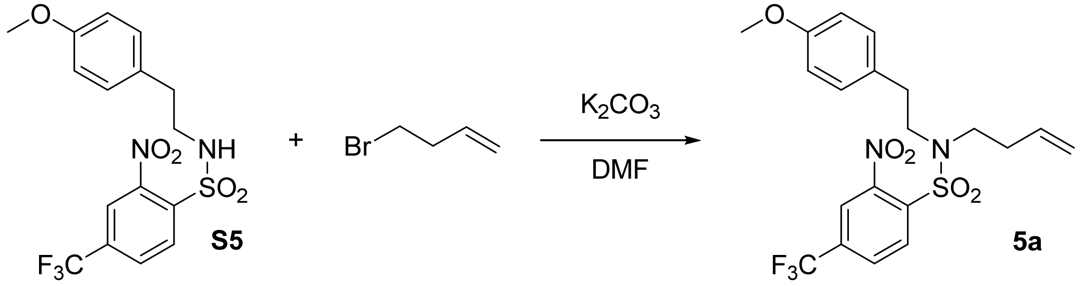

Following general procedure 2A, a stirred solution of **S5** (700 mg, 1.73 mmol, 1.0 eq) in DMF (9.0 mL, 0.2 M) was treated with K_2CO_3 (358 mg 2.59 mmol, 1.5 eq) followed by 4-bromobut-1-ene (0.26 mL, 2.59 mmol, 1.5 eq). The crude residue was purified by flash column chromatography on silica gel (eluent: 93:7 hexanes: EtOAc) to obtain the title compound **5a** (720 mg, 1.6 mmol, 91%) as a yellow oil.

TLC: R*f* = 0.61 (80:20 hexanes: EtOAc).

^{1}H NMR (500 MHz, CDCl$_3$): δ 8.03 (d, *J* = 8.6 Hz, 1H, ArH), 7.83 – 7.81 (m, 2H, ArH), 7.05 (d, *J* = 8.3 Hz, 2H, ArH), 6.75 (d, *J* = 8.3 Hz, 2H, ArH), 5.72 (ddt, *J* = 17.0, 10.2, 6.8 Hz, 1H, C*H*=CH$_2$), 5.10 (d, *J* = 17.1 Hz, 1H, =C*H*), 5.05 (d, *J* = 10.2 Hz, 1H, =C*H*), 3.76 (s, 3H, OMe CH$_3$), 3.54 (t, *J* = 7.7 Hz, 2H, ArCH$_2$), 3.46 (t, *J* = 8.5, 6.6 Hz, 2H, CH$_2$), 2.81 (t, *J* = 7.7 Hz, 2H, CH$_2$), 2.36 (q, *J* = 7.2 Hz, 2H, CH$_2$).

^{13}C NMR (126 MHz, CDCl$_3$): δ 158.6, 147.9, 137.4, 135.2 (q, *J* = 34.9 Hz), 134.1, 131.9, 129.84, 129.76, 128.5 (q, *J* = 3.3 Hz), 122.2 (q, *J* = 273.4 Hz), 121.6 (q, *J* = 3.6 Hz), 117.8, 114.1, 55.3, 49.1, 47.0, 33.9, 32.7.

HRMS-ESI (m/z): $[M+Na]^+$ calculated for $[C_{20}H_{21}F_3N_2O_5SNa]^+$: 481.1021, found 481.0996.

***N*-(4-Methoxyphenethyl)-2-nitro-*N*-(3-(triethylsilylperoxy)butyl)-4-(trifluoromethyl)-benzenesulfonamide (5b)**

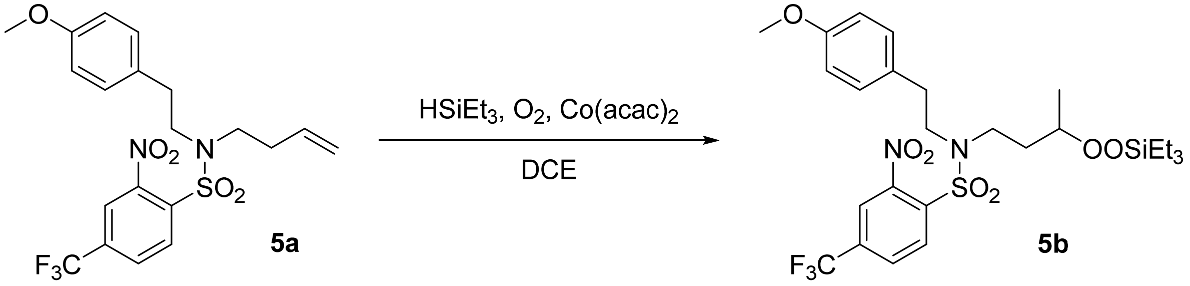

Following general procedure 6A, **5a** (900 mg, 1.96 mmol, 1 eq) in DCE (15 mL, 0.2 M) was treated with triethylsilane (0.74 mL, 4.9 mmol, 2.5 eq), and $Co(acac)_2$ (51 mg, 0.2 mmol, 10 mol%) under an atmosphere of oxygen. The crude product was then purified by flash column chromatography on silica gel (eluent: 93:7 hexanes: EtOAc) to obtain the title compound **5b** (703 mg, 1.16 mmol, 59%) as a colorless oil.

TLC: R_f = 0.33 (90:10 hexanes: EtOAc).

^{1}H NMR (500 MHz, $CDCl_3$): δ 8.02 (d, *J* = 8.1 Hz, 1H, ArH), 7.81 – 7.79 (m, 2H, ArH), 7.06 (d, *J* = 7.9 Hz, 2H, ArH), 6.75 (d, *J* = 8.4 Hz, 2H, ArH), 4.06 (h, *J* = 6.0 Hz, 1H, CH), 3.76 (s, 3H, OMe CH_3), 3.58 (m, 1H, ArCH), 3.53 (t, *J* = 7.6 Hz, 2H, CH_2), 3.46 (ddd, *J* = 15.1, 10.7, 5.2 Hz, 1H, ArCH), 2.83 (t, *J* = 7.6 Hz, 2H, CH_2), 1.99 – 1.88 (m, 1H, CH_2), 1.80 (ddt, *J* = 15.8, 10.8, 5.2 Hz, 1H, CH_2), 1.21 (d, *J* = 6.3 Hz, 3H, CH_3), 0.99 (t, *J* = 8.0 Hz, 9H, $SiCH_2CH_3$), 0.69 (q, *J* = 7.9 Hz, 6H, $SiCH_2CH_3$).

^{13}C NMR (126 MHz, $CDCl_3$): δ 158.6, 148.0, 137.3, 135.1 (q, *J* = 34.7 Hz), 131.9, 129.89, 129.87, 128.5 (q, *J* = 3.6 Hz), 121.6 (q, *J* = 3.6 Hz), 122.2 (q, *J* = 273.4 Hz), 114.1, 78.9, 55.3, 49.3, 44.4, 34.0, 33.1, 18.4, 6.9, 3.9.

HRMS-ESI (m/z): $[M+Na]^+$ calculated for $[C_{26}H_{37}F_3N_2O_7SSiNa]^+$: 629.1940, found 629.1927.

***N*-(3-(*tert*-Butyldiphenylsilylperoxy)butyl)-*N*-(4-methoxyphenethyl)-2-nitro-4-(trifluoromethyl)benzenesulfonamide (36)**

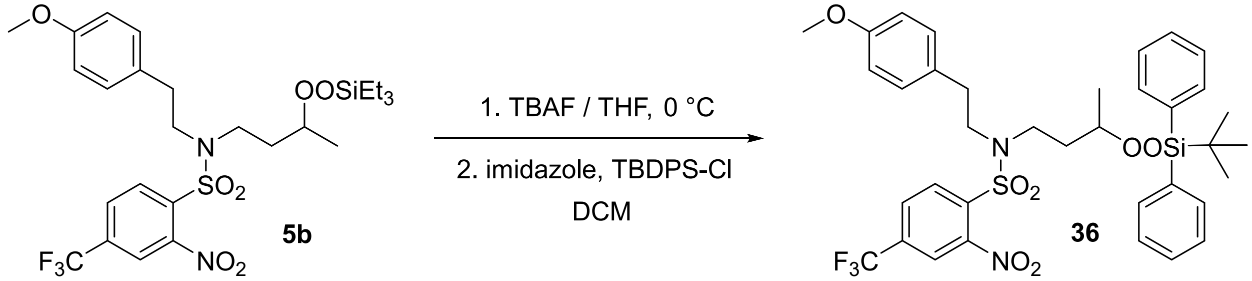

Following general procedure 7, TBAF (0.6 mL, 1.0 M in THF, 1.2 eq) was added to **5b** (300 mg, 0.49 mmol, 1 eq) in THF (3.0 mL, 0.2 M). Upon completion, the residue was dissolved in DCM (3.0 mL, 0.2 M) and treated with imidazole (50 mg, 0.74 mmol, 1.5 eq) and TBDPS-Cl (0.11 mL, 0.49 mmol, 1 eq). The crude product was purified by flash column chromatography on silica gel (eluent: 95:5 hexanes: EtOAc) to obtain the title compound **36** (308 mg, 0.43 mmol, 87% over 2 steps) as a colorless oil.

TLC: R_f = 0.33 (90:10 hexanes: EtOAc).

^{1}H NMR (500 MHz, C_6D_6): δ 7.86 (dd, *J* = 5.4, 2.2 Hz, 2H, ArH), 7.83 – 7.79 (m, 2H, ArH), 7.42 (d, *J* = 8.2 Hz, 1H, ArH), 7.27 – 7.18 (m, 6H, ArH), 7.10 (s, 1H, ArH), 6.82 (t, *J* = 9.3 Hz, 3H, ArH), 6.63 (d, *J* = 8.4 Hz, 2H, ArH), 3.95 (td, *J* = 6.8, 4.7 Hz, 1H, CH), 3.35 (t, *J* = 7.6 Hz, 2H, CH_2), 3.30 (s, 3H, OMe CH_3), 3.27 (d, *J* = 5.5 Hz, 1H, CH_2), 3.20 (ddt, *J* = 15.1, 10.5, 5.5 Hz, 1H, CH_2), 2.59 (t, *J* = 7.6 Hz, 2H, CH_2), 1.77 – 1.67 (m, 1H, CH_2), 1.66 – 1.56 (m, 1H, CH_2), 1.18 (s, 9H, *t*Bu), 1.02 (d, *J* = 6.2 Hz, 3H, CH_3).

^{13}C NMR (126 MHz, C_6D_6): δ 159.0, 148.2, 136.1, 134.6, 134.3, 133.2, 133.0 (q, *J* = 34.4 Hz), 132.9, 132.7, 131.6, 130.1 (q, *J* = 3.6 Hz), 129.8, 122.2 (q, *J* = 273.7 Hz), 120.8 (q, *J* = 3.9 Hz), 114.0, 79.3, 54.5, 49.2, 44.1, 33.9, 33.0, 27.2, 19.3, 18.1.

HRMS-ESI (m/z): $[M+Na]^+$ calculated for $[C_{36}H_{41}F_3N_2O_7SSiNa]^+$: 753.2254, found 753.2239.

2-Nitro-*N*-phenyl-4-(trifluoromethyl)benzenesulfonamide (S6)

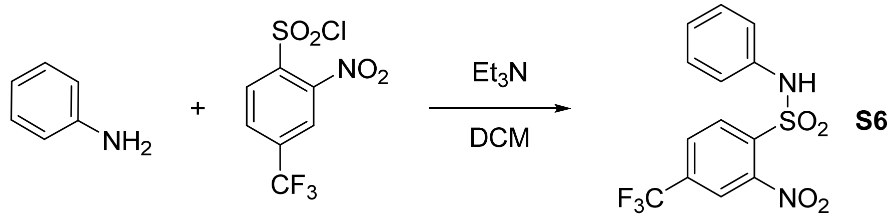

A stirred solution of aniline (0.24 mL, 2.6 mmol, 1.5 eq) in dry DCM (13 mL, 0.2 M) with triethylamine (0.25 mL, 1.73 mmol, 1 eq) was protected following general procedure 1B by treatment with 2-nitro-4-(trifluoromethyl)benzenesulfonyl chloride (500 mg, 1.73 mmol, 1 eq). The crude product was purified by flash column chromatography on silica gel (eluent: 95:5 hexanes: EtOAc) to obtain the title compound **S6** (520 mg, 1.5 mmol, 87%) as an orange solid.

TLC: R_f = 0.15 (90:10 hexanes: acetone).

^{1}H NMR (500 MHz, CDCl$_3$): δ 8.09 (s, 1H, ArH), 7.98 (d, *J* = 8.2 Hz, 1H, ArH), 7.82 (d, *J* = 8.2 Hz, 1H, ArH), 7.30 (t, *J* = 7.7 Hz, 2H, aniline ArH), 7.23 – 7.20 (m, 3H, aniline ArH).

^{13}C NMR (126 MHz, CDCl$_3$): δ 148.5, 136.2 (q, *J* = 34.9 Hz), 135.9, 135.0, 133.0, 129.8, 129.4 (q, *J* = 3.6 Hz), 127.2, 123.5, 122.7 (q, *J* = 3.8 Hz), 122.01 (q, *J* = 273.7 Hz).

HRMS-ESI (m/z): $[M+Na]^+$ calculated for $[C_{13}H_8F_3N_2O_4SNa]^+$: 369.0133, found 369.0124.

***N*-(But-3-en-1-yl)-2-nitro-*N*-phenyl-4-(trifluoromethyl)benzenesulfonamide (6a)**

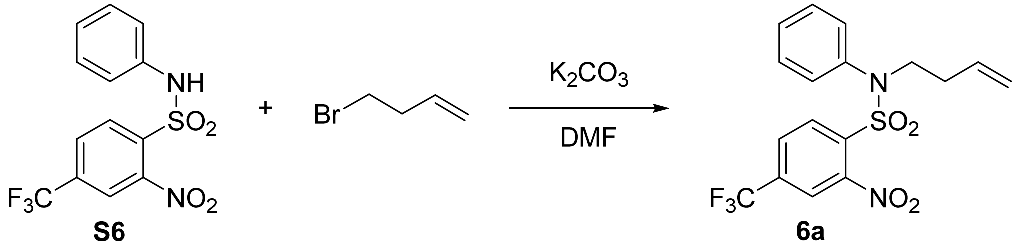

Following general procedure 2A, a stirred solution of **S6** (400 mg, 1.16 mmol, 1.0 eq) in DMF (6.0 mL, 0.2 M) was treated with K_2CO_3 (319 mg 2.31 mmol, 2 eq), followed by 4-bromobut-1-ene (0.23 mL 2.31 mmol, 2 eq). The crude residue was purified by flash column chromatography on silica gel (eluent: 93:7 hexanes: EtOAc) to obtain the title compound **6a** (450 mg, 1.13 mmol, 97%) as a yellow oil.

TLC: R_f = 0.41 (90:10 hexanes: acetone).

^{1}H NMR (500 MHz, $CDCl_3$): δ 7.85 (s, 1H, ArH), 7.69 (d, *J* = 8.4, 1.8 Hz, 1H, ArH), 7.60 (d, *J* = 8.3 Hz, 1H, ArH), 7.36 (d, *J* = 4.8, 1.8 Hz, 3H, aniline ArH), 7.24 – 7.18 (m, 2H, aniline ArH), 5.77 (ddt, *J* = 17.1, 10.4, 6.7 Hz, 1H, C*H*=CH_2), 5.10 (d, *J* = 8.6 Hz, 1H, =C*H*), 5.05 (s, 1H, =C*H*), 3.88 (t, *J* = 7.2 Hz, 2H, CH_2), 2.25 (q, *J* = 7.0 Hz, 2H, CH_2).

^{13}C {^{1}H, ^{19}F} NMR (126 MHz, $CDCl_3$): δ 148.2, 137.4, 135.8, 135.4, 134.2, 133.1, 129.8, 129.0, 127.9, 123.2, 121.4, 121.1, 117.9, 52.1, 33.2.

HRMS-ESI (m/z): $[M+Na]^+$ calculated for $[C_{17}H_{15}F_3N_2O_4SNa]^+$: 423.0602, found 409.0590.

2-Nitro-*N*-phenyl-*N*-(3-(triethylsilylperoxy)butyl)-4-(trifluoromethyl)benzenesulfonamide (6b)

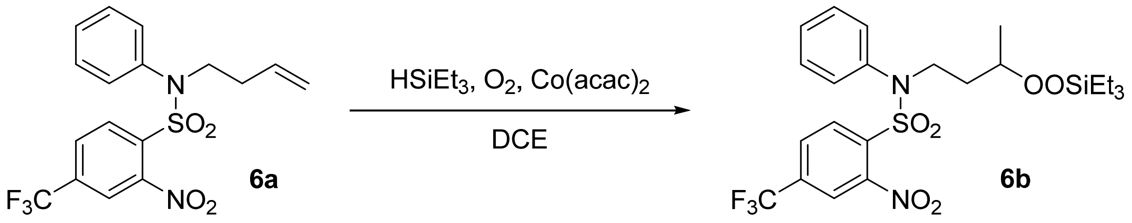

Following general procedure 6A, **6a** (460 mg, 1.15 mmol, 1 eq) in DCE (6.0 mL, 0.2 M) was treated with triethylsilane (0.46 mg, 2.87 mmol, 2.5 eq), and $Co(acac)_2$ (31 mg, 0.12 mmol, 10 mol%) under an atmosphere of oxygen. The crude product was then purified by flash column chromatography on silica gel (eluent: 95:5 hexanes: EtOAc) to obtain the title compound **6b** (394 g, 0.71 mmol, 62%) as a colorless oil.

TLC: R_f = 0.40 (90:10 hexanes: EtOAc).

^{1}H NMR (500 MHz, $CDCl_3$): δ 7.85 (s, 1H, ArH), 7.69 (d, *J* = 8.3, 1.8 Hz, 1H, ArH), 7.61 (d, *J* = 8.3 Hz, 1H, ArH), 7.39 – 7.32 (m, 3H, aniline ArH), 7.23 – 7.18 (m, 2H, aniline ArH), 4.10 (h, *J* = 6.2 Hz, 1H, CH), 3.96 (ddd, *J* = 13.6, 9.1, 6.4 Hz, 1H, CH_2), 3.86 (ddd, *J* = 14.1, 9.1, 5.4 Hz, 1H, CH_2), 1.90 (ddt, *J* = 13.3, 9.0, 6.5 Hz, 1H, CH_2), 1.64 (ddt, *J* = 14.3, 8.9, 5.5 Hz, 1H, CH_2), 1.20 (d, *J* = 6.3 Hz, 3H, CH_3), 0.95 (t, *J* = 8.0 Hz, 9H, $SiCH_2CH_3$), 0.64 (q, *J* = 8.0 Hz, 6H, $SiCH_2CH_3$).

^{13}C NMR (126 MHz, $CDCl_3$): δ 148.3, 137.5, 135.6, 135.3 (q, *J* = 35.3 Hz), 133.1, 129.7, 129.5, 129.0, 127.8 (q, *J* = 3.6 Hz), 122.1 (q, *J* = 273.7 Hz), 121.3 (q, *J* = 3.6 Hz), 78.8, 49.6, 33.8, 18.4, 6.8, 3.9.

HRMS-ESI (m/z): $[M+Na]^+$ calculated for $[C_{23}H_{31}F_3N_2O_6SSiNa]^+$: 571.1522, found 571.1512.

***N*-(3-(*tert*-Butyldiphenylsilylperoxy)butyl)-2-nitro-*N*-phenyl-4-(trifluoromethyl)benzene-sulfonamide (38)**

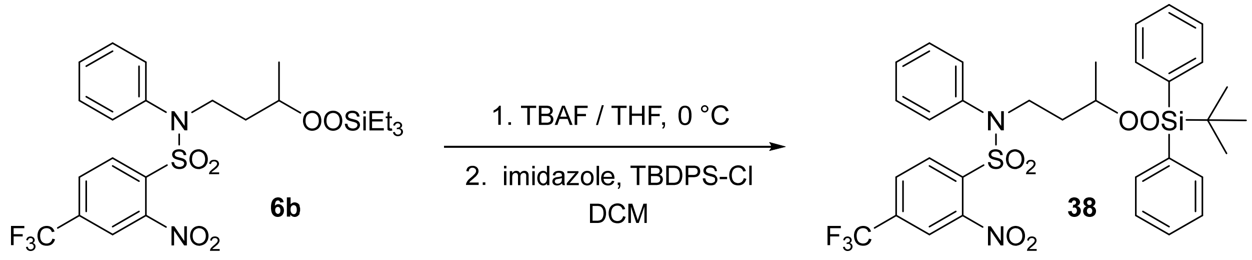

Following general procedure 7, TBAF (0.85 mL, 1.0 M in THF, 1.2 eq) was added to **6b** (390 mg, 0.71 mmol, 1 eq) in THF (4.0 mL, 0.2 M) Upon completion, the residue was dissolved in DCM (4.0 mL, 0.2 M) and treated with imidazole (73 mg, 1.07 mmol, 1.5 eq) and TBDPS-Cl (0.17 mL, 0.71 mmol, 1 eq). The crude product was purified by flash column chromatography on silica gel (eluent: 60:40 hexanes: DCM) to obtain the title compound **38** (410 mg, 0.61 mmol, 86% over 2 steps) as a colorless oil.

TLC: R*f* = 0.51 (50:50 hexanes: DCM).

^{1}H NMR (500 MHz, C_6D_6): δ 7.86 – 7.79 (m, 4H, ArH), 7.25 – 7.22 (m, 6H, ArH), 7.12 (d, *J* = 8.3 Hz, 1H, ArH), 7.09 (s, 1H, ArH), 7.04 – 6.98 (m, 2H, ArH), 6.91 – 6.89 (m, 3H, ArH), 6.56 (d, *J* = 8.3 Hz, 1H, ArH), 4.07 (h, *J* = 6.2 Hz, 1H, CH), 3.93 (ddd, *J* = 15.0, 9.1, 6.4 Hz, 1H, $C^{11}H$), 3.80 (ddd, *J* = 14.2, 9.3, 5.3 Hz, 1H, CH_2), 1.81 (ddt, *J* = 13.4, 9.1, 6.6 Hz, 1H, CH_2), 1.51 (ddt, *J* = 14.1, 9.7, 5.2 Hz, 1H, CH_2), 1.19 (s, 9H, *t*Bu), 0.99 (d, *J* = 6.2 Hz, 3H, CH_3).

^{13}C {^{1}H, ^{19}F} NMR (126 MHz, C_6D_6): δ 148.0, 137.9, 135.8, 134.5, 134.2, 133.0, 132.9, 129.9, 129.4, 129.3, 128.2, 127.0, 123.2, 121.0, 120.6, 79.2, 49.3, 33.6, 27.3, 19.3, 17.9.

HRMS-ESI (m/z): $[M+Na]^+$ calculated for $[C_{33}H_{35}F_3N_2O_6SSiNa]^+$: 695.1835, found 695.1822.

N-(2-Methylallyl)-N-(naphthalen-1-ylmethyl)-2-nitro-4-(trifluoromethyl)benzene-sulfonamide (3a)

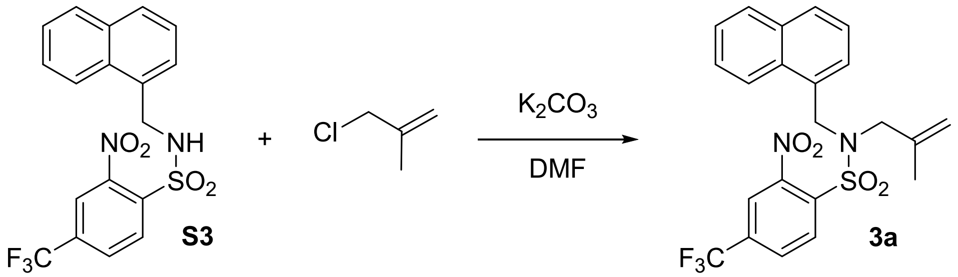

Following general procedure 2A, a stirred solution of **S3** (450 mg, 1.1 mmol, 1.0 eq) in DMF (6.0 mL, 0.2 M) was treated with K_2CO_3 (182 mg, 1.32 mmol, 1.2 eq), followed by 3-chloro-2-methylprop-1-ene (0.13 mL, 1.32 mmol, 1.2 eq). The crude residue was purified by flash column chromatography on silica gel (eluent: 95:5 hexanes: EtOAc) to obtain the title compound **3a** (430 mg, 0.924 mmol, 84%) as a yellow solid.

TLC: R_f = 0.37 (90:10 hexanes: EtOAc).

^{1}H NMR (500 MHz, $CDCl_3$): δ 7.88 (dt, *J* = 7.1, 3.6 Hz, 1H, ArH), 7.77 – 7.67 (m, 3H, ArH), 7.63 (d, *J* = 8.3 Hz, 1H, ArH), 7.46 – 7.38 (m, 4H, ArH), 7.33 (dd, *J* = 8.2, 7.0 Hz, 1H, ArH), 4.96 (s, 1H, =C*H*), 4.95 (s, 2H, $ArCH_2$), 4.92 (s, 1H, =C*H*), 4.13 (s, 2H, CH_2), 1.73 (s, 3H, CH_3).

^{13}C NMR (126 MHz, $CDCl_3$): δ 147.1, 139.8, 137.6, 134.7 (q, *J* = 34.7 Hz), 133.6, 131.8, 131.5, 130.3, 129.1, 128.7, 127.8 (q, *J* = 3.3 Hz), 127.8, 126.7, 126.1, 125.2, 123.0, 121.3 (q, *J* = 3.6 Hz), 120.9 (q, *J* = 271.9 Hz), 114.9, 55.5, 49.2, 20.0.

HRMS-ESI (m/z): $[M+Na]^+$ calculated for $[C_{21}H_{17}F_3N_2O_4SNa]^+$: 487.0915, found 487.0905.

***N*-(3-Hydroxy-2-methylpropyl)-*N*-(naphthalen-1-ylmethyl)-2-nitro-4-(trifluoromethyl)-benzenesulfonamide (3b)**

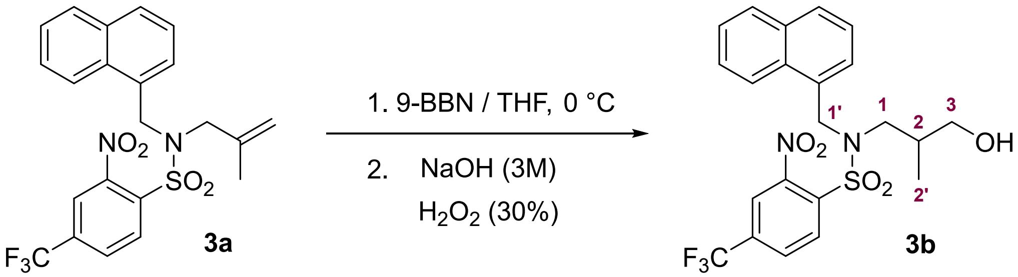

Following general procedure 3, a solution of **3a** (870 mg, 2.3 mmol, 1 eq) in dry THF (12 mL, 0.4 M) was treated with 9-BBN 0.5 M in THF (9.0 mL, 2 eq), 3 M NaOH (3.0 mL, 1.2 eq) and aq. 30% H_2O_2 (3.0 mL, 1.2 eq). The crude product was purified by flash column chromatography on silica gel (eluent: 75:25 hexanes: EtOAc) to obtain the title compound **3b** (477 mg, 1 mmol, 44%) as a colorless oil.

TLC: R*f* = 0.12 (80:20 hexanes: EtOAc).

^{1}H NMR (500 MHz, $CDCl_3$): δ 7.98 (d, *J* = 8.7 Hz, 1H, ArH), 7.73 (d, *J* = 8.2 Hz, 2H, ArH), 7.68 (s, 1H, ArH), 7.60 (d, *J* = 8.3 Hz, 1H, ArH), 7.48 – 7.39 (m, 4H, ArH), 7.34 (t, *J* = 7.6 Hz, 1H, ArH), 4.99 (d, *J* = 14.6 Hz, 1H, ArC$^{1'}$H), 4.86 (d, *J* = 14.6 Hz, 1H, ArC$^{1'}$H), 3.71 (dd, *J* = 14.7, 8.9 Hz, 1H, C^{1}H), 3.62 (dd, *J* = 11.5, 3.8 Hz, 1H, C^{1}H), 3.38 (dd, *J* = 11.5, 4.3 Hz, 1H, C^{3}H), 3.31 (dd, *J* = 14.7, 6.2 Hz, 1H, C^{3}H), 1.92 (br, 1H, OH), 1.69 (dtt, *J* = 8.5, 6.5, 4.2 Hz, 1H, C^{2}H), 0.87 (d, *J* = 6.9 Hz, 3H, C$^{2'}H_3$).

^{13}C NMR (126 MHz, $CDCl_3$): δ 147.4, 136.5, 134.7 (q, *J* = 34.7 Hz), 133.6, 131.65, 131.61, 130.2, 129.5, 128.8, 128.4, 127.8, (q, *J* = 3.6 Hz), 126.9, 126.3, 125.1, 123.2 (q, *J* = 273.7 Hz), 121.3 (q, *J* = 3.9 Hz), 120.9, 63.9, 53.1, 51.4, 34.6, 14.5.

HRMS-ESI (m/z): $[M+Na]^+$ calculated for $[C_{22}H_{21}F_3N_2O_5SNa]^+$: 505.1021, found 505.0993.

***N*-(3-Iodo-2-methylpropyl)-*N*-(naphthalen-1-ylmethyl)-2-nitro-4-(trifluoromethyl)-benzenesulfonamide (3c)**

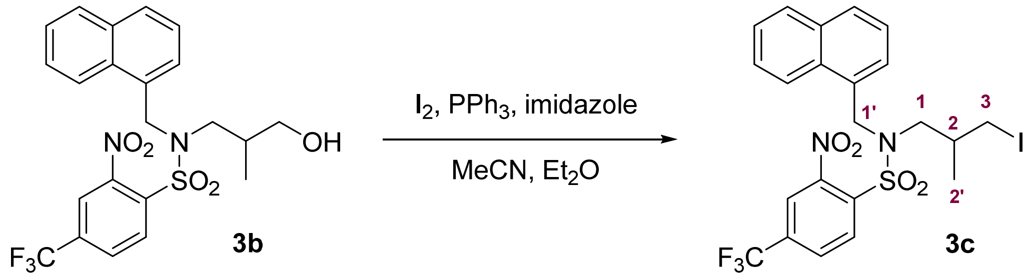

Following general procedure 4, triphenylphosphine (325 mg, 1.2 mmol, 1.5 eq), imidazole (84 mg, 1.2 mmol, 1.5 eq) and iodine (315 mg, 1.2 mmol, 1.5 eq) were added subsequently to a solution of **3b** (400 mg, 0.83 mmol, 1 eq) in a 1:3 ratio of MeCN and Et_2O (4.0 mL, 0.2 M) at RT. The crude product was purified by flash column chromatography on silica gel (eluent: 94:6 hexanes: EtOAc) to obtain the title compound **3c** (370 mg, 0.62 mmol, 75%) as a colorless oil.

TLC: R_f = 0.28 (90:10 hexanes: EtOAc).

^{1}H NMR (500 MHz, $CDCl_3$): δ 8.03 (d, *J* = 8.0 Hz, 1H, ArH), 7.79 – 7.71 (m, 4H, ArH), 7.56 (d, *J* = 8.2 Hz, 1H, ArH), 7.47 (p, *J* = 6.9 Hz, 2H, ArH), 7.42 (d, *J* = 6.9 Hz, 1H, ArH), 7.36 (t, *J* = 7.6 Hz, 1H, ArH), 4.98 (d, *J* = 14.6 Hz, 1H, $ArC^{1'}H$), 4.88 (d, *J* = 14.6 Hz, 1H, $ArC^{1'}H$), 3.49 (dd, *J* = 14.6, 7.0 Hz, 1H, C^1H), 3.38 (dd, *J* = 14.6, 7.5 Hz, 1H, C^1H), 3.12 (dd, *J* = 10.2, 5.0 Hz, 1H, C^3H), 2.97 (dd, *J* = 9.8, 6.5 Hz, 1H, C^3H), 1.58 (dq, *J* = 12.4, 5.7 Hz, 1H, C^2H), 0.86 (d, *J* = 7.0 Hz, 3H, $C^{2'}H_3$).

^{13}C NMR (126 MHz, $CDCl_3$): δ 147.5, 136.2 (q, *J* = 34.3 Hz), 135.1, 133.7, 131.9, 131.5, 129.9, 129.6, 128.8, 128.3, 127.6 (q, *J* = 3.5 Hz), 126.9, 126.3, 125.2, 123.1 (q, *J* = 274.1 Hz), 121.3 (q, *J* = 3.8 Hz), 120.9, 55.3, 51.7, 34.3, 18.6, 12.4.

HRMS-ESI (m/z): $[M+Na]^+$ calculated for $[C_{22}H_{20}F_3IN_2O_4SNa]^+$: 615.0038, found 615.0020.

***N*-(3-Hydroperoxy-2-methylpropyl)-*N*-(naphthalen-1-ylmethyl)-2-nitro-4-(trifluoro-methyl)benzenesulfonamide (3d)**

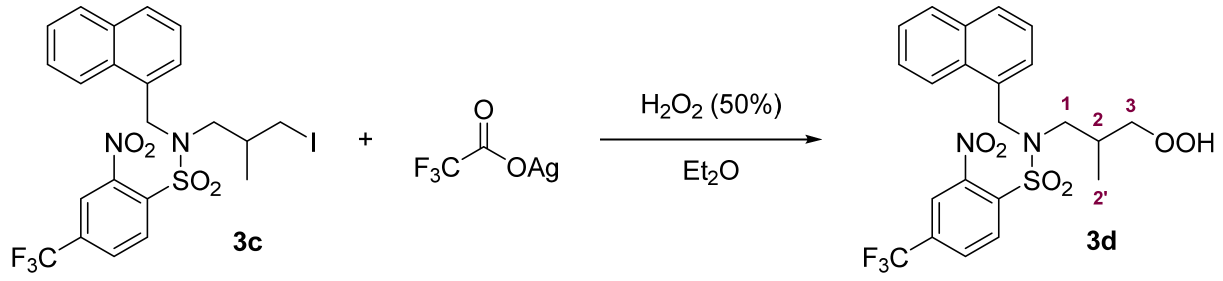

Following general procedure 5, **3c** (300 mg, 0.51 mmol, 1 eq) in diethyl ether (3.0 mL, 0.2 M) was treated with silver trifluoroacetate (134 mg, 0.61 mmol, 1.2 eq) and aqueous 50% hydrogen peroxide (0.6 mL, 2 eq). The crude hydroperoxide was then purified by flash column chromatography on silica gel (eluent: 70:30 hexanes: EtOAc) to obtain the title compound **3d** (106 mg, 0.21 mmol, 42%) as a colorless oil.

TLC: R_f = 0.21 (80:20 hexanes: EtOAc).

^{1}H NMR (500 MHz, $CDCl_3$): δ 8.09 (br, OOH, 1H), 7.99 (d, J = 8.0 Hz, 1H, ArH), 7.74 (d, J = 7.9 Hz, 2H, ArH), 7.71 – 7.64 (m, 2H, ArH), 7.49 (d, J = 8.6 Hz, 1H, ArH), 7.47 – 7.42 (m, 3H, ArH), 7.36 (t, J = 7.6 Hz, 1H, ArH), 4.95 (s, 2H, $ArCH_2$), 3.85 (qd, J = 10.8, 6.0 Hz, 2H, C^1H_2), 3.65 (dd, J = 14.7, 7.0 Hz, 1H, C^3H), 3.39 (dd, J = 14.7, 7.7 Hz, 1H, C^3H), 2.10 (dq, J = 12.1, 6.7 Hz, 1H, C^2H), 0.85 (d, J = 6.9 Hz, 3H, $C^{2'}H_3$).

^{13}C {^{1}H, ^{19}F} NMR (126 MHz, $CDCl_3$): δ 147.4, 136.8, 134.9, 133.8, 132.0, 131.8, 130.4, 129.7, 128.9, 128.6, 128.0, 127.1, 126.4, 125.3, 123.4, 121.4, 121.0, 79.8, 53.8, 51.5, 31.5, 15.0.

HRMS-ESI (m/z): $[M+Na]^+$ calculated for $[C_{22}H_{21}F_3N_2O_6SNa]^+$: 521.0970, found 521.0940.

N-(3-(*tert*-Butyldiphenylsilylperoxy)-2-methylpropyl)-N-(naphthalen-1-ylmethyl)-2-nitro-4-(trifluoromethyl)benzenesulfonamide (40)

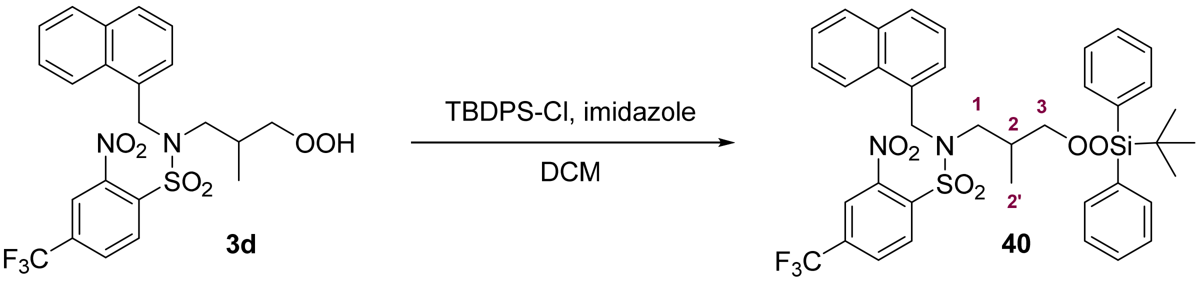

Following general procedure 8, **3d** (125 mg, 0.25 mmol, 1 eq) in DCM (2.0 mL, 0.2 M) was treated with imidazole (26 mg, 0.38 mmol, 1.5 eq) and TBDPS-Cl (0.07 mL, 0.25 mmol, 1 eq). The crude product was then purified by flash column chromatography on silica gel (eluent: 96:4 hexanes: acetone) to obtain the title compound **40** (160 mg, 0.22 mmol, 87%) as a colorless oil.

TLC: R_f = 0.19 (90:10 hexanes: acetone).

^{1}H NMR (500 MHz, C_6D_6): δ 7.95 (d, J = 8.3 Hz, 1H, ArH), 7.83 – 7.77 (m, 4H, ArH), 7.45 (d, J = 8.0 Hz, 1H, ArH), 7.38 (d, J = 8.2 Hz, 1H, ArH), 7.22 – 7.18 (m, 8H, ArH), 7.13 (d, J = 9.9 Hz, 2H, ArH), 7.03 – 6.94 (m, 2H, ArH), 6.54 (d, J = 8.1 Hz, 1H, ArH), 4.77 – 4.66 (s, 2H, $ArCH_2$), 3.68 – 3.61 (m, 2H, C^1H_2), 3.43 – 3.27 (m, 2H, C^3H_2), 1.96 (dq, J = 12.1, 6.5 Hz, 1H, C^2H), 1.16 (s, 9H, *t*Bu), 0.61 (d, J = 6.6 Hz, 3H, $C^{2'}H_3$).

^{13}C {^{1}H, ^{19}F} NMR (126 MHz, C_6D_6): δ 147.5, 136.6, 135.8, 133.9, 133.7, 132.74, 132.72, 131.7, 131.6, 130.5, 130.0, 129.0, 128.5, 127.0, 126.6, 126.0, 124.9, 123.3, 123.2, 120.66, 120.61, 79.4, 53.0, 50.7, 31.5, 27.2, 19.3, 14.5.

HRMS-ESI (m/z): $[M+Na]^+$ calculated for $[C_{38}H_{39}F_3N_2O_6SSiNa]^+$: 759.2148, found 759.2117.

N-Allyl-N-(naphthalen-1-ylmethyl)-2-nitro-4-(trifluoromethyl)benzenesulfonamide (3e)

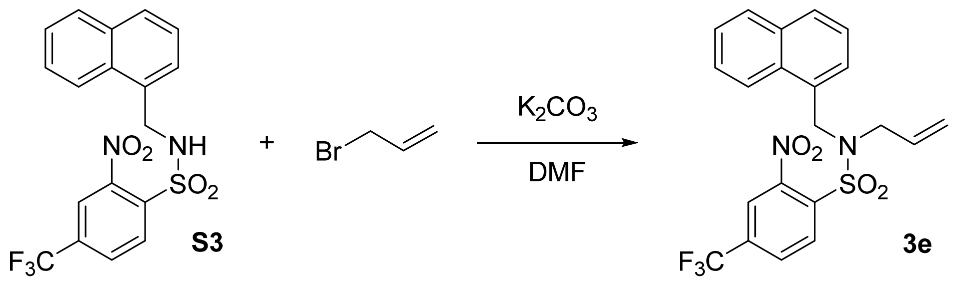

Following general procedure 2A, a stirred solution of **S3** (1.2 g, 3.02 mmol, 1 eq) in DMF (15 mL, 0.2 M) was treated with K_2CO_3 (500 mg 3.63 mmol, 1.2 eq), followed by 3-bromoprop-1-ene (0.32 mL, 3.63 mmol, 1.2 eq). The crude residue was purified by flash column chromatography on silica gel (eluent: 90:10 hexanes: EtOAc) to obtain the title compound **3e** (1.3 g, 2.84 mmol, 94%) as a yellow solid.

TLC: R_f = 0.39 (90:10 hexanes: EtOAc).

^{1}H NMR (500 MHz, $CDCl_3$): δ 8.00 – 7.93 (m, 1H, ArH), 7.76 (q, *J* = 8.1 Hz, 4H, ArH), 7.56 (d, *J* = 8.3 Hz, 1H, ArH), 7.48 – 7.40 (m, 3H, ArH), 7.36 (dd, *J* = 8.3, 6.9 Hz, 1H, ArH), 5.74 (ddt, *J* = 16.7, 10.2, 6.2 Hz, 1H, C*H*=CH_2), 5.20 (d, *J* = 7.4 Hz, 1H, =C*H*), 5.16 (s, 1H, =C*H*), 4.96 (s, 2H, ArCH_2), 4.06 (d, *J* = 6.2 Hz, 2H, CH_2).

^{13}C NMR (126 MHz, $CDCl_3$): δ 147.5, 137.4, 135.2, 133.8 (q, *J* = 34.7 Hz), 132.2, 132.0, 131.6, 130.2, 129.3, 128.8, 128.1, 128.0 (q, *J* = 3.6 Hz), 126.8, 126.2, 125.2, 123.1 (q, *J* = 273.7 Hz), 121.5 (q, *J* = 3.9 Hz), 121.0, 120.0, 51.4, 49.7.

HRMS-ESI (m/z): $[M+Na]^+$ calculated for $[C_{21}H_{17}F_3N_2O_4SNa]^+$: 473.0759, found 473.0810.

***N*-(3-Hydroxypropyl)-*N*-(naphthalen-1-ylmethyl)-2-nitro-4-(trifluoromethyl)-benzenesulfonamide (3f)**

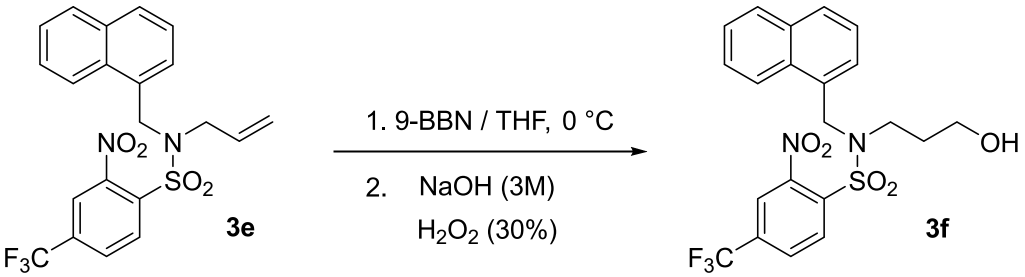

Following general procedure 3, **3e** (180 mg, 0.47 mmol, 1 eq) in dry THF (2.0 mL, 0.4 M) was treated with 9-BBN 0.5 M in THF (1.9 mL, 2 eq), 3 M NaOH (0.64 mL, 1.2 eq) and aq. 30% H_2O_2 (0.64 mL, 1.2 eq). The crude product was purified by flash column chromatography on silica gel (eluent: 60:40 hexanes: EtOAc) to obtain the title compound **3f** (132 mg, 0.31 mmol, 67%) as a colorless oil.

TLC: R*f* = 0.31 (60:40 hexanes: EtOAc).

^{1}H NMR (500 MHz, $CDCl_3$): δ 8.03 (d, *J* = 8.0 Hz, 1H, ArH), 7.81 – 7.72 (m, 4H, ArH), 7.57 (d, *J* = 8.3 Hz, 1H, ArH), 7.46 (dd, *J* = 17.0, 6.8 Hz, 3H, ArH), 7.37 (t, *J* = 7.6 Hz, 1H, ArH), 4.95 (s, 2H, $ArCH_2$), 3.63 (t, *J* = 6.9 Hz, 2H, CH_2), 3.55 (t, *J* = 5.7 Hz, 2H, CH_2), 1.65 (br, 1H, OH), 1.60 (p, *J* = 6.4 Hz, 2H, CH_2).

^{13}C NMR (126 MHz, $CDCl_3$): δ 147.7, 136.6, 135.0 (q, *J* = 34.3 Hz), 133.8, 131.8, 131.7, 130.3, 129.6, 128.9, 128.4, 128.0 (q, *J* = 3.7 Hz), 127.0, 126.4, 125.2, 123.3 (q, *J* = 273.5 Hz), 121.5 (q, *J* = 3.7 Hz), 121.0, 59.1, 51.1, 46.7, 31.3.

HRMS-ESI (m/z): $[M+Na]^+$ calculated for $[C_{21}H_{19}F_3N_2O_5SNa]^+$: 491.0864, found 491.0835.

***N*-(3-Iodopropyl)-*N*-(naphthalen-1-ylmethyl)-2-nitro-4-(trifluoromethyl)-benzenesulfonamide (3g)**

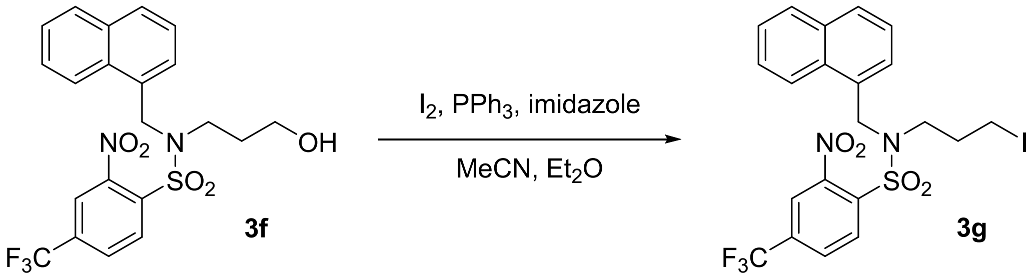

Following general procedure 4, triphenylphosphine (84 mg, 0.32 mmol, 1.5 eq), imidazole (22 mg, 0.32 mmol, 1.5 eq) and iodine (81 mg, 0.32 mmol, 1.5 eq) were added subsequently to a solution of **3f** (100 mg, 0.21 mmol, 1 eq) in a 1:3 ratio of MeCN and Et_2O (1.0 mL, 0.2 M) at RT. The crude product was purified by flash column chromatography on silica gel (eluent: 94:6 hexanes: EtOAc) to obtain the title compound **3g** (113 mg, 0.2 mmol, 92%) as a colorless oil.

TLC: R_f = 0.55 (80:20 hexanes: EtOAc).

^{1}H NMR (500 MHz, $CDCl_3$): δ 8.06 (d, *J* = 8.0 Hz, 1H, ArH), 7.87 (d, *J* = 8.2 Hz, 1H, ArH), 7.80 (d, *J* = 12.6 Hz, 3H, ArH), 7.66 (d, *J* = 8.3 Hz, 1H, ArH), 7.54 – 7.43 (m, 3H, ArH), 7.43 – 7.37 (m, 1H, ArH), 4.96 (s, 2H, $ArCH_2$), 3.53 – 3.46 (m, 2H, CH_2), 2.98 (t, *J* = 6.7 Hz, 2H, CH_2), 1.86 (p, *J* = 6.9 Hz, 2H, CH_2).

^{13}C NMR (126 MHz, $CDCl_3$): δ 171.2, 147.7, 136.3, 135.0 (q, *J* = 34.5 Hz), 133.7, 131.9, 130.0, 129.6, 128.8, 128.3, 128.2 (q, *J* = 3.7 Hz), 127.0, 126.3, 125.2, 123.1 (q, *J* = 273.7 Hz), 121.5 (q, *J* = 3.9 Hz), 120.9, 51.3, 50.0, 32.2, 1.6.

HRMS-ESI (m/z): $[M+Na]^+$ calculated for $[C_{21}H_{18}F_3IN_2O_4SNa]^+$: 600.9882, found 600.9854.

***N*-(3-Hydroperoxypropyl)-*N*-(naphthalen-1-ylmethyl)-2-nitro-4-(trifluoromethyl)-benzenesulfonamide (3h)**

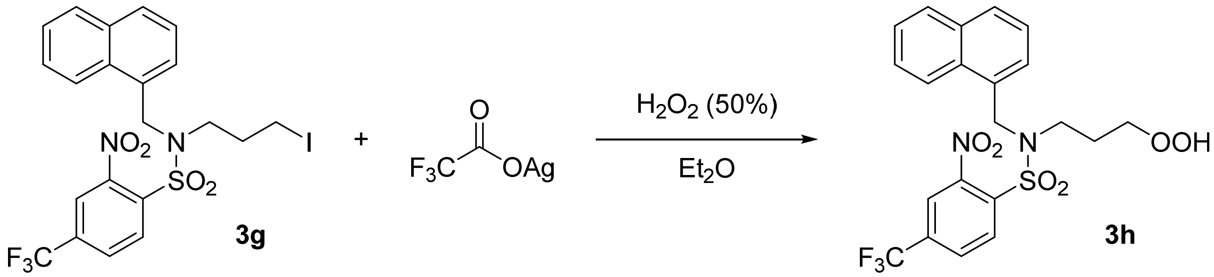

Following general procedure 5, **3g** (113 mg, 0.2 mmol, 1 eq) in diethyl ether (1.0 mL, 0.2 M) was treated with silver trifluoroacetate (46 mg, 0.24 mmol, 1.2 eq) and aqueous 50% hydrogen peroxide (0.2 mL, 2 eq). The crude residue was then purified by flash column chromatography on silica gel (eluent: 70:30 hexanes: EtOAc) to obtain the title compound **3h** (45 mg, 0.1 mmol, 47%) as a colorless oil.

TLC: R_f = 0.16 (80:20 hexanes: EtOAc).

^{1}H NMR (500 MHz, $CDCl_3$): δ 8.06 (d, *J* = 7.8 Hz, 1H, ArH), 7.95 (s, 1H, ArH), 7.84 (d, *J* = 8.3 Hz, 1H, ArH), 7.78 (d, *J* = 8.0 Hz, 3H, ArH), 7.66 – 7.61 (m, 1H, ArH), 7.53 – 7.43 (m, 3H, ArH), 7.42 – 7.36 (m, 1H, ArH), 4.98 (s, 2H, $ArCH_2$), 3.87 (t, *J* = 5.8 Hz, 2H, CH_2), 3.57 – 3.50 (m, 2H, CH_2), 1.73 (dq, *J* = 11.6, 6.0 Hz, 2H, CH_2).

^{13}C NMR (126 MHz, $CDCl_3$): δ 147.7, 136.6, 135.1 (q, *J* = 34.7 Hz), 133.8, 132.0, 131.7, 130.3, 129.6, 128.9, 128.3, 128.2 (q, *J* = 3.6 Hz), 127.1, 126.4, 125.2, 123.3 (q, *J* = 273.7 Hz), 121.5 (q, *J* = 3.9 Hz), 121.0, 73.8, 51.1, 46.4, 27.1.

HRMS-ESI (m/z): $[M+Na]^+$ calculated for $[C_{21}H_{19}F_3N_2O_6SNa]^+$: 507.0814, found 507.0788.

***N*-(3-(*tert*-Butyldiphenylsilylperoxy)propyl)-*N*-(naphthalen-1-ylmethyl)-2-nitro-4-(trifluoromethyl)benzenesulfonamide (42)**

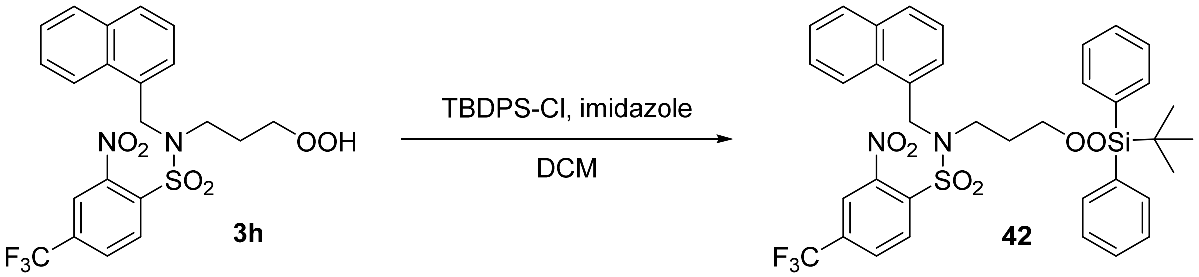

Following general procedure 8, **3h** (55 mg, 0.1 mmol, 1 eq) in DCM (0.5 mL, 0.2 M) was treated with imidazole (9 mg, 0.14 mmol, 1.2 eq) and TBDPS-Cl (0.03 mL, 0.1 mmol, 1 eq). The crude product was then purified by flash column chromatography on silica gel (eluent: 90:10 hexanes: acetone) to obtain the title compound **42** (78 mg, 0.12 mmol, 96%) as a colorless oil.

TLC: R_f = 0.27 (90:10 hexanes: acetone).

^{1}H NMR (500 MHz, C_6D_6): δ 8.06 (d, *J* = 8.4 Hz, 1H, ArH), 7.76 (dd, *J* = 6.9, 2.0 Hz, 4H, ArH), 7.51 (d, *J* = 8.1 Hz, 1H, ArH), 7.45 (d, *J* = 8.2 Hz, 1H, ArH), 7.29 (d, *J* = 8.2 Hz, 1H, ArH), 7.25 – 7.17 (m, 9H, ArH), 7.07 – 6.98 (m, 2H, ArH), 6.62 (d, *J* = 8.5 Hz, 1H, ArH), 4.75 (s, 2H, $ArCH_2$), 3.55 (t, *J* = 5.7 Hz, 2H, CH_2), 3.23 – 3.17 (m, 2H, CH_2), 1.46 (dq, *J* = 11.4, 5.7 Hz, 2H, CH_2), 1.13 (s, 9H, *t*Bu).

^{13}C {^{1}H, ^{19}F} NMR (126 MHz, C_6D_6): δ 136.9, 136.0, 135.3, 134.1, 133.0, 132.1, 132.0, 130.9, 130.3, 129.8, 129.3, 128.9, 128.4, 128.0, 127.6, 127.0, 126.3, 125.3, 123.7, 121.0, 73.9, 50.6, 46.2, 27.5, 27.1, 26.8, 19.5.

HRMS-ESI (m/z): $[M+Na]^+$ calculated for $[C_{37}H_{37}F_3N_2O_6SSiNa]^+$: 745.1991, found 745.1957.

N-Allyl-2-nitro-N-phenyl-4-(trifluoromethyl)benzenesulfonamide (6c)

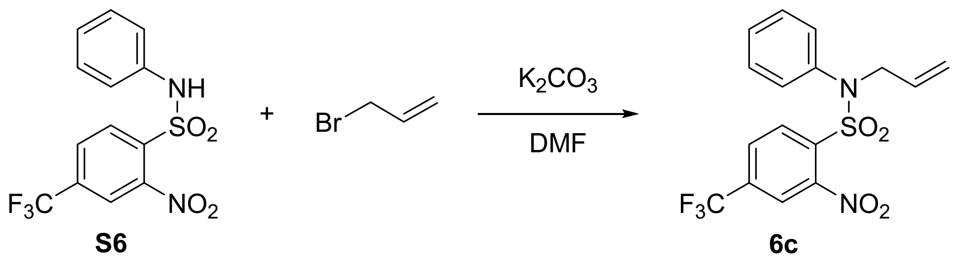

Following general procedure 2A, a stirred solution of **S6** (1 g, 2.9 mmol, 1 eq) in DMF (14 mL, 0.2 M) was treated with K_2CO_3 (600 mg 4.33 mmol, 1.5 eq), followed by 3-bromoprop-1-ene (0.37 mL, 4.33 mmol, 1.5 eq). The crude residue was purified by flash column chromatography on silica gel (eluent: 96:4 hexanes: EtOAc) to obtain the title compound **6c** (1 g, 2.6 mmol, 90%) as a white solid.

TLC: R_f = 0.43 (90:10 hexanes: EtOAc).

^{1}H NMR (500 MHz, $CDCl_3$): δ 7.88 (s, 1H, ArH), 7.71 (d, J = 8.3 Hz, 1H, ArH), 7.66 (d, J = 8.3 Hz, 1H, ArH), 7.38 – 7.30 (m, 3H, aniline ArH), 7.18 (dd, J = 6.1, 2.5 Hz, 2H, aniline ArH), 5.82 (ddt, J = 16.6, 9.9, 6.4 Hz, 1H, C*H*=CH_2), 5.14 (d, J = 4.8 Hz, 1H, =C*H*), 5.11 (s, 1H, =C*H*), 4.41 (d, J = 6.4 Hz, 2H, CH_2).

^{13}C {^{1}H, ^{19}F} NMR (126 MHz, $CDCl_3$): δ 137.8, 136.2, 136.0, 135.7, 133.4, 132.9, 130.1, 129.9, 129.3, 128.2, 121.7, 121.3, 120.0, 55.9.

HRMS-ESI (m/z): $[M+Na]^+$ calculated for $[C_{16}H_{13}F_3N_2O_4SNa]^+$: 409.0446, found 409.0447.

***N*-(3-Hydroxypropyl)-2-nitro-*N*-phenyl-4-(trifluoromethyl)benzenesulfonamide (6d)**

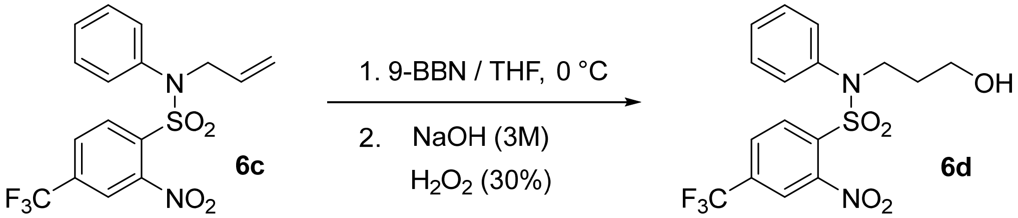

Following general procedure 3, **6c** (1.4 g, 3.62 mmol, 1 eq) in dry THF (9.0 mL, 0.4 M) was treated with 9-BBN 0.5 M in THF (14.5 mL, 2 eq), 3 M NaOH (5.0 mL, 1.2 eq) and aq. 30% H_2O_2 (5.0 mL, 1.2 eq). The crude product was purified by flash column chromatography on silica gel (eluent: 50:50 hexanes: EtOAc) to obtain the title compound **6d** (682 mg, 1.7 mmol, 47%) as a colorless oil.

TLC: R*f* = 0.10 (80:20 hexanes: EtOAc).

^{1}H NMR (500 MHz, $CDCl_3$): δ 7.86 (s, 1H, ArH), 7.72 (d, *J* = 8.3, 1.8 Hz, 1H, ArH), 7.64 (d, *J* = 8.3 Hz, 1H, ArH), 7.36 (dd, *J* = 5.0, 2.1 Hz, 3H, aniline ArH), 7.25 – 7.18 (m, 2H, aniline ArH), 3.95 (t, *J* = 6.6 Hz, 2H, CH_2), 3.75 (t, *J* = 5.9 Hz, 2H, CH_2), 1.99 (br, 1H, OH), 1.71 (p, *J* = 6.2 Hz, 2H, CH_2).

^{13}C NMR (126 MHz, $CDCl_3$): δ 148.1, 137.4, 136.0 (q, *J* = 34.9 Hz), 135.5, 133.0, 129.8, 129.5(q, *J* = 3.6 Hz), 129.1, 128.0, 123.2 (q, *J* = 3.8 Hz), 121.4 (q, *J* = 273.7 Hz), 59.0, 49.5, 31.2.

HRMS-ESI (m/z): $[M+Na]^+$ calculated for $[C_{16}H_{15}F_3N_2O_5SNa]^+$: 427.0551, found 427.0511.

_N_-(3-Iodopropyl)-2-nitro-_N_-phenyl-4-(trifluoromethyl)benzenesulfonamide (6e)

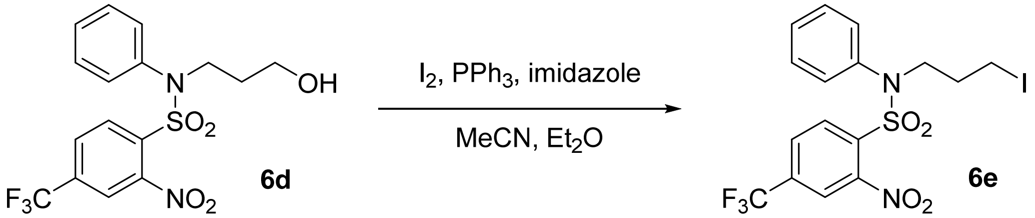

Following general procedure 4, triphenylphosphine (150 mg, 0.57 mmol, 1.5 eq), imidazole (39 mg, 0.57 mmol, 1.5 eq) and iodine (145 mg, 0.57 mmol, 1.5 eq) were added subsequently to a solution of **6d** (154 mg, 0.38 mmol, 1 eq) in a 1:3 ratio of MeCN and Et_2O (2.0 mL, 0.2 M) at RT. The crude product was purified by flash column chromatography on silica gel (eluent: 94:6 hexanes: EtOAc) to obtain the title compound **6e** (150 mg, 0.29 mmol, 77%) as a colorless oil.

TLC: R_f = 0.40 (90:10 hexanes: EtOAc).

^{1}H NMR (500 MHz, $CDCl_3$): δ 7.87 (s, 1H, ArH), 7.71 (d, *J* = 8.3, 1.8 Hz, 1H, ArH), 7.62 (d, *J* = 8.3 Hz, 1H, ArH), 7.37 (m, 3H, aniline ArH), 7.22 (dd, *J* = 6.7, 3.0 Hz, 2H, aniline ArH), 3.90 (t, *J* = 6.8 Hz, 2H, CH_2), 3.19 (t, *J* = 7.0 Hz, 2H, CH_2), 2.04 (p, *J* = 6.9 Hz, 2H, CH_2).

^{13}C NMR (126 MHz, $CDCl_3$): δ 148.2, 137.4, 135.8 (q, *J* = 35.1 Hz), 135.3, 133.2, 129.9, 129.3 (q, *J* = 3.7 Hz), 129.2, 128.0, 123.2 (q, *J* = 3.6 Hz), 121.4 (q, *J* = 273.6 Hz), 53.1, 32.8, 1.0.

HRMS-ESI (m/z): $[M+Na]^+$ calculated for $[C_{16}H_{14}F_3IN_2O_4SNa]^+$: 536.9569, found 536.9570.

***N*-(3-Hydroperoxypropyl)-2-nitro-*N*-phenyl-4-(trifluoromethyl)benzenesulfonamide (6f)**

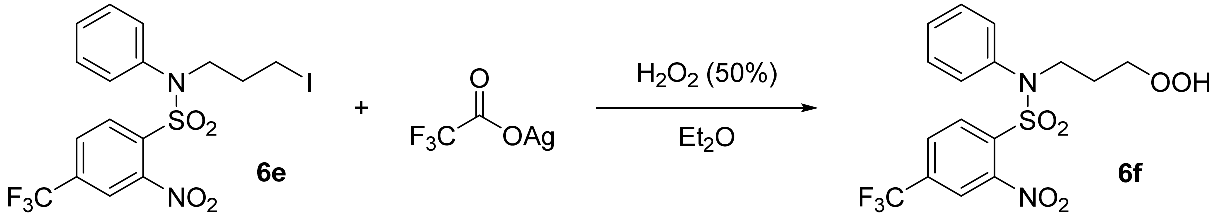

Following general procedure 5, **6e** (150 mg, 0.29 mmol, 1 eq) in diethyl ether (2.0 mL, 0.2 M) was treated with silver trifluoroacetate (77 mg, 0.35 mmol, 1.2 eq) and aqueous 50% hydrogen peroxide (0.34 mL, 2 eq). The crude residue was then purified by flash column chromatography on silica gel (eluent: 75:25 hexanes: EtOAc) to obtain the title compound **6f** (52 mg, 0.12 mmol, 43%) as a colorless oil.

TLC: R_f = 0.19 (80:20 hexanes: EtOAc).

^{1}H NMR (500 MHz, $CDCl_3$): δ 8.31 (br, 1H, OOH), 7.86 (s, 1H, ArH), 7.71 (d, *J* = 8.3, 1.8 Hz, 1H, ArH), 7.64 (d, *J* = 8.3 Hz, 1H, ArH), 7.36 (m, 3H, aniline ArH), 7.24 – 7.20 (m, 2H, aniline ArH), 4.10 (t, *J* = 6.7, 5.3 Hz, 2H, CH_2), 3.93 (t, *J* = 7.0 Hz, 2H, CH_2), 1.86 (p, *J* = 6.4 Hz, 2H, CH_2).

^{13}C NMR (126 MHz, $CDCl_3$): δ 148.2, 137.4, 135.8 (q, *J* = 34.8 Hz), 135.5, 133.1, 129.9, 129.5 (q, *J* = 3.7 Hz), 129.1, 128.0, 123.2 (q, *J* = 3.9 Hz), 121.4 (q, *J* = 273.6 Hz), 73.8, 49.8, 27.1.

HRMS-ESI (m/z): $[M+Na]^+$ calculated for $[C_{16}H_{15}F_3N_2O_6SNa]^+$: 443.0501, found 443.0476.

***N*-(3-(*tert*-Butyldiphenylsilylperoxy)propyl)-2-nitro-*N*-4-(trifluoromethyl)-benzenesulfonamide (44)**

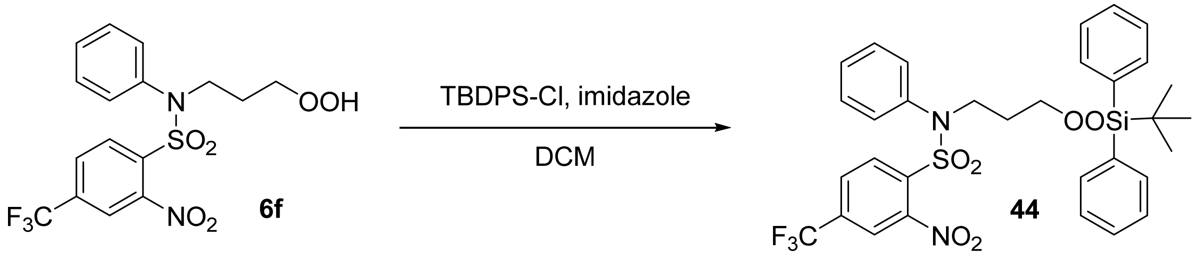

Following general procedure 8, **6f** (179 mg, 0.43 mmol, 1 eq) in DCM (3.0 mL, 0.2 M) was treated with imidazole (44 mg, 0.64 mmol, 1.5 eq) and TBDPS-Cl (0.1 mL, 0.43 mmol, 1 eq). The crude product was then purified by flash column chromatography on silica gel (eluent: 97:3 hexanes: EtOAc) to obtain the title compound **44** (243 mg, 0.37 mmol, 87%) as a colorless oil.

TLC: R_f = 0.31 (90:10 hexanes: EtOAc).

^{1}H NMR (500 MHz, C_6D_6): δ 7.74 (d, *J* = 6.7 Hz, 4H, ArH), 7.26 (d, *J* = 6.1 Hz, 6H, ArH), 7.12 (d, *J* = 8.3 Hz, 1H, ArH), 7.07 (s, 1H, ArH), 7.02 – 6.96 (m, 2H, ArH), 6.94 – 6.87 (m, 3H, ArH), 6.54 (d, *J* = 8.2 Hz, 1H, ArH), 4.03 (t, *J* = 7.1 Hz, 2H, CH_2), 3.57 (t, *J* = 5.8 Hz, 2H, CH_2), 1.59 (p, *J* = 6.1 Hz, 2H, CH_2), 1.16 (s, 9H, *t*Bu).

^{13}C NMR (126 MHz, C_6D_6): δ 148.1, 138.2, 135.7, 135.3 (q, *J* = 34.8 Hz), 135.0, 133.8, 132.8, 129.7, 129.5 (q, *J* = 3.6 Hz), 129.3, 129.2, 128.2, 126.9, 120.6 (q, *J* = 273.7 Hz), 120.5 (q, *J* = 3.9 Hz), 60.6, 49.6, 31.8, 26.8, 19.1.

HRMS-ESI (m/z): $[M+Na]^+$ calculated for $[C_{32}H_{33}F_3N_2O_6SSiNa]^+$: 681.1678, found 681.1648.

***N*-(3-Methylbut-3-en-1-yl)-*N*-(naphthalen-1-ylmethyl)-2-nitro-4-(trifluoromethyl)-benzenesulfonamide (3i)**

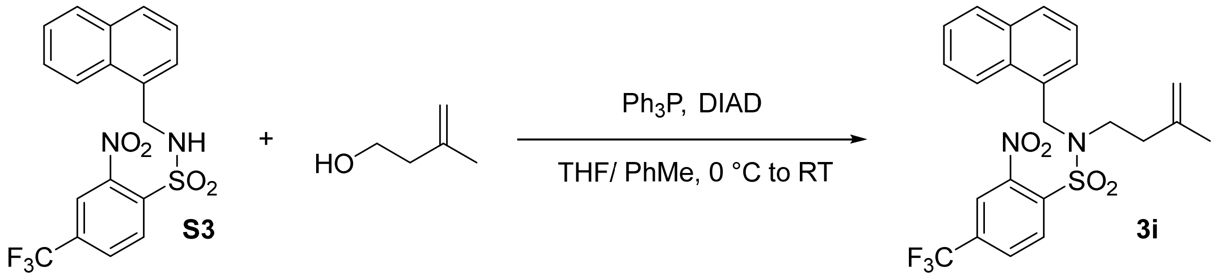

Following general procedure 2B, **S3** (400 mg, 0.97 mmol, 1 eq) was treated with 3-methylbut-3-en-1-ol (0.3 mL, 2.92 mmol, 3 eq), triphenyl phosphine (766 mg, 2.92 mmol, 3 eq) and DIAD (0.15 mL, 2.92 mmol, 3 eq) in a 1:1 mixture of THF and toluene (2.0 mL, 0.2 M). The crude product was purified by flash column chromatography on silica gel (eluent: 94:6 hexanes: EtOAc) to obtain the title compound **3i** (376 mg, 0.79 mmol, 81%) as a pale-yellow solid.

TLC: R_f = 0.41 (90:10 hexanes: EtOAc).

^{1}H NMR (500 MHz, $CDCl_3$): δ 8.05 (d, *J* = 7.8 Hz, 1H, ArH), 7.82 (t, *J* = 8.8 Hz, 2H, ArH), 7.77 (d, *J* = 10.5 Hz, 2H, ArH), 7.61 (d, *J* = 8.1 Hz, 1H, ArH), 7.52 – 7.43 (m, 3H, ArH), 7.39 (t, *J* = 7.6 Hz, 1H, ArH), 5.00 (s, 2H, $ArCH_2$), 4.71 (s, 1H, =C*H*), 4.58 (s, 1H, =C*H*), 3.56 – 3.49 (m, 2H, CH_2), 2.17 – 2.10 (m, 2H, CH_2), 1.59 (s, 3H, CH_3).

^{13}C {^{1}H, ^{19}F} NMR (126 MHz, $CDCl_3$): δ 147.6, 141.8, 137.0, 135.0, 133.8, 131.8, 131.6, 130.3, 129.5, 128.8, 128.10, 128.06, 126.9, 126.3, 125.2, 123.2, 122.1, 121.4, 112.7, 50.7, 48.2, 36.8, 22.3.

HRMS-ESI (m/z): $[M+Na]^+$ calculated for $[C_{23}H_{21}F_3N_2O_4SNa]^+$: 501.1072, found 501.1043.

***N*-(3-Methyl-3-(triethylsilylperoxy)butyl)-*N*-(naphthalen-1-ylmethyl)-2-nitro-4-(trifluoro-methyl)benzenesulfonamide (3j)**

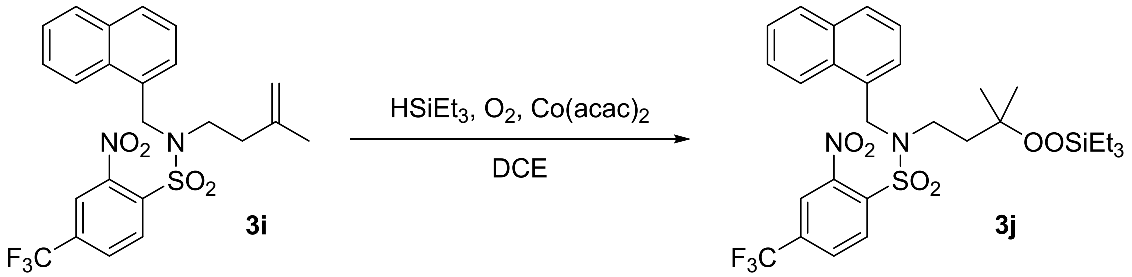

Following general procedure 6A, a stirred solution of **3i** (377 mg, 0.79 mmol, 1 eq) in DCE (4.0 mL, 0.2 M) was treated with triethylsilane (0.33 ml, 1.97 mmol, 2.5 eq), and Co(acac)$_2$ (21 mg, 0.08 mmol, 10 mol%) under an atmosphere of oxygen. The crude product was then purified by flash column chromatography on silica gel (eluent: 96:4 hexanes: EtOAc) to obtain the title compound **3j** (369 mg, 0.59 mmol, 75%) as a colorless oil.

TLC: R$_f$ = 0.27 (90:10 hexanes: acetone).

^{1}H NMR (500 MHz, CDCl$_3$): δ 8.04 (d, J = 8.0 Hz, 1H, ArH), 7.82 – 7.73 (m, 4H, ArH), 7.56 (d, J = 8.2 Hz, 1H, ArH), 7.47 (t, J = 8.1 Hz, 3H, ArH), 7.37 (t, J = 7.6 Hz, 1H, ArH), 5.01 (s, 2H, ArCH$_2$), 3.58 – 3.51 (m, 2H, CH$_2$), 1.83 – 1.76 (m, 2H, CH$_2$), 1.07 (s, 6H, (CH$_3$)$_2$), 0.88 (t, J = 7.9 Hz, 9H, SiCH$_2$CH_3), 0.56 (q, J = 8.0 Hz, 6H, SiCH_2CH$_3$).

^{13}C {^{1}H, ^{19}F} NMR (126 MHz, CDCl$_3$): δ 147.7, 137.3, 134.9, 133.8, 131.9, 131.7, 130.6, 129.3, 128.8, 127.94, 127.90, 126.8, 126.2, 125.2, 123.3, 122.1, 121.4, 81.0, 50.5, 45.3, 36.7, 24.5, 6.8, 3.9.

HRMS-ESI (m/z): [M+Na]$^+$ calculated for [$C_{29}H_{37}F_3N_2O_6SSiNa$]$^+$: 649.1991, found 649.1953.

***N*-(3-(*tert*-Butyldiphenylsilylperoxy)-3-methylbutyl)-*N*-(naphthalen-1-ylmethyl)-2-nitro-4-(trifluoromethyl)benzenesulfonamide (46)**

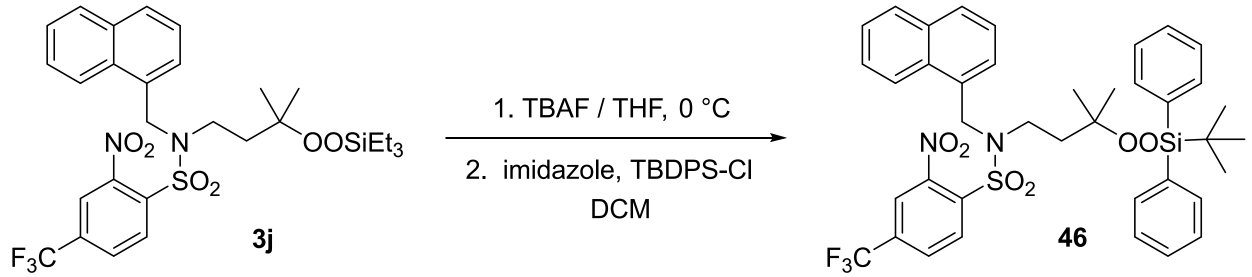

Following general procedure 7, TBAF (0.7 mL, 1.0 M in THF, 1.2 eq) added to **3j** (370 mg, 0.59 mmol, 1 eq) in THF (3.0 mL, 0.2 M) Upon completion, the residue in DCM (3.0 mL, 0.2 M) was treated with imidazole (60 mg, 0.89 mmol, 1.5 eq) and TBDPS-Cl (0.15 mL, 0.59 mmol, 1 eq). The crude product was purified by flash column chromatography on silica gel (eluent: 97:3 hexanes: EtOAc) to obtain the title compound **46** (364 mg, 0.51 mmol, 87% over 2 steps) as a colorless oil.

TLC: R_f = 0.24 (90:10 hexanes: acetone).

^{1}H NMR (500 MHz, C_6D_6): δ 8.13 (d, J = 8.5 Hz, 1H, ArH), 7.73 (d, J = 6.6 Hz, 4H, ArH), 7.50 (d, J = 8.1 Hz, 1H, ArH), 7.44 (d, J = 8.2 Hz, 1H, ArH), 7.36 (d, J = 8.2 Hz, 1H, ArH), 7.24 (t, J = 7.6 Hz, 1H, ArH), 7.22 – 7.19 (m, 2H, ArH), 7.18 (s, 4H, ArH), 7.15 (s, 2H, ArH), 7.07 (s, 1H, ArH), 7.00 (t, J = 7.6 Hz, 1H, ArH), 6.63 (d, J = 8.2 Hz, 1H, ArH), 4.85 (s, 2H, $ArCH_2$), 3.55 – 3.48 (m, 2H, CH_2), 1.75 – 1.68 (m, 2H, CH_2), 1.11 (s, 9H, *t*Bu), 0.85 (s, 6H, CH_3).

^{13}C {^{1}H, ^{19}F} NMR (126 MHz, C_6D_6): δ 148.0, 137.0, 136.2, 134.3, 134.1, 133.3, 132.1, 131.0, 130.2, 129.4, 128.9, 128.4, 128.1, 127.9, 127.6, 127.0, 126.4, 125.2, 123.8, 122.4, 121.0, 82.3, 51.0, 45.3, 37.6, 27.6, 24.2, 19.7.

HRMS-ESI (m/z): $[M+Na]^+$ calculated for $[C_{39}H_{41}F_3N_2O_6SSiNa]^+$: 773.3204, found 773.2303.

(3-(*tert*-Butyldiphenylsilylperoxy)-3-methyl-*N*-(naphthalen-1-ylmethyl)butan-1-amine (3k)

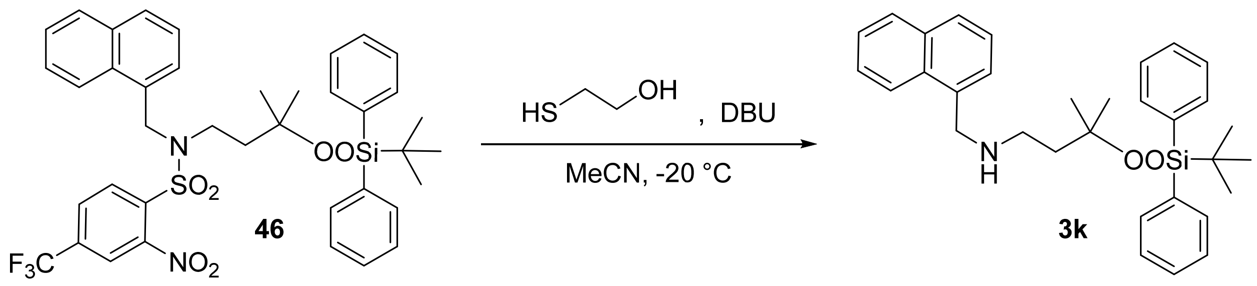

Following procedure 9D, a solution of **46** (215 mg, 0.29 mmol, 1 eq) in dry MeCN (2.0 mL, 0.2 M) was treated with a solution of 2-mercaptoethanol (0.03 mL, 0.29 mmol, 1 eq) and DBU (0.01 ml, 0.29 mmol, 1 eq) in MeCN (0.3 mL, 1 M). The crude residue was isolated after purification by flash column chromatography on silica gel (eluent: 75:25 hexanes: EtOAc) to obtain the final compound **3k** (134 mg, 0.27 mmol, 93%) as a colorless oil.

TLC: R_f = 0.15 (70:30 hexanes: EtOAc).

^{1}H NMR (500 MHz, CDCl$_3$): δ 8.19 (d, *J* = 8.4 Hz, 1H, ArH), 7.88 – 7.86 (m, 4H, ArH), 7.70 (d, *J* = 8.1 Hz, 1H, ArH), 7.62 (d, *J* = 8.2 Hz, 1H, ArH), 7.42 (d, *J* = 6.9 Hz, 1H, ArH), 7.36 (ddd, *J* = 8.3, 6.8, 1.3 Hz, 1H, ArH), 7.32 – 7.25 (m, 2H, ArH), 7.22 – 7.18 (m, 6H, ArH), 3.99 (s, 2H, ArCH$_2$), 2.64 – 2.57 (m, 2H, CH$_2$), 1.77 – 1.70 (m, 2H, CH$_2$), 1.23 (s, 9H, *t*Bu), 1.15 (s, 6H, (CH$_3$)$_2$), 0.89 (br, NH, 1H).

^{13}C NMR (126 MHz, CDCl$_3$): δ 136.7, 136.0, 135.0, 134.2, 133.44, 132.41, 129.8, 128.6, 127.6, 125.73, 125.71, 125.5, 125.4, 124.4, 83.1, 51.9, 45.3, 39.2, 27.4, 24.4, 19.5.

HRMS-ESI (m/z): $[M+Na]^+$ calculated for $[C_{33}H_{39}NO_2Si]^+$: 498.2823, found 498.2799.

Methylbut-3-en-1-yl 4-methylbenzenesulfonate (S11)

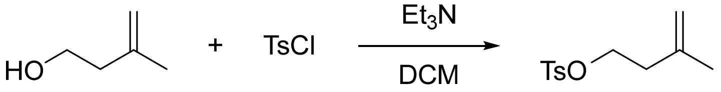

Following a literature procedure,[10] a solution of 3-methylbut-3-en-1-ol (1.17 mL, 11.61 mmol, 1 eq) in DCM (23 mL, 0.5 M) at 0 °C with stirring, was treated with TsCl (2.66 g, 13.93 mmol, 1.2 eq), and Et_3N (3.23 mL, 23.22 mmol, 2 eq). The mixture was then warmed to RT and stirred for 6 h. After such time, the solution was quenched with H_2O (50 mL) and extracted with DCM (3 x 50). The combined organic layers were dried over Na_2SO_4 and concentrated *in vacuo*. The crude product was purified by flash column chromatography on silica gel (eluent: 90:10 hexanes: EtOAc) to afford the title compound **S11** (2.4 g, 10.1 mmol, 87%) as a colorless oil with spectral data consistent with the literature.[11]

TLC: R_f = 0.46 (90:10 hexanes: EtOAc).

^{1}H NMR (500 MHz, $CDCl_3$): δ 7.79 (d, *J* = 7.9, 1.5 Hz, 2H, ArH), 7.34 (d, *J* = 7.9 Hz, 3H, ArH), 4.79 (s, 1H, =C*H*), 4.67 (s, 1H, =C*H*), 4.13 (t, *J* = 6.7, 1.0 Hz, 2H, CH_2), 2.45 (s, 3H, tosyl CH_3), 2.35 (t, *J* = 6.8 Hz, 2H, CH_2), 1.64 (s, 3H, CH_3).

^{13}C NMR (126 MHz, CDCl3): δ 144.9, 140.3, 133.3, 129.9, 128.0, 113.2, 68.7, 36.9, 22.4, 21.8.

HRMS-ESI (m/z): $[M+Na]^+$ calculated for $[C_{12}H_{16}O_3SNa]^+$: 263.0718, found 263.0705.

***N*-(3-Methylbut-3-en-1-yl)-2-nitro-*N*-phenyl-4-(trifluoromethyl)benzenesulfonamide (6g)**

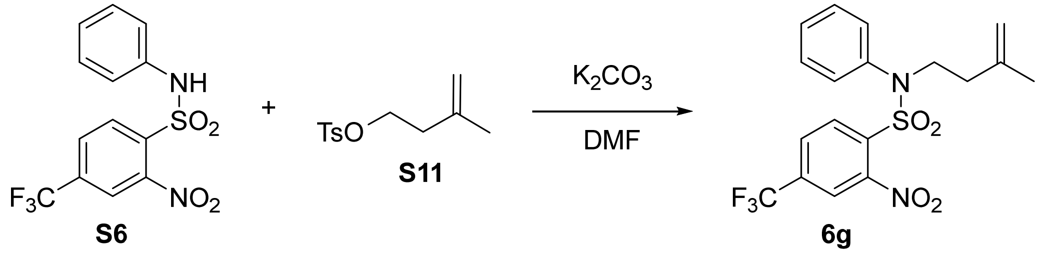

Following general procedure 2A, a stirred solution of **S6** (672 mg, 1.94 mmol, 1.0 eq) in DMF (10 mL, 0.2 M) was treated with K_2CO_3 (355 mg, 2.9 mmol, 1.5 eq), followed by **S11** (700 mg, 2.9 mmol, 1.5 eq). The crude residue was purified by flash column chromatography on silica gel (eluent: 95:5 hexanes: EtOAc) to obtain the title compound **6g** (700 mg, 1.7 mmol, 87%) as a yellow solid.

TLC: R_f = 0.57 (90:10 hexanes: EtOAc).

^{1}H NMR (500 MHz, $CDCl_3$): δ 7.85 (s, 1H, ArH), 7.69 (d, *J* = 8.3 Hz, 1H, ArH), 7.59 (d, *J* = 8.3 Hz, 1H, ArH), 7.38 – 7.33 (m, 3H, aniline ArH), 7.21 (dd, *J* = 6.7, 3.0 Hz, 2H, aniline ArH), 4.82 (s, 1H, =C*H*), 4.69 (s, 1H, =C*H*), 3.94 (t, *J* = 7.4 Hz, 2H, CH_2), 2.20 (t, *J* = 7.4 Hz, 2H, CH_2), 1.72 (s, 3H, CH_3).

^{13}C {^{1}H, ^{19}F} NMR (126 MHz, $CDCl_3$): δ 148.2, 141.6, 137.4, 135.7, 133.1, 131.7, 129.7, 129.0, 127.9, 123.2, 121.3, 121.0, 113.0, 50.8, 37.0, 22.3.

HRMS-ESI (m/z): $[M+Na]^+$ calculated for $[C_{18}H_{17}F_3N_2O_4SNa]^+$: 437.0759, found 437.0743.

***N*-(3-Methyl-3-(triethylsilylperoxy)butyl)-2-nitro-*N*-phenyl-4-(trifluoromethyl)-benzenesulfonamide (6h)**

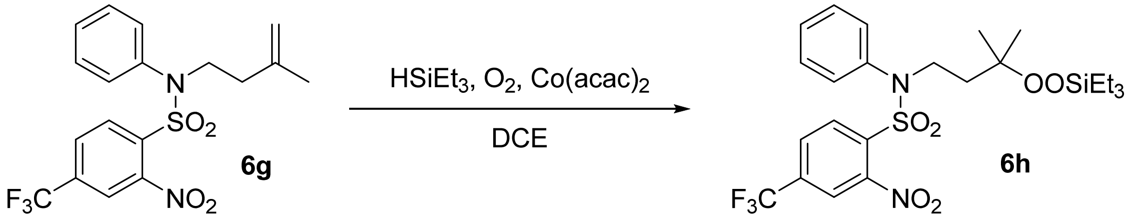

Following general procedure 6A, **6g** (361 mg, 0.87 mmol, 1 eq) in DCE (5.0 mL, 0.2 M) was treated with triethylsilane (0.35 mL, 2.17 mmol, 2.5 eq), and $Co(acac)_2$ (23 mg, 0.1 mmol, 10 mol%) under an atmosphere of oxygen. The crude product was then purified by flash column chromatography on silica gel (eluent: 95:5 hexanes: EtOAc) to obtain the title compound **6h** (417 mg, 0.74 mmol, 85%) as a colorless oil.

TLC: R_f = 0.48 (90:10 hexanes: EtOAc).

^{1}H NMR (500 MHz, $CDCl_3$): δ 7.83 (s, 1H, ArH), 7.69 (d, *J* = 8.3, 1.9 Hz, 1H, ArH), 7.62 (d, *J* = 8.3 Hz, 1H, ArH), 7.36 – 7.31 (m, 3H, aniline ArH), 7.23 – 7.16 (m, 2H, aniline ArH), 3.91 – 3.84 (m, 2H, CH_2), 1.84 – 1.77 (m, 2H, CH_2), 1.16 (s, 6H, $(CH_3)_2$), 0.89 (t, *J* = 8.0 Hz, 9H, $SiCH_2CH_3$), 0.58 (q, *J* = 8.1 Hz, 6H, $SiCH_2CH_3$).

^{13}C {^{1}H, ^{19}F} NMR (126 MHz, $CDCl_3$): δ 148.3, 137.8, 135.7, 135.3, 132.9, 129.6, 129.4, 128.8, 127.8, 121.3, 121.1, 81.0, 48.6, 37.6, 24.6, 6.8, 3.9.

HRMS-ESI (m/z): $[M+Na]^+$ calculated for $[C_{24}H_{33}F_3N_2O_6SSiNa]^+$: 585.1678, found 585.1664.

***N*-(3-(*tert*-Butyldiphenylsilylperoxy)-3-methylbutyl)-2-nitro-*N*-phenyl-4-(trifluoromethyl)-benzenesulfonamide (47)**

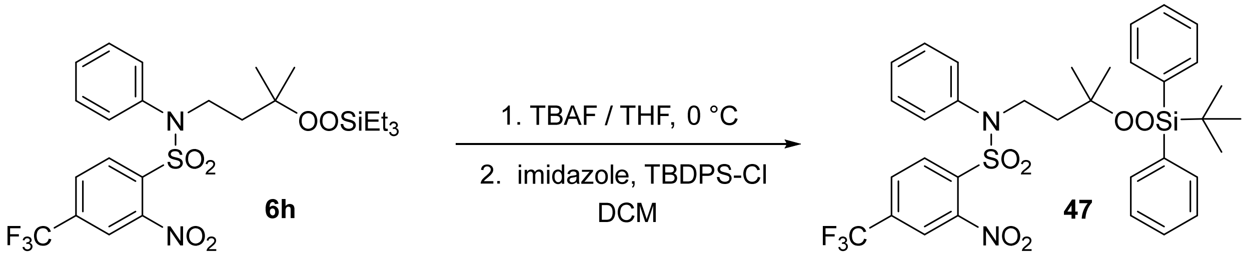

Following general procedure 7, TBAF (0.66 mL, 1.0 M in THF, 1.2 eq) was added to **6h** (307 mg, 0.55 mmol, 1 eq) in THF (3.0 mL, 0.2 M) Upon completion, the residue was dissolved in DCM (3 mL, 0.2 M) and treated with imidazole (56 mg, 0.82 mmol, 1.5 eq) and TBDPS-Cl (0.14 mL, 0.55 mmol, 1 eq). The crude product was purified by flash column chromatography on silica gel (eluent: 95:5 hexanes: EtOAc) to obtain the title compound **47** (303 mg, 0.49 mmol, 89% over 2 steps) as a colorless oil.

TLC: R*f* = 0.46 (90:10 hexanes: EtOAc).

^{1}H NMR (500 MHz, C_6D_6): δ 7.69 (d, *J* = 7.4, 1.9 Hz, 4H, ArH), 7.49 (d, *J* = 8.4 Hz, 2H, ArH), 7.26 (d, *J* = 8.4 Hz, 2H, ArH), 7.15 – 7.14 (m, 3H, ArH), 7.10 – 7.08 (m, 3H, ArH), 6.91 (d, *J* = 7.0 Hz, 3H, ArH), 6.84 (d, *J* = 7.7, 2.2 Hz, 2H, ArH), 3.65 – 3.59 (m, 2H, CH_2), 1.75 – 1.68 (m, 2H, CH_2), 1.09 (s, 9H, *t*Bu), 0.91 (s, 6H, $(CH_3)_2$).

^{13}C {^{1}H, ^{19}F} NMR (126 MHz, C_6D_6): δ 148.6, 137.2, 135.9, 135.7, 135.2, 134.6, 134.0, 133.8, 132.2, 129.9, 129.5, 129.1, 128.4, 127.3, 123.6, 81.9, 46.7, 37.3, 27.3, 24.2, 19.4.

HRMS-ESI (m/z): $[M+Na]^+$ calculated for $[C_{33}H_{38}F_3N_2O_6SSiNa]^+$: 709.1991, found 709.1980.

***N*-(3-(*tert*-Butyldiphenylsilylperoxy)-3-methylbutyl)-aniline (6i)**

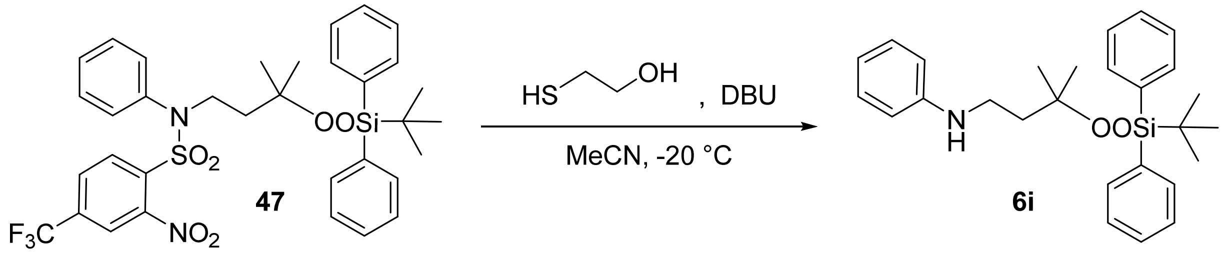

Following procedure 9D, a solution of **47** (333 mg, 0.49 mmol, 1 eq) in dry MeCN (3.0 mL, 0.2 M) was treated with a solution of 2-mercaptoethanol (0.4 mL, 0.49 mmol, 1 eq) and DBU (0.04 ml, 0.49 mmol, 1 eq) in MeCN (0.5 mL, 1 M). The crude residue was isolated after purification by flash column chromatography on silica gel (eluent: 97:3 hexanes: EtOAc) to obtain the final compound **6i** (193 mg, 0.45 mmol, 92%) as a colorless oil.

TLC: R*f* = 0.64 (90:10 hexanes: EtOAc).

^{1}H NMR (500 MHz, $CDCl_3$): δ 7.90 – 7.83 (m, 4H, ArH), 7.22 – 7.17 (m, 8H, ArH), 6.77 (t, J = 7.3 Hz, 1H, ArH), 6.44 (d, J = 7.9 Hz, 1H, ArH), 3.35 (br, 1H, NH), 2.92 (t, J = 7.3 Hz, 2H, CH_2), 1.60 (t, J = 7.3 Hz, 2H, CH_2), 1.24 (s, 9H, *t*Bu), 1.07 (s, 6H, $(CH_3)_2$).

^{13}C NMR (126 MHz, $CDCl_3$): δ 149.2, 136.6, 133.7, 130.5, 129.8, 128.3, 117.5, 113.3, 83.5, 39.7, 38.6, 28.0, 24.9, 20.0.

HRMS-ESI (m/z): $[M+Na]^+$ calculated for $[C_{28}H_{35}NO_2Si]^+$: 434.2523, found 434.2508.

2-(Cyclohex-1-en-yl)ethan-1-ol (S12)

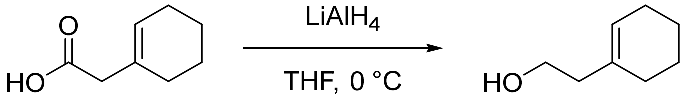

S12

Following a modified literature procedure,[11] a stirred solution of 2-(cyclohex-1-en-1-yl)acetic acid (1 g, 7.13 mmol, 1 eq) in dry THF (13 mL, 0.5 M) at 0 °C was treated with $LiAlH_4$ (410 mg, 10.7 mmol, 1.5 eq) portion wise over 10 min. The ice bath was then removed, and the mixture was warmed to RT and stirred for 3 h. After such time, the solution was cooled to 0 °C and quenched via dropwise addition of H_2O (50 mL). The solution was then acidified with 1M HCl until homogenous, after which the layers were separated, and the aqueous layer extracted with EtOAc. The combined organic layers were washed with brine, dried over Na_2SO_4, and concentrated *in vacuo*. The crude product was purified by flash column chromatography on silica gel (eluent: 88:12 hexanes: EtOAc) to afford title compound **S12** (780 mg, 6.2 mmol, 87%) as a colorless oil with spectral data consistent with the literature.[11]

TLC: R_f = 0.35 (80:20 hexanes: EtOAc; $KMnO_4$).

^{1}H NMR (500 MHz, $CDCl_3$): δ 5.51 (td, *J* = 3.7, 1.9 Hz, 1H, =C*H*), 3.65 (t, *J* = 6.3 Hz, 2H, CH_2), 2.20 (t, *J* = 6.3 Hz, 2H, CH_2), 2.00 (s, 2H, cyclohexane), 1.93 (s, 2H, cyclohexane), 1.63 (p, *J* = 5.9 Hz, 2H, cyclohexane), 1.59 – 1.53 (m, 2H, cyclohexane).

^{13}C NMR (126 MHz, CDCl3): δ 134.2, 124.4, 60.3, 41.3, 28.1, 25.4, 23.0, 22.5.

***N*-(2-(Cyclohex-1-en-1-yl)ethyl)-2-nitro-*N*-phenyl-4-(trifluoromethyl)benzenesulfonamide (12a)**

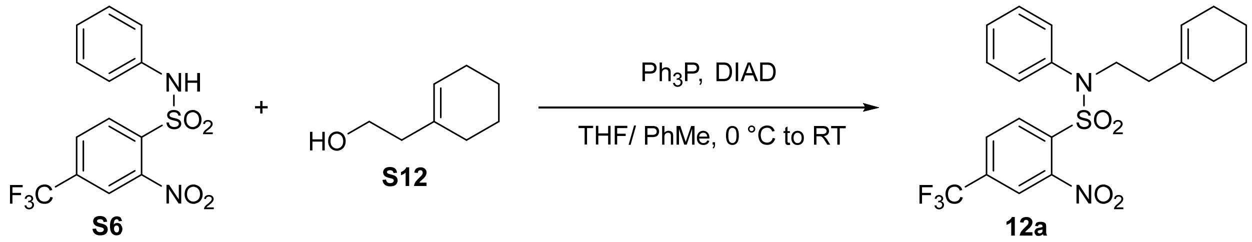

Following general procedure 2B, a solution of **S6** (225 mg, 0.65 mmol, 1 eq) in a 1:1 ratio of THF and toluene (3.0 mL, 0.2 M) was treated with alcohol **S12** (123 mg, 0.98 mmol, 1.5 eq), triphenyl phosphine (511 mg, 1.95 mmol, 3 eq) and DIAD (0.38 mL, 1.95 mmol, 3 eq). The crude residue was purified by flash column chromatography on silica gel (eluent: 95:5 hexanes: EtOAc) to obtain the title compound **12a** (270 mg, 0.59 mmol, 91%) as a yellow oil.

TLC: R_f = 0.37 (90:10 hexanes: acetone).

^{1}H NMR (500 MHz, $CDCl_3$): δ 7.84 (s, 1H, ArH), 7.69 (d, *J* = 8.3 Hz, 1H, ArH), 7.60 (d, *J* = 8.3 Hz, 1H, ArH), 7.38 – 7.29 (m, 3H, aniline ArH), 7.21 (dd, *J* = 7.7, 1.9 Hz, 2H, aniline ArH), 5.40 (td, *J* = 3.6, 1.9 Hz, 1H, =C*H*), 3.89 (t, *J* = 7.3 Hz, 2H, CH_2), 2.12 (t, *J* = 7.2 Hz, 2H, CH_2), 1.96 (s, 2H, cyclohexane), 1.90 – 1.84 (m, 2H, cyclohexane), 1.55 (pd, *J* = 5.3, 2.3 Hz, 2H, cyclohexane), 1.50 (pd, *J* = 6.0, 3.0 Hz, 2H, cyclohexane).

^{13}C NMR (126 MHz, $CDCl_3$): δ 148.1, 137.6, 135.2 (q, *J* = 34.7 Hz), 133.6, 133.0, 129.6, 128.7, 127.8 (q, *J* = 3.6 Hz), 124.3, 122.1 (q, *J* = 273.7 Hz), 121.2 (q, *J* = 3.6 Hz), 74.4, 51.0, 37.5, 28.0, 25.3, 22.8, 22.3, 21.6.

HRMS-ESI (m/z): $[M+Na]^+$ calculated for $[C_{21}H_{21}F_3N_2O_4SNa]^+$: 477.1072, found 477.1046.

2-Nitro-*N*-phenyl-*N*-(2-(1-(triethylsilylperoxy)cyclohexyl)ethyl)-4-(trifluoromethyl)-benzenesulfonamide (12b)

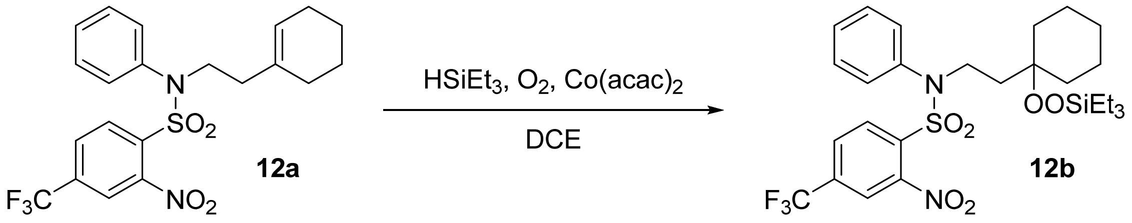

Following general procedure 6A, **12a** (430 mg, 0.95 mmol, 1 eq) in DCE (4.0 mL, 0.2 M) was treated with triethylsilane (0.37 mL, X mmol, 2.5 eq), and $Co(acac)_2$ (24 mg, 0.1 mmol, 10 mol%) under an atmosphere of oxygen. The crude product was then purified by flash column chromatography on silica gel (eluent: 97:3 hexanes: EtOAc) to obtain the title compound **12b** (295 mg, 0.49 mmol, 52%) as a white solid.

TLC: R_f = 0.51 (80:20 hexanes: EtOAc + 1% Et_3N).

^{1}H NMR (500 MHz, $CDCl_3$): δ 7.84 (s, 1H, ArH), 7.71 (d, *J* = 8.4 Hz, 1H, ArH), 7.64 (d, *J* = 8.3 Hz, 1H, ArH), 7.38 – 7.30 (m, 3H, aniline ArH), 7.23 – 7.18 (m, 2H, aniline ArH), 3.94 – 3.87 (m, 2H, CH_2), 1.86 – 1.79 (m, 2H, CH_2), 1.74 (d, *J* = 12.2 Hz, 2H, cyclohexane), 1.55 – 1.28 (m, 8H, cyclohexane), 0.89 (t, *J* = 8.0 Hz, 9H, $SiCH_2CH_3$), 0.58 (q, *J* = 7.9 Hz, 6H, $SiCH_2CH_3$).

^{13}C NMR (126 MHz, $CDCl_3$): δ 148.3, 137.9, 135.7 (q, *J* = 34.9 Hz), 135.3, 132.9, 129.5 (q, *J* = 3.6 Hz), 129.4, 128.7, 127.8 (q, *J* = 3.7 Hz), 123.3 (q, *J* = 273.6 Hz), 121.3, 81.9, 48.3, 35.9, 33.2, 25.8, 22.0, 6.8, 3.9.

HRMS-ESI (m/z): $[M+Na]^+$ calculated for $[C_{27}H_{37}F_3N_2O_6SSiNa]^+$: 625.1991, found 625.1954.

***N*-(2-(1-(*tert*-Butyldiphenylsilylperoxy)cyclohexyl)ethyl)-2-nitro-*N*-phenyl-4-(trifluoro-methyl)benzenesulfonamide (49)**

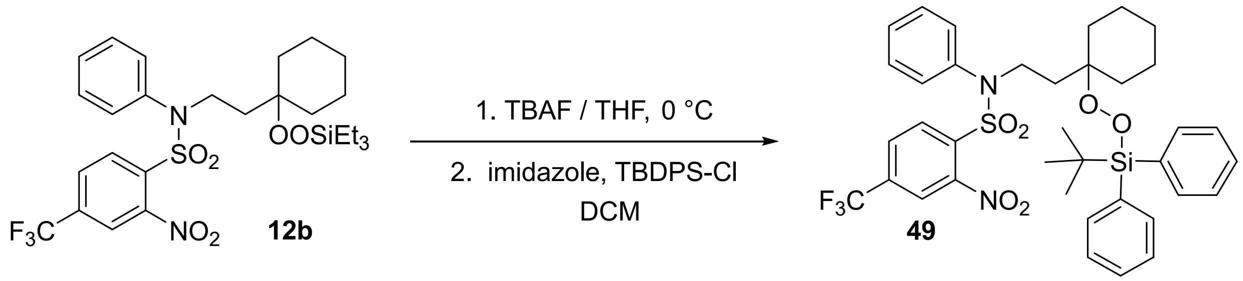

Following general procedure 7, TBAF (0.46 mL, 1.0 M in THF, 1.2 eq) was added to a solution of **12b** (230 mg, 0.38 mmol, 1 eq) in THF (2.0 mL, 0.2 M). Upon completion, the residue was dissolved in DCM (2.0 mL, 0.2 M) and treated with imidazole (39 mg, 0.57 mmol, 1.5 eq) and TBDPS-Cl (0.1 mL, 0.38 mmol, 1 eq). The crude product was purified by flash column chromatography on silica gel (eluent: 70:30 hexanes: DCM) to obtain the title compound **49** (190 mg, 0.26 mmol, 69% over 2 steps) as a colorless oil.

TLC: R*f* = 0.32 (90:10 hexanes: acetone).

^{1}H NMR (500 MHz, C_6D_6): δ 7.83 – 7.76 (m, 4H, ArH), 7.27 – 7.24 (m, 5H, ArH), 7.21 (s, 3H, ArH), 7.15 (s, 2H, ArH), 6.99 (d, *J* = 7.3 Hz, 3H, ArH), 6.63 (d, *J* = 8.1 Hz, 1H, ArH), 4.25 – 4.18 (m, 2H, CH_2), 2.09 – 2.02 (m, 2H, CH_2), 1.80 – 1.72 (m, 2H, cyclohexane), 1.35 – 1.24 (m, 4H, cyclohexane), 1.22 (s, 9H, *t*Bu), 1.19 (d, *J* = 11.3 Hz, 2H, cyclohexane), 1.09 – 0.87 (m, 2H, cyclohexane).

^{13}C NMR (126 MHz, C_6D_6): 148.4, 138.7, 136.5, 135.7, 134.7 (q, *J* = 34.4 Hz), 133.5, 133.0, 130.1, 129.7, 129.6, 128.5, 128.4, 127.3 (q, *J* = 3.3 Hz), 122.4 (q, *J* = 273.7 Hz), 120.9 (q, *J* = 3.6 Hz), 83.6, 48.7, 36.4, 33.2, 27.7, 25.8, 22.1, 19.8.

HRMS-ESI (m/z): $[M+Na]^+$ calculated for $[C_{37}H_{41}F_3N_2O_6SSiNa]^+$: 749.2304, found 749.2250.

***N*-(2-(1-((*tert*-Butyldiphenylsilyl)peroxy)cyclohexyl)ethyl)aniline (12c)**

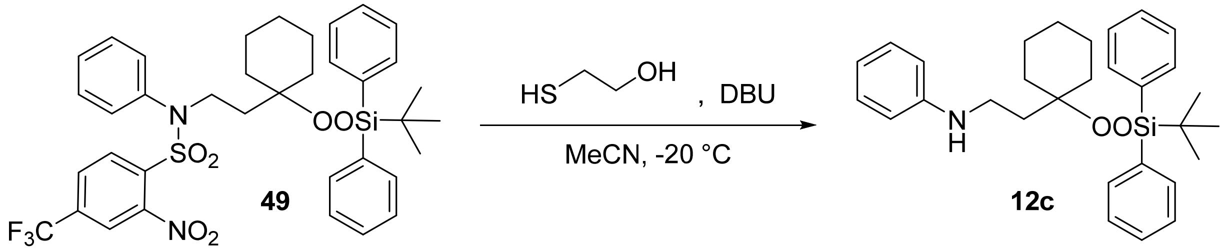

Following procedure 9D, a solution of **49** (140 mg, 0.19 mmol, 1 eq) in dry MeCN (3.0 mL, 0.2 M) was treated with a solution of 2-mercaptoethanol (0.16 mL, 0.19 mmol, 1 eq) and DBU (0.01 ml, 0.19 mmol, 1 eq) in MeCN (0.2 mL, 1 M). The crude residue was isolated after purification by flash column chromatography on silica gel (eluent: 97:3 hexanes: EtOAc) to obtain the final compound **12c** (93 mg, 0.19 mmol, 98%) as a colorless oil.

TLC: R_f = 0.60 (90:10 hexanes: acetone).

^{1}H NMR (500 MHz, CDCl$_3$): δ 7.90 – 7.82 (m, 4H, ArH), 7.23 – 7.18 (m, 8H, ArH), 6.78 (t, *J* = 7.3 Hz, 1H, ArH), 6.48 (d, *J* = 7.9 Hz, 2H, ArH), 3.25 (br, 1H, NH), 2.94 (t, *J* = 7.2 Hz, 2H, CH_2), 1.79 (dd, *J* = 12.4, 6.3 Hz, 2H, cyclohexane), 1.73 (t, *J* = 7.8 Hz, 2H, CH_2), 1.43 (dd, *J* = 9.2, 3.7 Hz, 2H, cyclohexane), 1.32 – 1.27 (m, 1H, cyclohexane), 1.26 (s, 9H, *t*Bu), 1.23 – 1.07 (m, 5H, cyclohexane).

^{13}C NMR (126 MHz, CDCl$_3$): δ 149.1, 136.5, 133.5, 130.3, 129.5, 128.0, 117.2, 113.1, 84.2, 38.8, 35.8, 33.3, 27.8, 26.0, 22.5, 19.9.

HRMS-ESI (m/z): $[M+Na]^+$ calculated for $[C_{31}H_{39}NO_2Si]^+$: 474.2823, found 474.2796.

1-(4-Chlorophenyl)but-3-en-1-ol (S13)

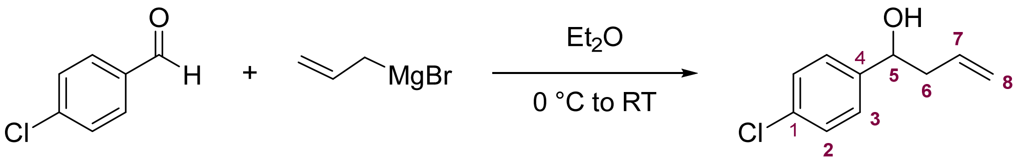

Following a modified literature procedure,[12] a stirred solution of 4-chlorobenzaldehyde (1.0 g, 7.11 mmol, 1 eq) in Et_2O (17 mL, 0.4 M) at 0 °C was treated with allylmagnesium bromide 2 M in THF (4.3 mL, 8.54 mmol, 1.2 eq) dropwise over 5 min. The mixture was warmed to RT and stirred for 1 h. After such time, the solution was quenched with 1 M HCl (10 mL) and H_2O (100 mL) and the aqueous layer extracted with EtOAc (3x, 100 mL). The combined organic layers were dried over Na_2SO_4 and concentrated *in vacuo*. The crude product was purified by flash column chromatography on silica gel (eluent: 94:6 hexanes: EtOAc) to afford the title compound **S13** (1.11 g, 6.04 mmol, 85%) as a colorless oil.

TLC: R_f = 0.31 (90:10 hexanes: acetone).

^{1}H NMR (500 MHz, CDCl$_3$): δ 7.28 (d, J = 8.2 Hz, 2H, ArH^3), 7.22 (d, J = 8.4 Hz, 2H, ArH^2), 5.73 (ddt, J = 14.2, 9.8, 7.1 Hz, 1H, C^7H=CH$_2$), 5.12 (s, 2H, =C^8H), 5.10 (d, J = 13.2 Hz, 1H, =C^8H), 4.63 (t, J = 6.0 Hz, 1H, C^{5}H), 2.62 – 2.57 (s, 1H, OH), 2.42 (h, J = 7.5, 6.6 Hz, 2H, C^{6}H$_2$).

^{13}C NMR (126 MHz, CDCl3): δ 142.4 (C^1), 134.0 (C^7), 133.1 (C^4), 128.5 (C^2), 127.3 (C^3), 118.7 (C^8), 72.7 (C^5), 43.8 (C^6).

HRMS-ESI (m/z): $[M-H_2O]^+$ calculated for $[C_{10}H_{10}Cl]^+$: 165.0437, found 165.0463.

***N*-(1-(4-Chlorophenyl)but-3-en-1-yl)-*N*-(naphthalen-1-ylmethyl)-2-nitro-4-(trifluoro-methyl)benzenesulfonamide (13a)**

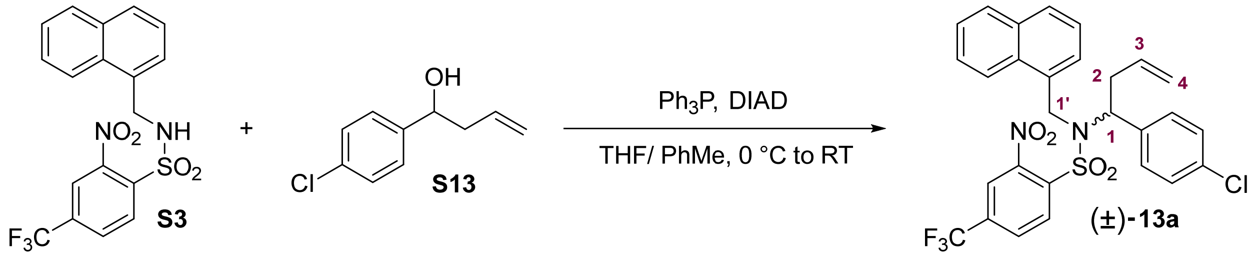

Following procedure 2B, a solution of **S3** (200 mg, 0.487 mmol, 1 eq) in a 1:1 ratio of THF and toluene (3.0 mL, 0.2 M) was treated with alcohol **S13** (267 mg, 1.46 mmol, 3 eq), triphenyl phosphine (383 mg, 1.46 mmol, 3 eq) and DIAD (0.08 mL, 1.46 mmol, 3 eq). The crude residue was purified by flash column chromatography on silica gel (eluent: 95:5 hexanes: EtOAc) to obtain the title compound **13a** (293 mg, 0.41 mmol, 84%) as a pale-yellow solid.

TLC: R_f = 0.25 (90:10 hexanes: EtOAc).

^{1}H NMR (500 MHz, $CDCl_3$): δ 7.62 (d, J = 8.4 Hz, 3H, ArH), 7.58 (d, J = 8.2 Hz, 1H, ArH), 7.35 (q, J = 8.4 Hz, 4H, ArH), 7.32 (d, J = 8.9 Hz, 2H, ArH), 7.24 (s, 1H, ArH), 7.21 (d, J = 6.2 Hz, 3H, ArH), 5.59 (ddt, J = 16.9, 10.3, 6.8 Hz, 1H, C^3H=CH_2), 5.42 (dd, J = 10.5, 5.0 Hz, 1H, C^1H), 5.07 (d, J = 17.1 Hz, 1H, =C^4H), 4.97 (d, J = 10.3 Hz, 1H, =C^4H), 4.91 (d, J = 14.7 Hz, 1H, $C^{1'}$H), 4.69 (d, J = 14.7 Hz, 1H, $C^{1'}$H), 3.01 (td, J = 12.3, 6.9 Hz, 1H, C^2H), 2.91 (td, J = 12.8, 6.6 Hz, 1H, C^2H).

^{13}C {^{1}H, ^{19}F} NMR (126 MHz, $CDCl_3$): δ 146.9, 137.4, 135.1, 134.6, 134.5, 133.47, 133.37, 132.1, 131.5, 130.7, 130.4, 129.3, 129.1, 128.8, 128.7, 127.7, 126.7, 126.0, 125.1, 122.9, 122.0, 121.3, 118.8, 61.2, 47.4, 36.0.

HRMS-ESI (m/z): $[M+Na]^+$ calculated for $[C_{28}H_{22}ClF_3N_2O_4SNa]^+$: 597.0839, found 597.0836.

anti **and** ***syn N*-(1-(4-Chlorophenyl)-3-(triethylsilylperoxy)butyl)-*N*-(naphthalene-1-ylmethyl)-2-nitro-4-(trifluoromethyl)benzenesulfonamide (13b** and **13c)**

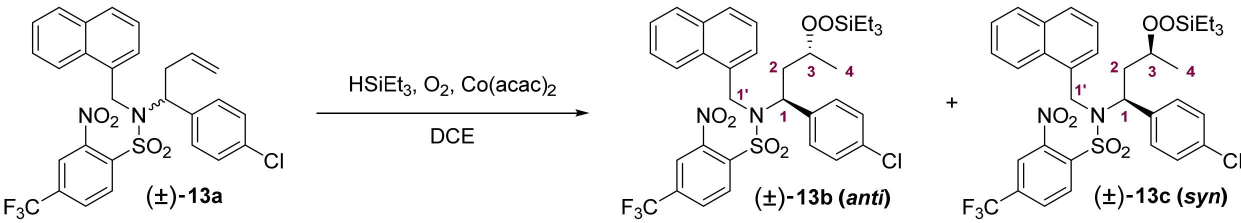

Following general procedure 6A, **13a** (290 mg, 0.504 mmol, 1 eq) in DCE (3.0 mL, 0.2 M) was treated with triethylsilane (0.2 mL, 1.26 mmol, 2.5 eq), and Co(acac)$_2$ (13 mg, 0.05 mmol, 10 mol%) under an atmosphere of oxygen. The crude residue was purified by flash column chromatography on silica gel (eluent: 95:5 hexanes: EtOAc) to obtain the title compounds **13b** (100 mg, 0.14 mmol, 28%) and **13c** (92 mg, 0.13 mmol, 26%) as colorless oils.

<u>Data for *anti*-isomer 13b:</u>

TLC: R$_f$ = 0.42 (90:10 hexanes: acetone).

^{1}H NMR (500 MHz, C$_6$D$_6$): δ 7.60 (dd, *J* = 6.3, 3.5 Hz, 1H, ArH), 7.39 (d, *J* = 8.4 Hz, 2H, ArH), 7.33 (dd, *J* = 6.3, 3.3 Hz, 1H, ArH), 7.22 (d, *J* = 8.3 Hz, 1H, ArH), 7.18 (s, 1H, ArH), 7.08 (s, 1H, ArH), 7.07 – 7.04 (m, 3H, ArH), 6.99 (s, 1H, ArH), 6.83 (d, *J* = 8.2 Hz, 2H, ArH), 6.37 (d, *J* = 8.2 Hz, 1H, ArH), 5.62 (dd, *J* = 11.9, 3.9 Hz, 1H, C^{1}H), 4.65 (d, *J* = 14.7 Hz, 1H, C$^{1'}$H), 4.53 (d, *J* = 14.7 Hz, 1H, C$^{1'}$H), 3.98 (q, *J* = 8.1 Hz, 1H, C^{3}H), 3.06 (td, *J* = 12.5, 4.4 Hz, 1H, C^{2}H), 2.27 (ddd, *J* = 12.8, 8.2, 4.0 Hz, 1H, C^{2}H), 1.25 (d, *J* = 6.2 Hz, 3H, C^{4}H$_3$), 0.98 (t, *J* = 7.8 Hz, 9H, SiCH$_2$C*H*$_3$), 0.64 (q, *J* = 7.9 Hz, 6H, SiC*H*$_2$CH$_3$).

^{13}C {^{1}H, ^{19}F} NMR (126 MHz, C$_6$D$_6$): δ 147.1, 137.2, 135.3, 135.2, 134.1, 133.7, 133.3, 131.9, 131.7, 130.8, 130.6, 129.1, 128.7, 128.6, 128.4, 127.2, 126.9, 126.4, 125.8, 124.8, 122.9, 122.0, 120.7, 78.9, 58.9, 17.7, 6.7, 3.8.

HRMS-ESI (m/z): $[M+Na]^+$ calculated for $[C_{34}H_{38}ClF_3N_2O_6SSiNa]^+$: 745.1758, found 745.1744.

Data for *syn*-isomer 13c:

TLC: R_f = 0.39 (90:10 hexanes: acetone).

^{1}H NMR (500 MHz, C_6D_6): δ 7.57 (d, *J* = 6.9 Hz, 1H, ArH), 7.36 (d, *J* = 7.1 Hz, 1H, ArH), 7.26 (dd, *J* = 18.0, 7.6 Hz, 2H, ArH), 7.14 (s, 1H, ArH), 7.08 (t, *J* = 3.3 Hz, 2H, ArH), 7.04 – 7.00 (m, 3H, ArH), 6.99 (s, 1H, ArH), 6.90 (t, *J* = 7.7 Hz, 1H, ArH), 6.47 (d, *J* = 6.4 Hz, 1H, ArH), 5.92 (dd, *J* = 11.6, 4.3 Hz, 1H, C^1H), 4.73 (d, *J* = 15.1 Hz, 1H, $C^{1'}H$), 4.61 (d, *J* = 15.1 Hz, 1H, $C^{1'}H$), 3.91 (ddp, *J* = 9.2, 5.8, 3.1 Hz, 1H, C^3H), 2.54 (ddd, *J* = 13.7, 9.0, 4.3 Hz, 1H, C^2H), 2.14 (ddd, *J* = 14.7, 11.5, 3.4 Hz, 1H, C^2H), 1.15 (d, *J* = 6.1 Hz, 3H, C^4H_3), 1.06 (t, *J* = 8.0 Hz, 9H, $SiCH_2CH_3$), 0.82 – 0.67 (q, *J* = 7.9 Hz, 6H, $SiCH_2CH_3$).

^{13}C {^{1}H, ^{19}F} NMR (126 MHz, C_6D_6): δ 147.2, 137.4, 135.9, 134.6, 133.9, 133.6, 132.0, 131.8, 131.1, 131.0, 129.0, 128.99, 128.92, 128.8, 127.1, 126.7, 126.1, 125.2, 123.1, 122.3, 120.9, 78.3, 58.7, 47.2, 37.1, 19.3, 7.1, 4.2.

HRMS-ESI (m/z): $[M+Na]^+$ calculated for $[C_{34}H_{38}ClF_3N_2O_6SSiNa]^+$: 745.1758, found 745.1744.

***anti-N*-(3-(*tert*-Butyldiphenylsilylperoxy)butyl)-1-(4-chlorophenyl)butyl)-*N*-(naphthalen-1-ylmethyl-2-nitro-4-(trifluoromethyl)benzenesulfonamide (51)**

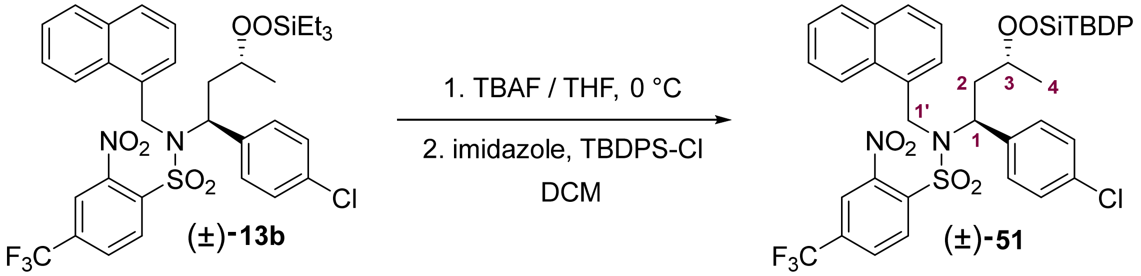

Following procedure 7, TBAF (0.16 mL, 1.0 M in THF, 1.2 eq) was added to **13b** (100 mg, 0.139 mmol, 1 eq) in THF (1.0 mL, 0.2 M) Upon completion, the residue was dissolved in DCM (1.0 mL, 0.2 M) and treated with imidazole (14 mg, 0.21 mmol, 1.5 eq) and TBDPS-Cl (0.04 mL, 0.139 mmol, 1 eq). The crude product was purified by flash column chromatography on silica gel (eluent: 97:3 hexanes: EtOAc) to obtain the title compound **51** (83 mg, 0.1 mmol, 71% over 2 steps) as a colorless oil.

TLC: R_f = 0.20 (90:10 hexanes: acetone).

^{1}H NMR (500 MHz, C_6D_6): δ 7.76 (t, *J* = 6.4 Hz, 4H, ArH), 7.73 – 7.70 (m, 1H, ArH), 7.61 – 7.55 (m, 1H, ArH), 7.38 – 7.30 (m, 1H, ArH), 7.25 – 7.18 (m, 8H, ArH), 7.13 (s, 1H, ArH), 7.08 – 7.04 (m, 2H, ArH), 7.03 – 6.97 (m, 3H, ArH), 6.87 – 6.80 (m, 2H, ArH), 6.37 (d, *J* = 10.2 Hz, 1H, ArH), 5.48 (dd, *J* = 11.8, 3.8 Hz, 1H, C^1H), 4.62 (d, *J* = 14.8 Hz, 1H, $C^{1'}H$), 4.51 (d, *J* = 14.7 Hz, 1H, $C^{1'}H$), 3.89 (ddd, *J* = 10.2, 6.2, 3.9 Hz, 1H, C^3H), 2.91 (td, *J* = 12.6, 4.1 Hz, 1H, C^2H), 2.19 (ddd, *J* = 13.0, 8.7, 3.8 Hz, 1H, C^2H), 1.17 (s, 3H, C^4H_3), 1.16 (s, 9H, *t*Bu).

^{13}C {^{1}H, ^{19}F} NMR (126 MHz, C_6D_6): δ 146.6, 137.1, 135.8, 135.6, 135.1, 135.0, 134.3, 133.5, 133.3, 132.7, 131.8, 131.6, 130.6, 130.0, 129.5, 129.1, 128.7, 128.4, 126.9, 126.4, 125.7, 124.8, 122.9, 122.0, 120.7, 79.3, 58.6, 46.9, 36.1, 27.2, 26.4, 19.3, 17.6.

HRMS-ESI (m/z): $[M+Na]^+$ calculated for $[C_{44}H_{42}ClF_3N_2O_6SSiNa]^+$: 869.2071, found 869.2056.

***syn-N*-(3-(*tert*-Butyldiphenylsilylperoxy)butyl)-1-(4-chlorophenyl)butyl)-*N*-(naphthalen-1-ylmethyl-2-nitro-4-(trifluoromethyl)benzenesulfonamide (52)**

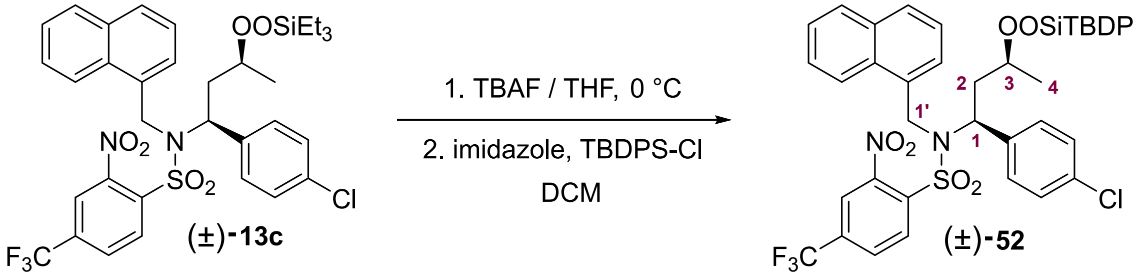

Following procedure 7, TBAF (0.15 mL, 1.0 M in THF, 1.2 eq) was added to **13c** (92 mg, 0.128 mmol, 1 eq) in THF (1.0 mL, 0.2 M) Upon completion, the residue was dissolved in DCM (1.0 mL, 0.2 M) and treated with imidazole (12 mg, 0.20 mmol, 1.5 eq) and TBDPS-Cl (0.03 mL, 0.128 mmol, 1 eq). The crude product was purified by flash column chromatography on silica gel (eluent: 96:4 hexanes: EtOAc) to obtain the title compound **52** (93 mg, 0.11 mmol, 91% over 2 steps) as a colorless oil.

TLC: R_f = 0.19 (90:10 hexanes: acetone).

^{1}H NMR (500 MHz, C_6D_6): δ 7.83 – 7.77 (m, 2H, ArH), 7.72 – 7.69 (m, 2H, ArH), 7.61 – 7.55 (m, 1H, ArH), 7.33 (dd, J = 6.4, 3.8 Hz, 1H, ArH), 7.23 – 7.13 (m, 9H, ArH), 6.93 (s, 1H, ArH), 6.84 (d, J = 7.7 Hz, 2H, ArH), 6.81 (s, 2H, ArH), 6.35 (d, J = 8.4 Hz, 1H, ArH), 5.66 (dd, J = 11.6, 4.0 Hz, 1H, C^1H), 4.79 (d, J = 15.3 Hz, 1H, $C^{1'}H$), 4.59 (d, J = 15.5 Hz, 1H, $C^{1'}H$), 3.93 – 3.82 (m, 1H, C^3H), 2.27 (ddd, J = 13.3, 9.1, 4.0 Hz, 1H, C^2H), 2.03 – 1.94 (m, 1H, C^2H), 1.12 (s, 9H, *t*Bu), 0.89 (d, J = 6.3 Hz, 3H, C^4H_3).

^{13}C {^{1}H, ^{19}F} NMR (126 MHz, C_6D_6): δ 147.6, 135.9, 135.7, 135.0, 134.0, 133.4, 132.7, 132.1, 131.3, 131.0, 130.6, 130.3, 130.1, 129.5, 129.1, 128.5, 128.4, 127.1, 126.3, 125.7, 124.9, 122.8, 122.1, 120.6, 78.6, 58.1, 47.0, 36.9, 27.2, 26.4, 19.3, 18.7.

HRMS-ESI (m/z): $[M+Na]^+$ calculated for $[C_{44}H_{42}ClF_3N_2O_6SSiNa]^+$: 869.2071, found 869.2056.

2-Nitro-*N*-(2-(prop-1-en-2-yl)phenyl)-4-(trifluoromethyl)benzenesulfonamide (S7)

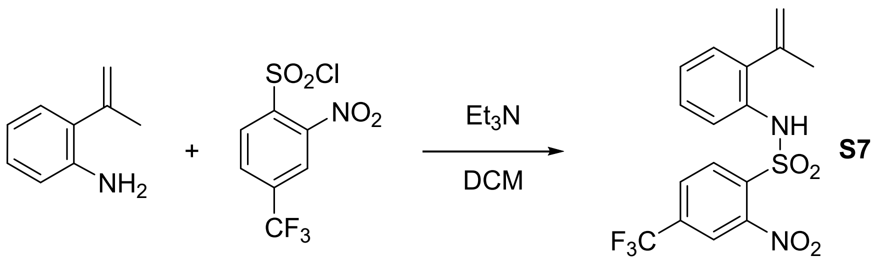

2-(Prop-1-en-2-y)aniline (0.45 mL, 1.02 mmol, 1.5 eq.) in dry DCM (5.0 mL, 0.2 M) was protected following procedure 1B by treatment with 2-nitro-4-(trifluoromethyl)benzenesulfonyl chloride (197 mg, 0.68 mmol, 1 eq.) and triethylamine (0.1 mL, 0.68 mmol, 1 eq). The crude residue was purified by flash column chromatography on silica gel (eluent: 95:5 hexanes: EtOAc) to obtain the title compound **S7** (218 mg, 0.56 mmol, 83%) as a yellow oil.

TLC: R_f = 0.15 (90:10 hexanes: acetone).

^{1}H NMR (500 MHz, $CDCl_3$): δ 8.08 (d, *J* = 8.0 Hz, 1H, ArH), 7.87 (d, *J* = 8.4 Hz, 2H, ArH), 7.63 (d, *J* = 8.2 Hz, 1H, ArH), 7.29 – 7.22 (m, 1H, aniline ArH), 7.14 (t, *J* = 7.5 Hz, 1H, aniline ArH), 7.08 (d, *J* = 7.4 Hz, 1H, aniline ArH), 5.29 (s, 1H, =C*H*), 4.77 (s, 1H, =C*H*), 1.76 (s, 3H, CH_3).

^{13}C NMR (126 MHz, $CDCl_3$): δ 148.3, 141.7, 136.6, 136.5 (q, *J* = 34.9 Hz), 135.9, 132.4, 131.7, 129.5 (q, *J* = 3.6 Hz), 128.7, 128.3, 125.9, 122.7 (q, *J* = 3.8 Hz), 122.1, 122.0 (q, *J* = 273.7 Hz), 117.8, 24.6.

HRMS-ESI (m/z): $[M+Na]^+$ calculated for $[C_{16}H_{13}F_3N_2O_4SNa]^+$: 409.0446, found 409.0430.

2-Nitro-*N*-(2-(prop-1-en-2-yl)phenyl)-4-(trifluoromethyl)-*N*-(3-(trifluoromethyl)benzyl)-benzenesulfonamide (7a)

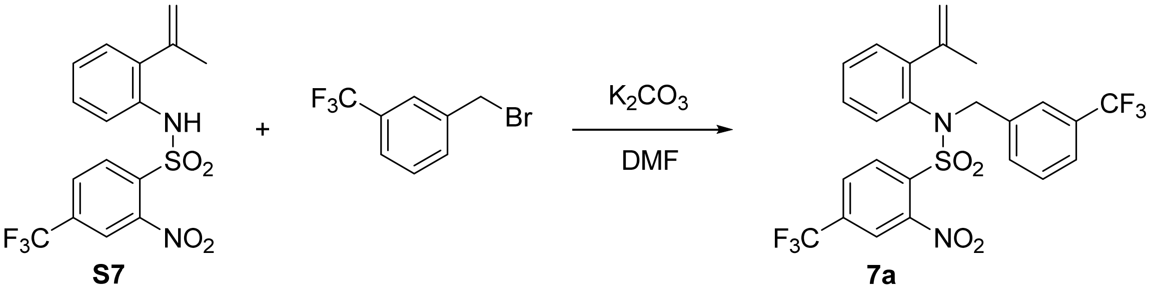

Following procedure 2A, a stirred solution of **S7** (280 mg, 0.73 mmol, 1.0 eq) in DMF (4.0 mL, 0.2 M) was treated with K_2CO_3 (150 mg, 1.1 mmol, 1.5 eq), followed by 1-(bromomethyl)-3-(trifluoromethyl)benzene (0.17 mL, 1.1 mmol, 1.5 eq). The crude residue was purified by flash column chromatography on silica gel (eluent: 95:5 hexanes: EtOAc) to obtain the title compound **7a** (388 mg, 0.71 mmol, 98%) as a yellow oil.

TLC: R_f = 0.61 (50:50 hexanes: DCM).

^{1}H NMR (500 MHz, $CDCl_3$): δ 7.90 (s, 1H, ArH), 7.80 (s, 2H, ArH), 7.52 (d, *J* = 5.4, 3.6 Hz, 1H, ArH), 7.38 (d, *J* = 4.7, 2.6 Hz, 2H, ArH), 7.33 (s, 1H, ArH), 7.30 (t, *J* = 7.5, 1.3 Hz, 1H, ArH), 7.20 (d, *J* = 7.7, 1.4 Hz, 1H, ArH), 7.14 (t, *J* = 8.8, 7.6, 1.4 Hz, 1H, ArH), 6.85 (d, *J* = 8.1, 1.5 Hz, 1H, ArH), 5.09 (br, 1H, ArCH), 4.93 (s, 1H, =C*H*), 4.74 (br, 1H, ArCH), 4.44 (s, 1H, =C*H*), 1.85 (s, 3H, CH_3).

^{13}C NMR (126 MHz, $CDCl_3$): δ 148.4, 144.3, 142.7, 136.8 (q, *J* = 34.8 Hz), 136.4, 135.8 (q, *J* = 3.1 Hz), 134.2, 133.2, 133.1, 131.6, 130.9 (q, *J* = 3.6 Hz), 130.8, 129.4 (q, *J* = 3.7 Hz), 129.2, 128.3, 127.9, 126.7, 125.2 (q, *J* = 4.4 Hz), 125.0 (q, *J* = 3.8 Hz), 122.9 (q, *J* = 3.9 Hz), 121.5 (q, *J* = 273.9 Hz), 117.0, 56.3, 24.1.

HRMS-ESI (m/z): $[M+Na]^+$ calculated for $[C_{24}H_{18}F_6N_2O_4SNa]^+$: 567.0789, found 567.0784.

2-Nitro-*N*-(2-(2-(triethylsilylperoxy)propan-2-yl)phenyl)-4-(trifluoromethyl)-*N*-(3-(trifluoromethyl)benzyl)benzenesulfonamide (55)

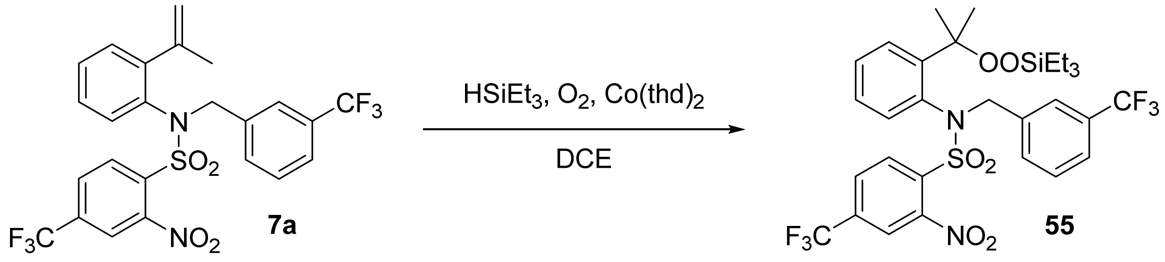

Following general procedure 6B, **7a** (380 mg, 0.67 mmol, 1 eq) in DCE (4.0 mL, 0.2 M) was treated with triethylsilane (0.27 mL, 1.74 mmol, 2.5 eq), and $Co(thd)_2$ (30 mg, 0.07 mmol, 10 mol%) under an atmosphere of oxygen. The crude residue was purified by flash column chromatography on silica gel (eluent: 95:5 hexanes: EtOAc) to obtain the title compound **55** (382 mg, 0.55 mmol, 82%) as a white solid.

TLC: R_f = 0.51 (90:10 hexanes: EtOAc).

^{1}H NMR (500 MHz, $CDCl_3$): δ 7.80 (d, *J* = 8.1 Hz, 1H, ArH), 7.27 (s, 1H, ArH), 7.19 (d, *J* = 9.4 Hz, 2H, ArH), 7.12 – 7.02 (m, 3H, ArH), 6.80 – 6.71 (m, 2H, ArH), 6.64 (t, *J* = 7.6 Hz, 1H, ArH), 6.14 (d, *J* = 8.0 Hz, 1H, ArH), 5.01 (d, *J* = 14.2 Hz, 1H, ArCH), 4.88 (d, *J* = 14.2 Hz, 1H, ArCH), 1.68 (s, 3H, dimethyl CH_3), 1.60 (s, 3H, dimethyl CH_3), 0.97 (t, *J* = 8.0 Hz, 9H, $SiCH_2CH_3$), 0.65 (q, *J* = 8.0 Hz, 6H, $SiCH_2CH_3$).

^{13}C NMR (126 MHz, $CDCl_3$): δ 148.7, 146.2, 135.5, 135.5 (q, *J* = 35.1 Hz), 135.2, 134.9, 134.3 (q, *J* = 3.1 Hz), 133.2, 131.4, 131.2, 130.3 (q, *J* = 3.6 Hz), 128.9, 128.7, 127.1, 125.0 (q, *J* = 271.8 Hz), 123.1 (q, *J* = 4.4 Hz), 122.7 (q, *J* = 3.8 Hz), 121.0 (q, *J* = 34.7 Hz), 120.7 (q, *J* = 273.7 Hz), 84.3, 56.1, 28.4, 25.8, 6.7, 3.8.

HRMS-ESI (m/z): $[M+Na]^+$ calculated for $[C_{30}H_{34}F_6N_2O_6SSiNa]^+$: 715.1709, found 715.1695.

***N*-(4-Methoxybenzyl)-2-nitro-*N*-(2-(prop-1-en-2-yl)phenyl)-4-(trifluoromethyl)-benzenesulfonamide (7b)**

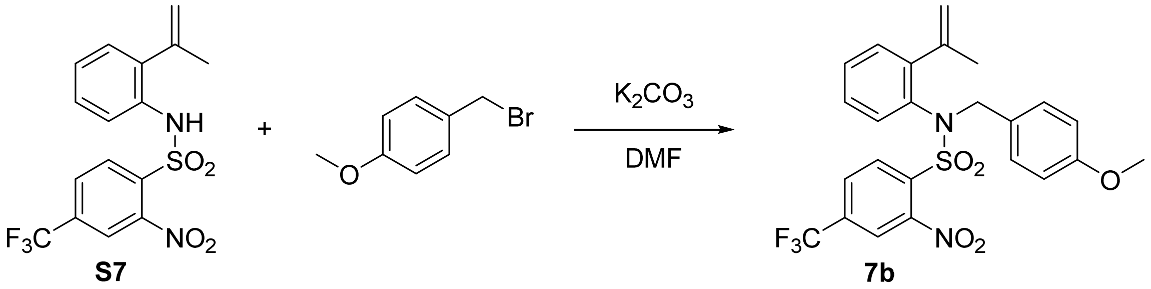

Following procedure 2A, a stirred solution of **S7** (244 mg, 0.63 mmol, 1.0 eq) in DMF (4.0 mL, 0.2 M) was treated with K_2CO_3 (174 mg 1.3 mmol, 2 eq), followed by 1-(bromomethyl)-4-(methoxy)benzene (0.2 mL, 1.3 mmol, 2 eq). The crude residue was purified by flash column chromatography on silica gel (eluent: 93:7 hexanes: EtOAc) to obtain the title compound **7b** (265 mg, 0.52 mmol, 83%) as a colorless oil.

TLC: R_f = 0.25 (90:10 hexanes: EtOAc).

^{1}H NMR (500 MHz, $CDCl_3$): δ 7.87 (s, 1H, ArH), 7.76 – 7.74 (m, 2H, ArH), 7.28 (t, *J* = 7.5 Hz, 1H, ArH), 7.19 (d, *J* = 7.8, 1.7 Hz, 1H, ArH), 7.13 (t, *J* = 7.7 Hz, 1H, ArH), 7.05 (d, *J* = 8.3 Hz, 2H, ArH), 6.84 (d, *J* = 8.0 Hz, 1H, ArH), 6.75 (d, *J* = 8.2 Hz, 2H, ArH), 5.07 (br, 1H, ArCH), 4.90 (s, 1H, =C*H*), 4.66 (br, 1H, ArCH), 4.43 (s, 1H, =C*H*), 3.76 (s, 3H, OMe CH_3), 1.87 (s, 3H, CH_3).

^{13}C {^{1}H, ^{19}F} NMR (126 MHz, $CDCl_3$): δ 159.7, 148.4, 144.4, 142.9, 137.0, 135.4, 134.4, 132.9, 132.0, 131.4, 130.5, 129.1, 128.1, 127.7, 127.4, 123.3, 121.4, 116.7, 113.9, 56.5, 55.3, 24.1.

HRMS-ESI (m/z): $[M+Na]^+$ calculated for $[C_{24}H_{21}F_3N_2O_5SNa]^+$: 529.1021, found 529.1004.

***N*-(4-Methoxybenzyl)-2-nitro-*N*-(2-(2-(triethylsilylperoxy)propan-2-yl)phenyl)-4-(trifluoro-methyl)benzenesulfonamide (57)**

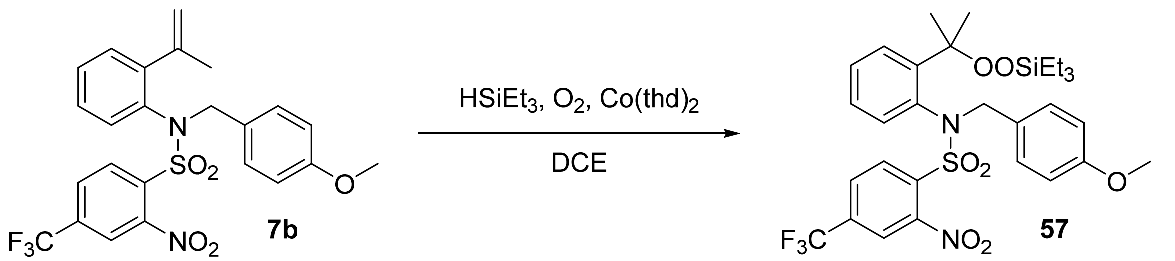

Following procedure 6B, **7b** (170 mg, 0.34 mmol, 1 eq) in DCE (2.0 mL, 0.2 M) was treated with triethylsilane (0.13 mL, 0.85 mmol, 2.5 eq), and Co(thd)$_2$ (15 mg, 0.03 mmol, 10 mol%) under an atmosphere of oxygen. The crude residue was purified by flash column chromatography on silica gel (eluent: 94:6 hexanes: EtOAc) to obtain the title compound **57** (160 mg, 0.24 mmol, 72%) as a white solid.

TLC: R_f = 0.33 (90:10 hexanes: EtOAc).

^{1}H NMR (500 MHz, CDCl$_3$): δ 7.80 (d, *J* = 7.8 Hz, 1H, ArH), 7.27 (s, 1H, ArH), 7.19 (d, *J* = 9.0 Hz, 2H, ArH), 7.11 – 7.06 (m, 2H, ArH), 7.04 (d, *J* = 7.4 Hz, 1H, ArH), 6.80 – 6.71 (m, 2H, ArH), 6.64 (t, *J* = 7.2 Hz, 1H, ArH), 6.14 (d, *J* = 7.7 Hz, 1H, ArH), 5.01 (d, *J* = 14.1 Hz, 1H, ArCH), 4.88 (d, *J* = 14.1 Hz, 1H, ArCH), 3.22 (d, *J* = 1.1 Hz, 3H, OMe CH$_3$), 1.68 (s, 3H, dimethyl CH$_3$), 1.60 (s, 3H, dimethyl CH$_3$), 0.97 (t, *J* = 7.7 Hz, 9H, SiCH$_2$C*H*$_3$), 0.65 (q, *J* = 7.6 Hz, 6H, SiC*H*$_2$CH$_3$).

^{13}C {^{1}H, ^{19}F} NMR (126 MHz, CDCl$_3$): δ 159.9, 148.8, 146.8, 135.5, 134.5, 133.3, 132.2, 131.14, 131.11, 128.7, 127.1, 126.9, 126.3, 121.0, 120.5, 113.7, 84.6, 56.9, 54.3, 28.4, 25.9, 6.7, 3.9.

HRMS-ESI (m/z): $[M+Na]^+$ calculated for $[C_{30}H_{37}F_3N_2O_7SSiNa]^+$: 677.1941, found 677.1905.

***N*-Benzyl-2-nitro-*N*-(2-(prop-1-en-2-yl)phenyl)-4-(trifluoromethyl)benzenesulfonamide (7c)**

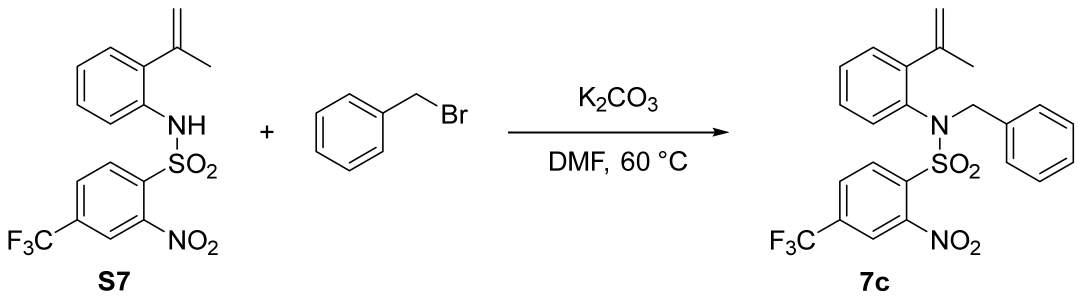

Following procedure 2A, a stirred solution of **S7** (340 mg, 0.88 mmol, 1.0 eq) in DMF (5.0 mL, 0.2 M) was treated with K_2CO_3 (146 mg, 1.06 mmol, 1.2 eq) followed by benzyl bromide (0.13 mL, 1.06 mmol, 1.2 eq). The crude residue was purified by flash column chromatography on silica gel (eluent: 94:6 hexanes: EtOAc) to obtain the title compound **7c** (389 mg, 0.82 mmol, 93%) as a white solid.

TLC: R*f* = 0.38 (50:50 hexanes: acetone).

^{1}H NMR (500 MHz, $CDCl_3$): δ 7.87 (s, 1H, ArH), 7.75 (s, 2H, ArH), 7.26 – 7.24 (m, 4H, ArH), 7.18 (d, J = 7.7 Hz, 1H, ArH), 7.13 – 7.11 (m, 3H, ArH), 6.87 (d, J = 8.0 Hz, 1H, ArH), 5.08 (br, 1H, ArCH), 4.90 (s, 1H, =C*H*), 4.75 (br, 1H, ArCH), 4.40 (s, 1H, =C*H*), 1.85 (s, 3H, CH_3).

^{13}C {^{1}H, ^{19}F} NMR (126 MHz, $CDCl_3$): δ 148.4, 144.4, 142.8, 137.0, 135.6, 135.3, 134.4, 132.9, 131.8, 130.6, 130.0, 129.1, 128.6, 128.4, 128.1, 127.7, 122.2, 121.4, 116.7, 56.9, 24.1.

HRMS-ESI (m/z): $[M+Na]^+$ calculated for $[C_{23}H_{19}F_3N_2O_4SNa]^+$: 499.0915, found 499.0913.

***N*-Benzyl-2-nitro-*N*-(2-(2-(triethylsilylperoxy)propan-2-yl)phenyl)-4-(trifluoromethyl)-benzenesulfonamide (59)**

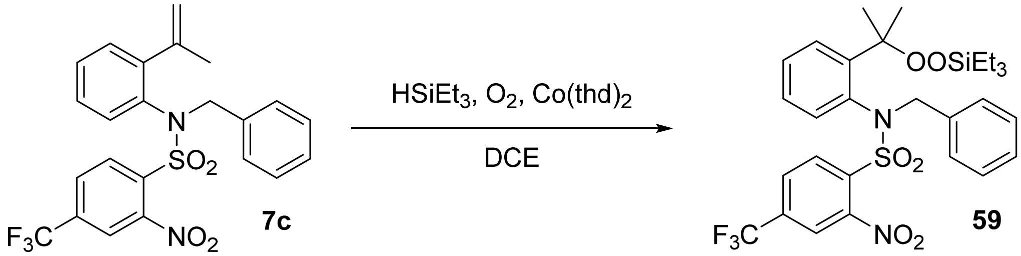

Following procedure 6B, **7c** (389 mg, 0.82 mmol, 1 eq) in DCE (4.0 mL, 0.2 M) was treated with triethylsilane (0.3 mL, 2.04 mmol, 2.5 eq), and $Co(thd)_2$ (35 mg, 0.08 mmol, 10 mol%) under an atmosphere of oxygen. The crude residue was purified by flash column chromatography on silica gel (eluent: 96:4 hexanes: EtOAc) to obtain the title compound **59** (397 mg, 0.64 mmol, 78%) as a white solid.

TLC: R_f = 0.40 (90:10 hexanes: acetone).

^{1}H NMR (500 MHz, $CDCl_3$): δ 7.91 (d, *J* = 8.1, 1.6 Hz, 1H, ArH), 7.12 – 7.01 (m, 4H, ArH), 7.00 – 6.90 (m, 3H, ArH), 6.72 – 6.63 (m, 2H, ArH), 6.25 (d, *J* = 8.0, 1.5 Hz, 1H, ArH), 5.12 (d, *J* = 13.9 Hz, 1H, ArCH), 4.77 (d, *J* = 13.9 Hz, 1H, ArCH), 1.76 (s, 3H, dimethyl CH_3), 1.55 (s, 3H, dimethyl CH_3), 0.98 (t, *J* = 7.9 Hz, 9H, $SiCH_2CH_3$), 0.67 (q, *J* = 7.9 Hz, 6H, $SiCH_2CH_3$).

^{13}C {^{1}H, ^{19}F} NMR (126 MHz, $CDCl_3$): δ 146.7, 134.3, 133.3, 132.0, 131.1, 131.0, 130.9, 130.2, 129.9, 129.0, 128.7, 128.3, 127.1, 126.8, 123.8, 121.2, 84.6, 57.4, 28.3, 25.9, 6.7, 3.9.

HRMS-ESI (m/z): $[M+Na]^+$ calculated for $[C_{29}H_{35}F_3N_2O_6SSiNa]^+$: 647.1835, found 647.1819.

***N*-(3-Benzyloxy)propyl)2-nitro-*N*-(2-(prop-1-en-2-yl)phenyl)-4-(trifluoromethyl)-benzenesulfonamide (7d)**

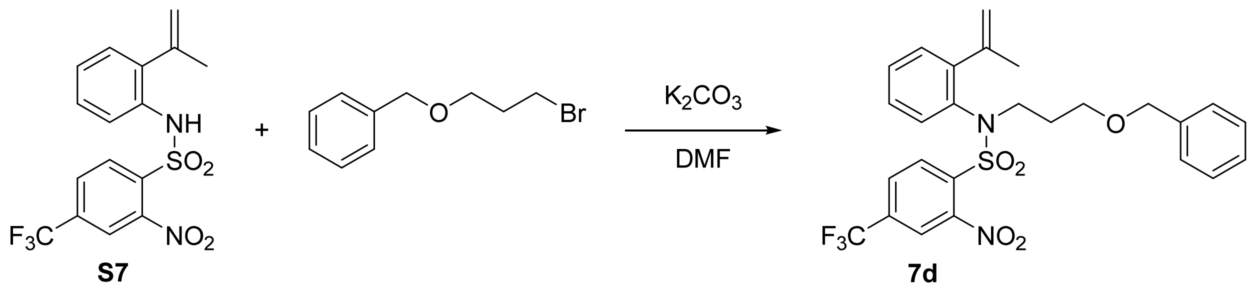

Following general procedure 2A, a stirred solution of **S7** (200 mg, 0.52 mmol, 1.0 eq) in DMF (3.0 mL, 0.2 M) was treated with K_2CO_3 (143 mg, 1.04 mmol, 2 eq), followed by ((3-bromopropoxy)methyl)benzene (0.18 mL, 1.04 mmol, 2 eq). The crude residue was purified by flash column chromatography on silica gel (eluent: 94:6 hexanes: EtOAc) to obtain the title compound **7d** (235 mg, 0.44 mmol, 85%) as a white solid.

TLC: R_f = 0.53 (50:50 hexanes: DCM).

^{1}H NMR (500 MHz, CDCl$_3$): δ 7.90 (d, *J* = 8.3 Hz, 1H, ArH), 7.87 (s, 1H, ArH), 7.81 (d, *J* = 8.3 Hz, 1H, ArH), 7.40 – 7.34 (m, 3H, ArH), 7.31 (d, *J* = 3.4 Hz, 3H, ArH), 7.28 (d, *J* = 8.4 Hz, 2H, ArH), 7.12 (d, *J* = 7.9 Hz, 1H, ArH), 5.09 (s, 1H, =C*H*), 4.79 (s, 1H, =C*H*), 4.46 (s, 2H, CH_2), 4.02 (br, 1H, ArCH), 3.80 (br, 1H, ArCH), 3.51 (t, *J* = 6.2 Hz, 2H, CH_2), 2.05 (s, 3H, CH_3), 1.89 (h, *J* = 7.3 Hz, 2H, CH_2).

^{13}C {^{1}H, ^{19}F} NMR (126 MHz, CDCl$_3$): δ 148.4, 144.3, 143.0, 138.2, 136.9, 135.7, 134.7, 132.8, 130.8, 130.6, 129.1, 128.5, 128.1, 127.9, 127.74, 127.73, 123.2, 121.3, 116.9, 73.1, 67.5, 50.1, 29.1, 24.3.

HRMS-ESI (m/z): $[M+Na]^+$ calculated for $[C_{26}H_{25}F_3N_2O_5SNa]^+$: 557.1334, found 557.1323.

***N*-(3-Benzyloxy)propyl)2-nitro-*N*-(2-(2-(triethylsilylperoxy)propan-2-yl)phenyl)-4-(trifluoromethyl)benzenesulfonamide (65)**

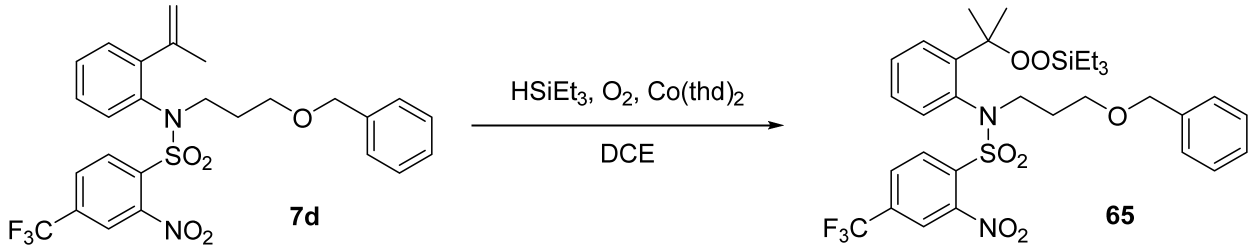

Following general procedure 6B, **7d** (123 mg, 0.23 mmol, 1 eq) in DCE (2.0 mL, 0.2 M) was treated with triethylsilane (0.1 mL, 0.58 mmol, 2.5 eq), and Co(thd)$_2$ (10 mg, 0.02 mmol, 10 mol%) under an atmosphere of oxygen. The crude residue was purified by flash column chromatography on silica gel (eluent: 95:5 hexanes: EtOAc) to obtain the title compound **65** (138 mg, 0.202 mmol, 88%) as a colorless oil.

TLC: R$_f$ = 0.48 (90:10 hexanes: EtOAc).

^{1}H NMR (500 MHz, CDCl$_3$): δ 7.97 (d, J = 8.1 Hz, 1H, ArH), 7.24 (d, J = 7.6 Hz, 2H, ArH), 7.20 – 7.17 (m, 3H, ArH), 7.14 (s, 1H, ArH), 7.08 (t, J = 7.3 Hz, 1H, ArH), 7.00 (s, 1H, ArH), 6.76 – 6.66 (m, 2H, ArH), 6.26 (d, J = 7.9 Hz, 1H, ArH), 4.21 (d, J = 2.0 Hz, 2H, ArCH$_2$), 4.10 (td, J = 12.4, 5.2 Hz, 1H, CH$_2$), 3.86 (td, J = 12.5, 4.7 Hz, 1H, CH$_2$), 3.23 (q, J = 6.0 Hz, 1H, CH$_2$), 3.17 (q, J = 4.6 Hz, 1H, CH$_2$), 2.15 (s, 3H, dimethyl CH$_3$), 1.91 (dt, J = 11.8, 5.9 Hz, 1H, CH$_2$), 1.80 (s, 3H, dimethyl CH$_3$), 1.75 (dt, J = 12.0, 6.3 Hz, 1H, CH$_2$), 0.98 (t, J = 7.9 Hz, 9H, SiCH$_2$C*H*$_3$), 0.67 (qd, J = 8.4, 5.0 Hz, 6H, SiC*H*$_2$CH$_3$).

^{13}C {^{1}H, ^{19}F} NMR (126 MHz, CDCl$_3$): δ 148.9, 146.8, 138.6, 135.8, 134.8, 134.5, 133.4, 131.4, 129.3, 128.7, 128.3, 127.46, 127.41, 126.8, 123.2, 120.5, 84.8, 72.8, 67.4, 50.8, 29.1, 28.3, 26.0, 6.7, 3.9.

HRMS-ESI (m/z): $[M+Na]^+$ calculated for $[C_{30}H_{37}F_3N_2O_7SSiNa]^+$: 705.2254, found 705.2252.

2-Nitro-4-(trifluoromethyl)-*N*-(2-styryl)benzenesulfonamide (S8)

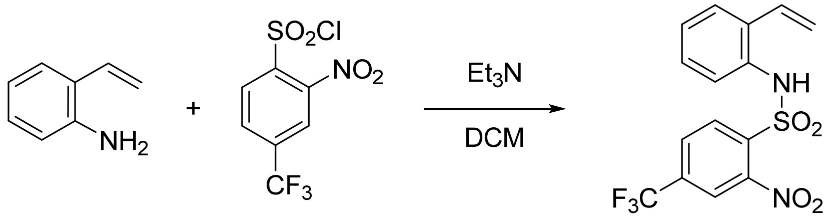

S8

2-styrylamine (310 mg, 2.6 mmol, 1.5 eq) in dry DCM (13 mL, 0.2 M) with triethylamine (0.25 mL, 1.73 mmol, 1 eq) was protected following procedure 1B by treatment with 2-nitro-4-(trifluoromethyl)benzenesulfonyl chloride (500 mg, 1.73 mmol, 1 eq). The crude product was purified by flash column chromatography on silica gel (eluent: 95:5 hexanes: EtOAc) to obtain the title compound **S8** (457 mg, 1.23 mmol, 71%) as a yellow oil.

TLC: R_f = 0.15 (90:10 hexanes: acetone).

^{1}H NMR (500 MHz, CDCl$_3$): δ 8.11 (s, 1H, ArH), 7.91 (d, *J* = 8.2 Hz, 1H, ArH), 7.83 (d, *J* = 8.2 Hz, 1H, ArH), 7.48 (d, *J* = 7.1 Hz, 1H, aniline ArH), 7.31 – 7.27 (m, 2H, aniline ArH), 7.25 – 7.19 (m, 1H, aniline ArH), 6.85 (dd, *J* = 17.3, 11.0 Hz, 1H, C*H*=CH$_2$), 5.55 (d, *J* = 17.3 Hz, 1H, =C*H*), 5.22 (d, *J* = 10.7 Hz, 1H, =C*H*).

^{13}C NMR (126 MHz, CDCl$_3$): δ 148.3, 136.6, 135.9 (q, *J* = 34.8 Hz), 134.8, 132.6, 131.8, 131.3, 129.5 (q, *J* = 3.3 Hz), 128.9, 128.3, 127.1, 123.2, 122.7 (q, *J* = 3.6 Hz), 122.1 (q, *J* = 273.2 Hz), 118.2.

HRMS-ESI (m/z): $[M+Na]^+$ calculated for $[C_{15}H_{11}F_3N_2O_4SNa]^+$: 395.0289, found 369.0278.

***N*-(2-Iodobenzyl)-2-nitro-4-(trifluoromethyl)-*N*-(2-vinylphenyl) benzenesulfonamide (8a)**

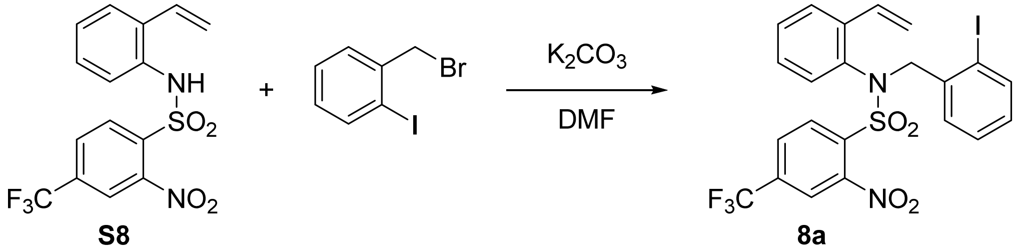

Following general procedure 2A, a stirred solution of **S8** (285 mg, 0.76 mmol, 1.0 eq) in DMF (5.0 mL, 0.2 M) was treated with K_2CO_3 (159 mg, 1.2 mmol, 1.5 eq), followed by ((3-bromopropoxy)methyl)benzene (340 mg 1.2 mmol, 1.5 eq). The crude residue was purified by flash column chromatography on silica gel (eluent: 95:5 hexanes: EtOAc) to obtain the title compound **8a** (399 mg, 0.68 mmol, 89%) as a white solid.

TLC: R*f* = 0.46 (90:10 hexanes: EtOAc).

^{1}H NMR (500 MHz, $CDCl_3$): δ 7.92 (s, 1H, ArH), 7.68 (d, *J* = 7.9 Hz, 2H, ArH), 7.62 – 7.55 (m, 2H, ArH), 7.47 (d, *J* = 7.9, 1.7 Hz, 1H, ArH), 7.32 (dt, *J* = 13.4, 7.6 Hz, 2H, ArH), 7.13 (t, *J* = 7.7 Hz, 1H, ArH), 6.94 (t, *J* = 7.7, 1.8 Hz, 1H, ArH), 6.88 (d, *J* = 7.9, 1.5 Hz, 1H, ArH), 6.59 (dd, *J* = 17.4, 11.0 Hz, 1H, C*H*=CH_2), 5.40 (d, *J* = 17.4 Hz, 1H, =C*H*), 5.26 (br, 1H, ArCH), 4.95 (br, 1H, ArCH), 4.91 (d, *J* = 11.0 Hz, 1H, =C*H*).

^{13}C {^{1}H, ^{19}F} NMR (126 MHz, $CDCl_3$): δ 148.1, 139.7, 138.3, 138.1, 136.0, 133.9, 133.6, 133.4, 133.0, 131.5, 131.2, 130.0, 129.8, 128.9, 128.8, 128.2, 126.9, 126.4, 121.4, 116.5, 100.8, 60.6.

HRMS-ESI (m/z): $[M+Na]^+$ calculated for $[C_{22}H_{16}F_3IN_2O_4SNa]^+$: 610.9725, found 610.9722.

***N*-(2-Iodobenzyl)-2-nitro-*N*-(2-(1-(triethylsilylperoxy)ethyl)phenyl)-4-(trifluoromethyl)-benzenesulfonamide (67)**

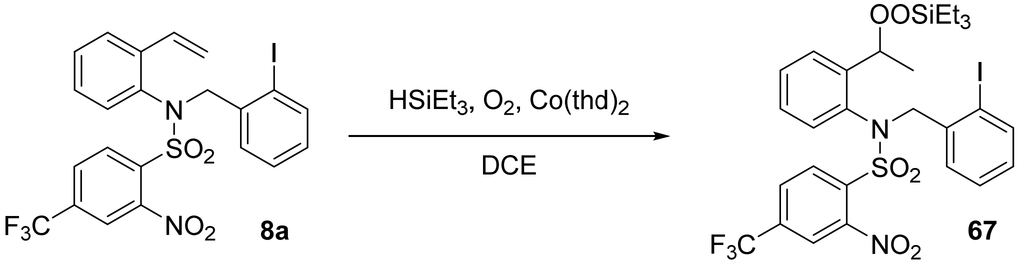

Following general procedure 6B, **8a** (400 mg, 0.68 mmol, 1 eq) in DCE (5.0 mL, 0.2 M) was treated with triethylsilane (0.26 mL, 1.7 mmol, 2.5 eq), and Co(thd)$_2$ (29 mg, 0.07 mmol, 10 mol%) under an atmosphere of oxygen. The crude product was then purified by flash column chromatography on silica gel (eluent: 95:5 hexanes: EtOAc) to obtain the title compound **67** (347 mg, 0.47 mmol, 69%) as a colorless oil.

TLC: R$_f$ = 0.50 (90:10 hexanes: EtOAc).

^{1}H NMR (500 MHz, CDCl$_3$): δ 7.96 (s, 1H, ArH), 7.74 (d, *J* = 8.3 Hz, 1H, ArH), 7.69 (d, *J* = 8.1 Hz, 1H, ArH), 7.46 (d, *J* = 7.9, 1.4 Hz, 1H, ArH), 7.38 (d, *J* = 7.7 Hz, 1H, ArH), 7.36 (d, *J* = 8.0, 1.2 Hz, 2H, ArH), 7.11 (t, *J* = 7.6, 1.5 Hz, 1H, ArH), 7.03 (d, *J* = 8.0, 1.5 Hz, 2H, ArH), 6.68 (d, *J* = 8.0, 1.5 Hz, 1H, ArH), 5.34 (d, *J* = 14.6 Hz, 1H, ArH), 5.24 (q, *J* = 6.5 Hz, 1H, CH), 4.76 (d, *J* = 14.6 Hz, 1H, ArH), 0.92 (t, *J* = 7.9 Hz, 9H, SiCH$_2$C*H*$_3$), 0.74 (d, *J* = 6.5 Hz, 3H, CH$_3$), 0.64 (q, *J* = 7.9 Hz, 6H, SiC*H*$_2$CH$_3$).

^{13}C {^{1}H, ^{19}F} NMR (126 MHz, CDCl$_3$): δ 148.1, 142.5, 136.9, 136.1, 135.8, 134.9, 134.1, 133.1, 133.0, 132.0, 131.8, 131.7, 131.5, 129.9, 128.4, 128.2, 122.5, 121.78, 121.73, 73.6, 56.3, 19.3, 6.8, 3.9.

HRMS-ESI (m/z): [M+Na]$^+$ calculated for [$C_{28}H_{32}F_3IN_2O_6SSiNa$]$^+$: 759.0645, found 759.0627.

2-Allyl-5-bromoindoline (S9)

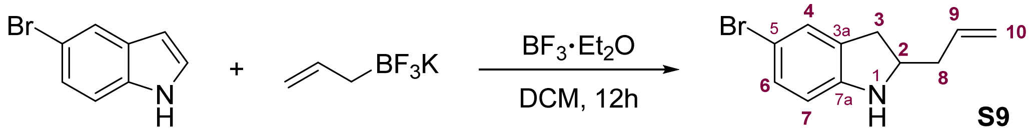

Following the literature protocol,[13] 5-bromo-1*H*-indole (500 mg, 2.55 mmol, 1 eq) in dry DCM (25 mL, 0.1 M) was treated with potassium allyltrifluoroborate (754 mg, 5.10 mmol, 2 eq), and $BF_3 \cdot Et_2O$ (0.31 mL, 2.55 mmol, 1 eq). The reaction mixture was vigorously stirred at RT for 12h, then quenched by addition of 1 M HCl (5.0 mL), neutralized by addition of saturated aqueous $NaHCO_3$, extracted with Et_2O and dried over $MgSO_4$. The solvent was removed *in vacuo* to afford the crude title compound **S9** (603 mg, 2.52 mmol, 99%) as a burgundy oil with spectral data consistent with the literature[13] that was carried to the next step without further purification.

TLC: R_f = 0.51 (80:20 hexanes: EtOAc).

^{1}H NMR (500 MHz, CDCl$_3$): δ 7.16 (s, 1H, ArH^4), 7.09 (d, *J* = 8.2 Hz, 1H, ArH^6), 6.45 (d, *J* = 8.2 Hz, 1H, ArH^7), 5.87 – 5.74 (m, 1H, C^9*H*=CH$_2$), 5.14 (s, 1H, =C^{10}*H*), 5.11 (d, *J* = 4.5 Hz, 1H, =C^{10}*H*), 3.90 (p, *J* = 7.2 Hz, 1H, C^{2}H), 3.80 (br, 1H, NH), 3.12 (dd, *J* = 15.8, 8.8 Hz, 1H, C^{3}H), 2.72 (dd, *J* = 15.9, 6.9 Hz, 1H, C^{3}H), 2.39 – 2.24 (m, 2H, C^{8}H$_2$).

^{13}C NMR (126 MHz, CDCl$_3$): δ 149.7, 134.9, 131.02, 130.03, 127.8, 117.9, 110.5, 110.1, 59.0, 40.9, 35.5.

HRMS-ESI (m/z): $[M+H]^+$ calculated for $[C_{11}H_{13}BrN]^+$: 240.0206, found 240.0193.

2-Allyl-5-bromo-1-((2-nitro-4-(trifluoromethyl)phenyl)sulfonyl)indoline (9a)

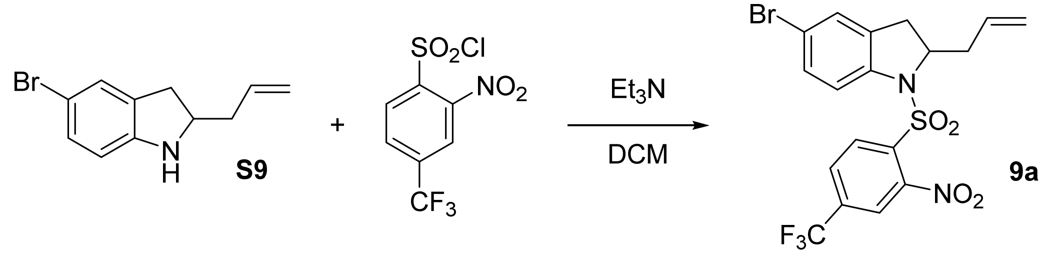

Following a modified procedure 1A, a solution of **S9** (300 mg, 1.26 mmol, 1 eq) in dry DCM (6 mL, 0.2 M) with triethylamine (0.37 mL, 2.52 mmol, 2 eq) was treated with 2-nitro-4-(trifluoromethyl)benzenesulfonyl chloride (730 mg, 2.52 mmol, 2 eq) and stirred for 48 h. The crude product was purified by flash column chromatography on silica gel (eluent: 95:5 hexanes: acetone) to obtain the title compound **9a** (513 mg, 1.05 mmol, 83%) as a colorless oil.

TLC: R_f = 0.36 (90:10 hexanes: acetone).

^{1}H NMR (500 MHz, CDCl$_3$): δ 8.00 (d, J = 8.2 Hz, 1H, ArH), 7.84 (s, 1H, ArH), 7.82 (s, 1H, ArH), 7.37 (d, J = 8.5 Hz, 1H, ArH), 7.33 (d, J = 9.3 Hz, 1H, ArH), 7.29 (s, 1H, ArH), 5.73 (td, J = 17.4, 7.0 Hz, 1H, C*H*=CH$_2$), 5.14 (d, J = 7.5 Hz, 1H, =C*H*), 5.11 (s, 1H, =C*H*), 4.67 (td, J = 8.5, 5.7 Hz, 1H, CH), 3.20 (dd, J = 16.4, 9.1 Hz, 1H, ArCH), 2.77 (d, J = 16.5 Hz, 1H, ArCH), 2.55 (dt, J = 11.5, 5.7 Hz, 1H, CH$_2$), 2.43 (dt, J = 14.6, 7.9 Hz, 1H, CH$_2$).

^{13}C {^{1}H, ^{19}F} NMR (126 MHz, CDCl$_3$): δ 148.4, 139.0, 136.2, 135.1, 134.2, 132.4, 132.0, 131.1, 129.2, 128.4, 122.0, 121.8, 119.4, 118.3, 117.2, 63.2, 40.8, 33.0.

HRMS-ESI (m/z): $[M+Na]^+$ calculated for $[C_{18}H_{14}BrF_3N_2O_4SNa]^+$: 514.9687, found 514.9675.

5-Bromo-1-((2-nitro-4-(trifluoromethyl)phenyl)sulfonyl)-2-(2-(triethylsilylperoxy)propyl)-indoline (9b)

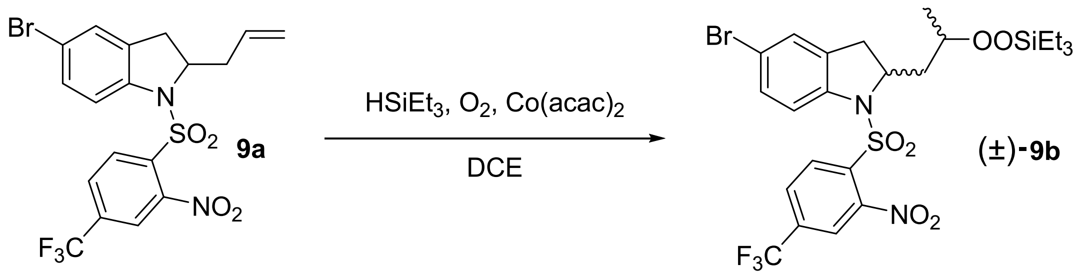

Following general procedure 6A, **9a** (750 mg, 1.53 mmol, 1 eq) in DCE (8.0 mL, 0.2 M) was treated with triethylsilane (0.56 mL, 3.8 mmol, 2.5 eq), and $Co(acac)_2$ (39 mg, 0.15 mmol, 10 mol%) under an atmosphere of oxygen. The crude product was then purified by flash column chromatography on silica gel (eluent: 95:5 hexanes: EtOAc) to obtain the title compound **9b** (548 mg, 0.857 mmol, 56%) as a colorless oil.

TLC: R_f = 0.42 (90:10 hexanes: acetone).

^{1}H NMR (500 MHz, $CDCl_3$): δ 7.95 (d, J = 8.2 Hz, 1H, ArH), 7.85 – 7.77 (m, 2H, ArH), 7.40 (d, J = 8.6 Hz, 1H, ArH), 7.35 (t, J = 6.5 Hz, 1H, ArH), 7.30 (s, 1H, ArH), 4.80 (q, CH, J = 7.8 Hz, 1H, CH), 4.20 (h, J = 6.3 Hz, 1H, CH), 3.19 (dd, J = 16.4, 9.0 Hz, 1H, ArCH), 2.85 (d, J = 16.5 Hz, 1H, ArCH), 2.15 (ddd, J = 14.1, 6.9, 5.4 Hz, 1H, CH_2), 1.80 (ddd, J = 13.7, 8.5, 4.9 Hz, 1H, CH_2), 1.28 (d, J = 6.2 Hz, 3H, CH_3), 0.98 (t, J = 7.9 Hz, 9H, $SiCH_2C\mathit{H}_3$), 0.68 (q, J = 8.1 Hz, 6H, $SiCH_2C\mathit{H}_3$).

^{13}C {^{1}H, ^{19}F} NMR (126 MHz, $CDCl_3$): δ 148.4, 139.0, 136.1, 135.0, 134.7, 132.0, 131.1, 129.2, 128.3, 122.0, 121.7, 118.5, 118.0, 78.0, 61.4, 40.8, 34.1, 18.6, 6.9, 3.9.

HRMS-ESI (m/z): $[M+Na]^+$ calculated for $[C_{24}H_{30}BrF_3N_2O_6SSiNa]^+$: 663.0607, found 663.0580.

5-Bromo-2-(2-(*tert*-Butyldiphenylsilylperoxy)propyl)-1-((2-nitro-4-(trifluoromethyl)-phenyl)sulfonyl)indoline (70 and 71)

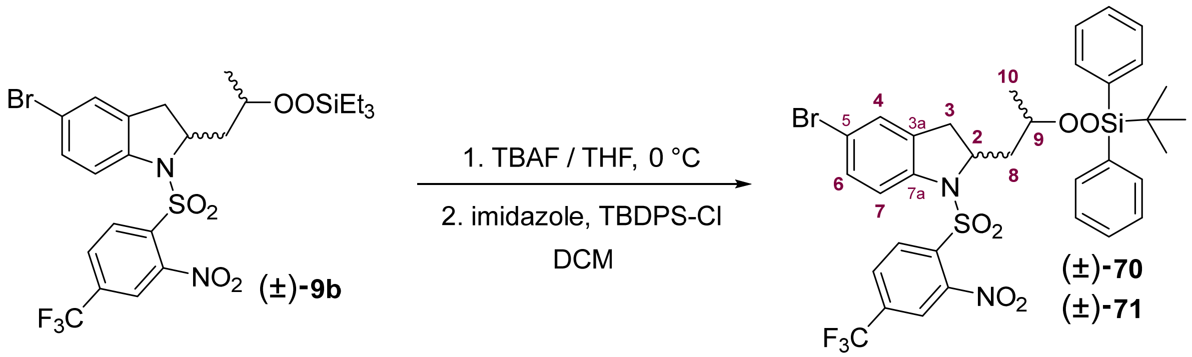

Following procedure 7, TBAF (0.31 mL, 1.0 M in THF, 1.2 eq) was added to a solution of **9b** (164 mg, 0.256 mmol, 1 eq) in THF (2.0 mL, 0.2 M). Upon completion, the residue was dissolved in DCM (2.0 mL, 0.2 M) and treated with imidazole (26 mg, 0.38 mmol, 1.5 eq) and TBDPS-Cl (0.07 mL, 0.256 mmol, 1 eq). The crude product was purified by flash column chromatography on silica gel (eluent: 95:5 hexanes: EtOAc) to obtain a 1:1.1 (*anti*: *syn*) mixture of diastereomers **70** and **71** (180 mg, 0.24 mmol, 92% over 2 steps) as a colorless oil.

TLC: R_f = 0.27 (90:10 hexanes: acetone).

^{1}H NMR (500 MHz, C_6D_6): δ 7.89 – 7.82 (m, 3H, ArH), 7.76 (dd, *J* = 7.5, 2.0 Hz, 1H, ArH), 7.36 (dd, *J* = 8.4, 3.2 Hz, 1H, ArH), 7.30 (dd, *J* = 8.5, 5.0 Hz, 1H, ArH), 7.25 (d, *J* = 6.8 Hz, 1H, ArH), 7.24 – 7.20 (m, 4H, ArH), 7.18 (s, 1H, ArH), 7.10 (dd, *J* = 19.5, 9.6 Hz, 1H, ArH), 6.95 (d, *J* = 12.0 Hz, 1H, ArH), 6.86 (s, 0.5H, ArH), 6.75 (s, 0.5H, ArH), 6.57 (t, *J* = 9.2 Hz, 1H, ArH), 4.60 – 4.49 (m, 1H, C^9H), 4.28 (h, *J* = 6.0 Hz, 0.5H, C^2H), 4.05 (h, *J* = 5.9 Hz, 0.5H, C^2H), 2.55 – 2.45 (m, 1H, C^3H), 2.21 (d, *J* = 16.8 Hz, 0.5H, C^3H), 2.16 – 2.10 (m, 0.5H, C^8H), 2.08 (d, *J* = 14.8 Hz, 0.5H, C^3H), 1.81 (dt, *J* = 14.3, 4.4 Hz, 0.5H, C^8H), 1.66 (dt, *J* = 14.2, 8.6 Hz, 0.5H, C^8H), 1.37 (ddd, *J* = 14.0, 9.5, 4.4 Hz, 0.5H, C^8H), 1.18 (s, 9H, *t*Bu), 1.12 (d, *J* = 6.4 Hz, 1.5H, $C^{10}H_3$), 1.10 (d, *J* = 6.3 Hz, 1.5H, $C^{10}H_3$).

^{13}C {^{1}H, ^{19}F} NMR (126 MHz, C_6D_6): δ 148.5, 148.3, 139.5, 139.2, 136.21, 136.18, 136.1, 136.1, 135.3, 135.2, 135.0, 134.9, 134.8, 134.2, 133.3, 133.2, 133.1, 132.1, 131.6, 131.1, 130.9, 130.4, 130.36, 130.32, 129.3, 129.2, 122.22, 122.19, 121.23, 121.18, 118.4, 118.3, 118.2, 117.7, 80.3, 78.5, 62.9, 61.1, 41.8, 40.9, 34.8, 33.5, 27.53, 27.47, 19.6, 19.3, 18.6.

HRMS-ESI (m/z): $[M+Na]^+$ calculated for $[C_{34}H_{34}BrF_3N_2O_6SSiNa]^+$: 785.0920, found 787.0908.

***N*-(4-Hydroxybutyl)-*N*-(naphthalen-1-ylmethyl)-2-nitro-4-(trifluoromethyl)-benzenesulfonamide (3l)**

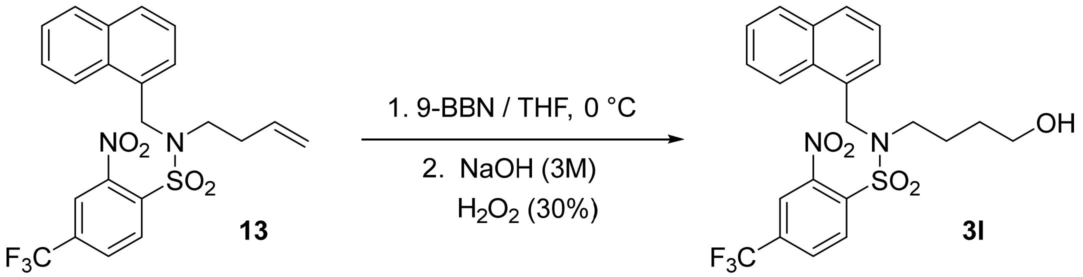

Following procedure 3, **13** (1.22 g, 2.63 mmol, 1 eq) in dry THF (6.0 mL, 0.4 M) was treated with 9-BBN 0.5 M in THF (10.5 mL, 2 eq), 3 M NaOH (3.6 mL, 1.2 eq) and aq. 30% H_2O_2 (3.6 mL, 1.2 eq). The crude product was purified by flash column chromatography on silica gel (eluent: 55:45 hexanes: EtOAc) to obtain the title compound **3l** (930 mg, 1.92 mmol, 73%) as a colorless oil.

TLC: R*f* = 0.10 (70:30 hexanes: EtOAc).

^{1}H NMR (500 MHz, $CDCl_3$): δ 8.01 (d, *J* = 7.8, 1.6 Hz, 1H, ArH), 7.77 – 7.71 (m, 4H, ArH), 7.56 (d, *J* = 8.3, 1.8 Hz, 1H, ArH), 7.49 – 7.41 (m, 3H, ArH), 7.36 (dd, *J* = 8.2, 7.0 Hz, 1H, ArH), 4.95 (s, 2H, $ArCH_2$), 3.50 (t, *J* = 6.2 Hz, 2H, CH_2), 3.47 (d, *J* = 7.5 Hz, 1H, CH_2), 1.54 (p, *J* = 7.3 Hz, 2H, CH_2), 1.43 (dt, *J* = 13.1, 6.6 Hz, 2H, CH_2), 1.25 (br, 1H, OH).

^{13}C {^{1}H, ^{19}F} NMR (126 MHz, $CDCl_3$): δ 147.6, 137.0, 135.2, 133.8, 131.8, 131.7, 130.4, 129.5, 128.8, 128.1, 128.0, 126.9, 126.3, 125.2, 123.2, 121.4, 121.0, 62.2, 50.6, 49.4, 29.5, 25.0.

HRMS-ESI (m/z): $[M+Na]^+$ calculated for $[C_{22}H_{21}F_3N_2O_5SNa]^+$: 505.1021, found 505.0991.

***N*-(4-Iodobutyl)-*N*-(naphthalen-1-ylmethyl)-2-nitro-4-(trifluoromethyl)benzenesulfonamide (3m)**

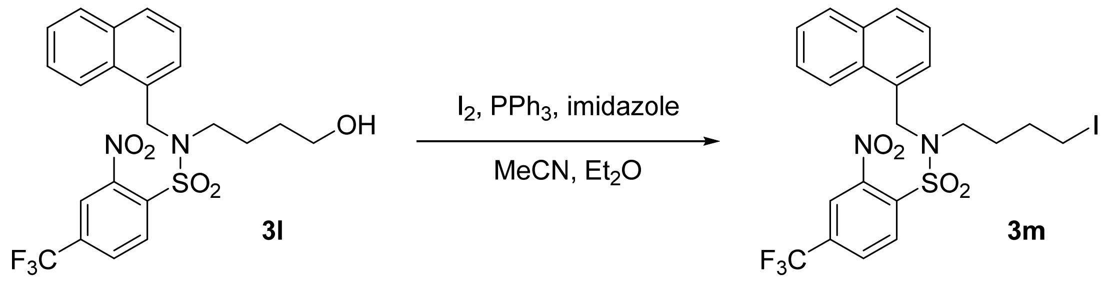

Following procedure 4, triphenylphosphine (758 mg, 2.9 mmol, 1.5 eq), imidazole (200 mg, 2.9 mmol, 1.5 eq) and iodine (735 mg, 2.9 mmol, 1.5 eq) were added subsequently to a solution of **3l** (930 mg, 1.93 mmol, 1 eq) in a 1:3 ratio of MeCN and Et_2O (15 mL, 0.2 M) at RT. The crude product was purified by flash column chromatography on silica gel (eluent: 94:6 hexanes: EtOAc) to obtain the title compound **3m** (1.2 g, 1.99 mmol, 97%) as a colorless oil.

TLC: R_f = 0.47 (80:20 hexanes: EtOAc).

^{1}H NMR (500 MHz, $CDCl_3$): δ 8.01 (d, *J* = 7.9, 1.6 Hz, 1H, ArH), 7.83 – 7.72 (m, 4H, ArH), 7.60 (d, *J* = 8.3 Hz, 1H, ArH), 7.48 – 7.42 (m, 3H, ArH), 7.40 – 7.34 (m, 1H, ArH), 4.94 (s, 2H, $ArCH_2$), 3.44 (t, *J* = 7.5 Hz, 2H, CH_2), 3.00 (t, *J* = 6.7 Hz, 2H, CH_2), 1.67 (p, *J* = 6.9 Hz, 2H, CH_2), 1.52 (p, *J* = 7.6 Hz, 2H, CH_2).

^{13}C {^{1}H, ^{19}F} NMR (126 MHz, $CDCl_3$): δ 147.6, 136.8, 134.9, 133.8, 131.8, 131.6, 130.1, 129.5, 128.8, 128.2, 128.1, 127.0, 126.3, 125.2, 123.2, 121.4, 120.9, 50.6, 48.2, 30.2, 29.2, 5.9.

HRMS-ESI (m/z): $[M+Na]^+$ calculated for $[C_{22}H_{20}F_3IN_2O_4SNa]^+$: 615.0038, found 615.0021.

***N*-(4-Hydroperoxybutyl)-*N*-(naphthalen-1-ylmethyl)-2-nitro-4-(trifluoromethyl)-benzenesulfonamide (3n)**

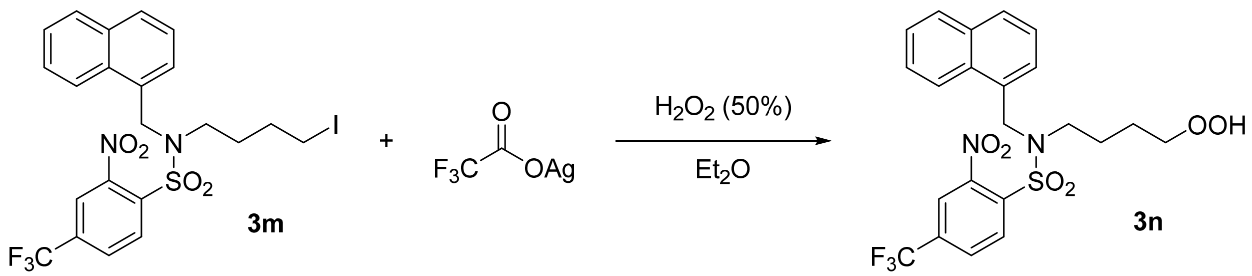

Following procedure 5, **3m** (970 mg, 1.64 mmol, 1 eq) in diethyl ether (8.0 mL, 0.2 M) was treated with silver trifluoroacetate (435 mg, 1.97 mmol, 1.2 eq) and aqueous 50% hydrogen peroxide (1.86 mL, 2 eq). The crude hydroperoxide was then purified by flash column chromatography on silica gel (eluent: 70:30 hexanes: EtOAc) to obtain the title compound **3n** (400 mg, 0.8 mmol, 49%) as a colorless oil.

TLC: R_f = 0.12 (80:20 hexanes: EtOAc).

^{1}H NMR (500 MHz, $CDCl_3$): δ 8.00 (d, *J* = 6.4, 2.8 Hz, 2H, ArH), 7.79 – 7.70 (m, 4H, ArH), 7.57 (d, *J* = 8.3, 1.8 Hz, 1H, ArH), 7.50 – 7.40 (m, 3H, ArH), 7.36 (dd, *J* = 8.2, 7.0 Hz, 1H, ArH), 4.94 (s, 2H, $ArCH_2$), 3.86 (t, *J* = 5.3 Hz, 2H, CH_2), 3.47 (t, *J* = 7.1 Hz, 2H, CH_2), 1.56 – 1.47 (m, 4H, overlapping CH_2).

^{13}C NMR (126 MHz, $CDCl_3$): δ 147.6, 136.9, 134.9 (q, *J* = 34.3 Hz), 133.7, 131.8, 131.6, 130.3, 129.5, 128.8, 128.2, 128.1 (q, *J* = 3.5 Hz), 126.9, 126.3, 125.2, 123.2, 121.4 (q, *J* = 3.6 Hz), 121.0 (q, *J* = 273.4 Hz), 76.2, 50.6, 49.3, 25.1, 24.7.

HRMS-ESI (m/z): $[M+Na]^+$ calculated for $[C_{22}H_{21}F_3N_2O_6SNa]^+$: 521.0970, found 521.0940.

N-(4-(tert-Butyldiphenylsilylperoxy)butyl)-N-(naphthalen-1-ylmethyl)-2-nitro-4-(trifluoromethyl)benzenesulfonamide (74)

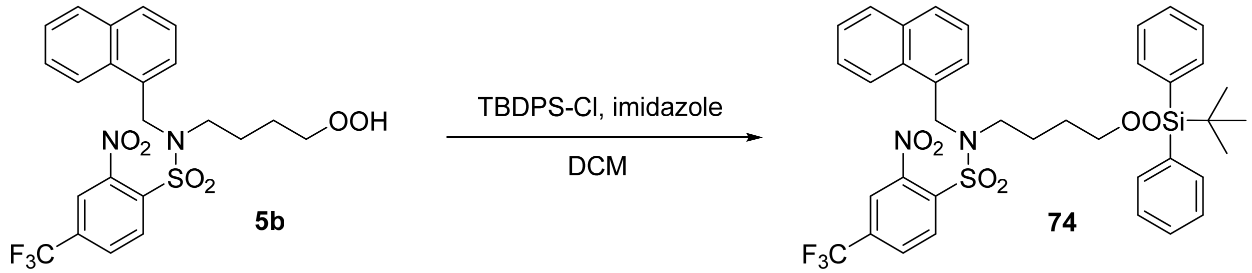

Following procedure 8, **5b** (170 mg, 0.34 mmol, 1 eq) in DCM (2.0 mL, 0.2 M) was treated with imidazole (28 mg, 0.41 mmol, 1.5 eq) and TBDPS-Cl (0.01 mL, 0.34 mmol, 1 eq). The crude product was then purified by flash column chromatography on silica gel (eluent: 95:5 hexanes: EtOAc) to obtain the title compound **74** (220 mg, 0.3 mmol, 88%) as a colorless oil.

TLC: R_f = 0.21 (90:10 hexanes: acetone).

^{1}H NMR (500 MHz, C_6D_6): δ 7.87 (d, *J* = 8.4 Hz, 1H, ArH), 7.82 – 7.74 (m, 4H, ArH), 7.41 (d, *J* = 7.5 Hz, 1H, ArH), 7.35 (d, *J* = 8.2 Hz, 1H, ArH), 7.20 – 7.11 (m, 10H, ArH), 7.00 – 6.93 (m, 2H, ArH), 6.57 (d, *J* = 8.3 Hz, 1H, ArH), 4.58 (s, 2H, $ArCH_2$), 3.60 (t, *J* = 5.3 Hz, 2H, CH_2), 3.11 (t, *J* = 6.8 Hz, 2H, CH_2), 1.12 – 1.14 (m, 4H, overlapping CH_2), 1.13 (s, 9H, *t*Bu).

^{13}C NMR (126 MHz, C_6D_6): δ 147.8, 137.2, 136.1, 135.3 (q, *J* = 34.6 Hz), 134.3, 134.1, 133.2, 132.0, 131.8, 130.9, 130.3, 129.3, 128.9 (q, *J* = 3.7 Hz), 127.4, 127.4, 126.9, 126.3, 125.2, 123.6, 121.0 (q, *J* = 3.6 Hz), 120.9 (q, *J* = 273.4 Hz), 76.3, 50.3, 49.3, 27.6, 25.3, 24.9, 19.6.

HRMS-ESI (m/z): $[M+Na]^+$ calculated for $[C_{38}H_{39}F_3N_2O_6SSiNa]^+$: 759.2148, found 759.2128.

6) Synthesis and Characterization of Cyclic Hydroxylamines

5-Methyl-2-(naphthalen-1-ylmethyl)isoxazolidine (29)

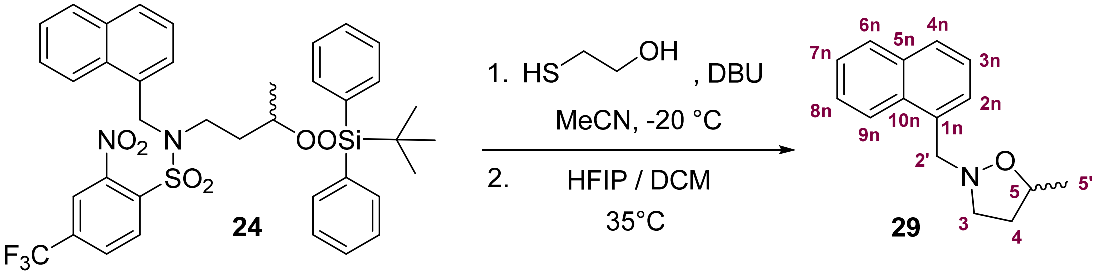

Following procedure 9B, **24** (200 mg, 0.27 mmol, 1 eq) in MeCN (2.0 mL, 0.2 M) was treated with 2-mercaptoethanol (0.2 mL, 0.27 mmol, 1 eq) and DBU (0.02 ml, 0.27 mmol, 1 eq). The crude mixture was diluted in DCM (2.0 mL, 0.2 M) and treated with HFIP (0.03 mL, 0.27 mmol, 1 eq) for 2h. The final cyclized product **29** (56 mg, 0.25 mmol, 91%) was isolated after purification by flash column chromatography on silica gel (eluent: 93:7 hexanes: EtOAc + 1% Et_3N) as a colorless oil.

TLC: R_f = 0.54 (95:5 hexanes: EtOAc + 1% Et_3N).

^{1}H NMR RT (500 MHz, C_6D_6): δ 8.42 (d, J = 8.4 Hz, 1H, ArH^{9n}), 7.66 (d, J = 8.1 Hz, 1H, ArH^{6n}), 7.61 (d, J = 8.3 Hz, 1H, ArH^{4n}), 7.46 (d, J = 6.5 Hz, 1H, ArH^{8n}), 7.35 (t, J = 7.8 Hz, 1H, ArH^{7n}), 7.29 – 7.24 (m, 2H, ArH^{3n} and ArH^{2n}), 4.65 (br, 2H, $C^{2'}H_2$), 4.10 (br, 1H, C^5H), 2.74 (br, 2H, C^3H_2), 1.93 (dt, J = 16.6, 7.7 Hz, 1H, C^4H), 1.42 (dt, J = 13.6, 6.3 Hz, 1H, C^4H), 1.11 (d, J = 6.0 Hz, 3H, $C^{5'}H_3$).

^{1}H NMR 70 °C (500 MHz, C_6D_6): δ 8.38 (d, J = 8.5 Hz, 1H, ArH^{9n}), 7.66 (d, J = 8.1 Hz, 1H, ArH^{6n}), 7.60 (d, J = 8.2 Hz, 1H, ArH^{4n}), 7.47 (d, J = 6.9 Hz, 1H, ArH^{8n}), 7.34 (t, J = 7.7 Hz, 1H, ArH^{7n}), 7.29 – 7.24 (m, 2H, ArH^{3n} and ArH^{2n}), 4.35 (br, 1H, $C^{2'}H$), 4.28 (br, 1H, $C^{2'}H$), 4.10 (h, J = 6.2 Hz, 1H, C^5H), 2.69 (br, 1H, C^3H), 2.60 (br, 1H, C^3H), 1.98 (dq, J = 16.2, 7.6 Hz, 1H, C^4H), 1.42 (td, J = 13.6, 6.3 Hz, 1H, C^4H), 1.12 (d, J = 6.0 Hz, 3H, $C^{5'}H_3$).

^{13}C NMR RT (126 MHz, C_6D_6): δ 134.4 (C^{10n}), 134.1 C^{5n}), 133.0 (C^{1n}), 128.7 (C^{6n}), 128.4 (C^{4n}), 127.6 (C^{8n}), 126.1 (C^{7n}), 125.9 (C^{3n}), 125.5 (C^{2n}), 125.4 (C^{9n}), 73.0 (br, C^5), 60.8 (br, $C^{2'}$), 54.7 (br, C^3), 36.0 (C^4), 20.6 (br, $C^{5'}$).

^{13}C NMR 70 °C (126 MHz, C_6D_6): δ 134.1 (C^{10n}), 133.9 (C^{5n}), 132.7 (C^{1n}), 128.3 (C^{6n}), 128.0 (C^{4n}), 127.1 (C^{8n}), 125.7 (C^{7n}), 125.4 (C^{3n}), 125.1 (C^{2n}), 124.9 (C^{9n}), 72.7 (C^5), 60.5 ($C^{2'}$), 54.4 (C^3), 35.9 (C^4), 20.3 ($C^{5'}$).

HRMS-ESI (m/z): $[M+H]^+$ calculated for $[C_{16}H_{17}NO]^+$: 228.1383, found 228.1380.

2-(4-Fluorobenzyl)-5-methylisoxazolidine (35)

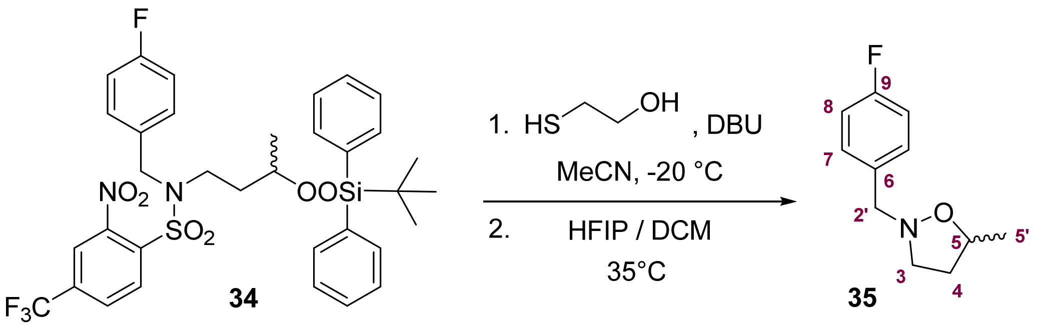

Following procedure 9B, **34** (497 mg, 0.71 mmol, 1 eq) in dry MeCN (4.0 mL, 0.2 M) was treated with a solution of 2-mercaptoethanol (0.64 mL, 0.71 mmol, 1 eq) and DBU (0.05 ml, 0.71 mmol, 1 eq) in MeCN (0.7 mL, 1 M). The crude mixture was then diluted in DCM (4 mL, 0.2 M) and treated with HFIP (0.07 mL, 0.71 mmol, 1 eq) for 2h. The final cyclized product was isolated after purification by flash column chromatography on silica gel (eluent: 80:20 hexanes: EtOAc) to obtain the title compound **35** (126 mg, 0.65 mmol, 92%) as a colorless oil.

TLC: R_f = 0.44 (80:20 hexanes: EtOAc + 1% Et_3N).

^{1}H NMR RT (500 MHz, C_6D_6): δ 7.18 (d, *J* = 5.3 Hz, 2H, ArH^{2p}), 6.82 (t, *J* = 8.6 Hz, 2H, ArH^{3p}), 4.03 (h, *J* = 6.3 Hz, 1H, C^{5}H), 3.63 (br, 2H, C$^{2'}$H$_2$), 2.81 (br, 1H, C^{3}H), 2.22 (br, 1H, C^{3}H), 1.93 (dt, *J* = 19.4, 7.1 Hz, 1H, C^{4}H), 1.36 (br, 1H, C^{4}H), 1.08 (d, *J* = 5.9 Hz, 3H, C$^{5'}$H$_3$).

^{1}H NMR 65 °C (500 MHz, C_6D_6): δ 7.18 (d, *J* = 6.9 Hz, 2H, ArH^{2p}), 6.82 (t, *J* = 8.4 Hz, 2H, ArH^{3p}), 4.03 (h, *J* = 6.0 Hz, 1H, C^{5}H), 3.72 (br, 2H, C$^{2'}$H$_2$), 2.63 (br, 1H, C^{3}H), 2.53 (br, 1H, C^{3}H), 1.93 (dt, *J* = 19.4, 7.1 Hz, 1H, C^{4}H), 1.46 – 1.36 (m, 1H, C^{4}H), 1.08 (d, *J* = 5.9 Hz, 3H, C$^{5'}$H$_3$).

^{13}C NMR (126 MHz, C_6D_6): δ 162.27 (d, *J* = 244.5 Hz, C^{4p}), 133.95 (d, *J* = 3.2 Hz, C^{1p}), 130.37 (d, *J* = 7.8 Hz, C^{3p}), 114.74 (d, *J* = 21.3 Hz, C^{2p}), 72.7 (C^5), 61.4 (C$^{2'}$), 54.4 (C^3), 35.8 (C^4), 20.2 (C$^{5'}$).

^{19}F NMR (470 MHz, C_6D_6): δ -115.7 (ddd, *J* = 14.3, 9.0, 5.5 Hz, 1F).

HRMS-ESI (m/z): $[M+H]^+$ calculated for $[C_{12}H_{14}NO]^+$: 196.1133, found 196.1129.

2-(Methoxyphenethyl)-5-methylisoxazolidine (37)

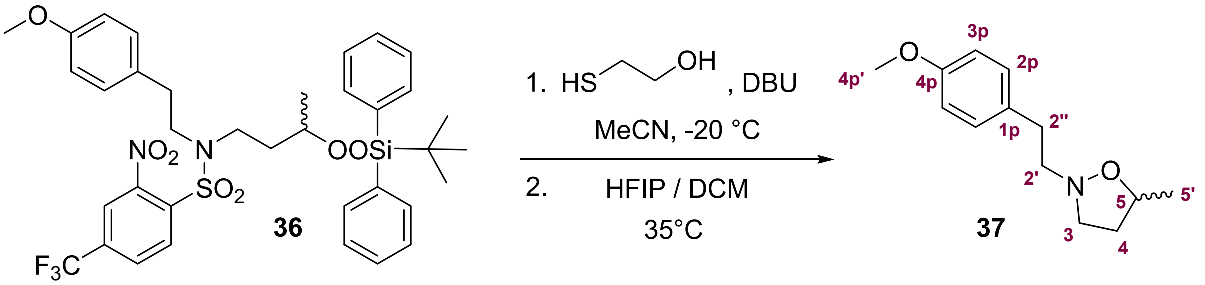

Following procedure 9B, **36** (340 mg, 0.47 mmol, 1 eq) in dry MeCN (3.0 mL, 0.2 M) was treated with a solution of 2-mercaptoethanol (0.4 mL, 0.47 mmol, 1 eq) and DBU (0.08 ml, 0.47 mmol, 1 eq) in MeCN (0.5 mL, 1 M). The crude mixture was then diluted in DCM (3.0 mL, 0.2 M) and treated with HFIP (0.05 mL, 0.47 mmol, 1 eq) for 2h. The final cyclized product was

isolated after purification by flash column chromatography on silica gel (eluent: 65:35 hexanes: EtOAc) to obtain the title compound **37** (89 mg, 0.40 mmol, 86%) as a colorless oil.

TLC: R_f = 0.29 (70:30 hexanes: EtOAc).

^{1}H NMR RT (500 MHz, C_6D_6): δ 7.08 (d, *J* = 8.2 Hz, 2H, ArH^{2p}), 6.78 (d, *J* = 8.4 Hz, 2H, ArH^{3p}), 4.07 (h, *J* = 6.3 Hz, 1H, C^5H), 3.32 (s, 3H, $C^{4p'}H_3$), 2.97 (t, *J* = 7.2 Hz, 2H, $C^{2''}H_2$), 2.93 (br, 4H, $C^{2'}H_2$), 2.25 (br, 2H, C^3H_2), 1.96 (br, 1H, C^4H), 1.39 (q, *J* = 8.9 Hz, 1H, C^4H).1.14 (d, *J* = 6.0 Hz, 3H, $C^{5'}H_3$).

^{1}H NMR 65 °C (500 MHz, C_6D_6): δ 7.08 (d, *J* = 8.5 Hz, 2H, ArH^{2p}), 6.78 (d, *J* = 8.4 Hz, 2H, ArH^{3p}), 4.07 (h, *J* = 6.1 Hz, 1H, C^5H), 3.38 (s, 3H, $C^{4p'}H_3$), 2.94 (br, 4H, $C^{2'}H_2$ and $C^{2''}H_2$ overlapping), 2.67 (br, 1H, C^3H), 2.59 (br, 1H, C^3H), 2.00 (dt, *J* = 19.4, 7.6 Hz, 1H, C^4H), 1.43 (dt, *J* = 19.2, 7.6 Hz, 1H, C^4H), 1.14 (d, *J* = 6.0 Hz, 3H, $C^{5'}H_3$).

^{13}C NMR RT (126 MHz, C_6D_6): δ 158.7 (C^{1p}), 132.8 (C^{4p}), 130.1 (C^{2p}), 114.2 (C^{3p}), 72.9 (br, C^5), 60.8 (br, C^3), 55.5 (br, $C^{2'}$), 54.8 ($C^{4p'}$), 36.2 (C^4), 34.4 ($C^{2''}$), 20.6 (br, $C^{5'}$).

^{13}C NMR 65 °C (126 MHz, C_6D_6): δ 158.9 (C^{1p}), 133.1 (C^{4p}), 130.1 (C^{2p}), 114.4 (C^{3p}), 72.7 (C^5), 60.8 (C^3), 55.5 ($C^{2'}$), 54.9 ($C^{4p'}$), 36.4 (C^4), 34.4 ($C^{2''}$), 20.7 ($C^{5'}$).

HRMS-ESI (m/z): $[M+H]^+$ calculated for $[C_{14}H_{19}NO_2]^+$: 222.1489, found 222.1483.

5-Methyl-2-phenylisoxazolidine (39)

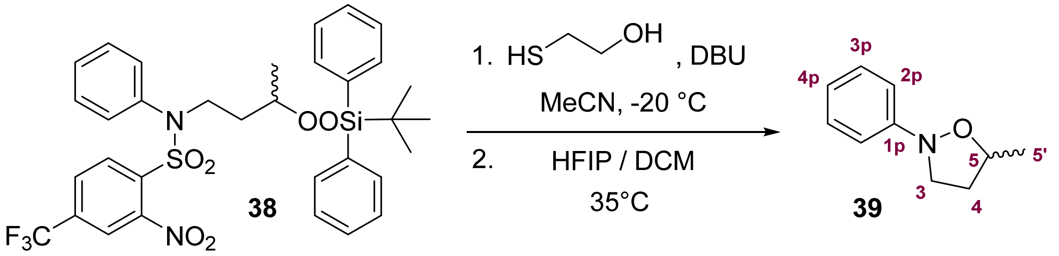

Following procedure 9B, **38** (410 mg, 0.61 mmol, 1 eq) in dry MeCN (3.0 mL, 0.2 M) was treated with a solution of 2-mercaptoethanol (0.52 mL, 0.61 mmol, 1 eq) and DBU (0.04 ml, 0.61 mmol, 1 eq) in MeCN (0.6 mL, 1 M). The crude mixture was then diluted in DCM (3.0 mL, 0.2 M) and treated with HFIP (0.06 mL, 0.61 mmol, 1 eq) for 3h. The final cyclized product was isolated after purification by flash column chromatography on silica gel (eluent: 96:4 hexanes: EtOAc + 1% Et_3N) to obtain the title compound **39** (86 mg, 0.53 mmol, 87%) as a colorless oil.

TLC: R_f = 0.44 (95:5 hexanes: EtOAc + 1% Et_3N).

^{1}H NMR (500 MHz, $CDCl_3$): δ 7.28 (t, J = 7.7 Hz, 2H, ArH^{3p}), 7.05 (d, J = 8.1 Hz, 2H, ArH^{2p}), 6.95 (t, J = 7.3 Hz, 1H, ArH^{4p}), 4.32 (h, J = 6.4 Hz, 1H, C^{5}H), 3.65 (td, J = 8.6, 5.2 Hz, 1H, C^{3}H), 3.53 (td, J = 9.1, 6.3 Hz, 1H, C^{3}H), 2.36 (dq, J = 14.8, 7.4 Hz, 1H, C^{4}H), 1.89 (ddt, J = 12.4, 8.3, 6.0 Hz, 1H, C^{4}H), 1.38 (d, J = 6.1 Hz, 3H, C$^{5'}$H$_3$).

^{13}C NMR (126 MHz, $CDCl_3$): δ 152.0 (C^{1p}), 128.9 (C^{3p}), 121.6 (C^{4p}), 115.0 (C^{2p}), 73.8 (C^5), 54.2 (C^3), 35.7 (C^4), 19.5 (C$^{5'}$).

HRMS-ESI (m/z): $[M+H]^+$ calculated for $[C_{11}H_{13}NO]^+$: 164.1070, found 164.1071.

4-Methyl-2-(naphthalen-1-ylmethyl)isoxazolidine (41)

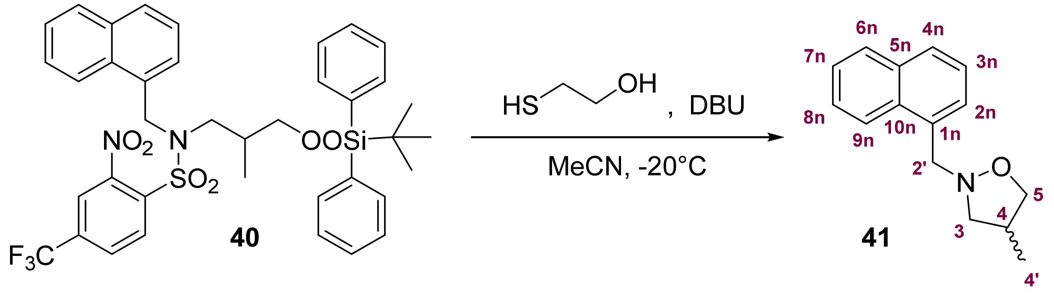

Following procedure 9A, **40** (300 mg, 0.41 mmol, 1 eq) in MeCN (2.0 mL, 0.2 M) was treated with a solution of 2-mercaptoethanol (0.31 mL, 0.41 mmol, 1 eq) and DBU (0.03 ml, 0.41 mmol, 1 eq) in MeCN (0.4 mL, 1 M). The crude residue was purified by flash column chromatography on silica gel (eluent: 95:5 hexanes: acetone) to obtain the title compound **41** (72 mg, 0.32 mmol, 78%) as a colorless oil.

TLC: R_f = 0.42 (90:10 hexanes: EtOAc).

^{1}H NMR RT (500 MHz, C_6D_6): δ 8.42 (d, J = 8.4 Hz, 1H, ArH^{9n}), 7.66 (d, J = 8.1 Hz, 1H, ArH^{6n}), 7.61 (d, J = 8.2 Hz, 1H, ArH^{4n}), 7.47 (d, J = 6.9 Hz, 1H, ArH^{8n}), 7.35 (t, J = 7.7 Hz, 1H, ArH^{7n}), 7.27 (q, J = 7.1 Hz, 2H, ArH^{3n} and ArH^{2n}), 4.61 (br, 1H, C$^{2'}$H), 4.04 (br, 1H, C$^{2'}$H), 3.90 (t, J = 7.7 Hz, 1H, C^{5}H), 3.22 (dd, J = 7.6, 5.9 Hz, 1H, C^{5}H), 2.59 (br, 1H, C^{3}H), 2.27 (dp, J = 13.9, 6.9 Hz, 1H, C^{4}H), 2.00 (br, 1H, C^{3}H), 0.73 (d, J = 6.7 Hz, 3H, C$^{4'}$H$_3$).

^{1}H NMR 60 °C (500 MHz, C_6D_6): δ 8.38 (d, J = 8.5 Hz, 1H, ArH^{9n}), 7.66 (d, J = 8.1 Hz, 1H, ArH^{6n}), 7.61 (d, J = 8.2 Hz, 1H, ArH^{4n}), 7.48 (d, J = 6.9 Hz, 1H, ArH^{8n}), 7.34 (t, J = 7.6 Hz, 1H, ArH^{7n}), 7.27 (q, J = 7.4 Hz, 2H, ArH^{3n} and ArH^{2n}), 4.35 (br, 2H, C$^{2'}$H$_2$), 3.91 (t, J = 7.8, Hz, 1H, C^{5}H), 3.29 (dd, J = 7.5, 6.0, 1.3 Hz, 1H, C^{5}H), 2.77 (br, 1H, C^{3}H), 2.31 (h, J = 6.9 Hz, 1H, C^{4}H), 2.25 (br, 1H, C^{3}H), 0.77 (d, J = 6.8 Hz, 3H, C$^{4'}$H$_3$).

^{13}C NMR (126 MHz, C_6D_6): δ 134.1 (C^{10n}), 133.8 (C^{5n}), 132.7 (C^{1n}), 128.3 (C^{6n}), 128.0 (C^{4n}), 127.2 (C^{8n}), 125.7 (C^{7n}), 125.5 (C^{3n}), 125.1 (C^{2n}), 124.9 (C^{9n}), 73.0 (C^{5}), 62.3 (C^{3}), 60.5 ($C^{2'}$), 36.6 (C^{4}), 17.5 ($C^{4'}$).

HRMS-ESI (m/z): $[M+H]^+$ calculated for $[C_{16}H_{17}NO]^+$: 228.1383, found 228.1376.

2-(Naphthalen-1-ylmethyl)-isoxazolidine (43)

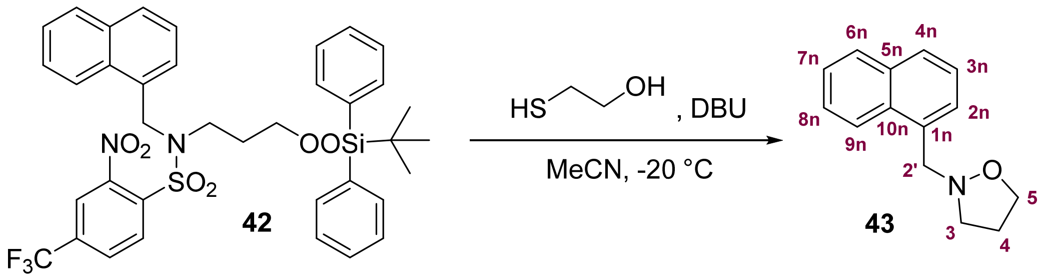

Following procedure 9A, **42** (70 mg, 0.1 mmol, 1 eq) in dry MeCN (0.5 mL, 0.2 M) was treated with a solution of 2-mercaptoethanol (0.08 mL, 0.1 mmol, 1 eq) and DBU (0.01 ml, 0.1 mmol, 1 eq) in MeCN (0.1 mL, 1 M). The final cyclized product was isolated after purification by flash column chromatography on silica gel (eluent: 80:20 hexanes: EtOAc) to obtain the title compound **43** (20 mg, 0.09 mmol, 96%) as a colorless oil.

TLC: R_f = 0.30 (90:10 hexanes: EtOAc).

^{1}H NMR RT (500 MHz, C_6D_6): δ 8.43 (d, J = 8.4 Hz, 1H, ArH^{9n}), 7.66 (d, J = 8.2 Hz, 1H, ArH^{6n}), 7.61 (d, J = 8.2 Hz, 1H, ArH^{4n}), 7.42 (d, J = 6.9 Hz, 1H, ArH^{8n}), 7.35 (t, J = 7.6 Hz, 1H, ArH^{7n}), 7.28 – 7.22 (m, 2H, ArH^{3n} and ArH^{2n}), 4.59 (br, 1H, $C^{2'}H$), 3.95 (br, 1H, $C^{2'}H$), 3.67 (s, 2H, C^5H_2), 2.72 (br, 1H, C^3H), 2.35 (br, 1H, C^3H), 1.68 (p, J = 8.5 Hz, 2H, C^4H_2).

^{1}H NMR 55 °C (500 MHz, C_6D_6): δ 8.39 (d, J = 8.4 Hz, 1H, ArH^{9n}), 7.66 (d, J = 8.0 Hz, 1H, ArH^{6n}), 7.61 (d, J = 8.3 Hz, 1H, ArH^{4n}), 7.44 (d, J = 6.9 Hz, 1H, ArH^{8n}), 7.34 (ddd, J = 8.5, 6.8,

1.5 Hz, 1H, ArH^{7n}), 7.29 – 7.23 (m, 2H, ArH^{3n} and ArH^{2n}), 4.28 (br, 2H, $C^{2'}H_2$), 3.68 (t, J = 7.3 Hz, 2H, C^5H_2), 2.56 (br, 2H, C^3H_2), 1.73 (p, J = 7.0 Hz, 2H, C^4H_2).

^{13}C NMR (126 MHz, C_6D_6): δ 134.5 (C^{10n}), 134.2 (C^{5n}), 133.1 (C^{1n}), 128.7 (C^{6n}), 128.5 (C^{4n}), 127.6 (C^{8n}), 126.1 (C^{7n}), 125.9 (C^{3n}), 125.5 (C^{2n}), 125.3 (C^{9n}), 65.5 (C^5), 60.3 ($C^{2'}$), 53.9 (C^3), 28.6 (C^4).

HRMS-ESI (m/z): $[M+H]^+$ calculated for $[C_{15}H_{15}NO]^+$: 214.1227, found 214.1220.

2-Phenylisoxazolidine (45)

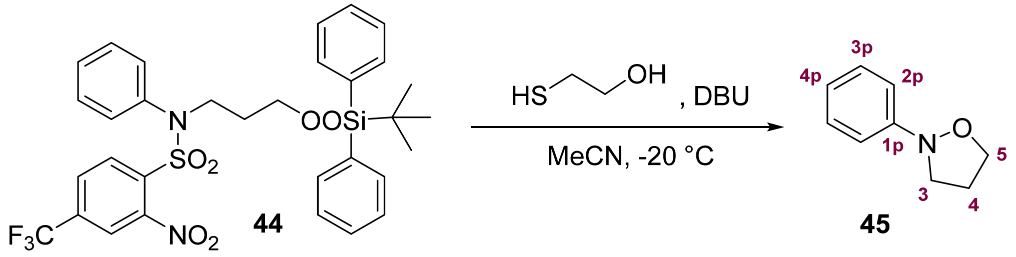

Following procedure 9A, **44** (200 mg, 0.304 mmol, 1 eq) in dry MeCN (1.5 mL, 0.2 M) was treated with a solution of 2-mercaptoethanol (0.26 mL, 0.304 mmol, 1 eq) and DBU (0.02 ml, 0.304 mmol, 1 eq) in MeCN (0.3 mL, 1 M). The final cyclized product was isolated after purification by flash column chromatography on silica gel (eluent: 98:2 hexanes: EtOAc + 1% Et_3N) to obtain the title compound **45** (43 mg, 0.29 mmol, 95%) as a colorless oil.

TLC: R_f = 0.53 (95:5 hexanes: EtOAc + 1% Et_3N).

^{1}H NMR (500 MHz, $CDCl_3$): δ 7.28 – 7.23 (m, 2H, aniline ArH^{3p}), 7.04 (d, J = 8.1 Hz, 2H, aniline ArH^{2p}), 6.94 (t, J = 7.3 Hz, 1H, aniline ArH^{4p}), 3.99 (t, J = 7.3 Hz, 2H, C^3H_2), 3.52 (t, J = 7.1 Hz, 2H, C^5H_2), 2.22 (p, J = 7.2 Hz, 2H, C^4H_2).

^{13}C NMR (126 MHz, $CDCl_3$): δ 151.6 (C^{1p}), 128.9 (C^{3p}), 121.9 (C^{4p}), 115.2 (C^{2p}), 66.0 (C^5), 53.6 (C^3), 28.2 (C^4).

HRMS-ESI (m/z): $[M+H]^+$ calculated for $[C_9H_{12}NO]^+$: 150.0914, found 150.0913

5,5-Dimethyl-2-(naphthalen-1-ylmethyl)isoxazolidine (25)

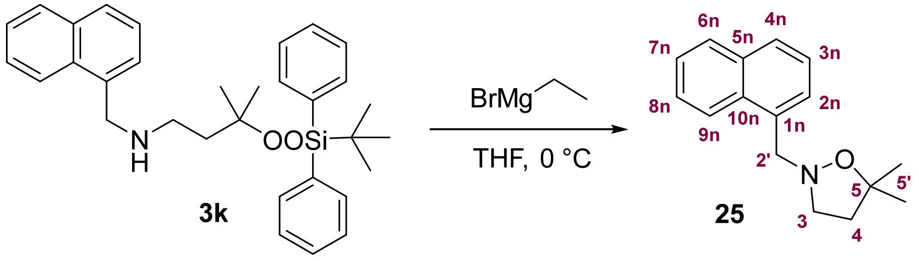

Following procedure 10, **3k** (134 mg, 0.27 mmol, 1 eq) in anhydrous THF (2 mL, 0.2 M) was added dropwise EtMgBr (0.36 mL, 1.08 mmol, 3 M in diethyl ether 4 eq). The final cyclized product was isolated after purification by flash chromatography on silica gel (eluent: 98:2 hexanes: EtOAc + 1% Et_3N) to obtain the title compound **25** (57 mg, 0.24 mmol, 88%) as a colorless oil.

TLC: R_f = 0.55 (95:5 hexanes: EtOAc + 1% Et_3N).

^{1}H NMR RT (500 MHz, C_6D_6): δ 8.40 (d, J = 8.4 Hz, 1H, ArH^{9n}), 7.66 (d, J = 8.1 Hz, 1H, ArH^{6n}), 7.60 (d, J = 8.2 Hz, 1H, ArH^{4n}), 7.50 (d, J = 6.9 Hz, 1H, ArH^{8n}), 7.35 (t, J = 7.6 Hz, 1H, ArH^{7n}), 7.29 – 7.23 (m, 2H, ArH^{3n} and ArH^{2n}), 4.46 (br, J = 132.4 Hz, 2H, $C^{2'}H_2$), 2.59 (br, 2H, C^3H_2), 1.68 (t, J = 6.3 Hz, 2H, C^4H_2), 1.27 (s, 6H, $(C^{5'}H_3)_2$).

^{1}H NMR 65 °C (500 MHz, C_6D_6): δ 8.36 (d, J = 8.5 Hz, 1H, ArH^{9n}), 7.66 (d, J = 8.1 Hz, 1H, ArH^{6n}), 7.60 (d, J = 8.2 Hz, 1H, ArH^{4n}), 7.50 (d, J = 7.0 Hz, 1H, ArH^{8n}), 7.35 (t, J = 7.6 Hz, 1H, ArH^{7n}), 7.30 – 7.24 (m, 2H, ArH^{3n} and ArH^{2n}), 4.36 (s, 2H, $C^{2'}H_2$), 2.67 – 2.61 (m, 2H, C^3H_2), 1.72 (t, J = 7.1 Hz, 2H, C^4H_2), 1.26 (s, 6H, $(C^{5'}H_3)_2$).

^{13}C NMR (126 MHz, C_6D_6): δ 134.0 (C^{10n}), 133.9 (C^{5n}), 132.6 (C^{1n}), 128.4 (C^{6n}), 128.0 (C^{4n}), 127.1 (C^{8n}), 125.7 (C^{7n}), 125.6 (C^{3n}), 125.3 (C^{2n}), 125.0 (C^{9n}), 78.7 (C^5), 60.9 (C$^{2'}$), 55.4 (C^3), 41.6 (C^4), 28.3 (C$^{5'}$).

HRMS-ESI (m/z): $[M+H]^+$ calculated for $[C_{17}H_{19}NO]^+$: 242.1540, found 242.1537.

5,5-Dimethyl-2-phenylisoxazolidine (48)

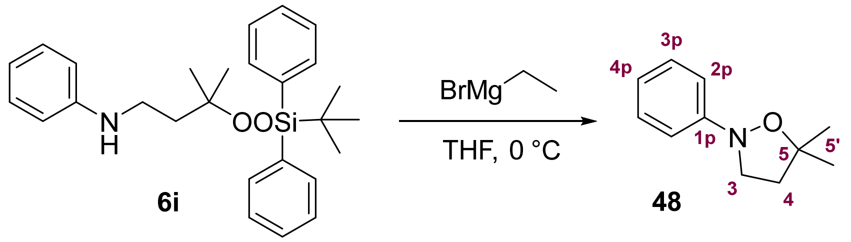

Following procedure 10, **6i** (130 mg, 0.3 mmol, 1 eq) in anhydrous THF (1.5 mL, 0.2 M) was added dropwise EtMgBr (1.2 mL, 1.2 mmol, 3 M in diethyl ether, 4 eq). The final cyclized product was isolated after purification by flash column chromatography on silica gel (eluent: 95:5 hexanes: EtOAc + 1% Et_3N) to obtain the title compound **48** (47 mg, 0.28 mmol, 92%) as a colorless oil.

TLC: R_f = 0.56 (95:5 hexanes: EtOAc + 1% Et_3N).

^{1}H NMR RT (500 MHz, C_6D_6): δ 7.19 (t, J = 7.8 Hz, 2H, ArH^{3p}), 7.09 (d, J = 8.1 Hz, 2H, ArH^{2p}), 6.85 (t, J = 7.2 Hz, 1H, ArH^{4p}), 3.13 (t, J = 7.2 Hz, 2H, C^3H_2), 1.65 (t, J = 7.1 Hz, 2H, C^4H_2), 1.20 (s, 6H, $(C^{5'}H_3)_2$).

^{13}C NMR (126 MHz, C_6D_6): δ 152.7 (C^{1p}), 128.4 (C^{3p}), 121.0 (C^{4p}), 115.1 (C^{2p}), 79.9 (C^5), 53.3 (C^3), 41.2 (C^4), 27.4 (C$^{5'}$).

HRMS-ESI (m/z): $[M+H]^+$ calculated for $[C_{12}H_{15}NO]^+$: 178.1227, found 178.1220.

2-Phenyl-1-oxa-2-azaspiro[4.5]decane (50)

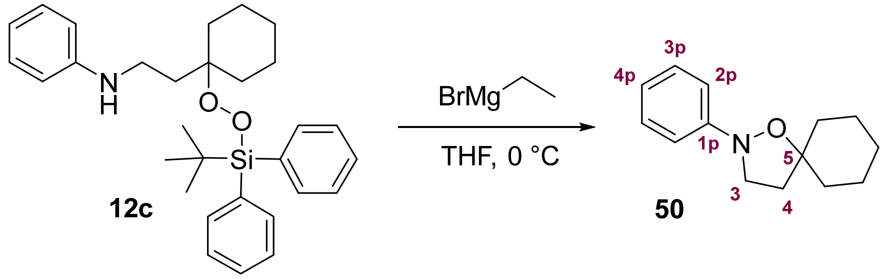

Following procedure 10, **12c** (93 mg, 0.2 mmol, 1 eq) in anhydrous THF (1.0 mL, 0.2 M) was added dropwise EtMgBr (0.26 mL, 0.79 mmol, 3 M in diethyl ether, 4 eq). The final cyclized product was isolated after purification by flash column chromatography on silica gel (eluent: 98:2 hexanes: EtOAc + 1% Et_3N) to obtain the title compound **50** (31 mg, 0.14 mmol, 73%) as a colorless oil.

TLC: R_f = 0.58 (95:5 hexanes: EtOAc + 1% Et_3N).

^{1}H NMR RT (500 MHz, C_6D_6): δ 7.24 – 7.17 (m, 2H, ArH^{3p}), 7.11 (d, J = 7.9 Hz, 2H, ArH^{2p}), 6.90 – 6.83 (m, 1H, ArH^{4p}), 3.13 (t, J = 7.1 Hz, 2H, C^3H_2), 1.71 (ddt, J = 11.8, 7.9, 4.3 Hz, 4H, cyclohexane), 1.63 (t, J = 7.1 Hz, 2H, C^4H_2), 1.37 (d, J = 10.6 Hz, 3H, cyclohexane), 1.30 – 1.24 (m, 2H, cyclohexane), 1.20 (dd, J = 8.3, 3.9z Hz, 1H, cyclohexane).

^{13}C NMR (126 MHz, C_6D_6): δ 152.7 (C^{1p}), 128.9 (C^{3p}), 121.0 (C^{4p}), 115.3 (C^{2p}), 82.0 (C^5), 52.6 (C^3), 39.6 (cyclohexane), 37.0 (cyclohexane), 25.8 (C^4), 24.1 (cyclohexane).

HRMS-ESI (m/z): $[M+H]^+$ calculated for $[C_{15}H_{19}NO]^+$: 218.1540, found 218.1528.

***trans*-3-(4-Chlorophenyl)-5-methyl-2-(naphthalene-1-ylmethyl)isoxazolidine (53)**

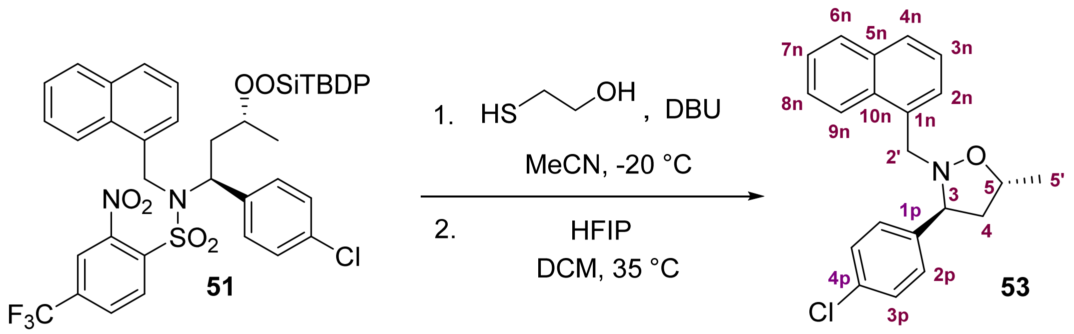

Following procedure 9B, **51** (100 mg, 0.106 mmol, 1 eq) in dry MeCN (1.0 mL, 0.2 M) was treated with a solution of 2-mercaptoethanol (0.09 mL, 0.106 mmol, 1 eq) and DBU (0.01 ml, 0.106 mmol, 1 eq) in MeCN (0.1 mL, 1 M). The crude mixture was then diluted in DCM (1.0 mL, 0.2 M) and treated with HFIP (0.02 mL, 0.106 mmol, 1 eq). Purification by flash column chromatography on silica gel (eluent: 60:40 hexanes: DCM) gave the title compound **53** (25 mg, 0.07 mmol, 70%) as a colorless oil.

TLC: R_f = 0.41 (90:10 hexanes: acetone).

^{1}H NMR (500 MHz, C_6D_6): δ 7.91 (d, J = 8.2 Hz, 1H, ArH^{9n}), 7.57 – 7.53 (m, 2H, ArH^{6n} and ArH^{4n}), 7.48 (d, J = 8.2 Hz, 1H, ArH^{8n}), 7.20 – 7.18 (m, 3H, ArH^{7n}, ArH^{2}, and ArH^{2n}), 7.02 (d, J = 8.4 Hz, 2H, ArH^{3p}), 6.98 (d, J = 8.4 Hz, 2H, ArH^{2p}), 4.14 (s, 2H, $C^{2'}H_2$), 4.02 (h, J = 6.5 Hz, 1H, C^{5}H), 3.46 (t, J = 8.2 Hz, 1H, C^{3}H), 1.86 (dt, J = 12.2, 7.6 Hz, 1H, C^{4}H), 1.78 (dt, J = 12.1, 7.7 Hz, 1H, C^{4}H), 0.99 (d, J = 6.1 Hz, 3H, $C^{5'}H_3$).

^{13}C NMR (126 MHz, C_6D_6): δ 139.8 (C^{4p}), 134.3 (C^{10n}), 134.1 (C^{5n}), 133.5 (C^{1p}), 132.7 (C^{1n}), 129.6 (C^{3p}), 128.8 (C^{2p}), 128.4 (C^{6n}), 128.2 (C^{4n}), 127.8 (C^{8n}), 125.8 (C^{7n}), 125.6 (C^{3n}), 125.5 (C^{2n}), 124.6 (C^{9n}), 72.9 (C^5), 70.9 (C^3), 58.8 (C$^{2'}$), 46.9 (C^4), 19.8 (C$^{5'}$).

HRMS-ESI (m/z): $[M+H]^+$ calculated for $[C_{21}H_{21}ClNO]^+$: 338.1307, found 338.1299.

***cis*-3-(4-Chlorophenyl)-5-methyl-2-(naphthalene-1-ylmethyl)isoxazolidine (54)**

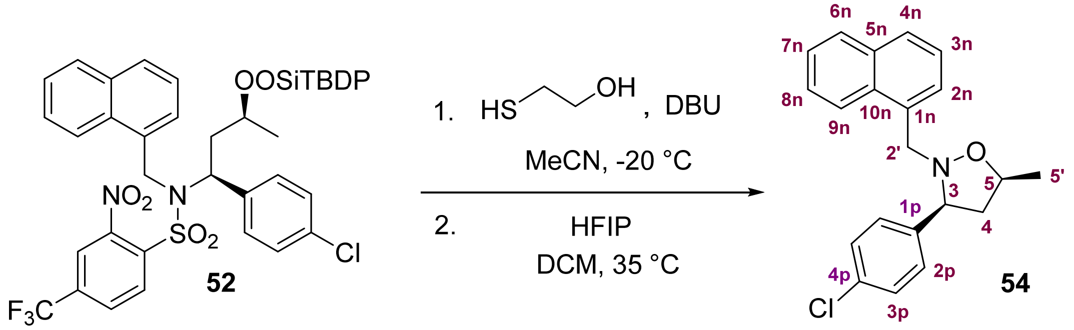

Following procedure 9B, **52** (115 mg, 0.136 mmol, 1 eq) in dry MeCN (1.0 mL, 0.2 M) was treated with a solution of 2-mercaptoethanol (0.12 mL, 0.136 mmol, 1 eq) and DBU (0.01 ml, 0.136 mmol, 1 eq) in MeCN (0.1 mL, 1 M). The crude mixture was then diluted in DCM (1.0 mL, 0.2 M) and treated with HFIP (0.03 mL, 0.136 mmol, 1 eq). Purification by flash column chromatography on silica gel (eluent: 55:45 hexanes: DCM) gave the title compound **54** (33 mg, 0.1 mmol, 72%) as a colorless oil.

TLC: R_f = 0.52 (50:50 hexanes: DCM).

^{1}H NMR (500 MHz, C_6D_6): δ 8.11 (d, J = 8.4 Hz, 1H, ArH^{9n}), 7.62 – 7.60 (m, 2H, ArH^{6n} and ArH^{4n}), 7.55 (d, J = 8.2 Hz, 1H, ArH^{8n}), 7.29 – 7.27 (m, 2H, ArH^{7n} and ArH^{3n}), 7.27 – 7.23 (m, 1H, ArH^{2n}), 7.05 (s, 4H, ArH^{2p} and ArH^{3p}), 4.32 (d, J = 13.7 Hz, 1H, C$^{2'}$H), 4.17 (d, J = 13.7 Hz, 1H, C$^{2'}$H), 4.13 (h, J = 6.5 Hz, 1H, C^{5}H), 3.66 (t, J = 7.8 Hz, 1H, C^{3}H), 2.33 (dt, J = 12.2, 7.5 Hz, 1H, C^{4}H), 1.57 (dt, J = 12.3, 8.01, 6.8 Hz, 1H, C^{4}H), 1.10 (d, J = 6.1 Hz, 3H, C$^{5'}$H$_3$).

^{13}C NMR (126 MHz, C_6D_6): δ 140.3 (C^{4p}), 138.6 (C^{10n}), 135.7 (C^{5n}), 133.2 (C^{1p}), 130.1 (C^{1n}), 129.1 (C^{3p}), 128.9 (C^{2p}), 128.8 (C^{6n}), 128.4 (C^{4n}), 127.6 (C^{8n}), 125.9 (C^{7n}), 125.7 (C^{3n}), 125.5 (C^{2n}), 124.6 (C^{9n}), 72.0 (C^5), 70.3 (C^3), 58.9 (C$^{2'}$), 47.0 (C^4), 21.1 (C$^{5'}$).

HRMS-ESI (m/z): $[M+H]^+$ calculated for $[C_{21}H_{21}ClNO]^+$: 338.1307, found 338.1299.

3,3-Dimethyl-1-(3-trifluoromethyl)benzyl)-1,3-dihydrobenzo[*c*]isoxazole (56)

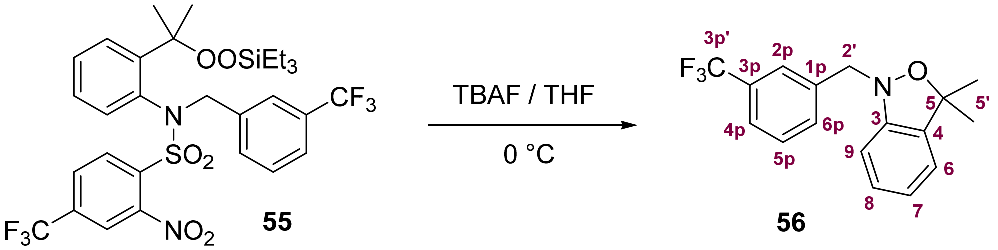

Following procedure 9C, **55** (287 mg, 0.41 mmol, 1 eq) in THF (3.0 mL, 0.2 M) was desilylated with TBAF (0.5 mL, 1.0 M in THF 1.2 eq). The crude residue was purified by flash column chromatography on silica gel (eluent: 95:5 hexanes: EtOAc) to obtain the title compound **56** (77 mg, 0.25 mmol, 61%) as white needle crystals.

TLC: R_f = 0.53 (90:10 hexanes: acetone).

^{1}H NMR (500 MHz, C_6D_6): δ 7.73 (s, 1H, ArH^{2p}), 7.39 (d, J = 7.7 Hz, 1H, ArH^{6p}), 7.26 (d, J = 7.8 Hz, 1H, ArH^{4p}), 6.98 (t, J = 7.7, 1.3 Hz, 1H, ArH^{7}), 6.93 (t, J = 7.8 Hz, 1H, ArH^{5p}), 6.81 (t, J = 7.5, 1.2 Hz, 1H, ArH^{8}), 6.67 (d, J = 7.5 Hz, 1H, ArH^{9}), 6.38 (d, J = 7.9 Hz, 1H, ArH^{6}), 4.14 (s, 2H, $C^{2'}H_2$), 1.35 (s, 6H, $(C^{5'}H_3)_2$).

^{13}C {^{1}H, ^{19}F} NMR (126 MHz, C_6D_6): δ 149.0 (C^{3}), 138.4 (C^{4}), 137.2 (C^{1p}), 131.9 (C^{6p}), 130.4 (C^{3p}), 128.5 (C^{5p}), 127.6 (C^{8}), 125.5 (C^{2p}), 124.8 ($C^{3p'}$), 123.9 (C^{4p}), 122.9 (C^{7}), 120.7 (C^{9}), 110.1 (C^{6}), 83.3 (C^{5}), 61.0 ($C^{2'}$), 27.7 ($C^{5'}$).

^{19}F NMR (470 MHz, C_6D_6): δ -62.1 (s, 3F).

HRMS-ESI (m/z): $[M+H]^+$ calculated for $[C_{18}H_{16}F_3NO]^+$: 308.1262, found 308.1254.

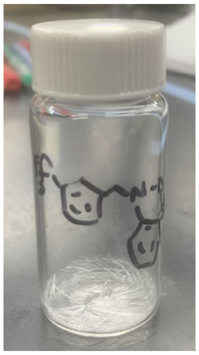

1-(4-Methoxybenzyl)-3,3-dimethyl-1,3-dihydrobenzo[*c*]isoxazole (58)

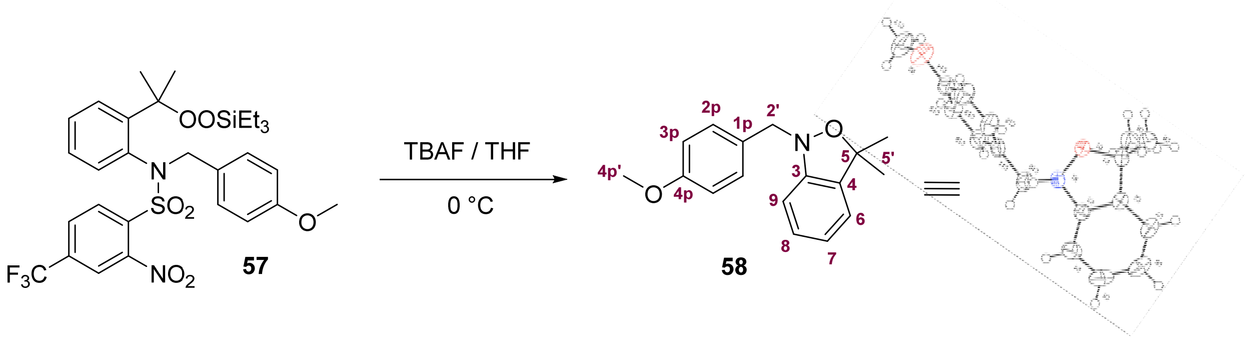

Following procedure 9C, **57** (160 mg, 0.24 mmol, 1 eq) in THF (2.0 mL, 0.2 M) was desilylated with TBAF (0.3 mL, 1.0 M in THF 1.2 eq). The crude residue was purified by flash column chromatography on silica gel (eluent: 97:3 hexanes: EtOAc) to obtain the title compound **58** (37 mg, 0.14 mmol, 57%) as a colorless monoclinic crystalline solid.

TLC: R_f = 0.57 (95:5 hexanes: EtOAc + 1% Et_3N).

^{1}H NMR (500 MHz, C_6D_6): δ 7.36 (d, *J* = 8.3 Hz, 1H, ArH^9), 6.97 (t, *J* = 7.6 Hz, 1H, ArH^7), 6.81 – 6.79 (m, 2H, ArH^8 and ArH^6), 6.70 (d, *J* = 7.4 Hz, 2H, ArH^{2p}), 6.47 (d, *J* = 7.8 Hz, 2H, ArH^{3p}), 4.35 (s, 2H, $C^{2'}H_2$), 3.28 (s, 3H, $C^{4p'}H_3$), 1.42 (s, 6H, $(C^{5'}H_3)_2$).

^{13}C NMR (126 MHz, C_6D_6): δ 159.6 (C^{10}), 149.9 (C^3), 137.7 (C^{13}), 130.6 (C^{11}), 129.5 (C^4), 128.5 (C^9), 122.8 (C^8), 120.9 (C^7), 114.0 (C^{12}), 110.9 (C^6), 83.3 (C^5), 61.7 (C$^{2'}$), 54.7 (C$^{13'}$), 28.3 (C$^{5'}$).

HRMS-ESI (m/z): $[M+Na]^+$ calculated for $[C_{17}H_{19}NO_2Na]^+$: 292.1313, found 292.1303.

1-Benzyl-3,3-dimethyl-1,3-dihydrobenzo[*c*]isoxazole (60) and 2-Nitro-4-(trifluoromethyl)phenol (61)

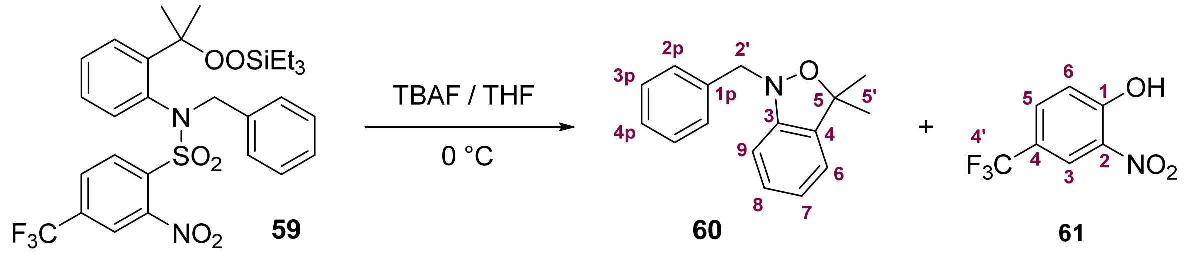

Following procedure 9C, a solution of **59** (108 mg, 0.17 mmol, 1 eq) in THF (1.0 mL, 0.2 M) was treated with TBAF (0.21 mL, 1.0 M in THF 1.2 eq) with stirring at 0 °C. The reaction mixture was then concentrated to dryness and diluted in 10 mL of diethyl ether and extracted with 2M NaOH. The ether layer was washed with brine, dried over Na_2SO_4, concentrated *in vacuo*, and purified by flash column chromatography on silica gel (eluent: 97:3 hexanes: EtOAc) to obtain the title compound **60** (41 mg, 0.15 mmol, 88%) as a white solid. The NaOH layer was neutralized with 2M HCl (20 mL) and extracted with diethyl ether (3 x 10 mL). The combined ether extracts were washed with brine, dried over Na_2SO_4, concentrated *in vacuo*. The crude residue was purified by flash chromatography on silica gel (eluent: 99:1 hexanes: acetone) to obtain the title compound **61** (32 mg, 0.16 mmol, 92%) as a yellow liquid, with spectra data consistent with the literature.[14]

Data for 60:

TLC: R_f = 0.50 (95:5 hexanes: EtOAc + 1% Et_3N).

^{1}H NMR (500 MHz, C_6D_6): δ 7.45 (d, *J* = 7.5 Hz, 2H, ArH^{2p}), 7.18 (t, *J* = 7.4 Hz, 2H, ArH^{3p}), 7.09 (t, *J* = 7.3 Hz, 1H, ArH^{4p}), 6.96 (t, *J* = 6.2 Hz, 1H, ArH^7), 6.81 (t, *J* = 7.4 Hz, 1H^8), 6.70 (d, *J* = 6.4 Hz, 1H, ArH^9), 6.43 (d, *J* = 7.9 Hz, 1H, ArH^6), 4.48 (s, 2H, $C^{2'}H_2$), 1.53 (s, 6H, $(C^{5'}H_3)_2$).

^{13}C NMR (126 MHz, C_6D_6): δ 149.6 (C^3), 137.4 (C^{1p}), 137.3 (C^4), 128.8 (C^{2p}), 128.2 (C^{3p}), 128.0 (C^{4p}), 127.2 (C^7), 122.6 (C^8), 120.6 (C^9), 110.4 (C^6), 83.0 (C^5), 62.0 (C$^{2'}$), 27.9 (C$^{5'}$).

HRMS-ESI (m/z): $[M+Na]^+$ calculated for $[C_{16}H_{18}NO]^+$: 240.1388, found 240.1384.

Data for 61:

TLC: R_f = 0.24 (90:10 hexanes: acetone).

^{1}H NMR (500 MHz, $CDCl_3$): δ 10.80 (s, OH, 1H), 8.43 (d, *J* = 1.5 Hz, 1H, ArH^3), 7.82 (dd, *J* = 8.8, 2.3 Hz, 1H, ArH^6), 7.31 (d, *J* = 8.8 Hz, 1H, ArH^5).

^{13}C NMR (126 MHz, $CDCl_3$): δ 157.3 (C^2), 133.9 (q, ^2J = 3.1 Hz, C^4), 133.2 (C^1), 123.3 (q, ^3J = 4.4 Hz, C^3), 123.1 (q, ^1J = 271.8 Hz, $C^{4'}$), 122.9 (q, ^3J = 34.7 Hz, C^5), 121.4 (C^6).

^{13}C {^{1}H, ^{19}F} NMR (126 MHz, $CDCl_3$): δ 157.3 (C^2), 133.9 (C^4), 133.3 (C^1), 123.3 (C^3), 123.2 ($C^{4'}$), 123.1 (C^5), 121.4 (C^6).

^{19}F NMR (470 MHz, $CDCl_3$): δ -62.3 (s, 3F)

HRMS-ESI (m/z): $[M-H]^-$ calculated for $[C_7H_3F_3NO_3]^-$: 206.0070, found 206.0072.

1-(3-(Benzyloxy)propyl)-3,3-dimethyl-1,3-dihydrobenzo[*c*]isoxazole (66)

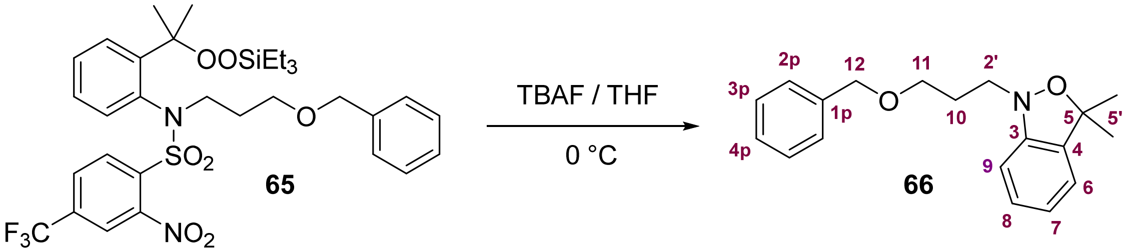

Following procedure 9C, **65** (138 mg, 0.2 mmol, 1 eq) in THF (1.0 mL, 0.2 M) was desilylated with TBAF (0.24 mL, 1.0 M in THF, 1.2 eq). The crude residue was purified by flash column chromatography on silica gel (eluent: 95:5 hexanes: EtOAc + 1% Et_3N) to obtain the title compound **66** (51 mg, 0.17 mmol, 86%) as a colorless oil.

TLC: R_f = 0.53 (95:5 hexanes: EtOAc + 1% Et_3N).

^{1}H NMR (500 MHz, C_6D_6): δ 7.28 (d, *J* = 7.5 Hz, 2H, ArH^{2p}), 7.18 (t, *J* = 8.0 Hz, 2H, ArH^{3p}), 7.09 (t, *J* = 7.3 Hz, 1H, ArH^{4p}), 6.98 (t, *J* = 7.6 Hz, 1H, ArH^7), 6.82 (t, *J* = 7.4 Hz, 1H, ArH^8), 6.72 (d, *J* = 7.4 Hz, 1H, ArH^9), 6.47 (d, *J* = 7.8 Hz, 1H, ArH^6), 4.32 (s, 2H, $C^{12}H_2$), 3.50 (t, *J* = 6.0 Hz, 2H, $C^{11}H_2$), 3.43 (t, *J* = 6.9 Hz, 2H, $C^{2'}H_2$), 2.13 (p, *J* = 6.1 Hz, 2H, $C^{10}H_2$), 1.46 (s, 6H, $(C^{5'}H_3)_2$).

^{13}C NMR (126 MHz, C_6D_6): δ 150.3 (C^3), 139.5 (C^{1p}), 137.4 (C^4), 128.5 (C^{2p}), 127.8 (C^{3p}), 127.6 (C^{4p}), 127.6 (C^7), 122.6 (C^8), 120.8 (C^9), 110.3 (C^6), 83.0 (C^5), 73.0 (C^{12}), 68.1 (C^{11}), 55.2 (C$^{2'}$), 28.2 (C$^{5'}$), 28.1 (C^{10}).

HRMS-ESI (m/z): $[M+H]^+$ calculated for $[C_{20}H_{23}NO_2]^+$: 298.1807, found 298.1800.

1-(2-Iodobenzyl)-3-methyl-1,3-dihydrobenzo[*c*]isoxazole (68) and 2-Iodobenzylaminoacetophenone (69)

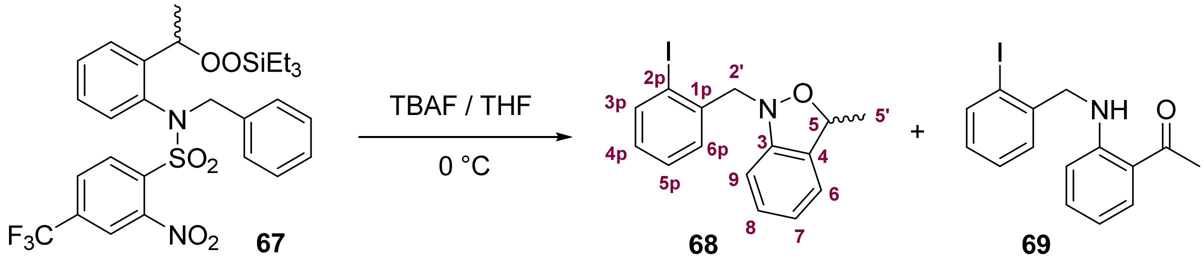

Following procedure 9C, **67** (347 mg, 0.47 mmol, 1 eq) in THF (3.0 mL, 0.2 M) was desilylated with TBAF (0.6 mL, 1.0 M in THF, 1.2 eq). The crude residue was purified by flash column chromatography on silica gel (eluent: 97:3 hexanes: EtOAc) to obtain the title compound **68** (43 mg, 0.12 mmol, 26%) as a colorless oil, and **69** (35 mg, 0.1 mmol, 21%) with spectral data consistent with the literature[15] as a colorless oil.

Data for 68:

TLC: R_f = 0.58 (95:5 hexanes: EtOAc + 1% Et_3N).

^{1}H NMR (500 MHz, C_6D_6): δ 7.81 (d, *J* = 7.7 Hz, 1H, ArH^9), 7.68 (d, *J* = 7.7 Hz, 1H, ArH^{3p}), 7.05 (t, *J* = 7.4 Hz, 1H, ArH^{5p}), 6.98 (t, *J* = 8.1 Hz, 1H, ArH^{4p}), 6.83 (t, *J* = 7.4 Hz, 1H, ArH^8), 6.69 (d, *J* = 7.6 Hz, 1H, ArH^{6p}), 6.59 – 6.54 (m, 2H, ArH^6 and ArH^7), 5.20 (q, *J* = 6.1 Hz, 1H, C^{5}H), 4.49 (d, *J* = 15.4 Hz, 1H, C$^{2'}$H), 4.37 (d, *J* = 15.7 Hz, 1H, C$^{2'}$H), 1.26 (d, *J* = 6.1 Hz, 3H, C$^{5'}$H$_3$).

^{13}C NMR (126 MHz, C_6D_6): δ 150.1 (C^3), 139.7 (C^{1p}), 139.1 (C^{3p}), 133.9 (C^4), 130.4 (C^9), 128.9 (C^7), 128.1 (C^{5p}), 128.0 (C^{4p}), 123.0 (C^8), 121.2 (C^{6p}), 110.6 (C^6), 99.3 (C^{2p}), 77.2 (C^5), 67.3 (C$^{2'}$), 20.6 (C$^{5'}$).

HRMS-ESI (m/z): $[M+Na]^+$ calculated for $[C_{15}H_{14}INONa]^+$: 374.0018, found 374.0007.

6-Bromo-2-methyl-2,3,3a,4-tetrahydroisoxazolo[2,3-*a*]indole (6-*exo*-methyl 72 and 6-*endo*-methyl 73)

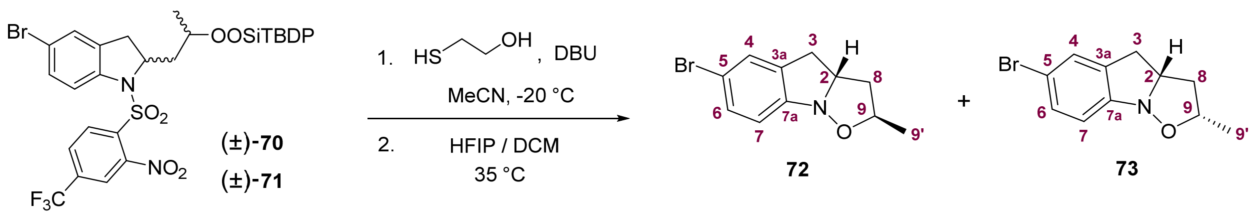

Following procedure 9B, the mixture of diastereomers **70** and **71** (170 mg, 0.223 mmol, 1 eq) in dry MeCN (1.5 mL, 0.2 M) were treated with a solution of 2-mercaptoethanol (0.19 mL, 0.223 mmol, 1 eq) and DBU (0.02 ml, 0.223 mmol, 1 eq) in MeCN (0.15 mL, 1 M). The crude residue was then diluted in DCM (1.5 mL, 0.2 M) and treated with HFIP (0.04 mL, 0.223 mmol, 1 eq) with stirring at 35 °C for 6h. The crude residue was purified by flash column chromatography on silica gel (eluent: 96:4 hexanes: EtOAc + 1% Et_3N (**72**) and 93:7 hexanes: EtOAc + 1% Et_3N (**73**)) to obtain the *exo*-methyl **72** (25 mg, 0.1 mmol, 44%) and *endo*-methyl **73** (23 mg, 0.1 mmol, 41%) as colorless oils.

Data for *exo*-methyl isomer 72:

TLC: R_f = 0.27 (90:10 hexanes: acetone).

^{1}H NMR (500 MHz, C_6D_6): δ 7.12 (d, *J* = 8.3 Hz, 1H, ArH^6), 6.98 (s, 1H, ArH^4), 6.96 (d, *J* = 8.3 Hz, 1H, ArH^7), 3.76 (td, *J* = 8.8, 4.9 Hz, 1H, C^9H), 3.51 (h, *J* = 5.9 Hz, 1H, C^2H), 2.54 (dd, *J* = 16.0, 7.9 Hz, 1H, C^3H), 2.23 (d, *J* = 16.0 Hz, 1H, C^3H), 1.45 (dt, *J* = 12.4, 8.9 Hz, 1H, C^8H), 1.28 (ddd, *J* = 12.3, 7.3, 5.0 Hz, 1H, C^8H), 1.02 (d, *J* = 6.0 Hz, 3H, $C^{9'}H_3$).

^{13}C NMR (126 MHz, C_6D_6): δ 151.8 (C^5), 131.9 (C^{7a}), 131.3 (C^4), 128.4 (C^6), 118.0 (C^7), 116.8 (C^{3a}), 74.0 (C^2), 65.0 (C^9), 43.4 (C^8), 34.6 (C^3), 18.5 ($C^{9'}$).

HRMS-ESI (m/z): $[M+H]^+$ calculated for $[C_{11}H_{13}BrNO]^+$: 254.0176, found 254.0175.

Data for *endo*-methyl isomer 73:

TLC: R_f = 0.22 (90:10 hexanes: acetone).

^{1}H NMR (500 MHz, C_6D_6): δ 7.11 (d, J = 8.3 Hz, 1H, ArH^6), 7.00 (s, 1H, ArH^4), 6.99 (s, 1H, ArH^7), 4.04 (dt, J = 9.3, 6.1 Hz, 1H, C^{9}H), 3.69 (dt, J = 10.0, 7.1 Hz, 1H, C^{2}H), 2.51 (dd, J = 15.7, 7.6 Hz, 1H, C^{3}H), 2.27 (d, J = 15.7 Hz, 1H, C^{3}H), 1.65 (dt, J = 12.5, 6.5 Hz, 1H, C^{8}H), 1.03 (dt, J = 12.3, 9.6 Hz, 1H, C^{8}H), 0.93 (d, J = 6.2 Hz, 3H, C$^{9'}$H$_3$).

^{13}C NMR (126 MHz, C_6D_6): δ 154.0 (C^5), 131.1 (C^{7a}), 129.8 (C^4), 128.6 (C^6), 118.5 (C^7), 116.4 (C^{3a}), 77.4 (C^2), 66.1 (C^9), 42.0 (C^8), 32.5 (C^3), 21.4 (C$^{9'}$).

HRMS-ESI (m/z): $[M+H]^+$ calculated for $[C_{11}H_{13}BrNO]^+$: 254.0176, found 254.0175.

2-(Naphthalen-1-ylmethyl)-1,2-oxazinane (76)

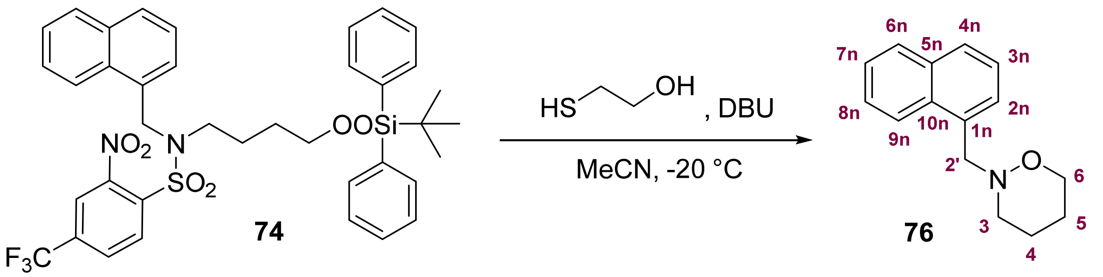

Following procedure 9A, **74** (182 mg, 0.25 mmol, 1 eq) in dry MeCN (2.0 mL, 0.2 M) was treated with a solution of 2-mercaptoethanol (0.2 mL, 0.25 mmol, 1 eq) and DBU (0.02 ml, 0.25 mmol, 1 eq) in MeCN (0.3 mL, 1 M). The final cyclized product was isolated after purification by flash chromatography on silica gel (eluent: 97:3 hexanes: EtOAc) to obtain the title compound **76** (30 mg, 0.13 mmol, 53%) as a colorless oil.

TLC: R_f = 0.57 (90:10 hexanes: EtOAc).

^{1}H NMR RT (500 MHz, C_6D_6): δ 8.37 (d, J = 8.5 Hz, 1H, ArH^{9n}), 7.66 (d, J = 8.1 Hz, 1H, ArH^{6n}), 7.60 (d, J = 8.2 Hz, 1H, ArH^{4n}), 7.47 (d, J = 6.9 Hz, 1H, ArH^{8n}), 7.37 (ddt, J = 8.3, 6.8,

1.2 Hz, 1H, ArH^{7n}), 7.32 – 7.23 (m, 2H, ArH^{3n} and ArH^{2n}), 4.24 (br, 2H, $C^{2'}H_2$), 3.82 (t, J = 5.2 Hz, 2H, C^6H_2), 2.59 (br, 2H, C^3H_2), 1.44 (br, 2H, C^4H_2), 1.24 (br, 2H, C^5H_2).

^{1}H NMR 65 °C (500 MHz, C_6D_6): δ 8.34 (d, J = 8.4 Hz, 1H, ArH^{9n}), 7.66 (d, J = 8.1 Hz, 1H, ArH^{6n}), 7.60 (d, J = 8.2 Hz, 1H, ArH^{4n}), 7.47 (d, J = 6.9 Hz, 1H, ArH^{8n}), 7.36 (t, J = 7.6 Hz, 1H, ArH^{7n}), 7.32 – 7.22 (m, 2H, ArH^{3n} and ArH^{2n}), 4.23 (s, 2H, $C^{2'}H_2$), 3.82 (t, J = 5.5 Hz, 2H, C^6H_2), 2.61 (dd, J = 6.7, 4.2 Hz, 2H, C^3H_2), 1.48 (p, J = 5.8 Hz, 2H, C^4H_2), 1.27 (p, J = 5.9 Hz, 2H, C^5H_2).

^{13}C NMR (126 MHz, C_6D_6): δ 134.5 (C^{10n}), 133.7 (C^{5n}), 133.1 (C^{1n}), 128.8 (C^{6n}), 128.3 (C^{4n}), 127.7 (C^{8n}), 126.0 (C^{7n}), 125.8 (C^{3n}), 125.5 (C^{2n}), 125.3 (C^{9n}), 70.2 (C^6), 62.2 ($C^{2'}$), 56.3 (C^3), 25.2 (C^4), 24.3 (C^5).

HRMS-ESI (m/z): $[M+H]^+$ calculated for $[C_{16}H_{17}NO]^+$: 228.1383, found 228.1369.

***N*-(Naphthalen-1-ylmethyl)-2-nitro-*N*-(pent-4-en-1-yl)-4-(trifluoromethyl)benzenesulfonamide (3o)**

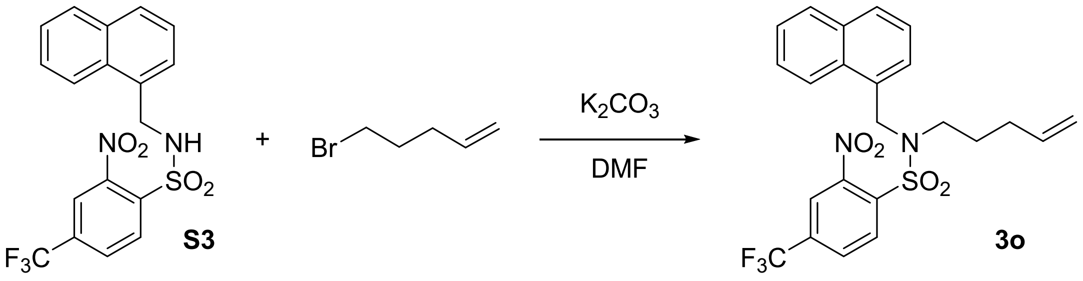

Following general procedure 2A, a stirred solution of **S3** (1.75 g, 4.25 mmol, 1 eq) in DMF (25 mL, 0.2 M) was treated with K_2CO_3 (880 mg 6.38 mmol, 1.5 eq), followed by 5-bromopent-1-ene (0.76 mL, 6.38 mmol, 1.5 eq). The crude residue was purified by flash column chromatography on silica gel (eluent: 95:5 hexanes: EtOAc) to obtain the title compound **3o** (1.78 g, 3.74 mmol, 88%) as a yellow solid.

TLC: R*f* = 0.39 (90:10 hexanes: EtOAc).

^{1}H NMR (500 MHz, $CDCl_3$): δ 8.02 (d, *J* = 7.1 Hz, 1H, ArH), 7.82 – 7.73 (m, 4H, ArH), 7.60 (d, *J* = 8.2 Hz, 1H, ArH), 7.50 – 7.43 (m, 3H, ArH), 7.42 – 7.35 (m, 1H, ArH), 5.61 (ddt, *J* = 17.4, 9.7, 6.6 Hz, 1H, =C*H*), 4.97 (s, 2H, $ArCH_2$), 4.92 (s, 1H, =C*H*$_2$), 4.90 (d, *J* = 2.6 Hz, 1H, =C*H*$_2$), 3.47 – 3.41 (m, 2H, CH_2), 1.95 (q, *J* = 7.4 Hz, 2H, CH_2), 1.55 (p, *J* = 7.6 Hz, 2H, CH_2).

^{13}C NMR (126 MHz, $CDCl_3$): δ 161.1, 147.6, 137.1, 137.0, 134.9 (q, *J* = 34.7 Hz), 133.7, 131.8, 131.6, 130.4, 129.4, 128.8, 128.0, 127.9 (q, *J* = 3.9 Hz), 126.9, 126.2, 125.2, 123.2, 122.0 (q, *J* = 273.4 Hz), 121.4 (q, *J* = 3.6 Hz), 50.6, 49.0, 30.7, 27.6.

HRMS-ESI (m/z): $[M+Na]^+$ calculated for $[C_{23}H_{21}F_3N_2O_4SNa]^+$: 501.1072, found 501.1065.

***N*-(5-Hydzroxypentyl)-*N*-(naphthalen-1-ylmethyl)-2-nitro-4-(trifluoromethyl)benzenesulfonamide (3p)**

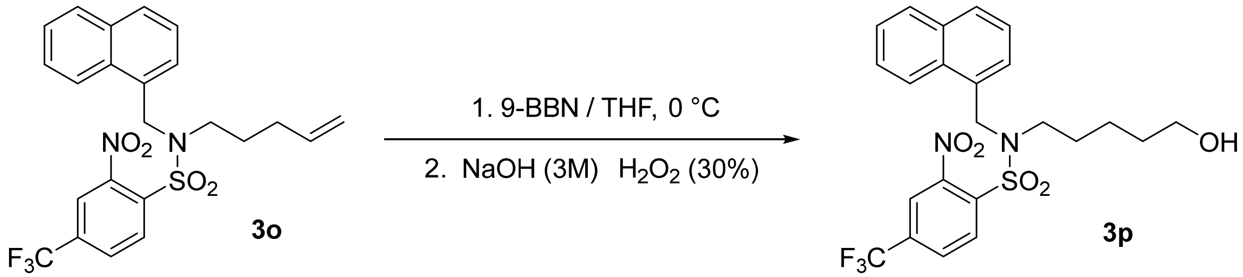

Following general procedure 3, **3o** (1.6 g, 3.34 mmol, 1 eq) in dry THF (17 mL, 0.2 M) was treated with 9-BBN 0.5 M in THF (13.4 mL, 2 eq), 3 M NaOH (4.3 mL, 1.2 eq) and aq. 30% H_2O_2 (4.3 mL, 1.2 eq). The crude residue was purified by flash column chromatography on silica gel (eluent: 75:35 hexanes: EtOAc) to obtain the title compound **3p** (796 mg, 1.60 mmol, 48%) as a yellow oil.

TLC: R*f* = 0.13 (70:30 hexanes: EtOAc).

^{1}H NMR (500 MHz, $CDCl_3$): δ 8.02 (d, *J* = 7.8 Hz, 1H, ArH), 7.77 (d, *J* = 6.5, 2H, ArH), 7.74 (d, *J* = 7.3 Hz, 2H, ArH), 7.58 (d, *J* = 8.2 Hz, 1H, ArH), 7.51 – 7.41 (m, 3H, ArH), 7.37 (t, *J* = 7.6 Hz, 1H, ArH), 4.95 (s, 2H, $ArCH_2$), 3.49 (t, *J* = 6.4 Hz, 2H, CH_2), 3.44 (t, *J* = 7.6 Hz, 2H, CH_2), 1.53 (Br, 1H, OH), 1.47 (p, *J* = 7.8 Hz, 2H, CH_2), 1.40 (p, *J* = 7.0 Hz, 2H, CH_2), 1.23 (p, *J* = 6.2 Hz, 2H, CH_2).

^{13}C {^{1}H, ^{19}F} NMR (126 MHz, $CDCl_3$): δ 160.6, 147.6, 137.0, 135.0, 133.7, 131.8, 131.6, 130.4, 129.4, 128.8, 128.0, 126.9, 126.3, 125.2, 123.2, 122.1, 121.4, 62.5, 50.6, 49.5, 32.1, 28.3, 22.8.

HRMS-ESI (m/z): $[M+Na]^+$ calculated for $[C_{23}H_{23}F_3N_2O_5SNa]^+$: 519.1177, found 519.1190.

***N*-(5-Iodopentyl)-*N*-(naphthalen-1-ylmethyl)-2-nitro-4-(trifluoromethyl)benzenesulfonamide (3q)**

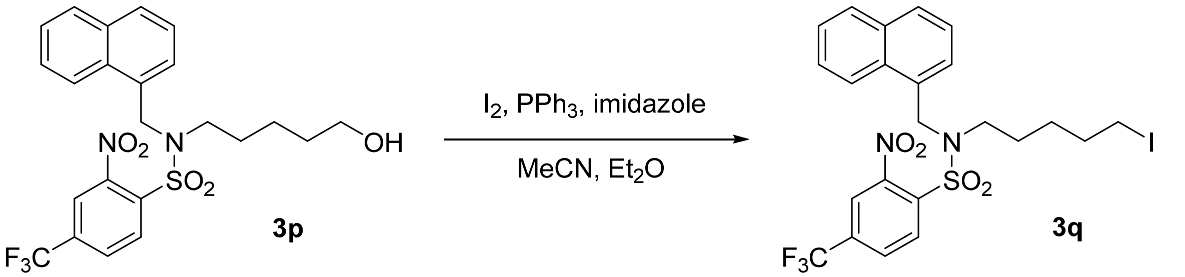

Following general procedure 4, triphenylphosphine (619 mg, 2.36 mmol, 1.5 eq), imidazole (160 mg, 2.36 mmol, 1.5 eq) and iodine (599 mg, 2.36 mmol, 1.5 eq) were added subsequently to a solution of **3p** (780 mg, 1.57 mmol, 1 eq) in a 1:3 ratio of MeCN and Et_2O (8.0 mL, 0.2 M) at RT. The crude product was purified by flash column chromatography on silica gel (eluent: 94:6 hexanes: EtOAc) to obtain the title compound **3q** (667 mg, 1.11 mmol, 71%) as a colorless oil.

TLC: R_f = 0.41 (90:10 hexanes: EtOAc).

^{1}H NMR (500 MHz, $CDCl_3$): δ 8.04 (d, J = 8.7 Hz, 1H, ArH), 7.79 (d, J = 9.1 Hz, 4H, ArH), 7.61 (d, J = 8.4 Hz, 1H, ArH), 7.50 – 7.46 (m, 2H, ArH), 7.44 (d, J = 7.2 Hz, 1H, ArH), 7.38 (t, J = 7.6 Hz, 1H, ArH), 4.95 (s, 2H, $ArCH_2$), 3.42 (t, J = 7.5 Hz, 2H, CH_2), 3.00 (t, J = 7.0 Hz, 2H, CH_2), 1.63 (p, J = 7.0 Hz, 2H, CH_2), 1.42 (p, J = 7.7 Hz, 2H, CH_2), 1.24 (p, J = 6.8 Hz, 2H, CH_2).

^{13}C {^{1}H, ^{19}F} NMR (126 MHz, $CDCl_3$): δ 155.3, 147.6, 136.8, 135.0, 133.7, 131.7, 131.6, 130.3, 129.4, 128.8, 128.1, 128.0, 126.9, 126.2, 125.2, 123.2, 122.0, 121.4, 50.6, 49.1, 32.7, 27.4, 6.4.

HRMS-ESI (m/z): $[M+Na]^+$ calculated for $[C_{23}H_{22}F_3IN_2O_4SNa]^+$: 629.0195, found 629.0188.

***N*-(5-Hydroperoxypentyl)-*N*-(naphthalen-1-ylmethyl)-2-nitro-4-(trifluoromethyl)benzenesulfonamide (3r)**

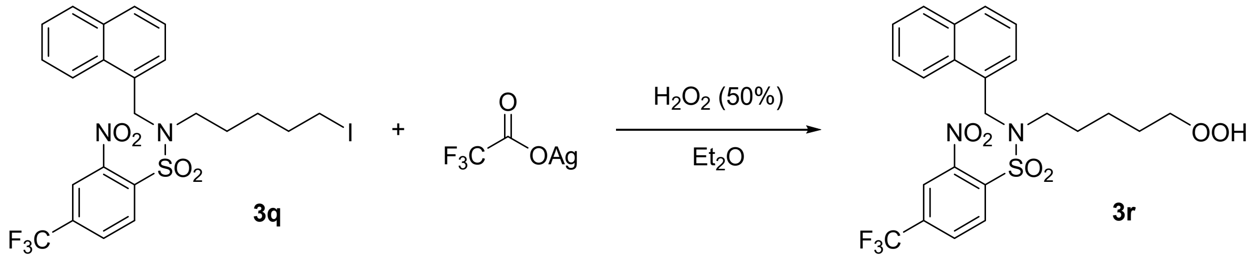

Following general procedure 5, **3q** (670 mg, 1.1 mmol, 1 eq) in diethyl ether (6.0 mL, 0.2 M) was treated with silver trifluoroacetate (287 mg, 1.3 mmol, 1.2 eq) and aqueous 50% hydrogen peroxide (1.28 mL, 2 eq). The crude residue was then purified by flash column chromatography on silica gel (eluent: 75:25 hexanes: EtOAc) to obtain the title compound **3r** (287 mg, 0.56 mmol, 51%) as a colorless oil.

TLC: R_f = 0.16 (80:20 hexanes: EtOAc).

^{1}H NMR (500 MHz, C_6D_6): δ 8.06 (d, *J* = 8.4 Hz, 1H, ArH), 7.72 (br, 1H, OOH), 7.55 (d, *J* = 8.2 Hz, 1H, ArH), 7.50 (d, *J* = 8.2 Hz, 1H, ArH), 7.35 (d, *J* = 8.2 Hz, 1H, ArH), 7.33 – 7.28 (m, 2H, ArH), 7.26 – 7.21 (m, 2H, ArH), 7.14 (t, *J* = 7.4 Hz, 1H, ArH), 6.82 (d, *J* = 8.7 Hz, 1H, ArH), 4.77 (s, 2H, $ArCH_2$), 3.69 (t, *J* = 6.3 Hz, 2H, CH_2), 3.31 (t, *J* = 7.5 Hz, 2H, CH_2), 1.34 – 1.23 (m, 4H, overlapping CH_2), 1.04 (p, *J* = 7.7 Hz, 2H, CH_2).

^{13}C {^{1}H, ^{19}F} NMR (126 MHz, C_6D_6): δ 147.8, 137.0, 134.3, 134.1, 132.0, 131.8, 130.9, 129.4, 128.9, 128.4, 127.6, 127.0, 126.4, 125.3, 123.6, 122.4, 121.1, 76.3, 50.6, 49.4, 28.4, 27.2, 23.1.

HRMS-ESI (m/z): $[M+Na]^+$ calculated for $[C_{23}H_{23}F_3N_2O_6SNa]^+$: 535.1127, found 535.1107.

***N*-(5-(*tert*-Butyldiphenylsilylperoxy)pentyl)-*N*-(naphthalen-1-ylmethyl)-2-nitro-4-(trifluoromethyl)benzenesulfonamide (75)**

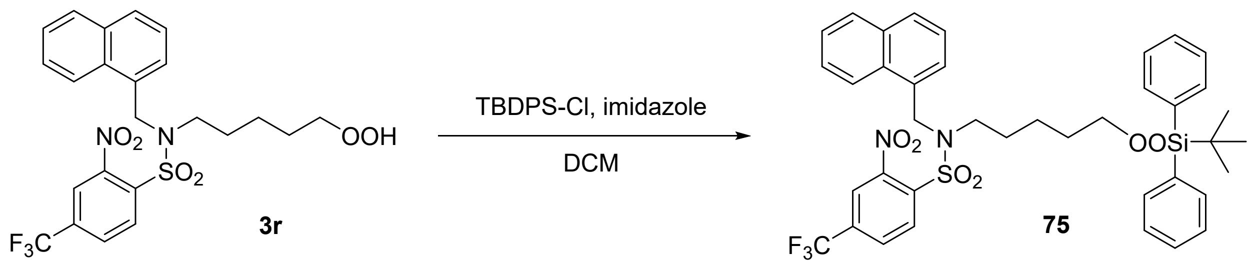

Following procedure 8, a solution of **3r** (250 mg, 0.49 mmol, 1 eq) in DCM (3.0 mL, 0.2 M) was treated with imidazole (49 mg, 0.73 mmol, 1.5 eq) and TBDPS-Cl (0.13 mL, 0.49 mmol, 1 eq). The crude residue was purified by flash column chromatography on silica gel (eluent: 96:4 hexanes: EtOAc) to obtain the title compound **75** (245 mg, 0.33 mmol, 67%) as a colorless oil.

TLC: R_f = 0.18 (90:10 hexanes: acetone).

^{1}H NMR (500 MHz, C_6D_6): δ 7.97 (d, *J* = 8.4 Hz, 1H, ArH), 7.91 – 7.84 (m, 4H, ArH), 7.49 (d, *J* = 8.1 Hz, 1H, ArH), 7.42 (d, *J* = 8.2 Hz, 1H, ArH), 7.25 – 7.22 (m, 7H, ArH), 7.21 (d, *J* = 8.2 Hz, 2H, ArH), 7.17 (s, 1H, ArH), 7.08 – 7.01 (m, 2H, ArH), 6.66 (d, *J* = 8.2 Hz, 1H, ArH), 4.66 (s, 2H, $ArCH_2$), 3.74 (t, *J* = 6.3 Hz, 2H, CH_2), 3.20 – 3.13 (m, 2H, CH_2), 1.22 (s, 9H, *t*Bu), 1.20 – 1.16 (m, 2H, CH_2), 1.11 (p, *J* = 7.6 Hz, 2H, CH_2), 0.84 (p, *J* = 7.8 Hz, 2H, CH_2).

^{13}C {^{1}H, ^{19}F} NMR (126 MHz, C_6D_6): δ 147.8, 137.2, 136.1, 134.2, 134.1, 133.3, 132.0, 131.8, 131.0, 130.3, 129.3, 128.9, 128.4, 128.0, 127.4, 126.9, 126.3, 125.2, 123.6, 122.4, 121.0, 76.7, 50.5, 49.4, 28.4, 27.6, 27.3, 23.2, 19.6.

HRMS-ESI (m/z): $[M+Na]^+$ calculated for $[C_{39}H_{41}F_3N_2O_6SSiNa]^+$: 773.2304, found 773.2292.

7) ***Attempted formation of Oxazepanes***

1-Naphthaldehyde (78)

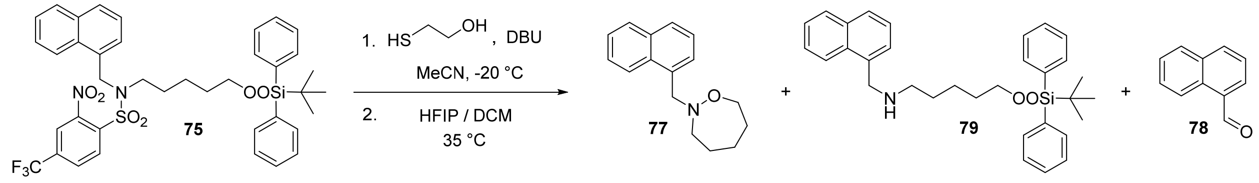

Following procedure 9B, a solution of **75** (59 mg, 0.079 mmol, 1 eq) in dry MeCN (1.0 mL, 0.2 M) was treated with a solution of 2-mercaptoethanol (0.07 mL, 0.079 mmol, 1 eq) and DBU (0.01 ml, 0.079 mmol, 1 eq) in MeCN (0.1 mL, 1 M). The crude mixture was then diluted in DCM (1.0 mL, 0.2 M) and treated with HFIP (0.01 mL, 0.079 mmol, 1 eq). The crude product was isolated after purification by flash column chromatography on silica gel (eluent: 98:2 hexanes: EtOAc) to give the title compound **78** (16 mg, 0.74 mmol, 94%, $\mathbf{R}_f$ = 0.52 (90:10 hexanes: EtOAc + 1% Et_3N) with spectral data consistent with the literature[16] as a yellow oil and **79** (trace). **77** was not detected.

8) Determination of Barrier to Inversion by VT NMR

5,5-dimethyl-2-(naphthalen-1-ylmethyl)isoxazolidine **25** (25 mg, 0.1 mmol) was dissolved in CD_3CN (0.85 mL) and a ^1H NMR recorded at room temperature (25 °C). A series of spectra were then collected in 5 °C increments between -20 °C and 75 °C.

The barrier to stereomutation ($\Delta G_c^{\neq}$) was calculated from coalescence temperature (T_C in K) and the maximum frequency difference ($\Delta\nu$ in Hz) between the two resolved signals. This data was then plugged into the equation: $\Delta G_c^{\neq} = 4.575 \times 10^{-3}\, T_C\,[9.972 + \log(T_C/\Delta\nu)]$ leading to a consistent calculated barrier from multiple sets of signals.

Table S1. T_C, $\Delta\nu$, and $\Delta G_c^{\neq}$ values obtained from VT NMR studies.

Entry	Proton	Coalescence Temperature (T_C in K)	Maximum Frequency Difference ($\Delta\nu$ in Hz)	Barrier ($\Delta G_c^{\neq}$)
**1**	$H_{2'}$	298	68.05	14.5
**2**	H_3	308	186.29	14.4
**3**	H_4	283	57.27	13.8
**4**	$H_{5'}$	288	51.06	14.1

25

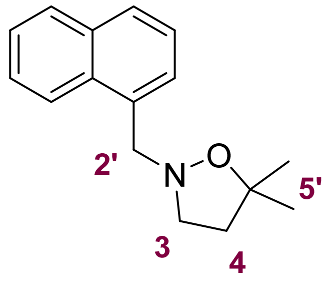

9) ***Spectra***

^{1}H NMR (500 MHz, CDCl$_3$) spectrum of *N*-(Naphthalen-1-ylmethyl)-4-nitrobenzenesulfonamide **(S1)**

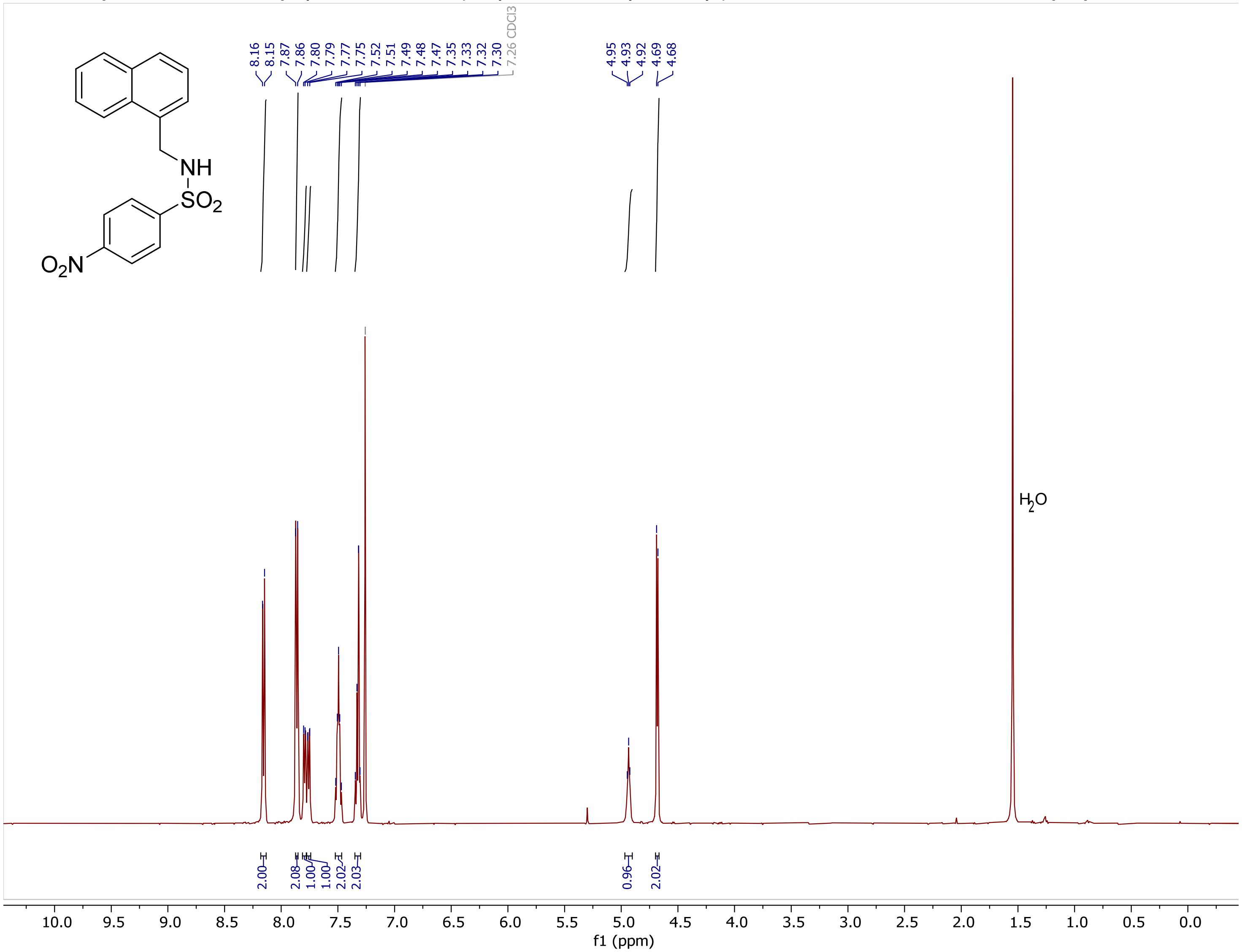

^{13}C {^{1}H} NMR (126 MHz, $CDCl_3$) spectrum of *N*-(naphthalen-1-ylmethyl)-4-nitrobenzenesulfonamide **(S1)**

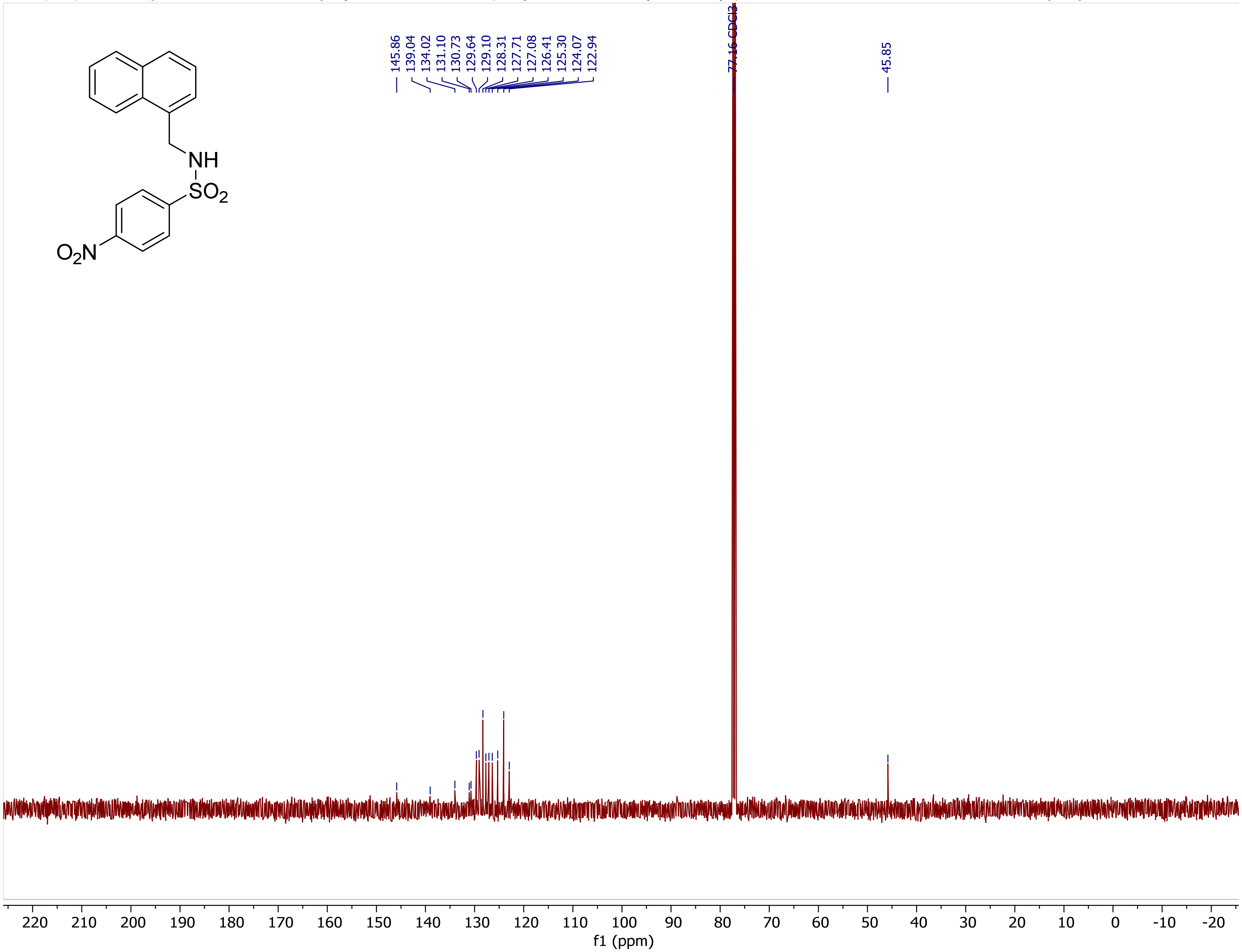

^{1}H NMR (500 MHz, $CDCl_3$) spectrum of *N*-(3-Methylbut-3-en-1-yl)-*N*-(naphthalen-1-ylmethyl)-4-nitrobenzenesulfonamide **(10)**

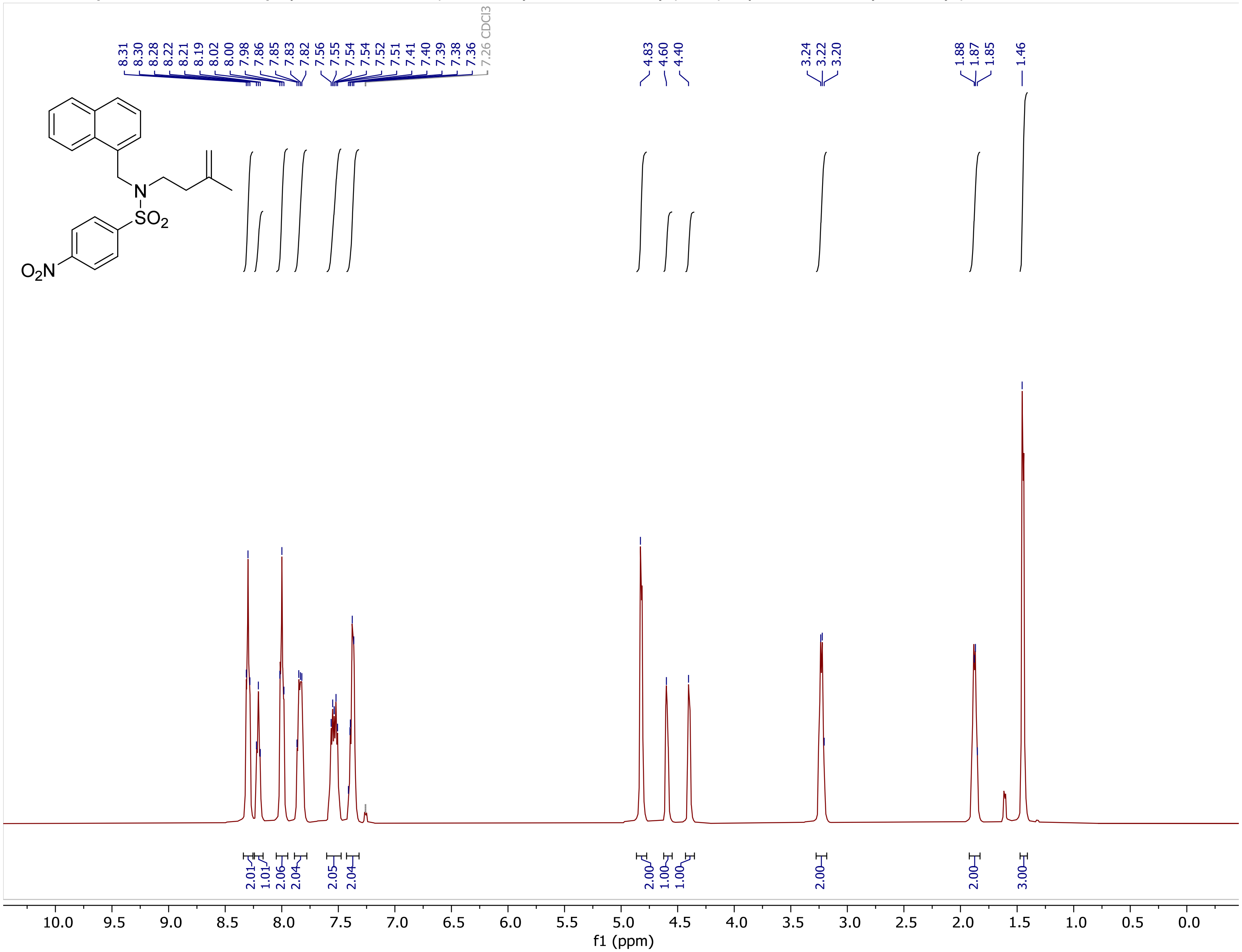

^{13}C NMR (126 MHz, $CDCl_3$) spectrum of *N*-(3-Methylbut-3-en-1-yl)-*N*-(naphthalen-1-ylmethyl)-4-nitrobenzenesulfonamide **(10)**

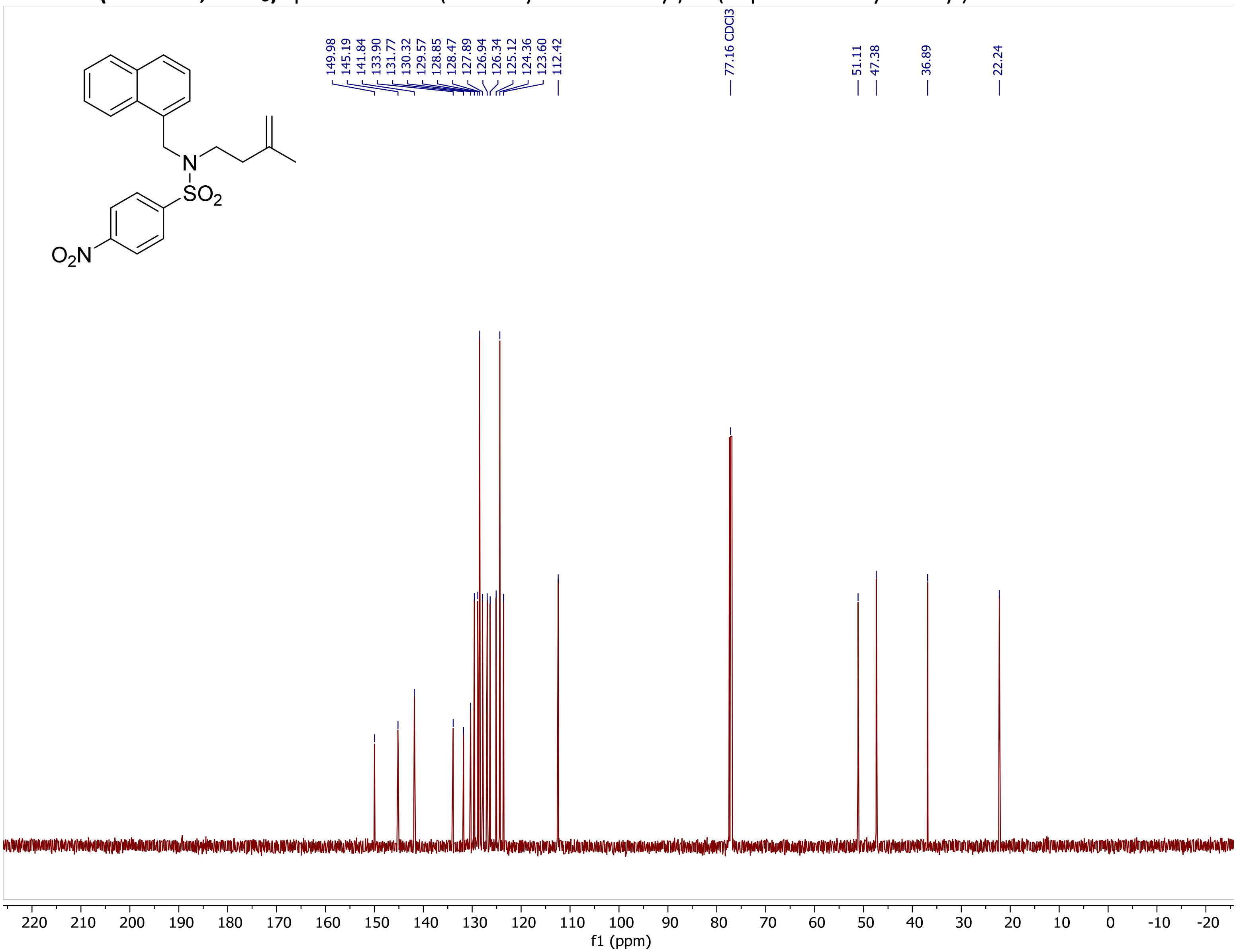

^{1}H NMR (500 MHz, CDCl$_3$) spectrum of *N*-(3-Methyl-3-((triethylsilylperoxy)butyl)-*N*-(naphthalene-1-ylmethyl)-4-nitrobenzenesulfonamide **(16)**

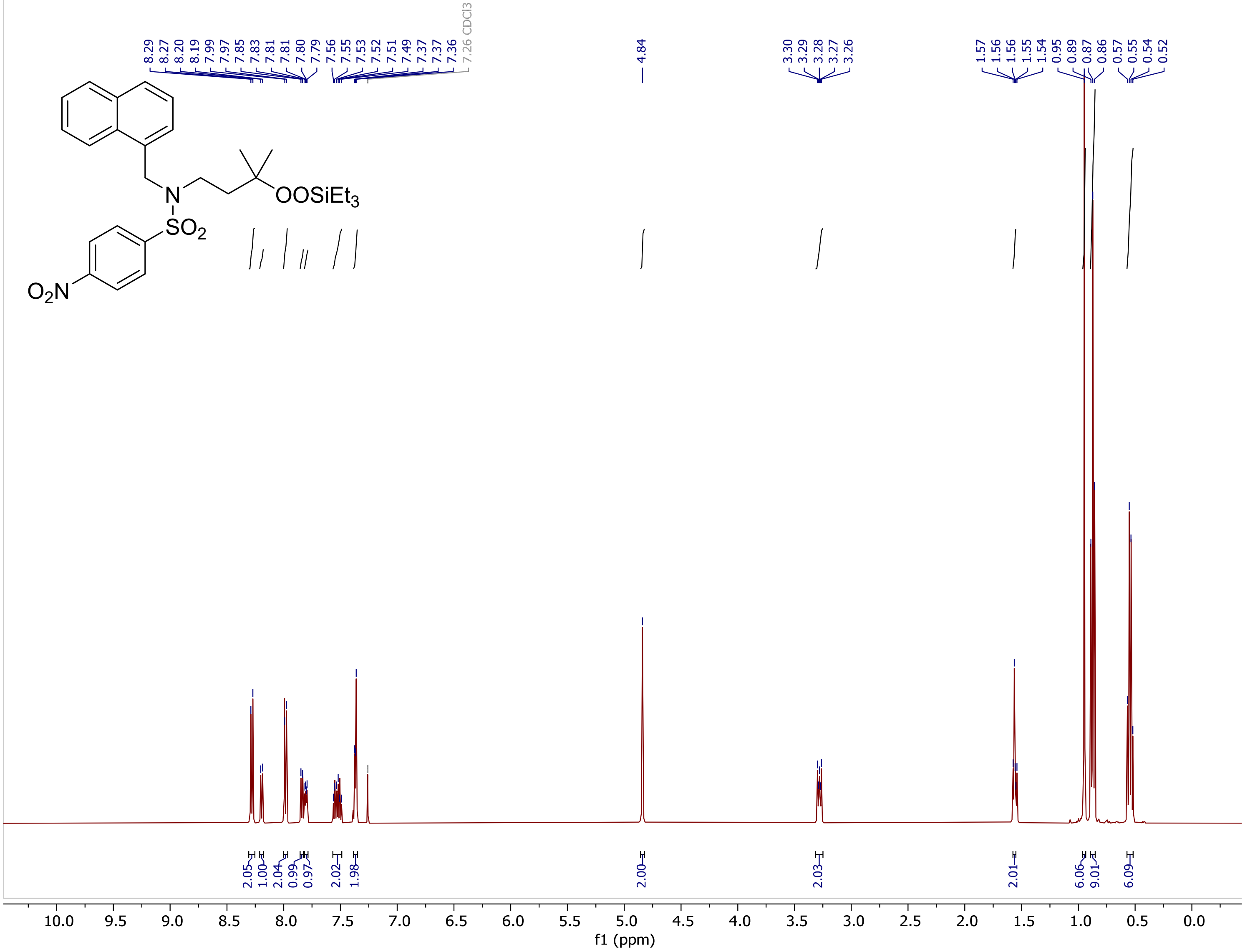

^{13}C NMR (126 MHz, $CDCl_3$) spectrum of *N*-(3-Methyl-3-((triethylsilylperoxy)butyl)-*N*-(naphthalene-1-ylmethyl)-4-nitrobenzenesulfonamide **(16)**

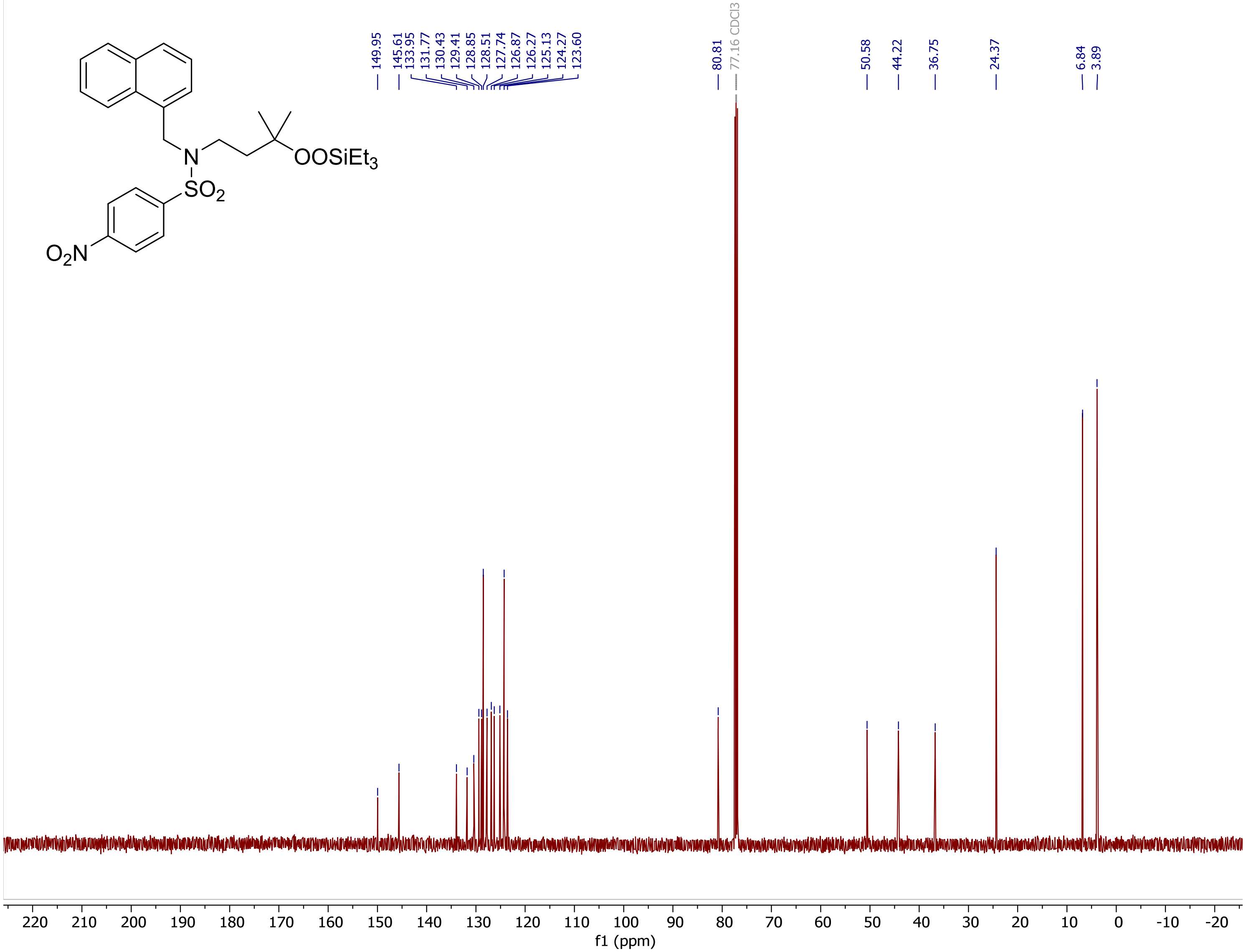

^{1}H NMR (500 MHz, CDCl$_3$) spectrum of 2-Methyl-4-((naphthalen-1-ylmethyl)amino)butan-2-ol **(26)**

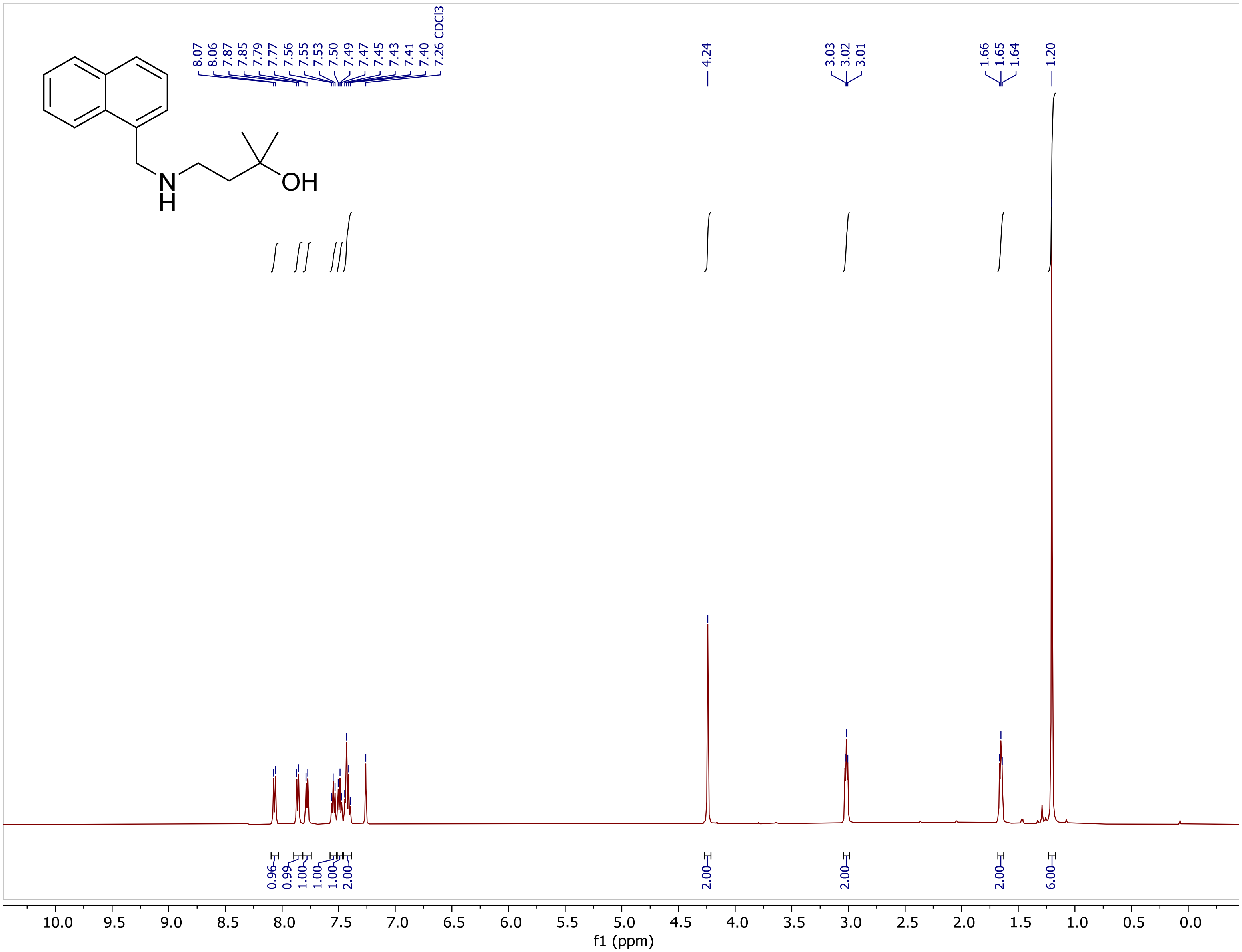

^{13}C NMR (126 MHz, CDCl$_3$) spectrum of 2-Methyl-4-((naphthalen-1-ylmethyl)amino)butan-2-ol **(26)**

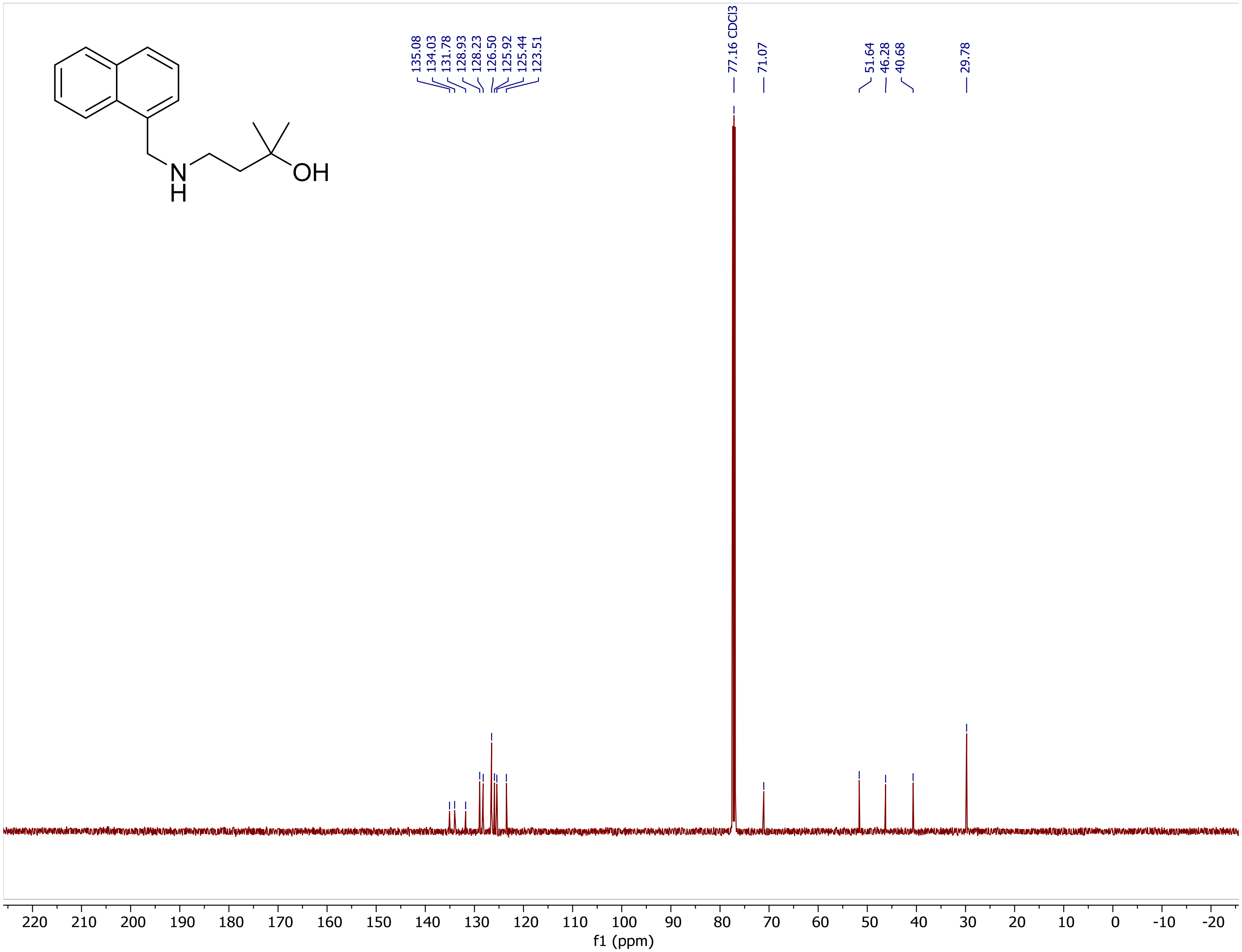

^{1}H NMR (500 MHz, $CDCl_3$) spectrum of *N*-(But-3-en-1-yl)-*N*-(naphthalen-1-ylmethyl)-4-nitrobenzenesulfonamide **(11)**

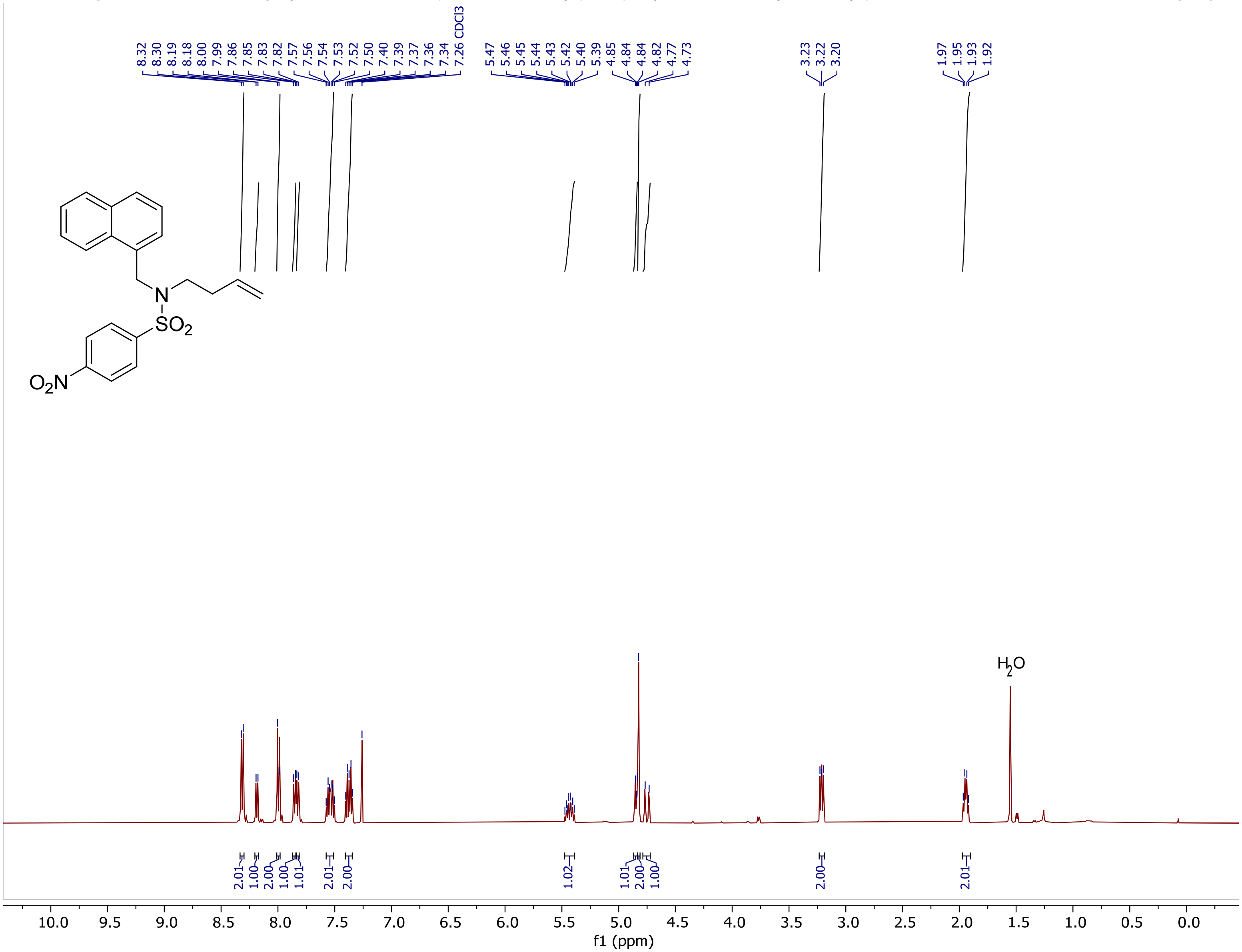

^{13}C {^{1}H} NMR (126 MHz, CDCl$_3$) spectrum of *N*-(But-3-en-1-yl)-*N*-(naphthalen-1-ylmethyl)-4-nitrobenzenesulfonamide **(11)**

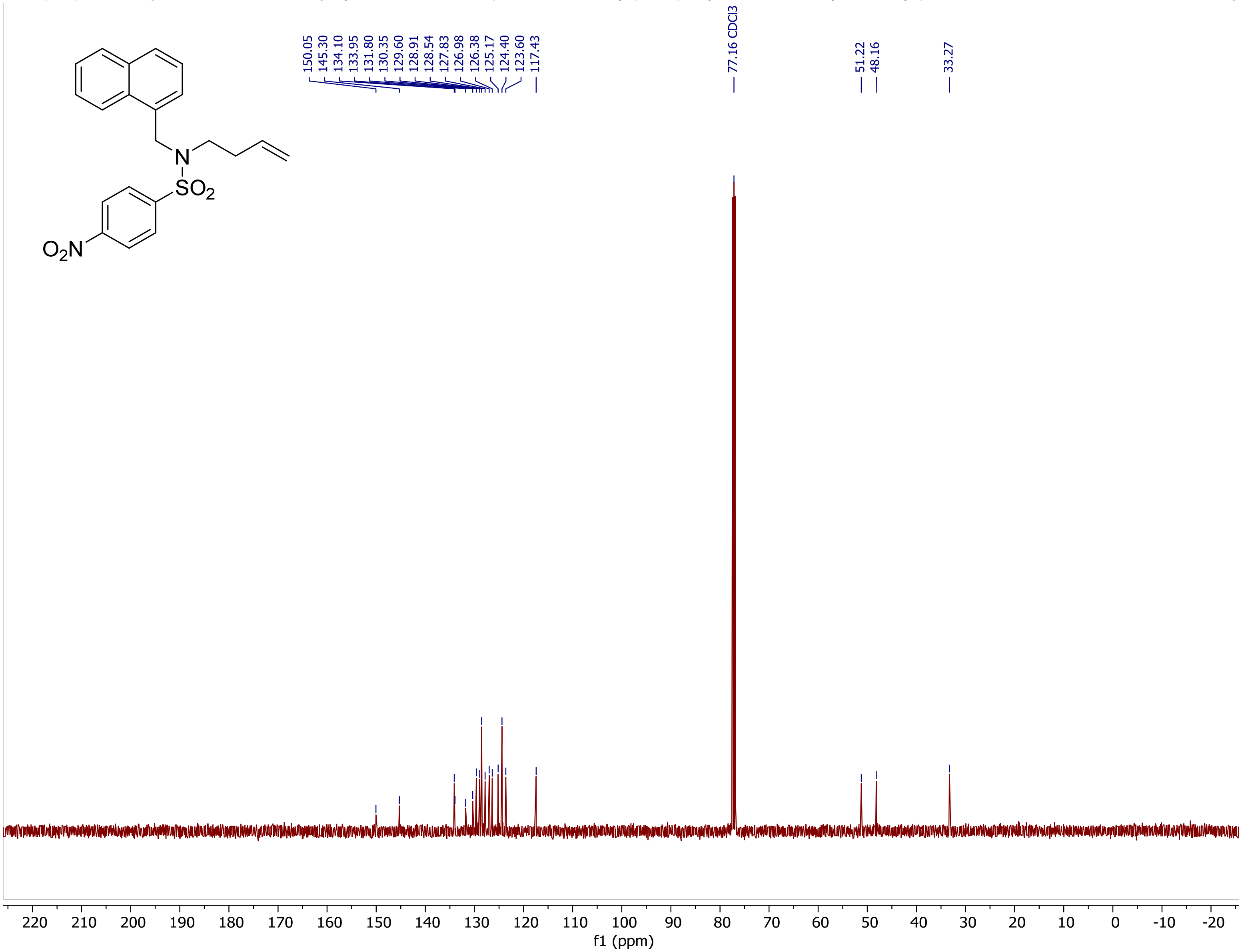

^{1}H NMR (500 MHz, C_6D_6) spectrum of *N*-(Naphthalen-1-ylmethyl)-4-nitro-*N*-(3-((triethylsilyl)peroxy)butyl)benzenesulfonamide **(17)**

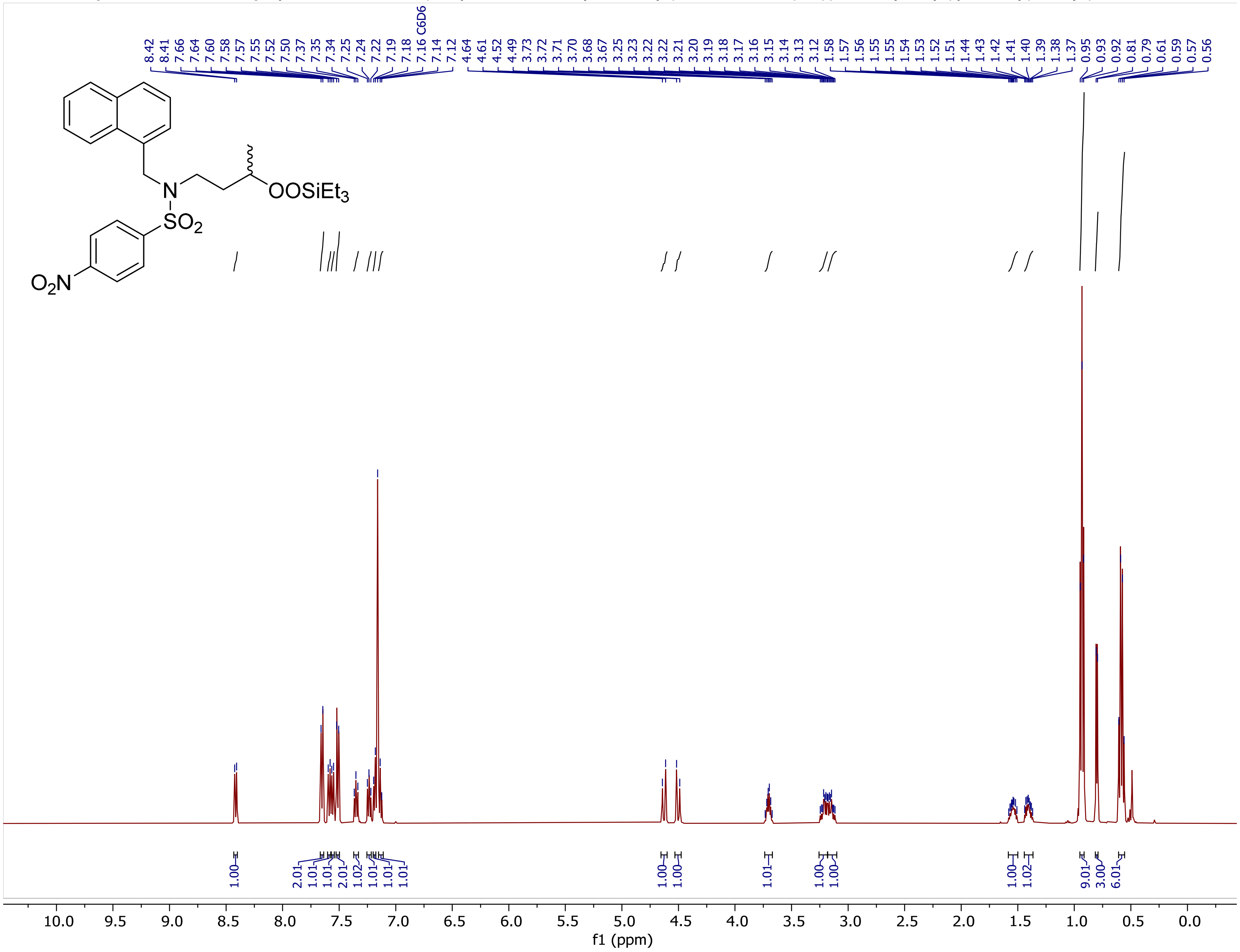

$^{13}C\{^1H\}$ NMR (126 MHz, C_6D_6) spectrum of *N*-(Naphthalen-1-ylmethyl)-4-nitro-*N*-(3-((triethylsilyl)peroxy)butyl)benzenesulfonamide **(17)**

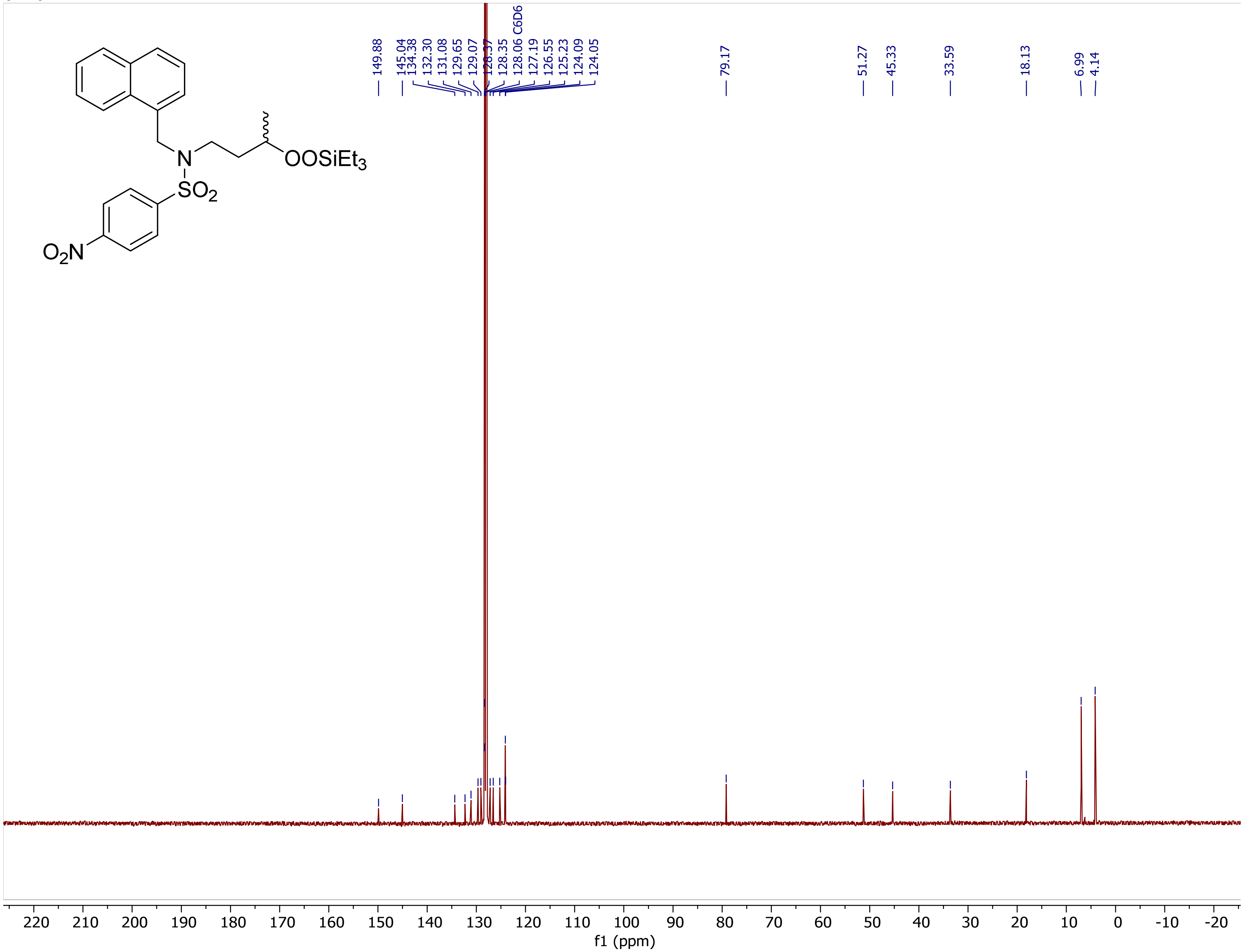

^{1}H NMR (500 MHz, $CDCl_3$) spectrum of *N*-(3-((tert-butyldiphenylsilyl)peroxy)butyl) *N*-(Naphthalen-1-ylmethyl)-4-nitrobenzenesulfonamide **(22)**

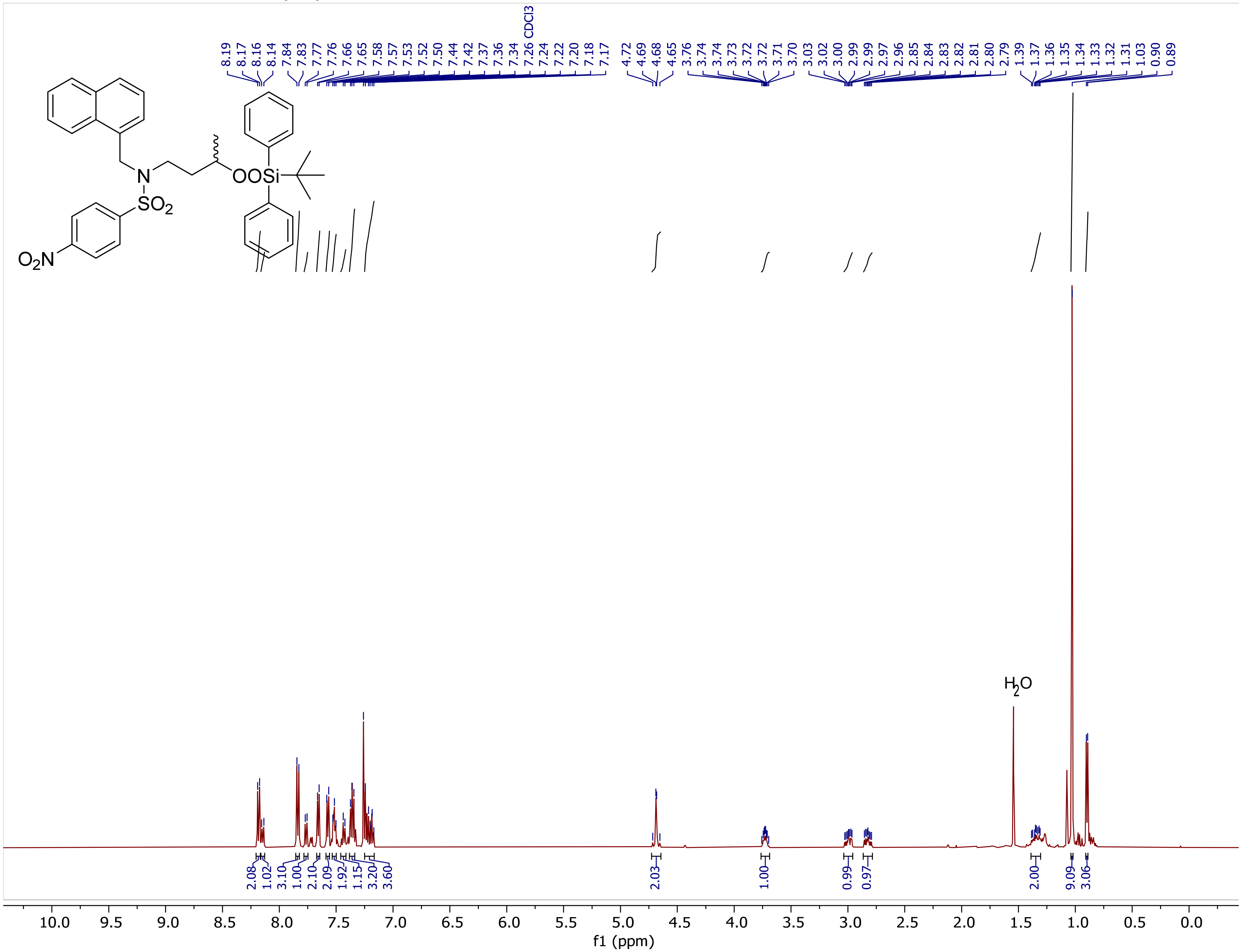

^{13}C {^{1}H} NMR (126 MHz, $CDCl_3$) spectrum of *N*-(3-((tert-butyldiphenylsilyl)peroxy)butyl) *N*-(Naphthalen-1-ylmethyl)-4-nitrobenzenesulfonamide **(22)**

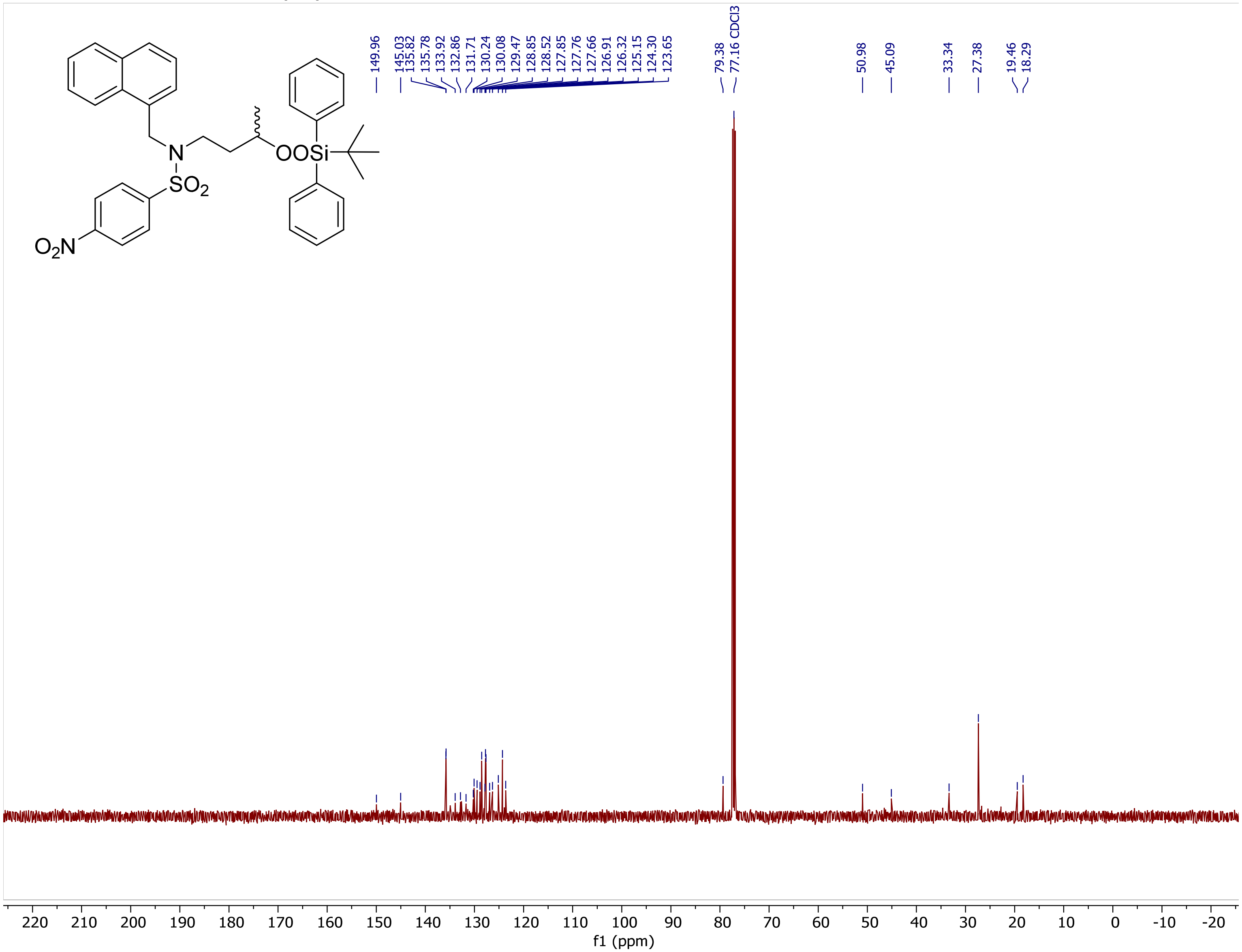

^{1}H NMR (500 MHz, CDCl$_3$) spectrum of *N*-(Naphthalen-1-ylmethyl)-4-nitro-*N*-(3-oxobutyl)benzenesulfonamide **(27)**

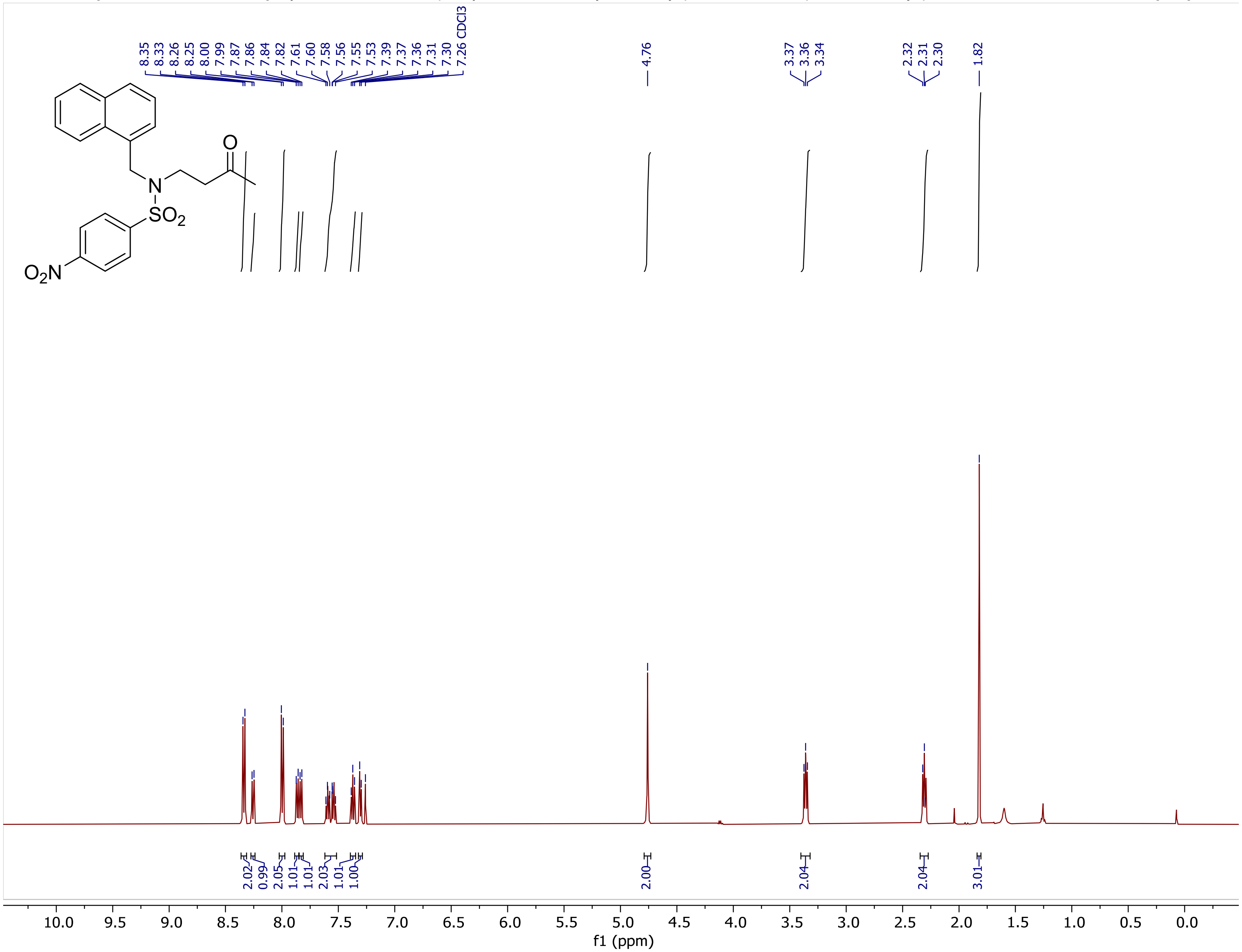

^{13}C {^{1}H} NMR (126 MHz, $CDCl_3$) spectrum of *N*-(Naphthalen-1-ylmethyl)-4-nitro-*N*-(3-oxobutyl)benzenesulfonamide **(27)**

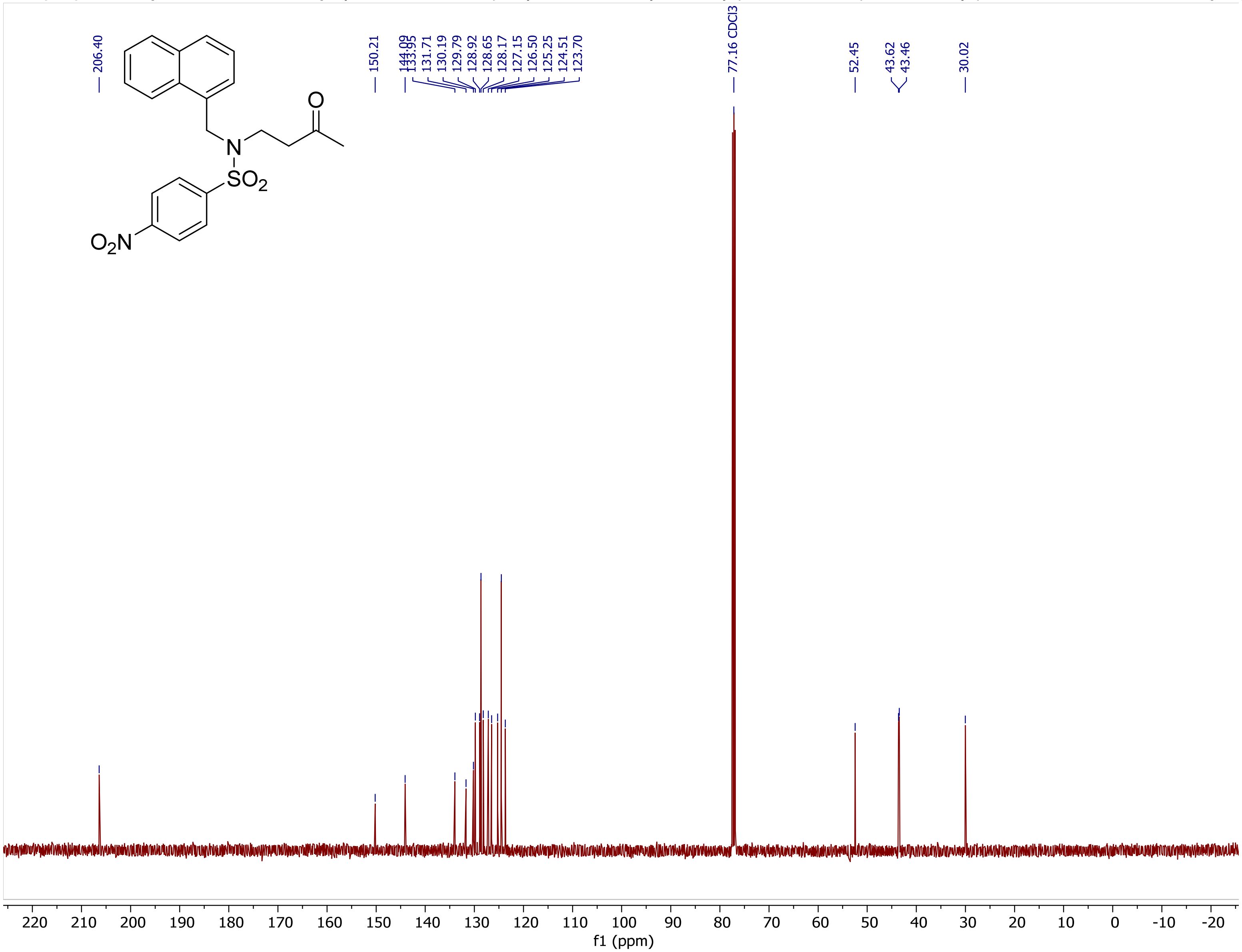

^{1}H NMR (500 MHz, C_6D_6) spectrum of *N*-(Naphthalen-1-ylmethyl)-2, 4-dinitrobenzenesulfonamide **(S2)**

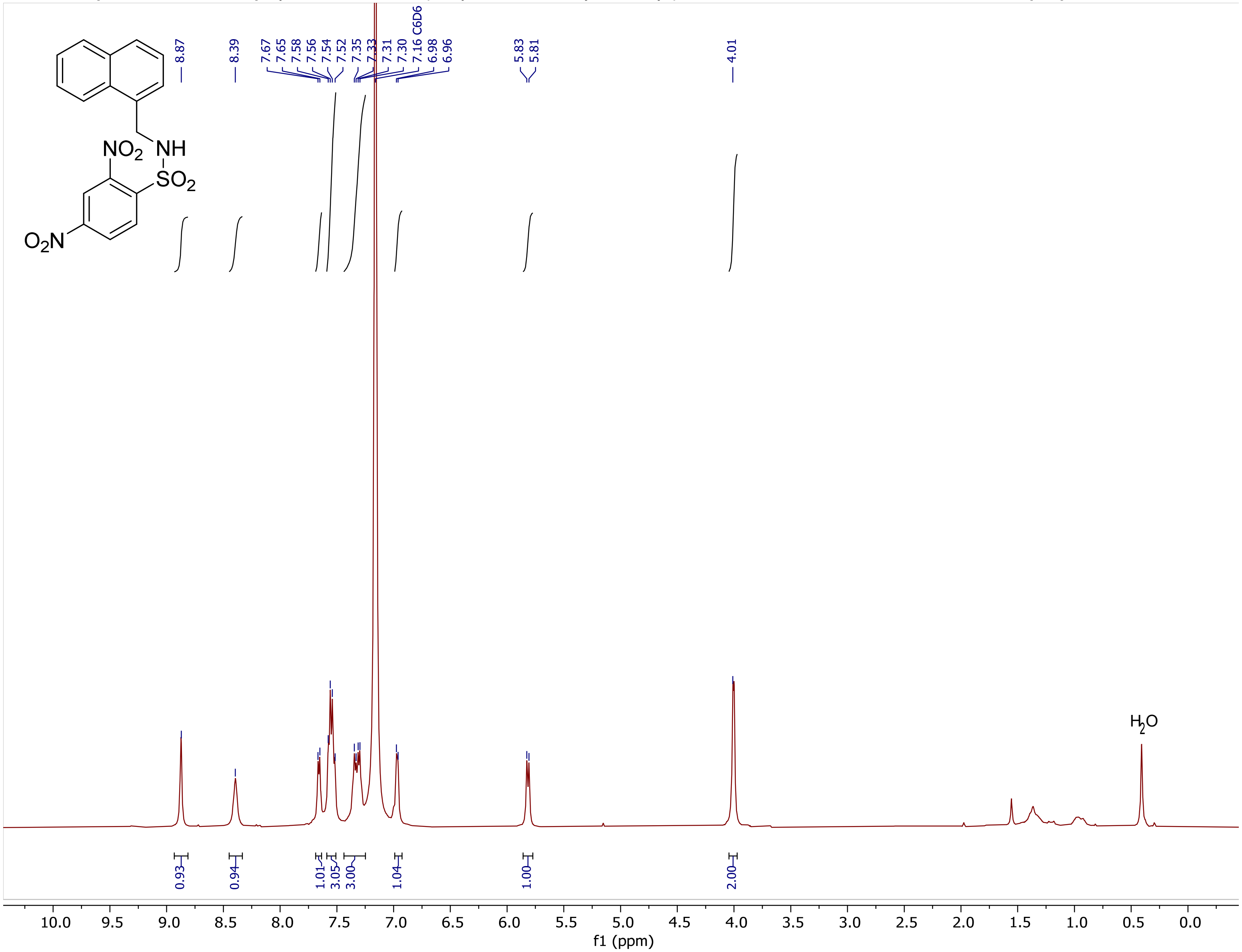

^{13}C NMR (126 MHz, C_6D_6) spectrum of *N*-(Naphthalen-1-ylmethyl)-2, 4-dinitrobenzenesulfonamide **(S2)**

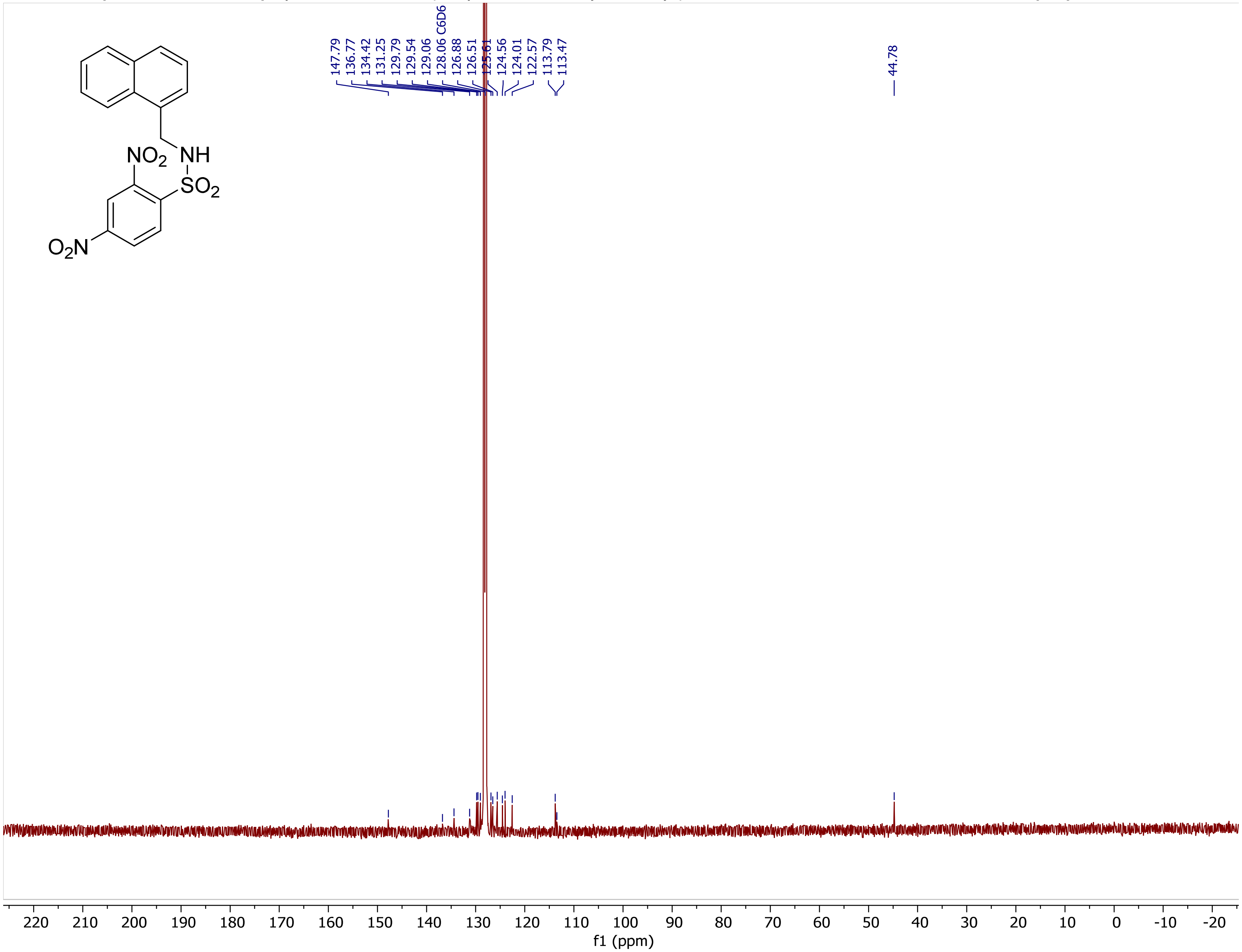

^{1}H NMR (500 MHz, CDCl$_3$) spectrum of *N*-(But-3-en-1-yl)-*N*-(naphthalen-1-ylmethyl)-2, 4-dinitro-4-benzenesulfonamide **(12)**

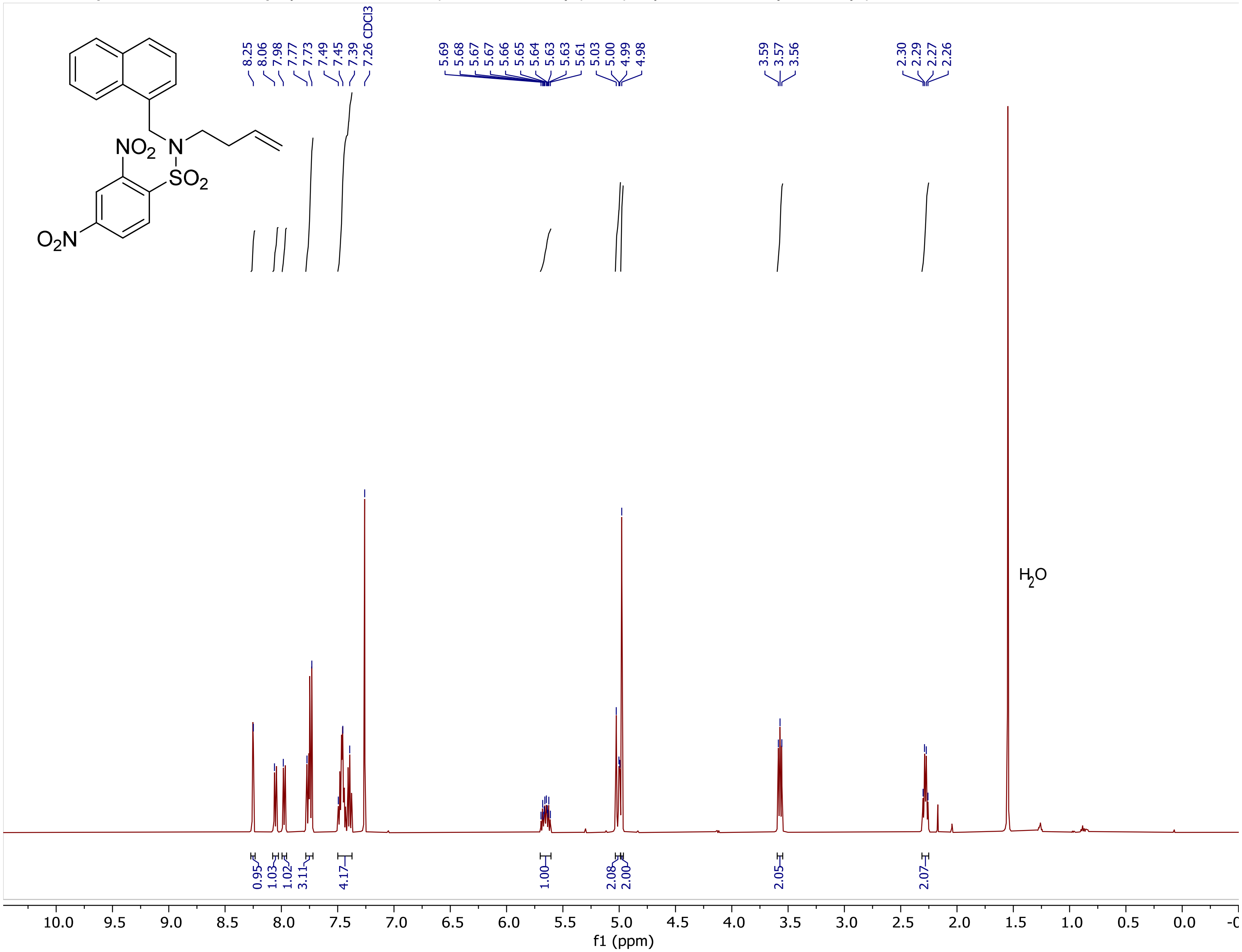

$^{13}C\ \{^{1}H\}$ NMR (126 MHz, $CDCl_3$) spectrum of *N*-(But-3-en-1-yl)-*N*-(naphthalen-1-ylmethyl)-2, 4-dinitro-4-benzenesulfonamide **(12)**

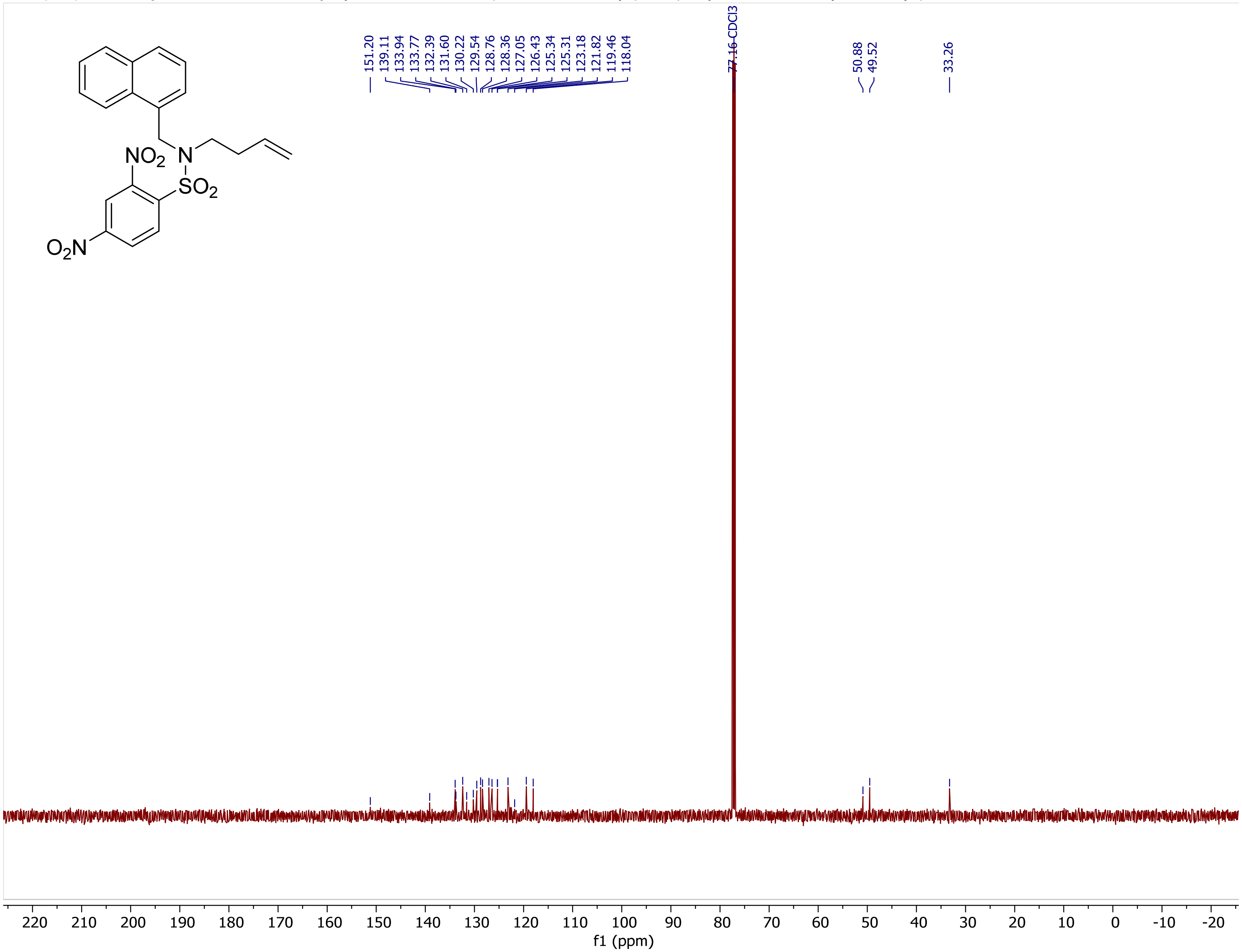

^{1}H NMR (500 MHz, CDCl$_3$) spectrum of *N*-(naphthalen-1-ylmethyl)-2, 4-dinitro-*N*-(3-((triethylsilyl)peroxy)butyl)benzenesulfonamide **(18)**

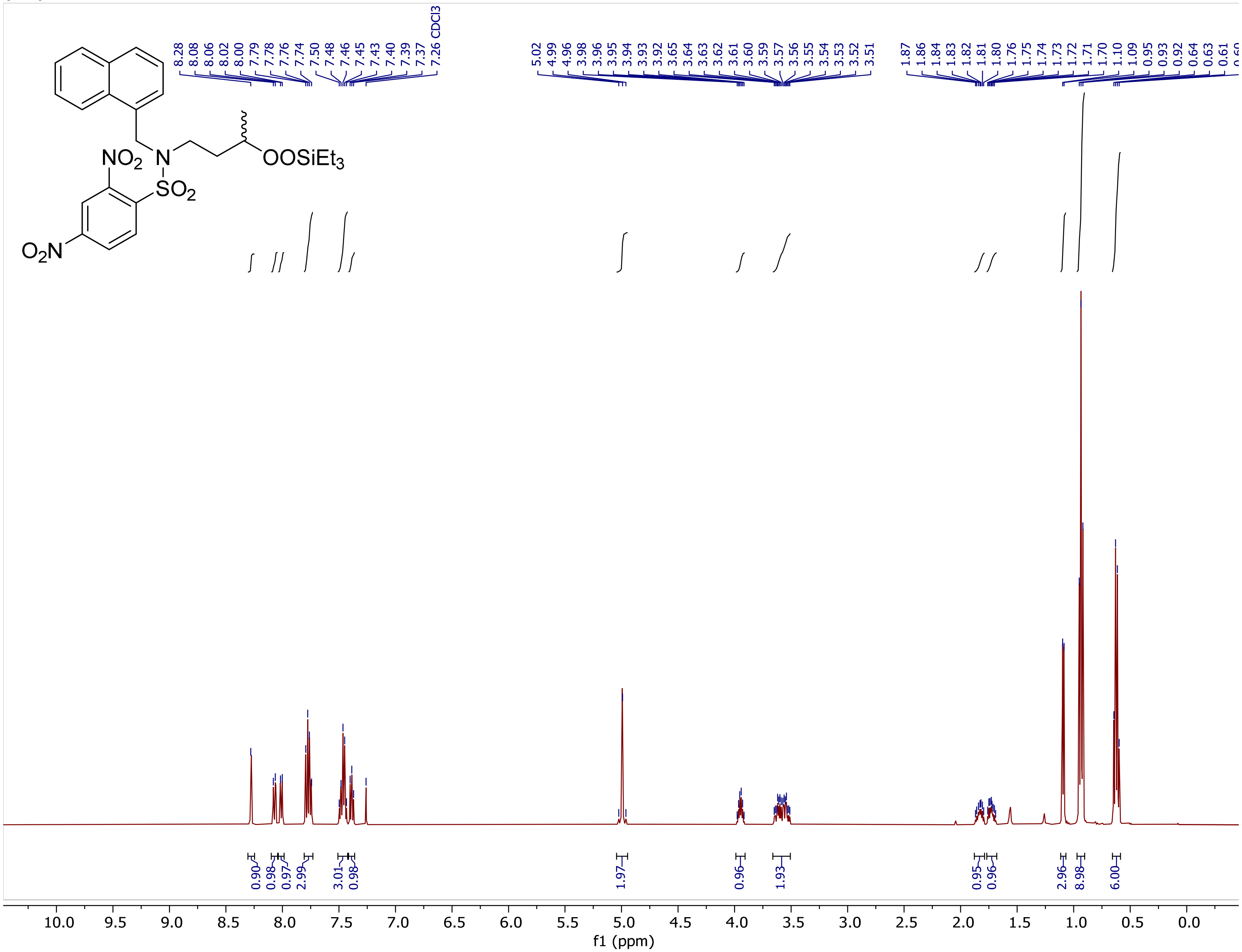

^{13}C {^{1}H} NMR (126 MHz, $CDCl_3$) spectrum of *N*-(naphthalen-1-ylmethyl)-2, 4-dinitro-*N*-(3-((triethylsilyl)peroxy)butyl)benzenesulfonamide **(18)**

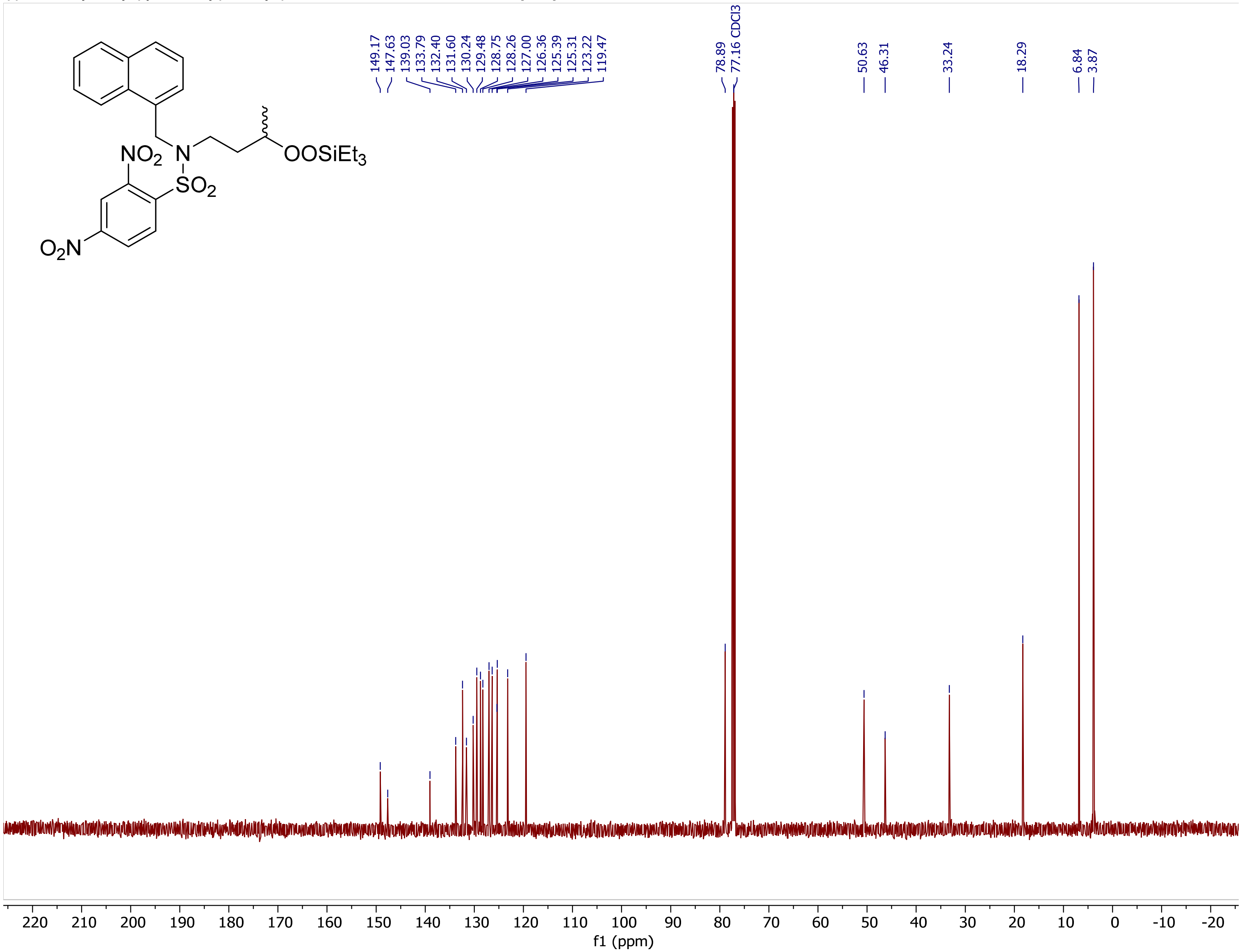

^{1}H NMR (500 MHz, CDCl$_3$) spectrum of *N*-(3-((*tert*-Butyldiphenylsilyl)peroxy)butyl)-*N*-(naphthalene-1-ylmethyl)-2, 4-dinitrobenzenesulfonamide **(23)**

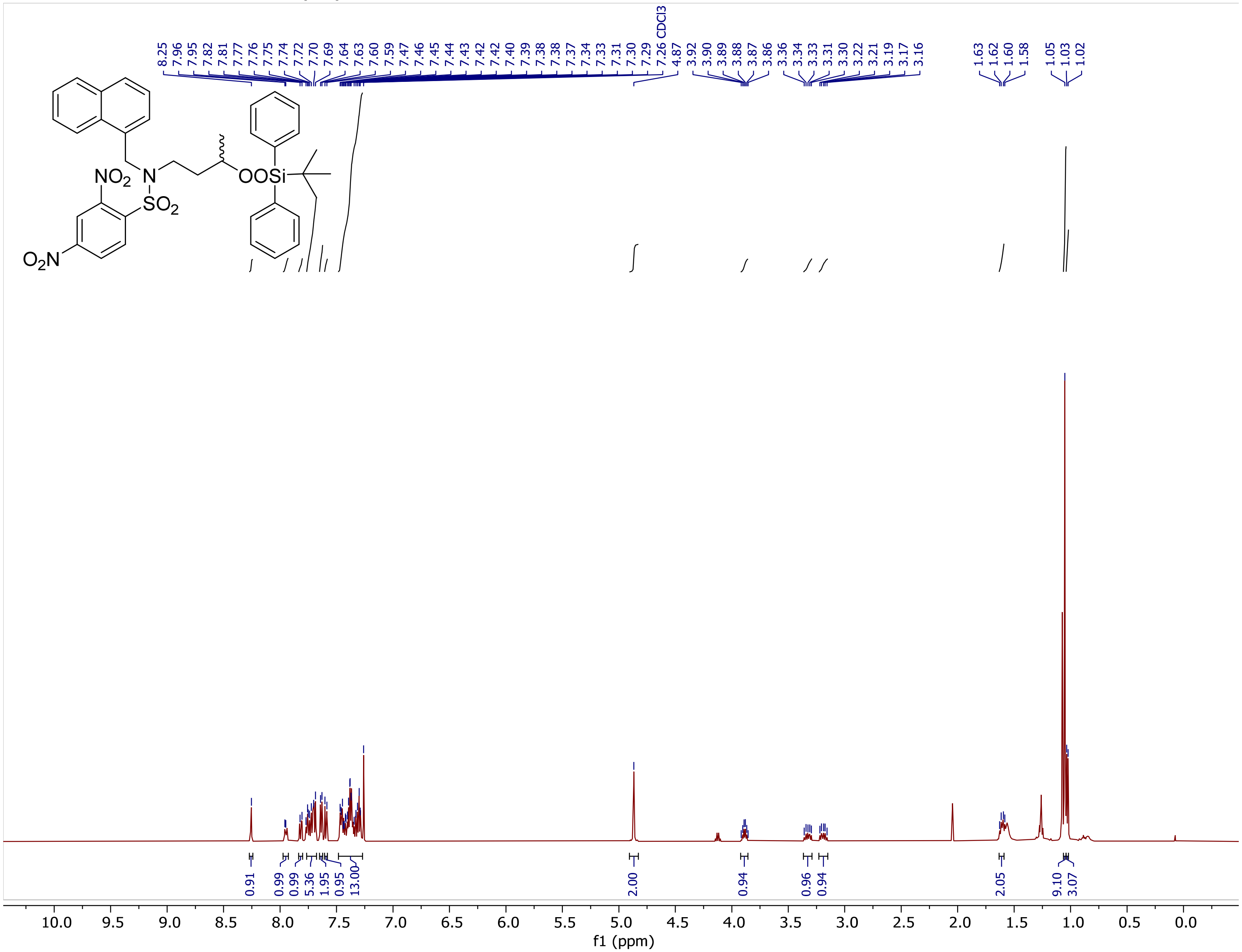

^{13}C {^{1}H} NMR (126 MHz, $CDCl_3$)** spectrum of *N*-(3-((*tert*-Butyldiphenylsilyl)peroxy)butyl)-*N*-(naphthalene-1-ylmethyl)-2, 4-dinitrobenzenesulfonamide **(23)**

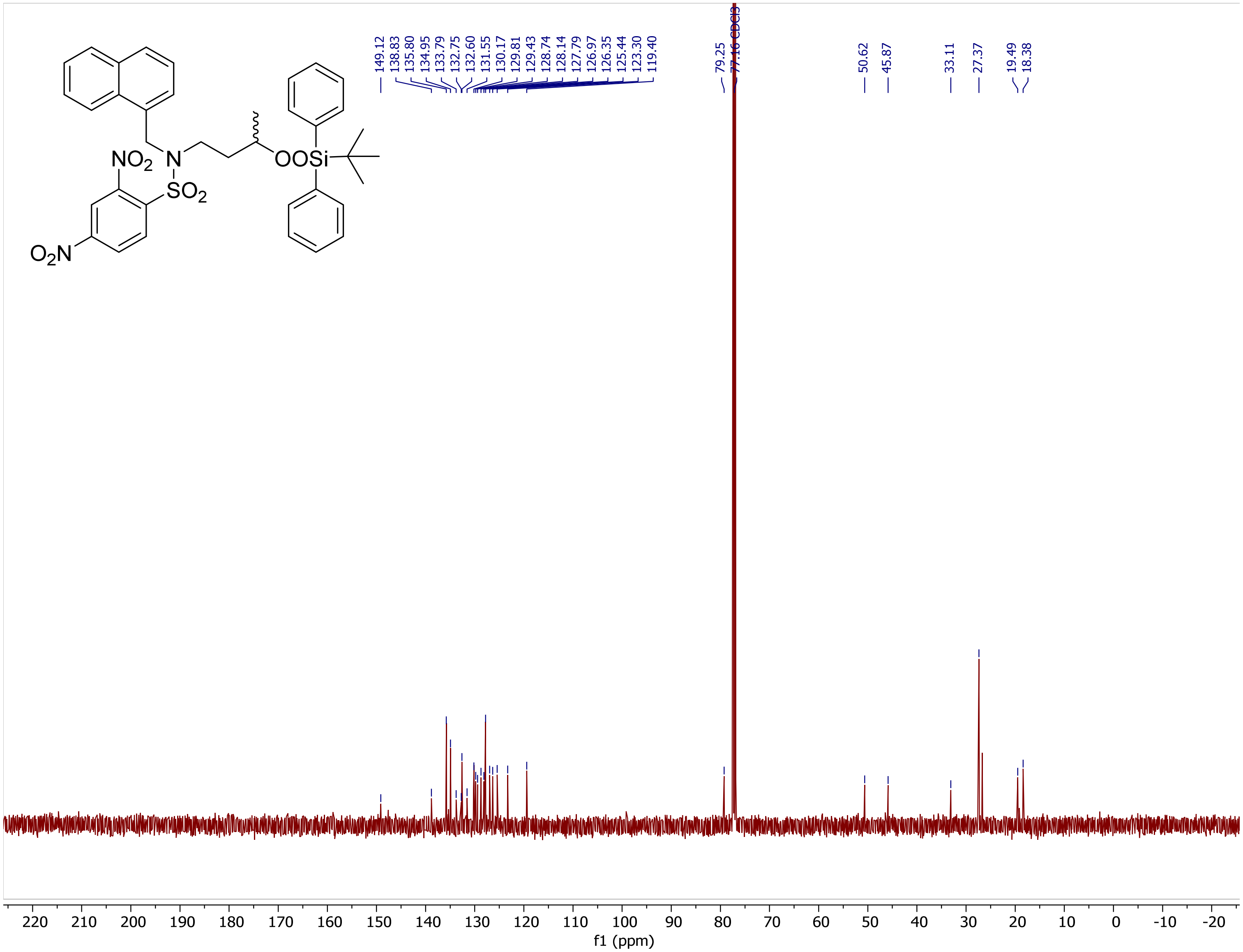

^{1}H NMR (500 MHz, $CDCl_3$) spectrum of 4-((Naphthalen-1-ylmethyl)amino)butan-2-one (**28**)

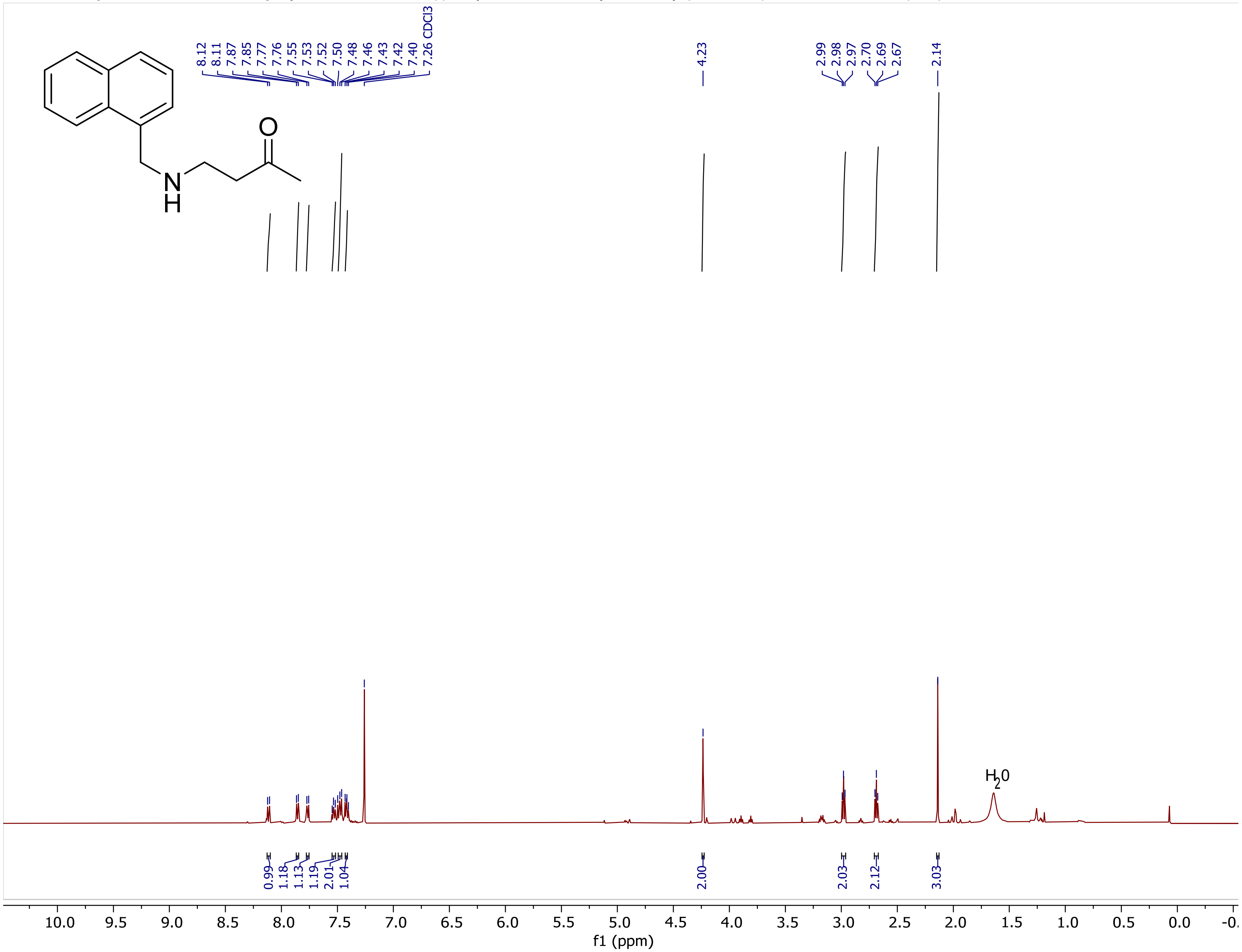

^{1}H NMR (500 MHz, C_6D_6) spectrum of 4-((2-Hydroxyethyl)thio)-*N*-(naphthalene-1-ylmethyl)-2-nitro-*N*-(3-oxobutyl)benzenesulfonamide (**30**)

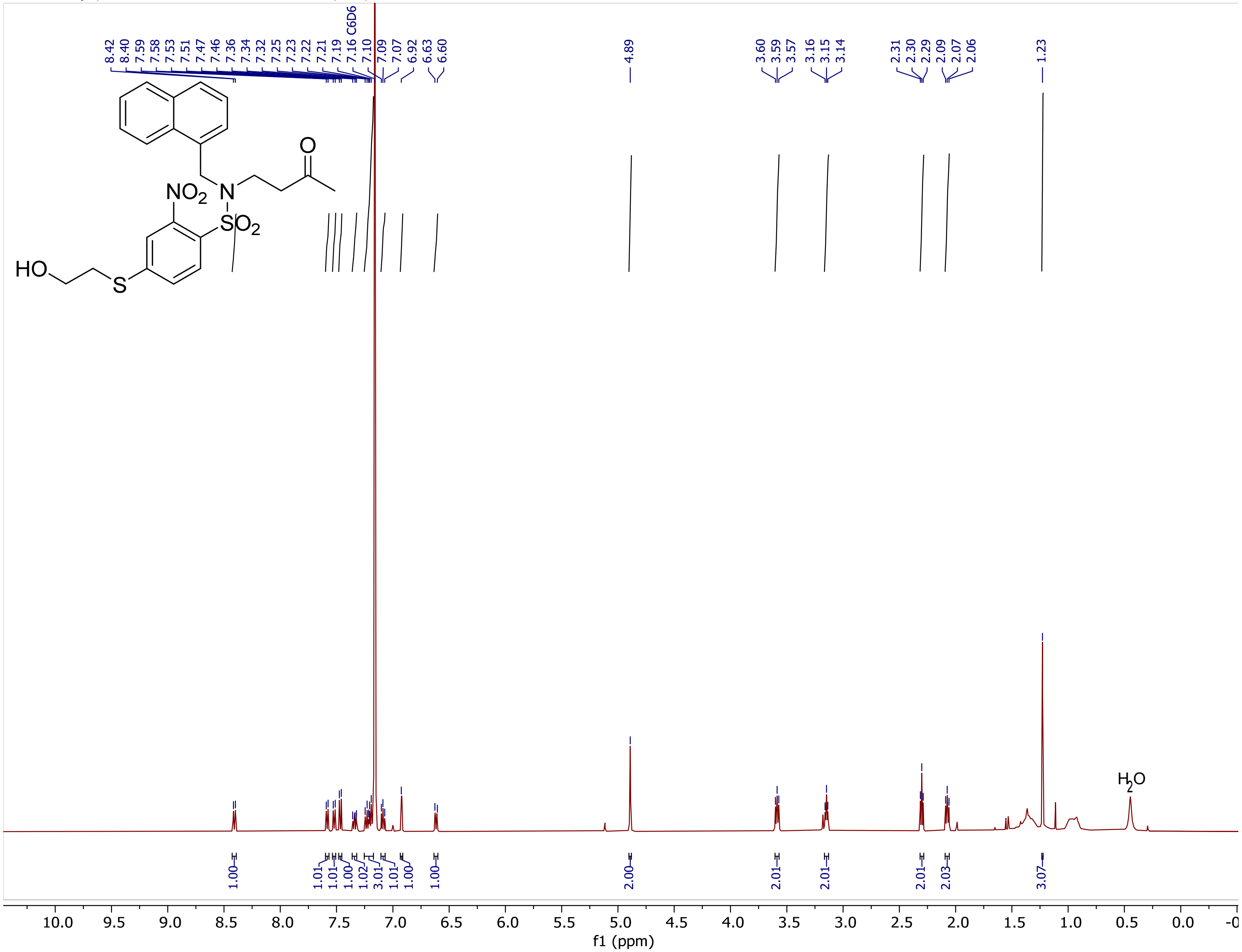

COSY NMR (500 MHz, $CDCl_3$) spectrum of 4-((2-Hydroxyethyl)thio)-*N*-(naphthalene-1-ylmethyl)-2-nitro-*N*-(3-oxobutyl)benzenesulfonamide (**30**)

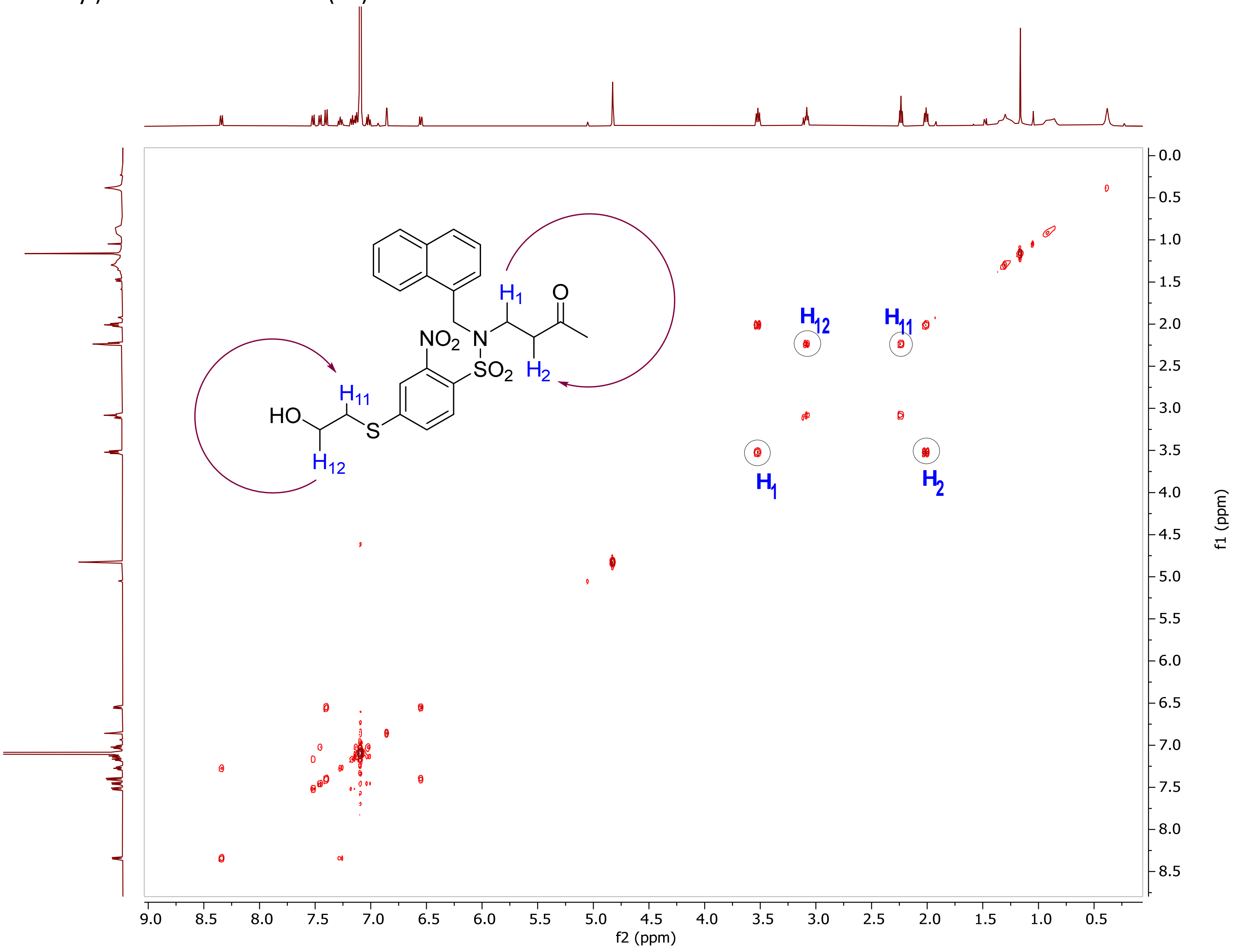

1D NOESY NMR and ^{1}H NMR stacked (500 MHz, $CDCl_3$) spectrum of 4-((2-Hydroxyethyl)thio)-*N*-(naphthalene-1-ylmethyl)-2-nitro-*N*-(3-oxobutyl)benzenesulfonamide (**30**)

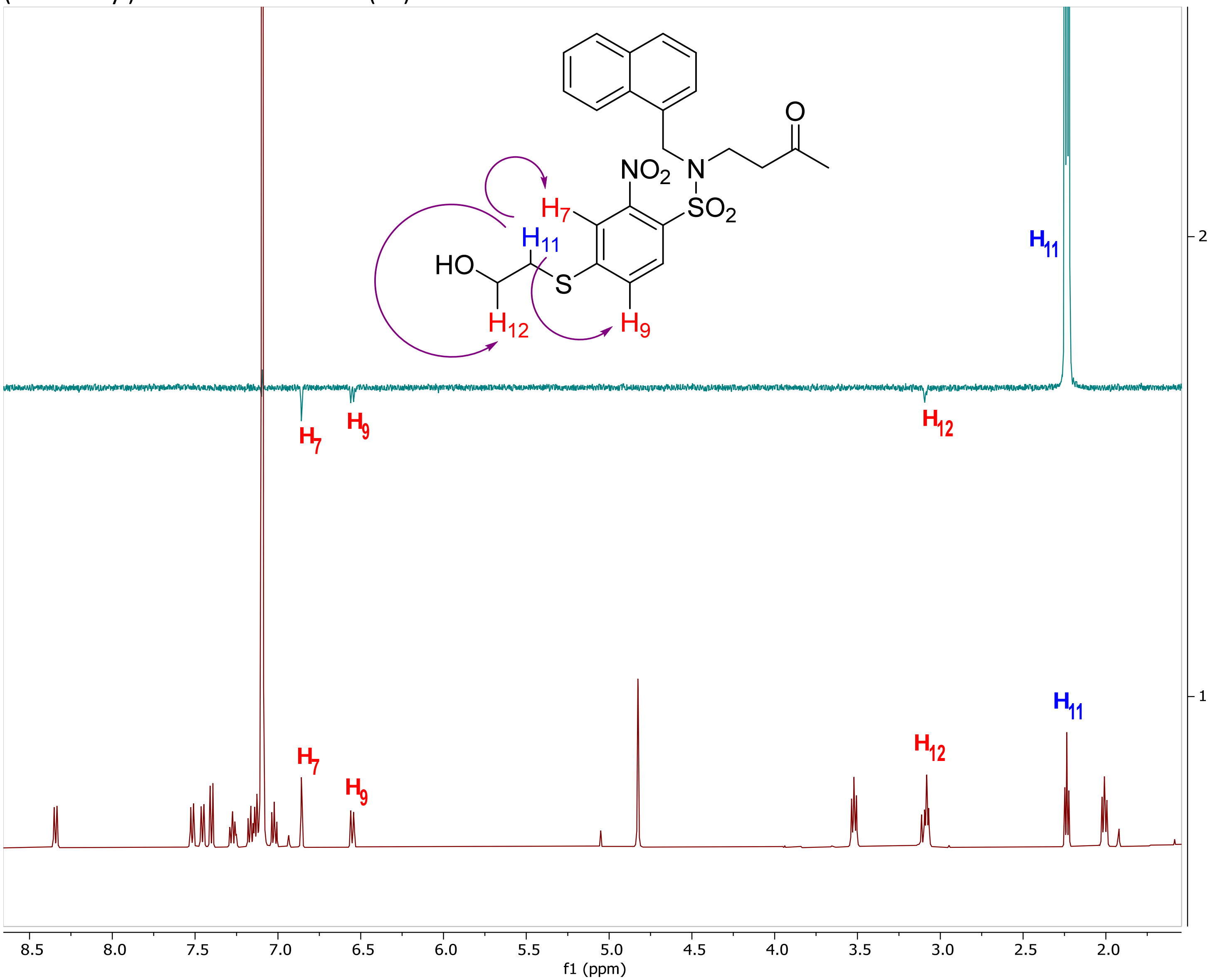

^{1}H NMR (500 MHz, $CDCl_3$) spectrum of *N*-(3-((*tert*-Butyldiphenylsilyl)peroxy)butyl)-4-((2-hydroxyethyl)thio)-*N*-(Naphthalen-1-ylmethyl)-2-nitrobenzenesulfonamide **(31)**

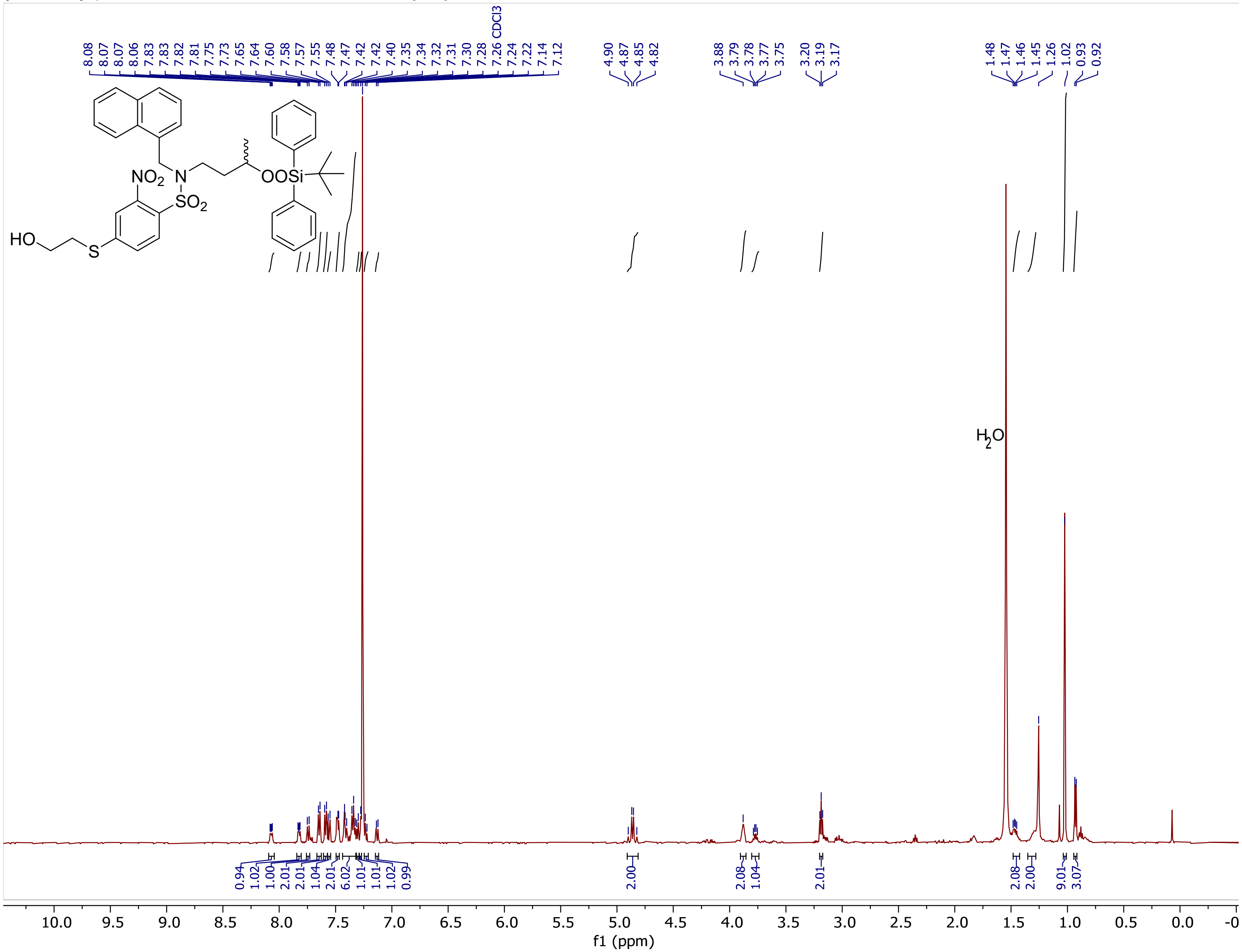

^{1}H NMR (500 MHz, $CDCl_3$) spectrum of *N*-(Naphthalen-1-ylmethyl)-2-nitro-4-(trifluoromethyl)benzenesulfonamide (**S3**)

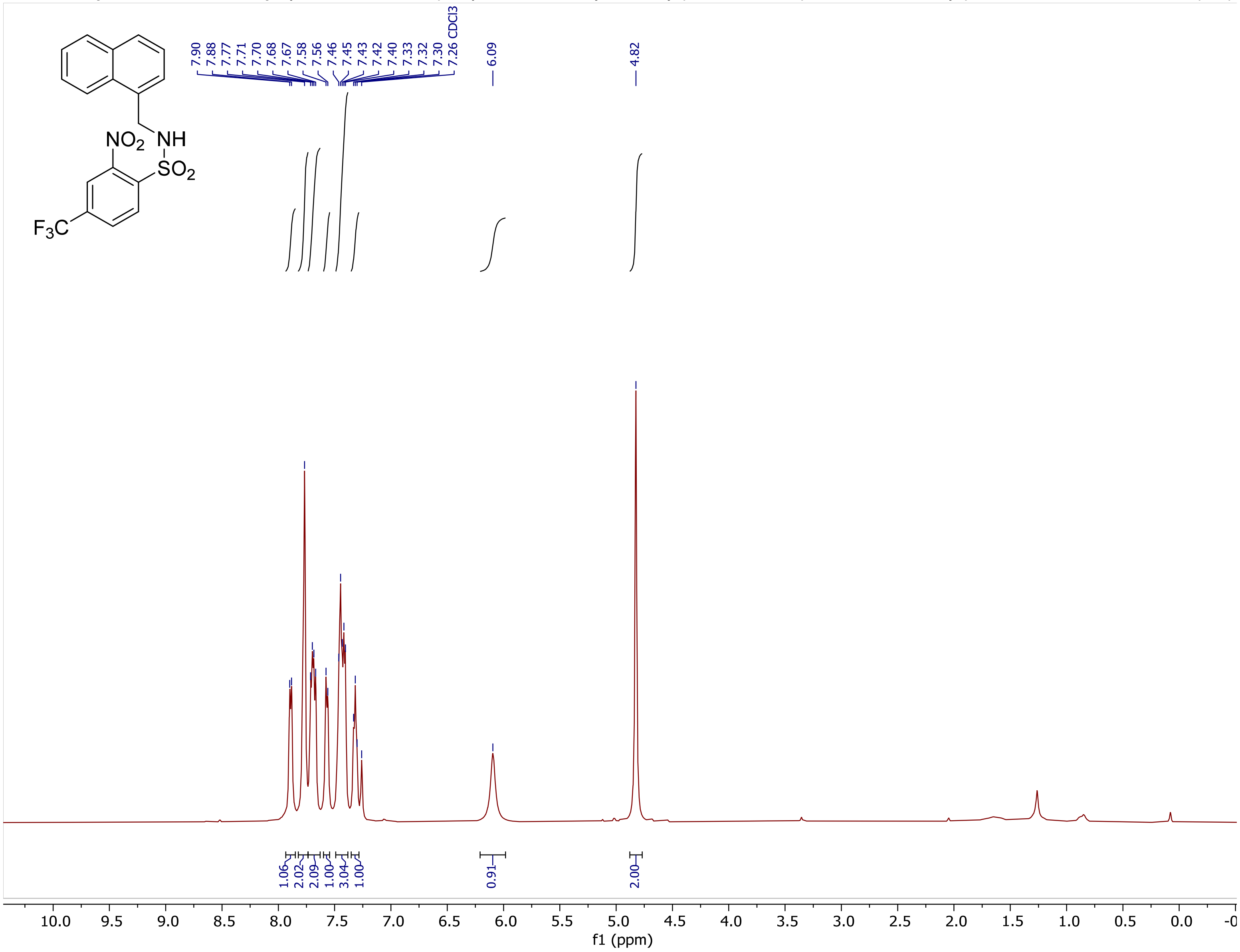

^{13}C {^{1}H} NMR (126 MHz, $CDCl_3$) spectrum of *N*-(Naphthalen-1-ylmethyl)-2-nitro-4-(trifluoromethyl)benzenesulfonamide (**S3**)

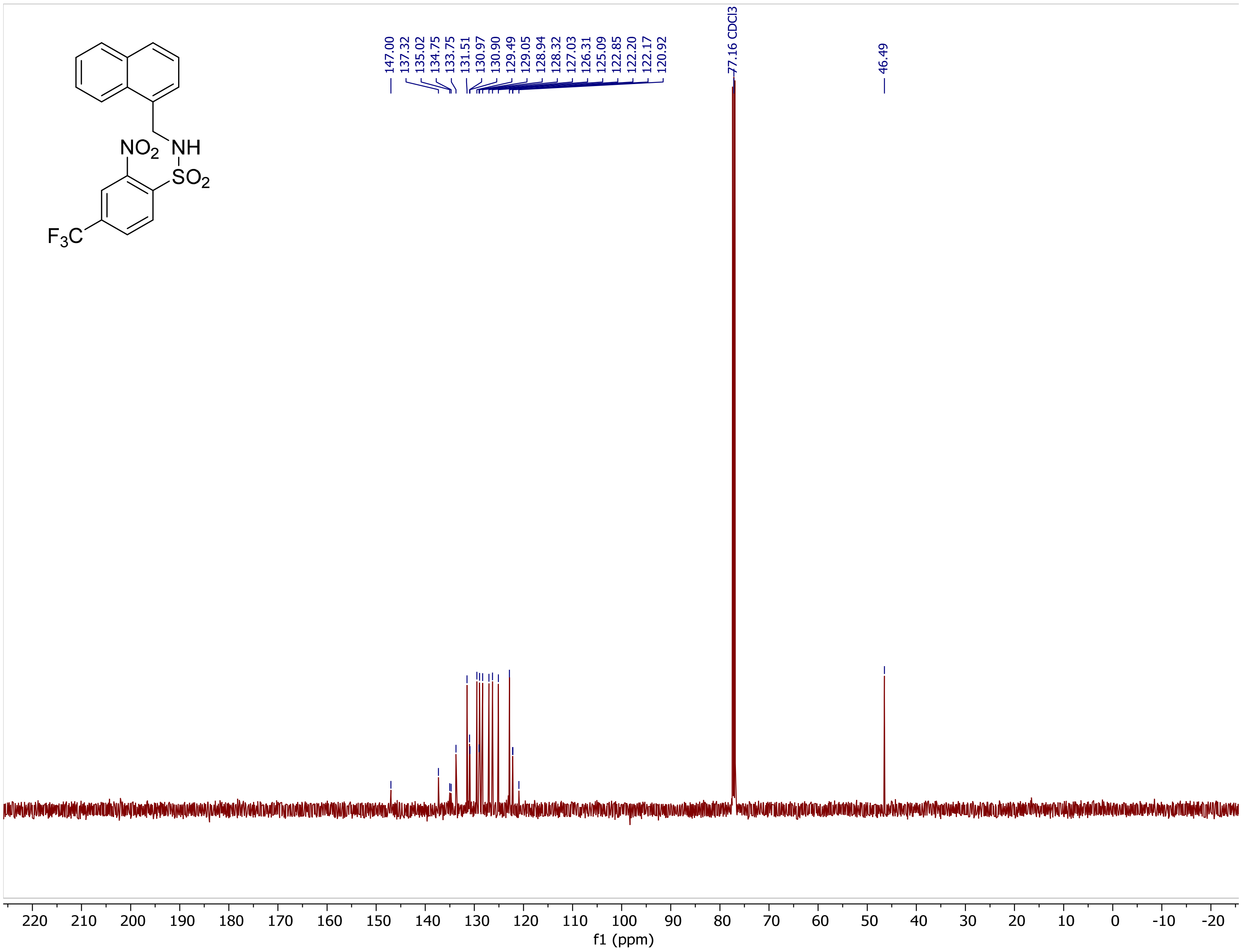

^{1}H NMR (500 MHz, $CDCl_3$) spectrum of *N*-(But-3-en-1-yl)-*N*-(naphthalen-1-ylmethyl)-2-nitro-4-(trifluoromethyl)benzenesulfonamide(**13**)

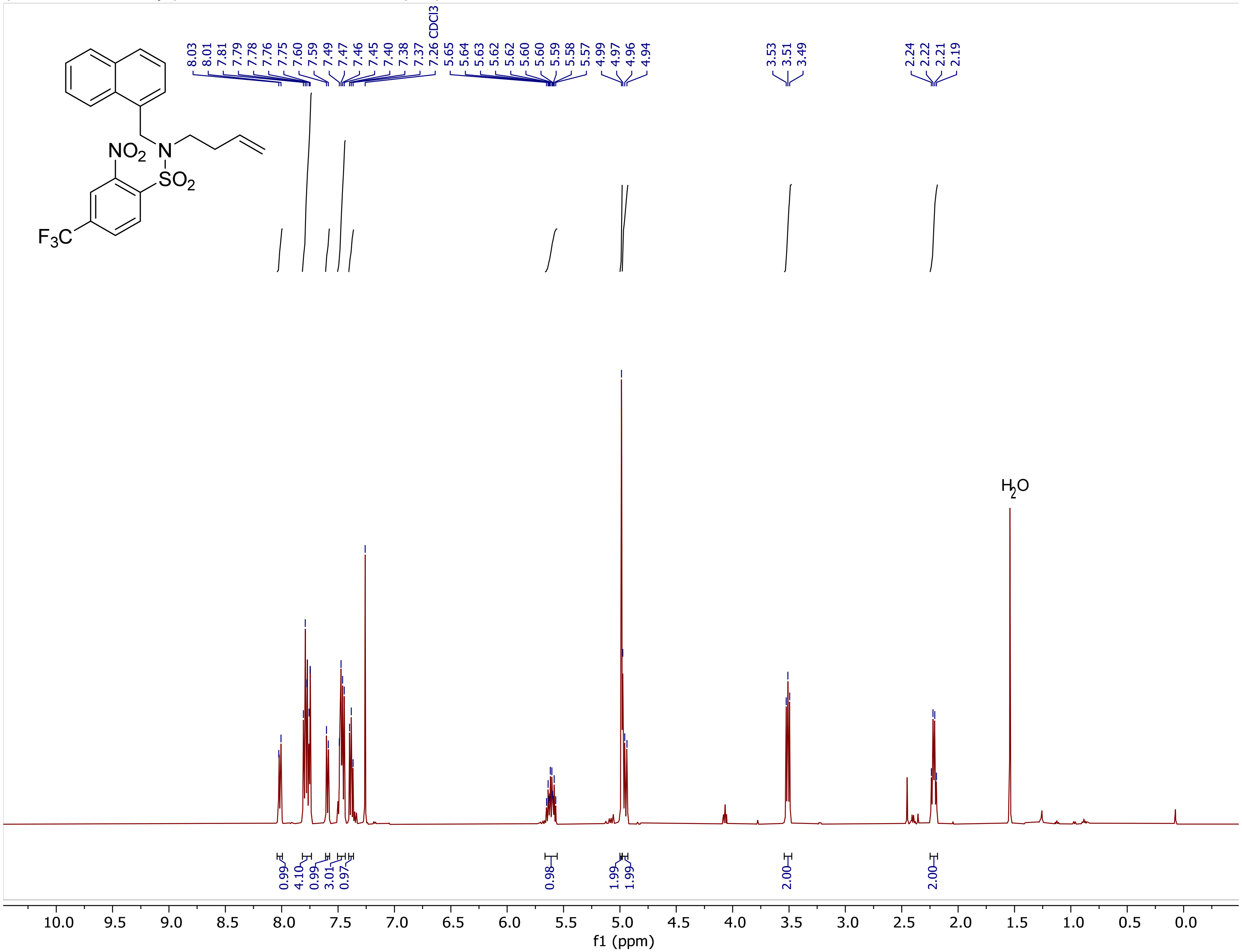

^{13}C {^{1}H, ^{19}F} NMR (126 MHz, $CDCl_3$) spectrum of *N*-(But-3-en-1-yl)-*N*-(naphthalen-1-ylmethyl)-2-nitro-4-(trifluoromethyl)benzenesulfonamide (**13**)

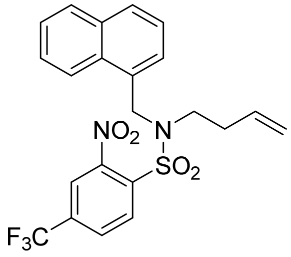

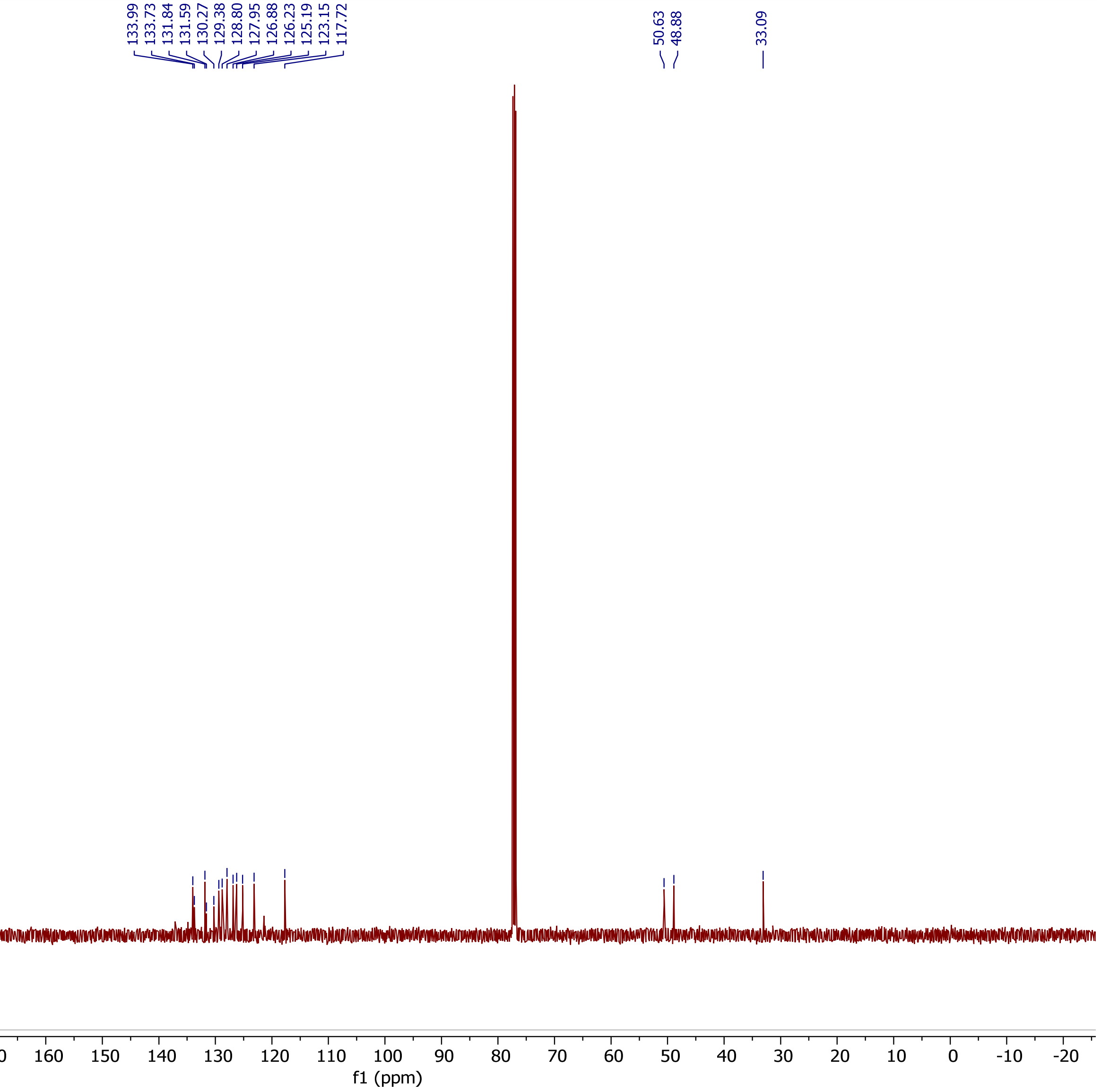

^{1}H NMR (500 MHz, CDCl$_3$) spectrum of *N*-(Naphthalen-1-ylmethyl)-2-nitro-*N*-(3-((triethylsilyl)peroxy)butyl)-4-(trifluoromethyl)benzenesulfonamide (**19**)

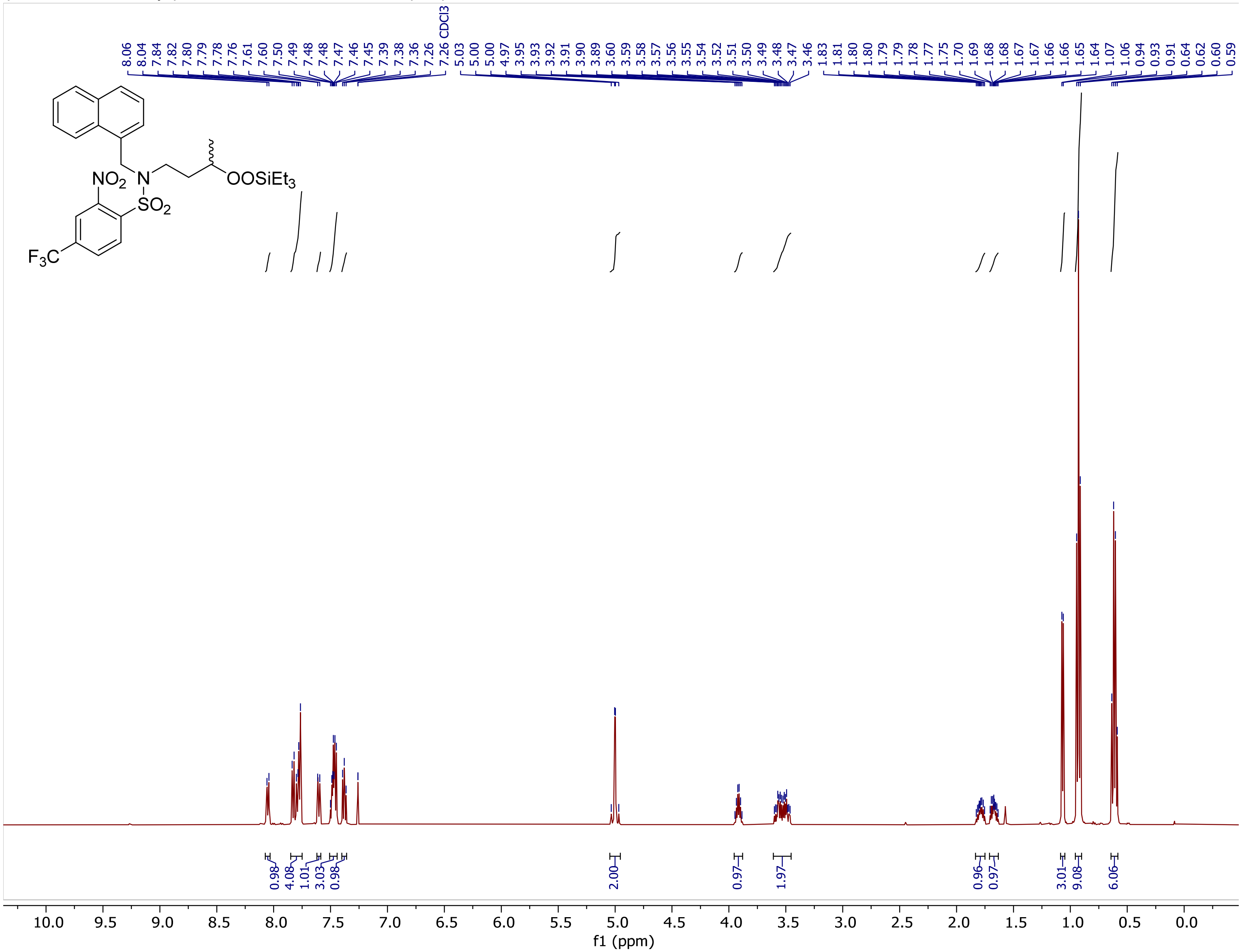

$^{13}C\ \{^{1}H\}$ NMR (126 MHz, $CDCl_3$) spectrum of *N*-(Naphthalen-1-ylmethyl)-2-nitro-*N*-(3-((triethylsilyl)peroxy)butyl)-4-(trifluoromethyl)benzenesulfonamide (**19**)

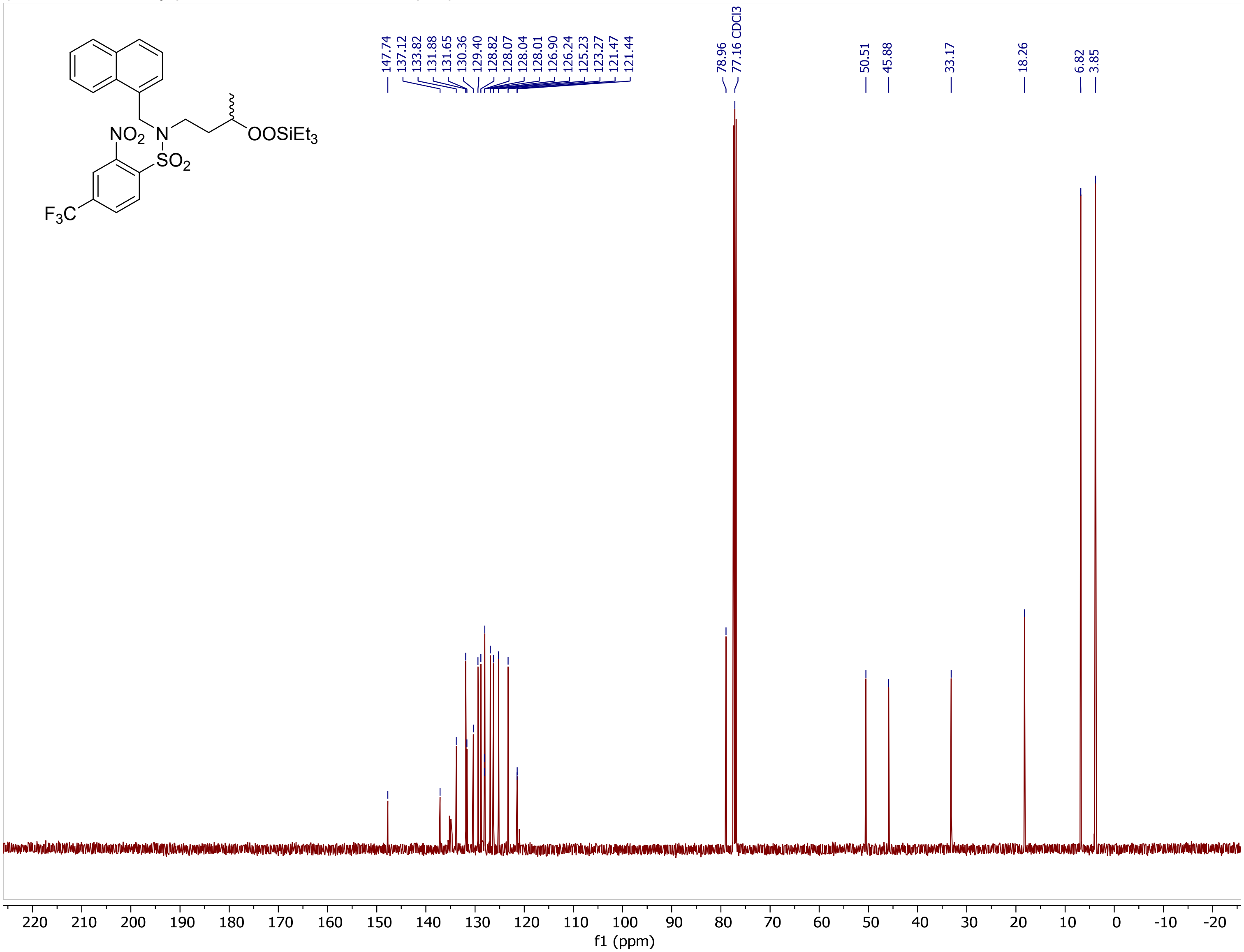

^1H NMR (500 MHz, C_7D_8) spectrum of *N*-(3-((*tert*-Butyldiphenylsilyl)peroxy)butyl)-*N*-(naphthalen-1-ylmethyl)-2-nitro-4-(trifluoromethyl)benzenesulfonamide (**24**)

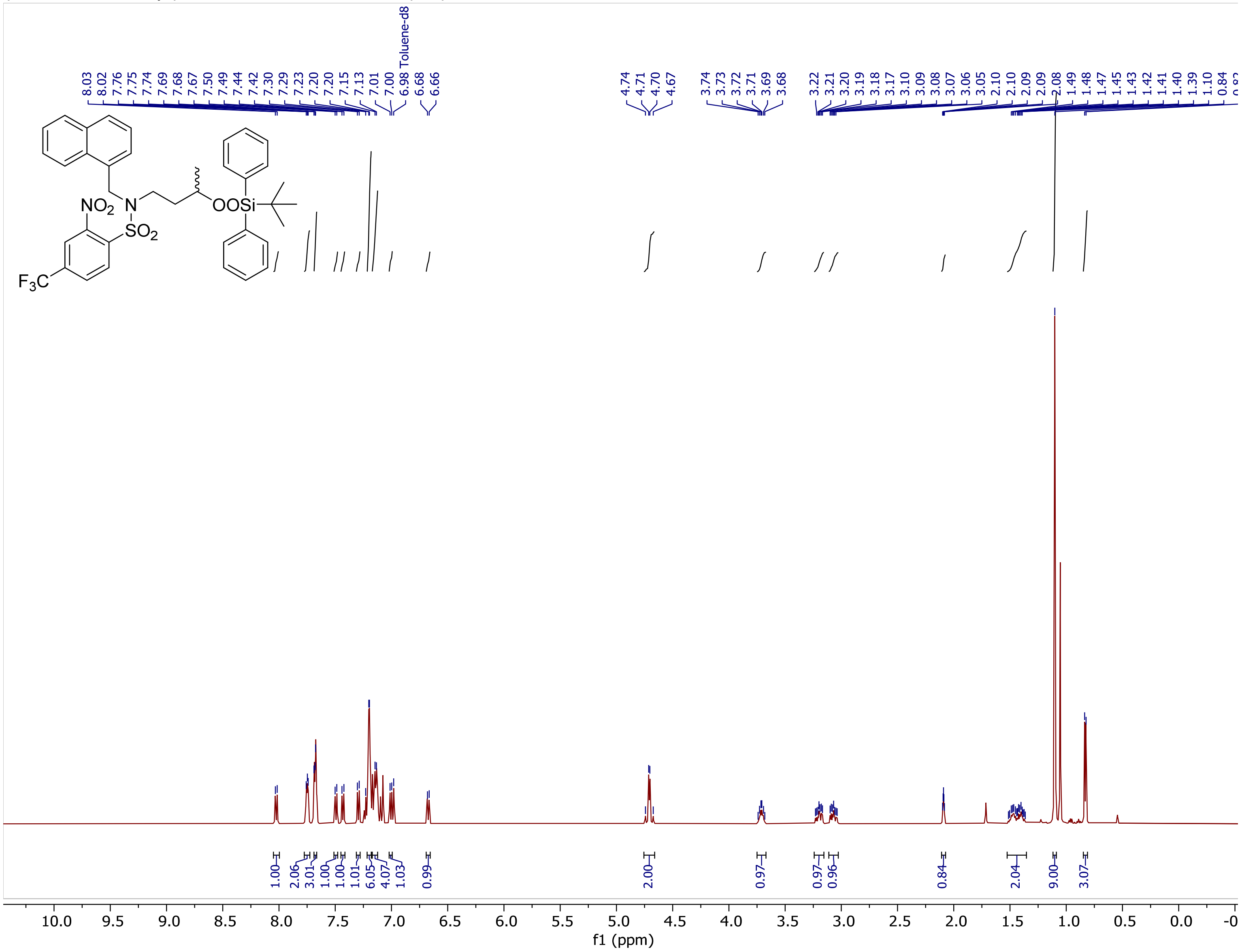

^{13}C {^{1}H} NMR (126 MHz, C_7D_8) spectrum of *N*-(3-((*tert*-Butyldiphenylsilyl)peroxy)butyl)-*N*-(naphthalen-1-ylmethyl)-2-nitro-4-(trifluoromethyl)benzenesulfonamide (**24**)

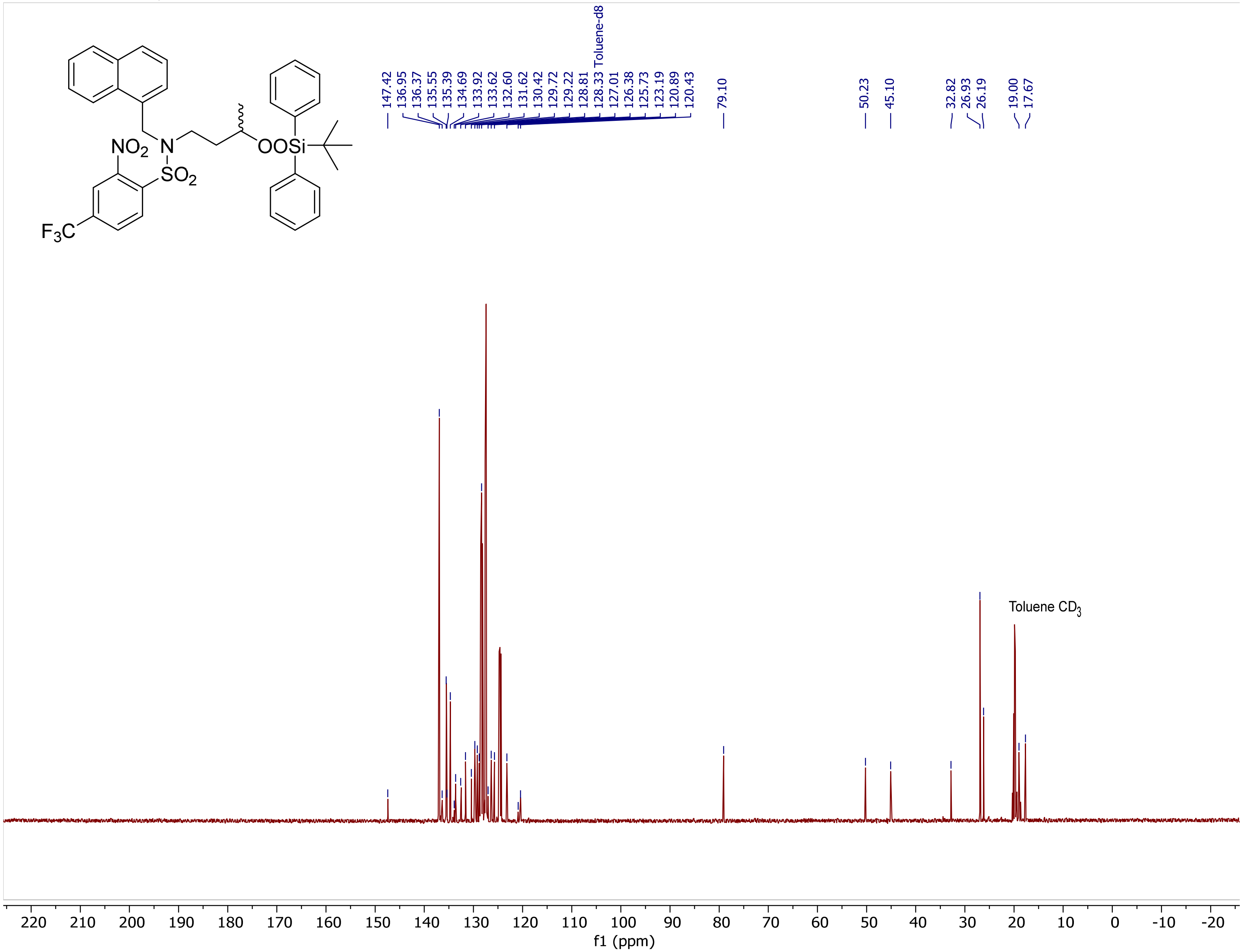

^{1}H NMR (500 MHz, C_6D_6) spectrum of 5-Methyl-2-(naphthalen-1-ylmethyl)isoxazolidine (**29**)

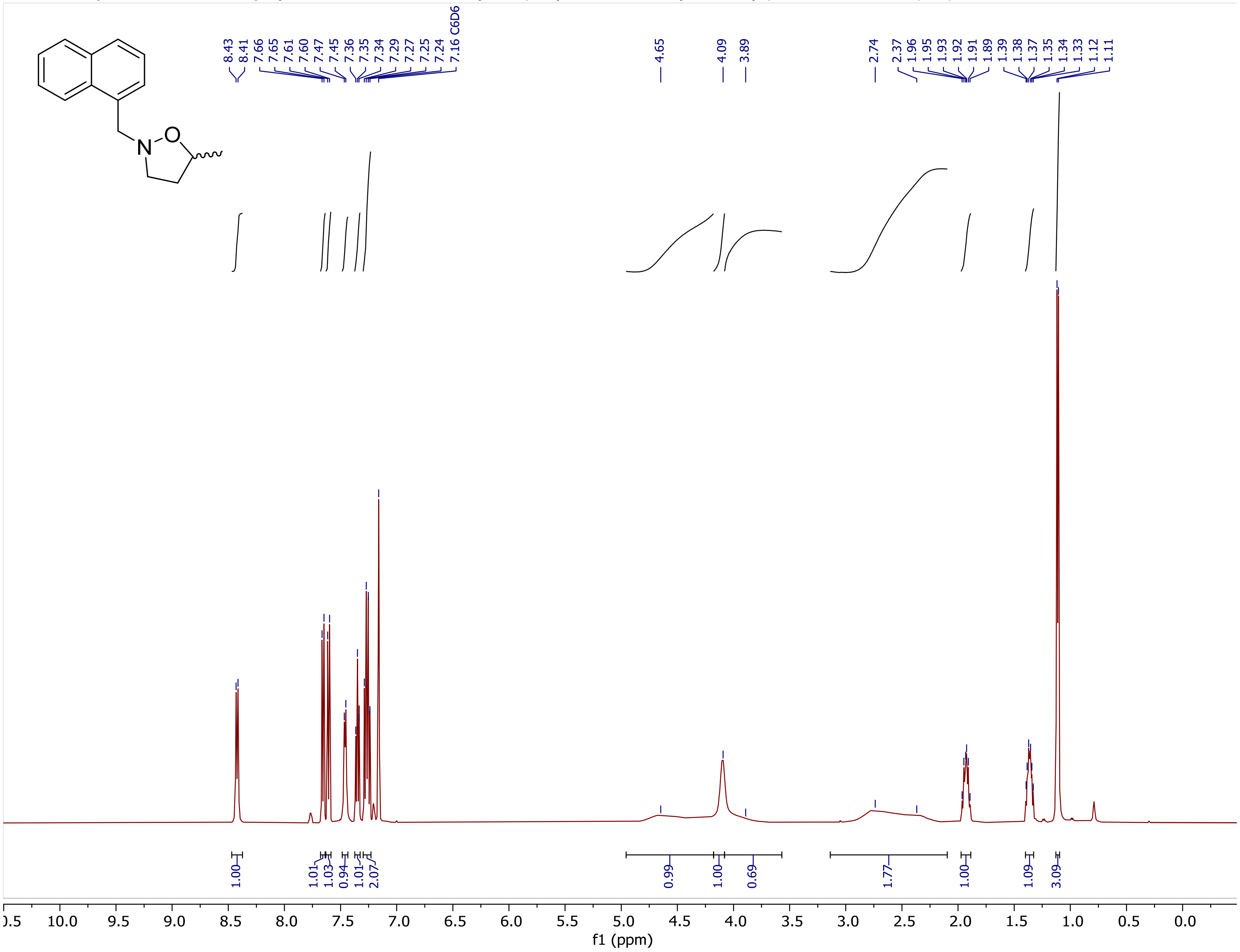

^{1}H NMR (500 MHz, C_6D_6) spectrum of 5-Methyl-2-(naphthalen-1-ylmethyl)isoxazolidine at 70 °C (**29**)

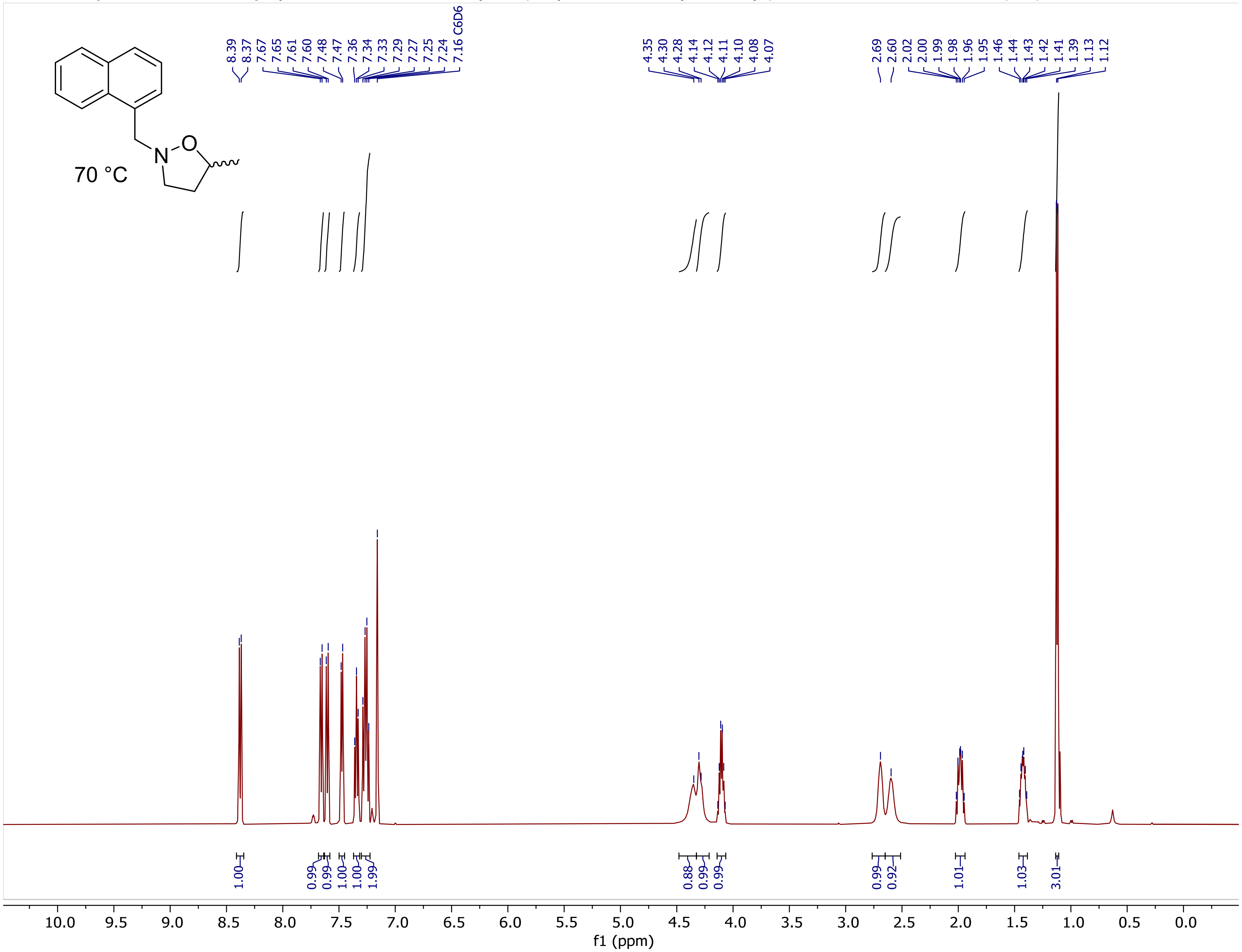

^{13}C {^{1}H} NMR (126 MHz, C_6D_6) spectrum of 5-Methyl-2-(naphthalen-1-ylmethyl)isoxazolidine at 70 °C (**29**)

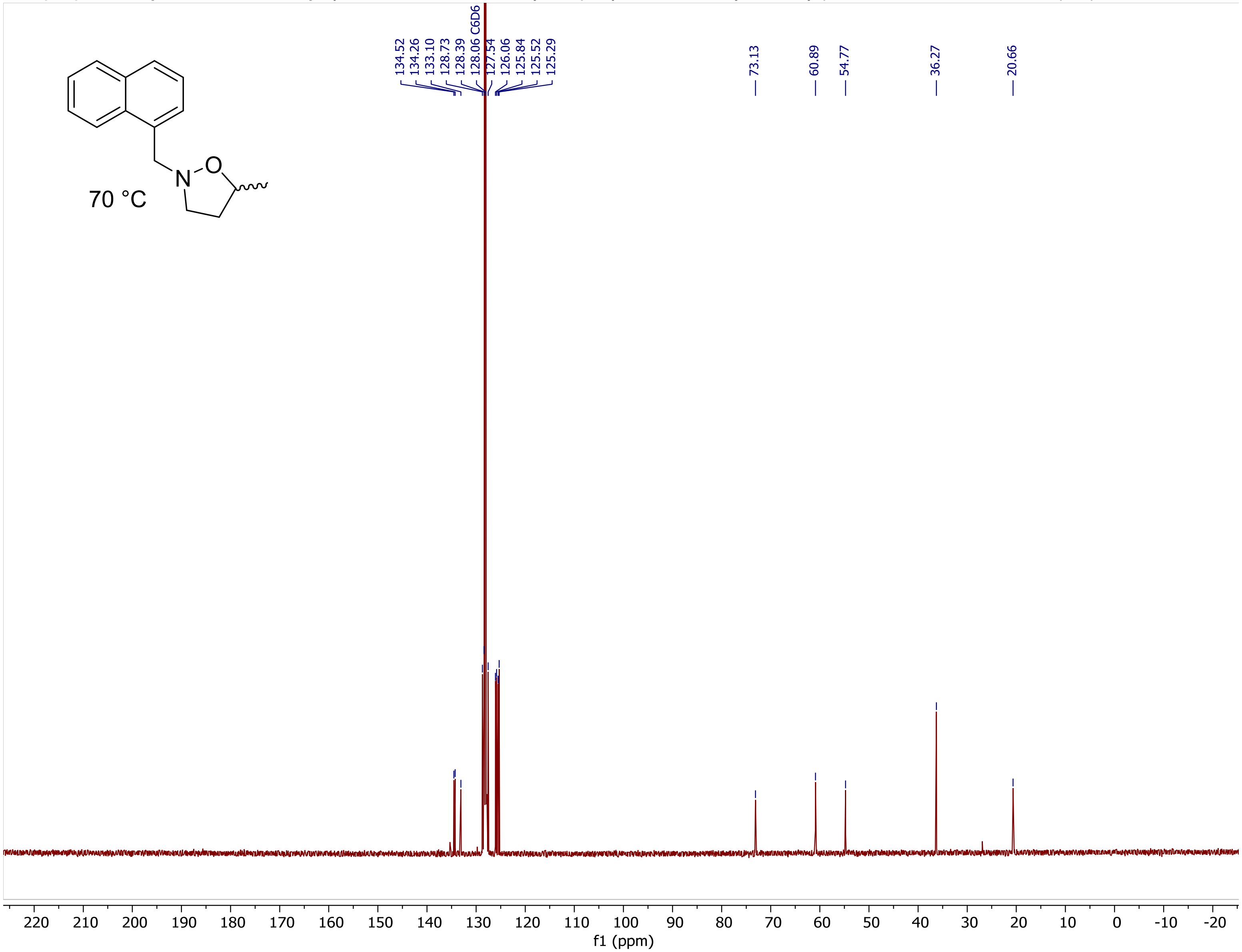

$^{13}C\{^{1}H\}$ **NMR (126 MHz, C_6D_6)** spectrum of 5-Methyl-2-(naphthalen-1-ylmethyl)isoxazolidine (**29**)

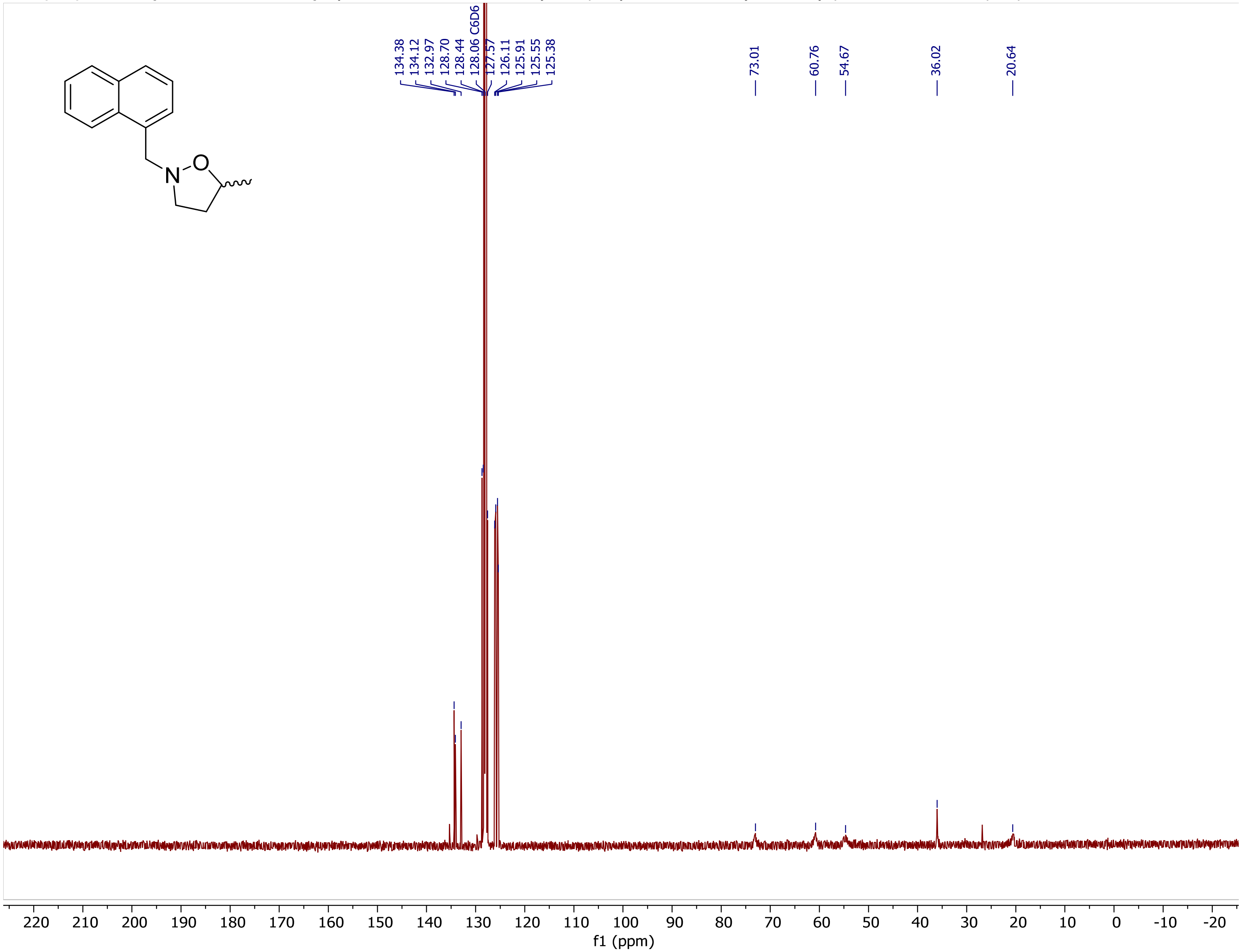

HSQC NMR (500 MHz, C_6D_6) spectrum of 5-Methyl-2-(naphthalen-1-ylmethyl)isoxazolidine at 70 °C (**29**)

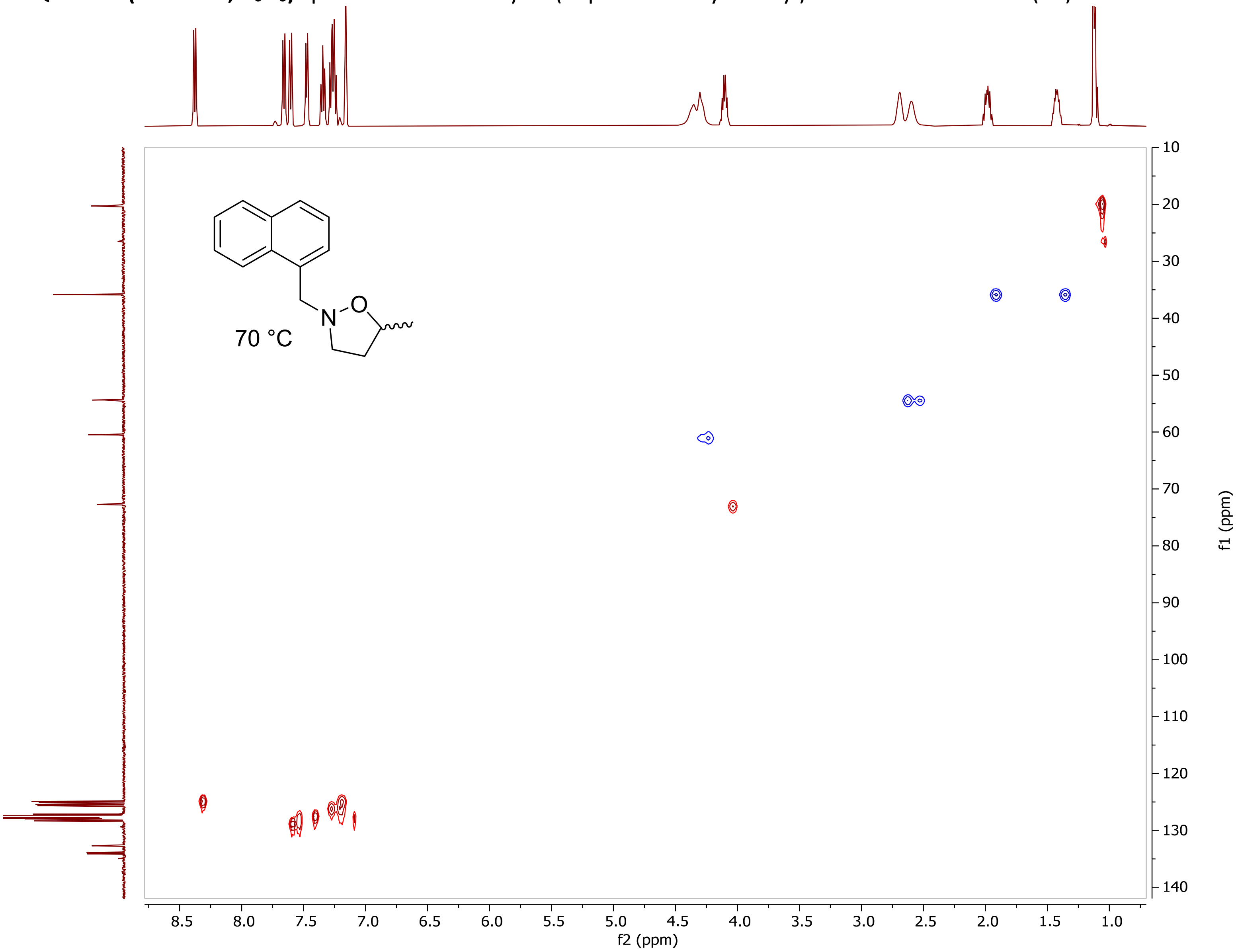

COSY NMR (500 MHz, C_6D_6) spectrum of 5-Methyl-2-(naphthalen-1-ylmethyl)isoxazolidine at 70 °C (**29**)

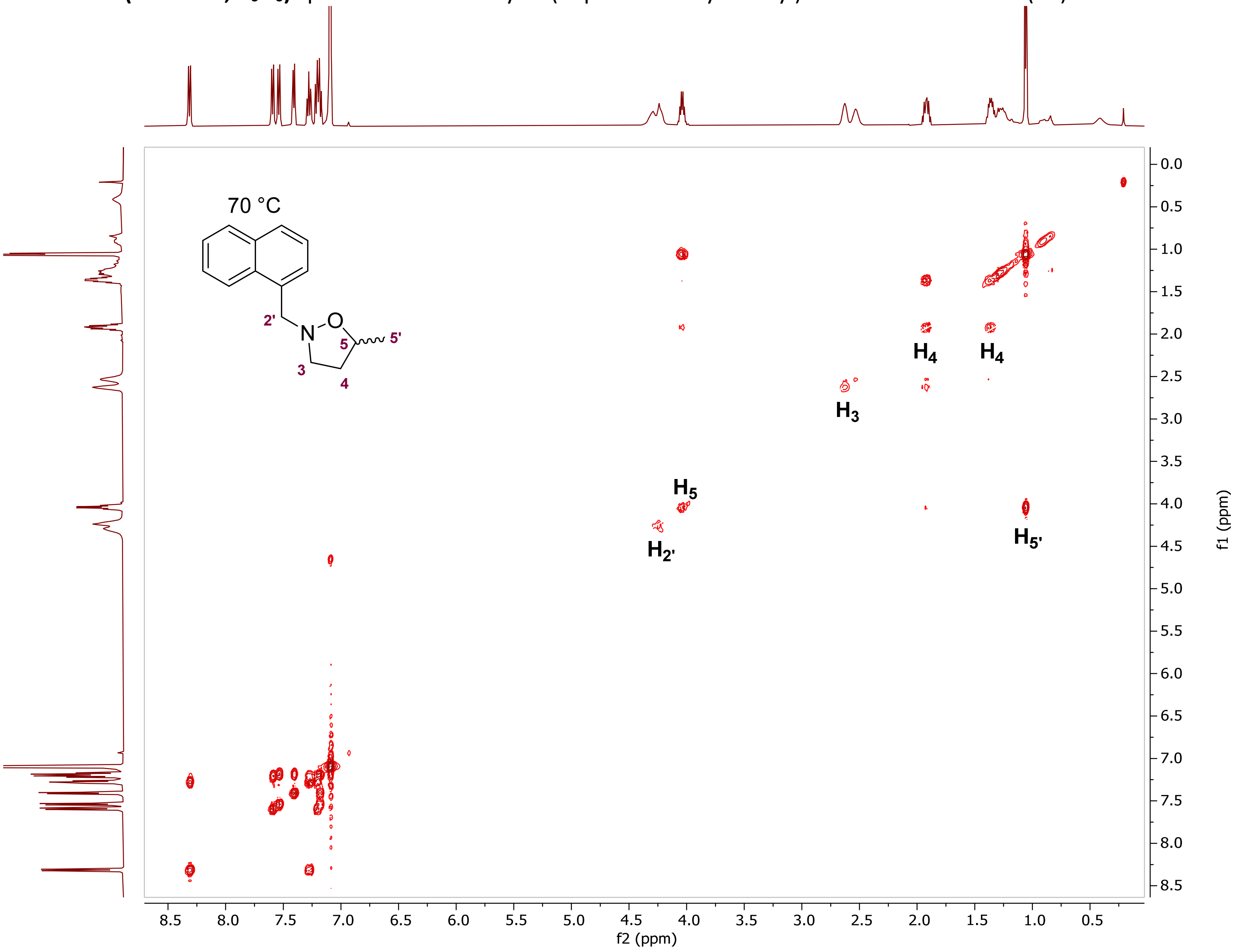

^{1}H NMR (500 MHz, $CDCl_3$) spectrum of *N*-(4-Fluorobenzyl)-2-nitro-4-(trifluoromethyl)benzenesulfonamide (**S4**)

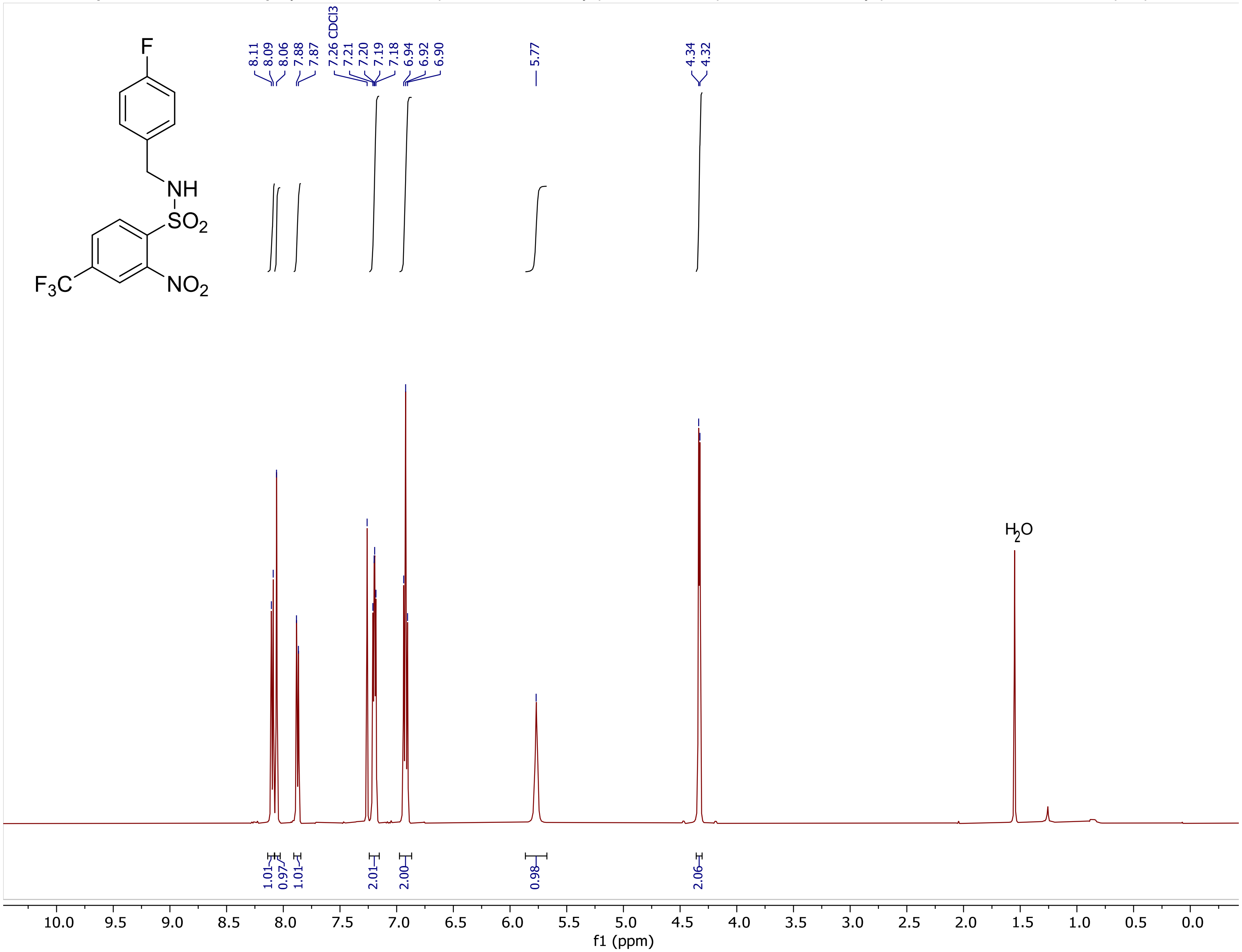

^{13}C {^{1}H, ^{19}F} NMR (126 MHz, $CDCl_3$) spectrum of *N*-(4-Fluorobenzyl)-2-nitro-4-(trifluoromethyl)benzenesulfonamide (**S4**)

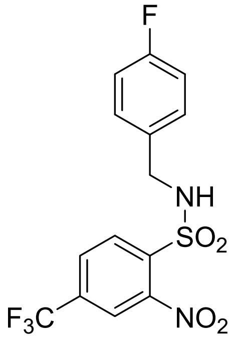

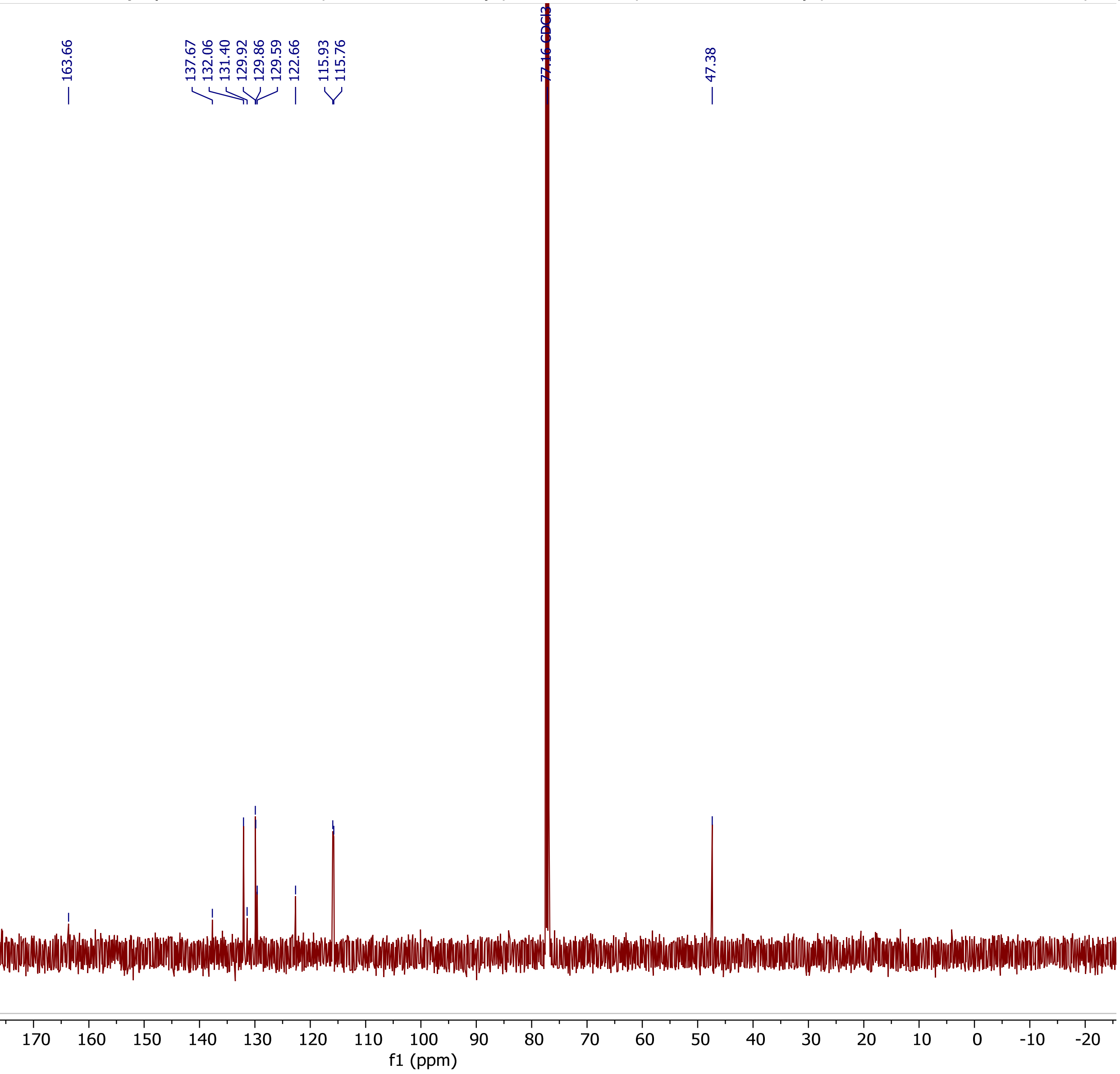

^{1}H NMR (500 MHz, CDCl$_3$) spectrum of *N*-(But-3-en-1-yl)-*N*-(4-fluorobenzyl)-2-nitro-4-(trifluoromethyl)benzenesulfonamide (**4a**)

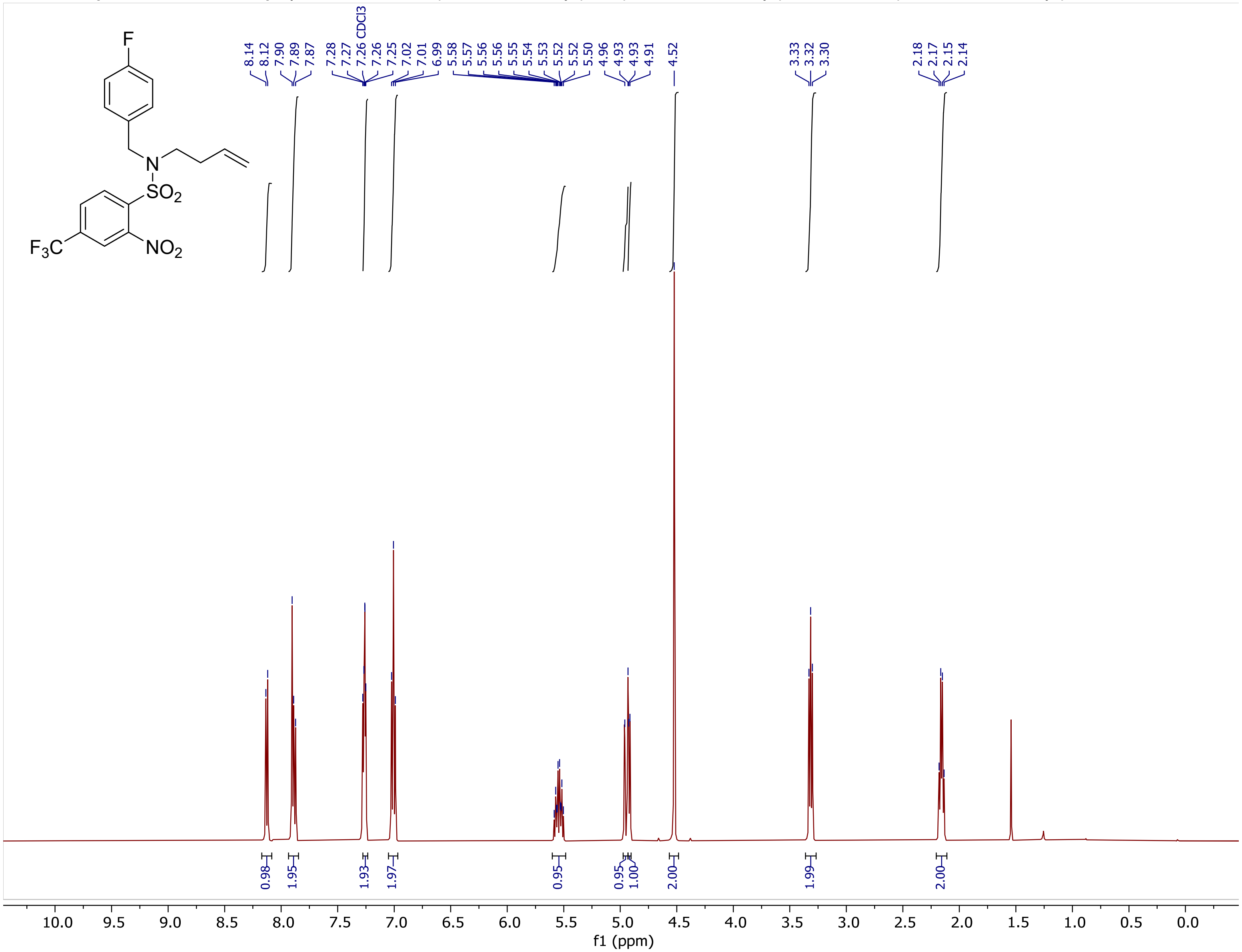

^{13}C {^{1}H} NMR (126 MHz, $CDCl_3$) spectrum of *N*-(But-3-en-1-yl)-*N*-(4-fluorobenzyl)-2-nitro-4-(trifluoromethyl)benzenesulfonamide (**4a**)

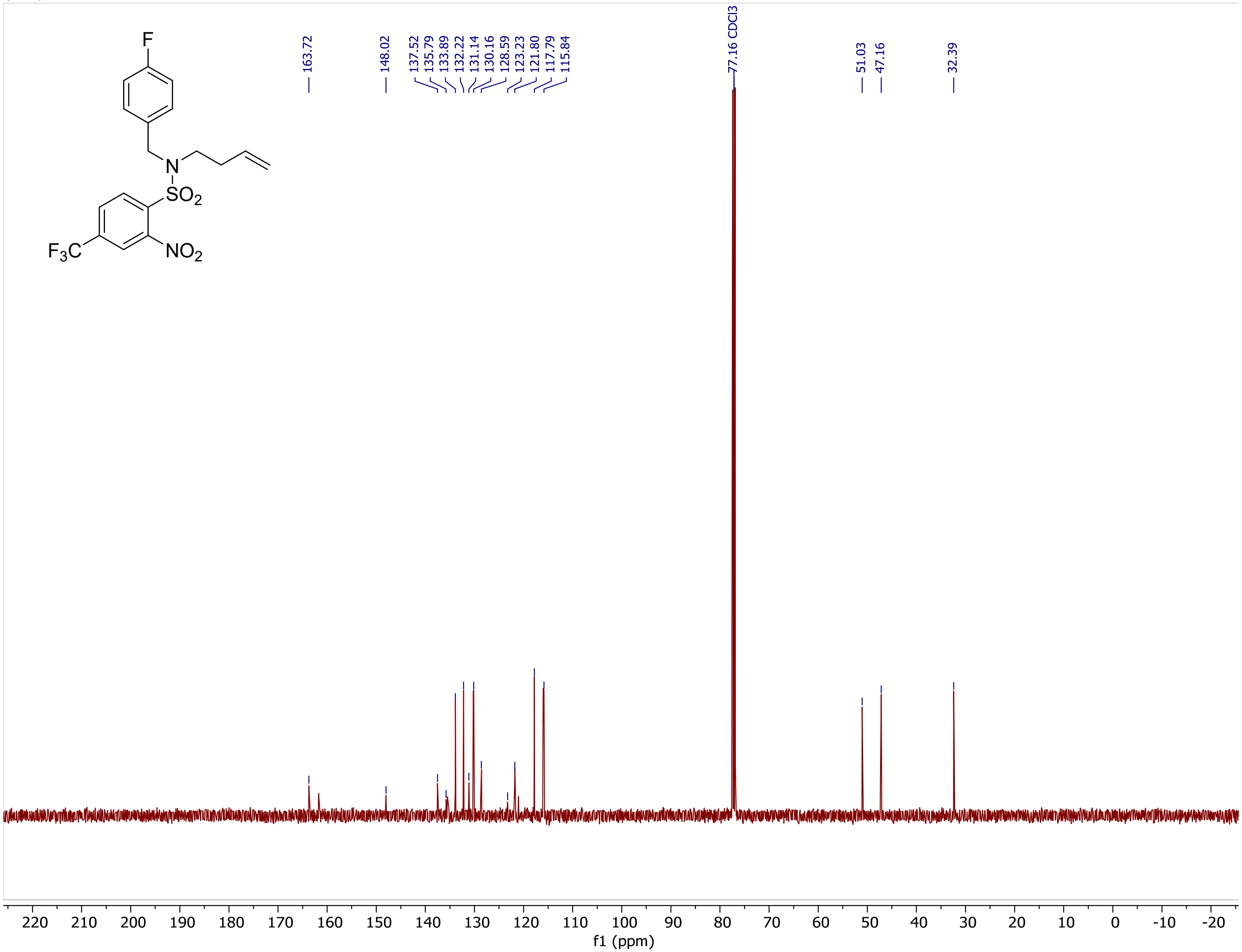

^{19}F NMR (470 MHz, $CDCl_3$) spectrum of *N*-(But-3-en-1-yl)-*N*-(4-fluorobenzyl)-2-nitro-4-(trifluoromethyl)benzenesulfonamide (**4a**)

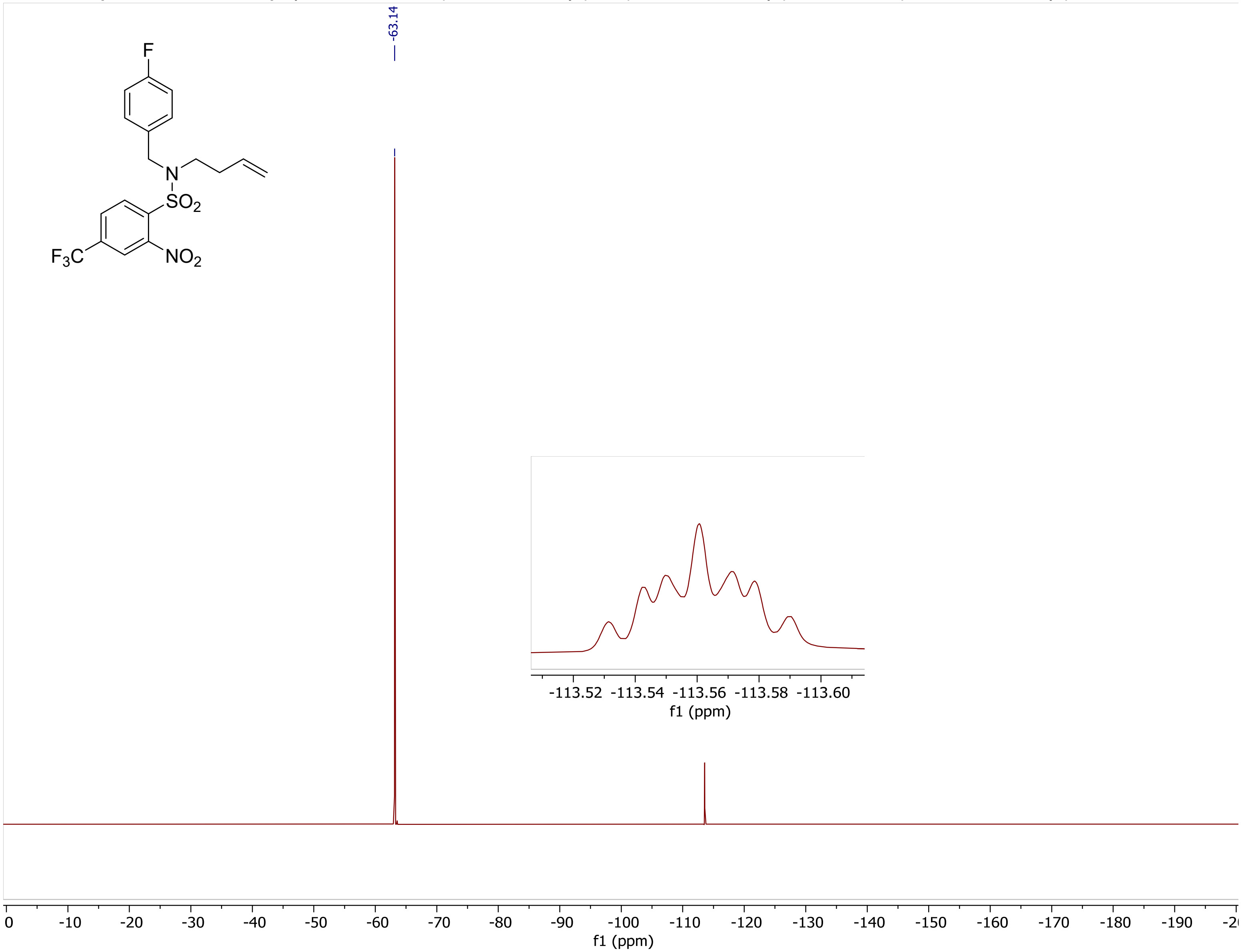

^{1}H NMR (500 MHz, CDCl$_3$) spectrum of *N*-(4-Fluorobenzyl)-2-nitro-*N*-(3-((triethylsilyl)peroxy)butyl)-4-(trifluoromethyl)benzenesulfonamide (**4b**)

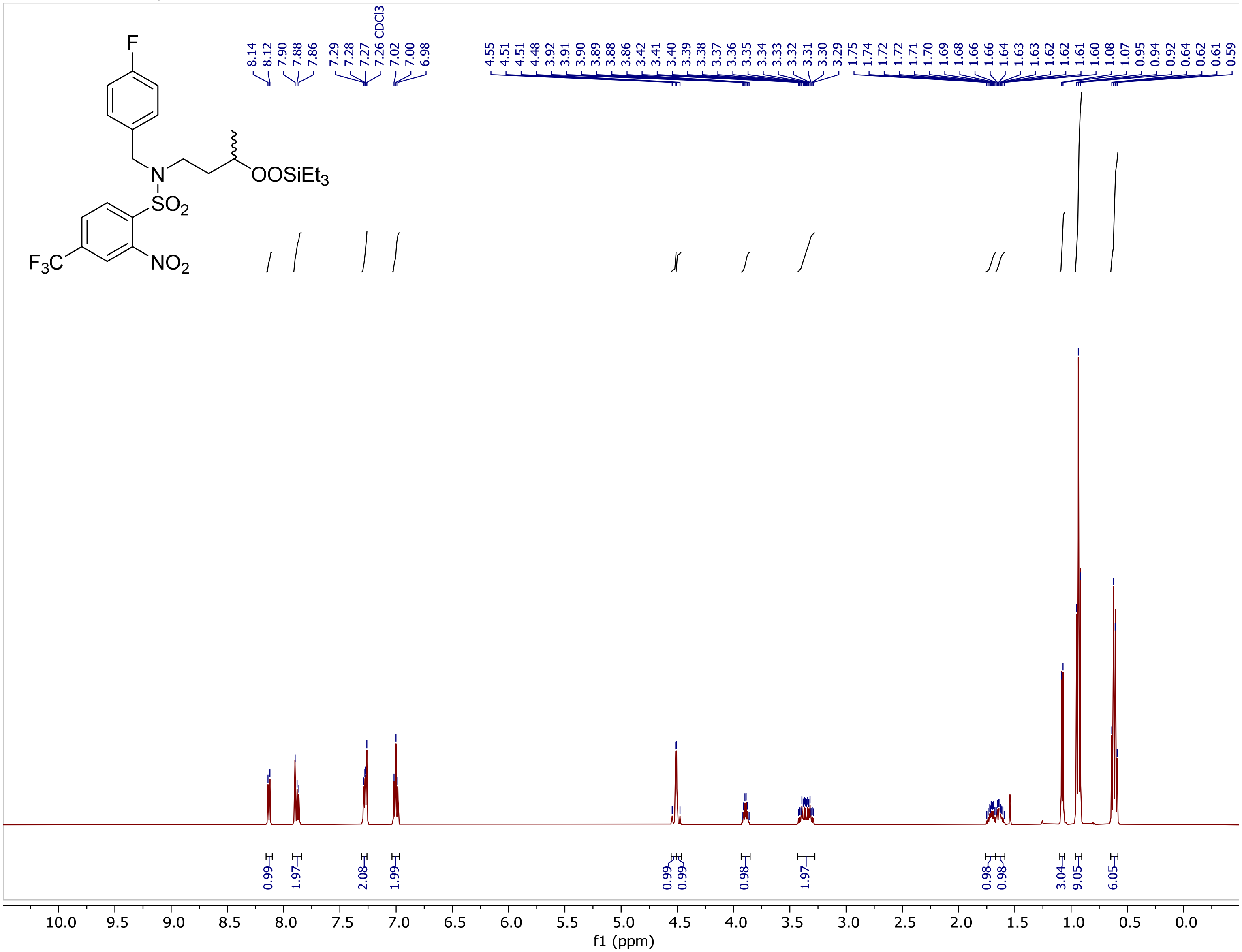

^{13}C {^{1}H} NMR (126 MHz, $CDCl_3$) spectrum of *N*-(4-Fluorobenzyl)-2-nitro-*N*-(3-((triethylsilyl)peroxy)butyl)-4-(trifluoromethyl)benzenesulfonamide (**4b**)

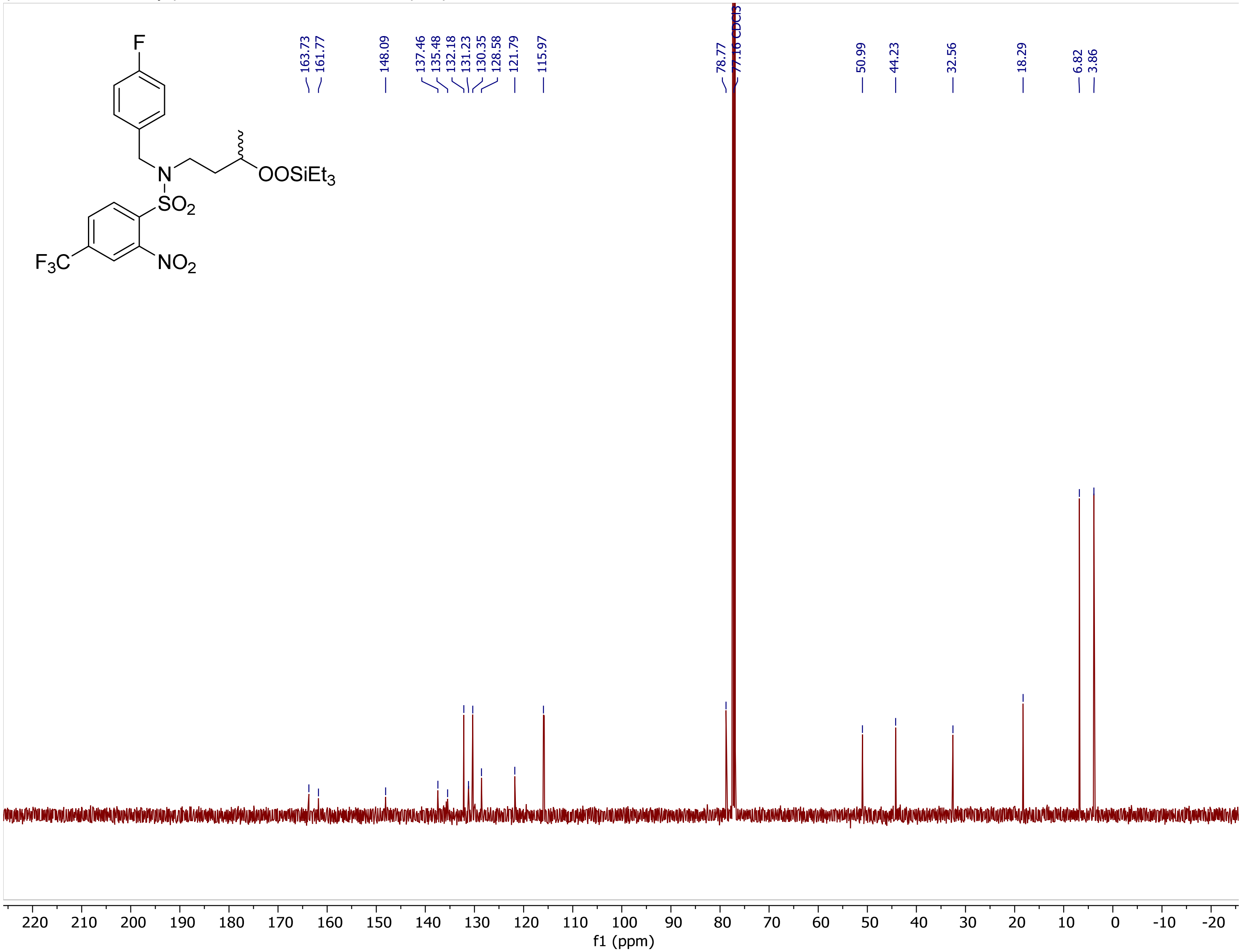

^{1}H NMR (500 MHz, C_6D_6) spectrum of *N*-(3-((*tert*-Butyldiphenylsilyl)peroxy)butyl)-*N*-(4-fluorobenzyl-2-nitro-4-(trifluoromethyl)benzenesulfonamide (**34**)

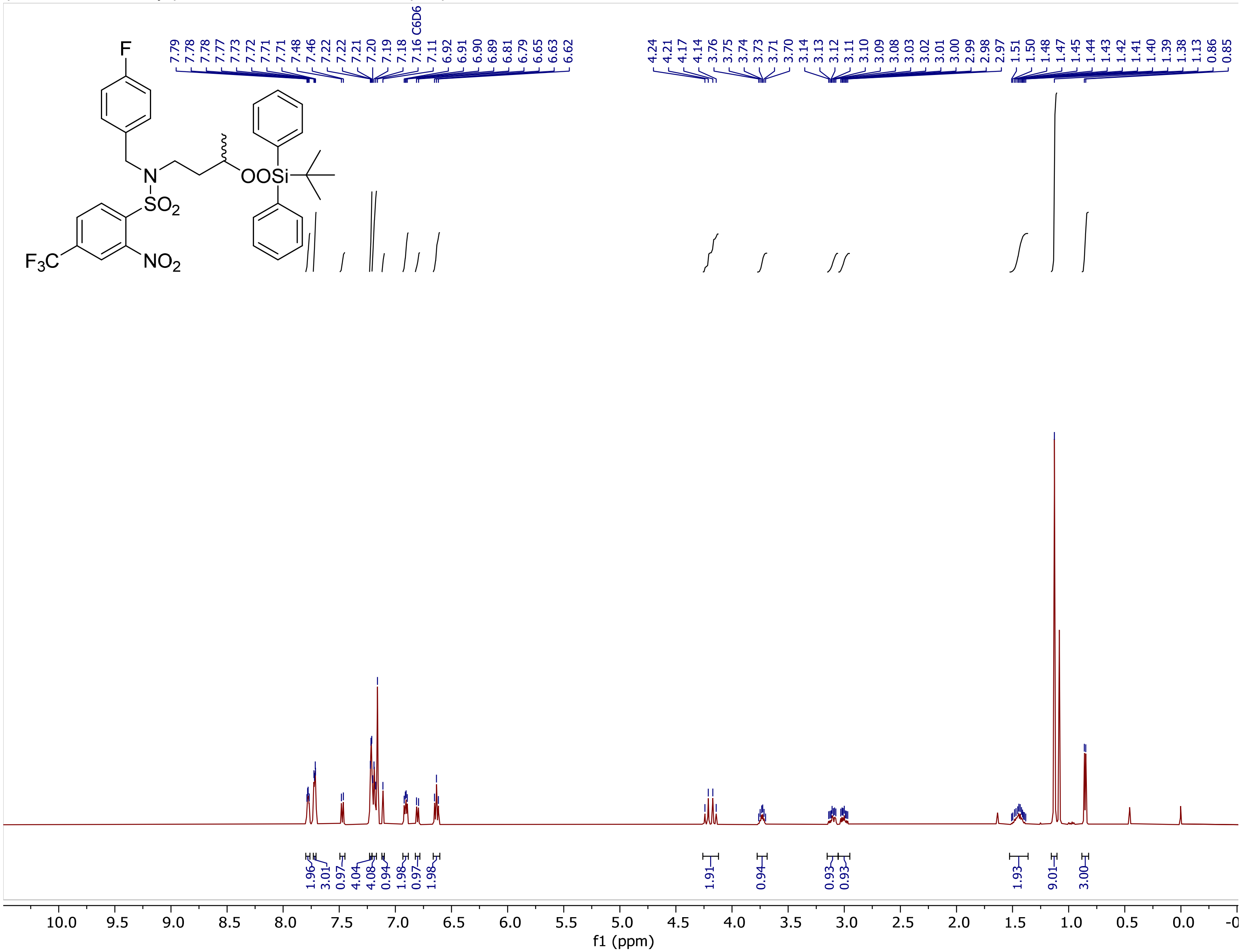

^{13}C {^{1}H} NMR (126 MHz, C_6D_6) spectrum of *N*-(3-((*tert*-Butyldiphenylsilyl)peroxy)butyl)-*N*-(4-fluorobenzyl-2-nitro-4-(trifluoromethyl)benzenesulfonamide (**34**)

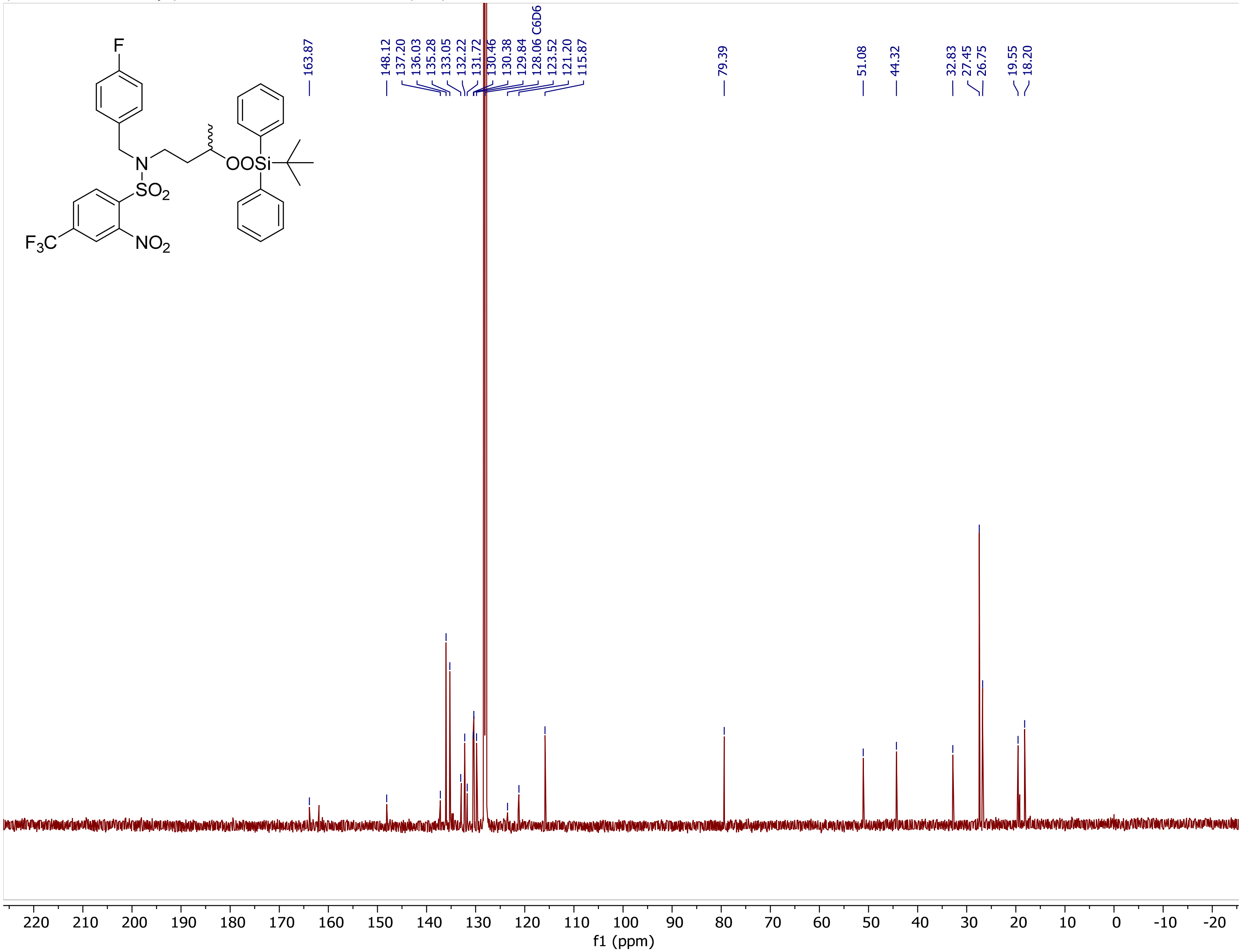

^{1}H NMR (500 MHz, C_6D_6) spectrum of 2-(4-Fluorobenzyl)-5-methylisoxazolidine (**35**)

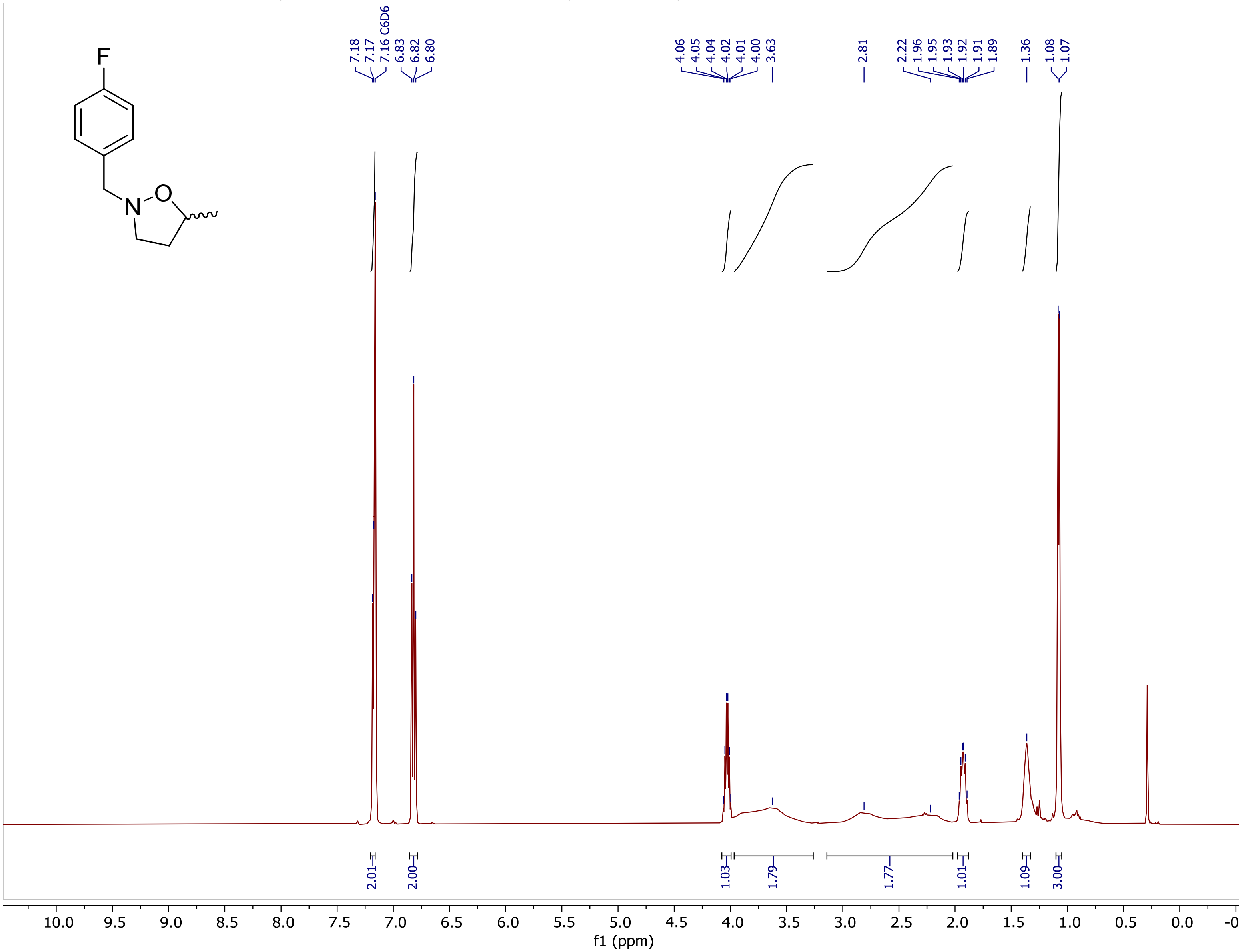

^{1}H NMR (500 MHz, C_6D_6) spectrum of 2-(4-Fluorobenzyl)-5-methylisoxazolidine at 65 °C (**35**)

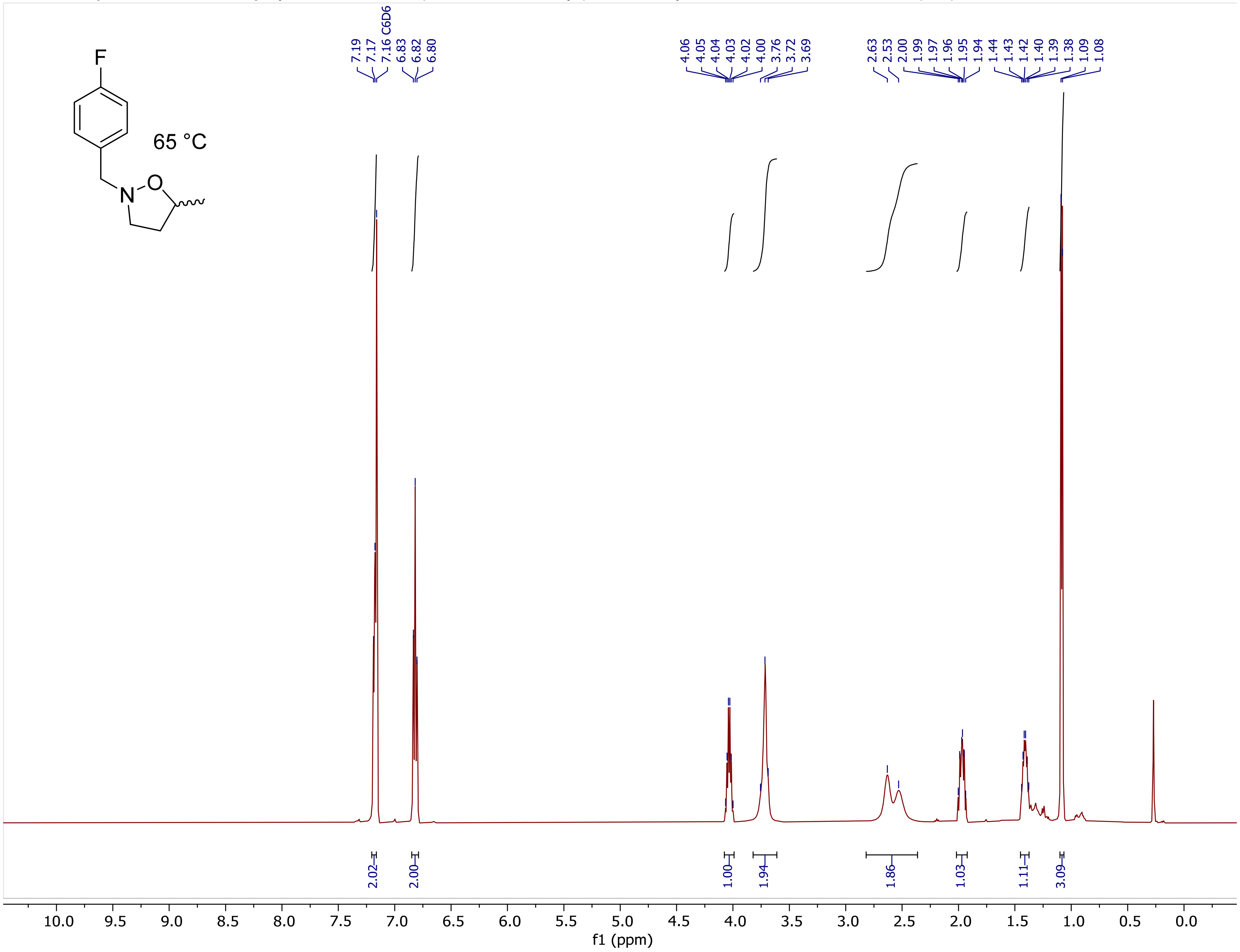

^{13}C {^{1}H} NMR (126 MHz, C_6D_6) spectrum of 2-(4-Fluorobenzyl)-5-methylisoxazolidine at 65 °C (**35**)

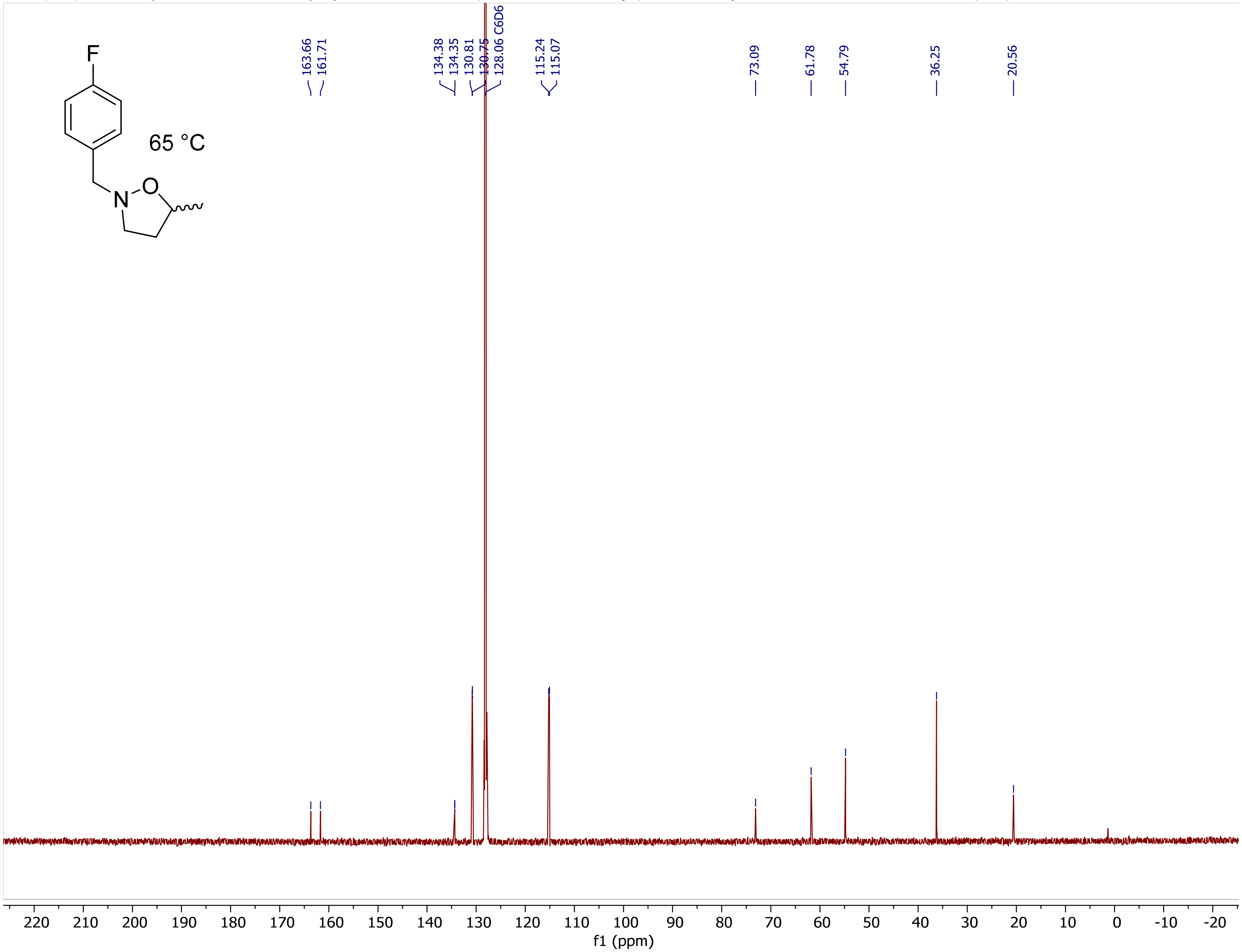

^{19}F NMR (470 MHz, C_6D_6) spectrum of 2-(4-Fluorobenzyl)-5-methylisoxazolidine (**35**)

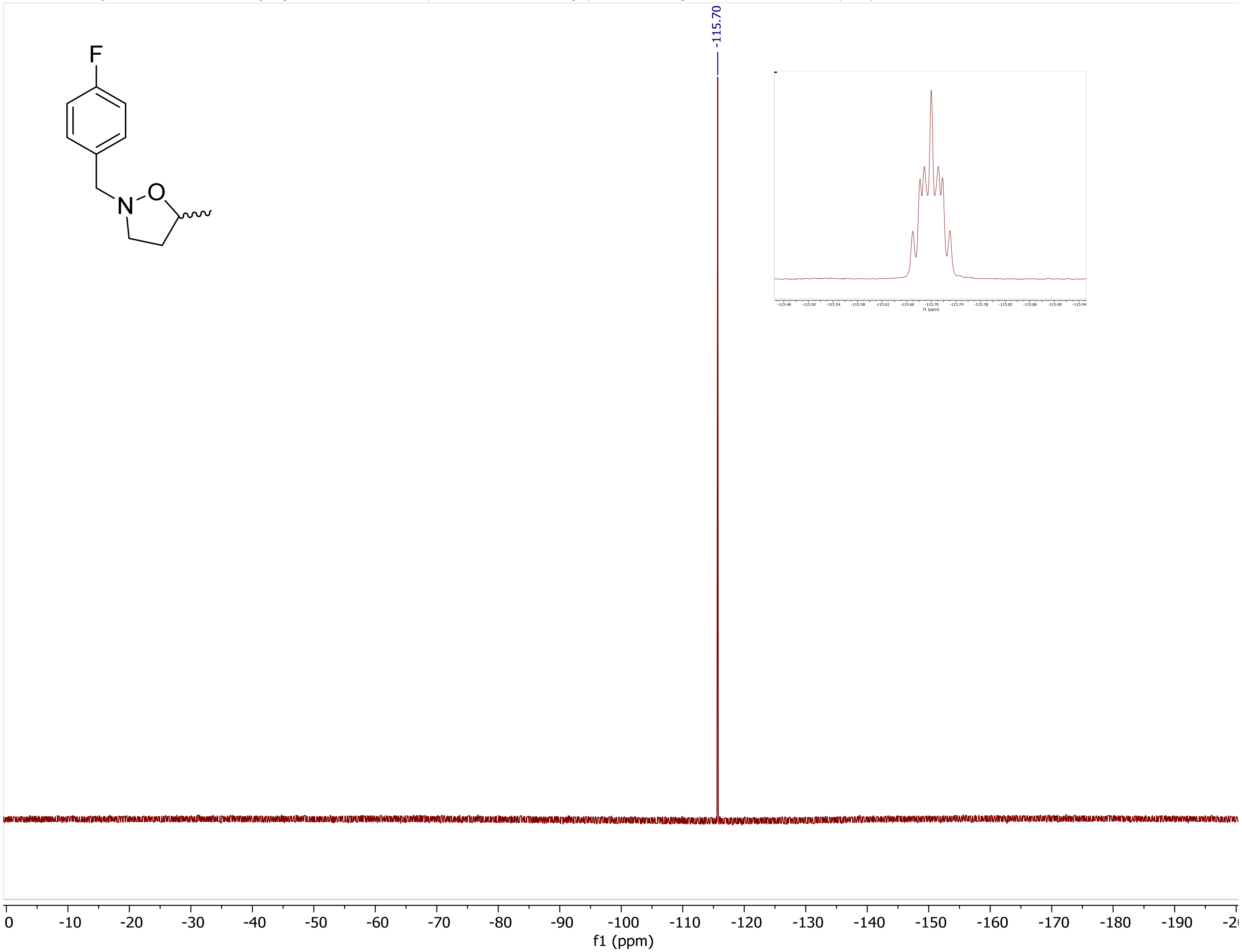

HSQC NMR (500 MHz, C_6D_6) spectrum of 2-(4-Fluorobenzyl)-5-methylisoxazolidine at 65 °C (**35**)

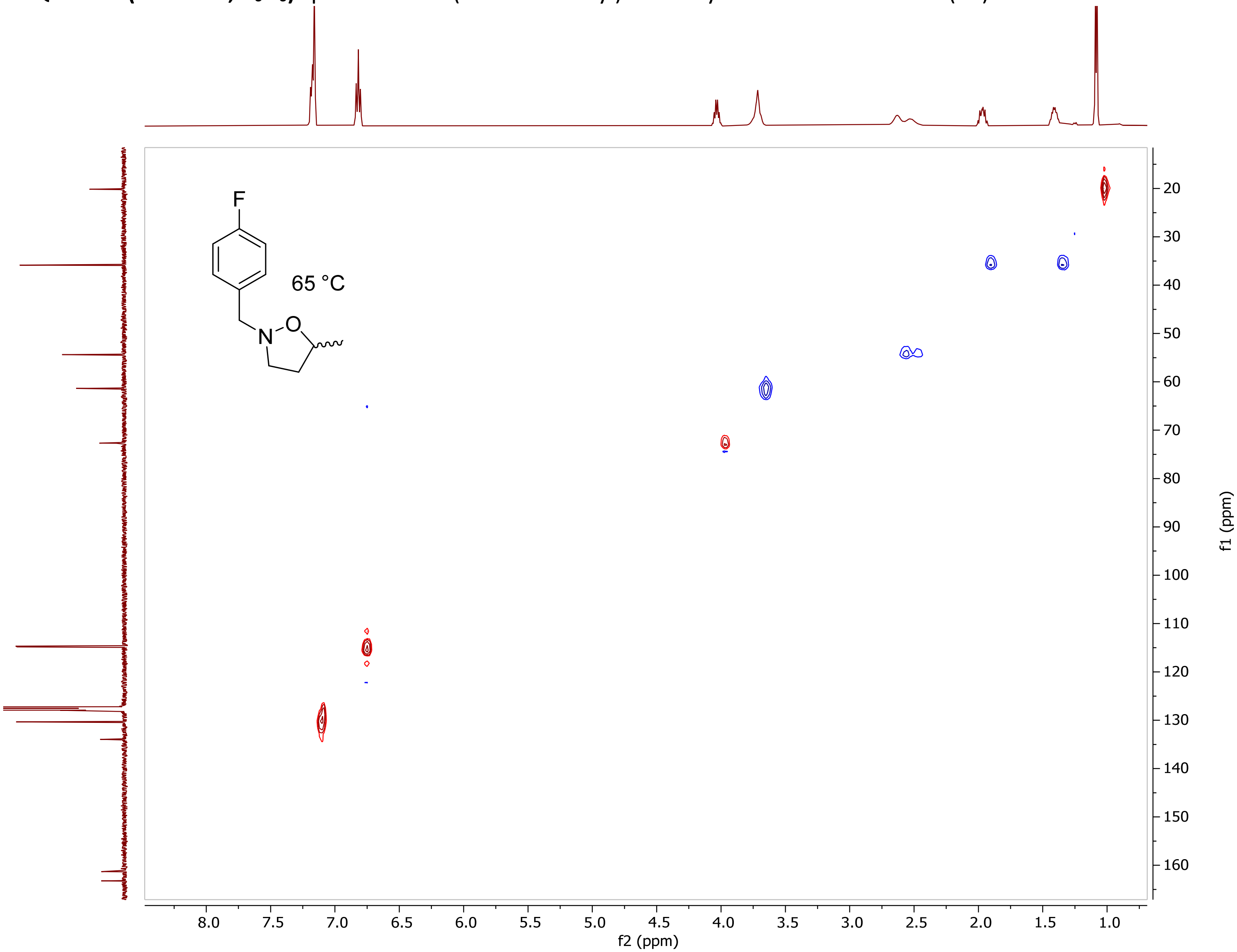

^{1}H NMR (500 MHz, CDCl$_3$) spectrum of *N*-(4-Methoxyphenethyl)-2-nitro-4-(trifluoromethyl)benzenesulfonamide (**S5**)

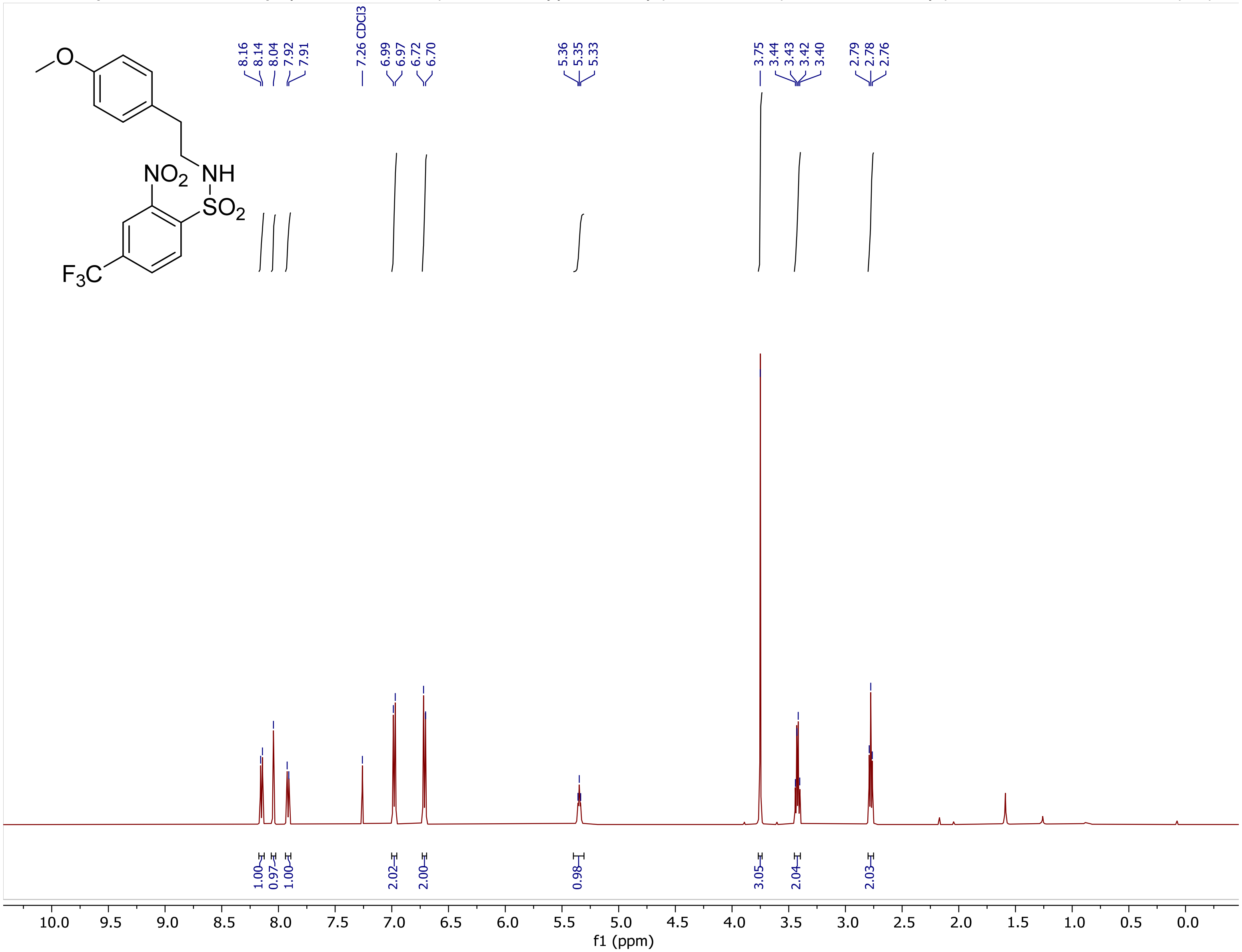

$^{13}C\ \{^1H\}$ NMR (126 MHz, $CDCl_3$) spectrum of *N*-(4-Methoxyphenethyl)-2-nitro-4-(trifluoromethyl)benzenesulfonamide (**S5**)

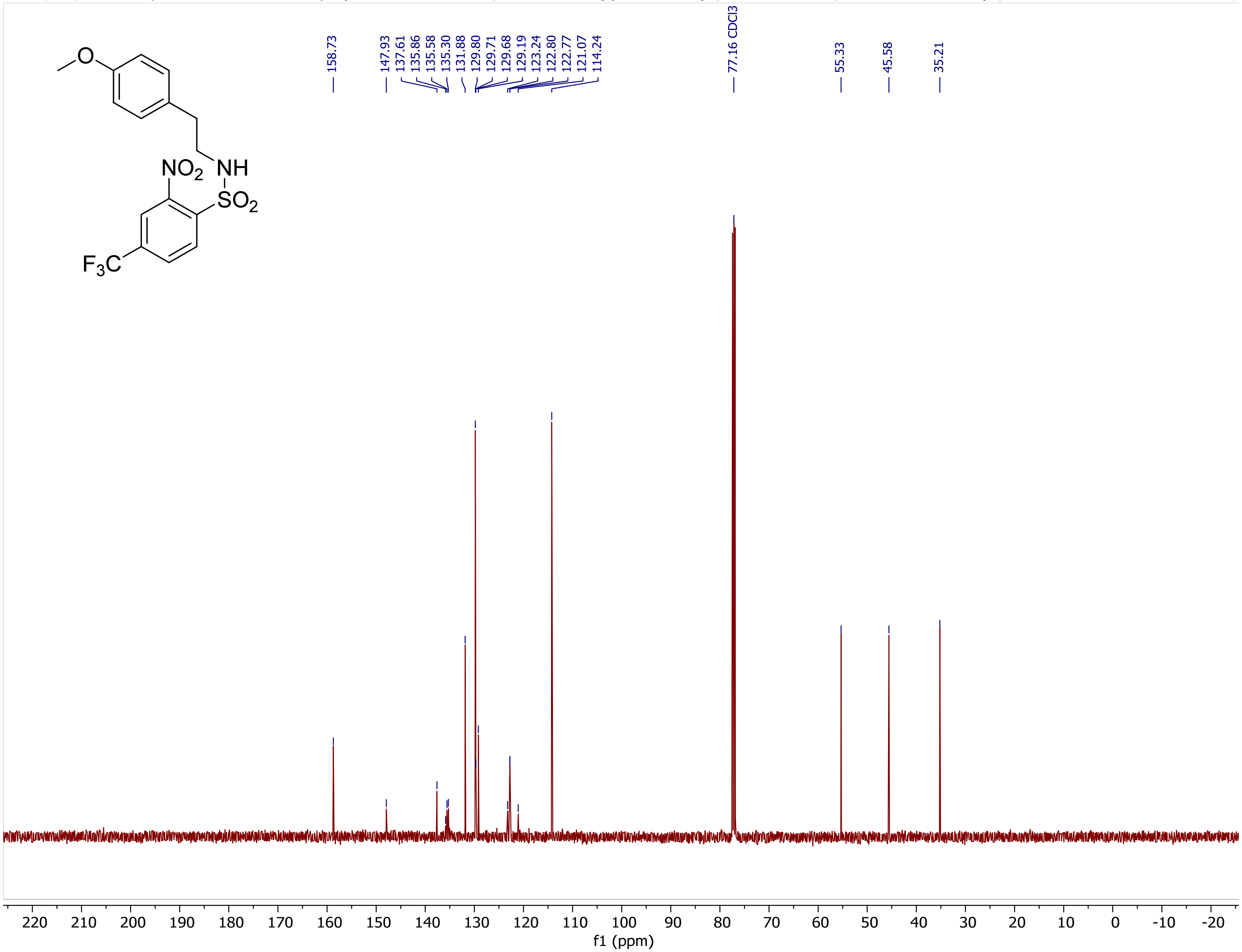

^1H NMR (500 MHz, $CDCl_3$) spectrum of *N*-(But-3-en-1-yl)-*N*-(4-methoxyphenethyl)-2-nitro-4-(trifluoromethyl)benzenesulfonamide (**5a**)

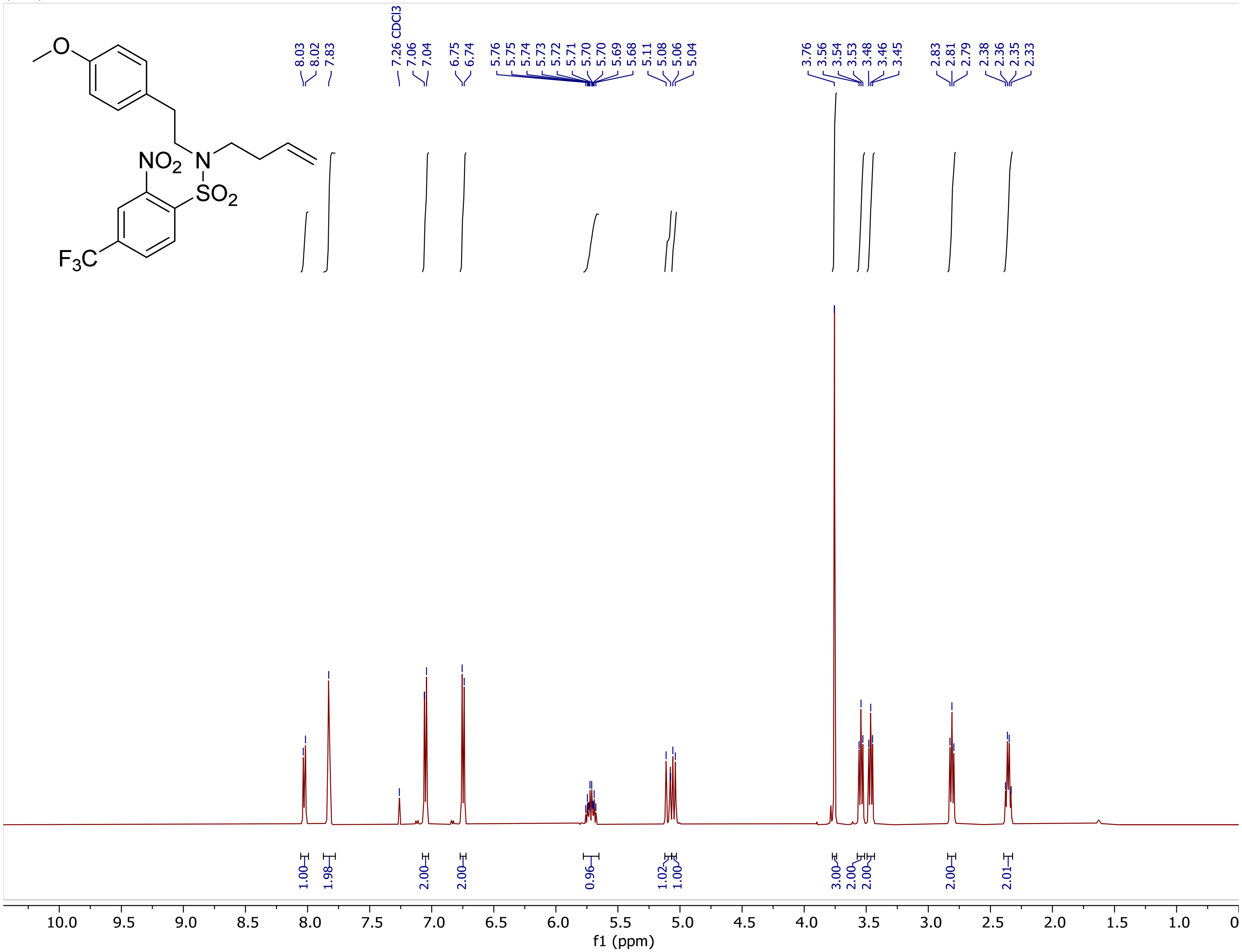

^{13}C {^{1}H} NMR (126 MHz, $CDCl_3$) spectrum of *N*-(But-3-en-1-yl)-*N*-(4-methoxyphenethyl)-2-nitro-4-(trifluoromethyl)benzenesulfonamide (**5a**)

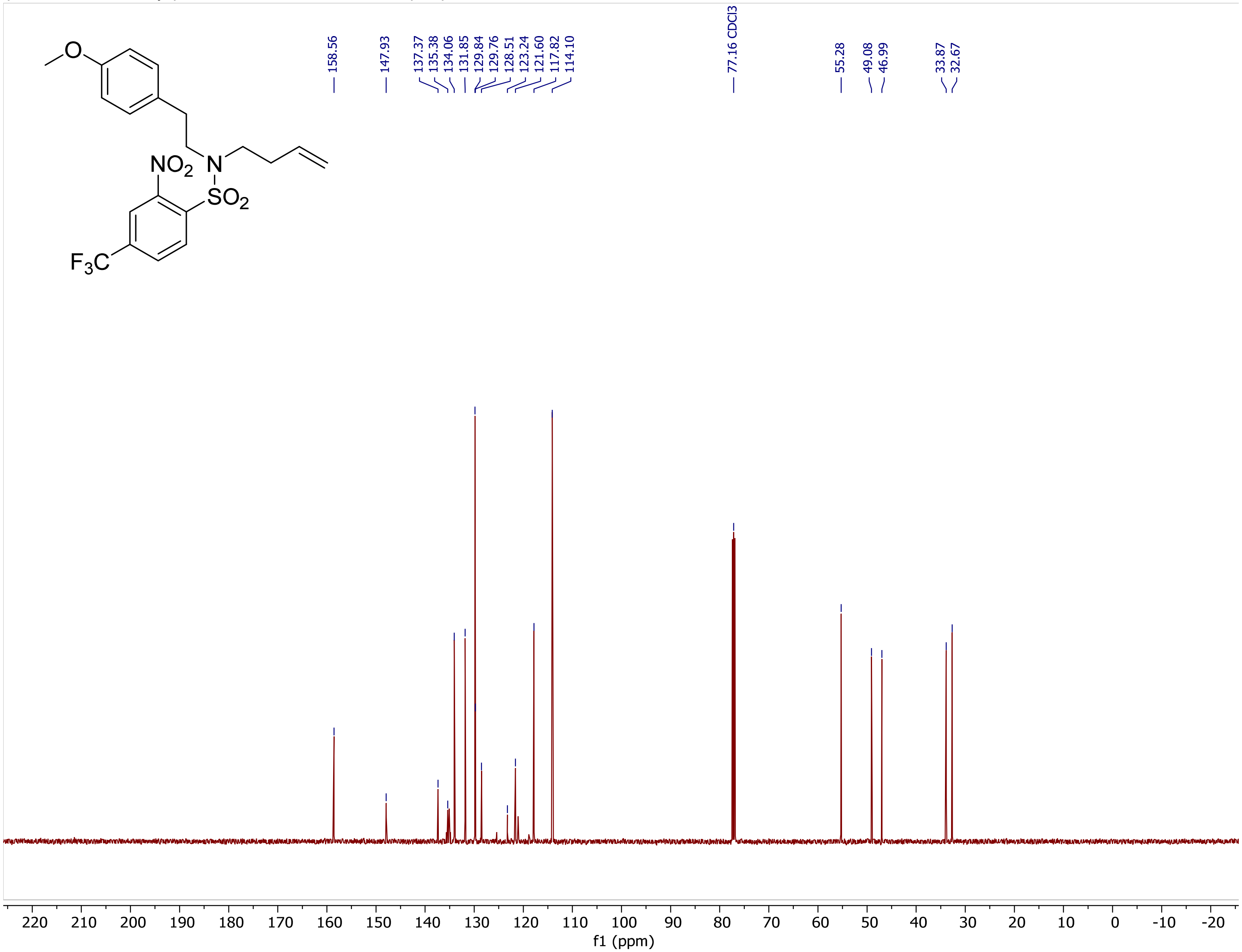

^{1}H NMR (500 MHz, $CDCl_3$) spectrum of *N*-(4-Methoxyphenethyl)-2-nitro-*N*-(3-((triethylsilyl)peroxy)butyl)-4-(trifluoromethyl)benzenesulfonamide (**5b**)

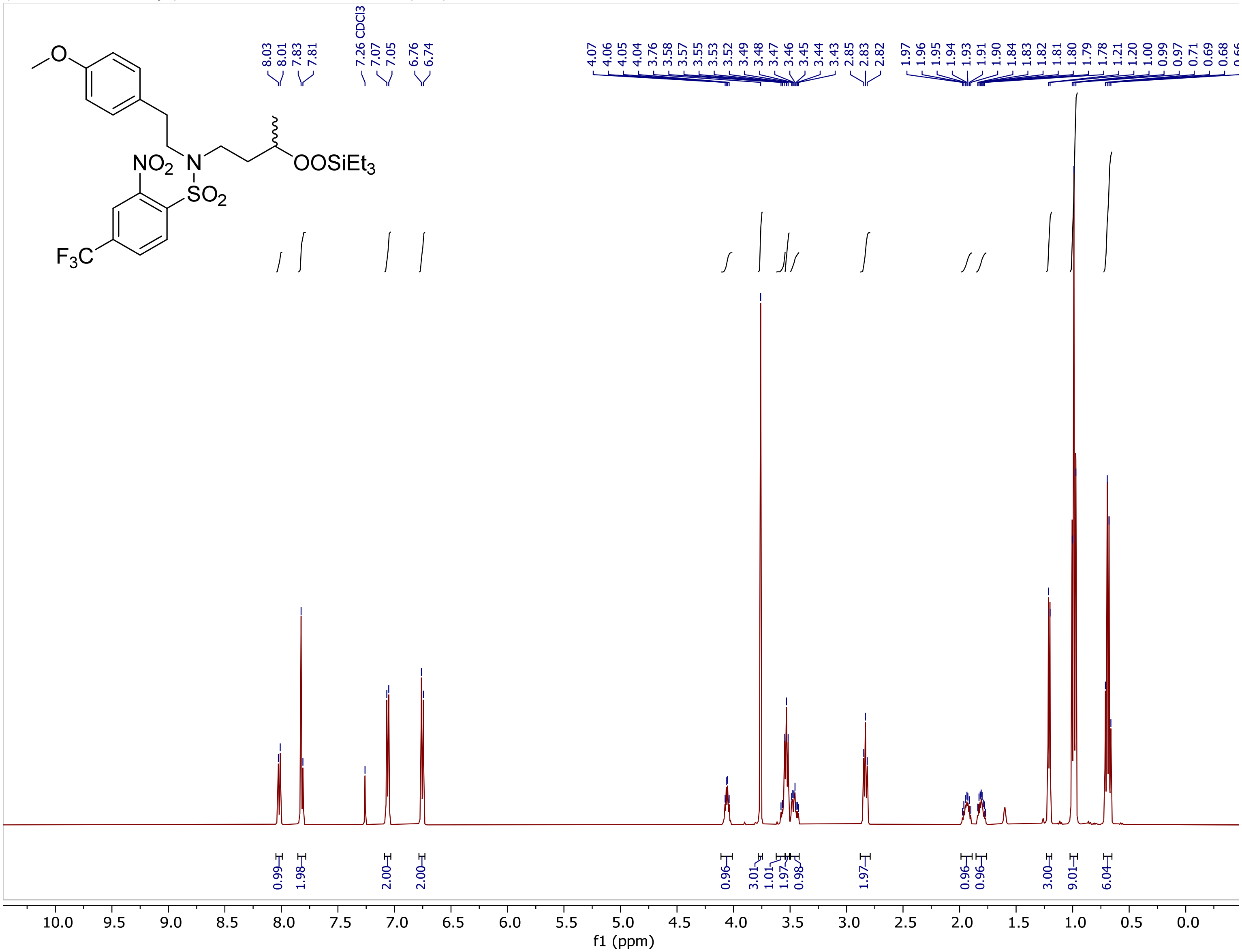

^{13}C {^{1}H} NMR (126 MHz, $CDCl_3$) spectrum of *N*-(4-Methoxyphenethyl)-2-nitro-*N*-(3-((triethylsilyl)peroxy)butyl)-4-(trifluoromethyl)benzenesulfonamide (**5b**)

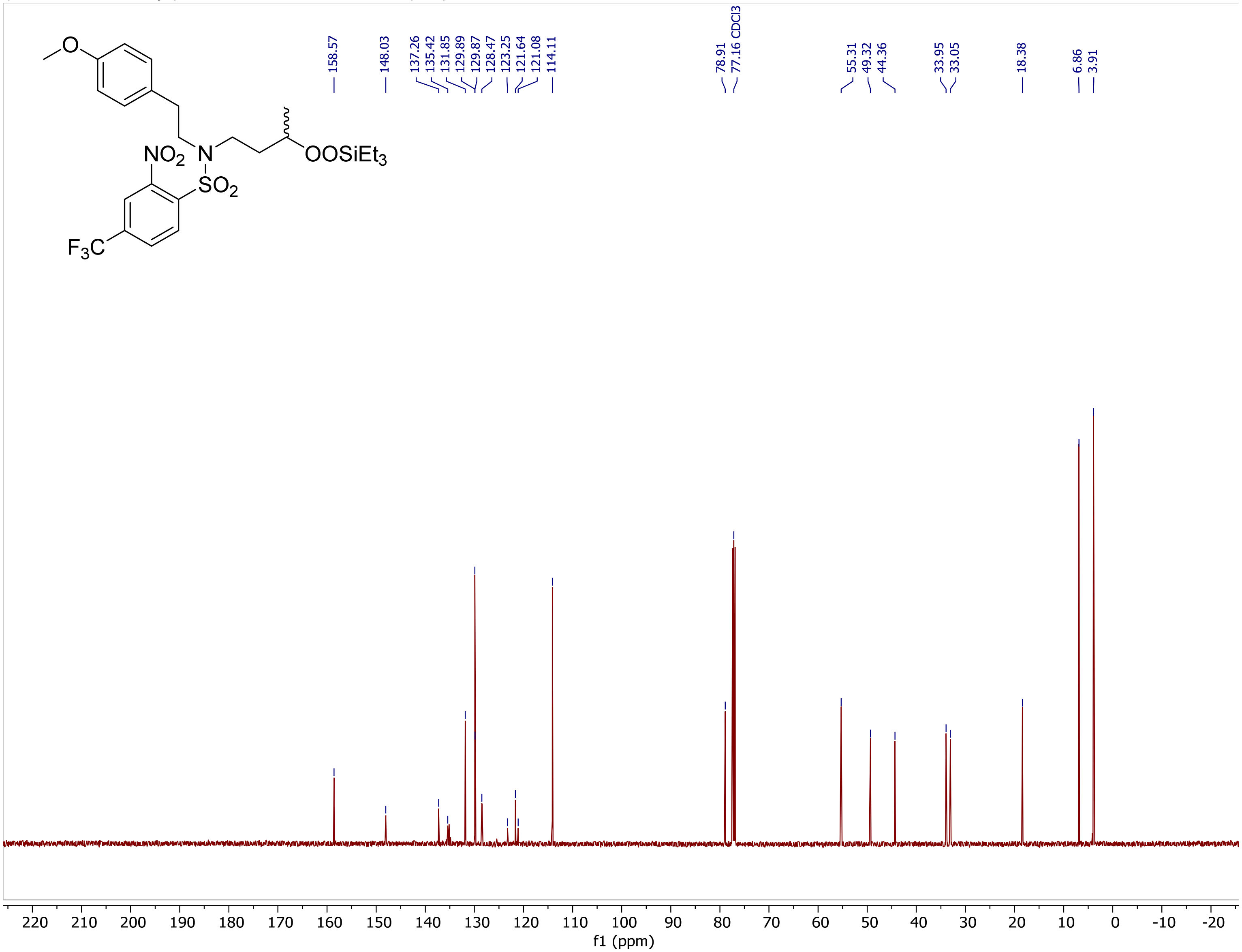

^{1}H NMR (500 MHz, C_6D_6) spectrum of *N*-(3-((*tert*-Butyldiphenylsilyl)peroxy)butyl)-*N*-(4-methoxyphenethyl)-2-nitro-4-(trifluoromethyl)benzenesulfonamide (**36**)

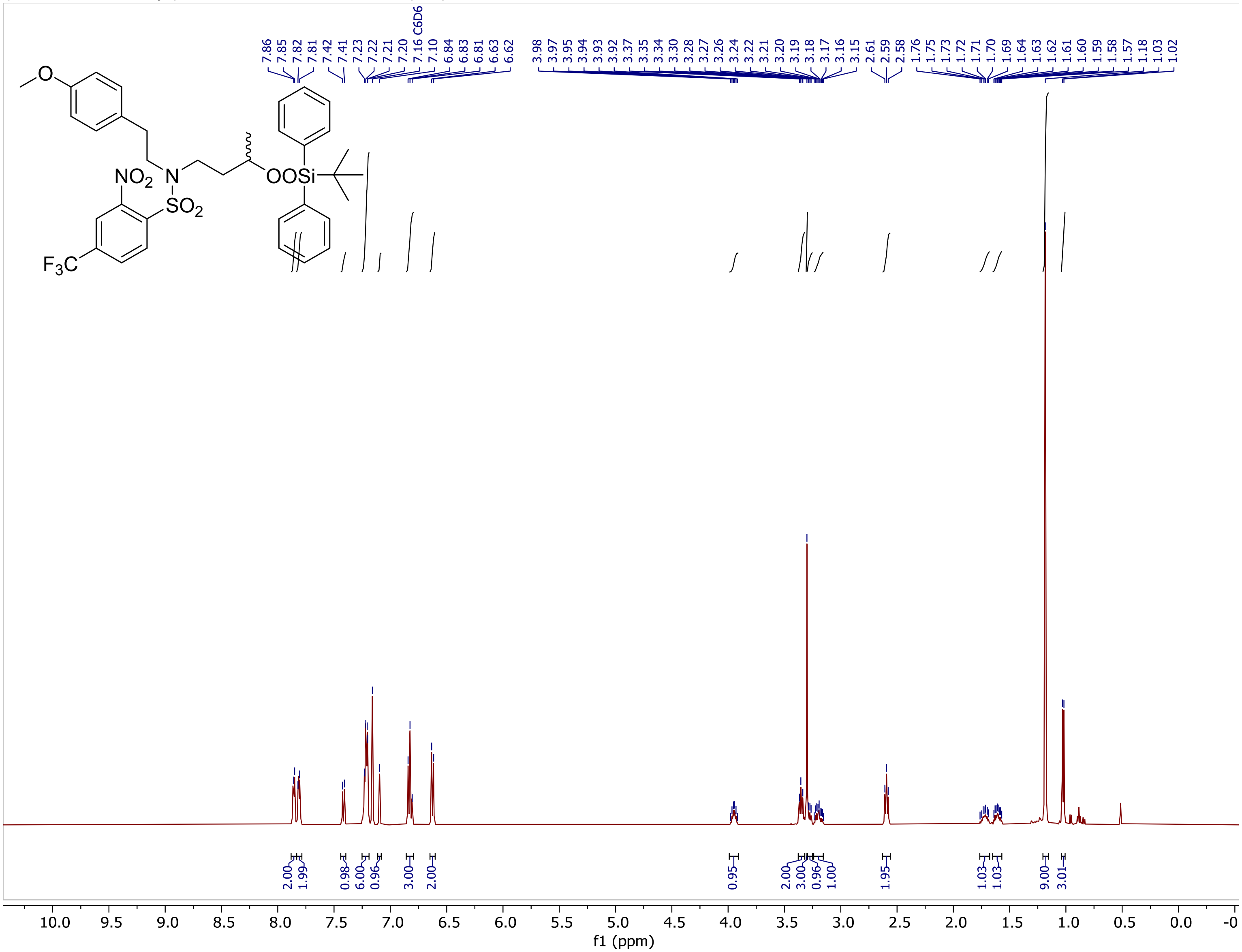

^{13}C {^{1}H} NMR (126 MHz, C_6D_6) spectrum of *N*-(3-((*tert*-Butyldiphenylsilyl)peroxy)butyl)-*N*-(4-methoxyphenethyl)-2-nitro-4-(trifluoromethyl)benzenesulfonamide (**36**)

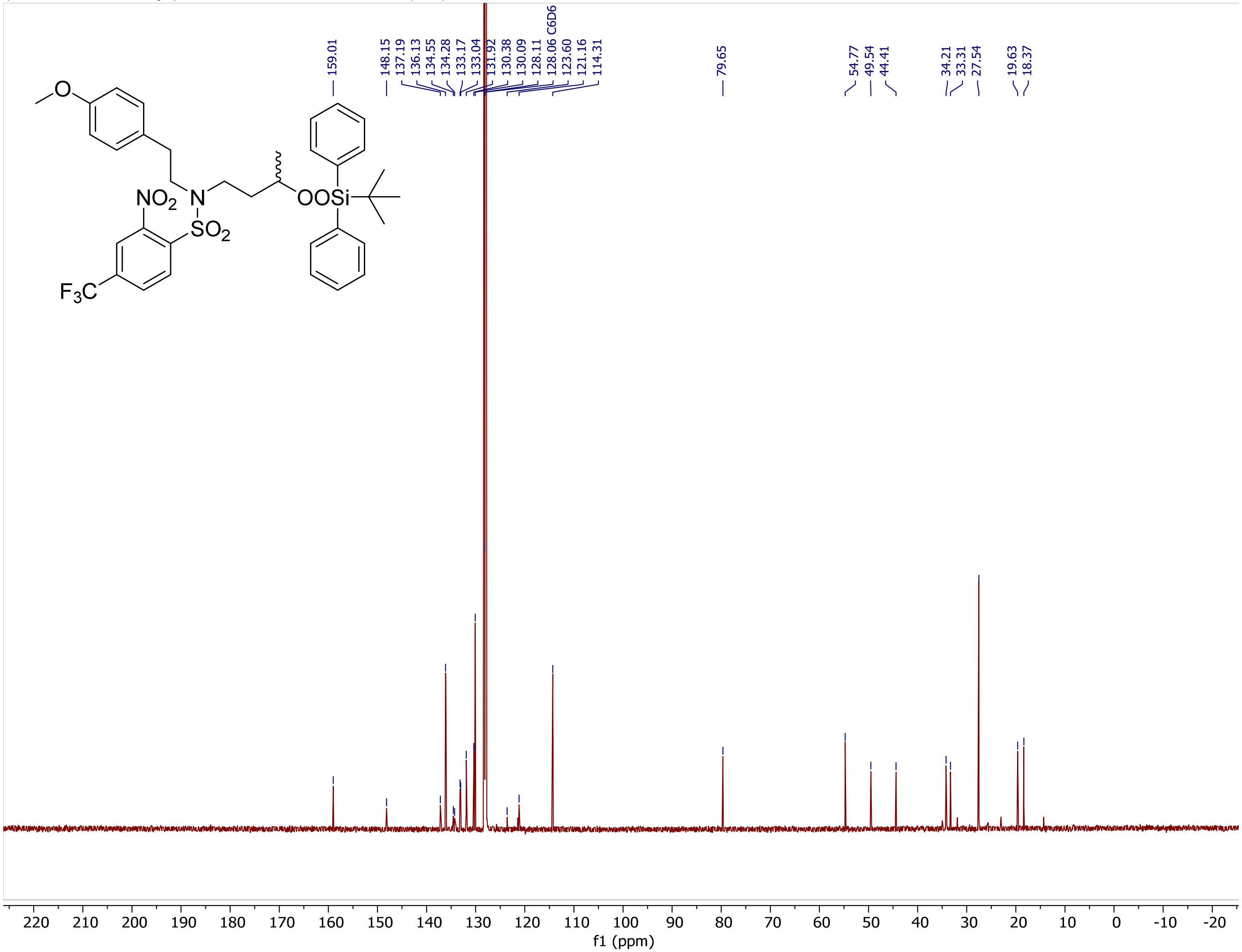

^{1}H NMR (500 MHz, C_6D_6) spectrum of 2-(Methoxyphenethyl)-5-methylisoxazolidine (**37**)

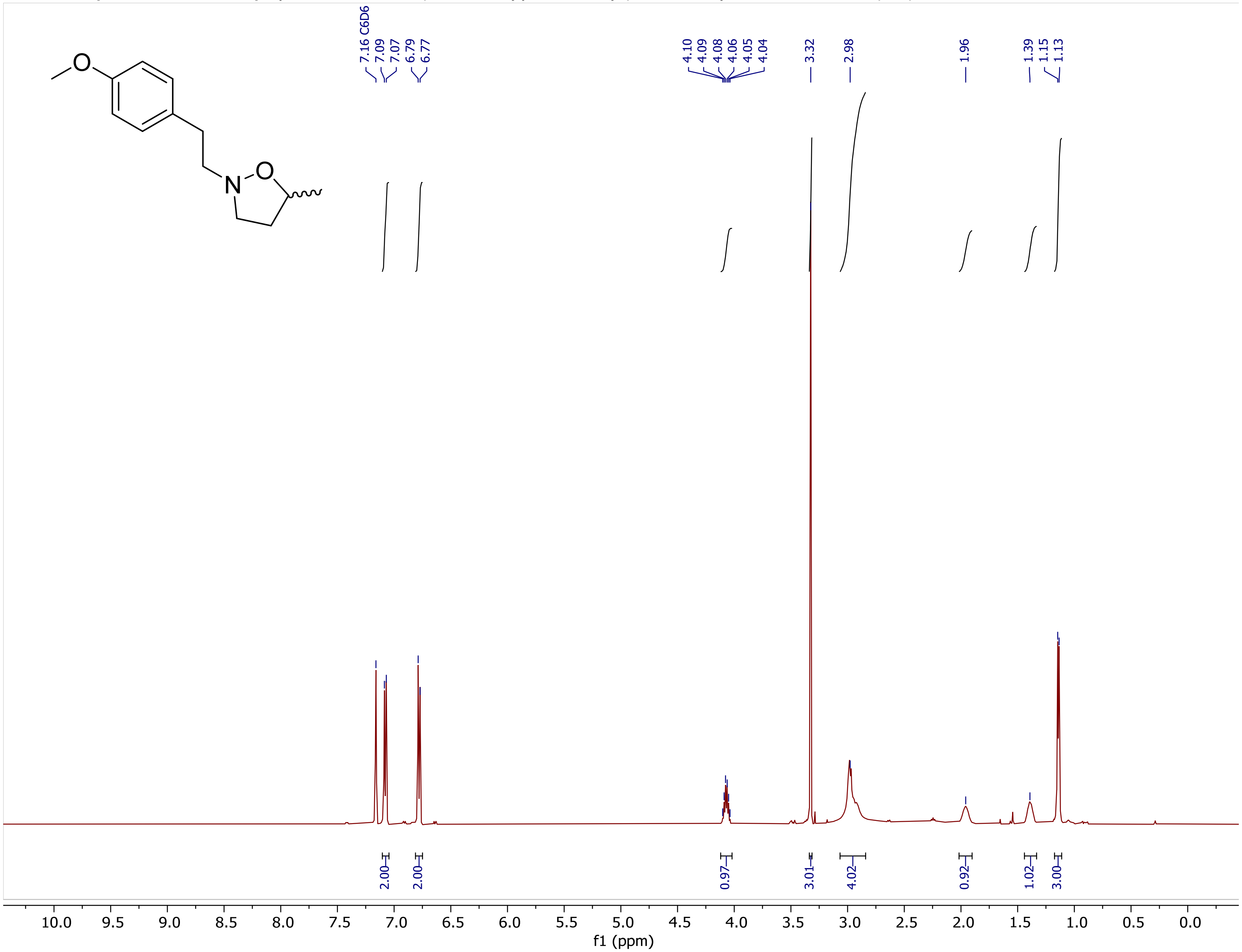

^{1}H NMR (500 MHz, C_6D_6) spectrum of 2-(Methoxyphenethyl)-5-methylisoxazolidine at 65 °C (**37**)

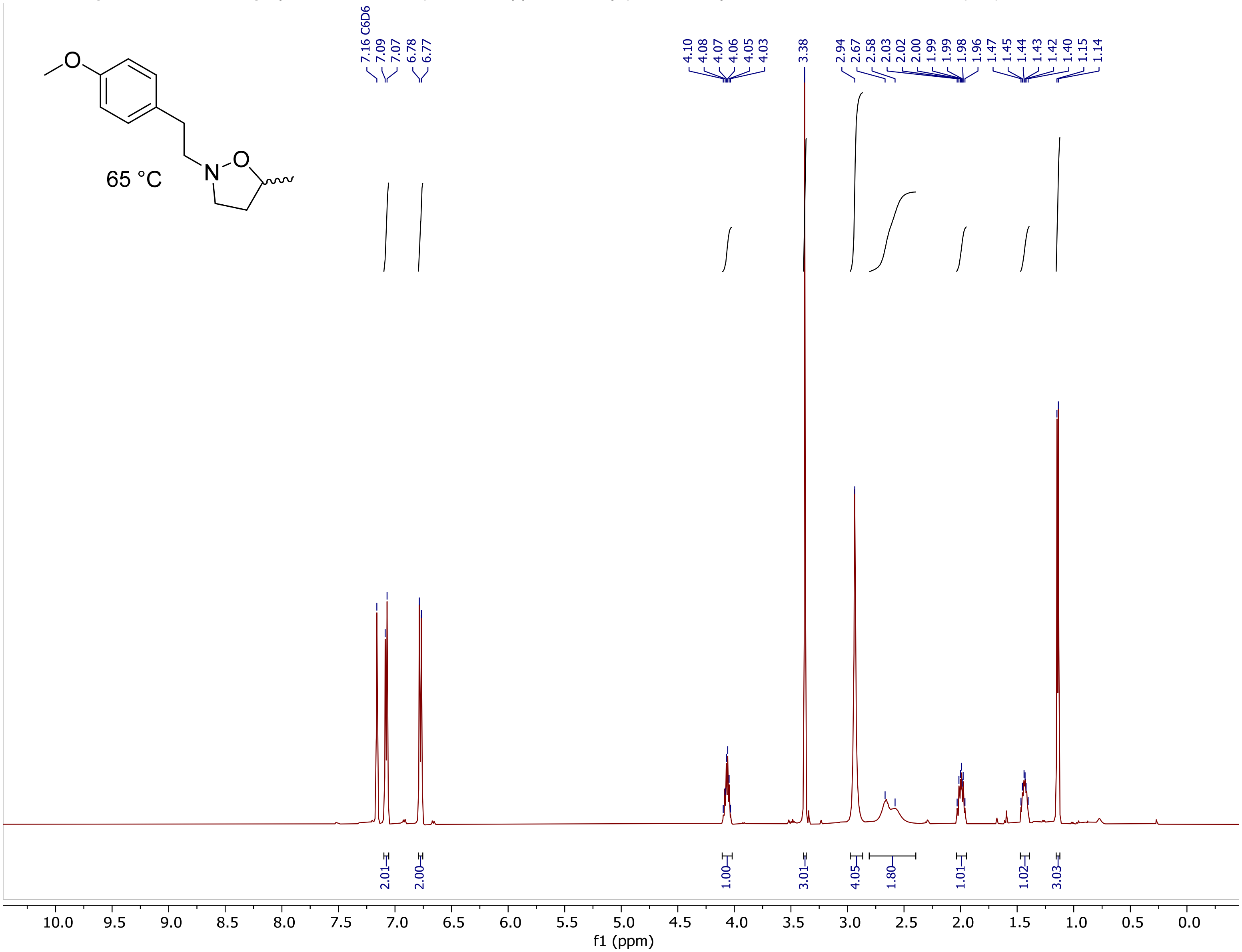

^{13}C {^{1}H} NMR (126 MHz, C_6D_6) spectrum of 2-(Methoxyphenethyl)-5-methylisoxazolidine (**37**)

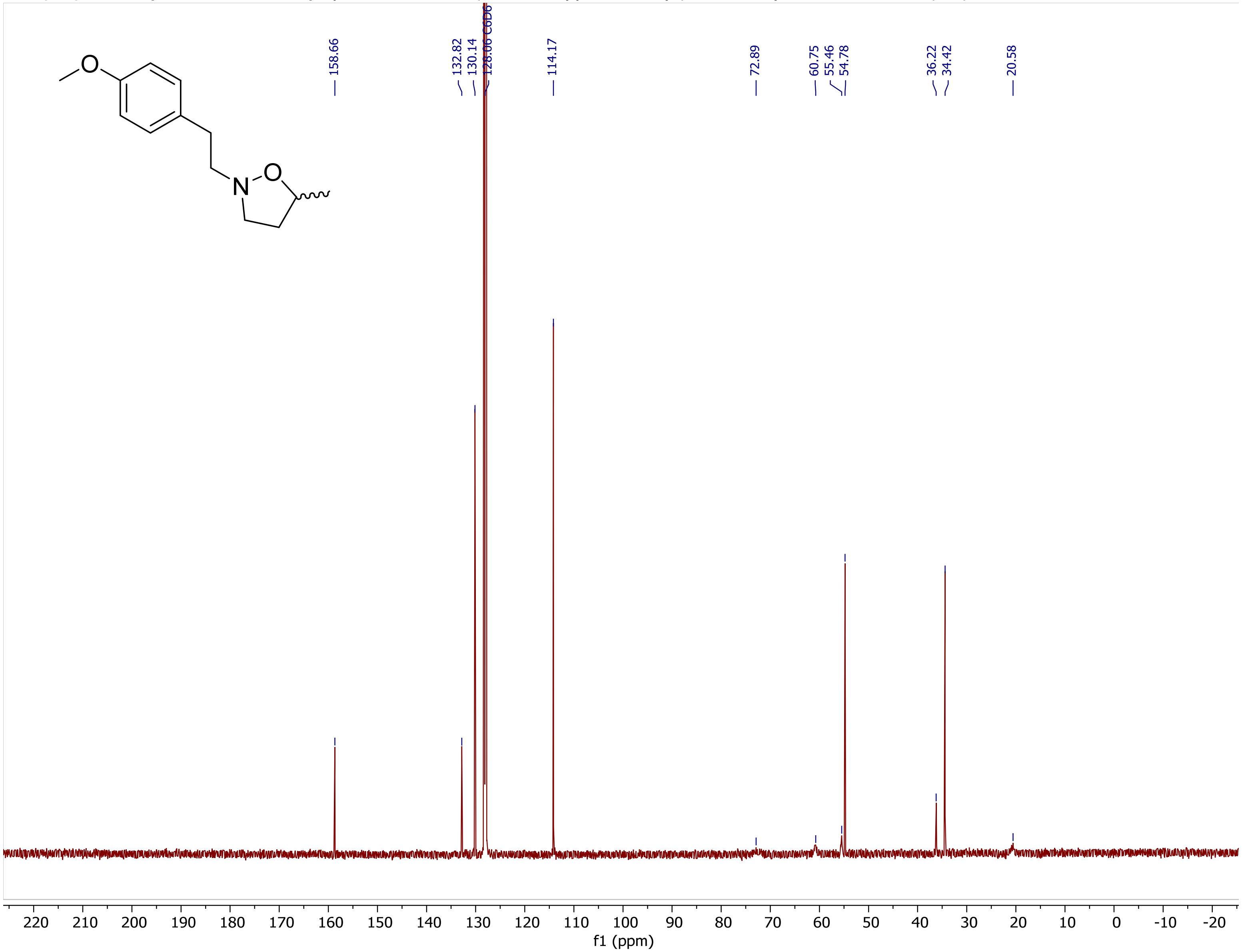

^{13}C {^{1}H} NMR (126 MHz, C_6D_6) spectrum of 2-(Methoxyphenethyl)-5-methylisoxazolidine at 65 °C (**37**)

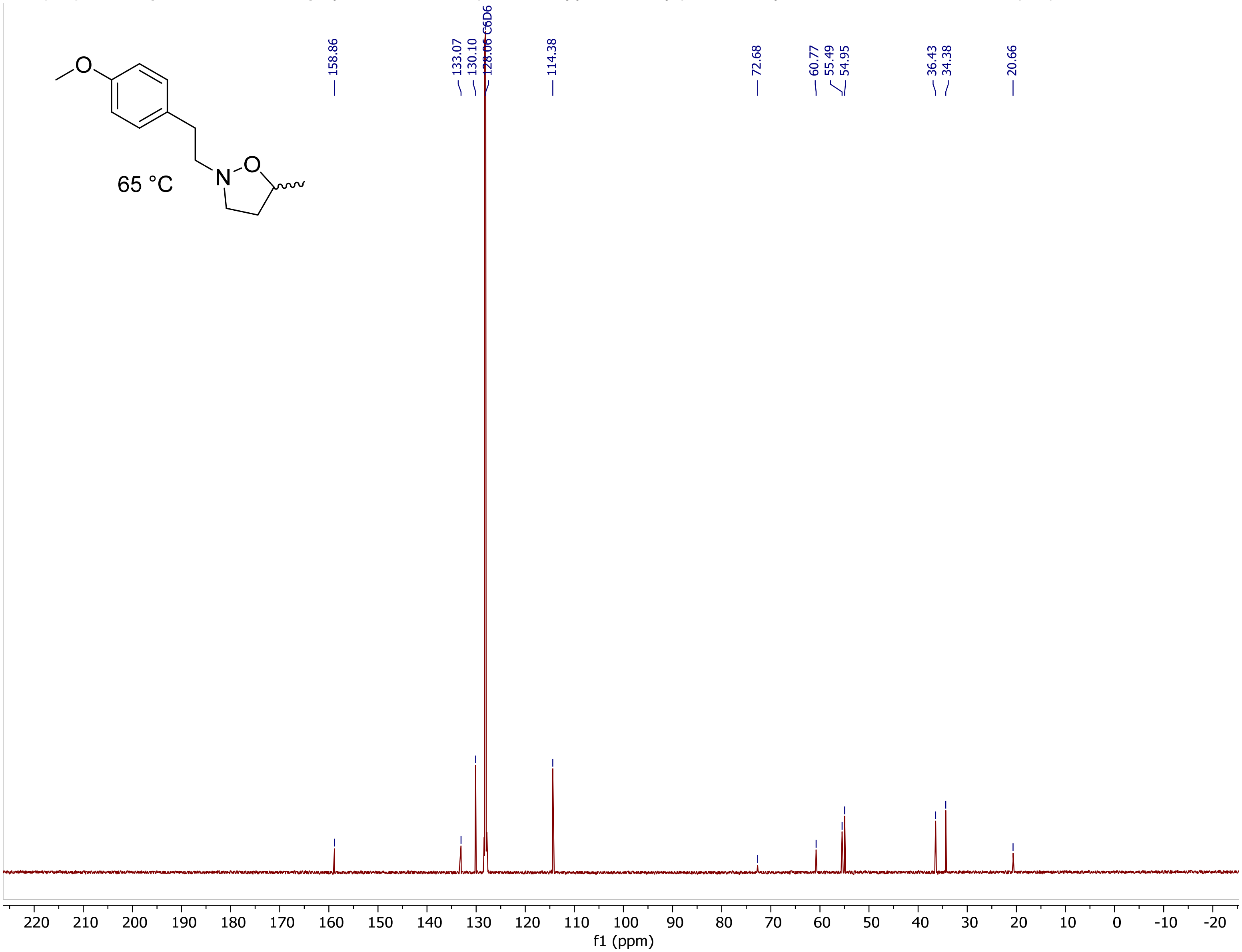

HSQC NMR (500 MHz, C_6D_6) spectrum of 2-(Methoxyphenethyl)-5-methylisoxazolidine at 65 °C (**37**)

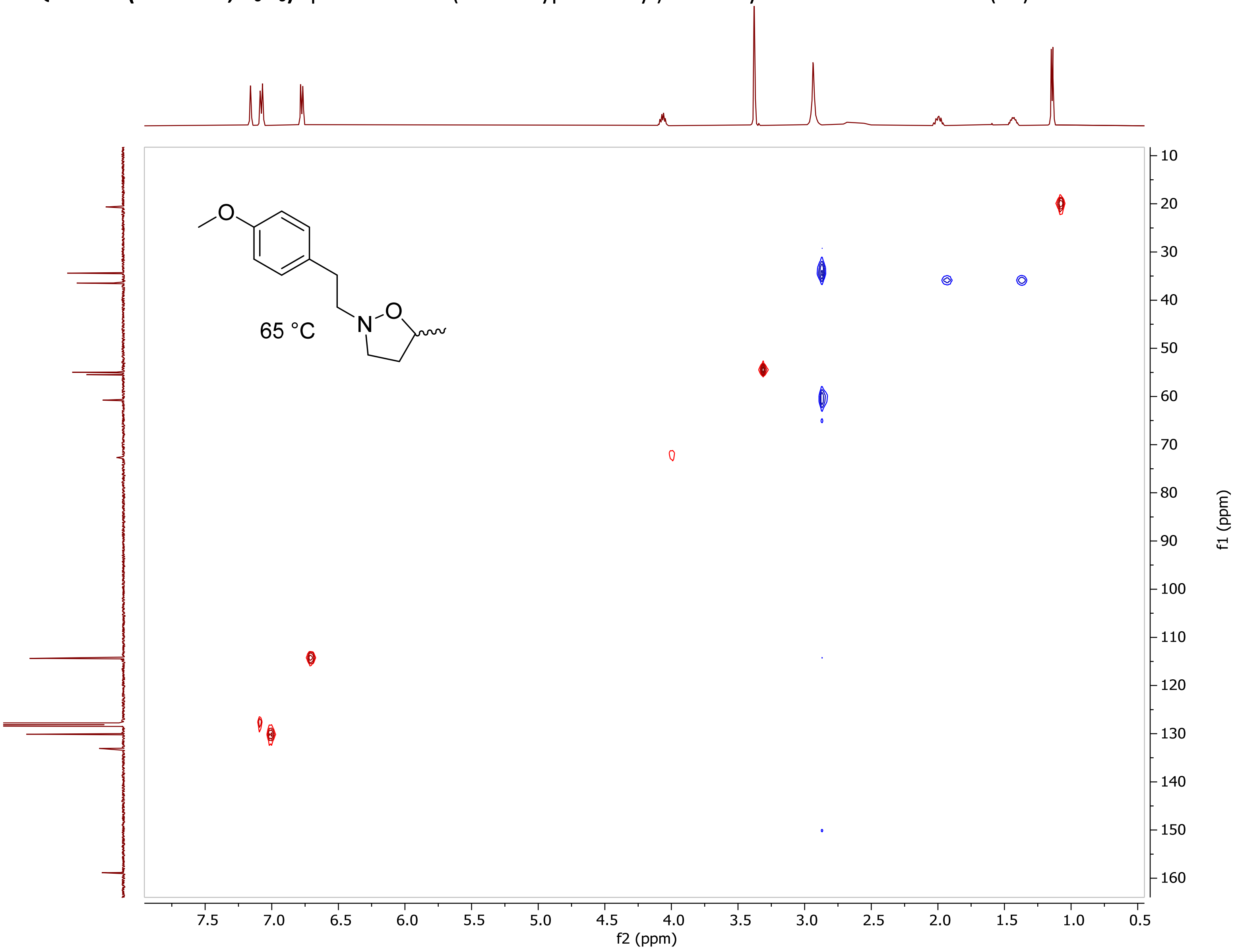

^{1}H NMR (500 MHz, $CDCl_3$) spectrum of 2-Nitro-*N*-phenyl-4-(trifluoromethyl)benzenesulfonamide (**S6**)

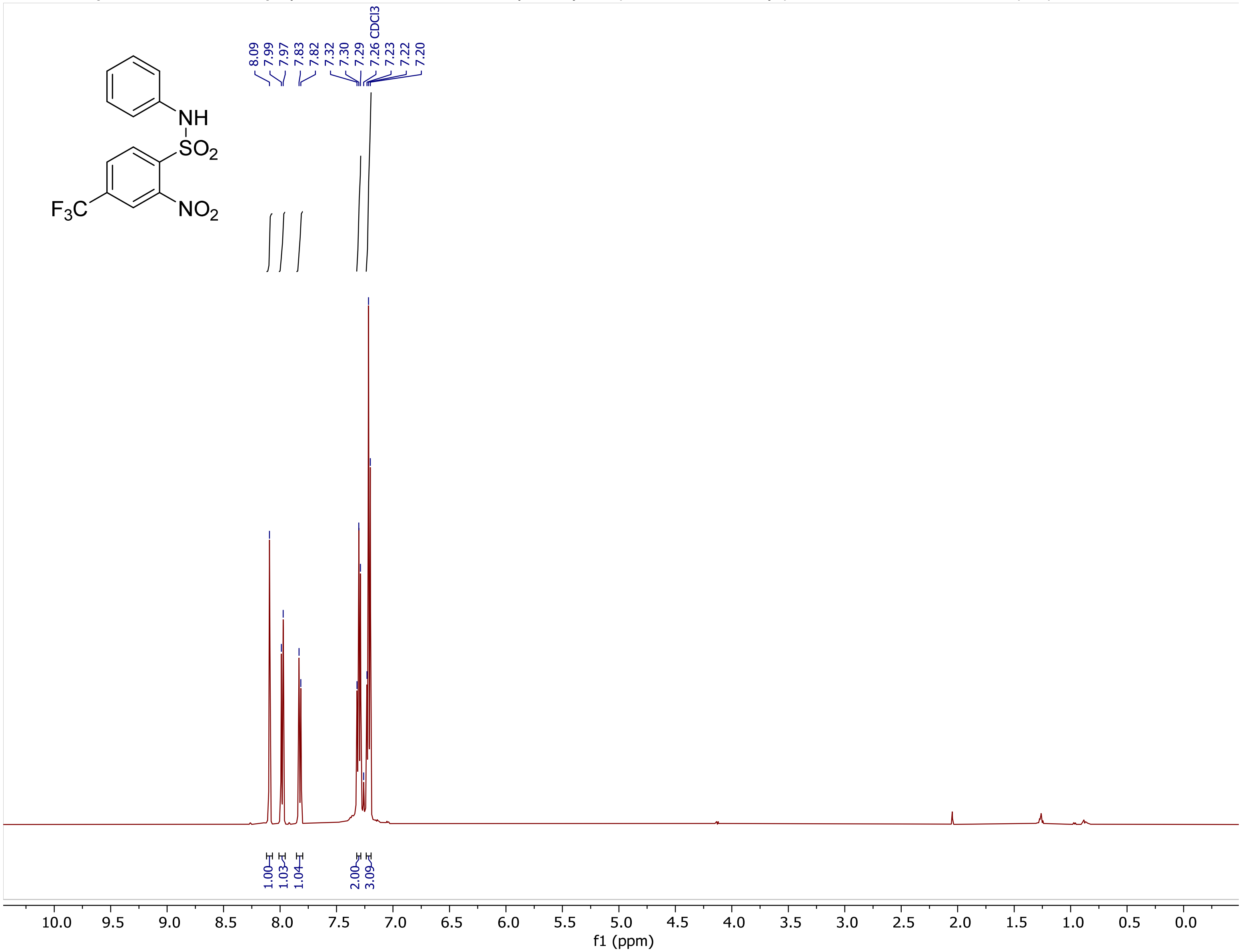

^{13}C {^{1}H} NMR (126 MHz, $CDCl_3$) spectrum of 2-Nitro-*N*-phenyl-4-(trifluoromethyl)benzenesulfonamide (**S6**)

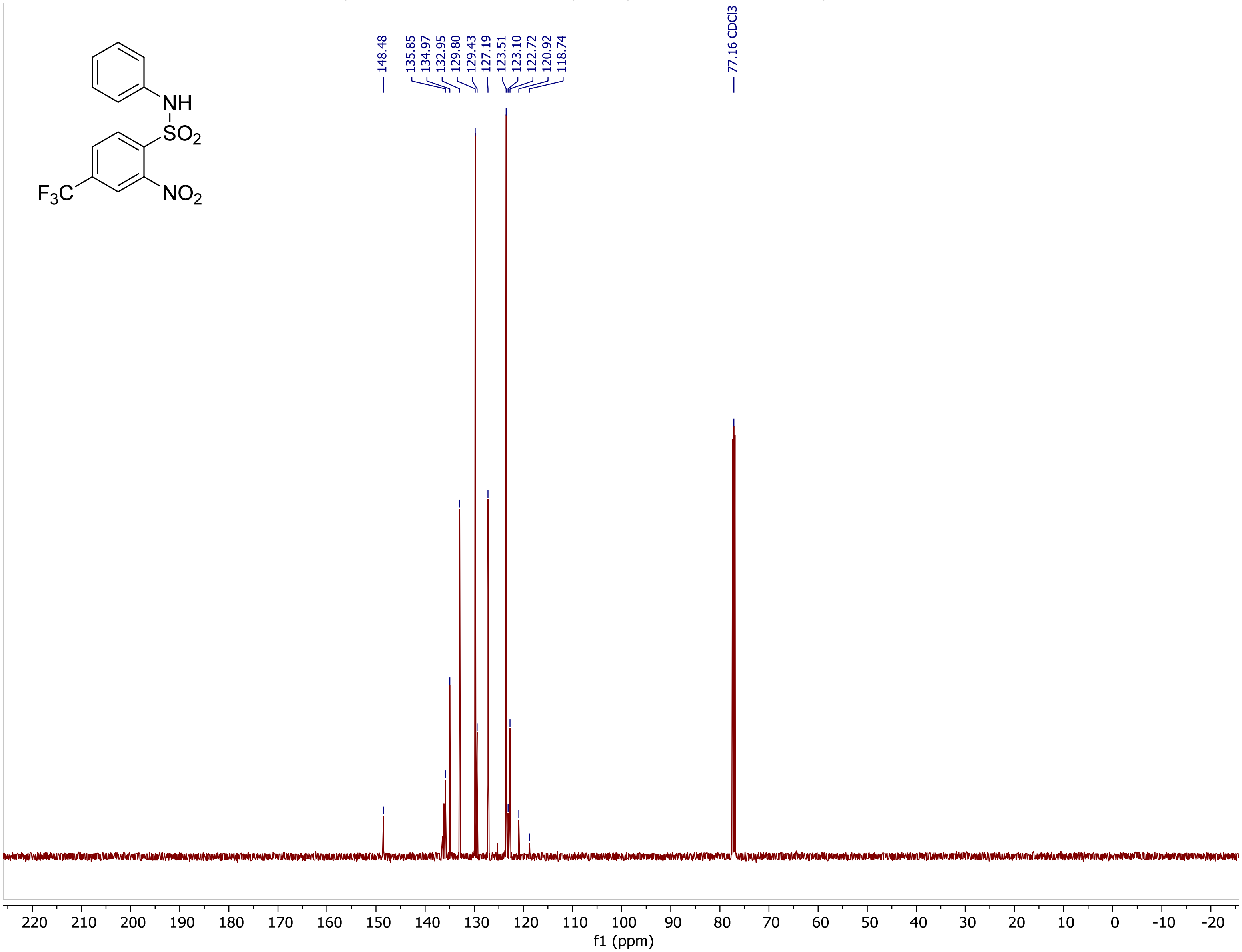

^{1}H NMR (500 MHz, $CDCl_3$) spectrum of *N*-(But-3-en-1-yl)-2-nitro-*N*-phenyl-4-(trifluoromethyl)benzenesulfonamide (**6a**)

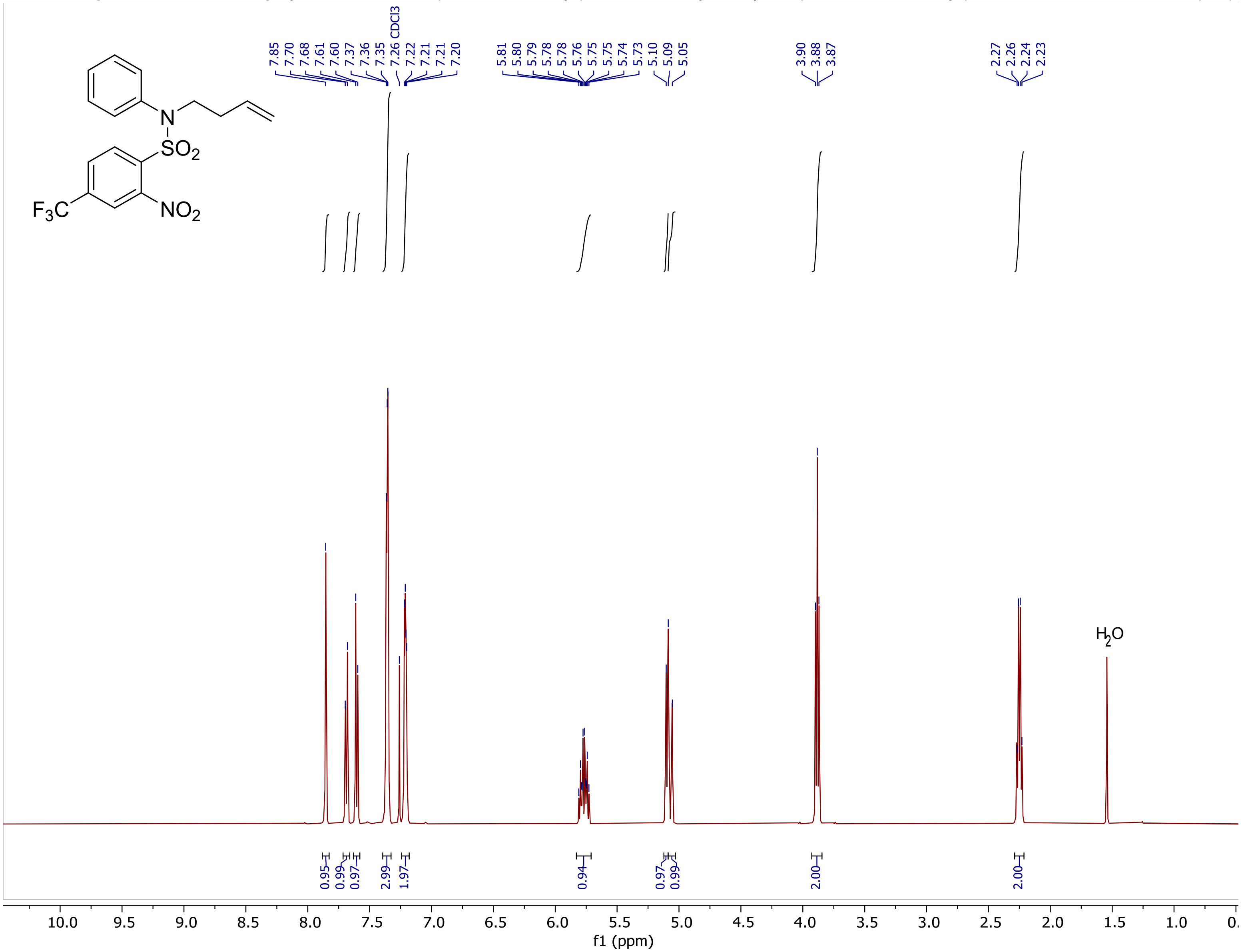

$^{13}C\ \{^{1}H\}$ NMR (126 MHz, $CDCl_3$) spectrum of *N*-(But-3-en-1-yl)-2-nitro-*N*-phenyl-4-(trifluoromethyl)benzenesulfonamide (**6a**)

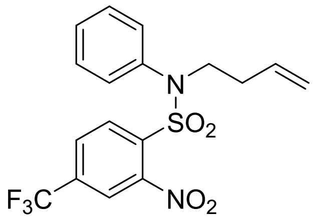

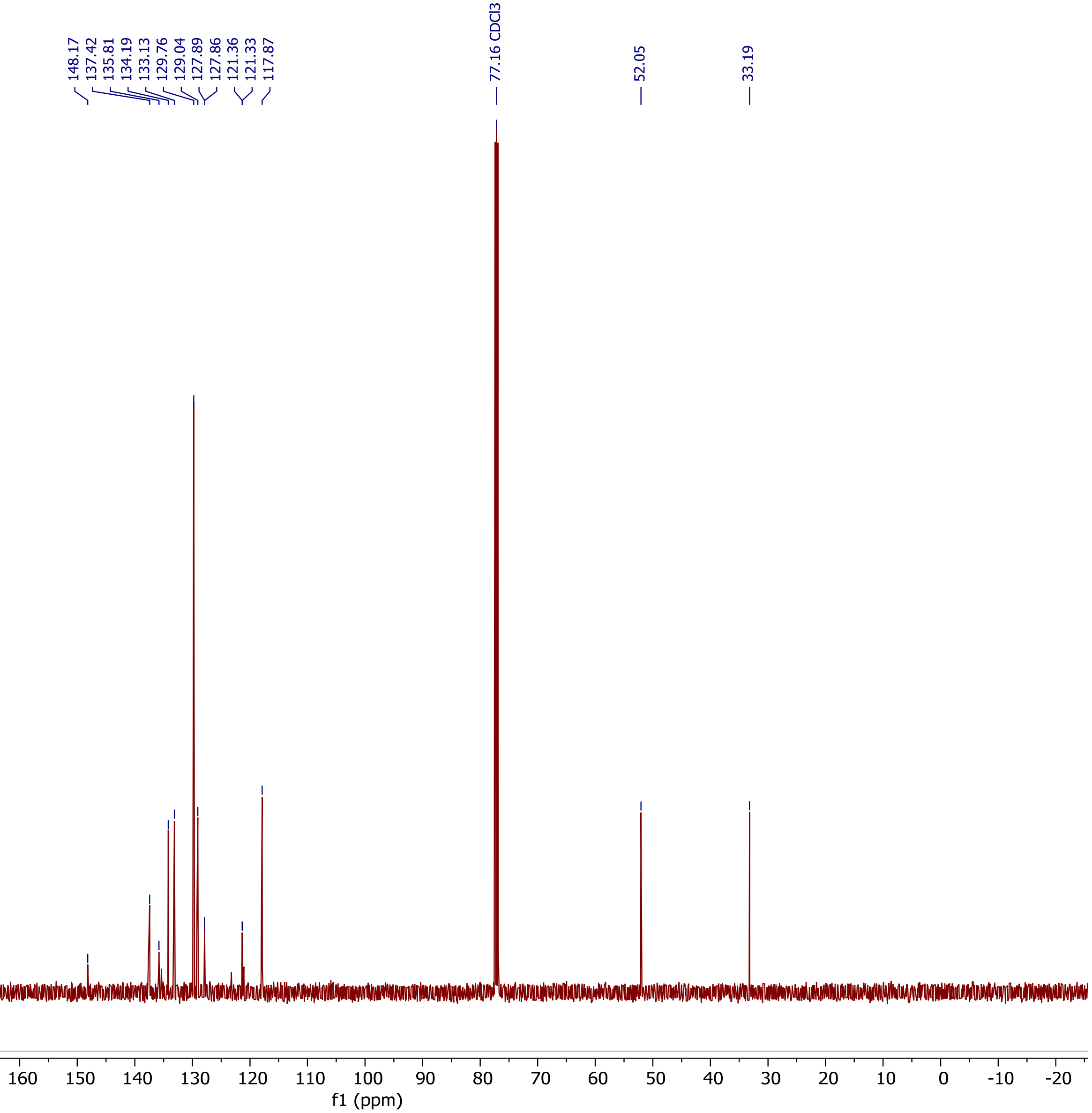

^{1}H NMR (500 MHz, $CDCl_3$) spectrum of 2-Nitro-*N*-phenyl-*N*-(3-((triethylsilyl)peroxy)butyl)-4-(trifluoromethyl)benzenesulfonamide (**6b**)

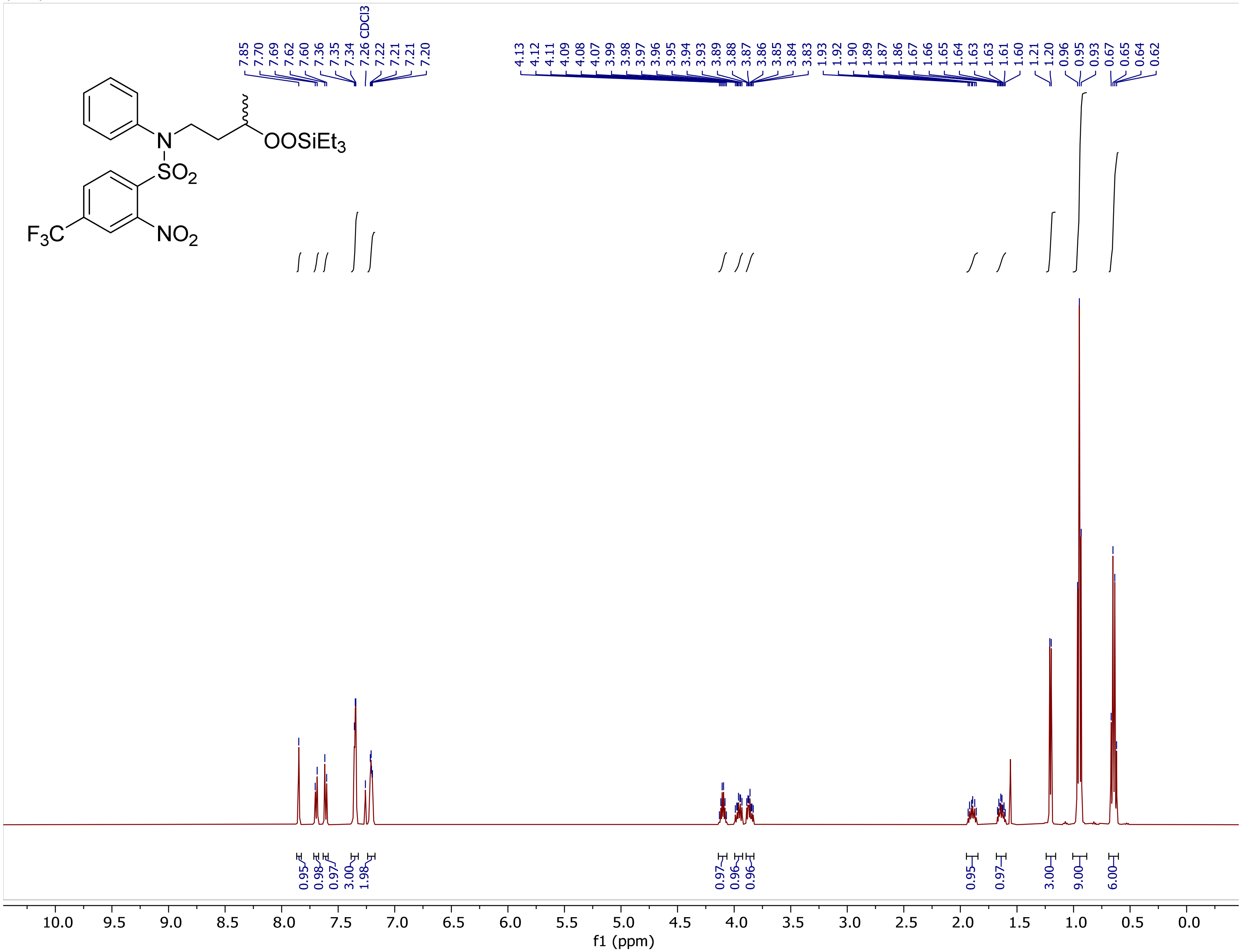

^{13}C {^{1}H} NMR (126 MHz, $CDCl_3$) spectrum of 2-Nitro-*N*-phenyl-*N*-(3-((triethylsilyl)peroxy)butyl)-4-(trifluoromethyl)benzenesulfonamide (**6b**)

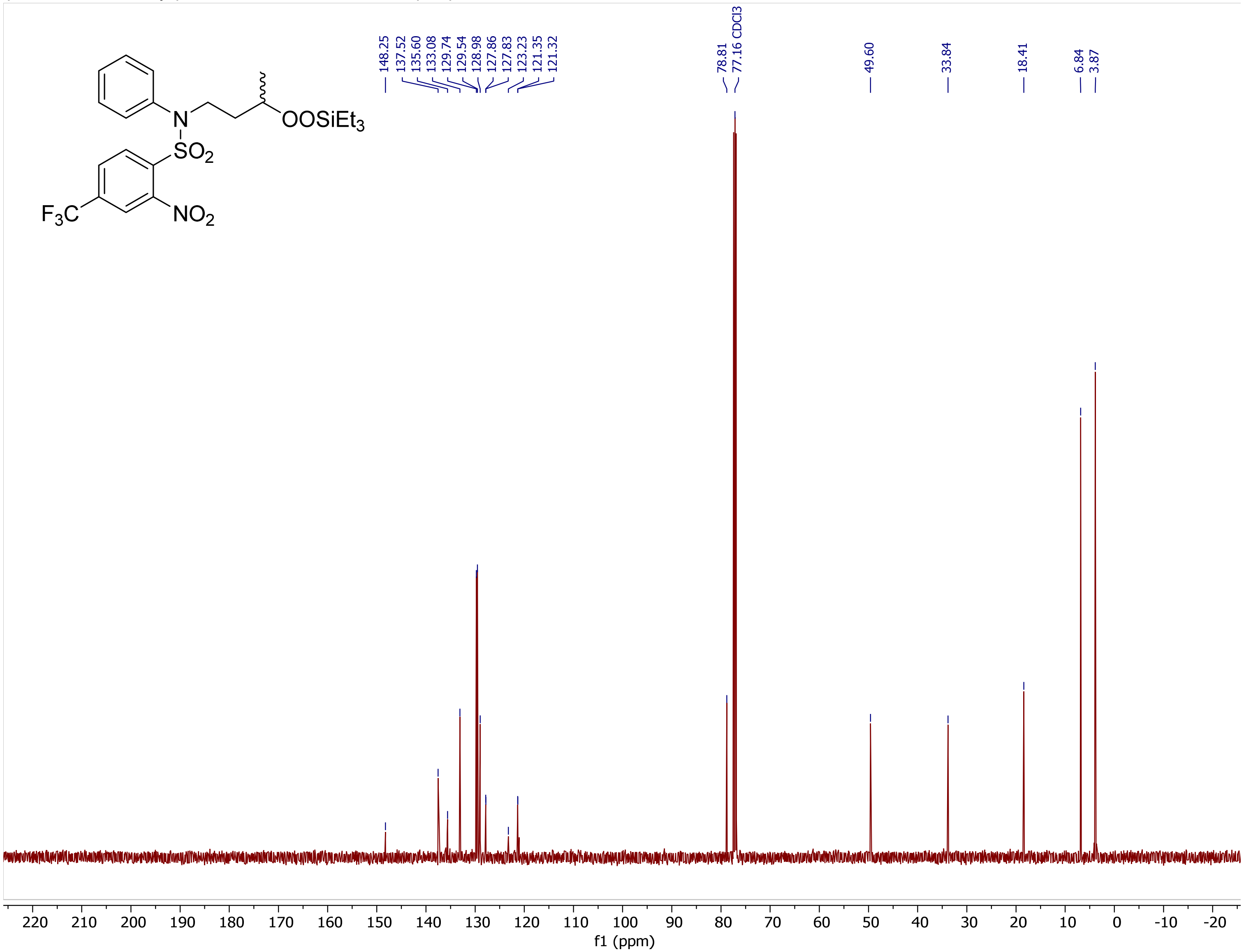

^{1}H NMR (500 MHz, C_6D_6) spectrum of *N*-(3-((*tert*-Butyldiphenylsilyl)peroxy)butyl)-2-nitro-*N*-phenyl-4-(trifluoromethyl)benzenesulfonamide (**38**)

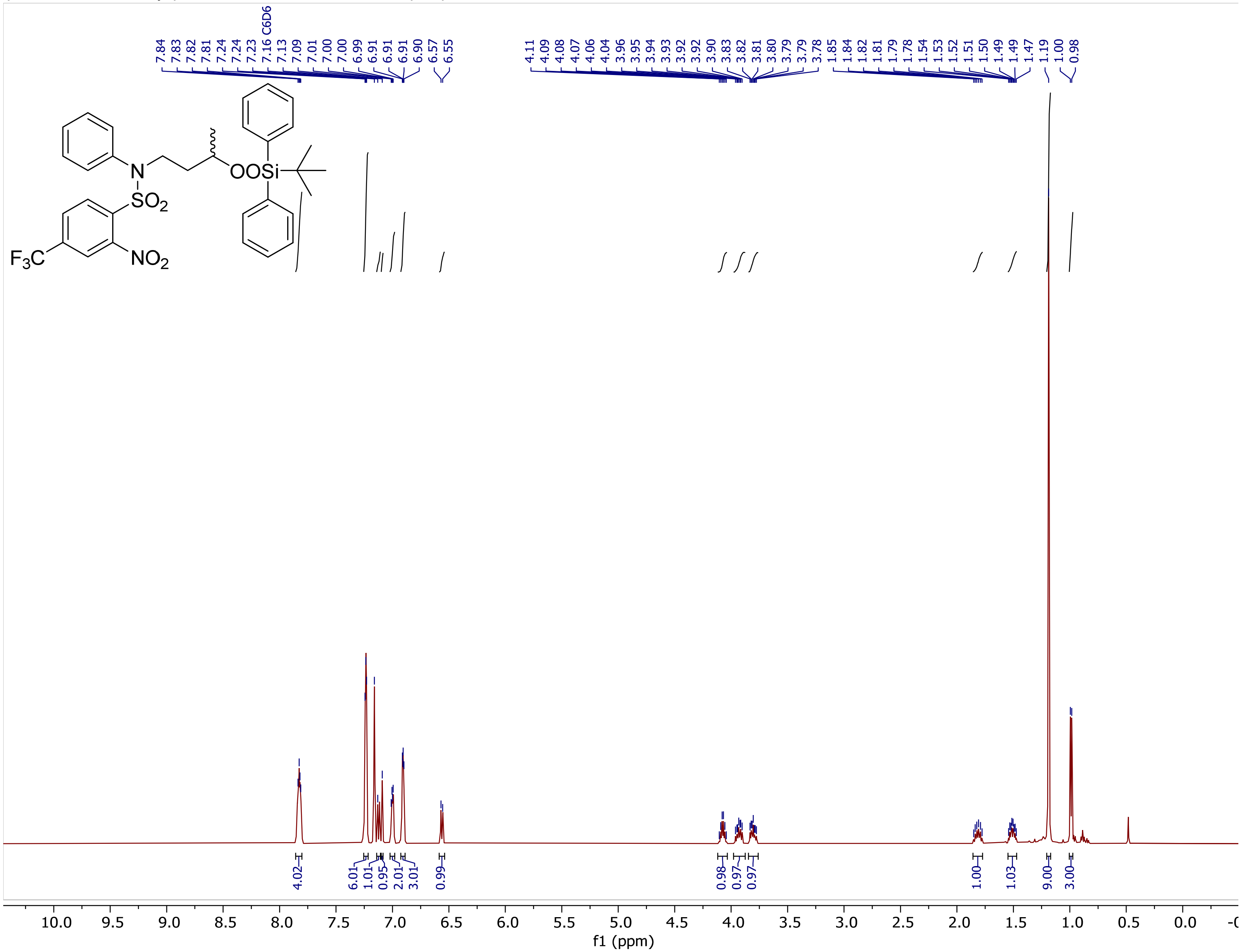

^{13}C {^{1}H} NMR (126 MHz, C_6D_6) spectrum of *N*-(3-((*tert*-Butyldiphenylsilyl)peroxy)butyl)-2-nitro-*N*-phenyl-4-(trifluoromethyl)benzenesulfonamide (**38**)

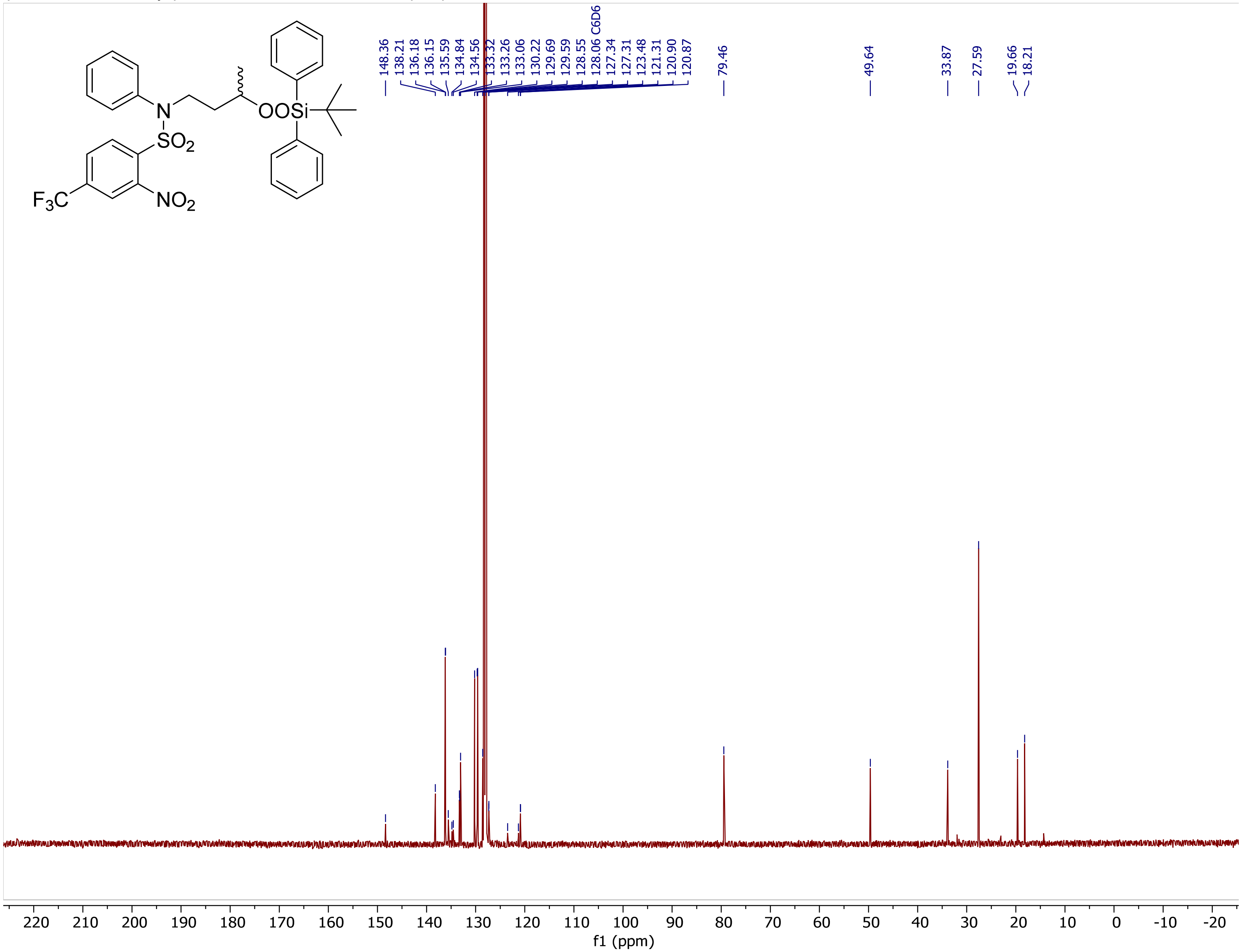

^{1}H NMR (500 MHz, $CDCl_3$) spectrum of 5-Methyl-2-phenylisoxazolidine (**39**)

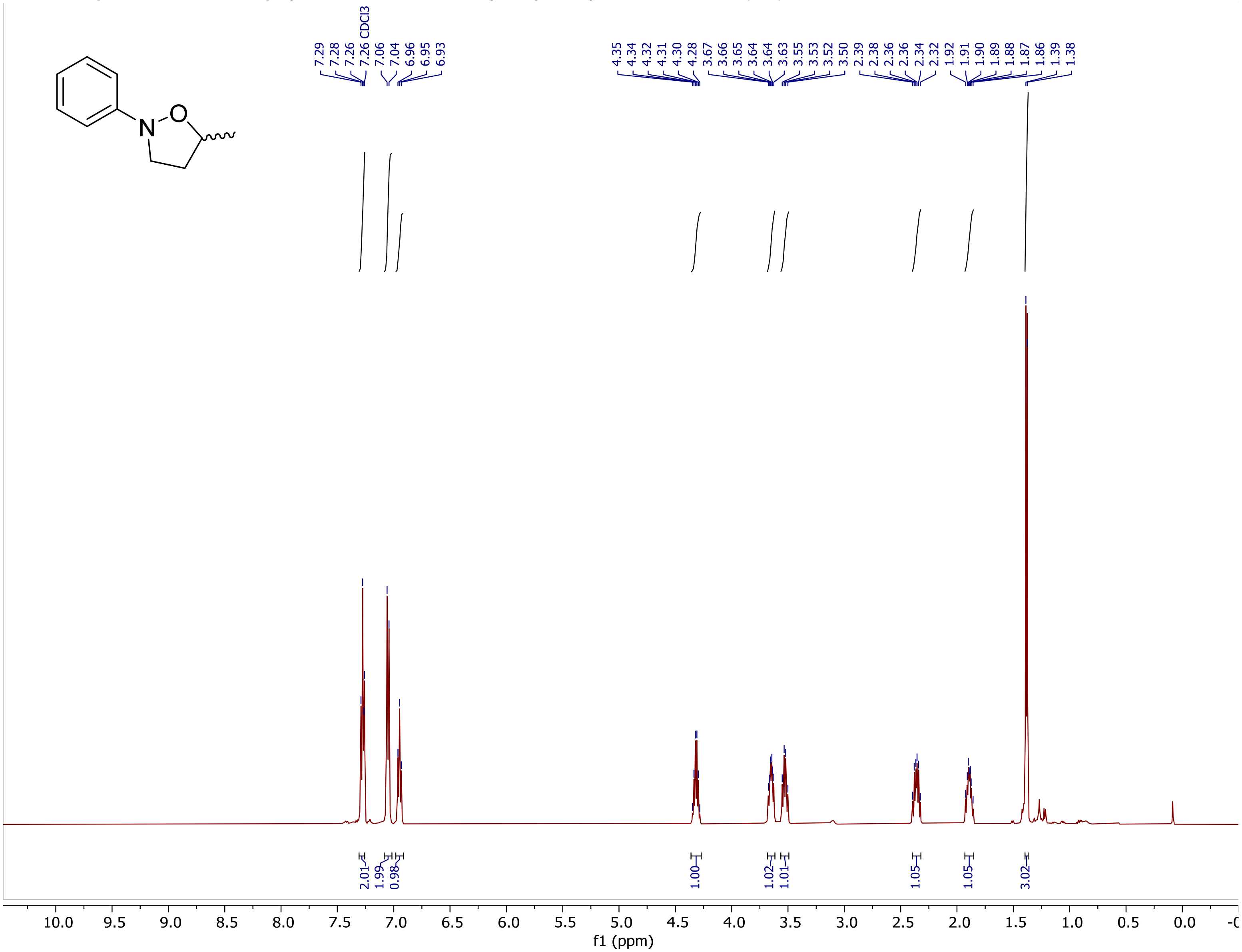

^{13}C {^{1}H} NMR (126 MHz, $CDCl_3$) spectrum of 5-Methyl-2-phenylisoxazolidine (**39**)

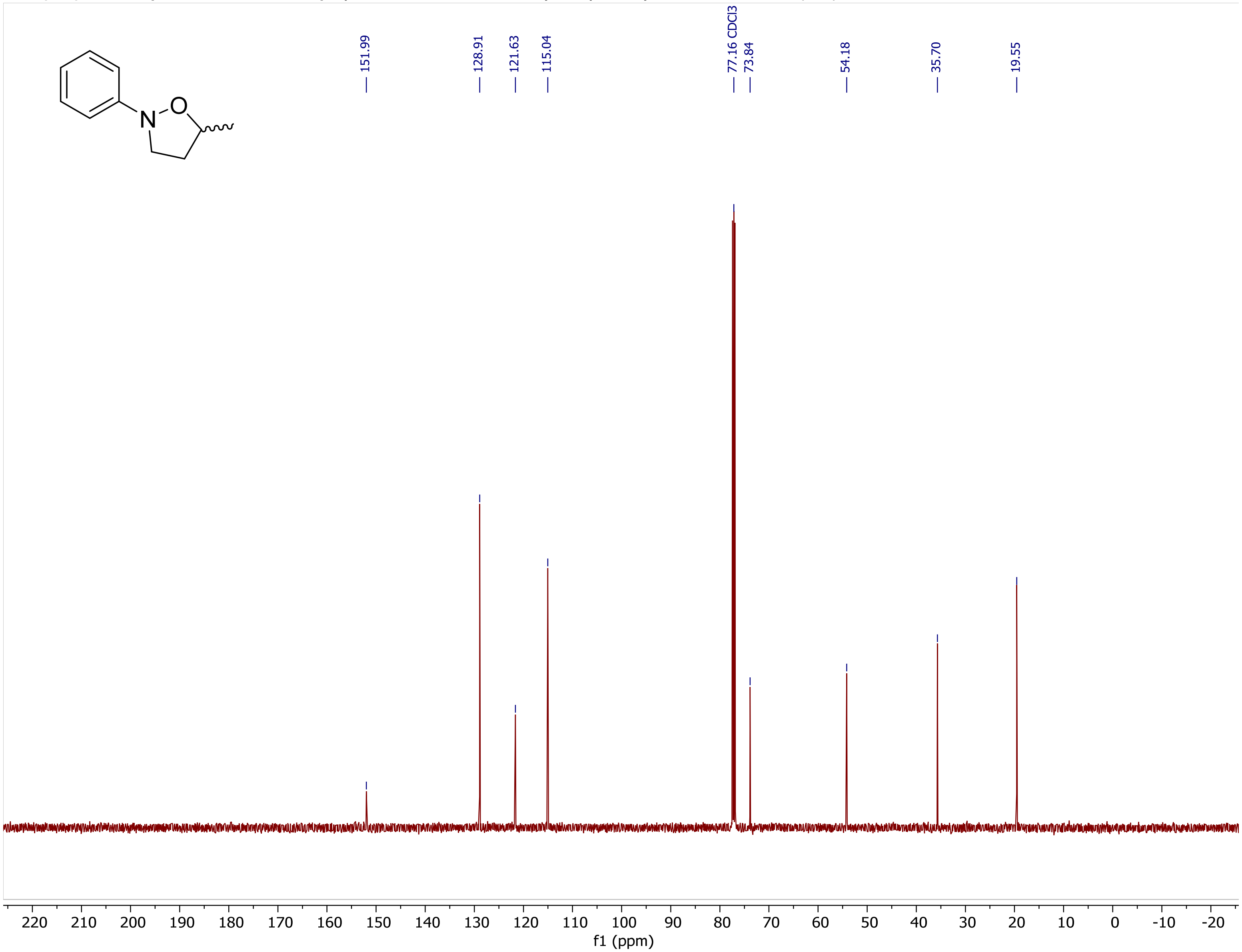

HSQC NMR (500 MHz, CDCl₃) spectrum of 5-Methyl-2-phenylisoxazolidine (**39**)

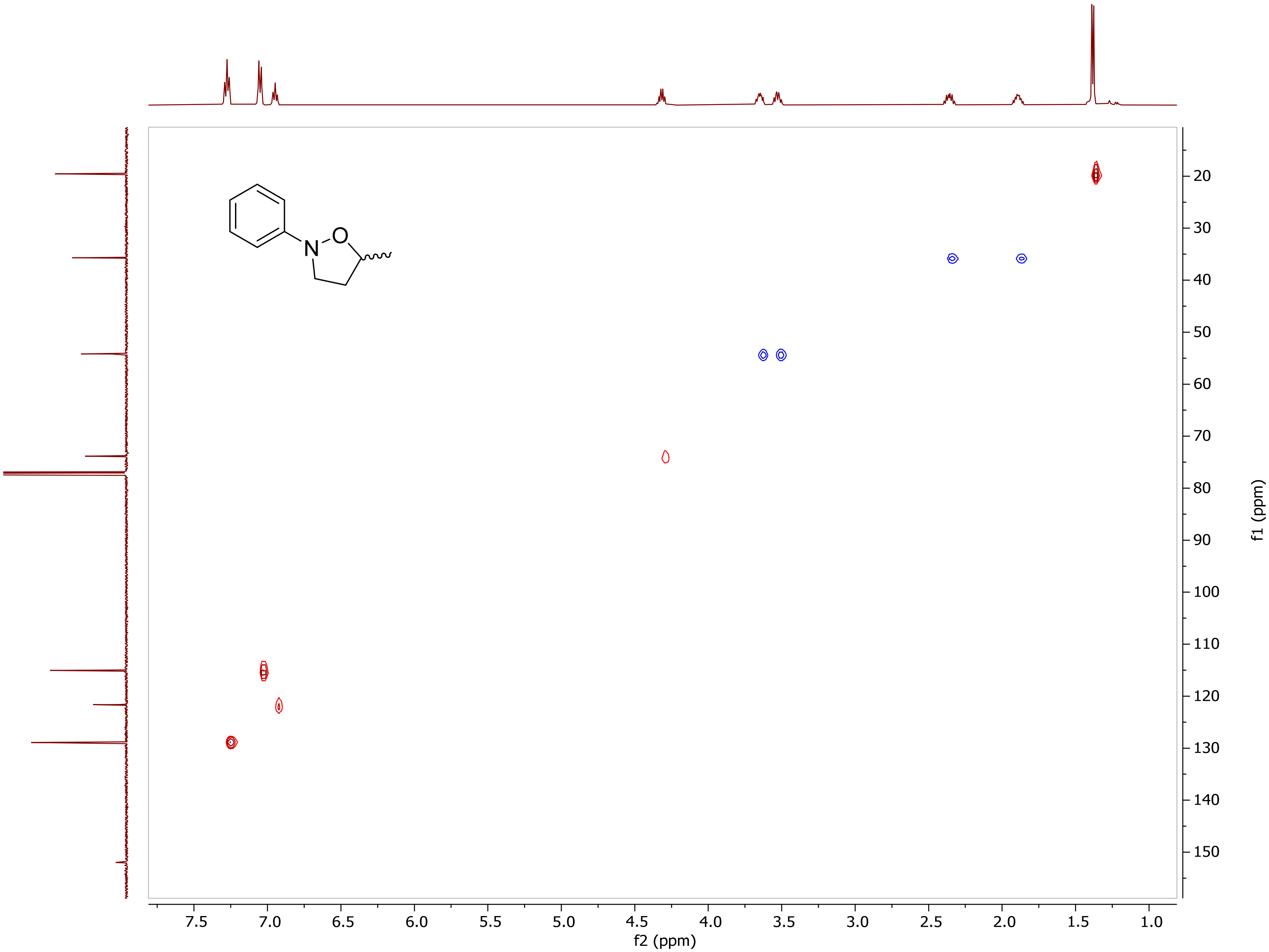

^{1}H NMR (500 MHz, CDCl$_3$) spectrum of *N*-(2-Methylallyl)-*N*-(naphthalen-1-ylmethyl)-2-nitro-4-(trifluoromethyl)benzenesulfonamide (**3a**)

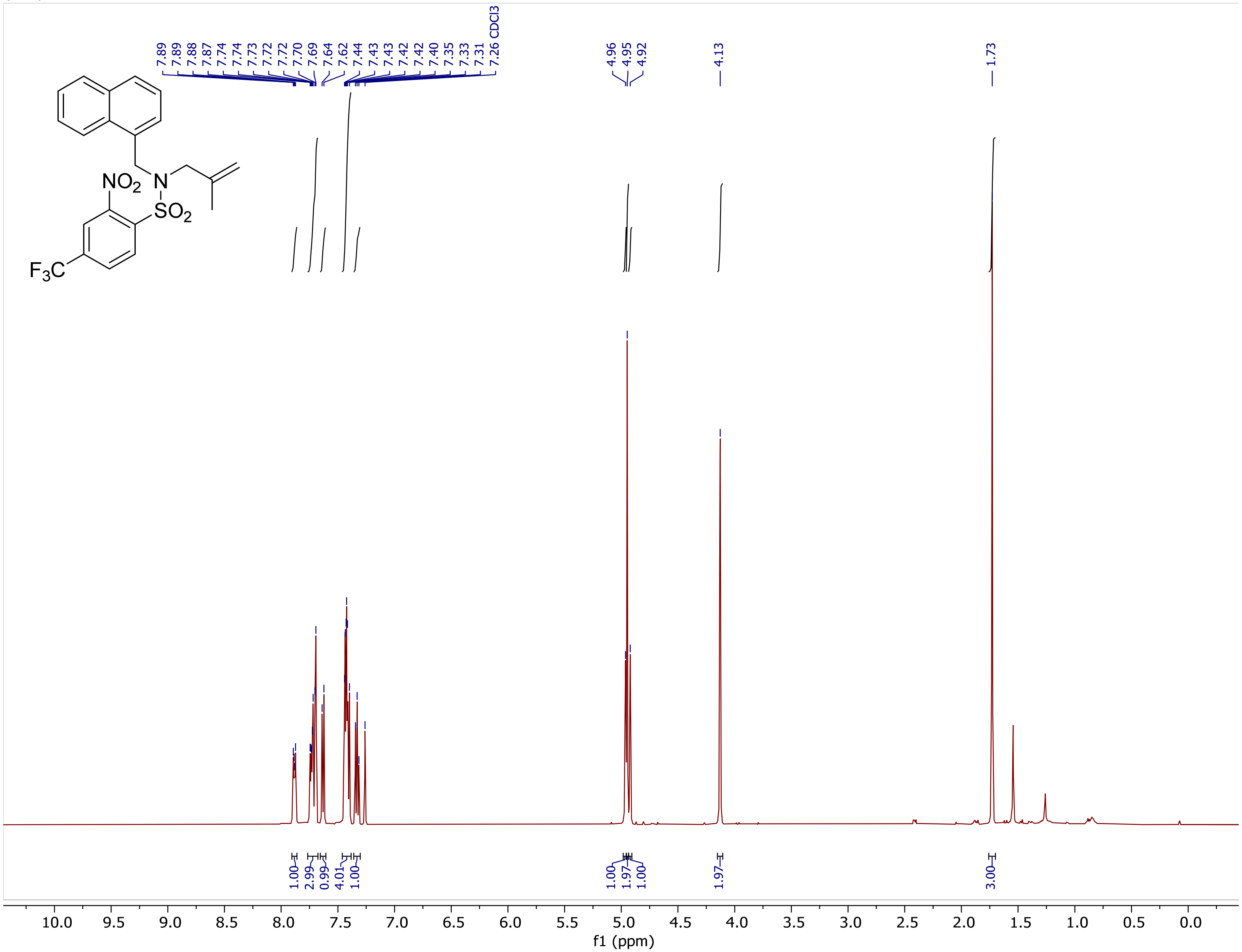

$^{13}C\ \{^{1}H\}$ NMR (126 MHz, $CDCl_3$) spectrum of *N*-(2-Methylallyl)-*N*-(naphthalen-1-ylmethyl)-2-nitro-4-(trifluoromethyl)benzenesulfonamide (**3a**)

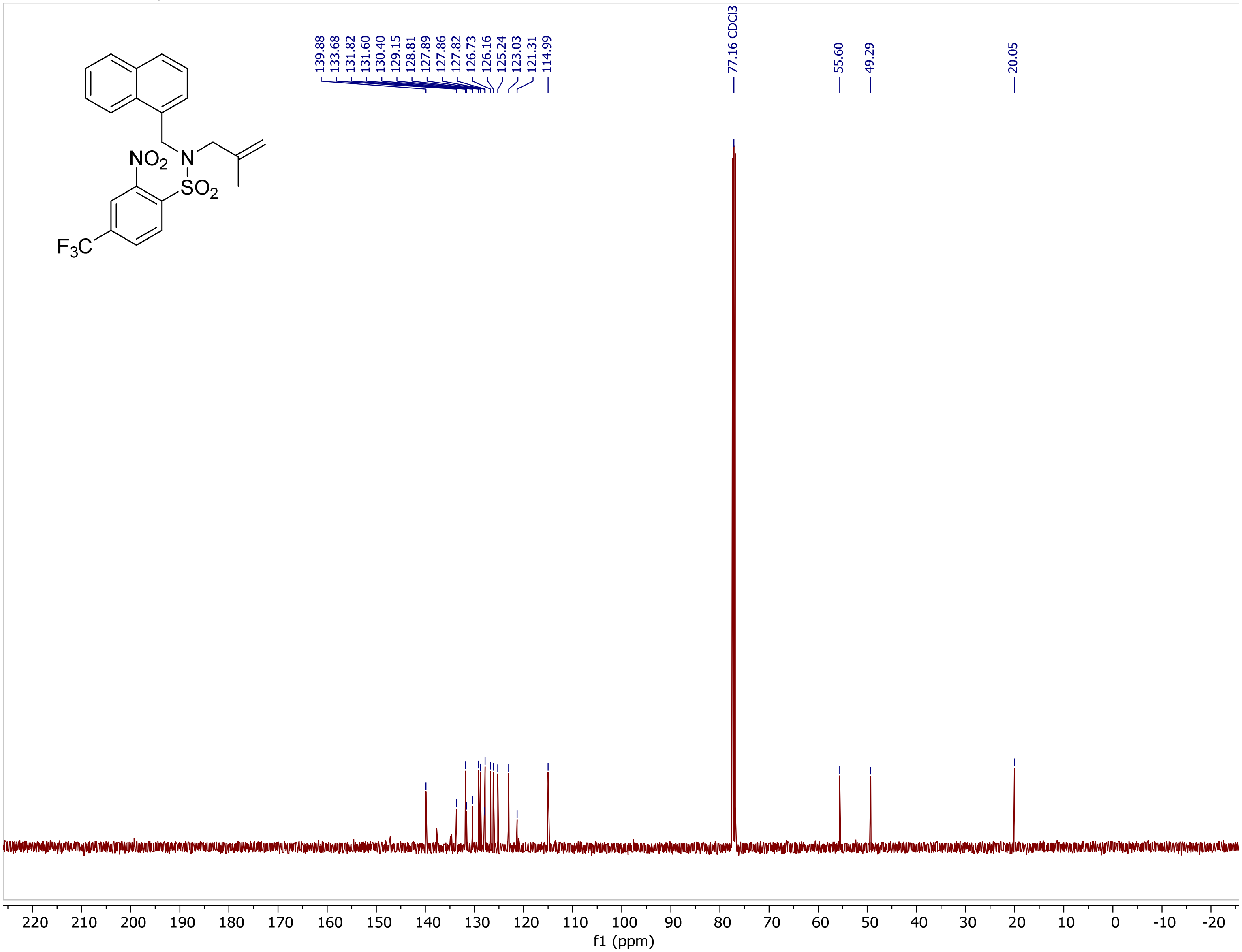

^{1}H NMR (500 MHz, $CDCl_3$) spectrum of *N*-(3-Hydroxy-2-methylpropyl)-*N*-(naphthalen-1-ylmethyl)-2-nitro-4-(trifluoromethyl)benzenesulfonamide (**3b**)

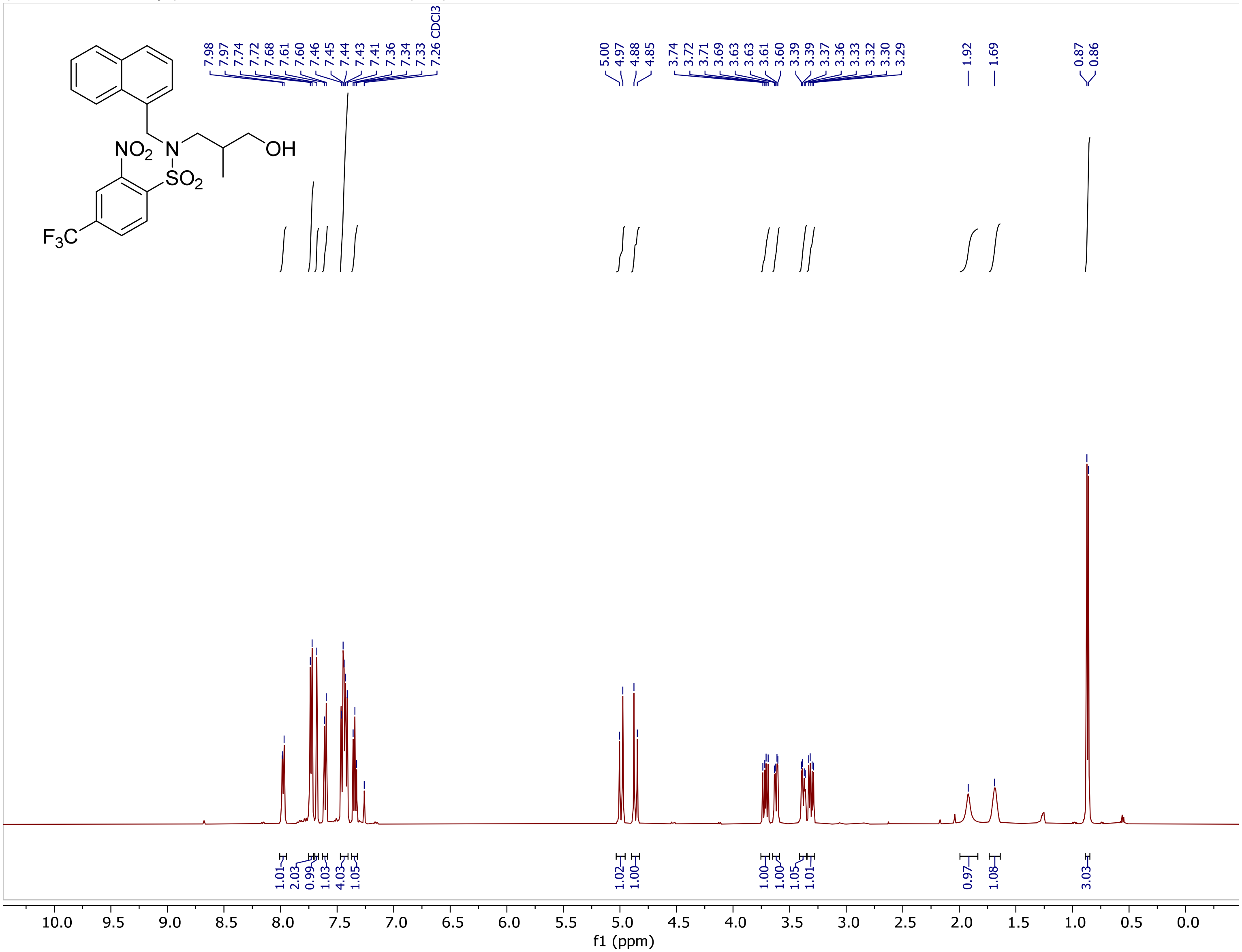

^{13}C {^{1}H} NMR (126 MHz, $CDCl_3$) spectrum of *N*-(3-Hydroxy-2-methylpropyl)-*N*-(naphthalen-1-ylmethyl)-2-nitro-4-(trifluoromethyl)benzenesulfonamide (**3b**)

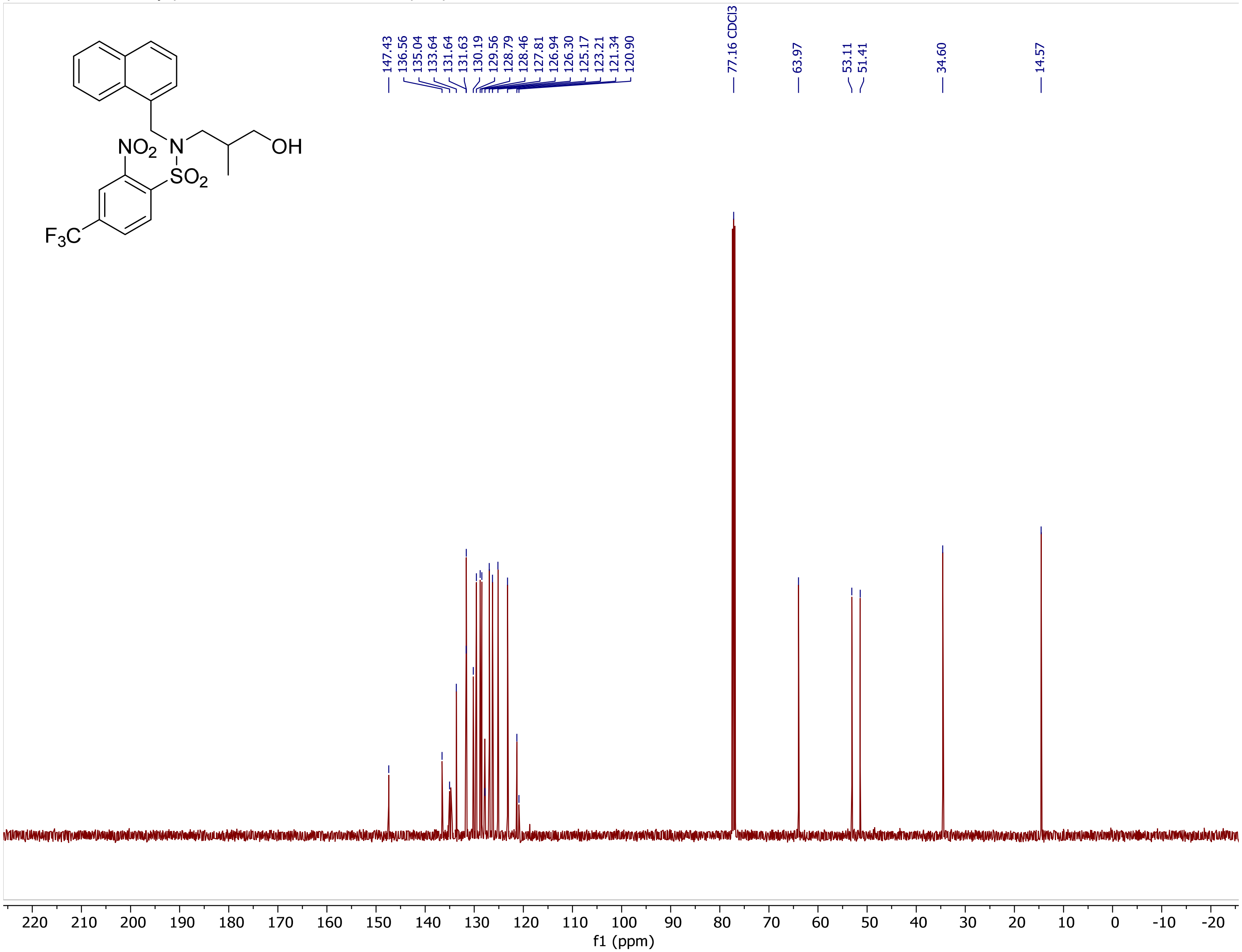

HSQC NMR (500 MHz, $CDCl_3$) spectrum of *N*-(3-Hydroxy-2-methylpropyl)-*N*-(naphthalen-1-ylmethyl)-2-nitro-4-(trifluoromethyl)benzenesulfonamide (**3b**)

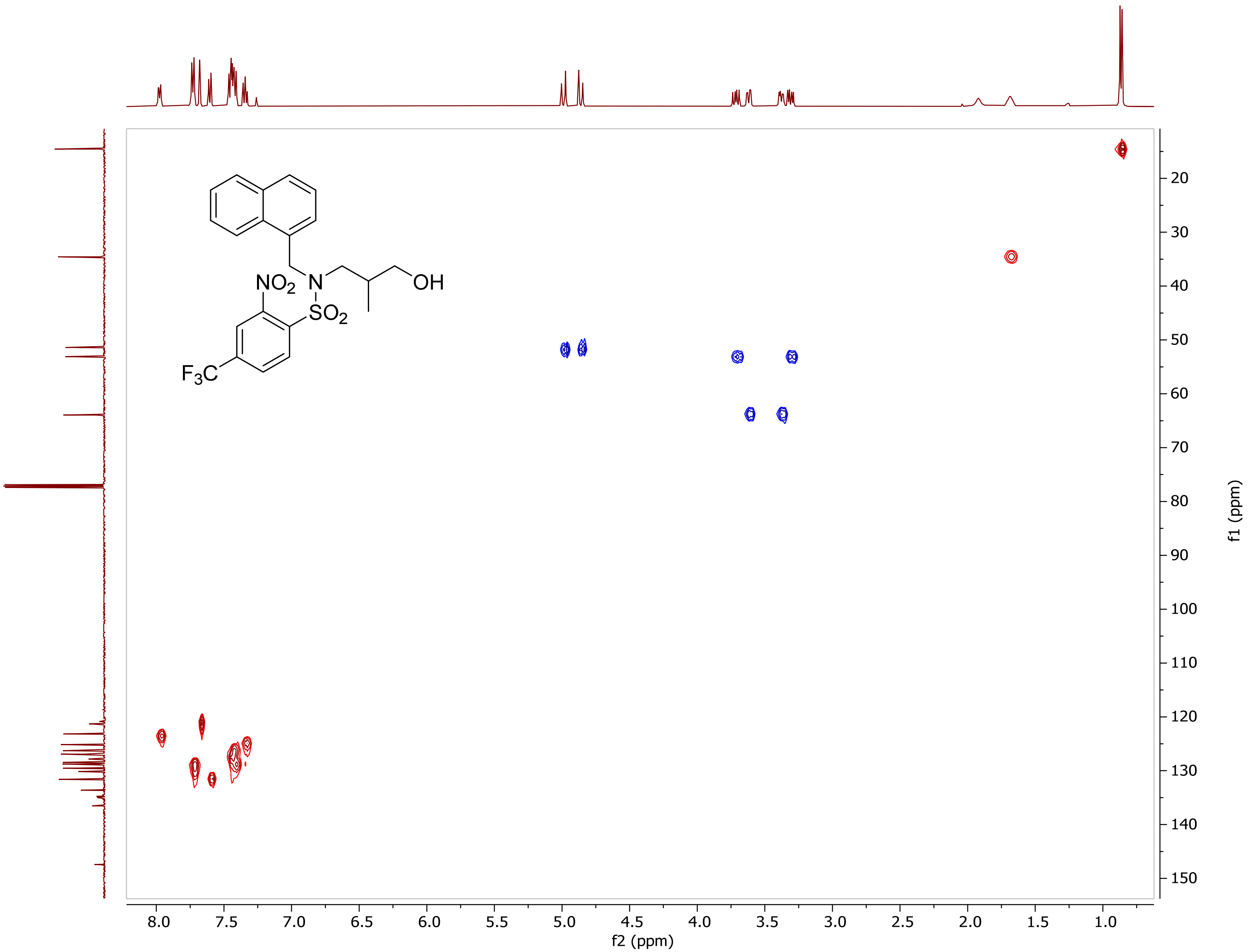

^{1}H NMR (500 MHz, CDCl$_3$) spectrum of *N*-(3-Iodo-2-methylpropyl)-*N*-(naphthalen-1-ylmethyl)-2-nitro-4-(trifluoromethyl)benzenesulfonamide (**3c**)

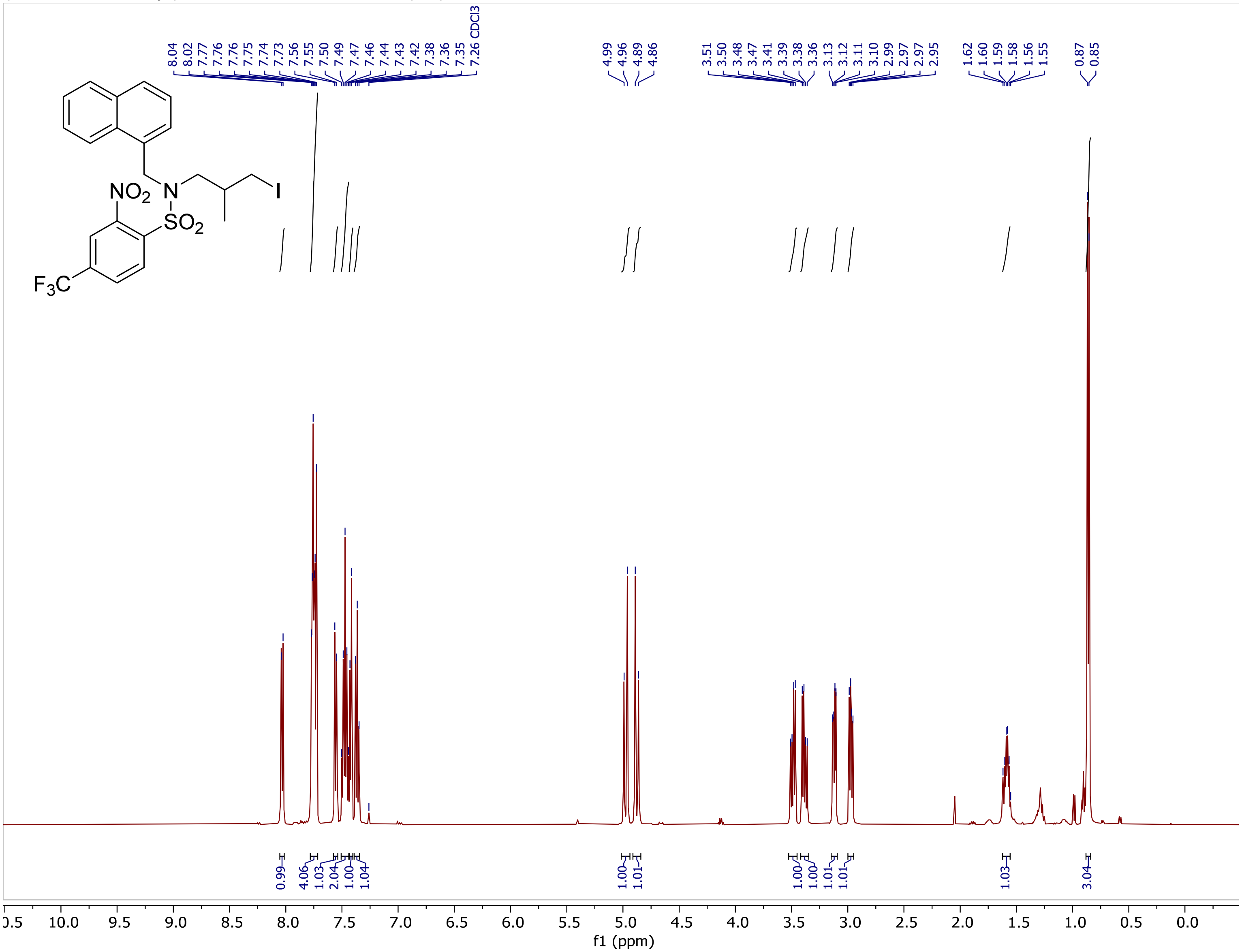

^{13}C {^{1}H} NMR (126 MHz, CDCl$_3$) spectrum of *N*-(3-Iodo-2-methylpropyl)-*N*-(naphthalen-1-ylmethyl)-2-nitro-4-(trifluoromethyl)benzenesulfonamide (**3c**)

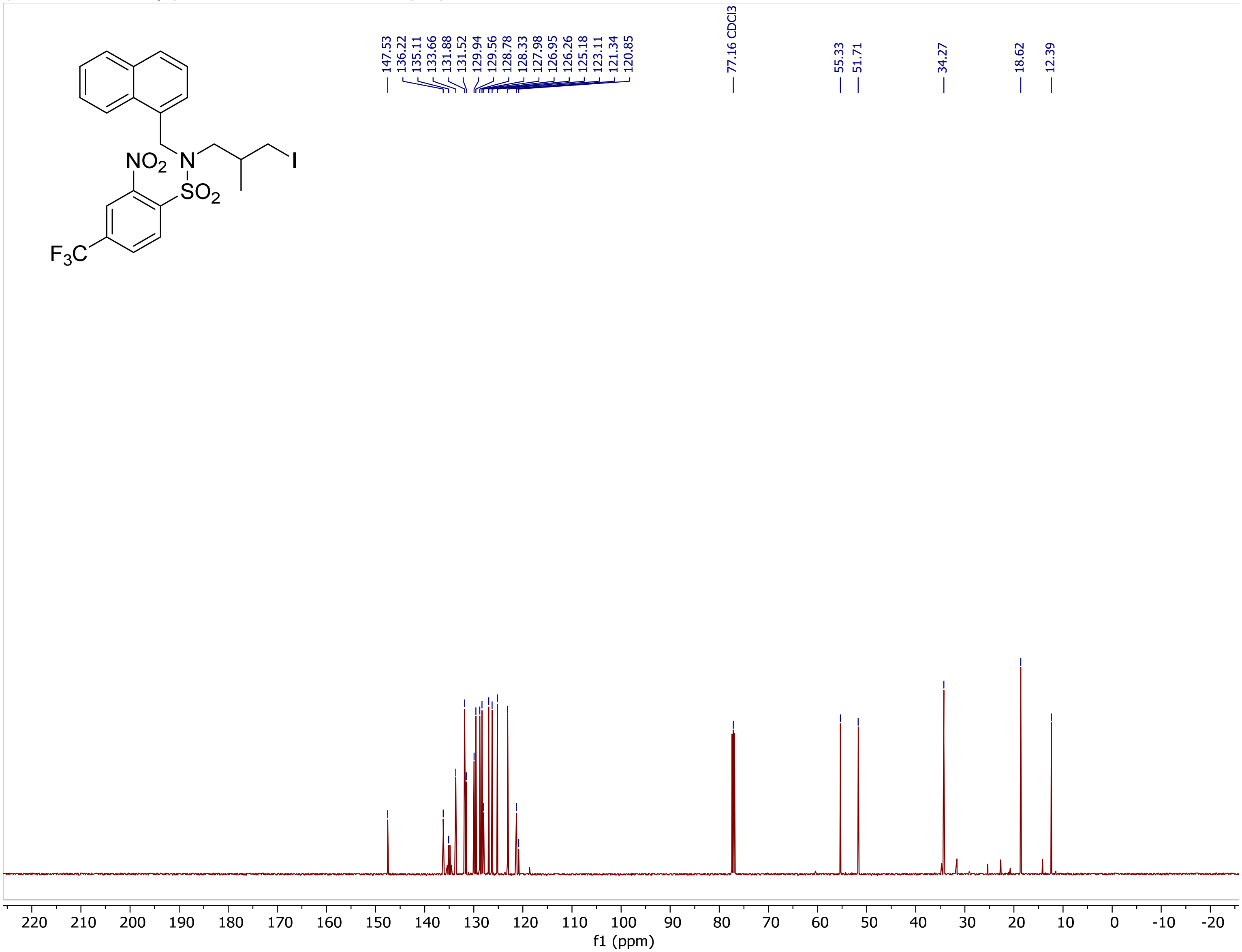

HSQC NMR (500 MHz, CDCl₃) spectrum of *N*-(3-Iodo-2-methylpropyl)-*N*-(naphthalen-1-ylmethyl)-2-nitro-4-(trifluoromethyl)benzenesulfonamide (**3c**)

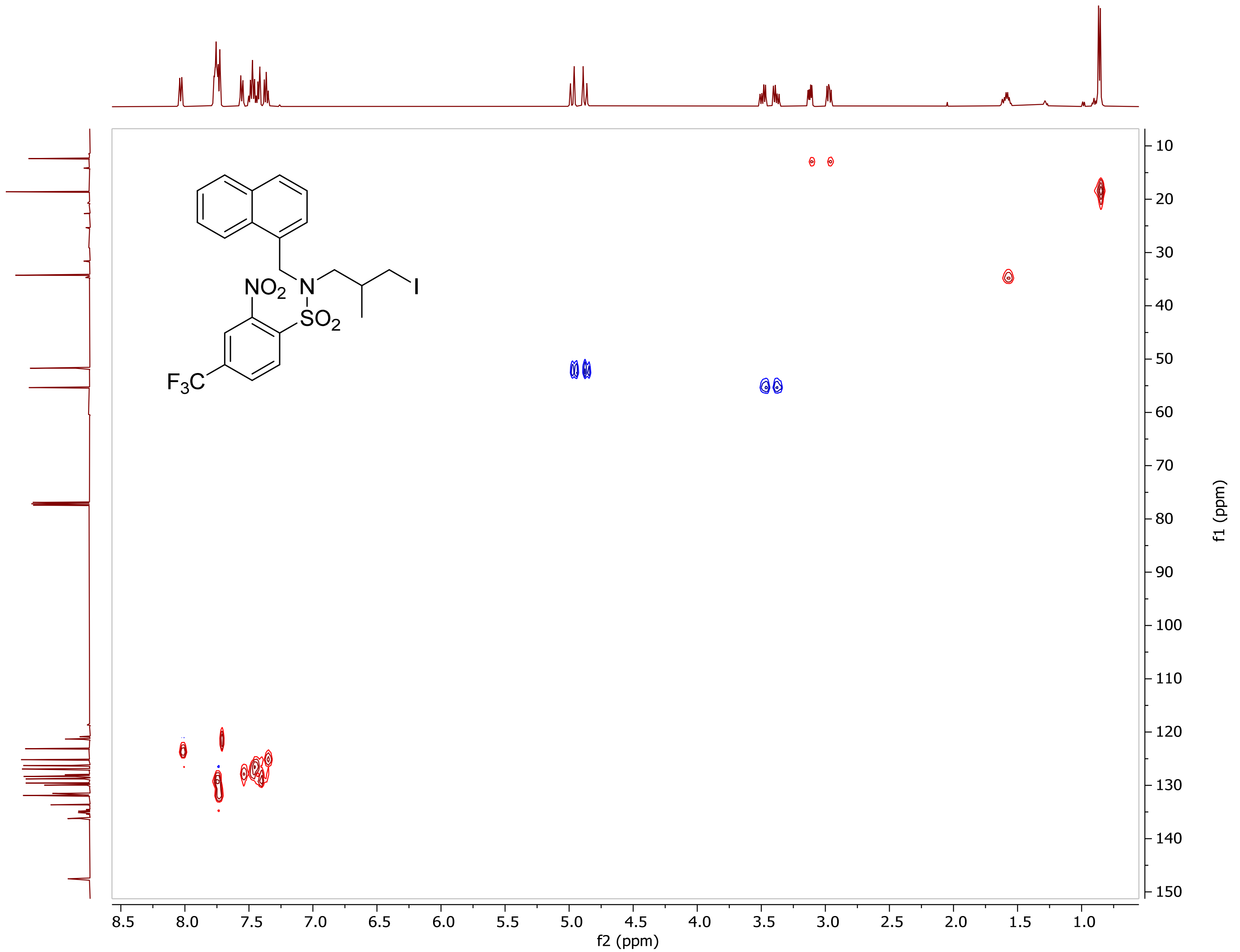

^{1}H NMR (500 MHz, $CDCl_3$) spectrum of *N*-(3-Hydroperoxy-2-methylpropyl)-*N*-(naphthalen-1-ylmethyl)-2-nitro-4-(trifluoromethyl)benzenesulfonamide (**3d**)

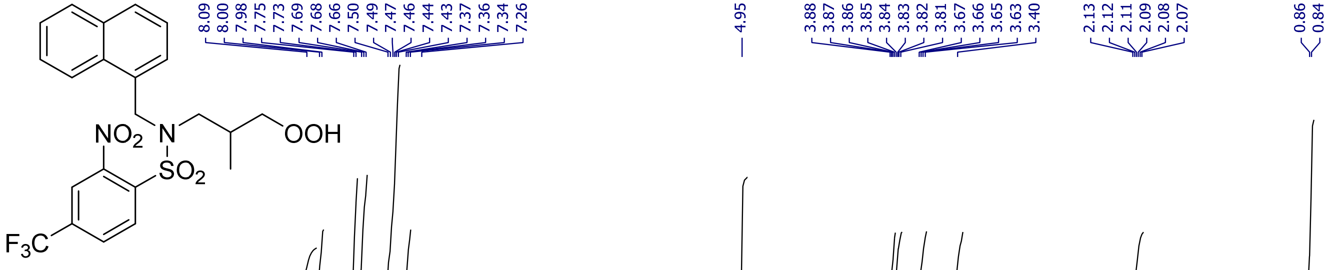

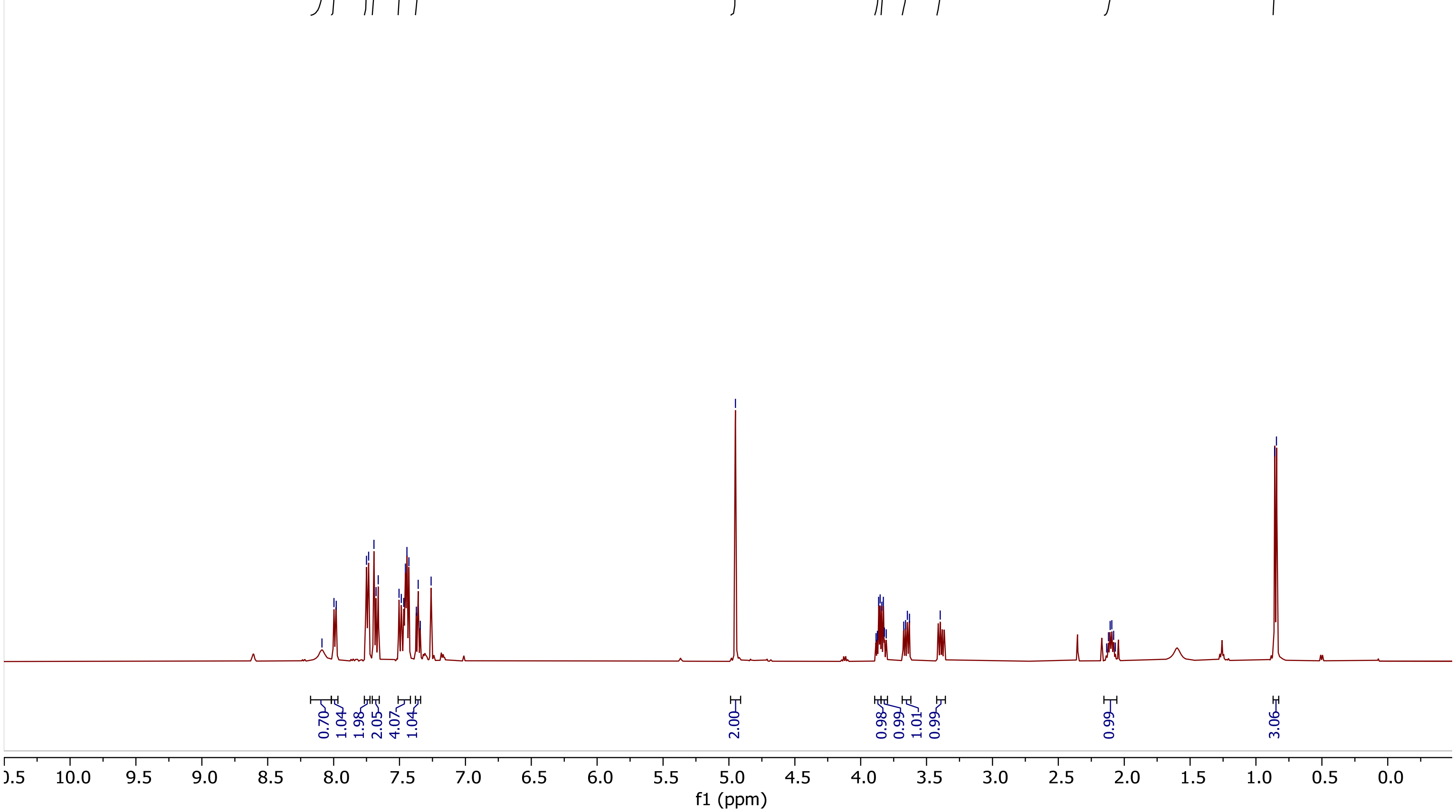

$^{13}C\ \{^1H\}$ NMR (126 MHz, $CDCl_3$) spectrum of *N*-(3-Hydroperoxy-2-methylpropyl)-*N*-(naphthalen-1-ylmethyl)-2-nitro-4-(trifluoromethyl)benzenesulfonamide (**3d**)

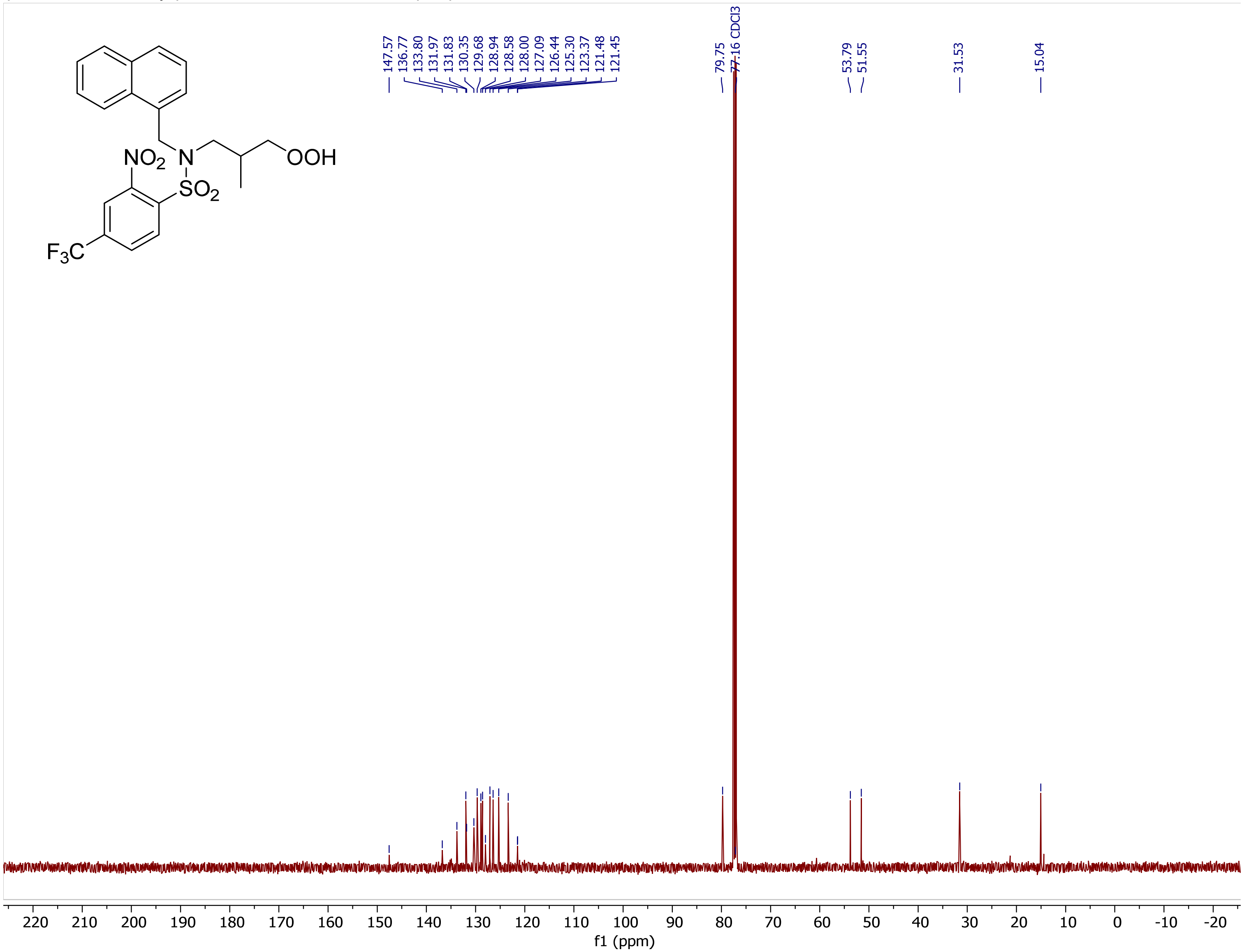

HSQC NMR (500 MHz, CDCl₃) spectrum of *N*-(3-Hydroperoxy-2-methylpropyl)-*N*-(naphthalen-1-ylmethyl)-2-nitro-4-(trifluoromethyl)benzenesulfonamide (**3d**)

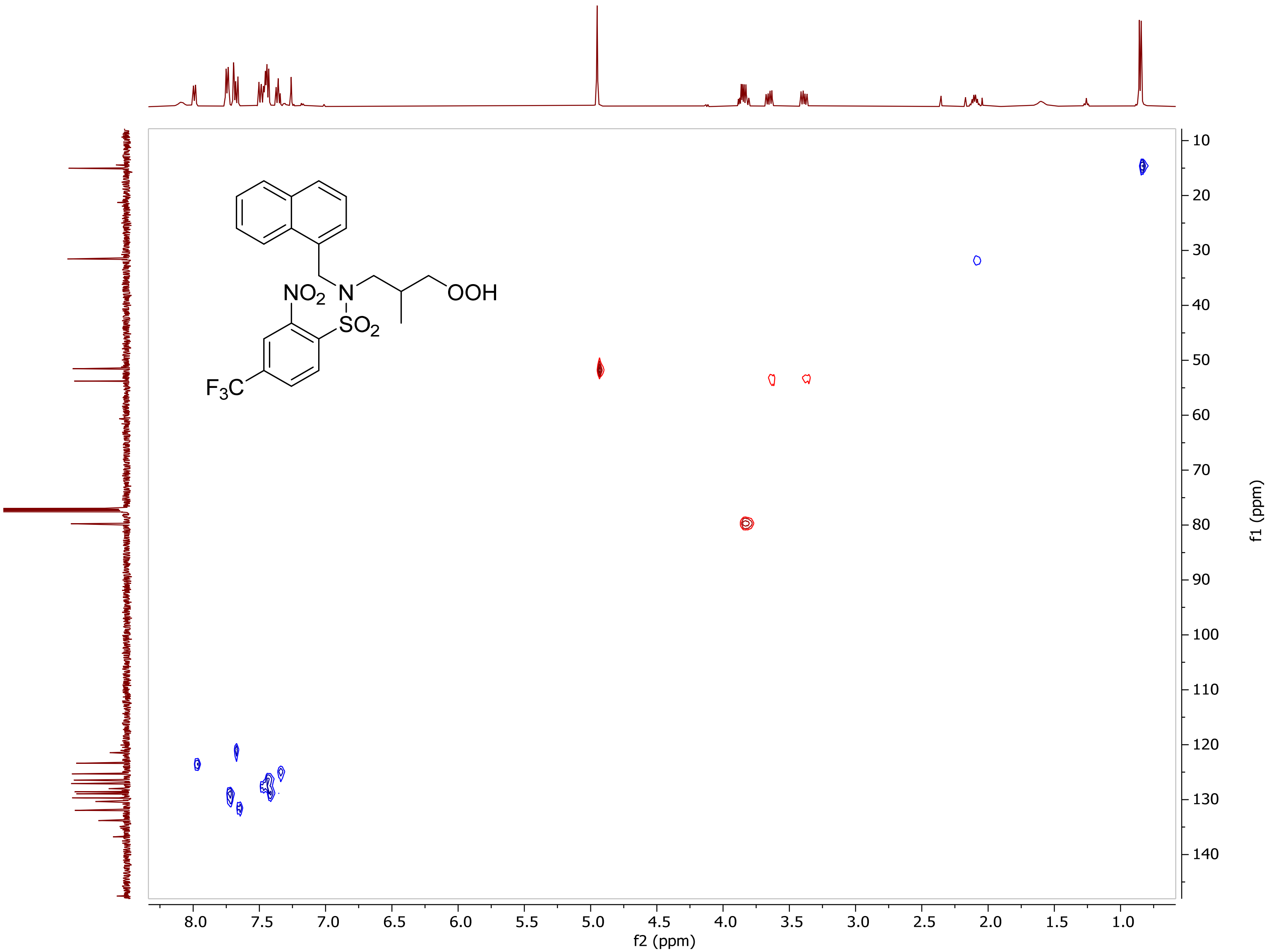

^{1}H NMR (500 MHz, C_6D_6) spectrum of *N*-(3-((*tert*-Butyldiphenylsilyl)peroxy)-2-methylpropyl)-*N*-(naphthalen-1-ylmethyl)-2-nitro-4-(trifluoromethyl)benzenesulfonamide (**40**)

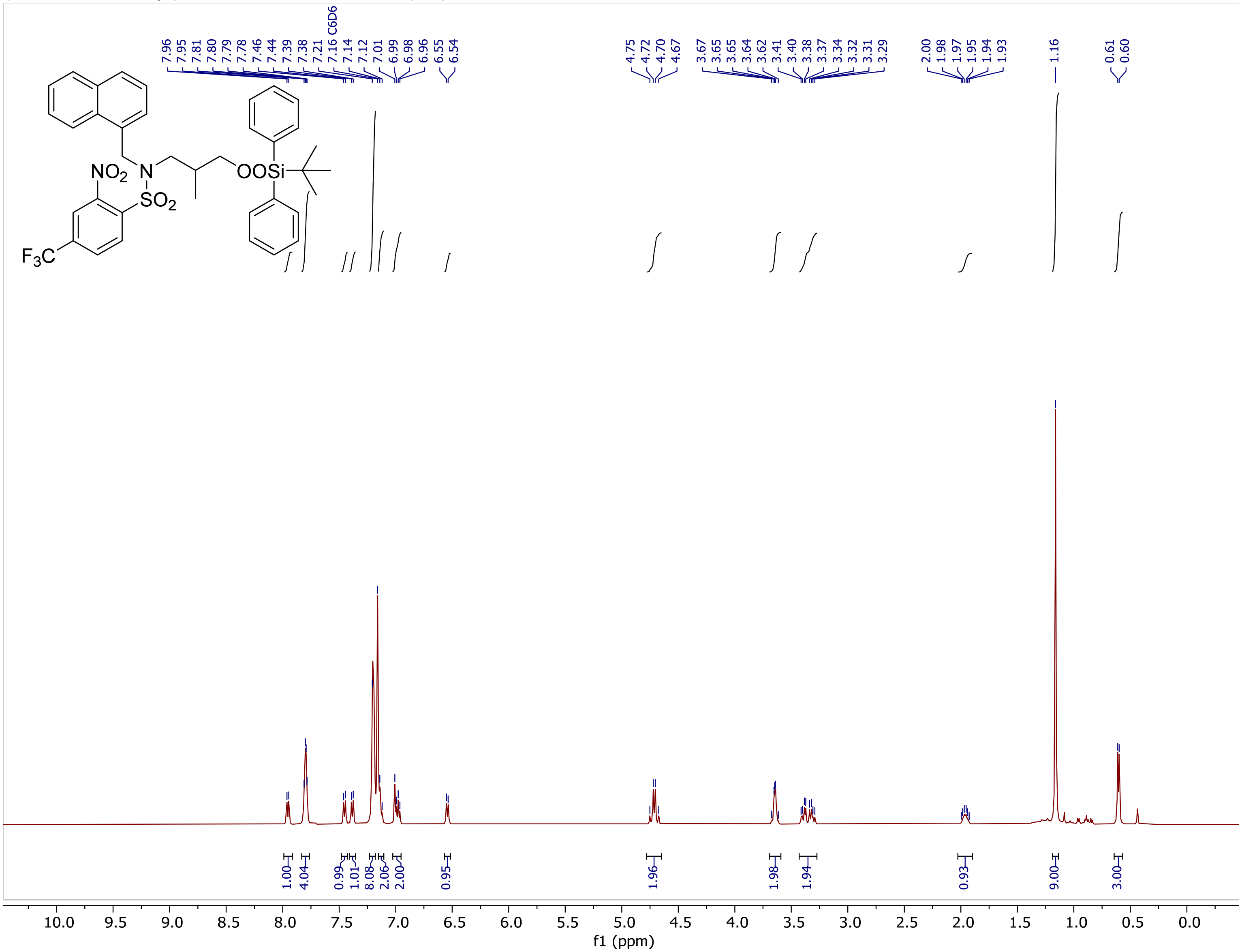

^{13}C {^{1}H} NMR (126 MHz, C_6D_6) spectrum of *N*-(3-((*tert*-Butyldiphenylsilyl)peroxy)-2-methylpropyl)-*N*-(naphthalen-1-ylmethyl)-2-nitro-4-(trifluoromethyl)benzenesulfonamide (**40**)

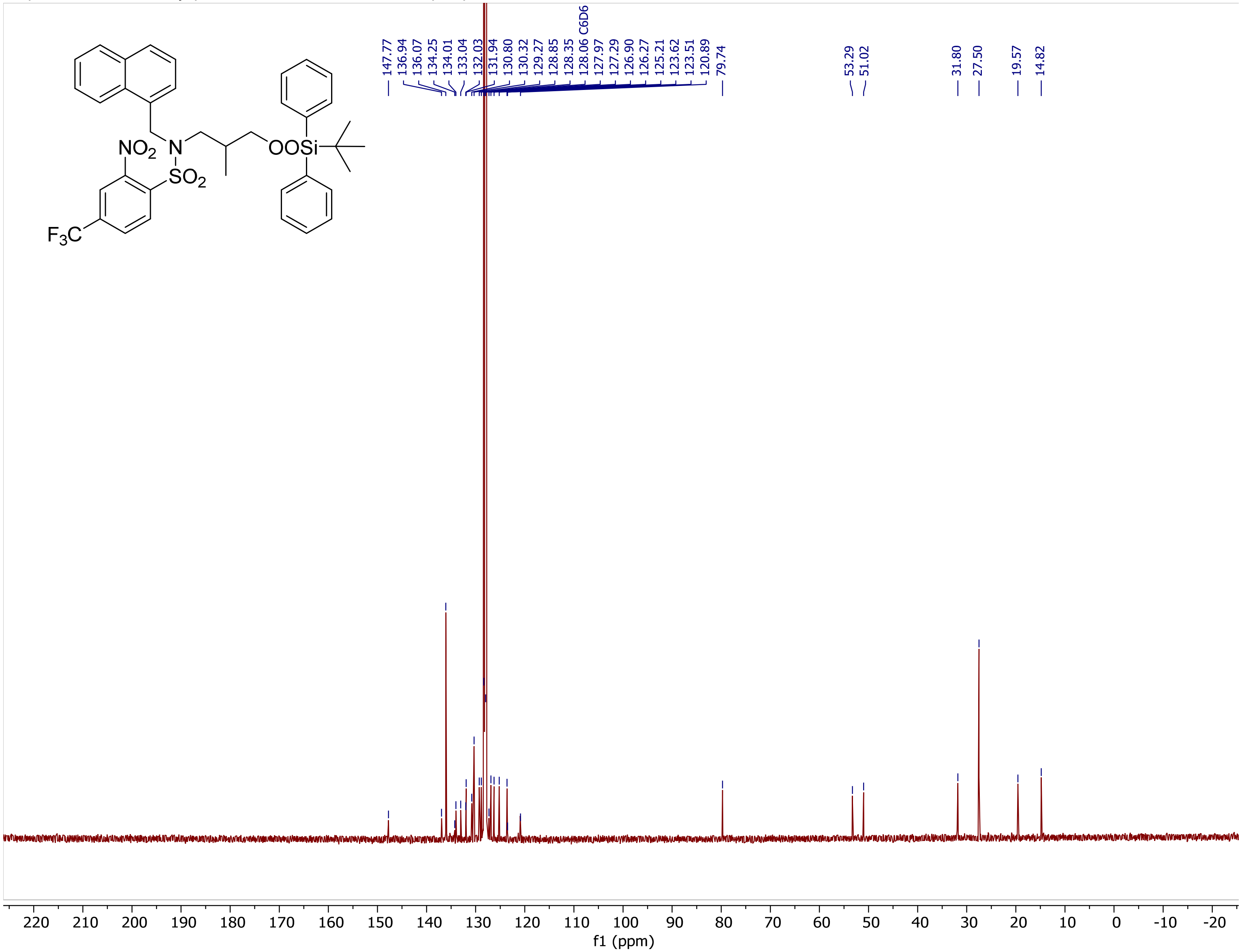

HSQC NMR (500 MHz, C_6D_6) spectrum of *N*-(3-((*tert*-Butyldiphenylsilyl)peroxy)-2-methylpropyl)-*N*-(naphthalen-1-ylmethyl)-2-nitro-4-(trifluoromethyl)benzenesulfonamide (**40**)

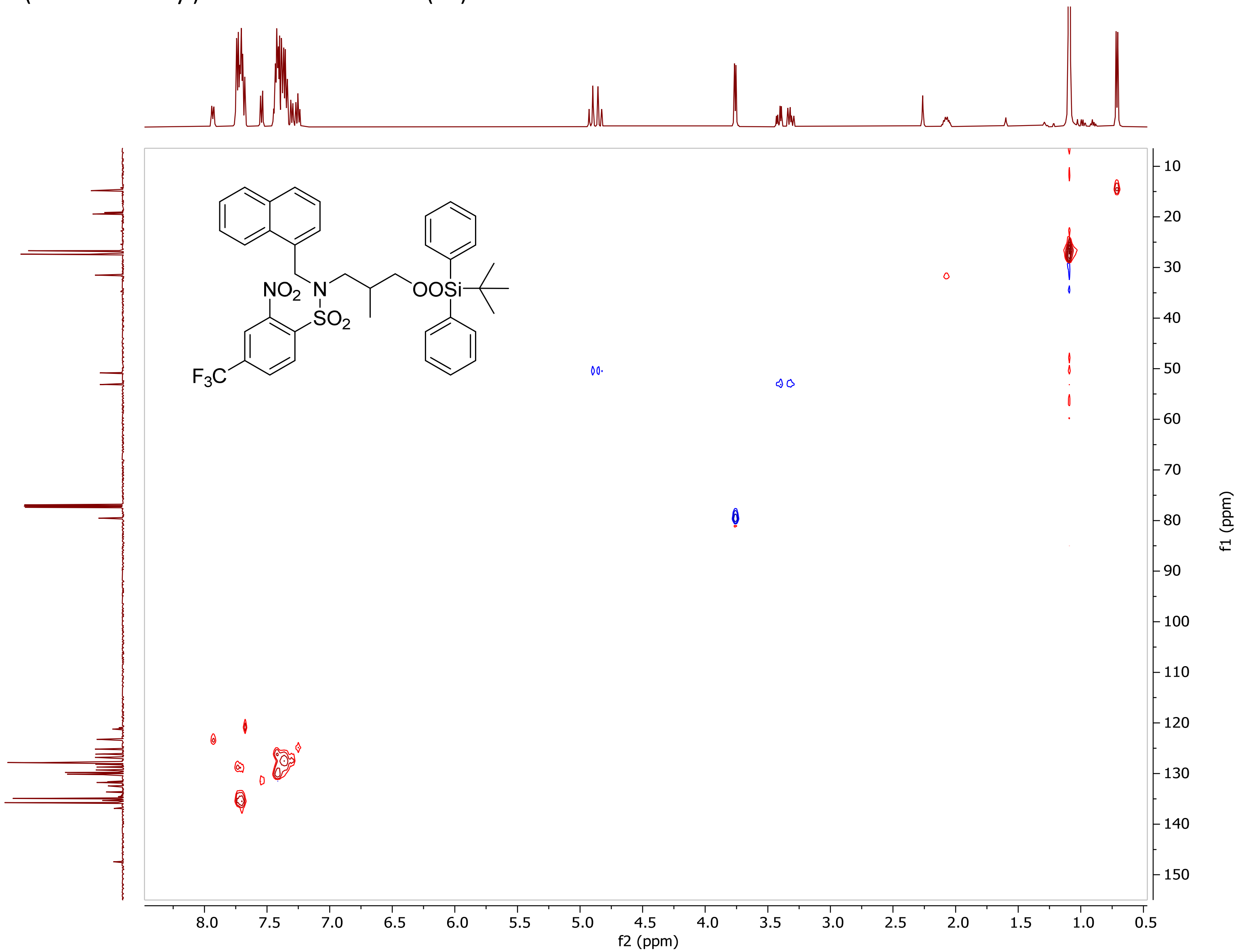

^{1}H NMR (500 MHz, C_6D_6) spectrum of 4-Methyl-2-(naphthalen-1-ylmethyl)isoxazolidine (**41**)

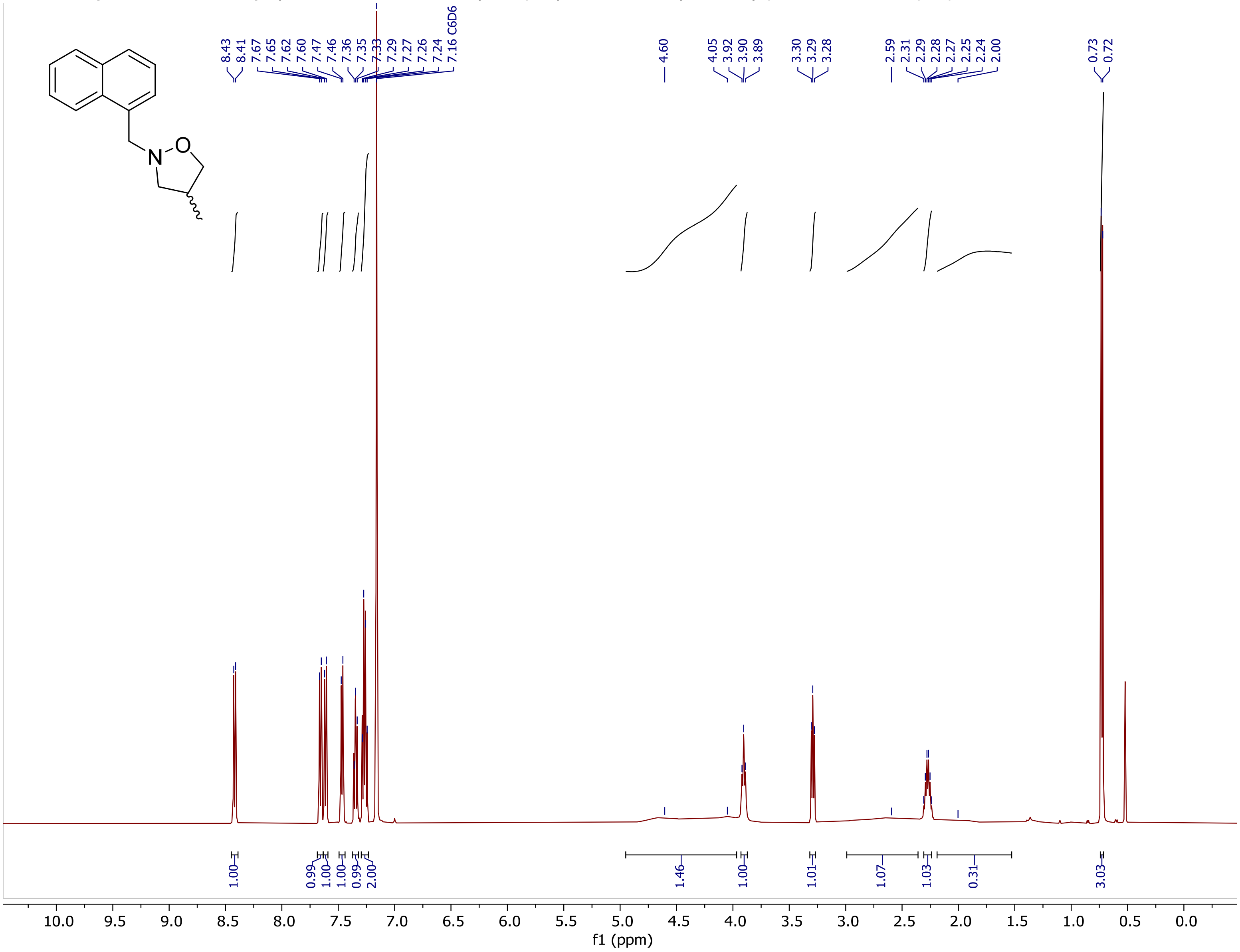

^{1}H NMR (500 MHz, C_6D_6) spectrum of 4-Methyl-2-(naphthalen-1-ylmethyl)isoxazolidine at 60 °C (**41**)

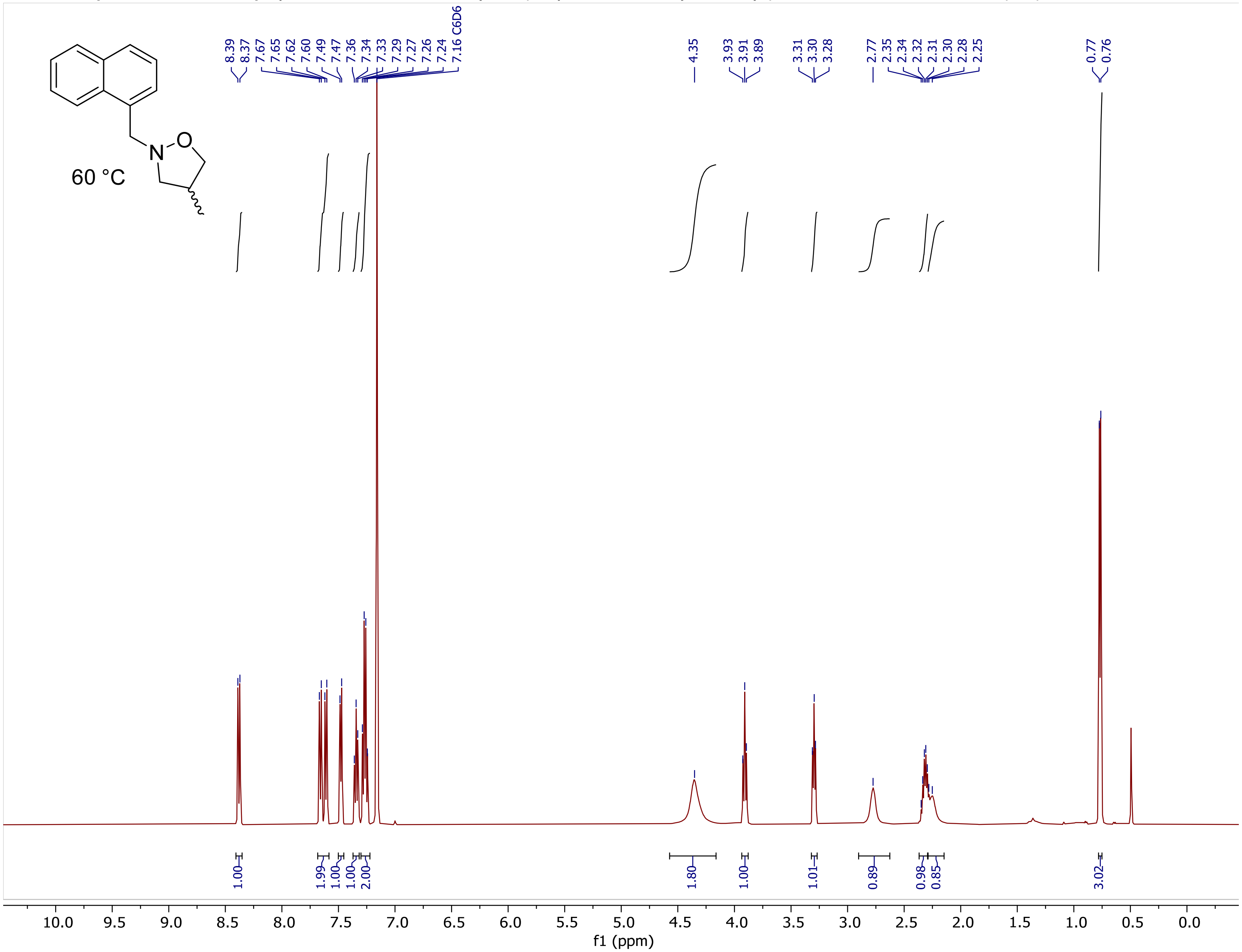

^{13}**C {^{1}H} NMR (126 MHz, C_6D_6)** spectrum of 4-Methyl-2-(naphthalen-1-ylmethyl)isoxazolidine (**41**)

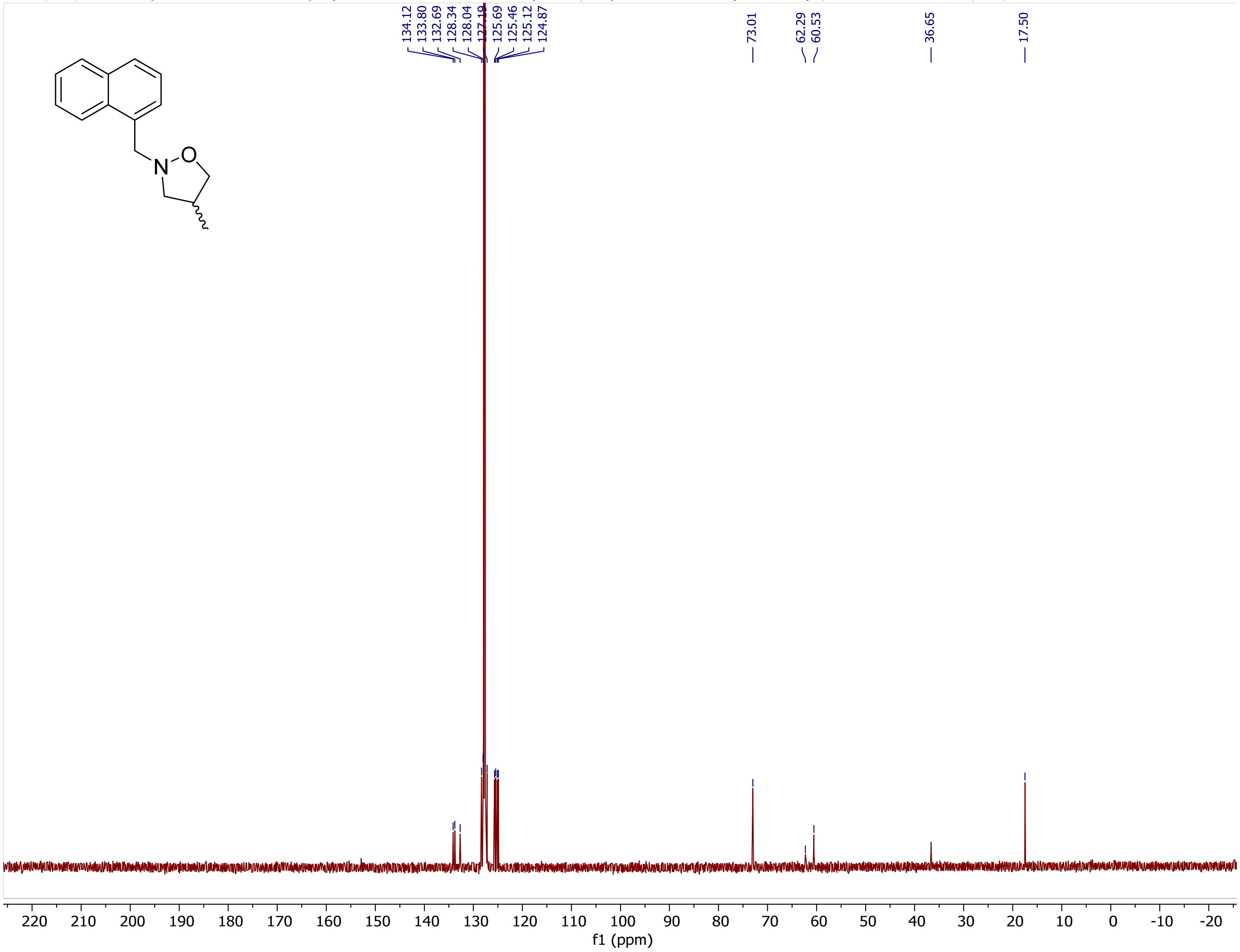

HSQC NMR (500 MHz, C_6D_6) spectrum of 4-Methyl-2-(naphthalen-1-ylmethyl)isoxazolidine at 60 °C (**41**)

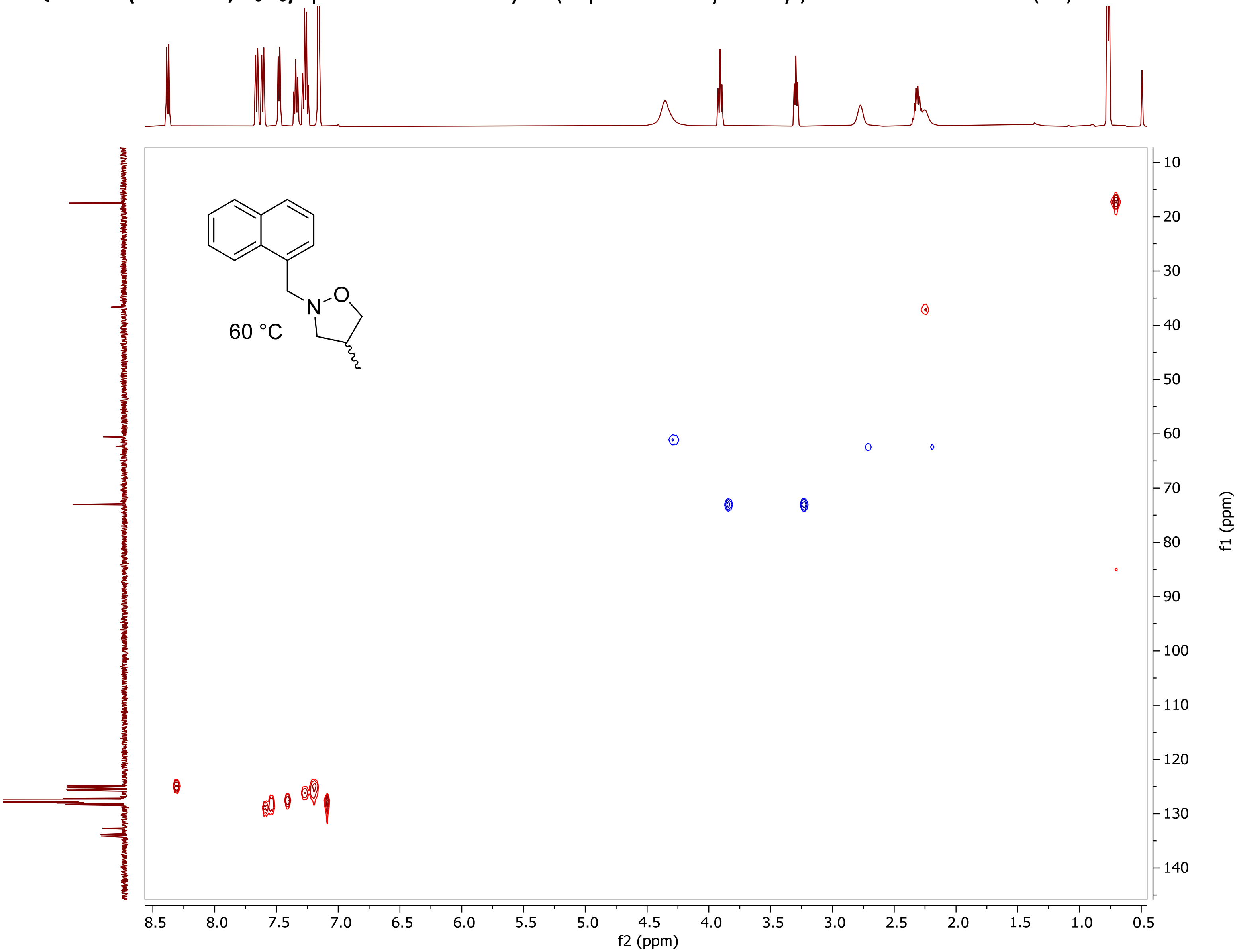

^{1}H NMR (500 MHz, CDCl$_3$) spectrum of *N*-Allyl-*N*-(naphthalen-1-ylmethyl)-2-nitro-4-(trifluoromethyl)benzenesulfonamide (**3e**)

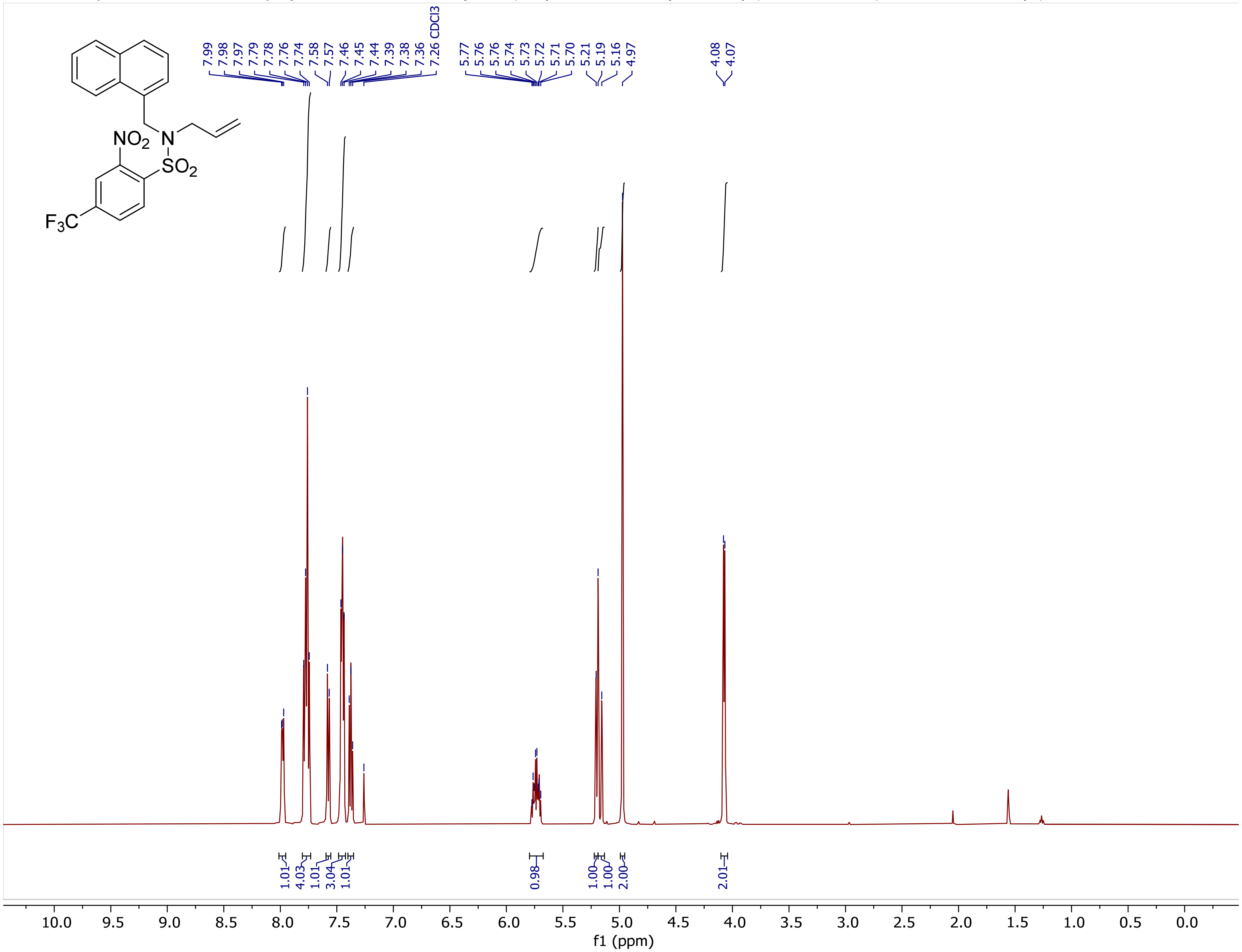

^{13}C {^{1}H} NMR (126 MHz, $CDCl_3$) spectrum of *N*-Allyl-*N*-(naphthalen-1-ylmethyl)-2-nitro-4-(trifluoromethyl)benzenesulfonamide (**3e**)

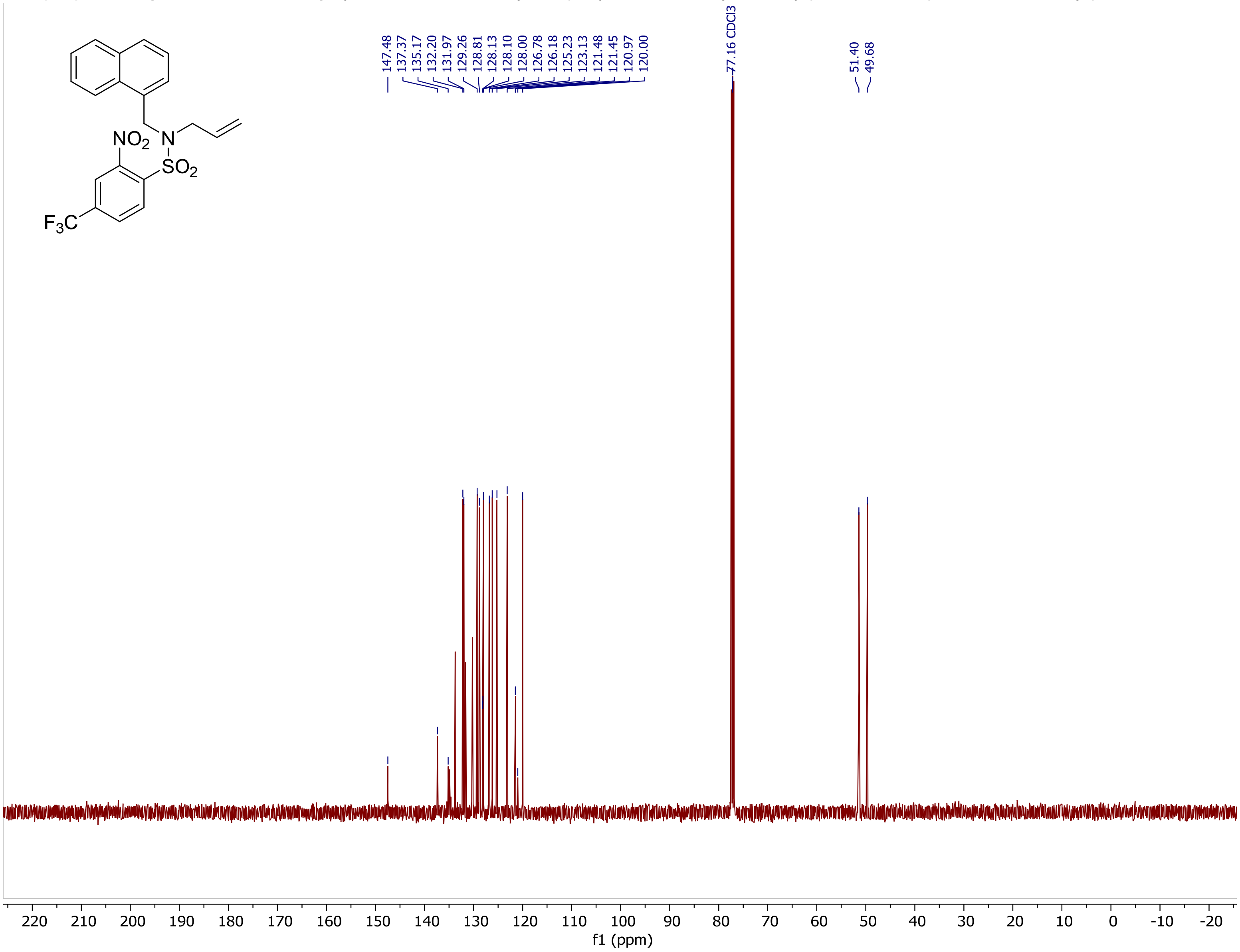

^{1}H NMR (500 MHz, CDCl$_3$) spectrum of *N*-(3-Hydroxypropyl)-*N*-(naphthalen-1-ylmethyl)-2-nitro-4-(trifluoromethyl)benzenesulfonamide (**3f**)

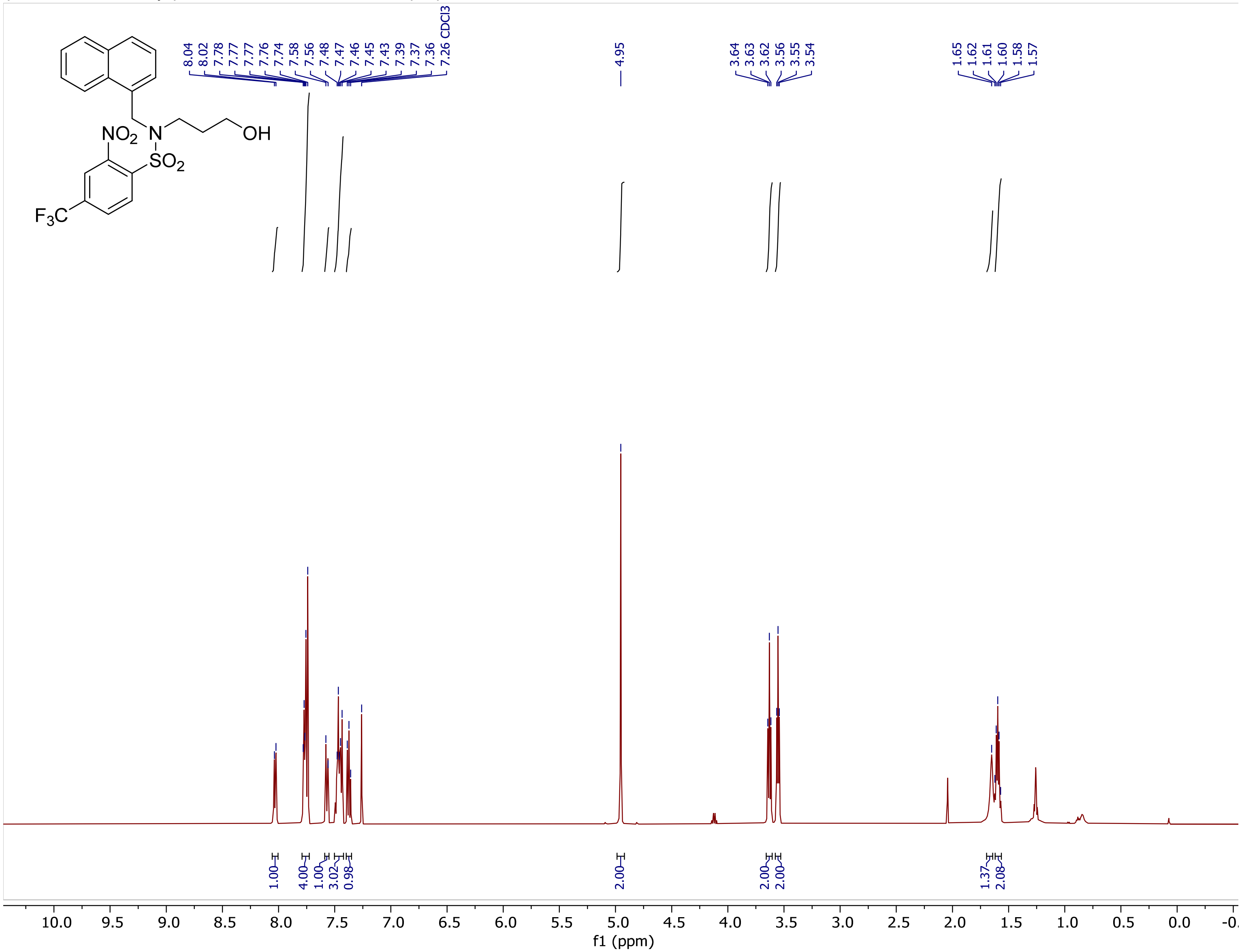

^{13}C {^{1}H} NMR (126 MHz, CDCl$_3$) spectrum of *N*-(3-Hydroxypropyl)-*N*-(naphthalen-1-ylmethyl)-2-nitro-4-(trifluoromethyl)benzenesulfonamide (**3f**)

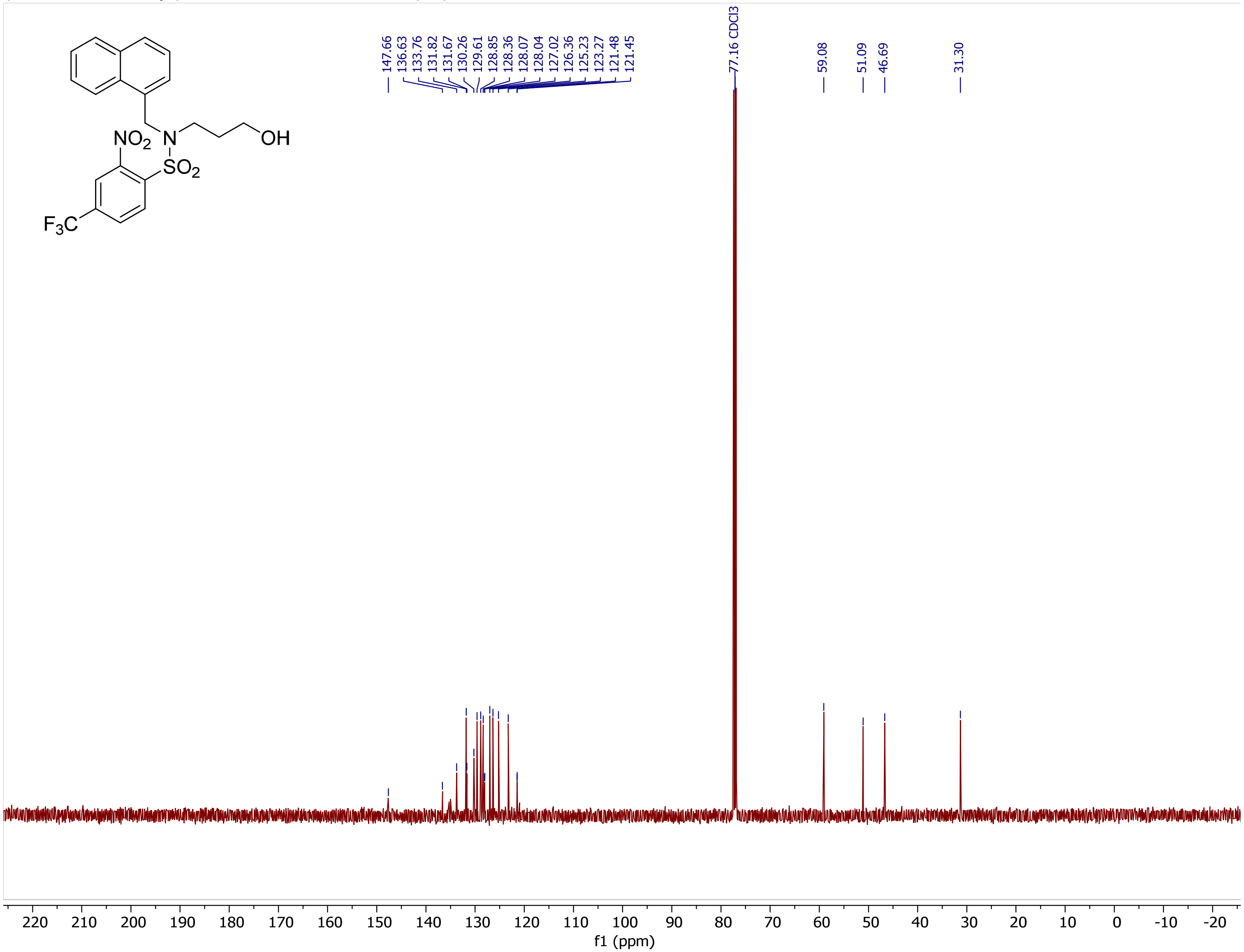

^{1}H NMR (500 MHz, CDCl$_3$) spectrum of *N*-(3-Iodopropyl)-*N*-(naphthalen-1-ylmethyl)-2-nitro-4-(trifluoromethyl)benzenesulfonamide (**3g**)

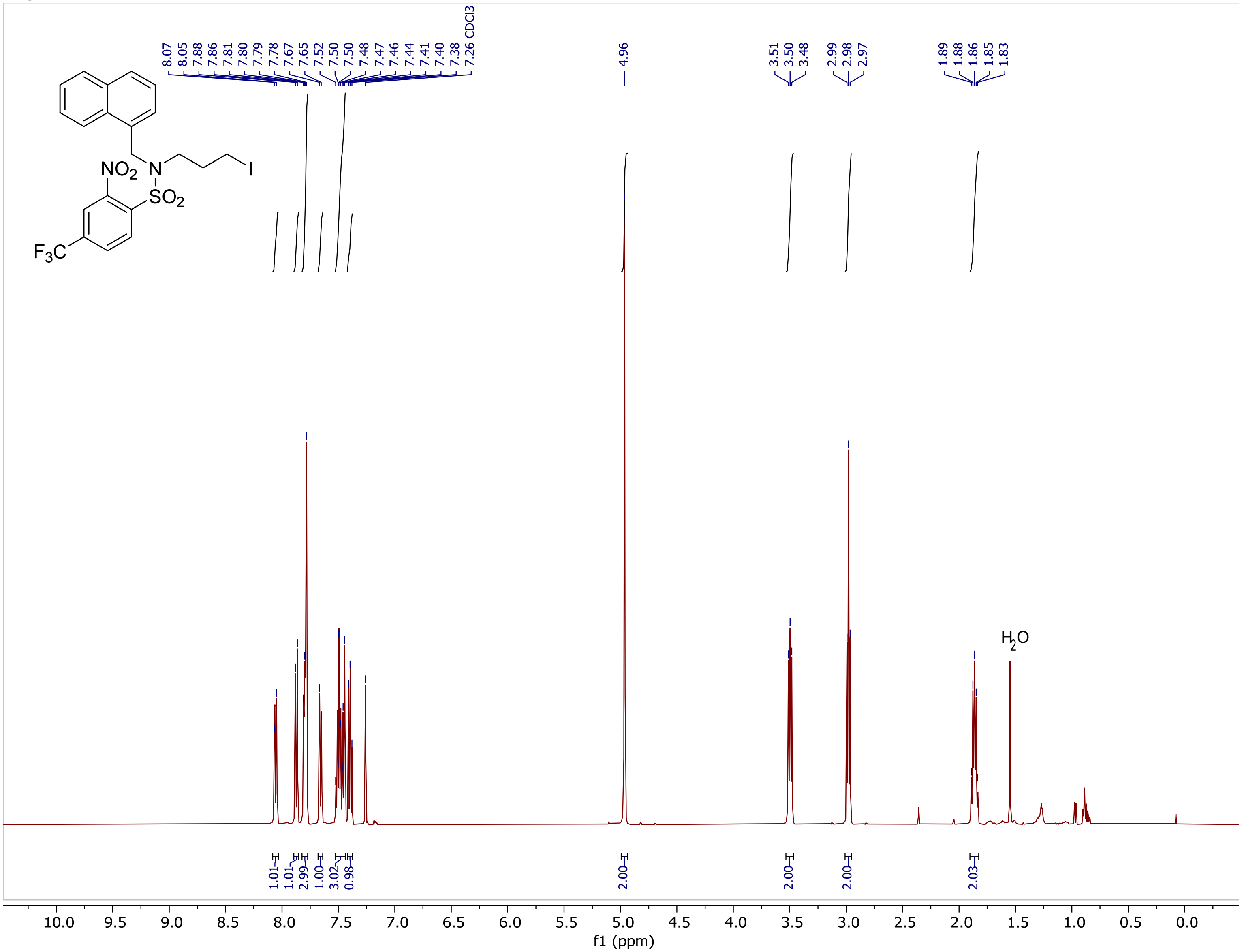

$^{13}C\{^{1}H\}$ NMR (126 MHz, CDCl$_3$) spectrum of *N*-(3-Iodopropyl)-*N*-(naphthalen-1-ylmethyl)-2-nitro-4-(trifluoromethyl)benzenesulfonamide (**3g**)

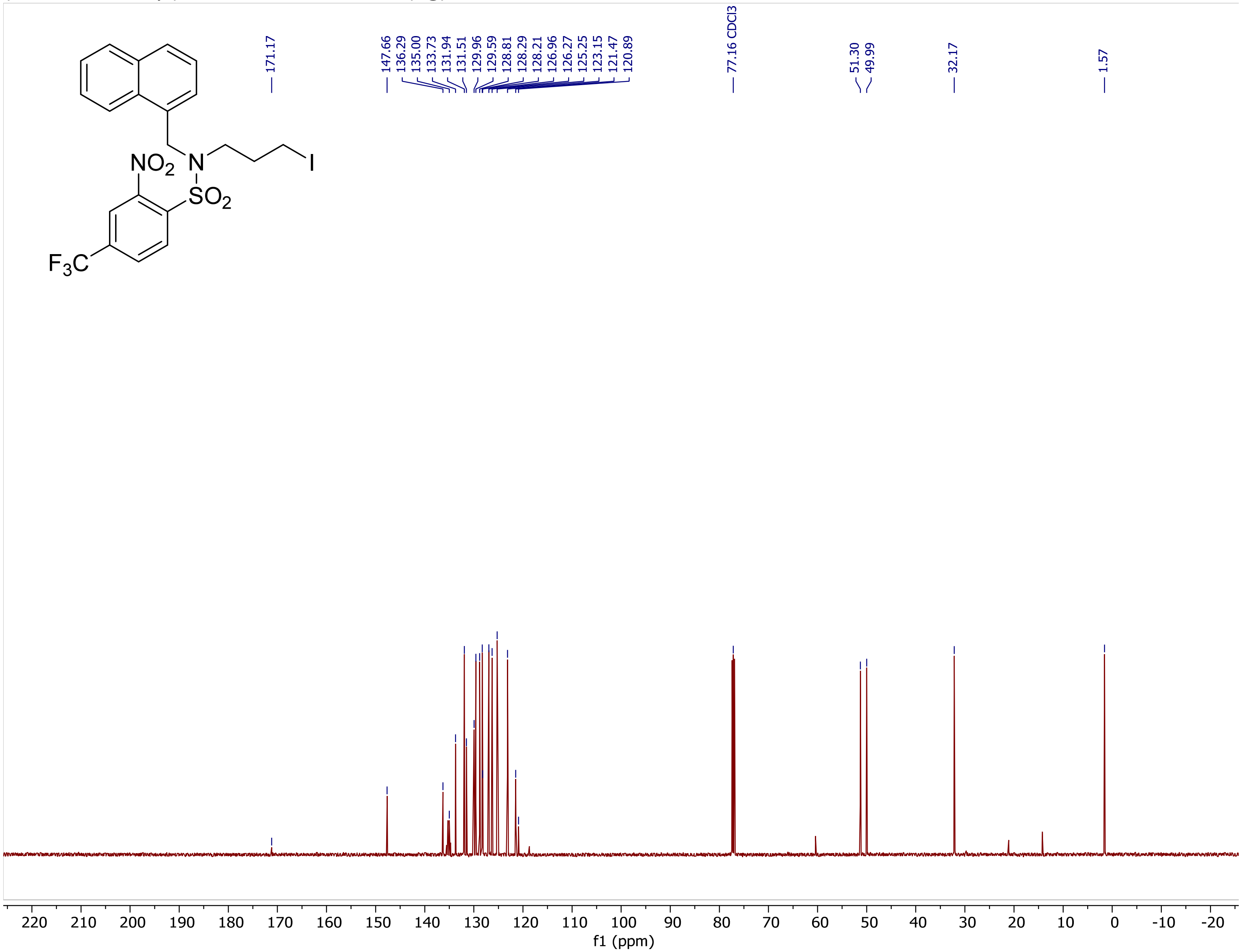

¹H NMR (500 MHz, CDCl₃) spectrum of *N*-(3-Hydroperoxypropyl)-*N*-(naphthalen-1-ylmethyl)-2-nitro-4-(trifluoromethyl)benzenesulfonamide (**3h**)

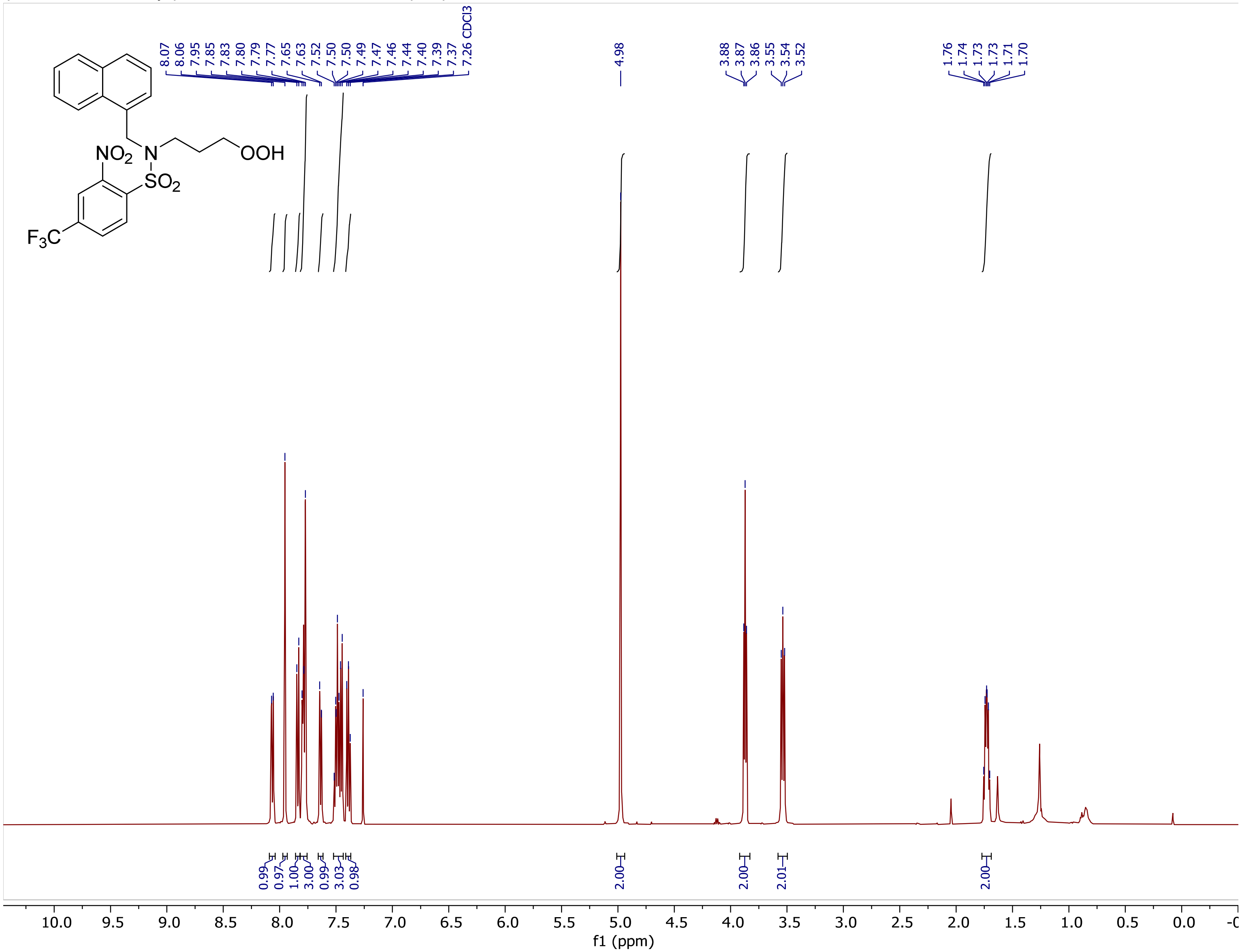

^{13}C {^{1}H} NMR (126 MHz, $CDCl_3$) spectrum of *N*-(3-Hydroperoxypropyl)-*N*-(naphthalen-1-ylmethyl)-2-nitro-4-(trifluoromethyl)benzenesulfonamide (**3h**)

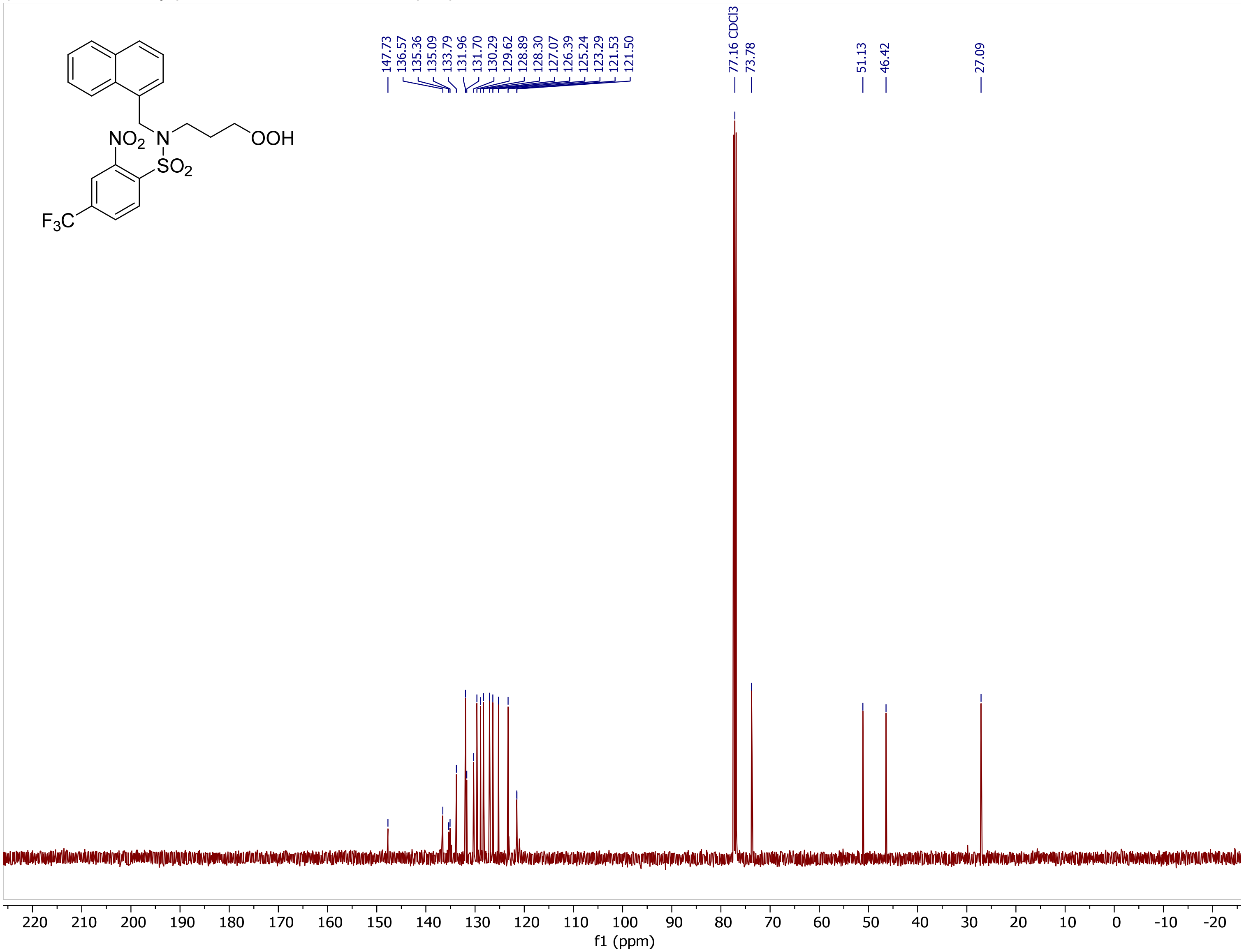

^{1}**H NMR (500 MHz, C_6D_6)** spectrum of *N*-(3-((*tert*-Butyldiphenylsilyl)peroxy)propyl)-*N*-(naphthalen-1-ylmethyl)-2-nitro-4-(trifluoromethyl)benzenesulfonamide (**42**)

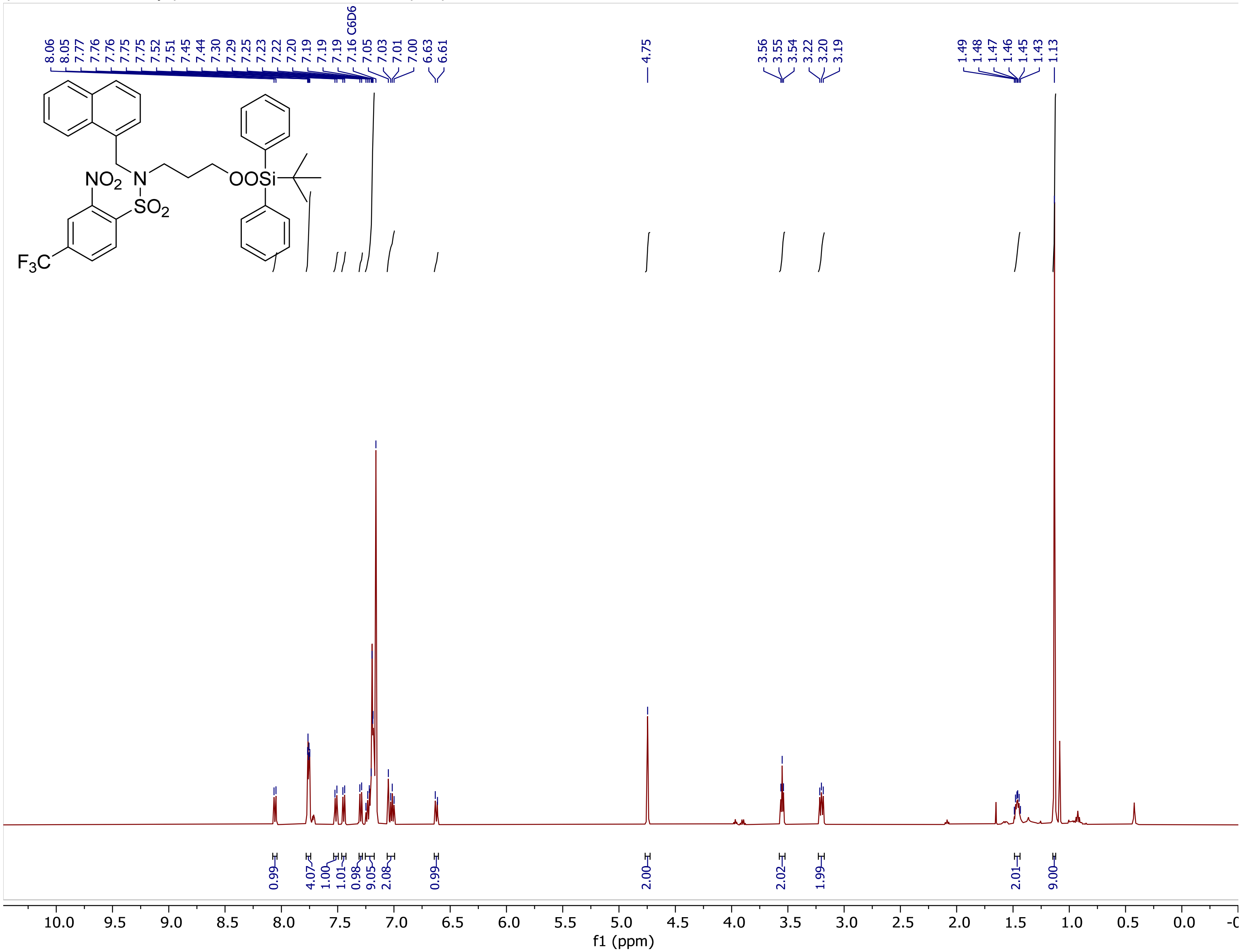

$^{13}C\ \{^1H\}$ **NMR (126 MHz, C_6D_6)** spectrum of *N*-(3-((*tert*-Butyldiphenylsilyl)peroxy)propyl)-*N*-(naphthalen-1-ylmethyl)-2-nitro-4-(trifluoromethyl)benzenesulfonamide (**42**)

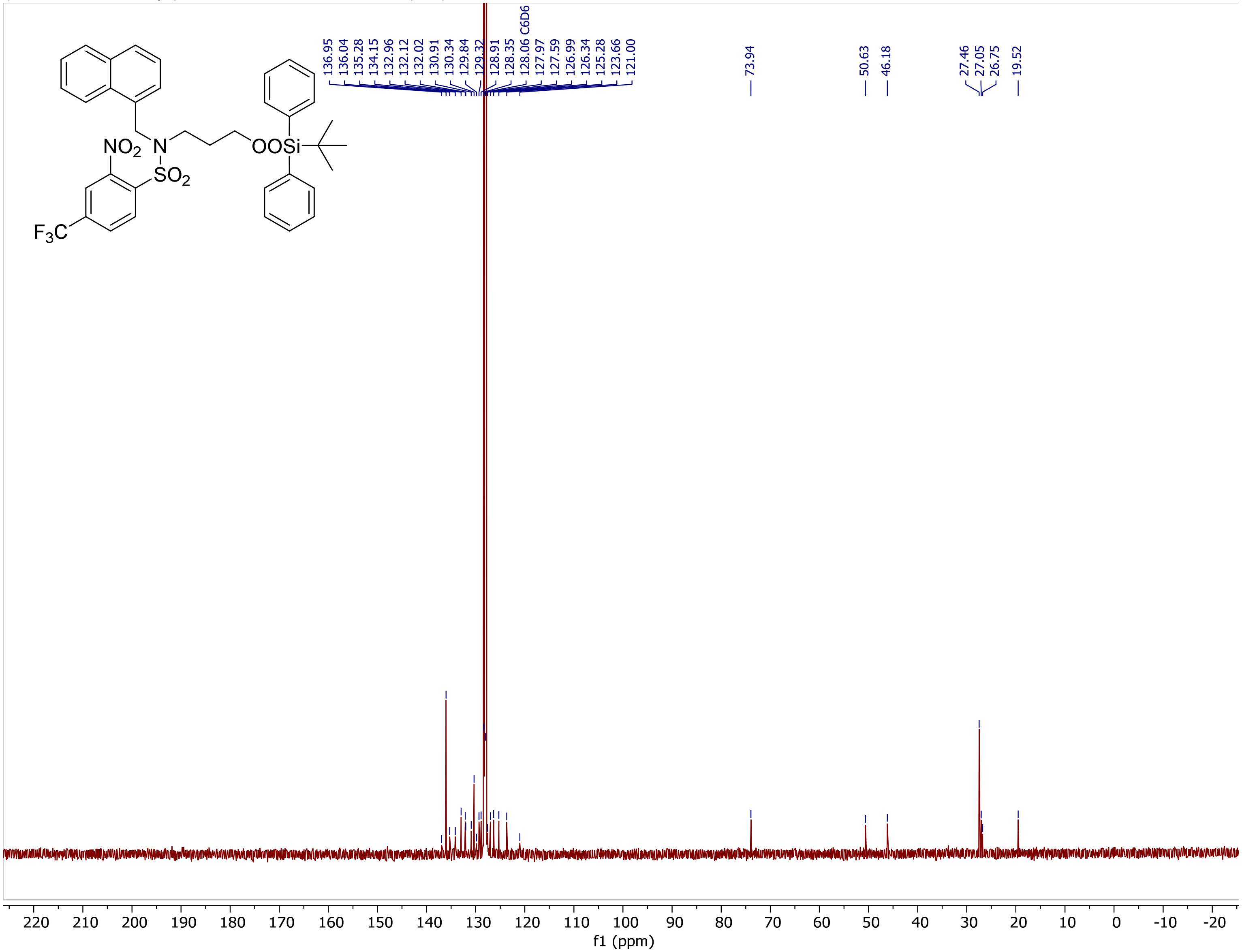

^{1}H NMR (500 MHz, C_6D_6) spectrum of 2-Phenylisoxazolidine (**43**)

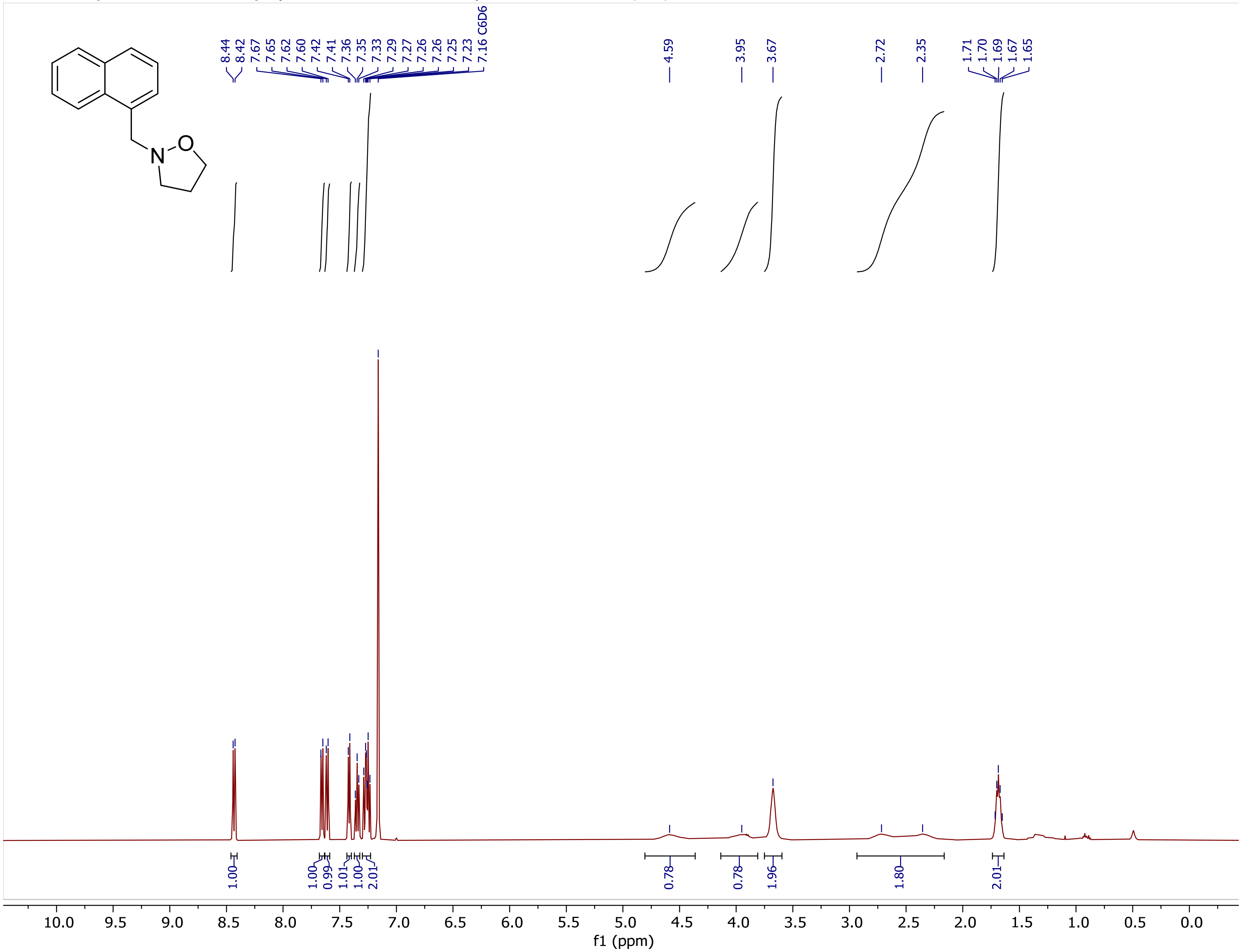

^{1}H NMR (500 MHz, C_6D_6) spectrum of 2-Phenylisoxazolidine at 65 °C (**43**)

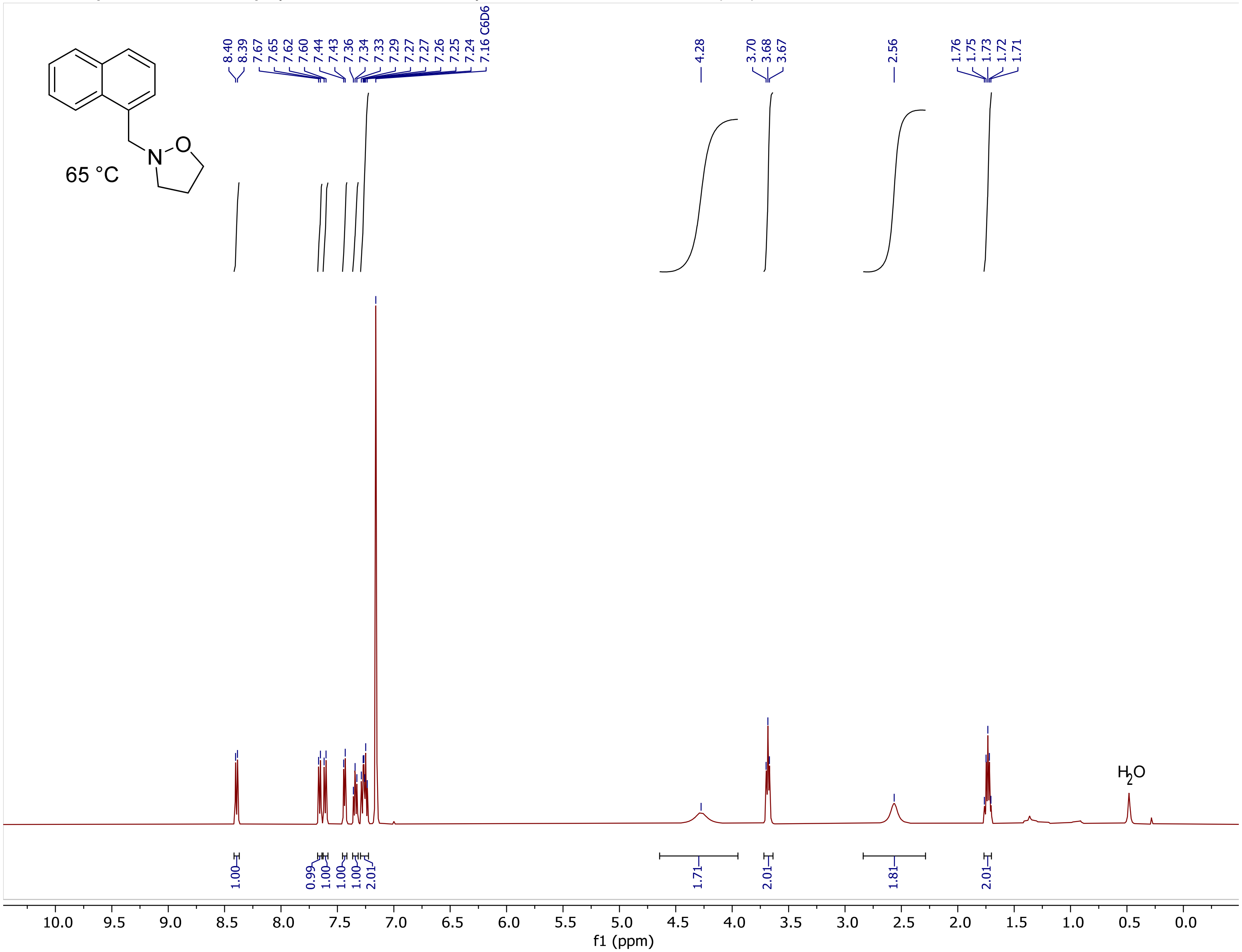

^{13}C {^{1}H} NMR (126 MHz, C_6D_6) spectrum of 2-Phenylisoxazolidine (**43**)

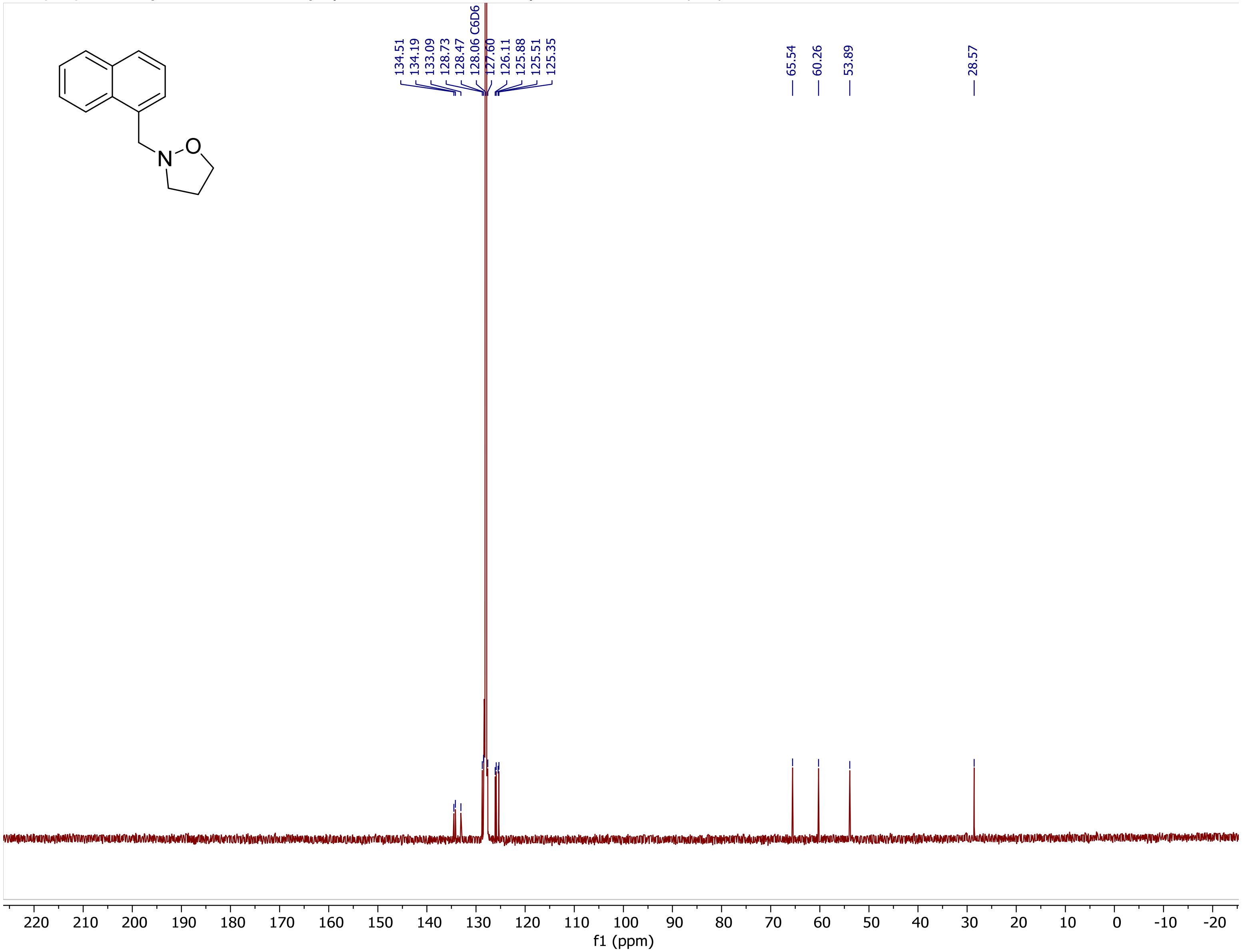

HSQC NMR (500 MHz, C_6D_6) spectrum of 2-Phenylisoxazolidine (**43**)

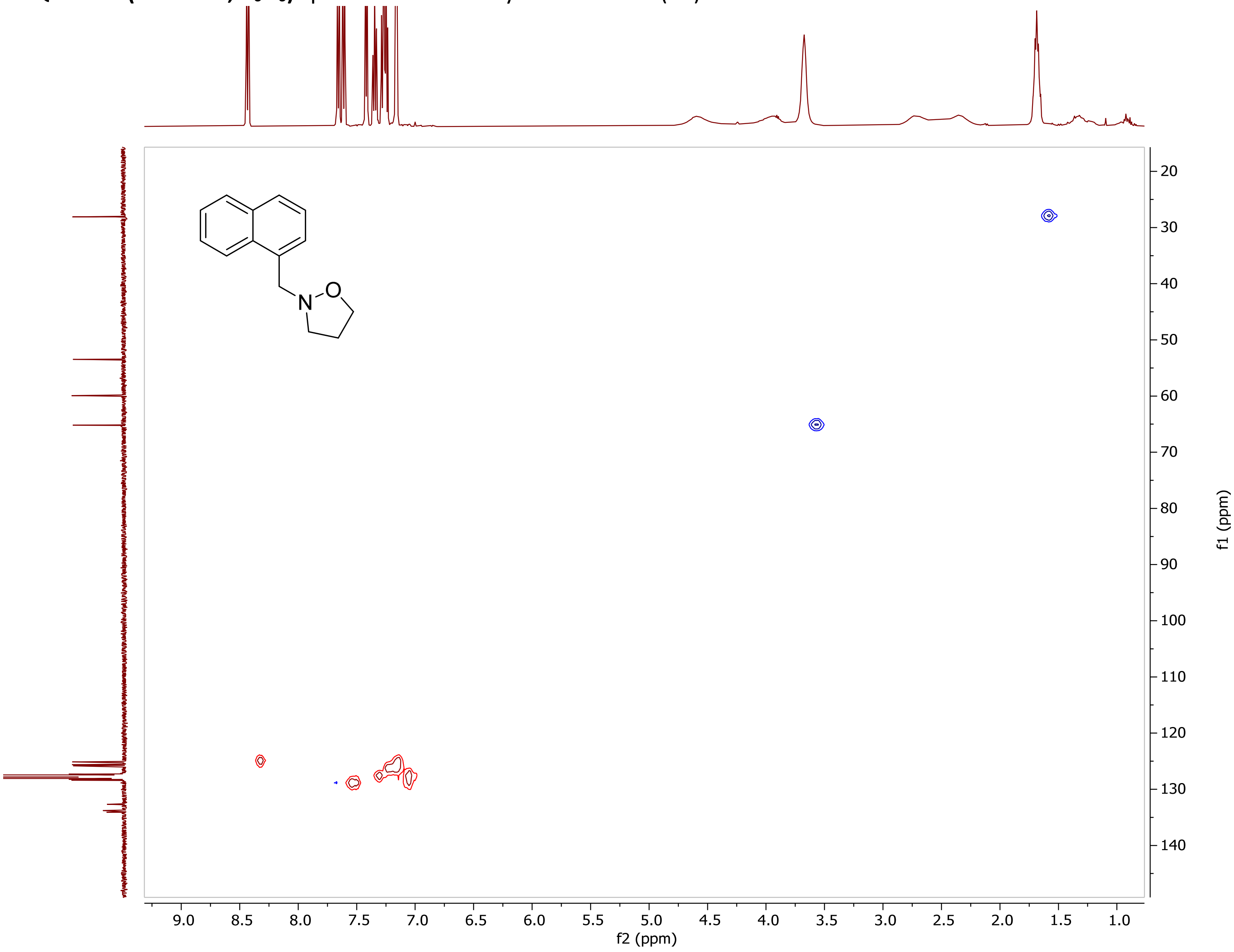

^{1}H NMR (500 MHz, $CDCl_3$) spectrum of *N*-Allyl-2-nitro-*N*-phenyl-4-(trifluoromethyl)benzenesulfonamide (**6c**)

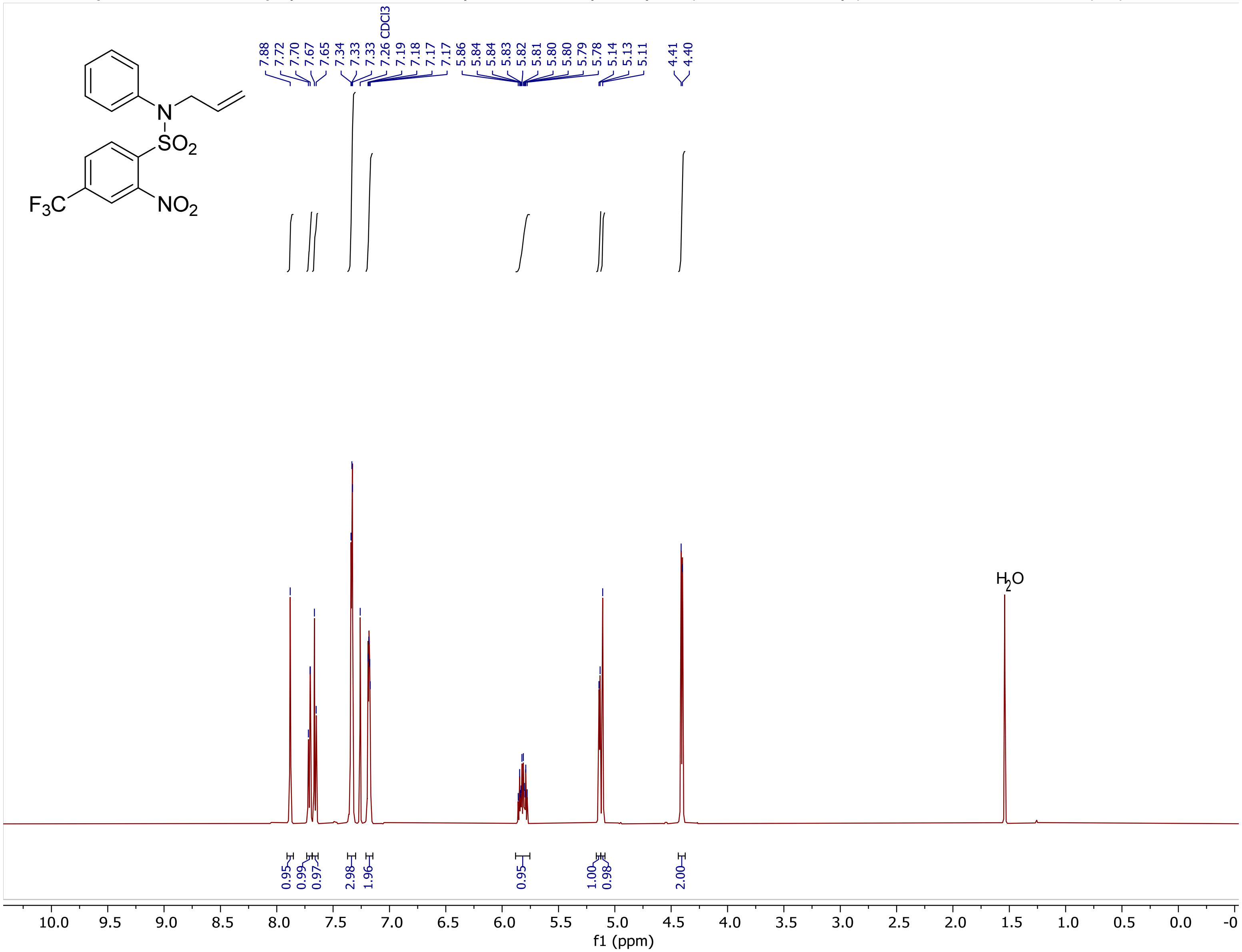

$^{13}C\ \{^{1}H\}$ NMR (126 MHz, $CDCl_3$) spectrum of *N*-Allyl-2-nitro-*N*-phenyl-4-(trifluoromethyl)benzenesulfonamide (**6c**)

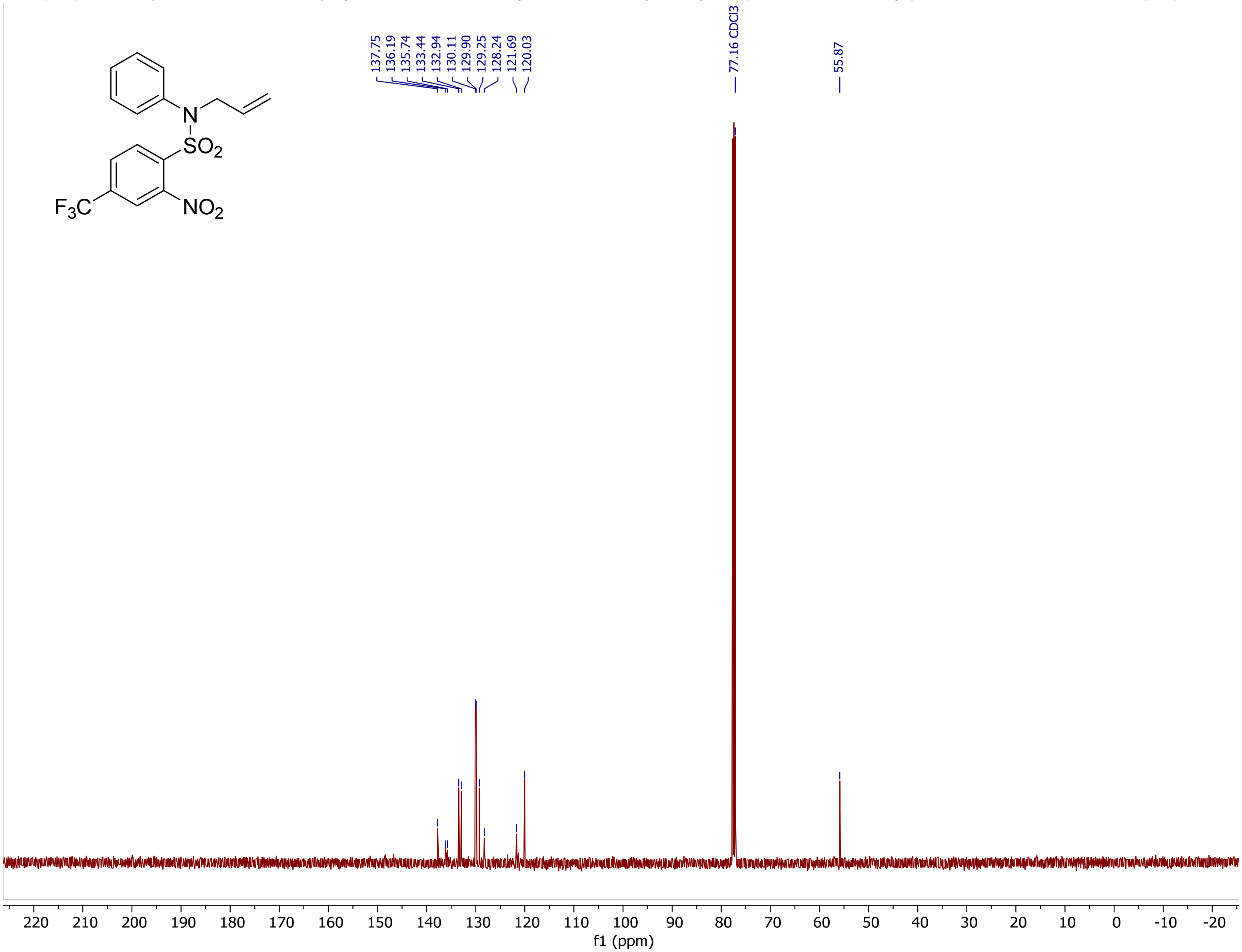

^{1}H NMR (500 MHz, $CDCl_3$) spectrum of *N*-(3-Hydroxypropyl)-2-nitro-*N*-phenyl-4-(trifluoromethyl)benzenesulfonamide (**6d**)

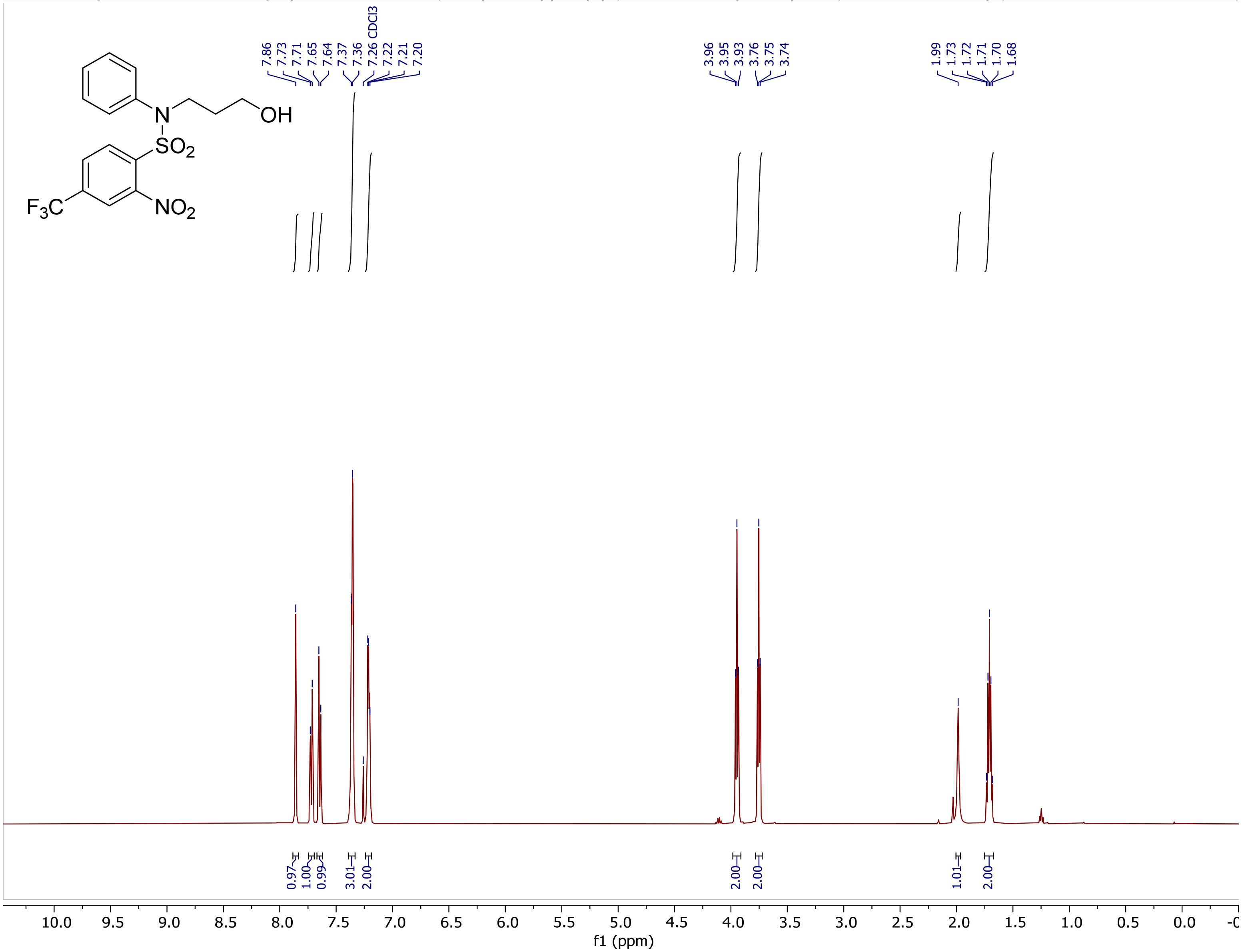

$^{13}C\ \{^1H\}$ NMR (126 MHz, $CDCl_3$) spectrum of *N*-(3-Hydroxypropyl)-2-nitro-*N*-phenyl-4-(trifluoromethyl)benzenesulfonamide (**6d**)

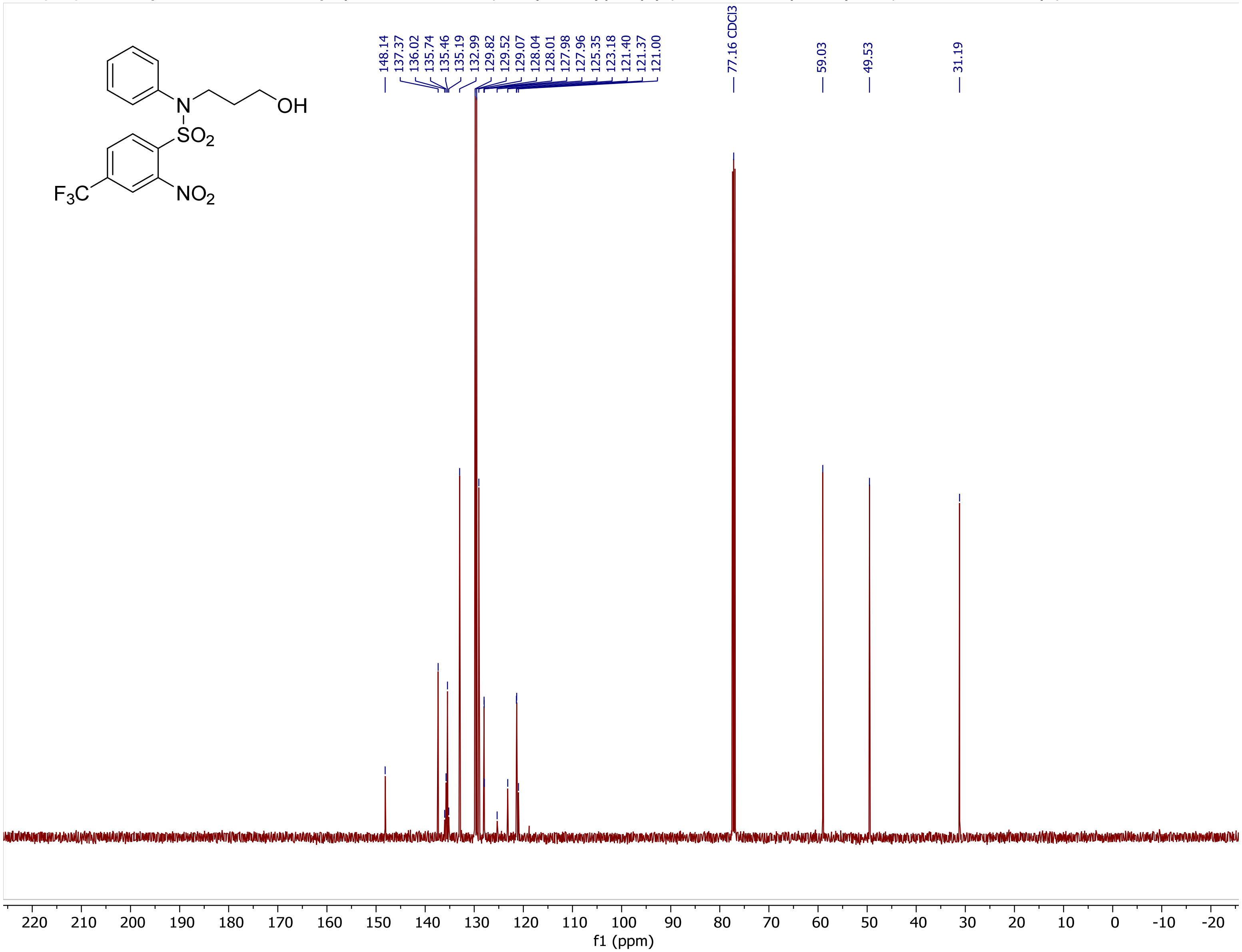

^{1}H NMR (500 MHz, $CDCl_3$) spectrum of *N*-(3-Iodopropyl)-2-nitro-*N*-phenyl-4-(trifluoromethyl)benzenesulfonamide (**6e**)

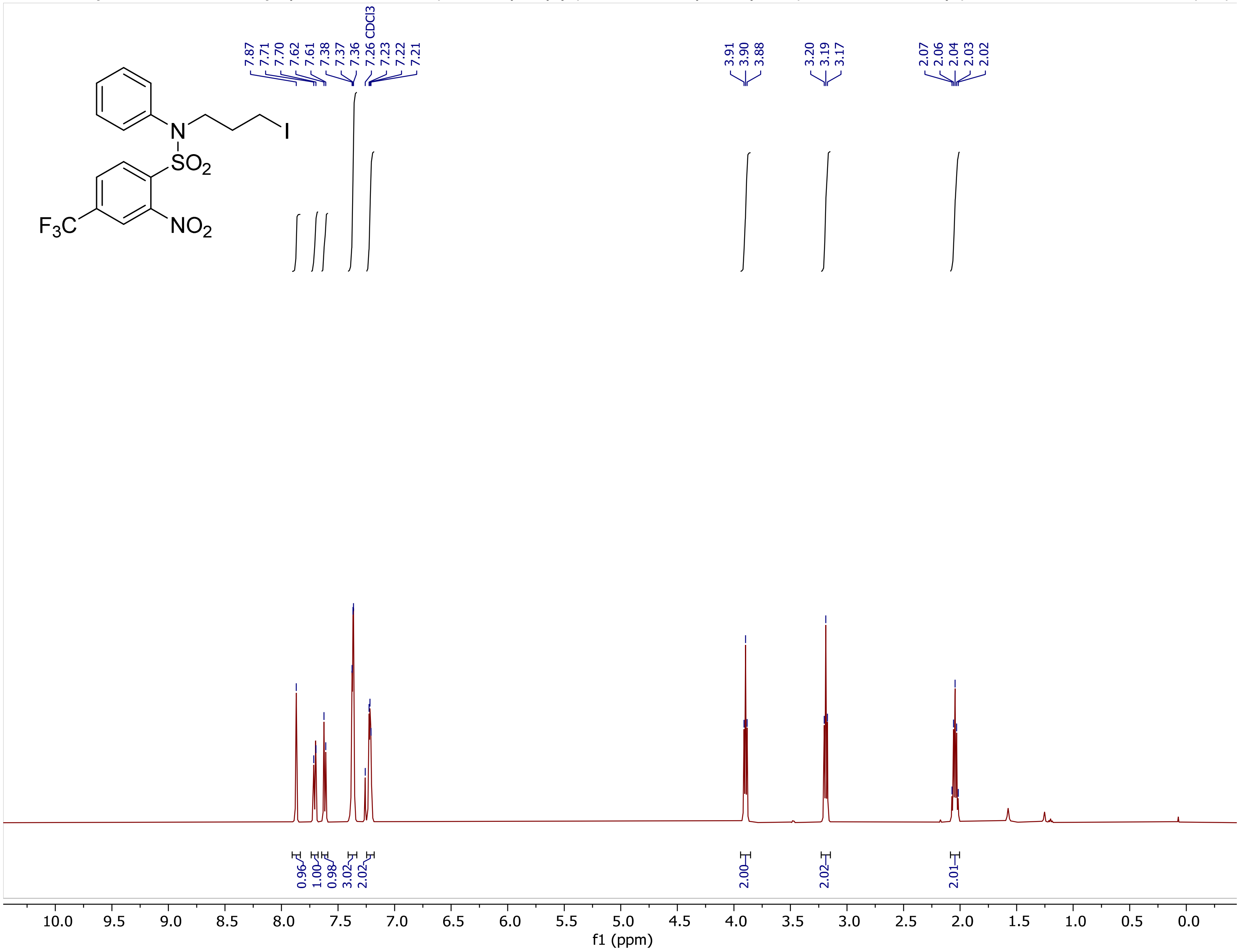

^{13}C {^{1}H} NMR (126 MHz, $CDCl_3$) spectrum of *N*-(3-Iodopropyl)-2-nitro-*N*-phenyl-4-(trifluoromethyl)benzenesulfonamide (**6e**)

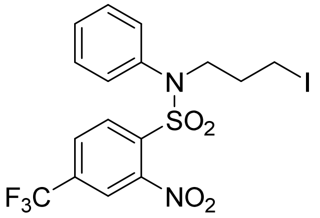

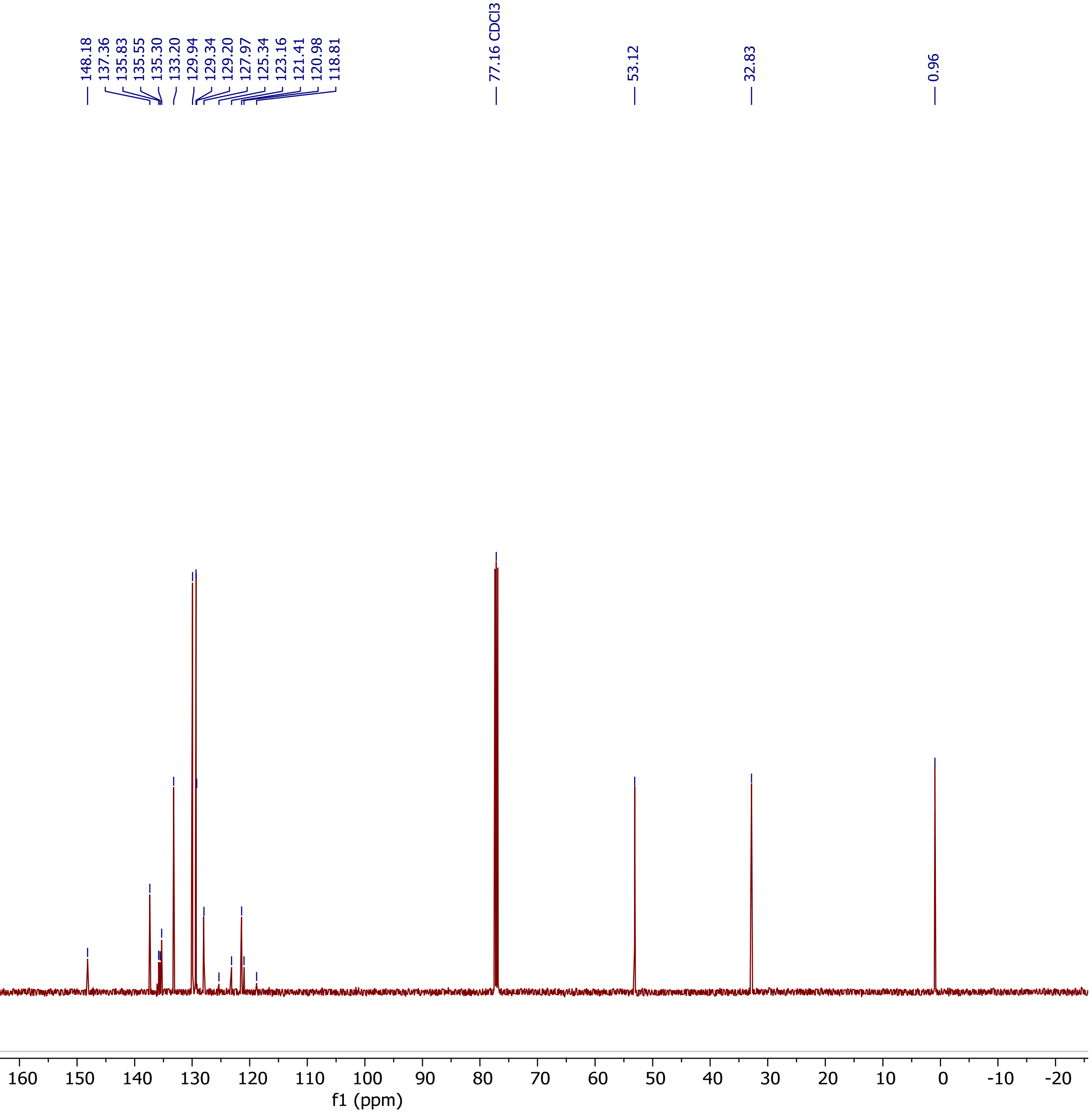

HSQC NMR (500 MHz, $CDCl_3$) spectrum of *N*-(3-Iodopropyl)-2-nitro-*N*-phenyl-4-(trifluoromethyl)benzenesulfonamide (**6e**)

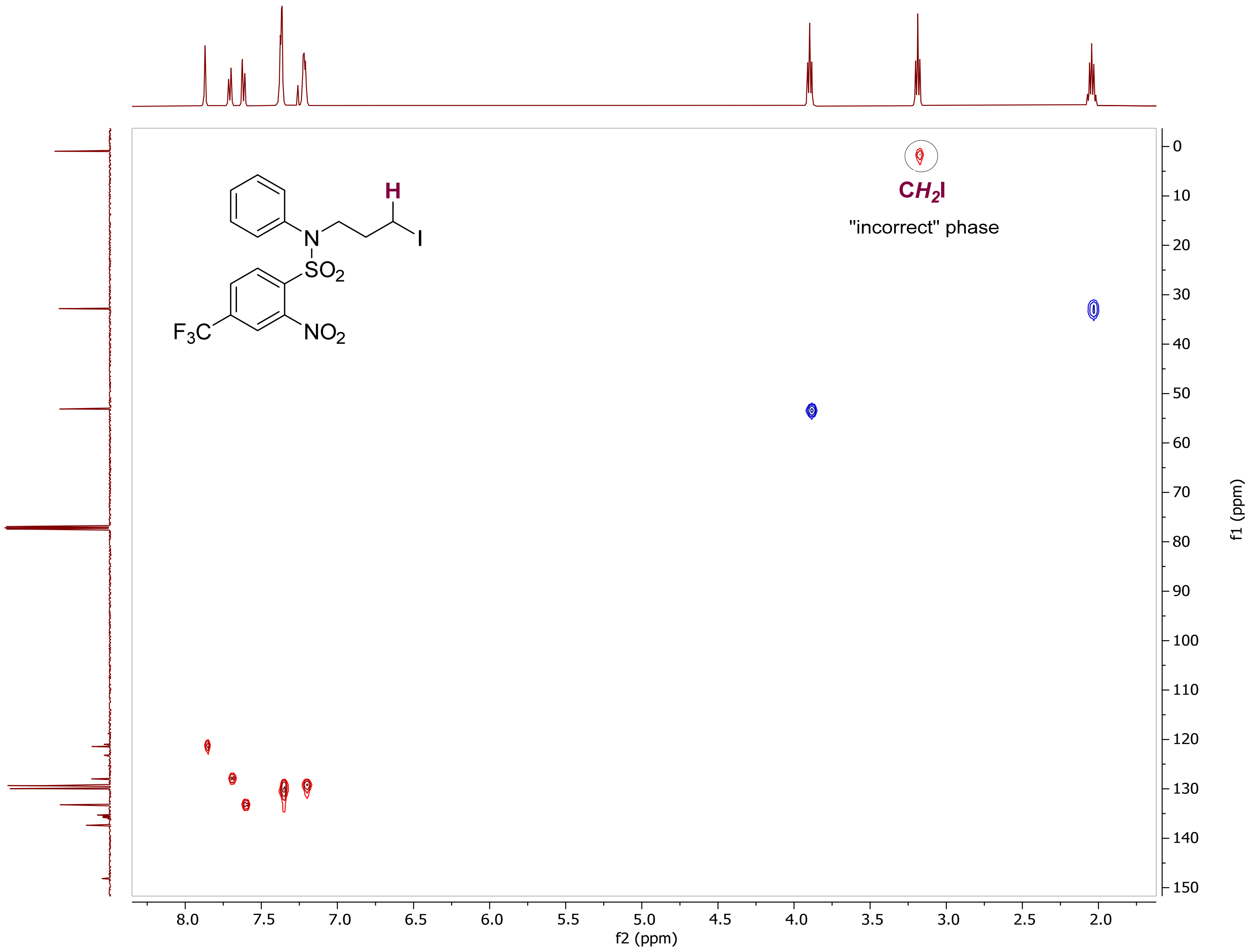

DEPT 135 and $^{13}C\{^1H\}$ stacked NMR (126 MHz, $CDCl_3$) spectrum of *N*-(3-Iodopropyl)-2-nitro-*N*-phenyl-4-(trifluoromethyl)benzenesulfonamide (**6e**)

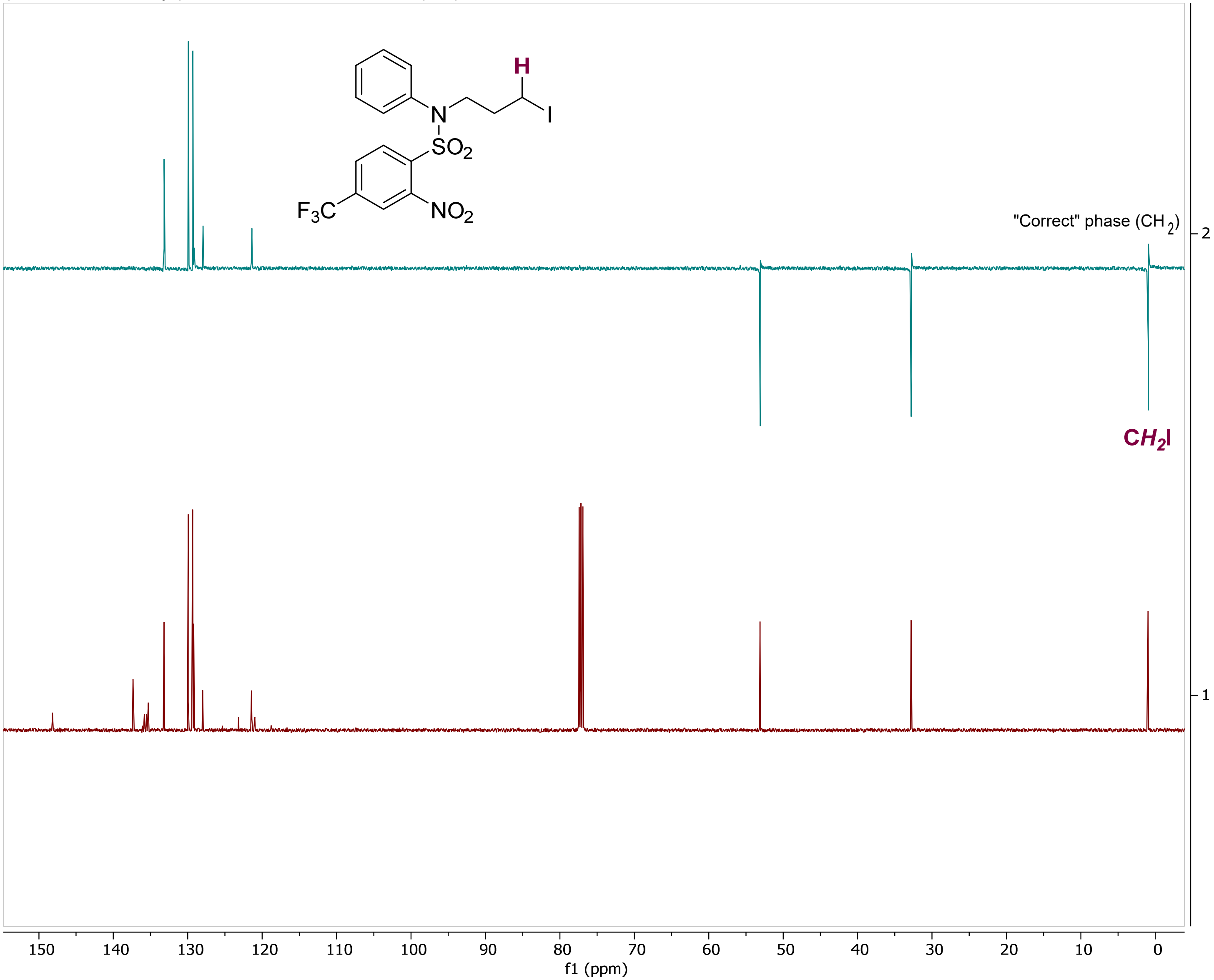

^{1}H NMR (500 MHz, $CDCl_3$) spectrum of *N*-(3-Hydroperoxypropyl)-2-nitro-*N*-phenyl-4-(trifluoromethyl)benzenesulfonamide (**6f**)

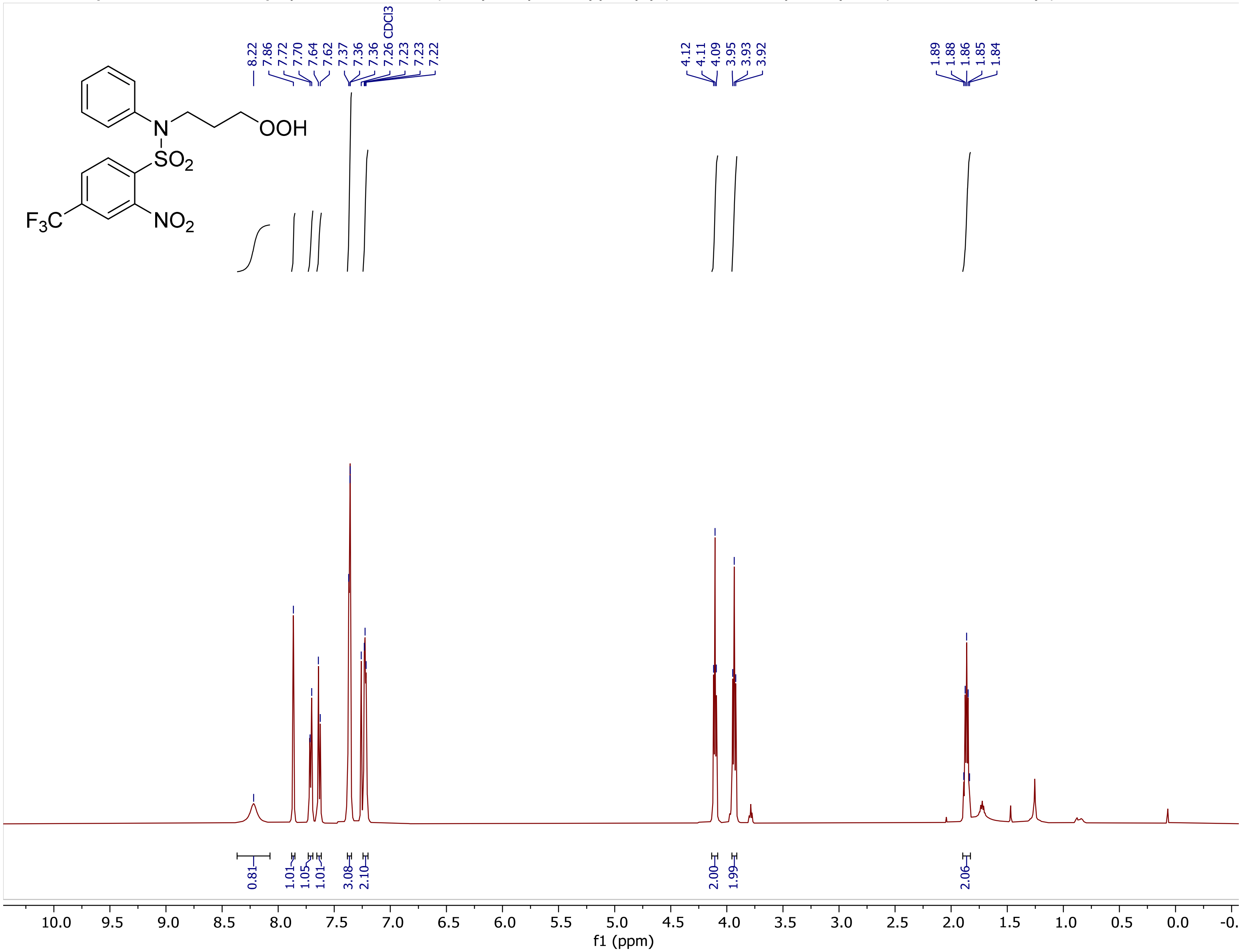

^{13}C {^{1}H} NMR (126 MHz, $CDCl_3$) spectrum of *N*-(3-Hydroperoxypropyl)-2-nitro-*N*-phenyl-4-(trifluoromethyl)benzenesulfonamide (**6f**)

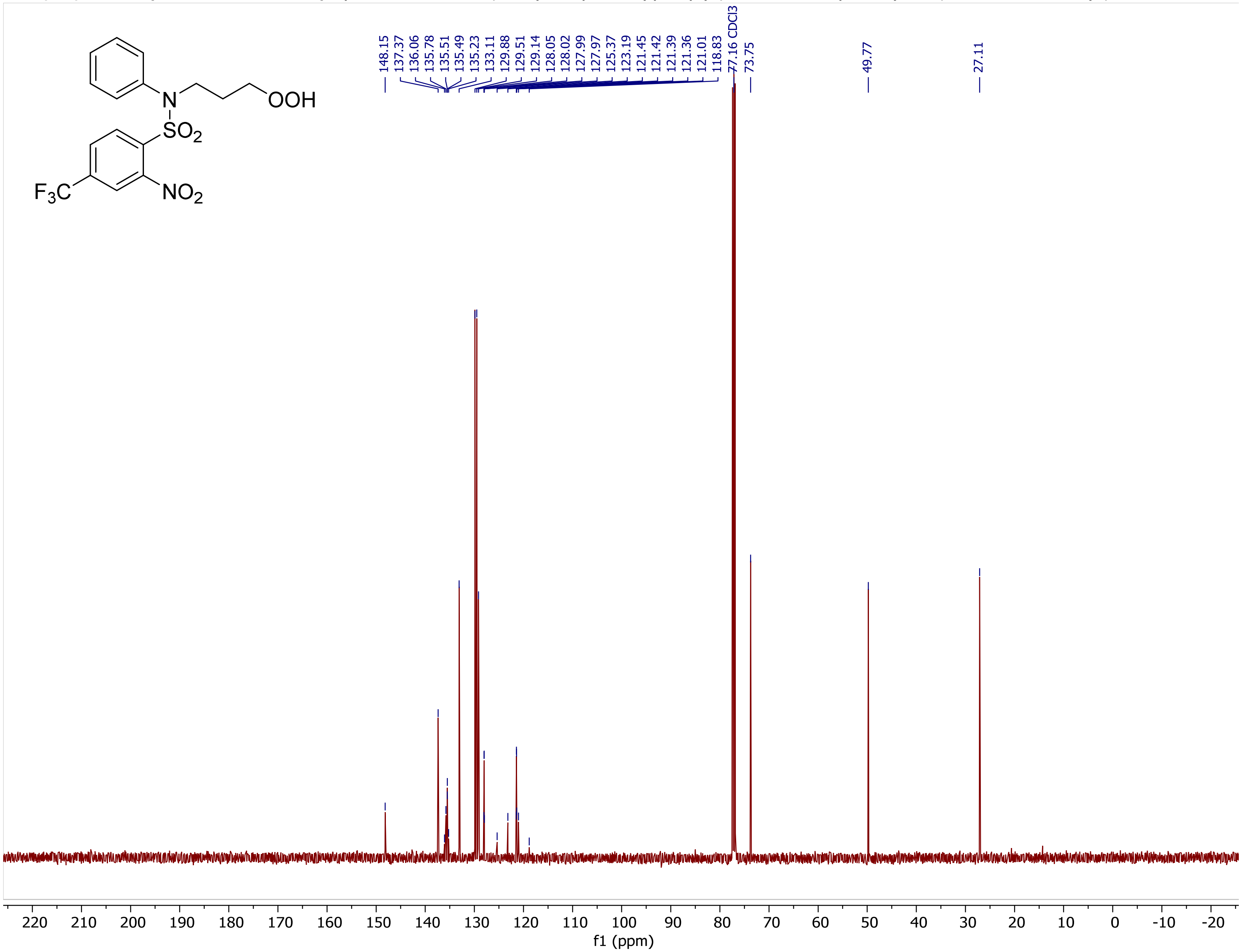

^{1}H NMR (500 MHz, C_6D_6) spectrum of *N*-(3-((*tert*-Butyldiphenylsilyl)peroxy)propyl)-2-nitro-*N*-4-(trifluoromethyl)benzenesulfonamide (**44**)

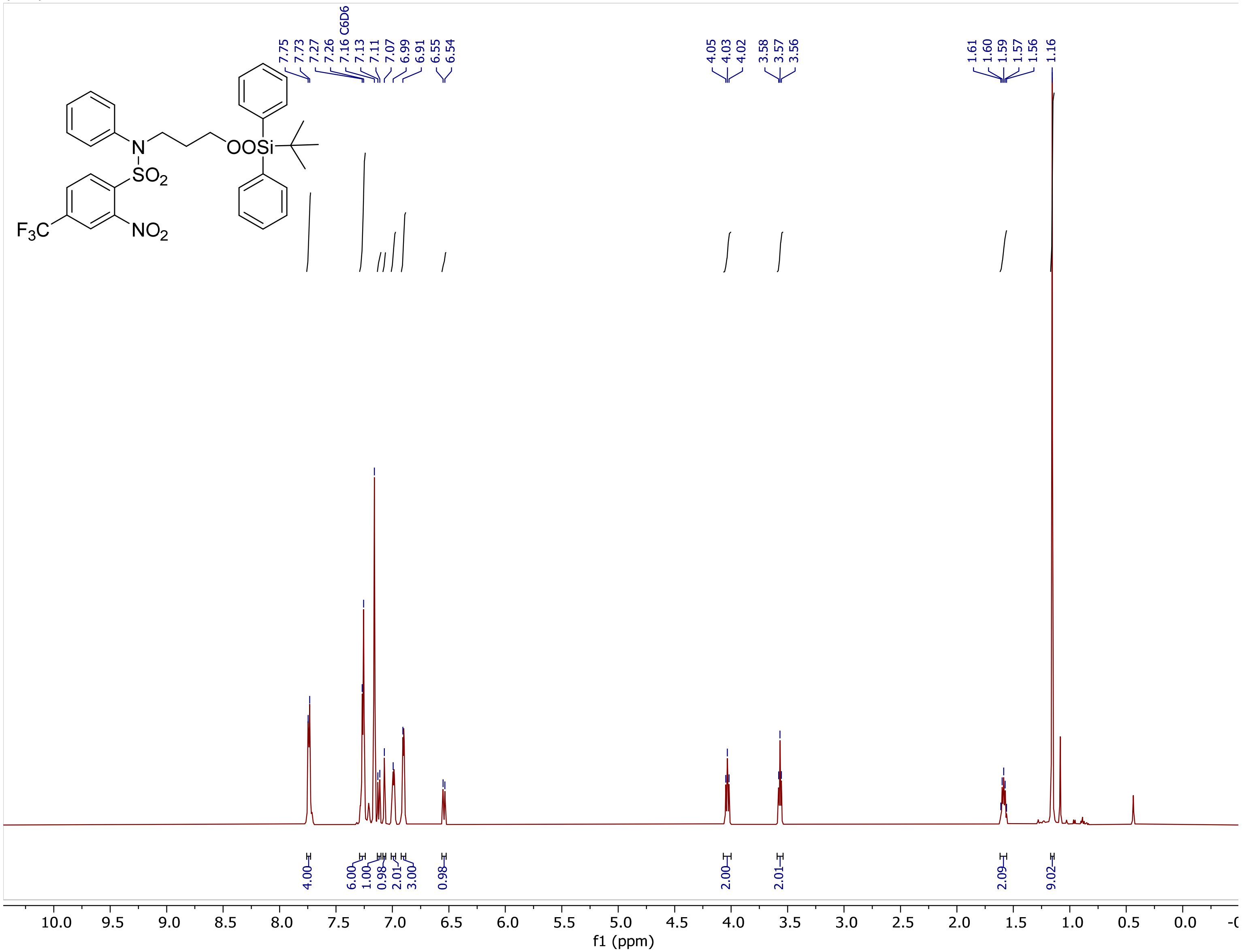

^{13}C {^{1}H} NMR (126 MHz, C_6D_6) spectrum of *N*-(3-((*tert*-Butyldiphenylsilyl)peroxy)propyl)-2-nitro-*N*-4-(trifluoromethyl)benzenesulfonamide (**44**)

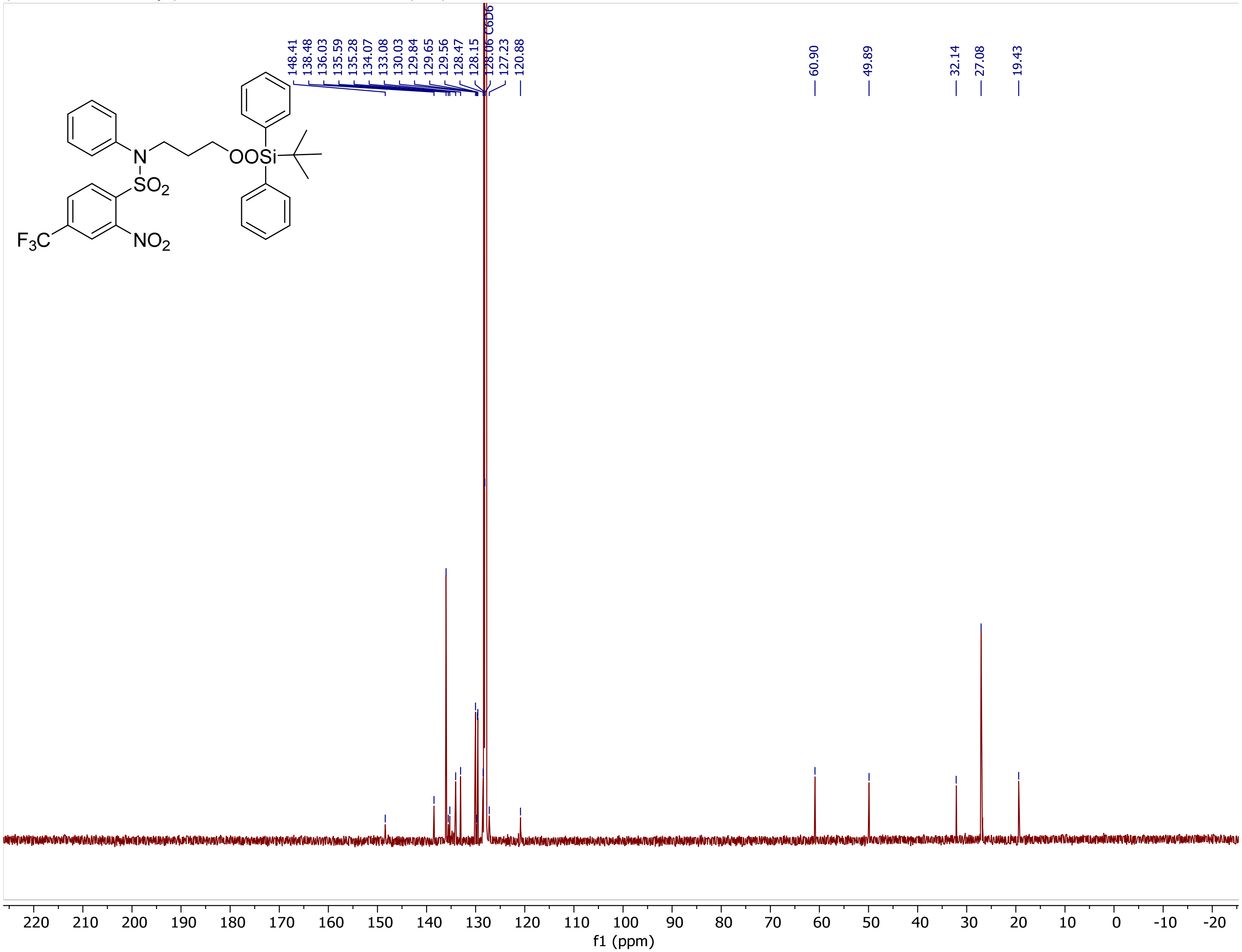

^{1}H NMR (500 MHz, $CDCl_3$) spectrum of 2-Phenylisoxazolidine (**45**)

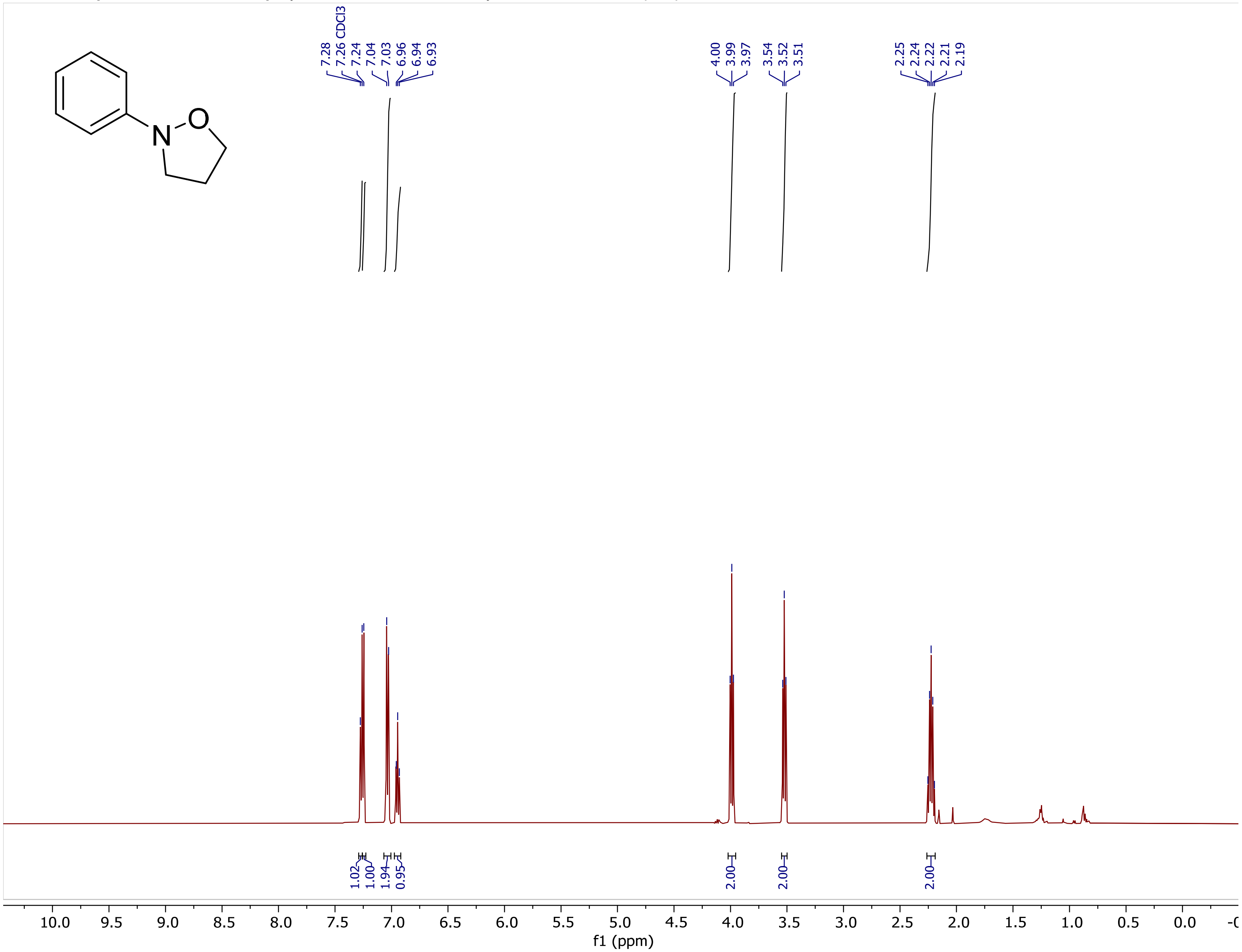

$^{13}C\{^1H\}$ **NMR (126 MHz, $CDCl_3$)** spectrum of 2-Phenylisoxazolidine (**45**)

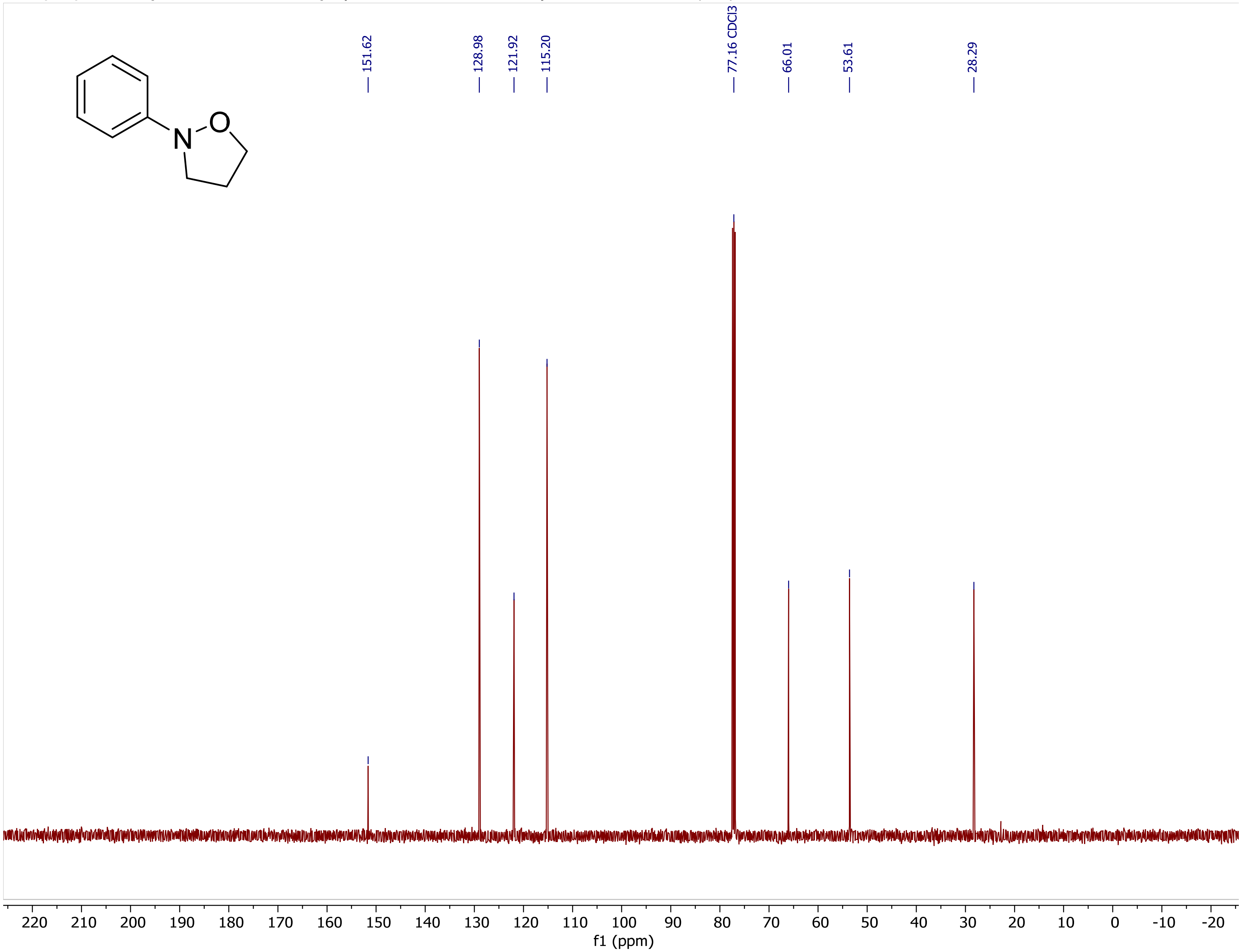

HSQC NMR (500 MHz, $CDCl_3$) spectrum of 2-Phenylisoxazolidine (**45**)

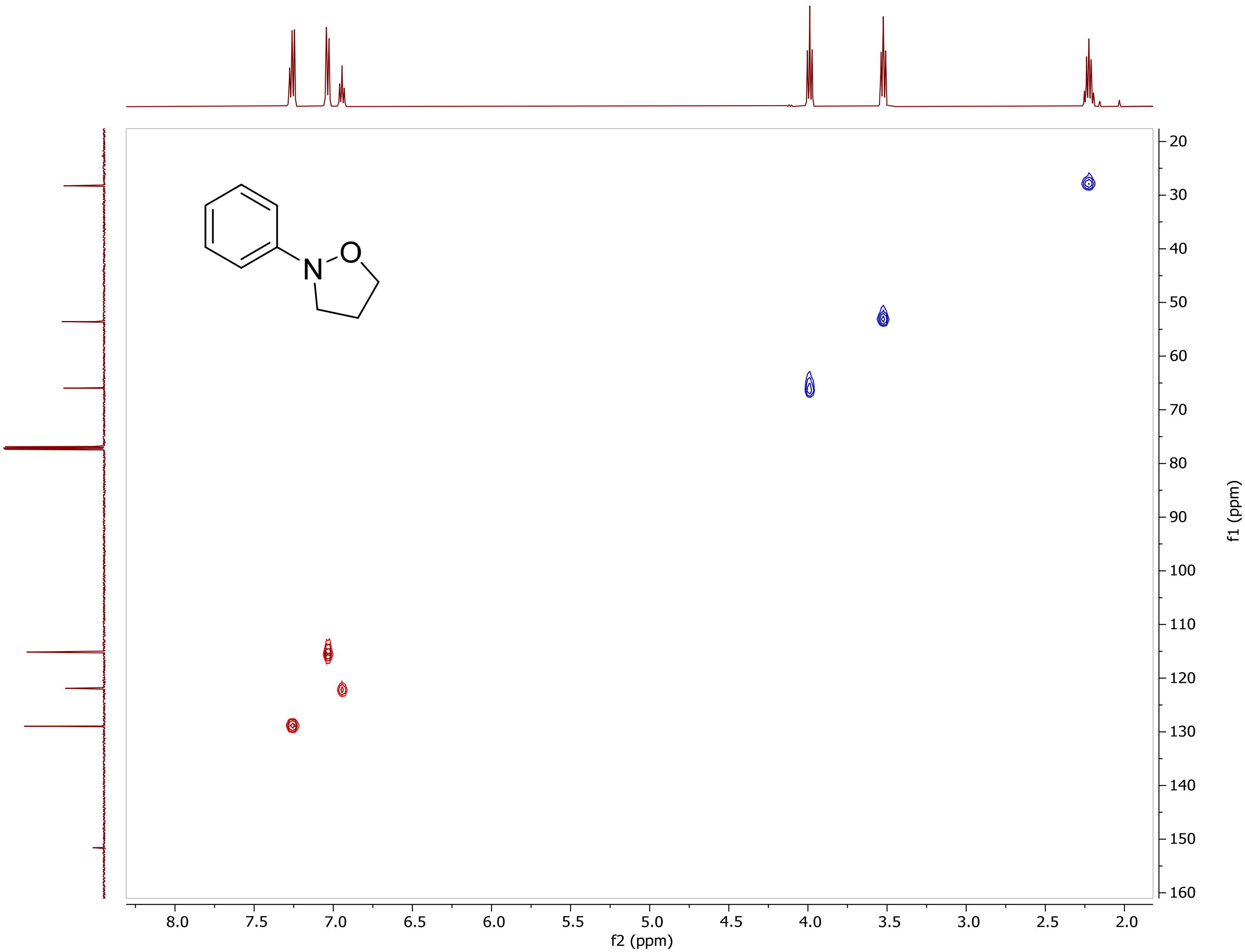

^{1}H NMR (500 MHz, CDCl$_3$) spectrum of *N*-(3-Methylbut-3-en-1-yl)-*N*-(naphthalen-1-ylmethyl)-2-nitro-4-(trifluoromethyl)benzenesulfonamide (**3i**)

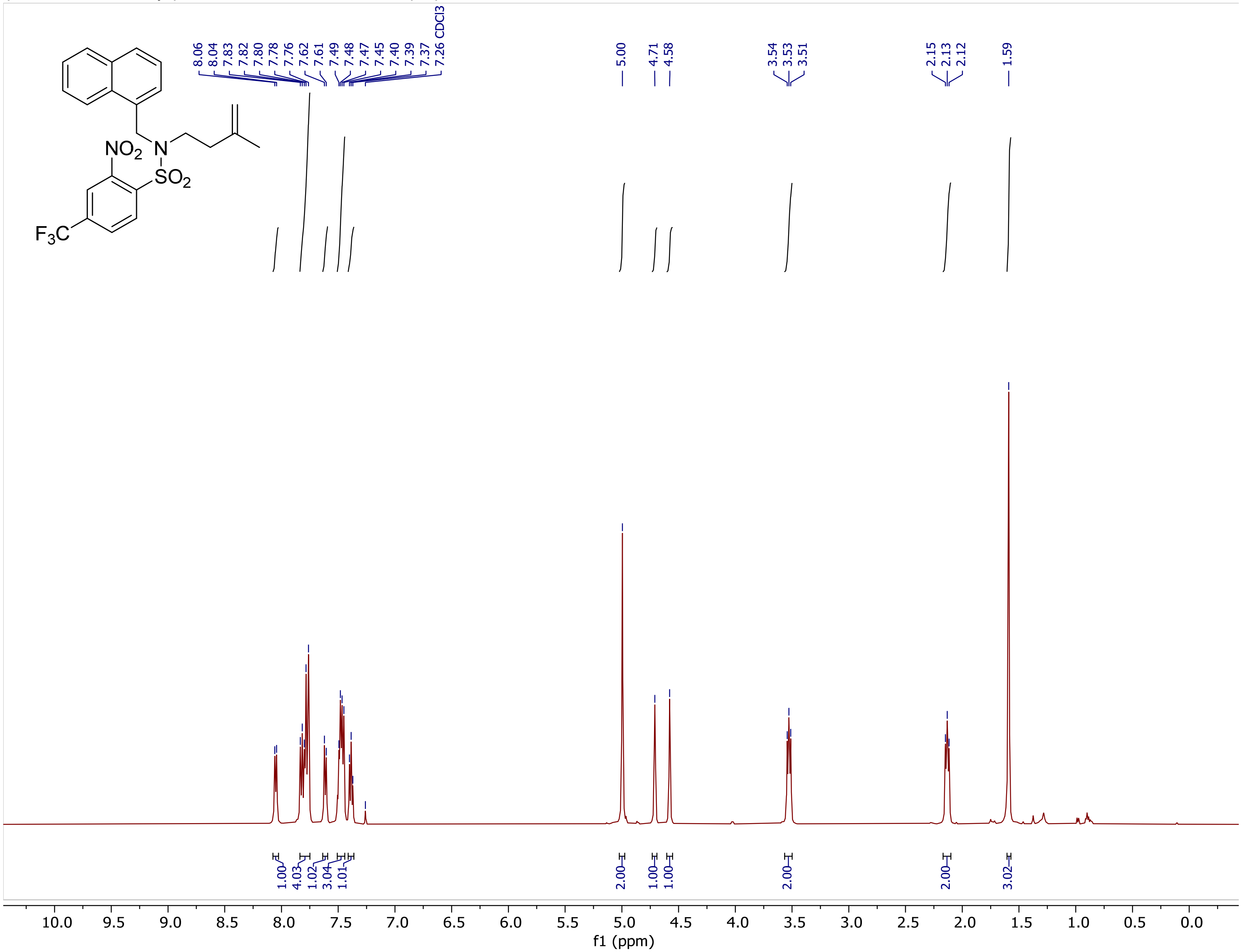

$^{13}C\ \{^1H\}$ NMR (126 MHz, $CDCl_3$) spectrum of *N*-(3-Methylbut-3-en-1-yl)-*N*-(naphthalen-1-ylmethyl)-2-nitro-4-(trifluoromethyl)benzenesulfonamide (**3i**)

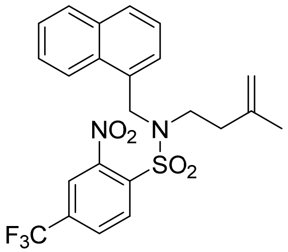

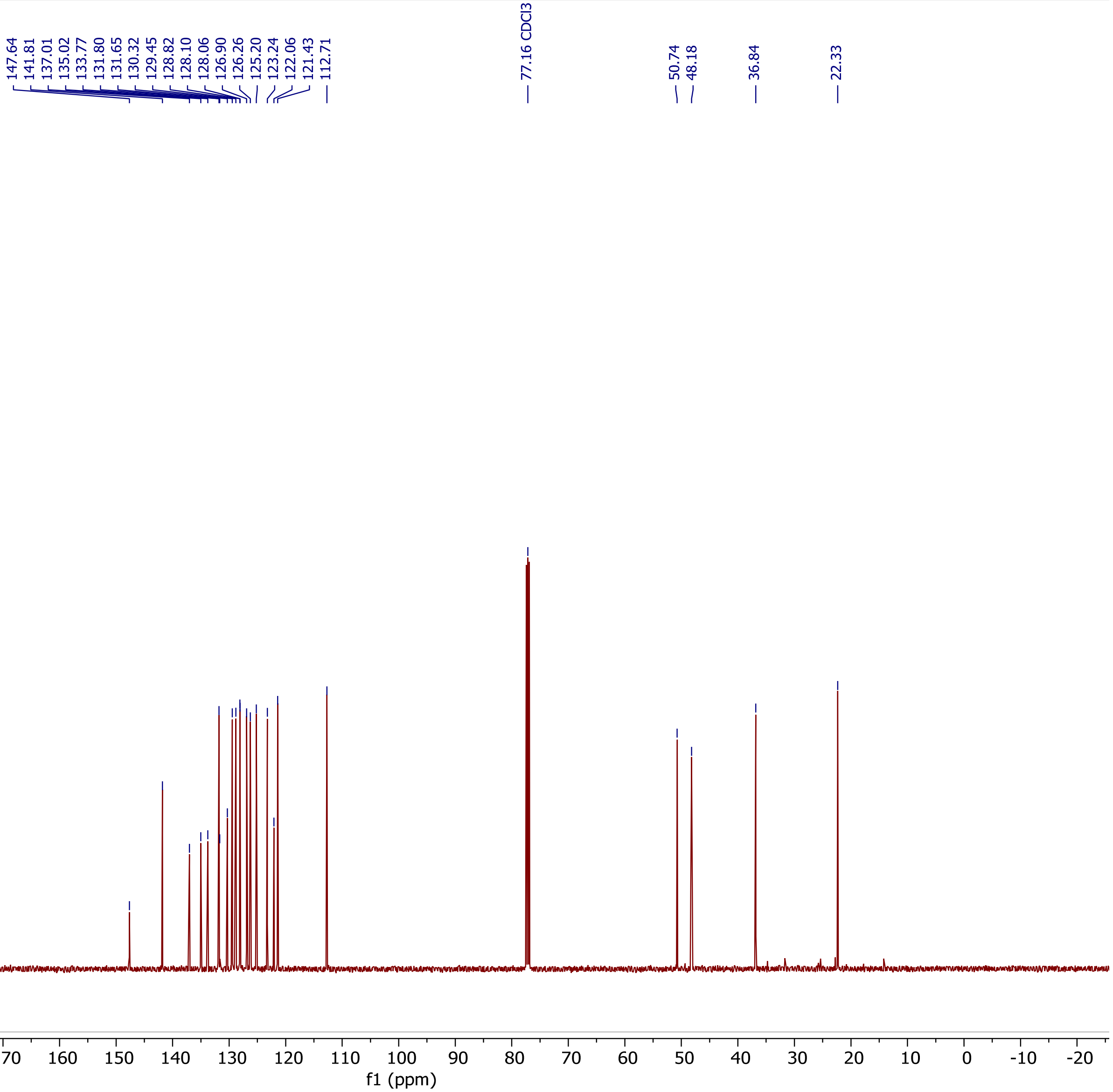

^{1}H NMR (500 MHz, $CDCl_3$) spectrum of *N*-(3-Methyl-3-((triethylsilyl)peroxy)butyl)-*N*-(naphthalen-1-ylmethyl)-2-nitro-4-(trifluoromethyl)benzenesulfonamide (**3j**)

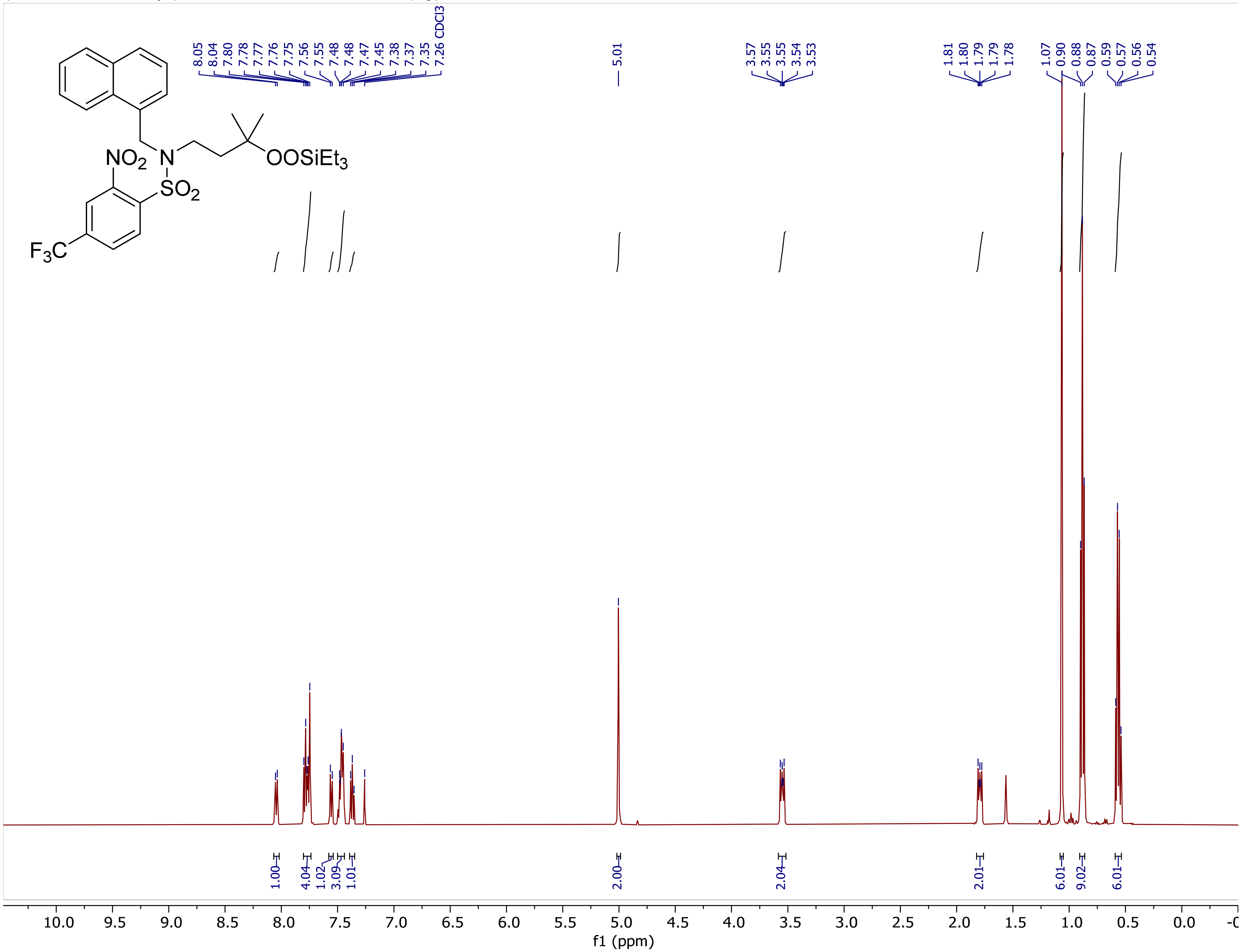

^{13}C {^{1}H} NMR (126 MHz, CDCl$_3$) spectrum of *N*-(3-Methyl-3-((triethylsilyl)peroxy)butyl)-*N*-(naphthalen-1-ylmethyl)-2-nitro-4-(trifluoromethyl)benzenesulfonamide (**3j**)

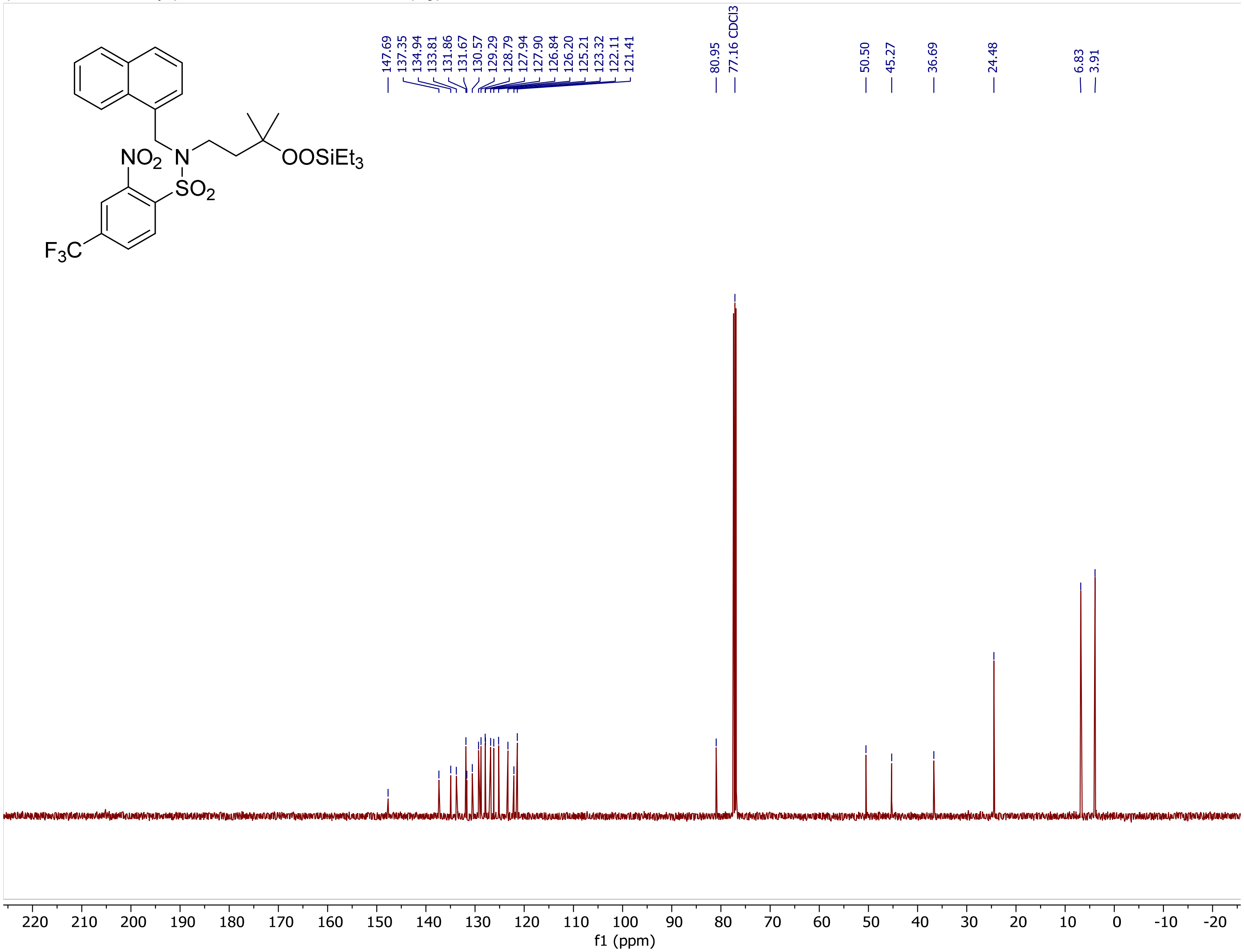

^{1}H NMR (500 MHz, C_6D_6) spectrum of *N*-(3-((*tert*-Butyldiphenylsilyl)peroxy)-3-methylbutyl)-*N*-(naphthalen-1-ylmethyl)-2-nitro-4-(trifluoromethyl)benzenesulfonamide (**46**)

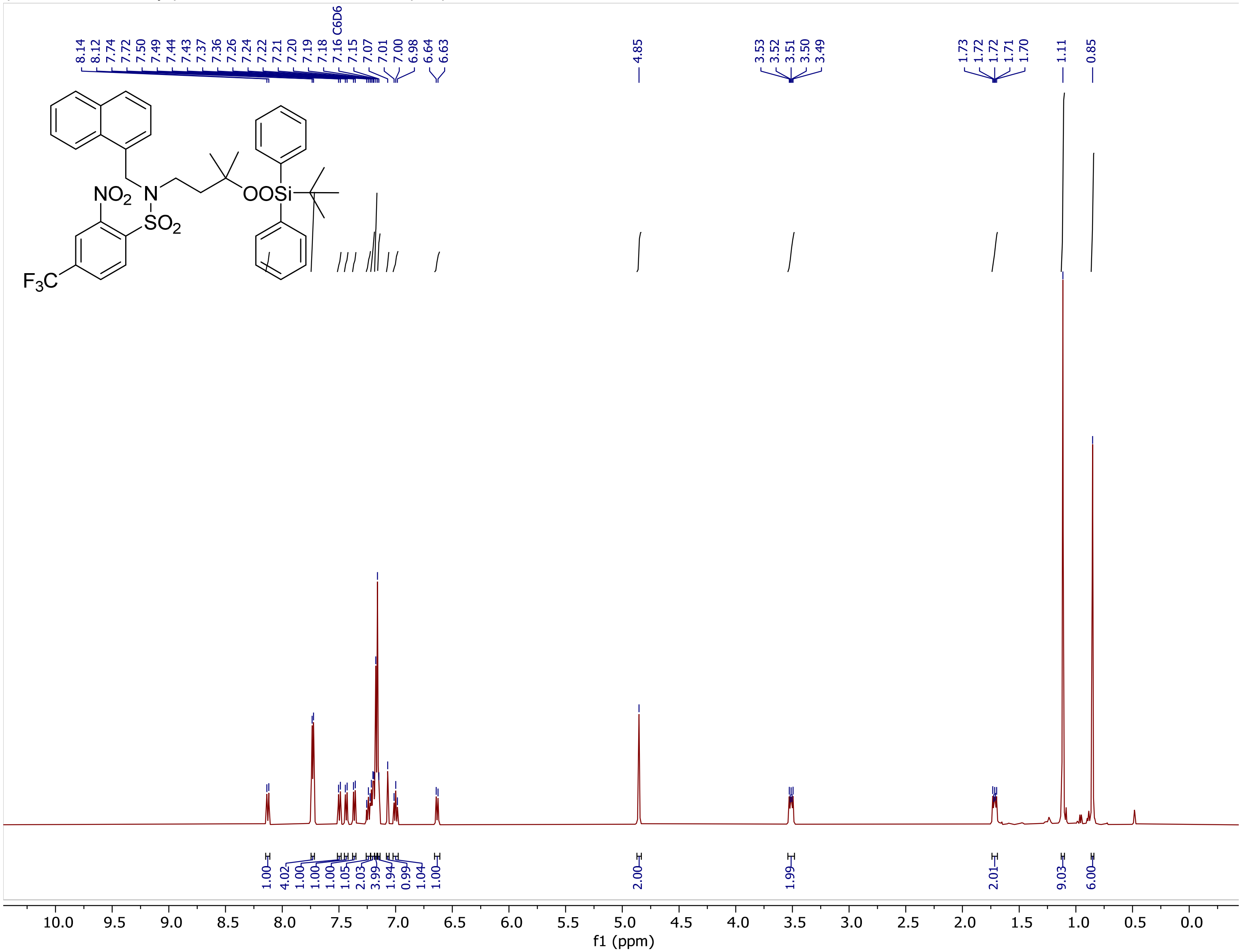

$^{13}C\ \{^{1}H\}$ NMR (126 MHz, C_6D_6) spectrum of *N*-(3-((*tert*-Butyldiphenylsilyl)peroxy)-3-methylbutyl)-*N*-(naphthalen-1-ylmethyl)-2-nitro-4-(trifluoromethyl)benzenesulfonamide (**46**)

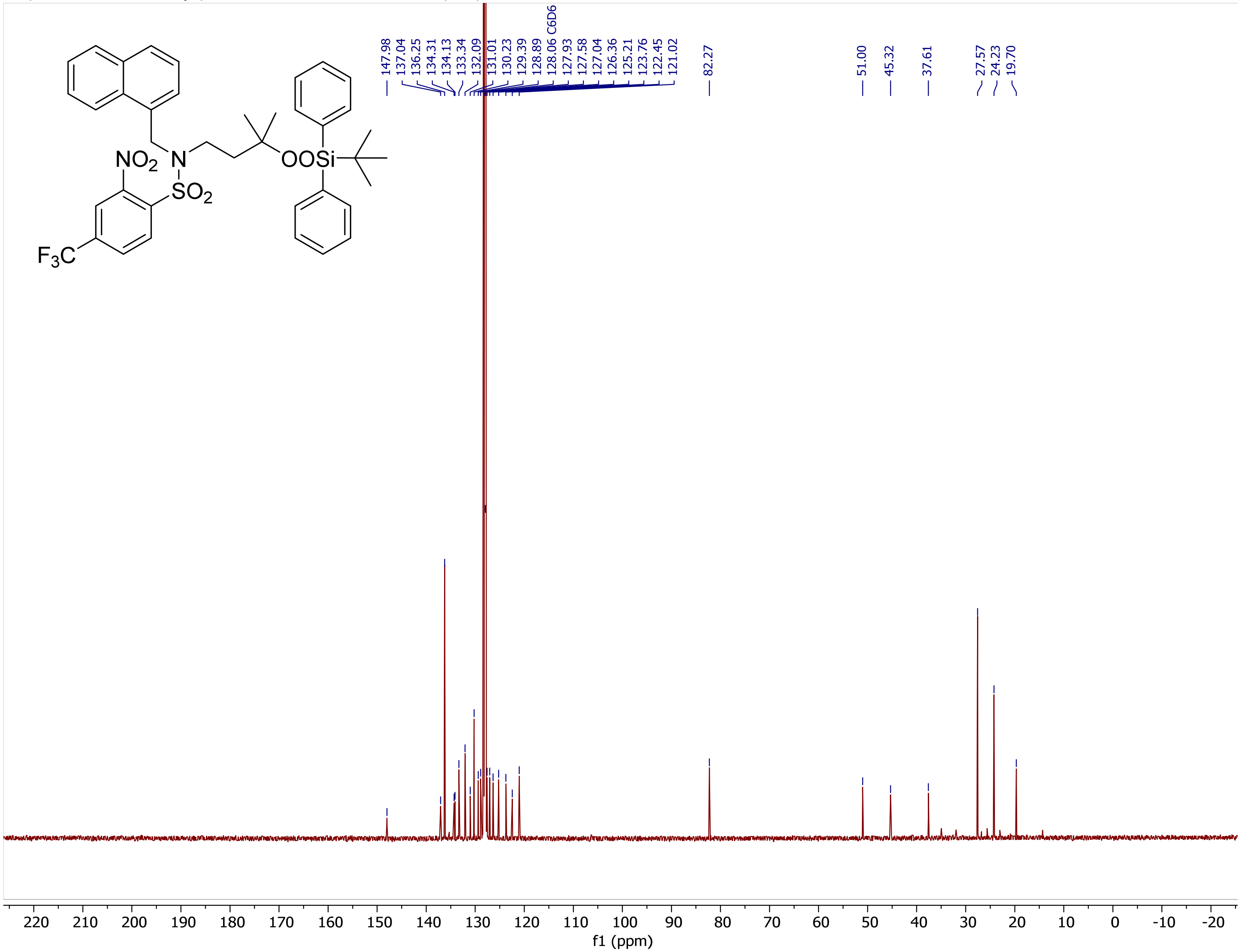

^{1}H NMR (500 MHz, C_6D_6) spectrum of (3-((*tert*-Butyldiphenylsilyl)peroxy)-3-methy-*N*-(naphthalen-1-ylmethyl)butan-1-amine (**3k**)

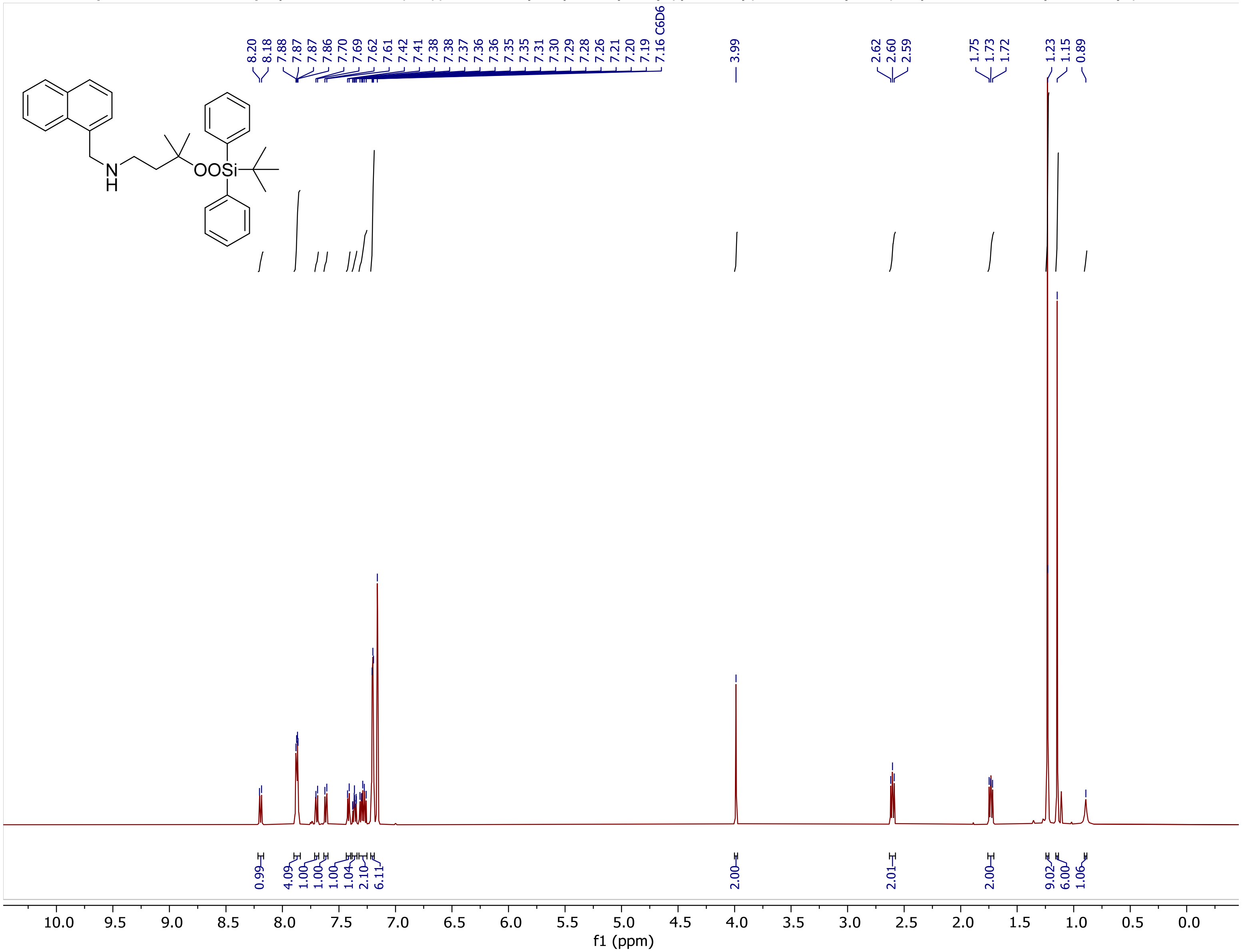

$^{13}C\{^1H\}$ NMR (126 MHz, C_6D_6) spectrum of (3-((*tert*-Butyldiphenylsilyl)peroxy)-3-methy-*N*-(naphthalen-1-ylmethyl)butan-1-amine (**3k**)

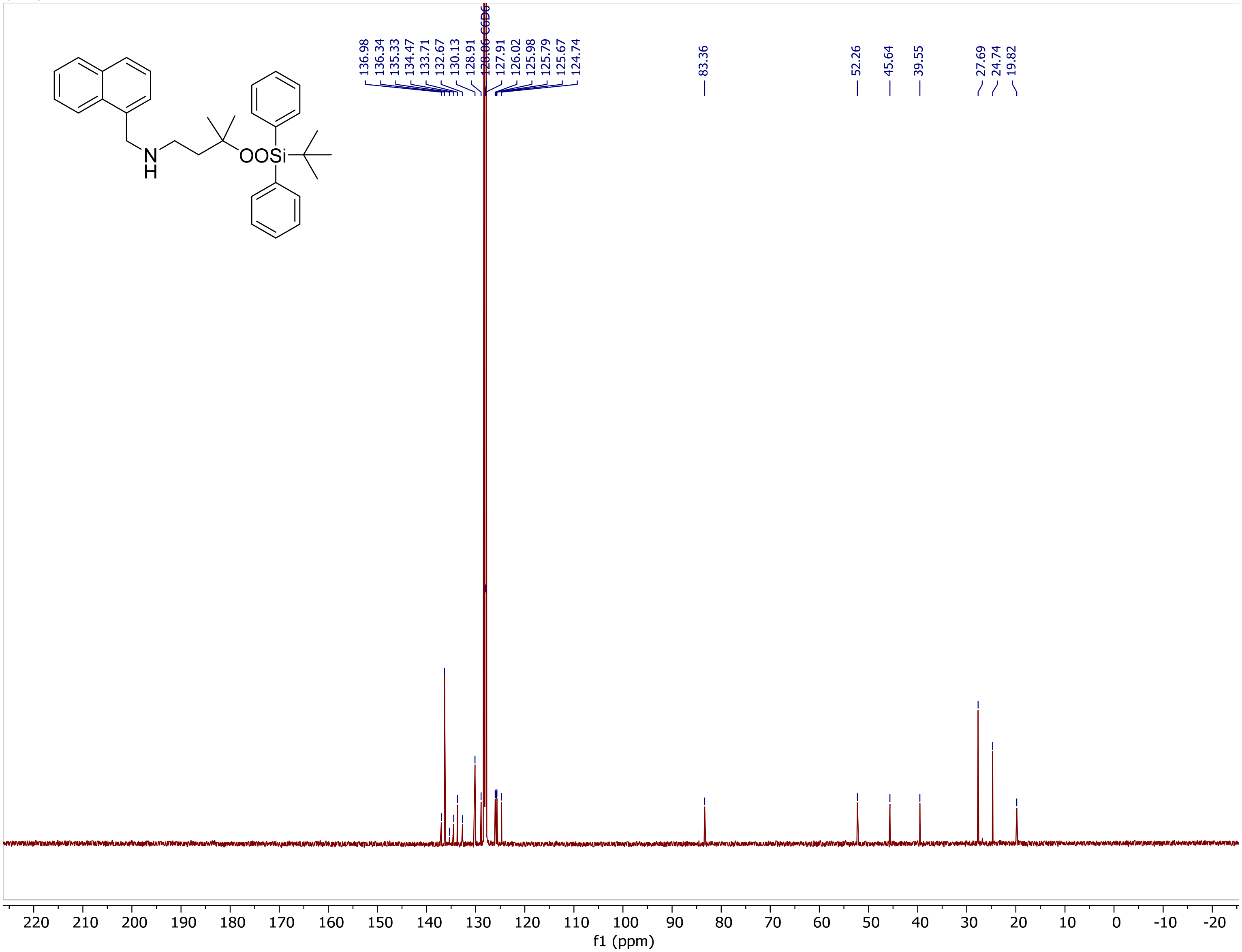

^{1}H NMR (500 MHz, C_6D_6) spectrum of 5,5-Dimethyl-2-(naphthalen-1-ylmethyl)isoxazolidine (**25**)

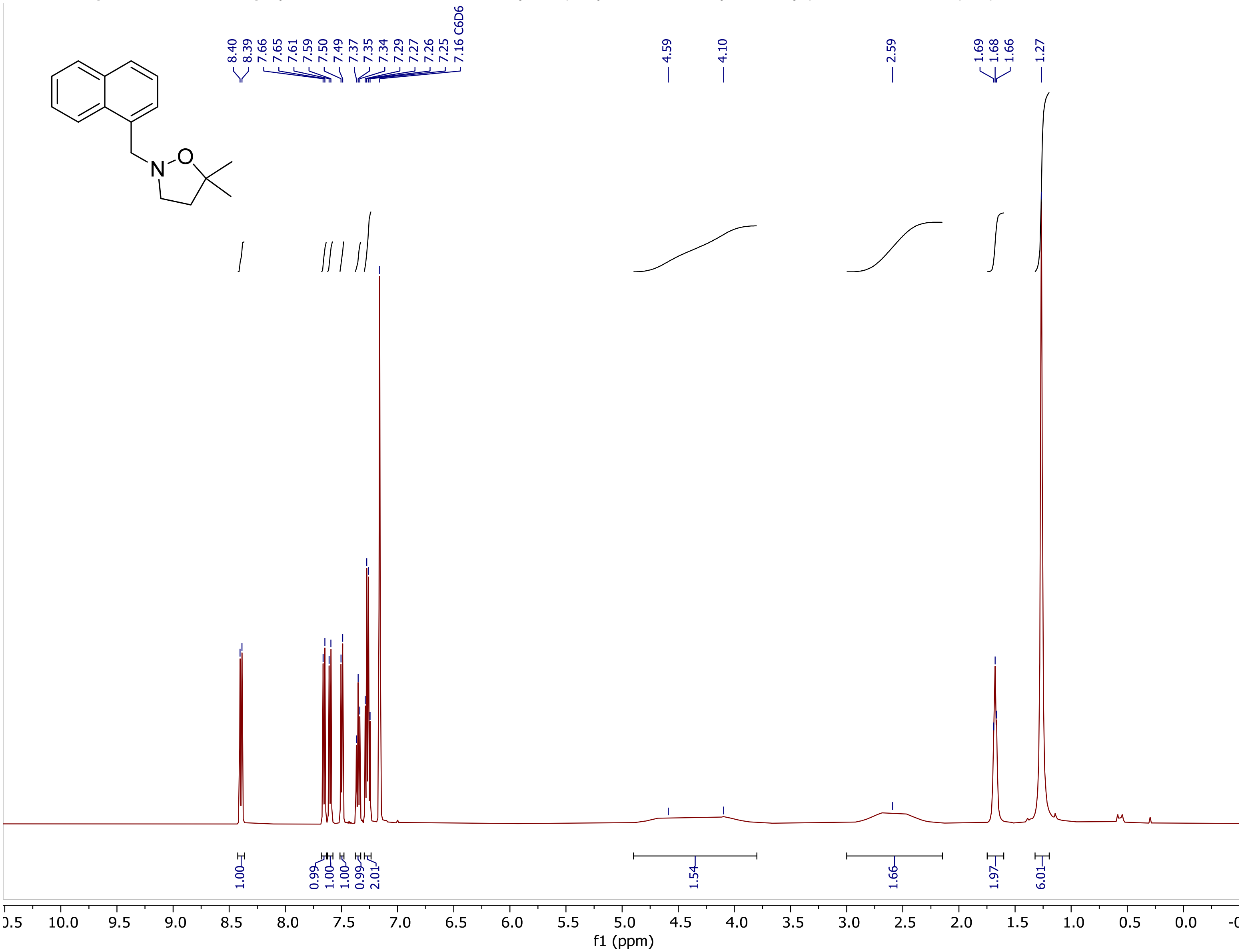

^{1}H NMR (500 MHz, C_6D_6) spectrum of 5,5-Dimethyl-2-(naphthalen-1-ylmethyl)isoxazolidine at 65 °C (**25**)

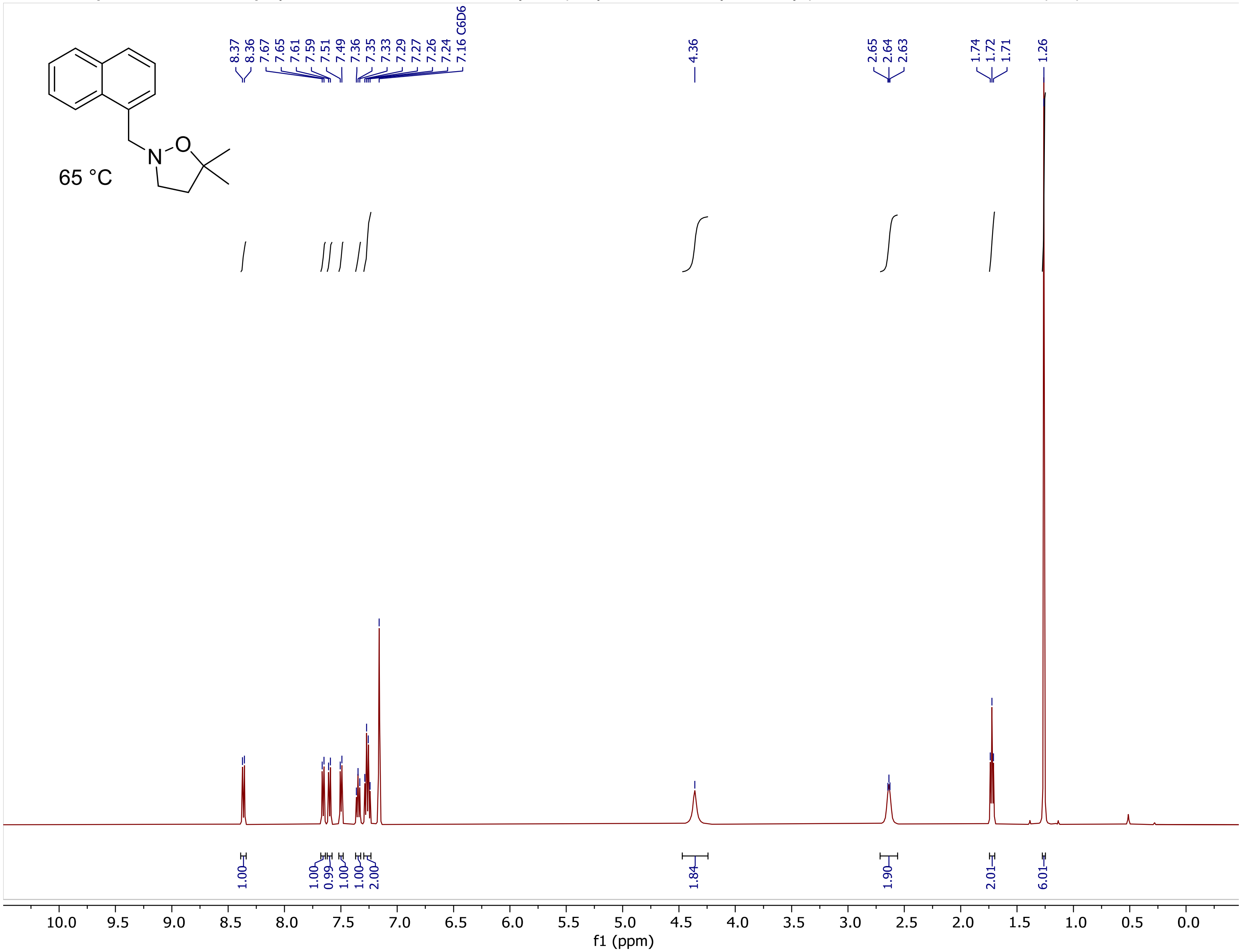

^{13}C {^{1}H} NMR (126 MHz, C_6D_6) spectrum of 5,5-Dimethyl-2-(naphthalen-1-ylmethyl)isoxazolidine at 65 °C (**25**)

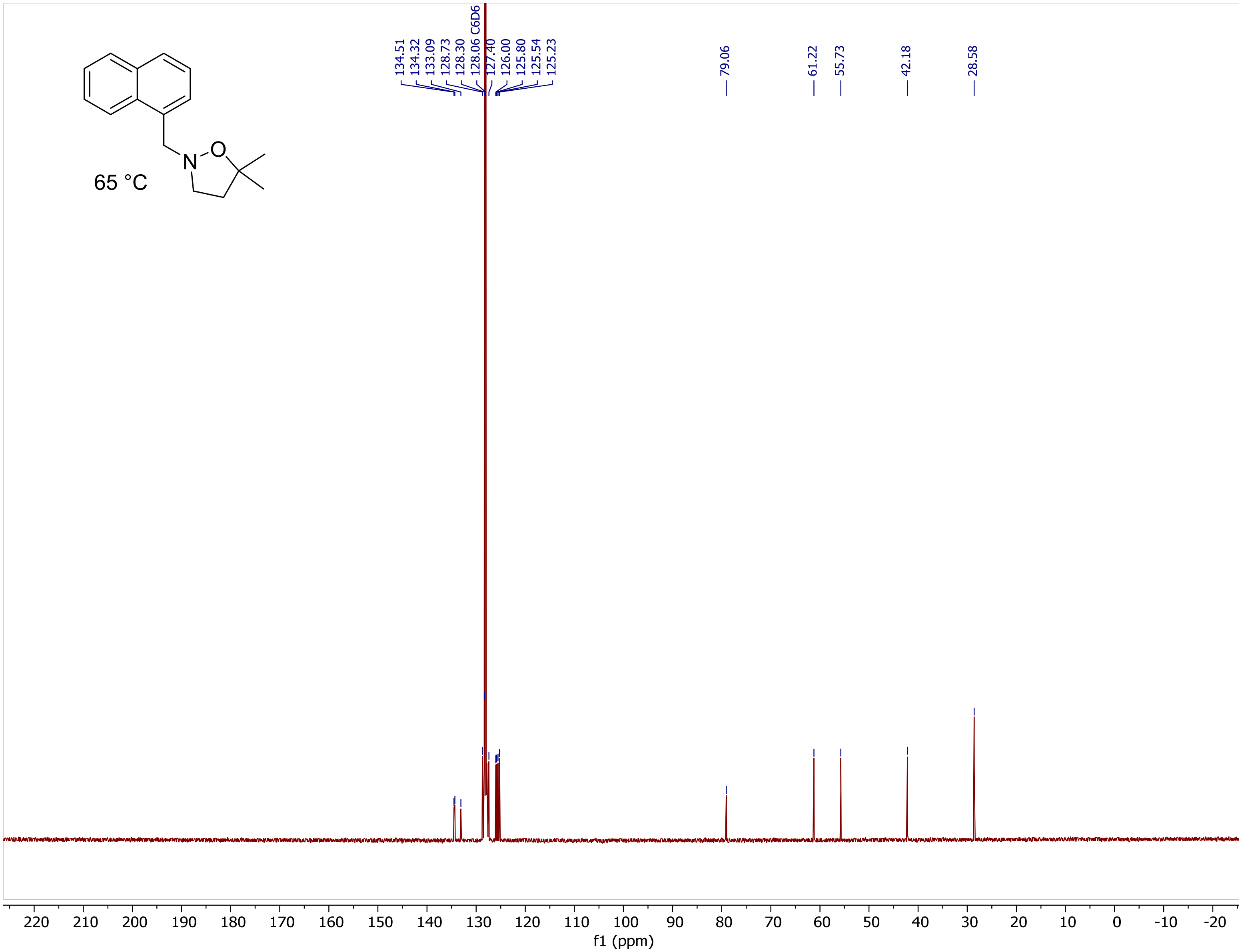

HSQC NMR (500 MHz, C_6D_6) spectrum of 5,5-Dimethyl-2-(naphthalen-1-ylmethyl)isoxazolidine at 65 °C (**25**)

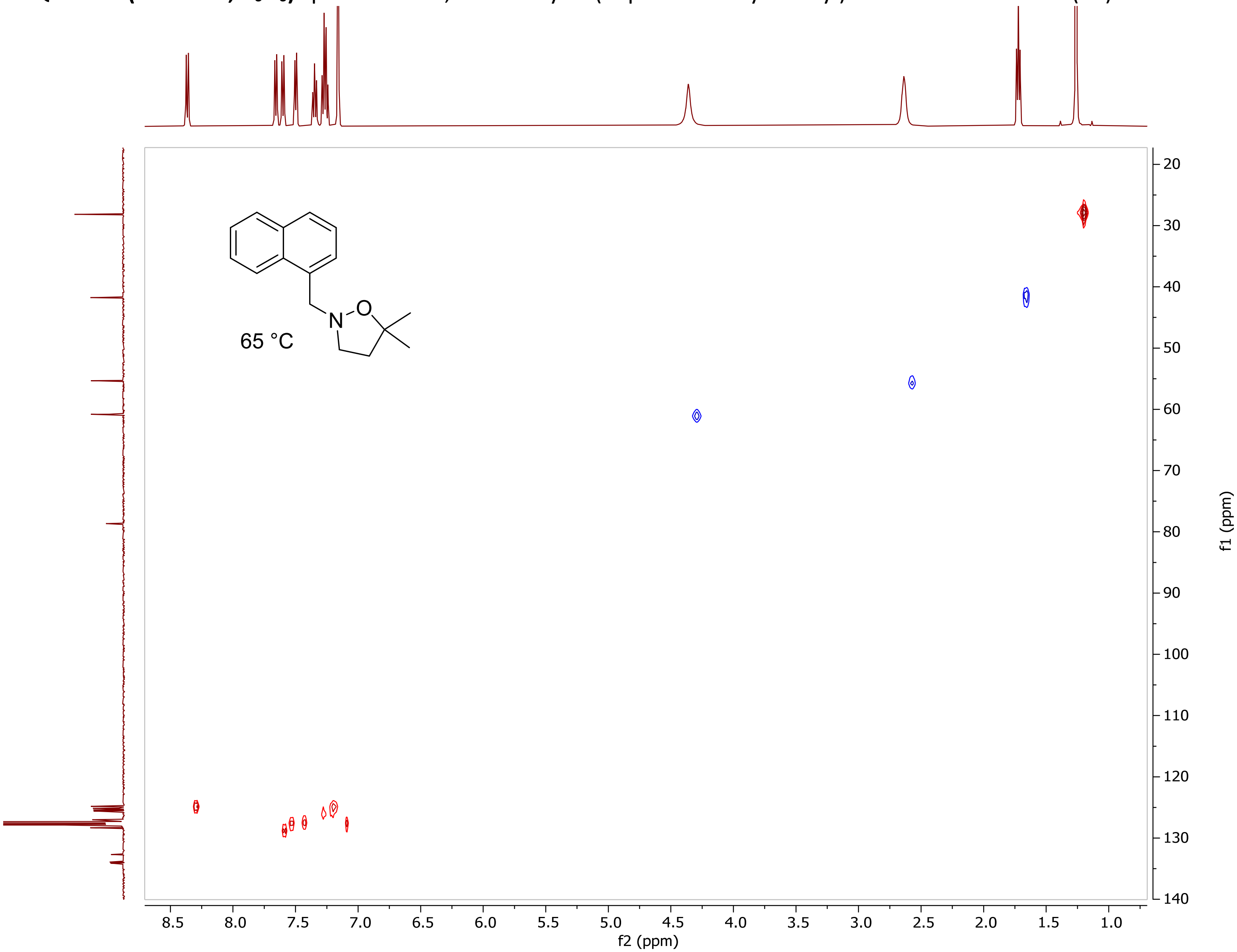

^{1}H NMR (500 MHz, CDCl$_3$) spectrum of 3-Methylbut-3-en-1-yl 4-methylbenzenesulfonate (**S11**)

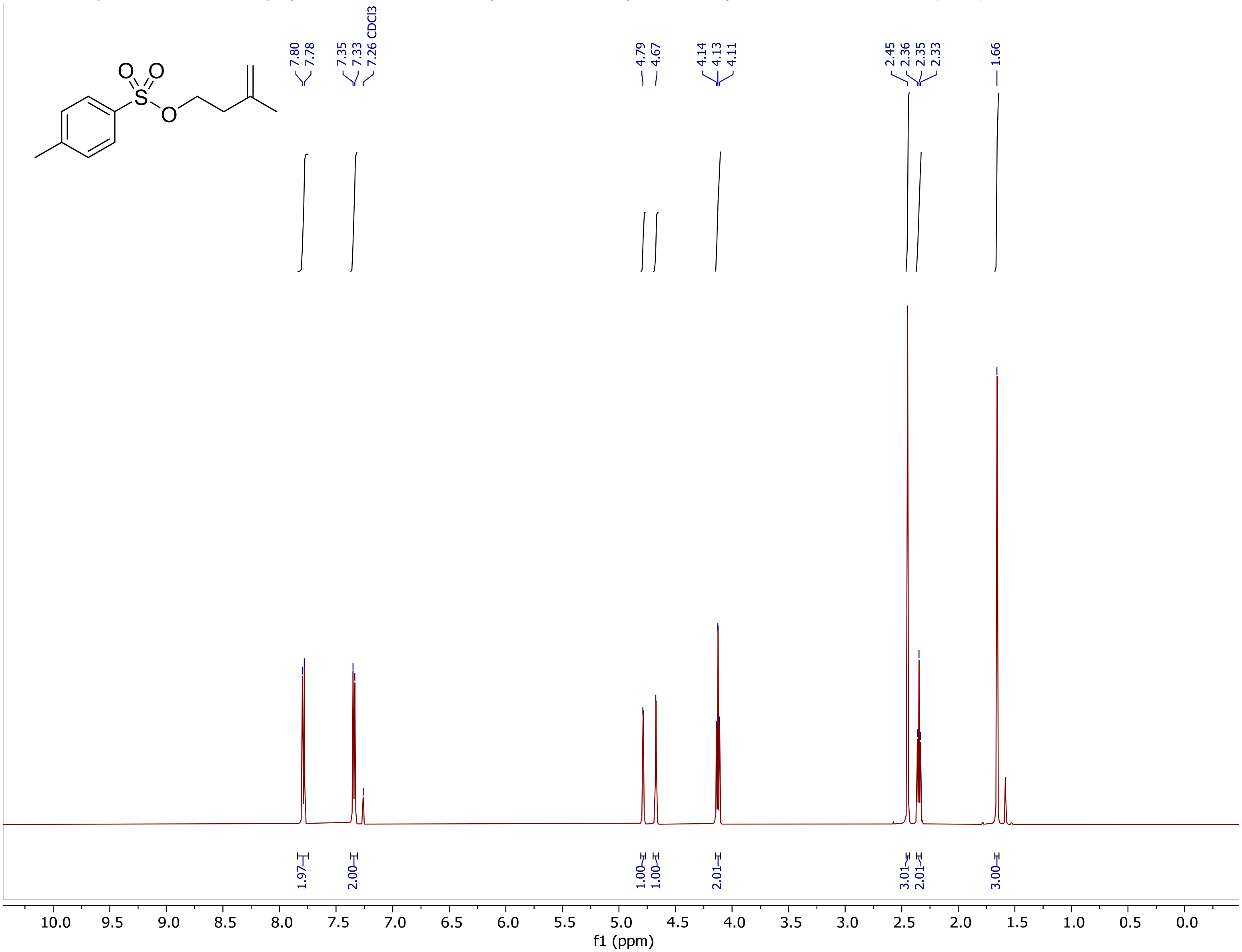

^{13}C {^{1}H} NMR (126 MHz, CDCl$_3$) spectrum of 3-Methylbut-3-en-1-yl 4-methylbenzenesulfonate (**S11**)

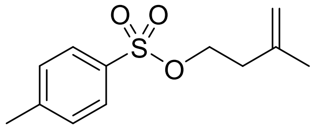

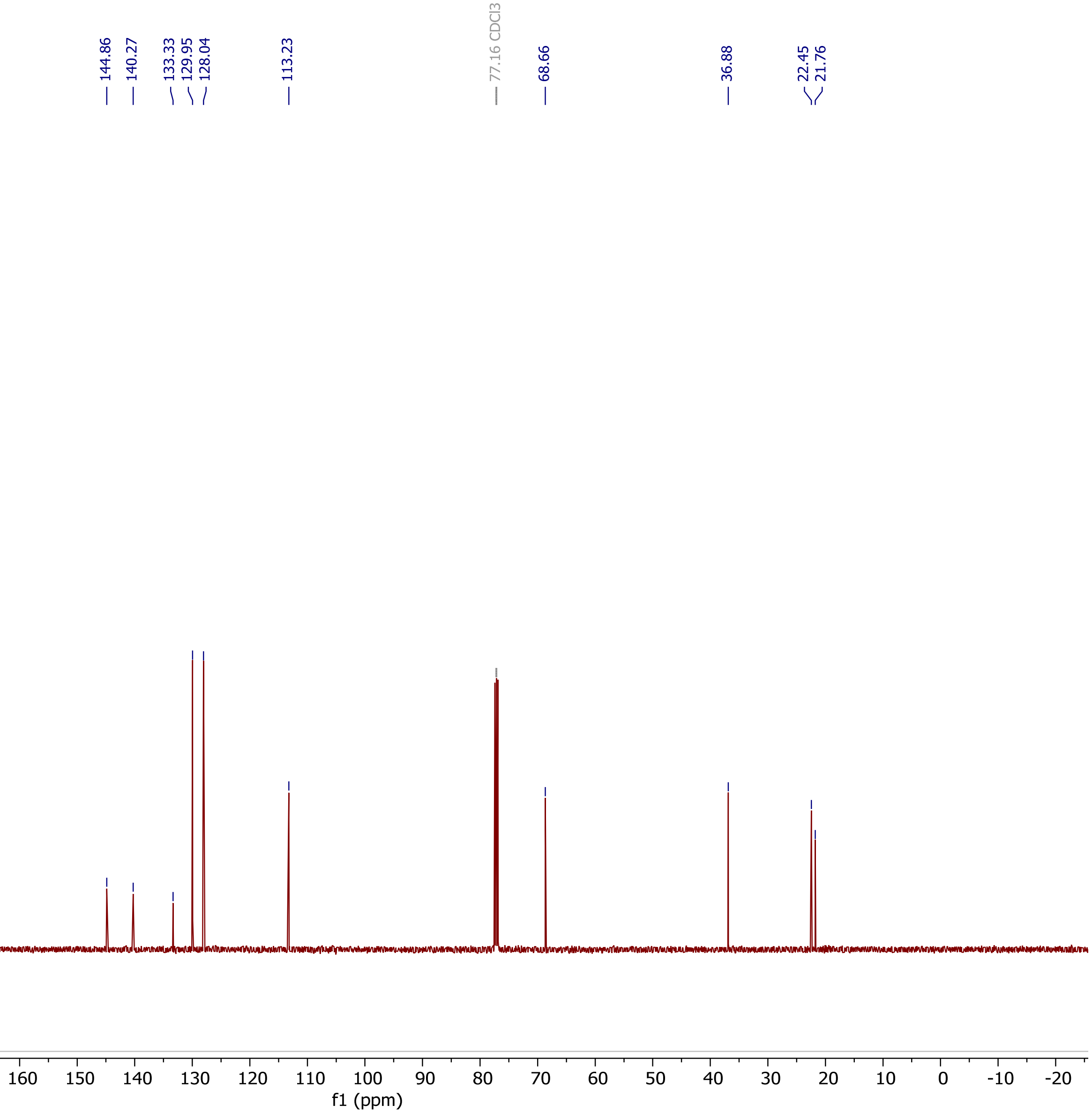

^{1}H NMR (500 MHz, CDCl$_3$) spectrum of *N*-(3-Methylbut-3-en-1-yl)-2-nitro-*N*-phenyl-4-(trifluoromethyl)benzenesulfonamide (**6g**)

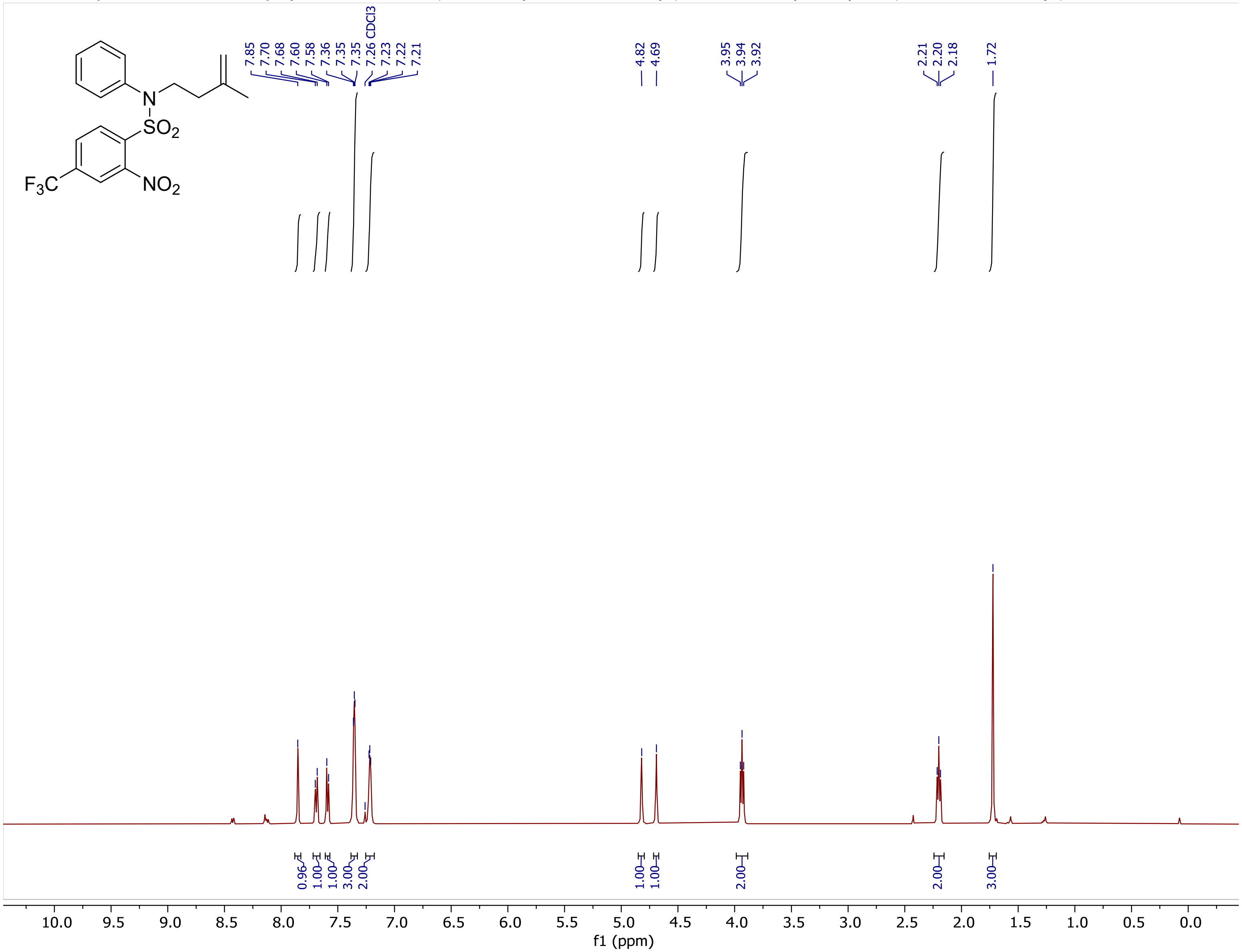

^{13}C {^{1}H} NMR (126 MHz, $CDCl_3$) spectrum of *N*-(3-Methylbut-3-en-1-yl)-2-nitro-*N*-phenyl-4-(trifluoromethyl)benzenesulfonamide (**6g**)

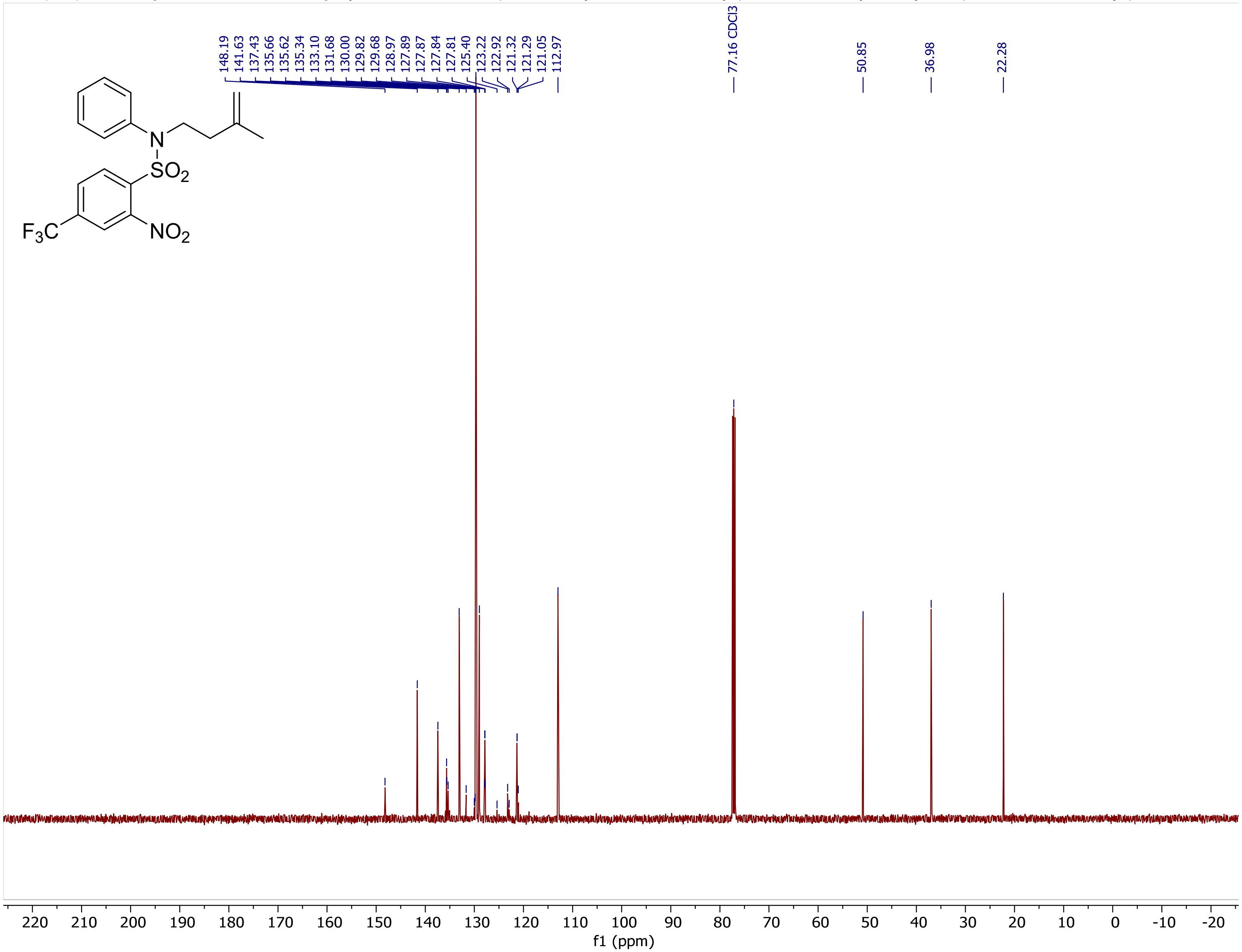

^{1}H NMR (500 MHz, $CDCl_3$) spectrum of *N*-(3-Methyl-3-((triethylsilyl)peroxy)butyl)-2-nitro-*N*-phenyl-4-(trifluoromethyl)benzenesulfonamide (**6h**)

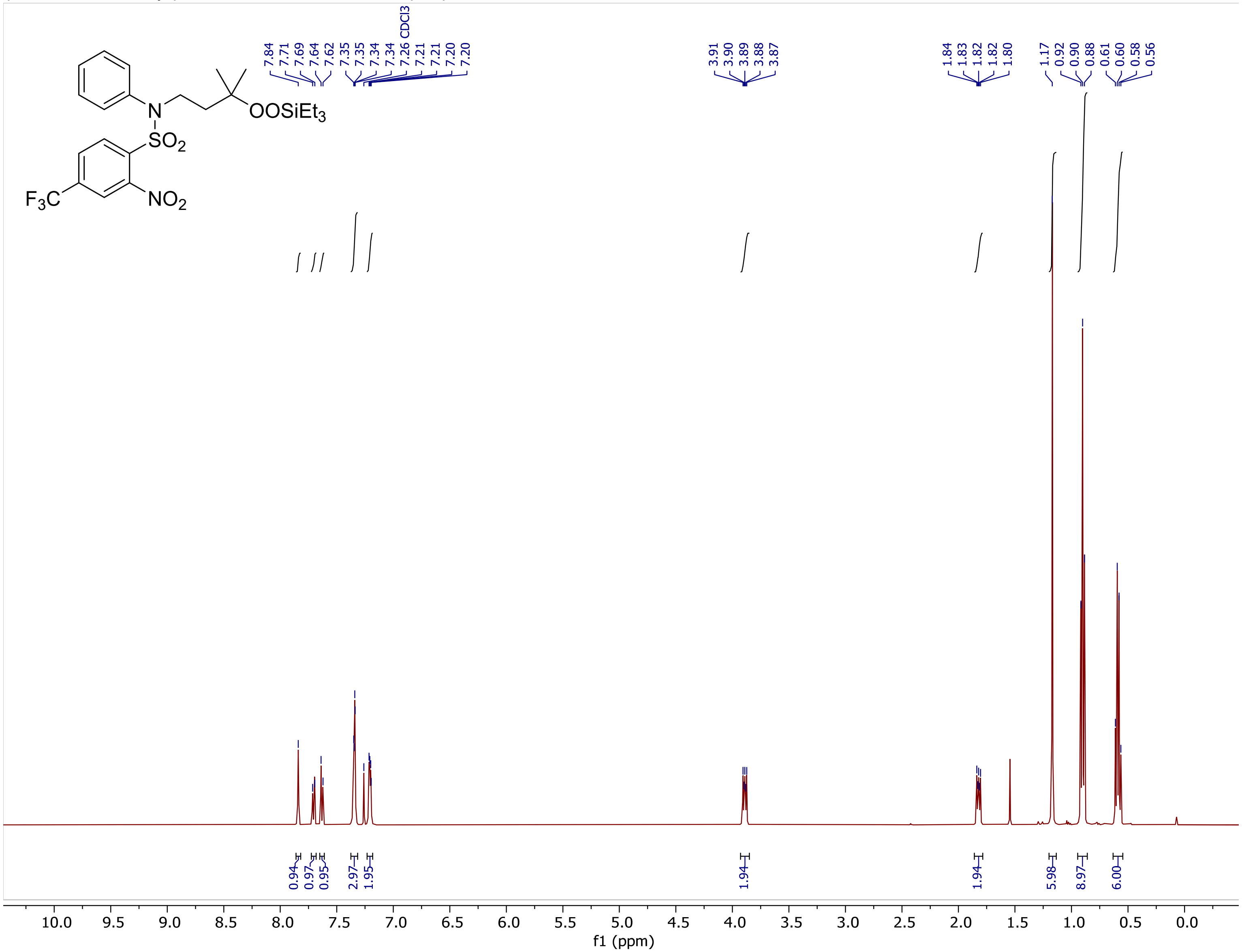

$^{13}C\ \{^{1}H\}$ NMR (126 MHz, $CDCl_3$) spectrum of *N*-(3-Methyl-3-((triethylsilyl)peroxy)butyl)-2-nitro-*N*-phenyl-4-(trifluoromethyl)benzenesulfonamide (**6h**)

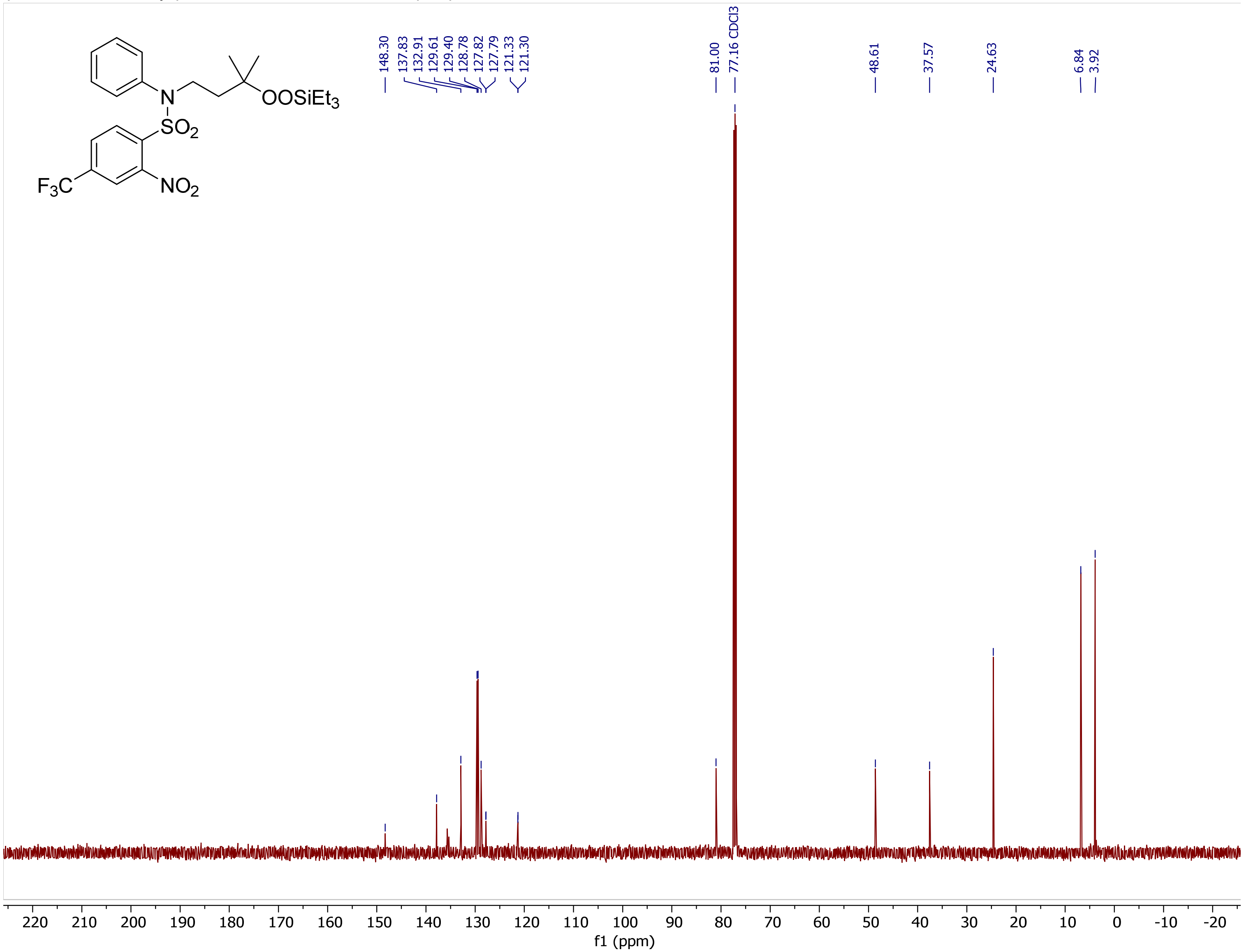

^{1}H NMR (500 MHz, C_6D_6) spectrum of *N*-(3-((*tert*-Butyldiphenylsilyl)peroxy)-3-methylbutyl)-2-nitro-*N*-phenyl-4-(trifluoromethyl)benzenesulfonamide (**47**)

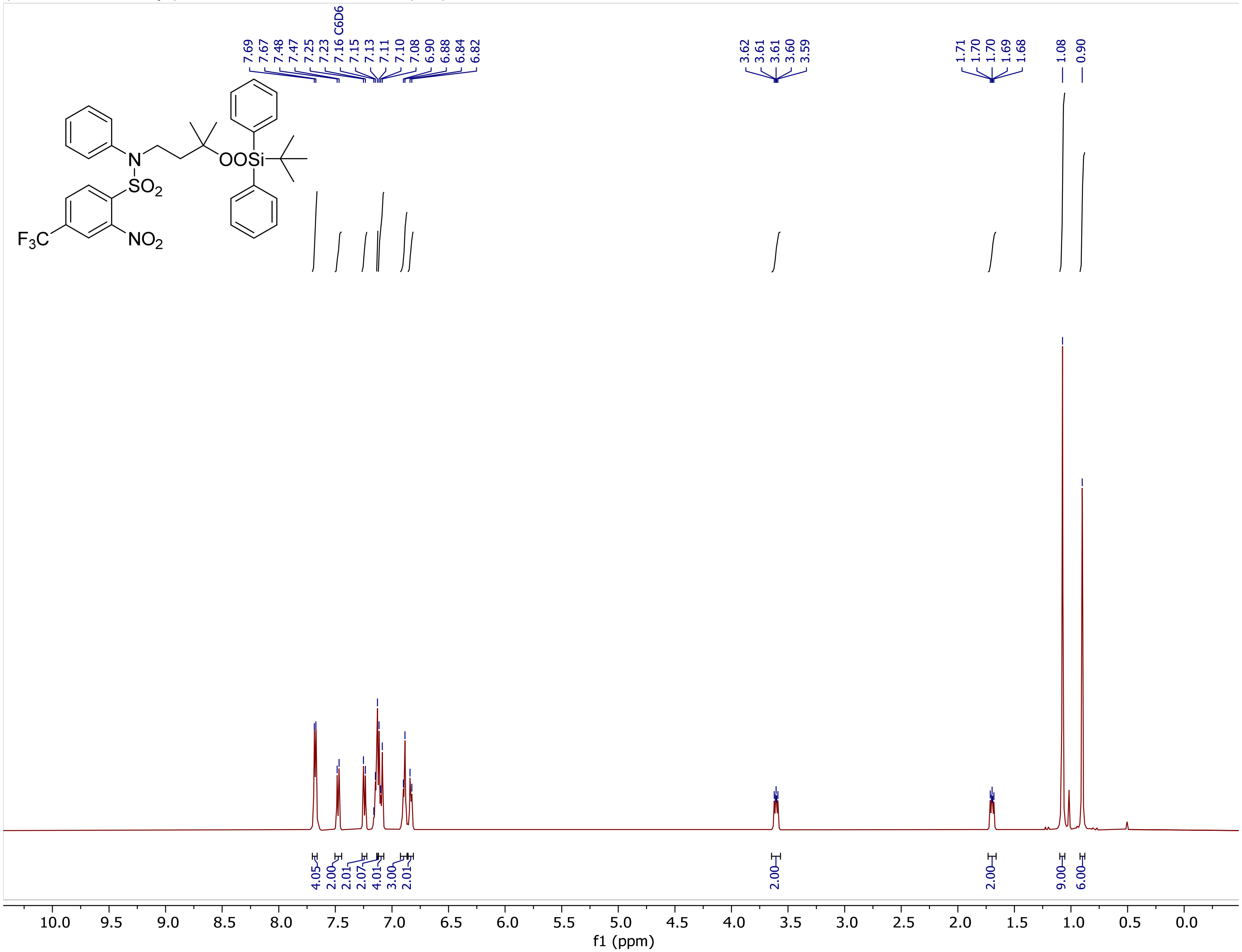

^{13}C {^{1}H, ^{19}F} NMR (126 MHz, C_6D_6) spectrum of *N*-(3-((*tert*-Butyldiphenylsilyl)peroxy)-3-methylbutyl)-2-nitro-*N*-phenyl-4-(trifluoromethyl)benzenesulfonamide (**47**)

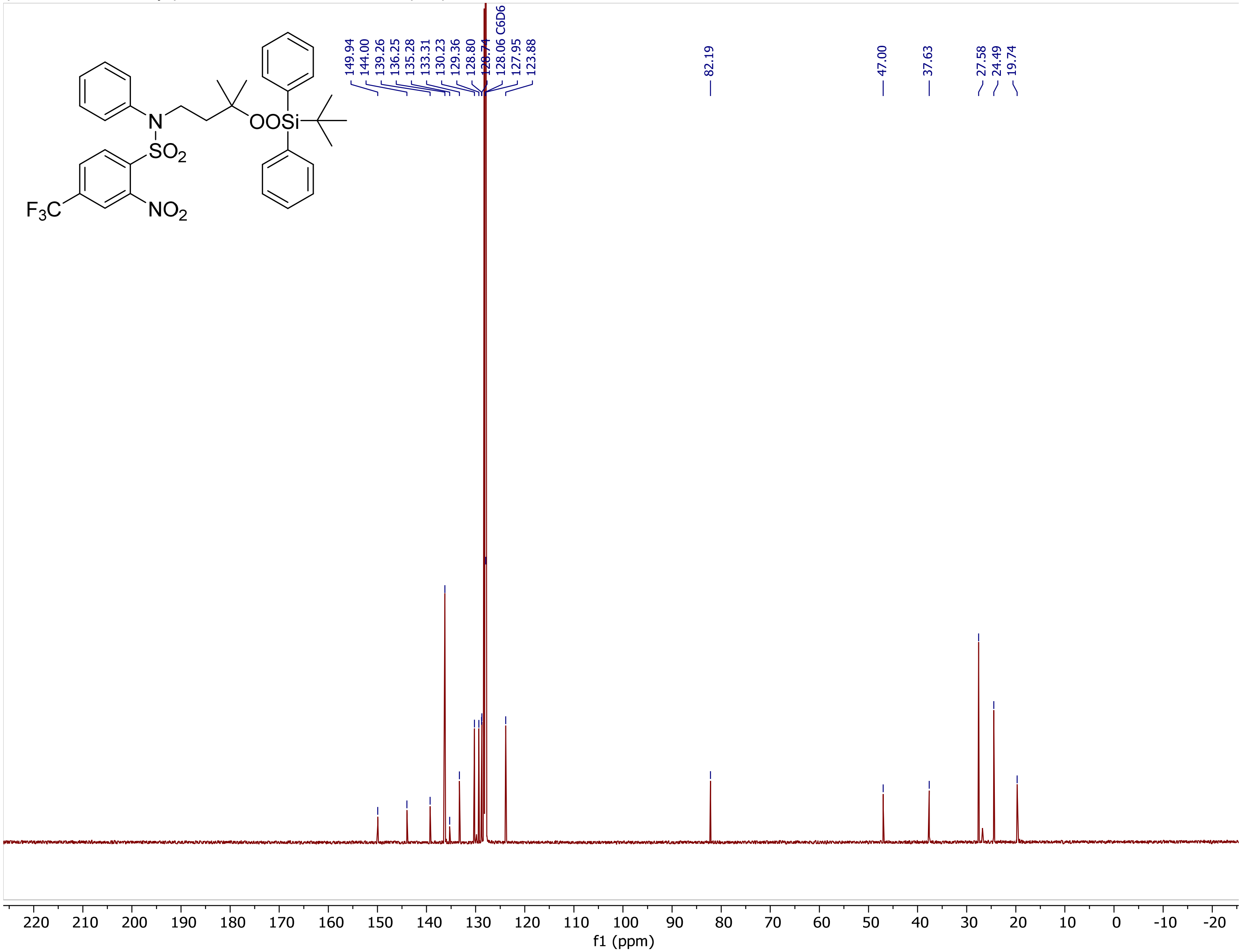

^{1}H NMR (500 MHz, C_6D_6) spectrum of *N*-(3-((tert-Butyldiphenylsilyl)peroxy)-3-methylbutyl)-aniline (**6i**)

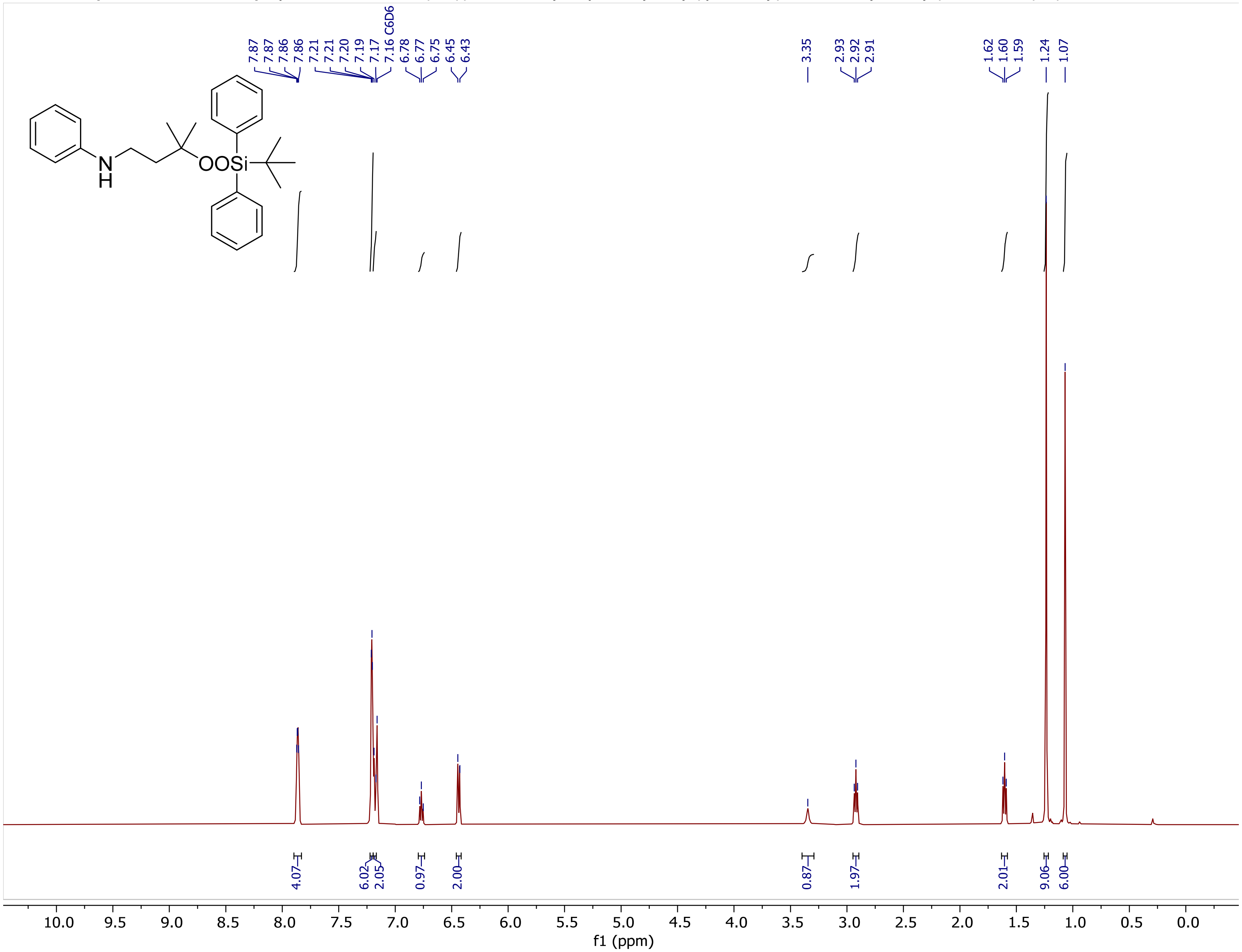

$^{13}C\{^1H\}$ NMR (126 MHz, C_6D_6) spectrum of *N*-(3-((*tert*-Butyldiphenylsilyl)peroxy)-3-methylbutyl)-aniline (**6i**)

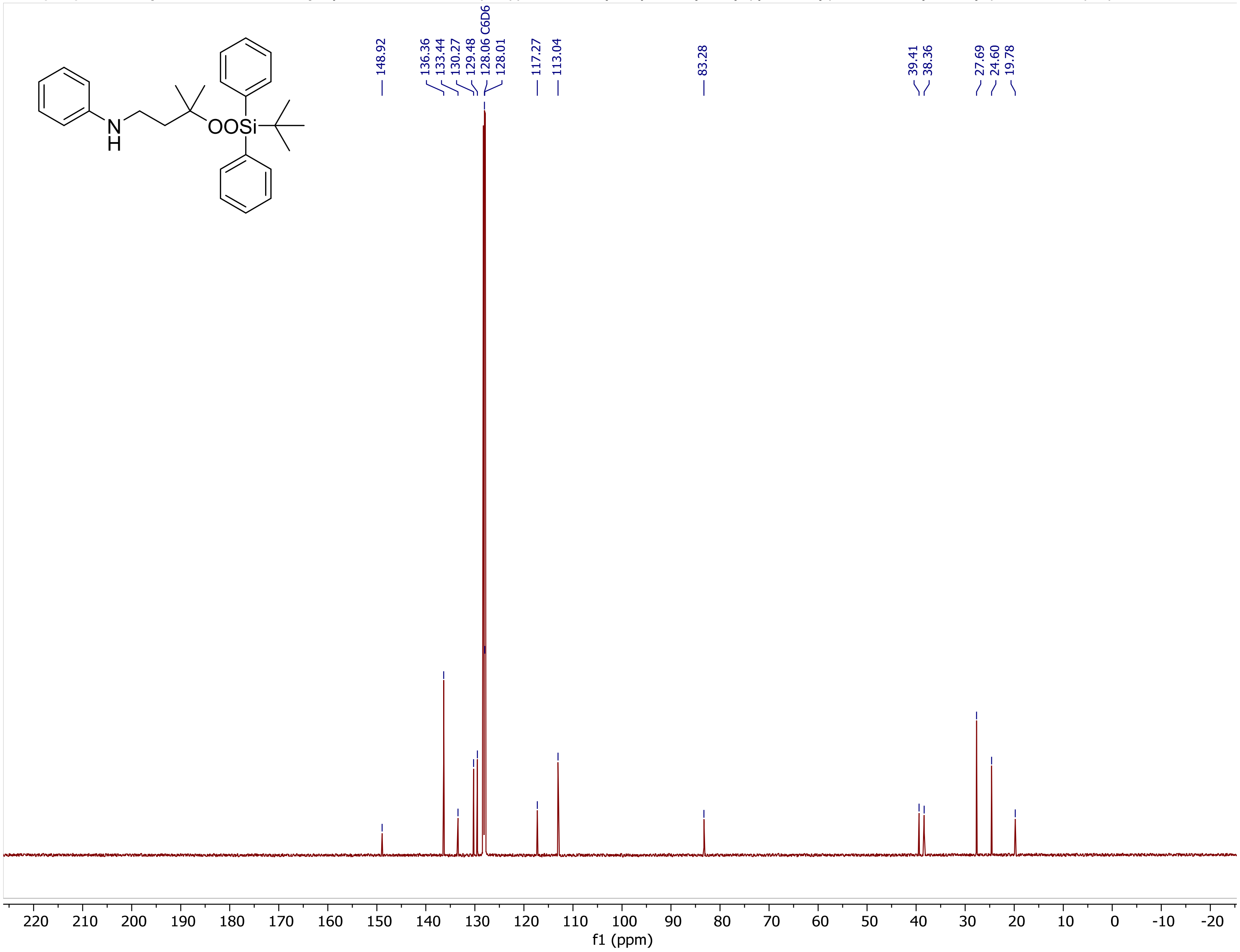

¹H NMR (500 MHz, CDCl₃) spectrum of 5,5-Dimethyl-2-phenylisoxazolidine (**48**)

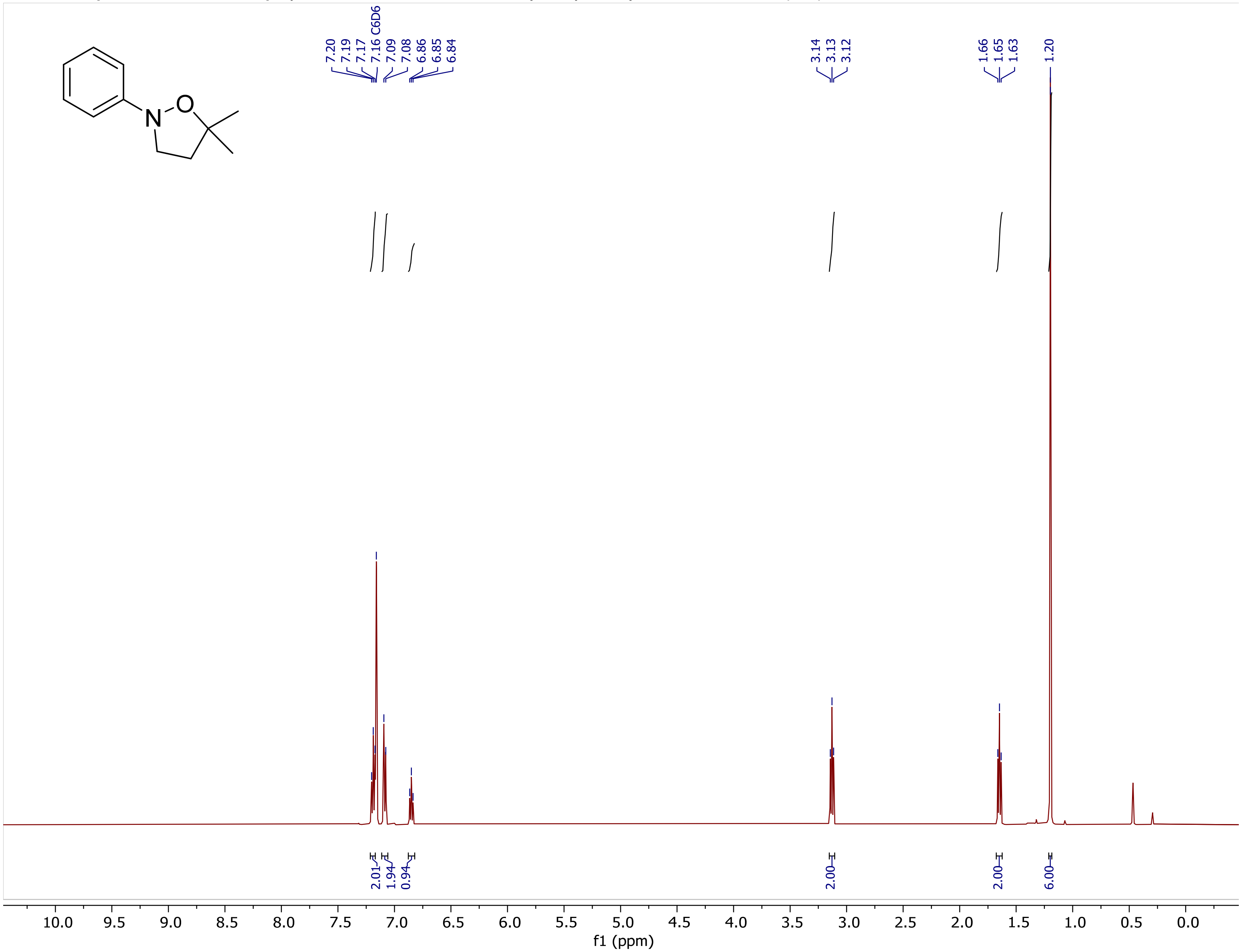

^{13}**C {^{1}H} NMR (126 MHz, CDCl$_3$)** spectrum of 5,5-Dimethyl-2-phenylisoxazolidine (**48**)

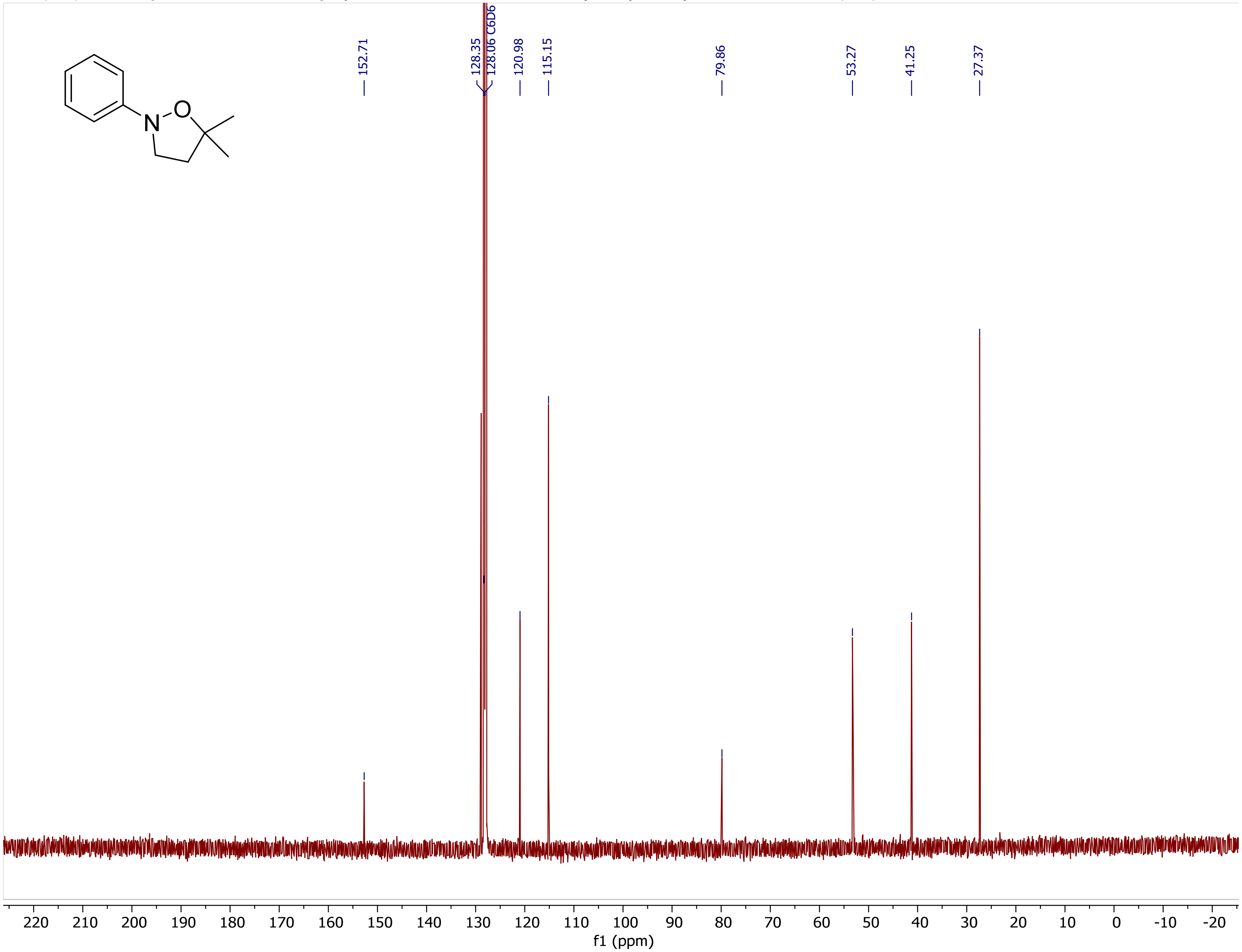

HSQC NMR (500 MHz, $CDCl_3$) spectrum of 5,5-Dimethyl-2-phenylisoxazolidine (**48**)

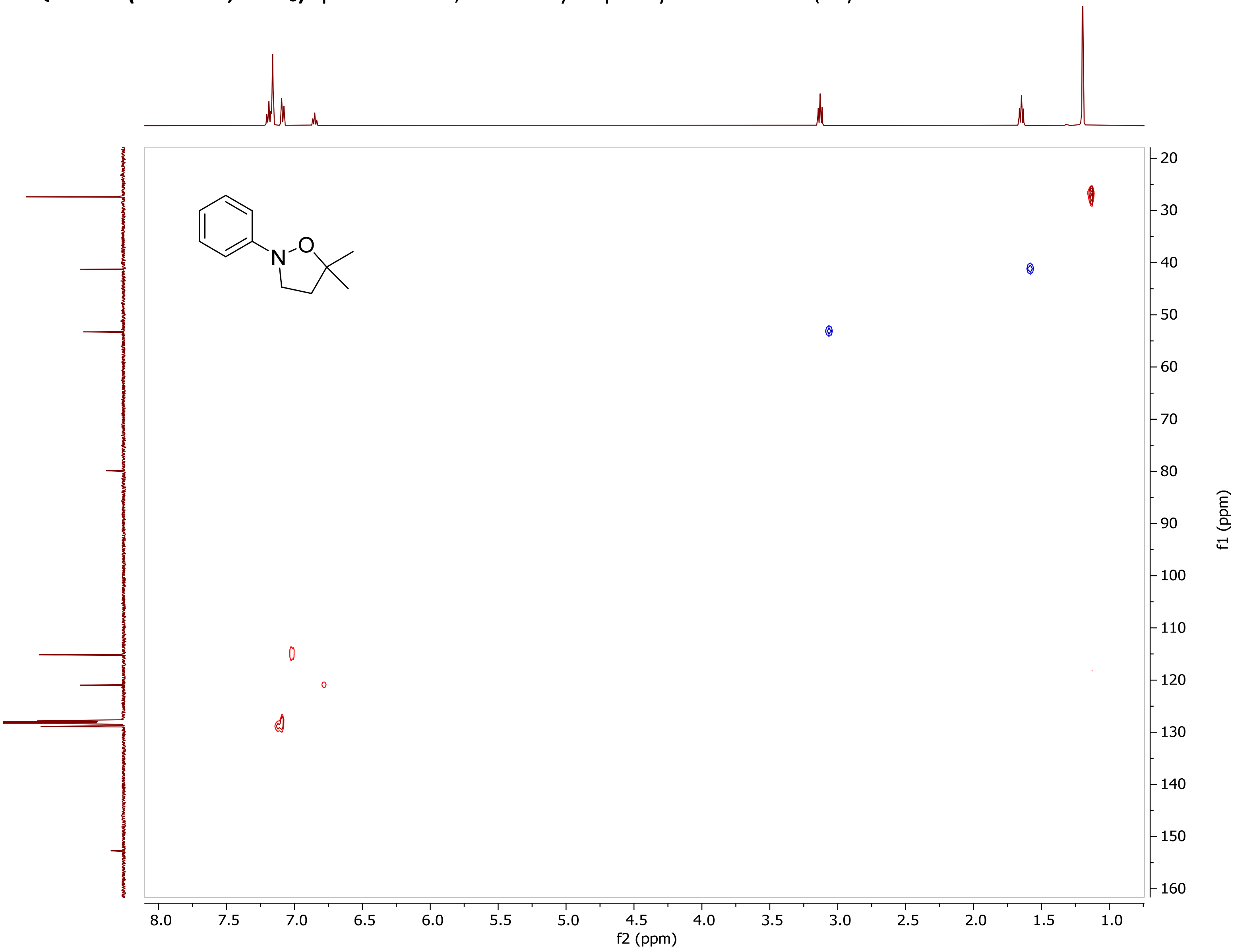

^{1}H NMR (500 MHz, $CDCl_3$) spectrum of 2-(Cyclohex-1-en-yl)ethan-1-ol (**S12**)

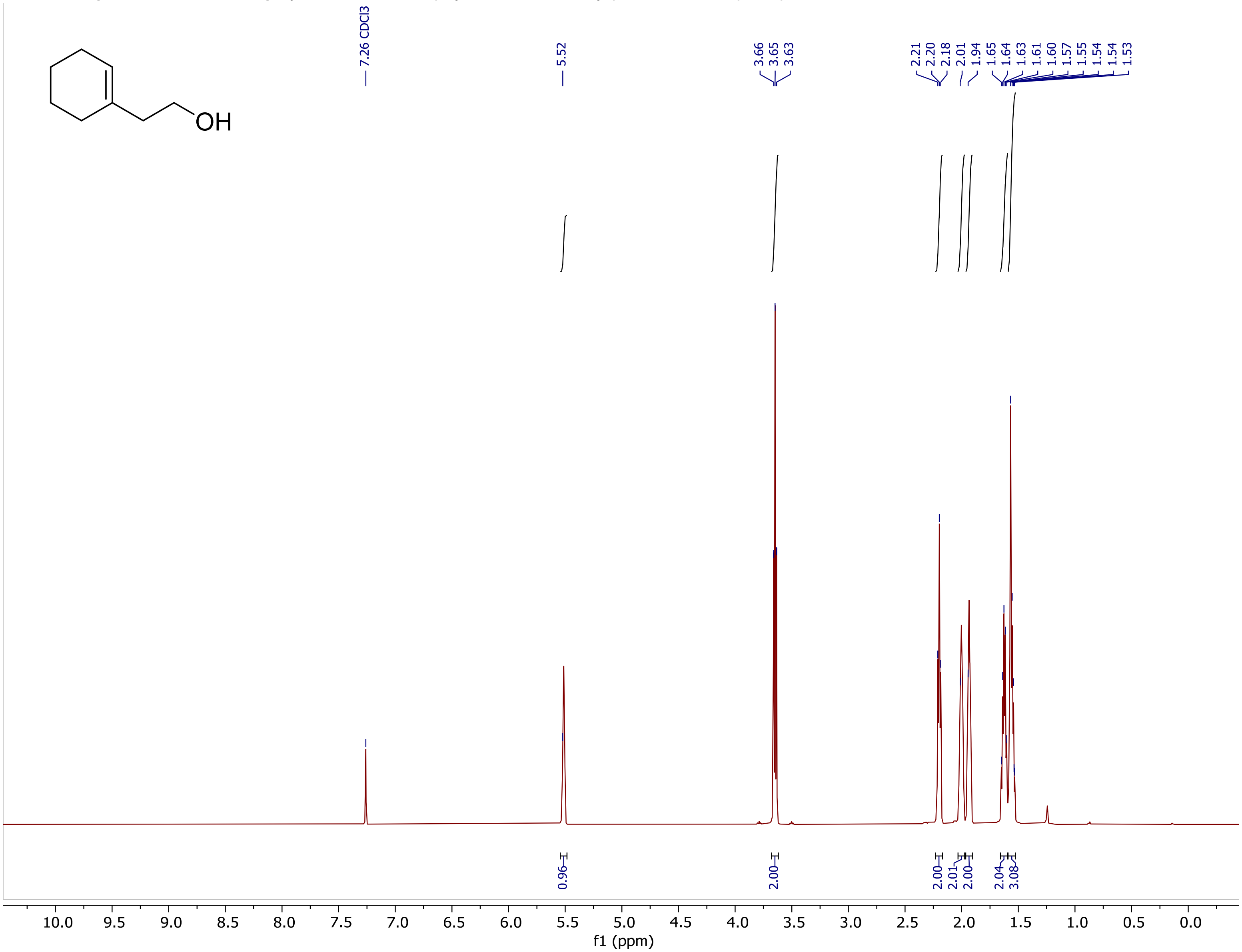

$^{12}C\ \{^1H\}$ NMR (126 MHz, $CDCl_3$) spectrum of 2-(Cyclohex-1-en-yl)ethan-1-ol (**S12**)

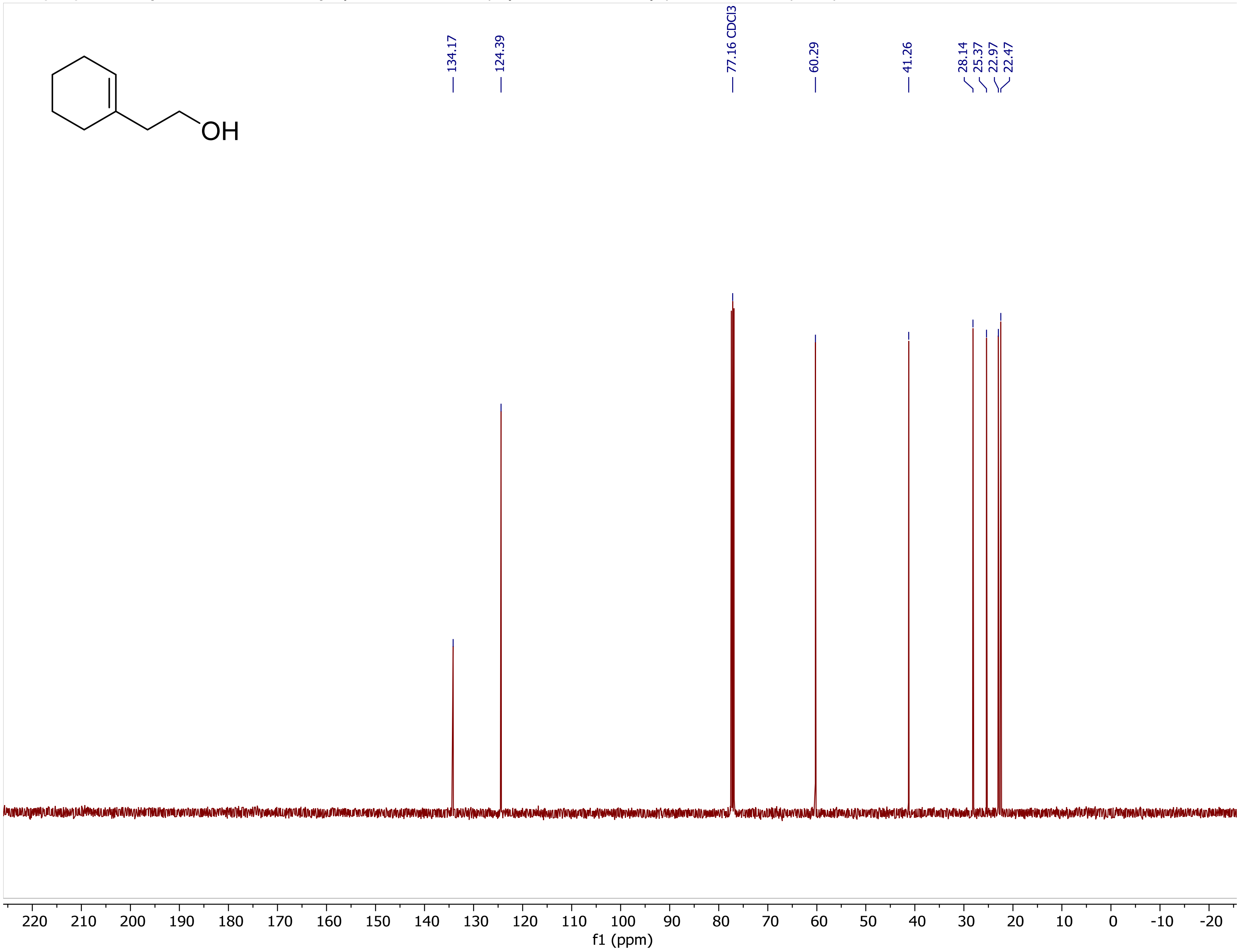

^{1}H NMR (500 MHz, CDCl$_3$) spectrum of *N*-(2-(Cyclohex-1-en-1-yl)ethyl)-2-nitro-*N*-phenyl-4-(trifluoromethyl)benzenesulfonamide (**12a**)

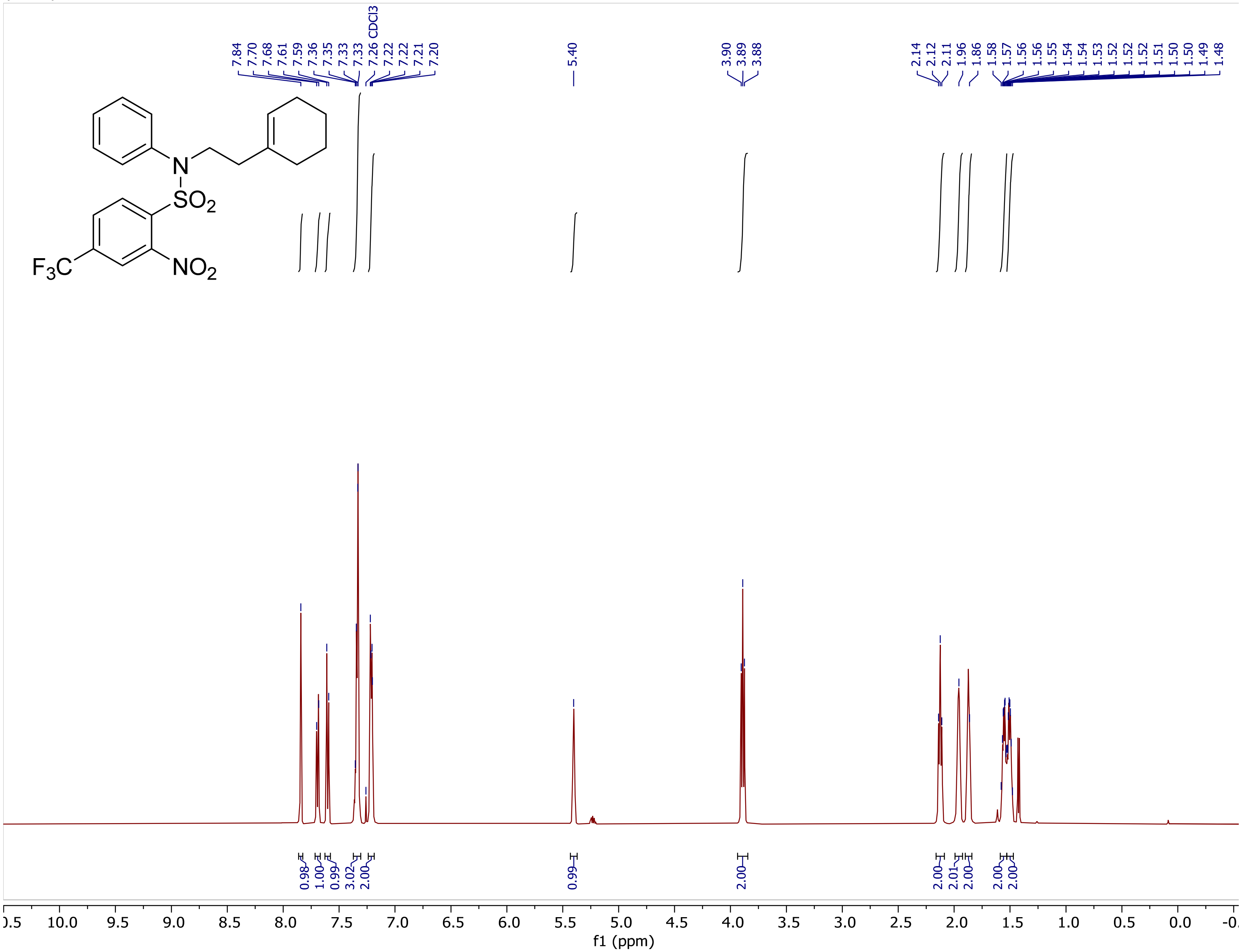

^{13}C {^{1}H} NMR (126 MHz, $CDCl_3$) spectrum of *N*-(2-(Cyclohex-1-en-1-yl)ethyl)-2-nitro-*N*-phenyl-4-(trifluoromethyl)benzenesulfonamide (**12a**)

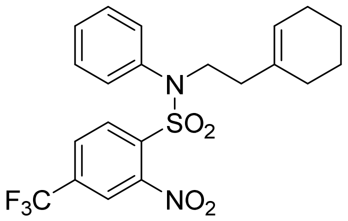

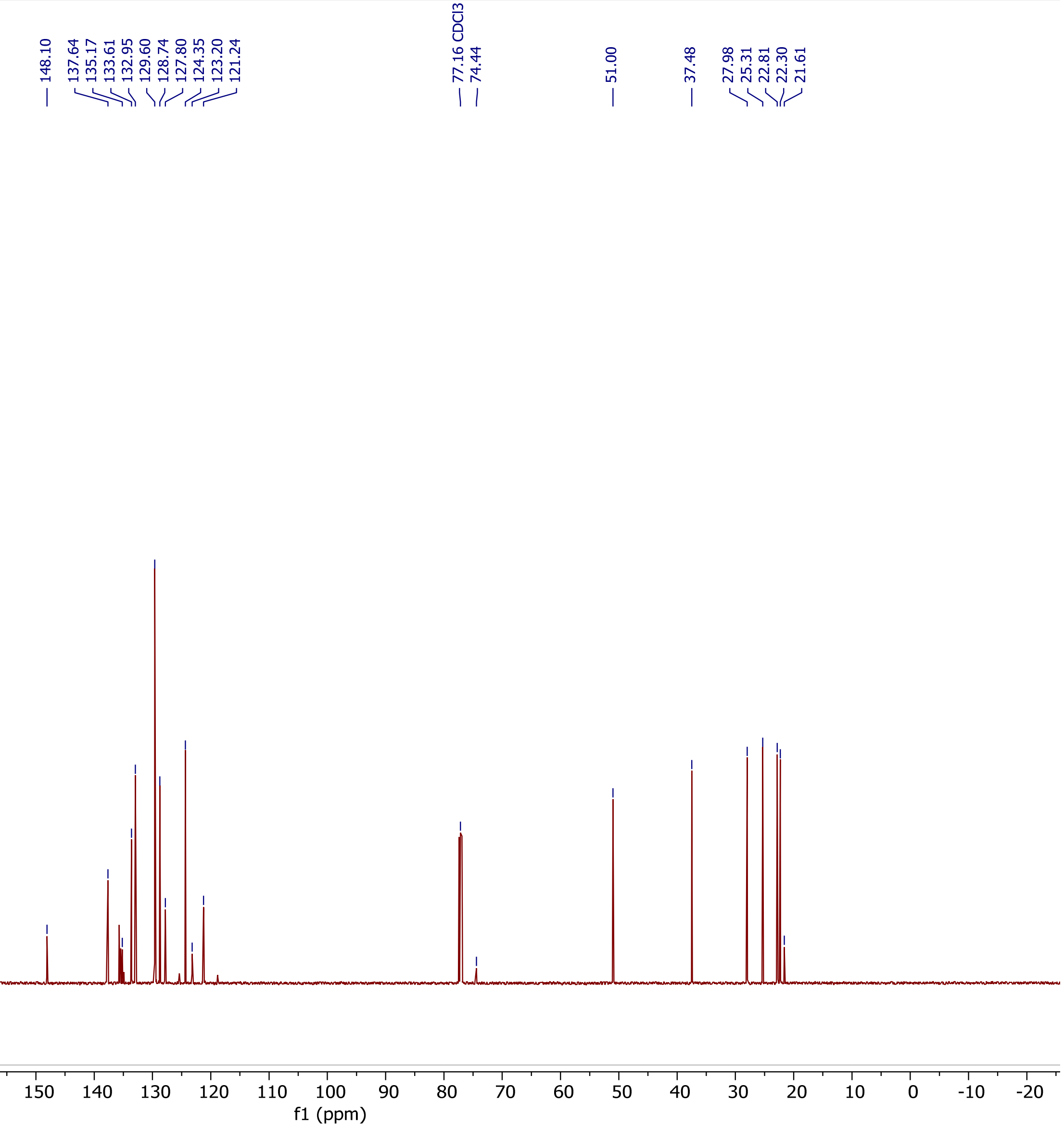

^{1}H NMR (500 MHz, CDCl$_3$) spectrum of 2-Nitro-*N*-phenyl-*N*-(2-(1-((triethylsilyl)peroxy)cyclohexyl)ethyl)-4-(trifluoromethyl)benzenesulfonamide (**12b**)

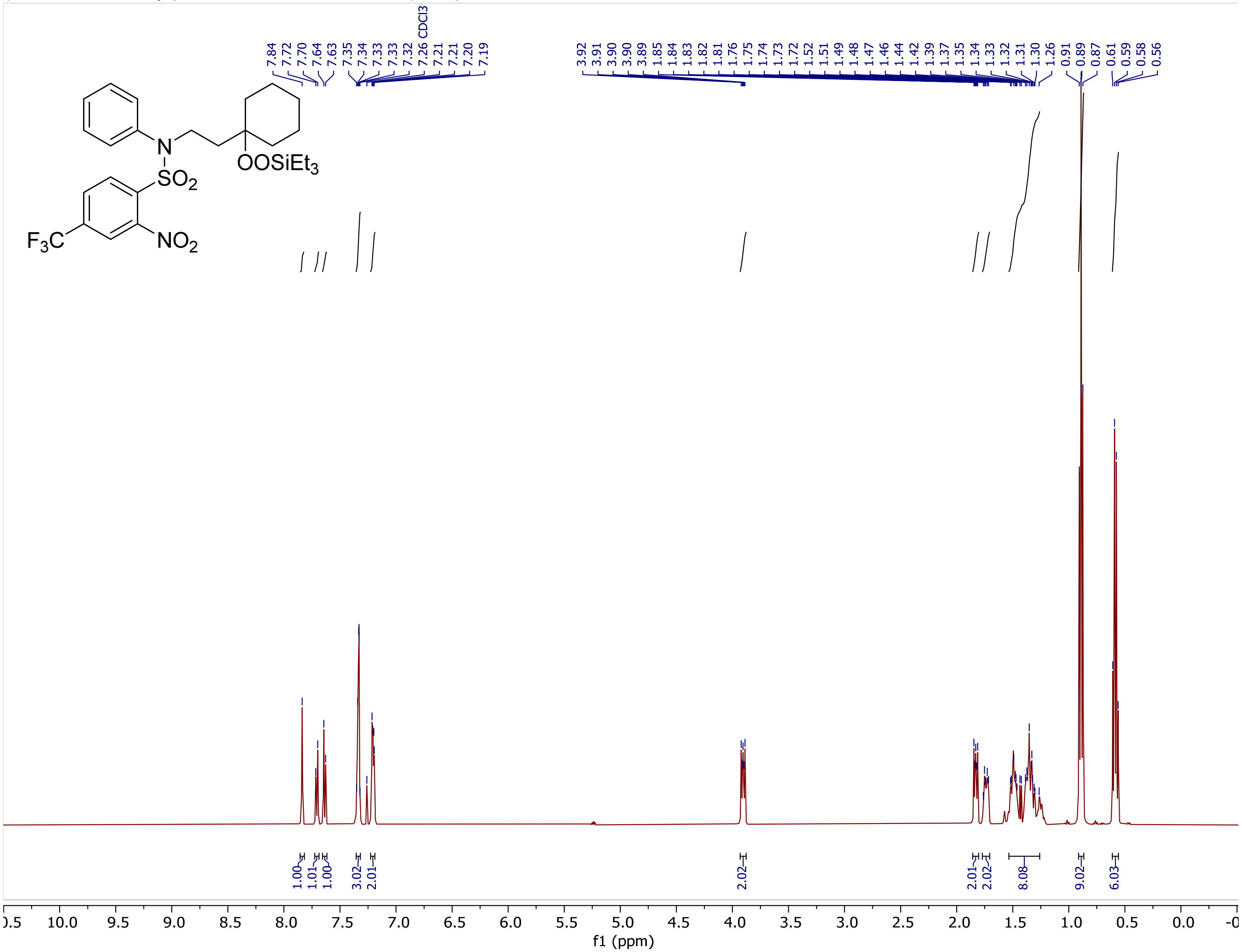

^{13}C {^{1}H} NMR (126 MHz, $CDCl_3$) spectrum of 2-Nitro-*N*-phenyl-*N*-(2-(1-((triethylsilyl)peroxy)cyclohexyl)ethyl)-4-(trifluoromethyl)benzenesulfonamide (**12b**)

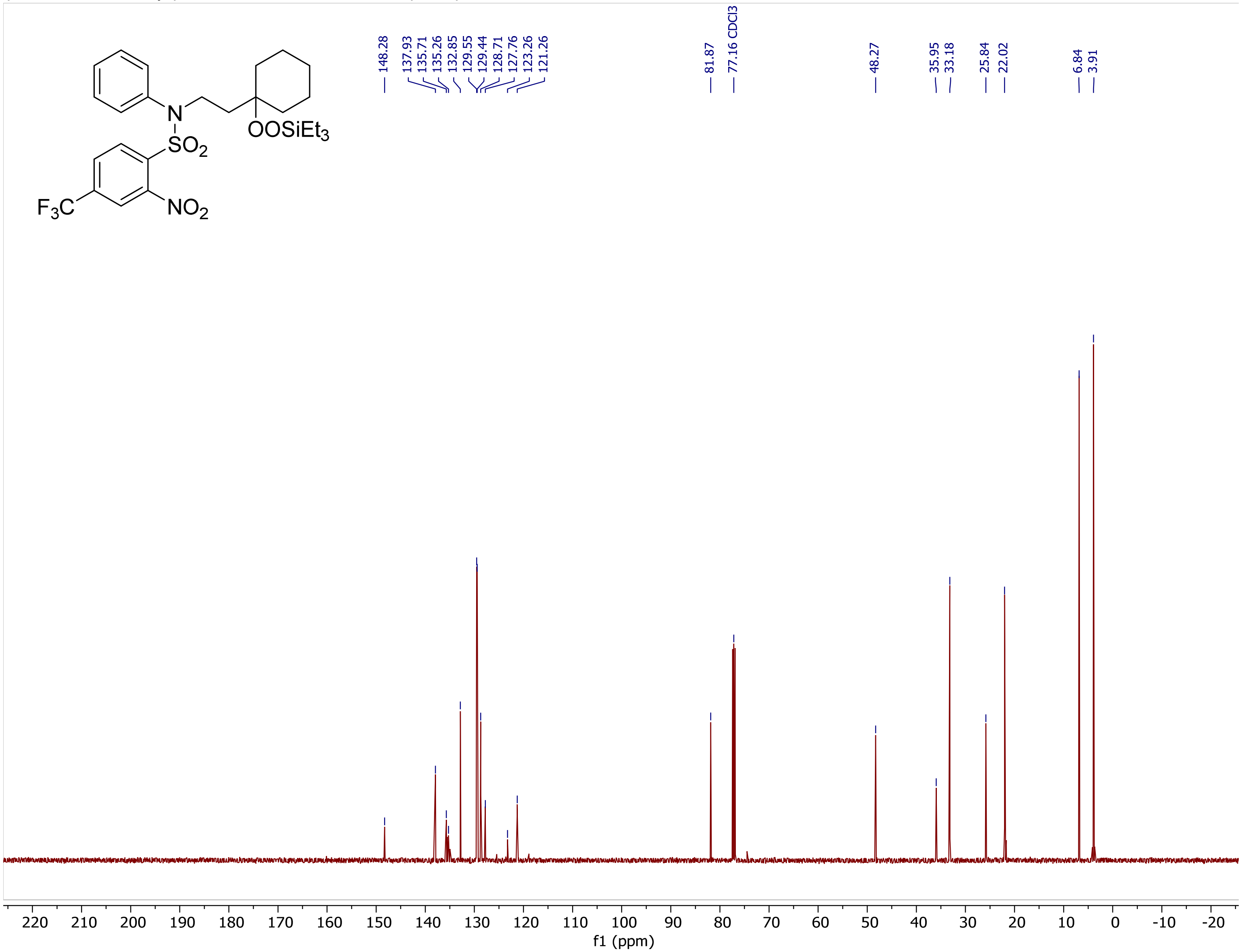

^{1}H NMR (500 MHz, C_6D_6) spectrum of *N*-(2-(1-((*tert*-Butyldiphenylsilyl)peroxy)cyclohexyl)ethyl)-2-nitro-*N*-phenyl-4-(trifluoromethyl)benzenesulfonamide (**49**)

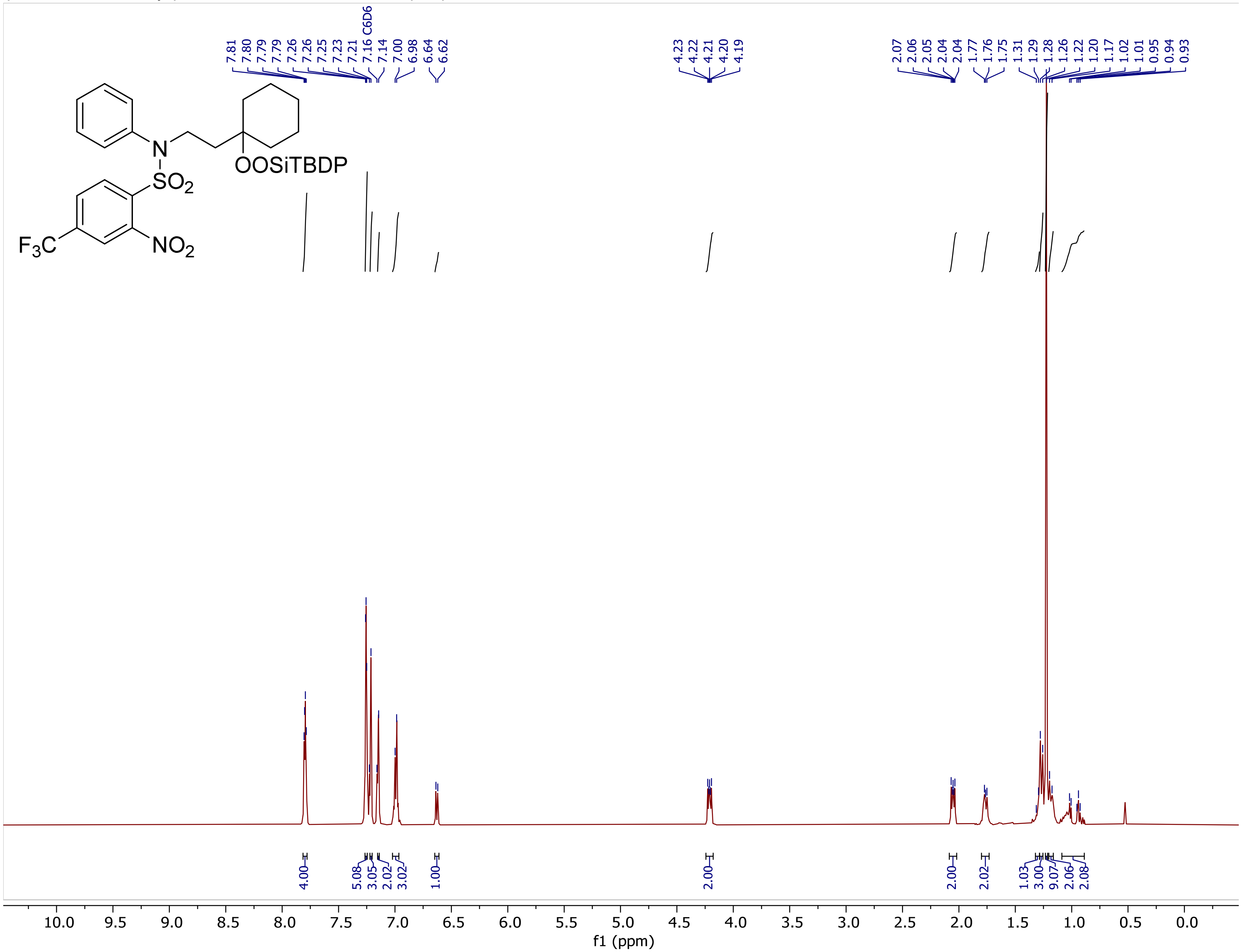

^{13}C {^{1}H} NMR (126 MHz, C_6D_6) spectrum of *N*-(2-(1-((*tert*-Butyldiphenylsilyl)peroxy)cyclohexyl)ethyl)-2-nitro-*N*-phenyl-4-(trifluoromethyl)benzenesulfonamide (**49**)

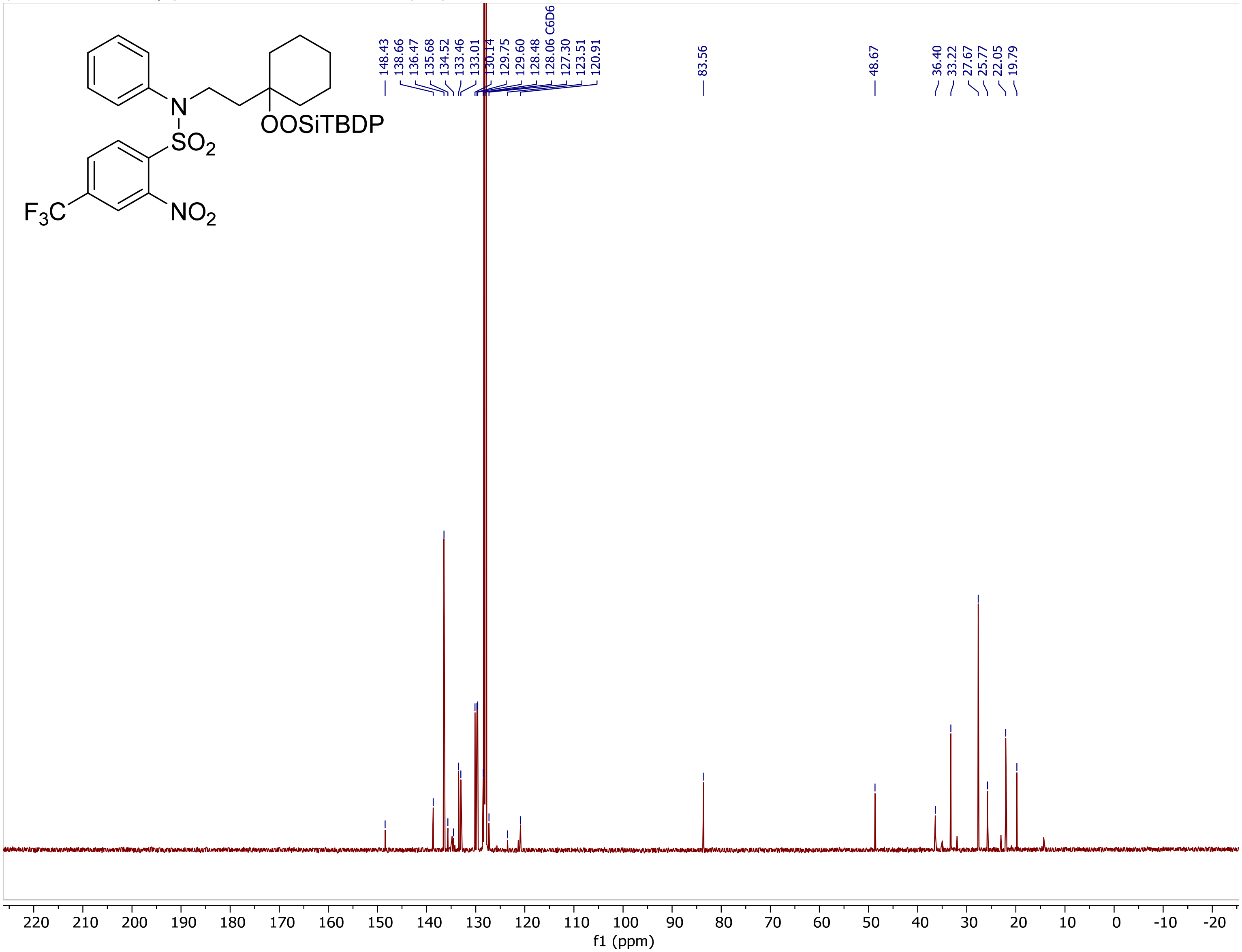

^{1}H NMR (500 MHz, C_6D_6) spectrum of *N*-(2-(1-((*tert*-Butyldiphenylsilyl)peroxy)cyclohexyl)ethyl)aniline (**12c**)

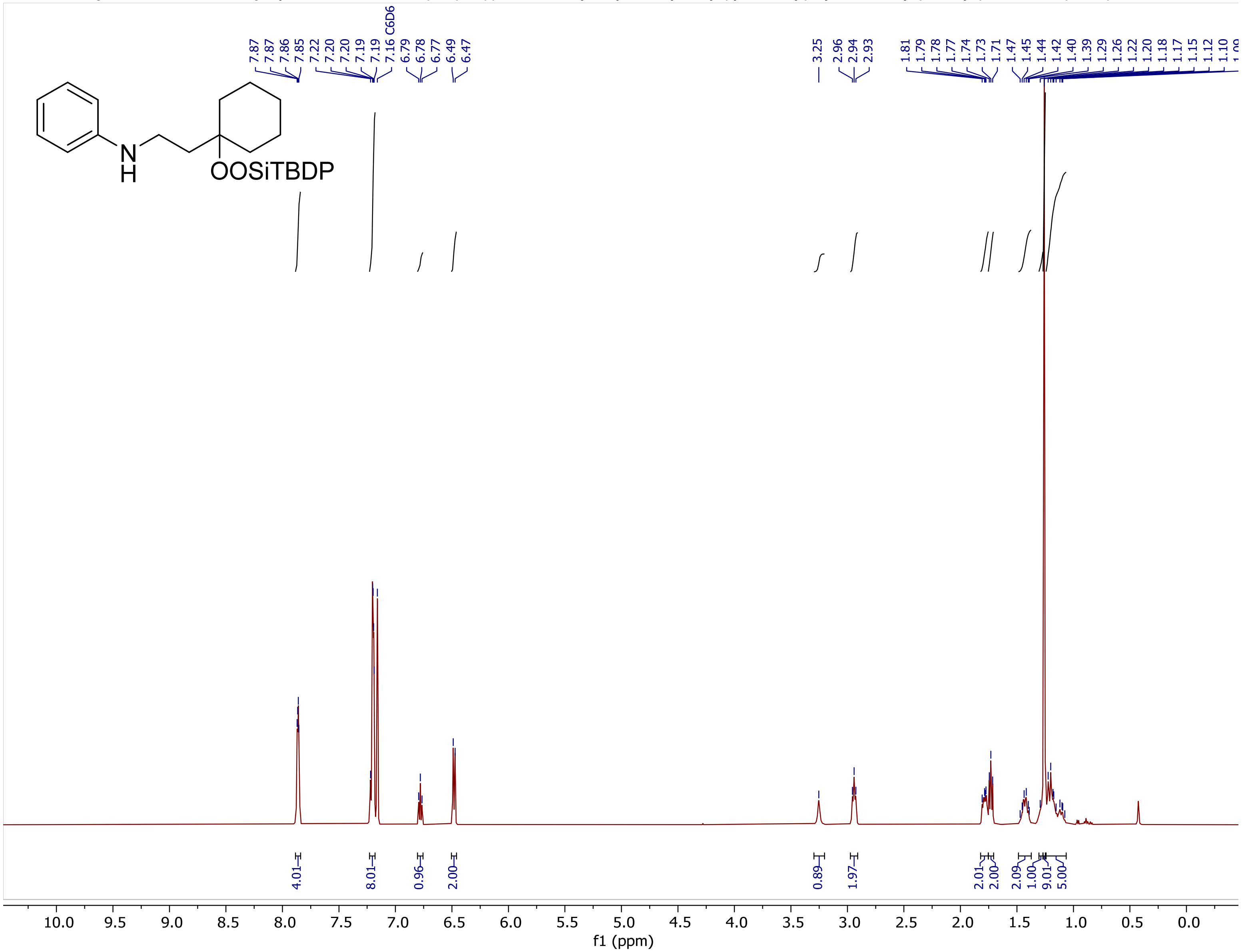

$^{13}C\{^1H\}$ NMR (126 MHz, C_6D_6) spectrum of *N*-(2-(1-((*tert*-Butyldiphenylsilyl)peroxy)cyclohexyl)ethyl)aniline (**12c**)

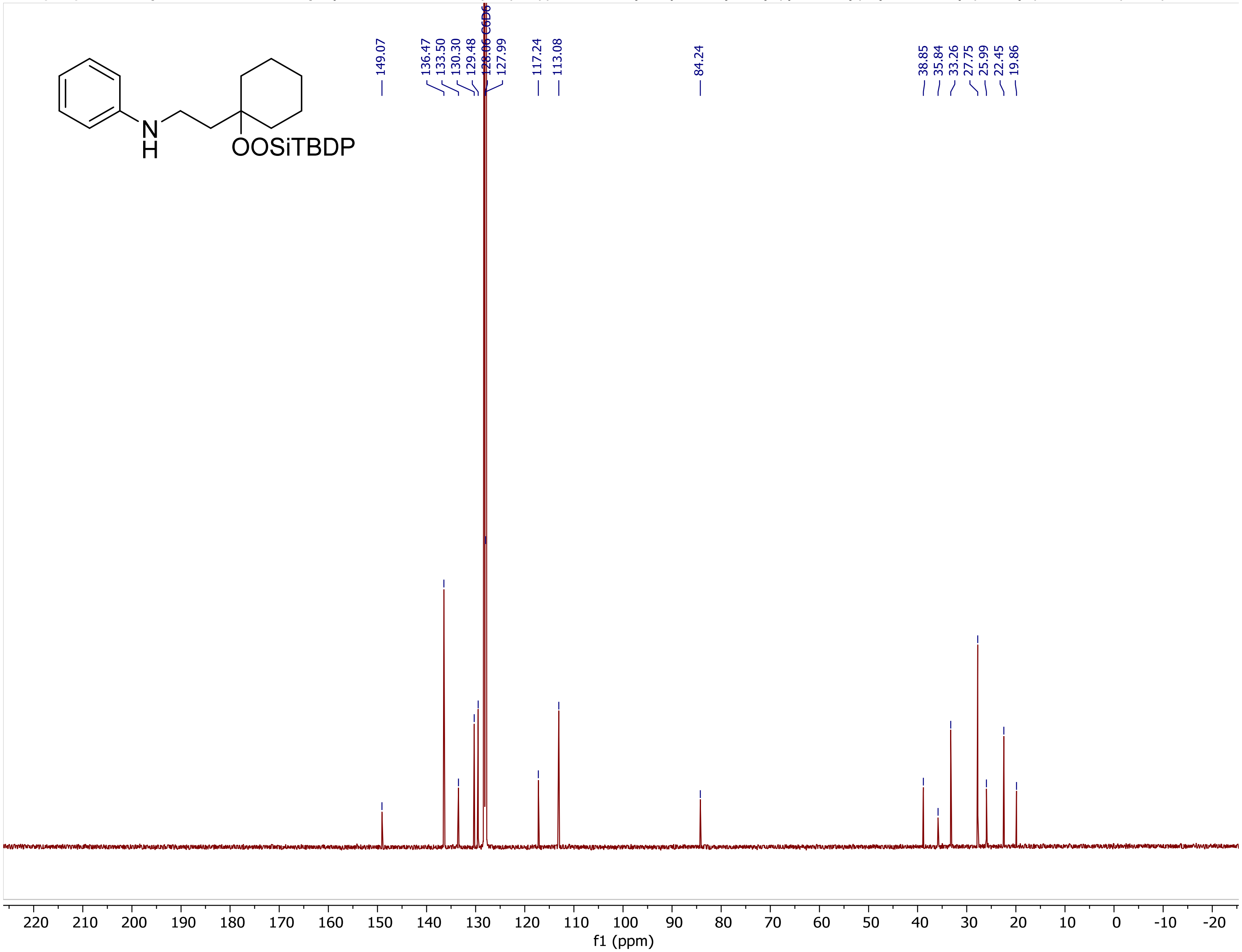

^{1}H NMR (500 MHz, C_6D_6) spectrum of 2-Phenyl-1-oxa-2-azaspiro[4.5]decane (**50**)

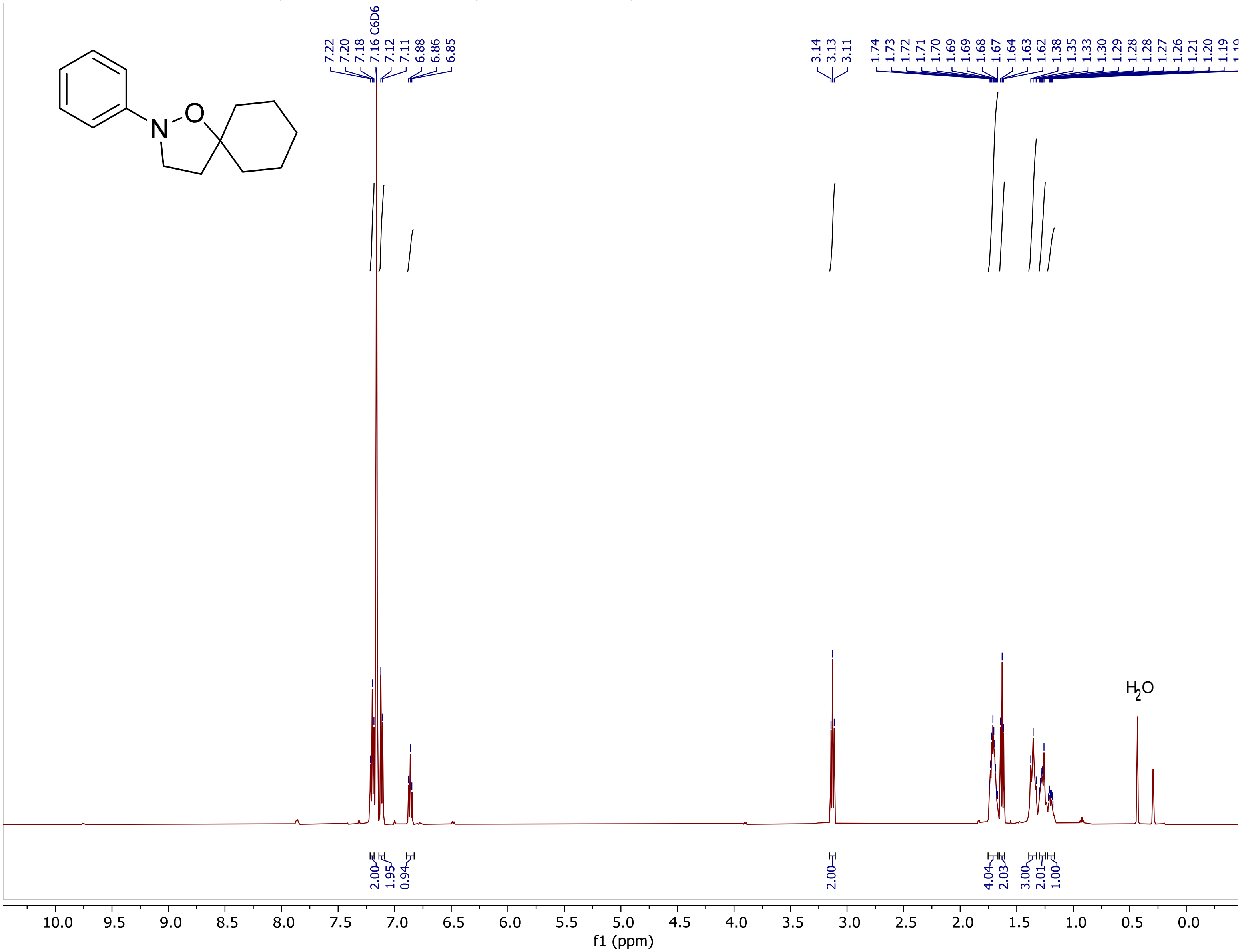

$^{13}C\{^1H\}$ NMR (126 MHz, C_6D_6) spectrum of 2-Phenyl-1-oxa-2-azaspiro[4.5]decane (**50**)

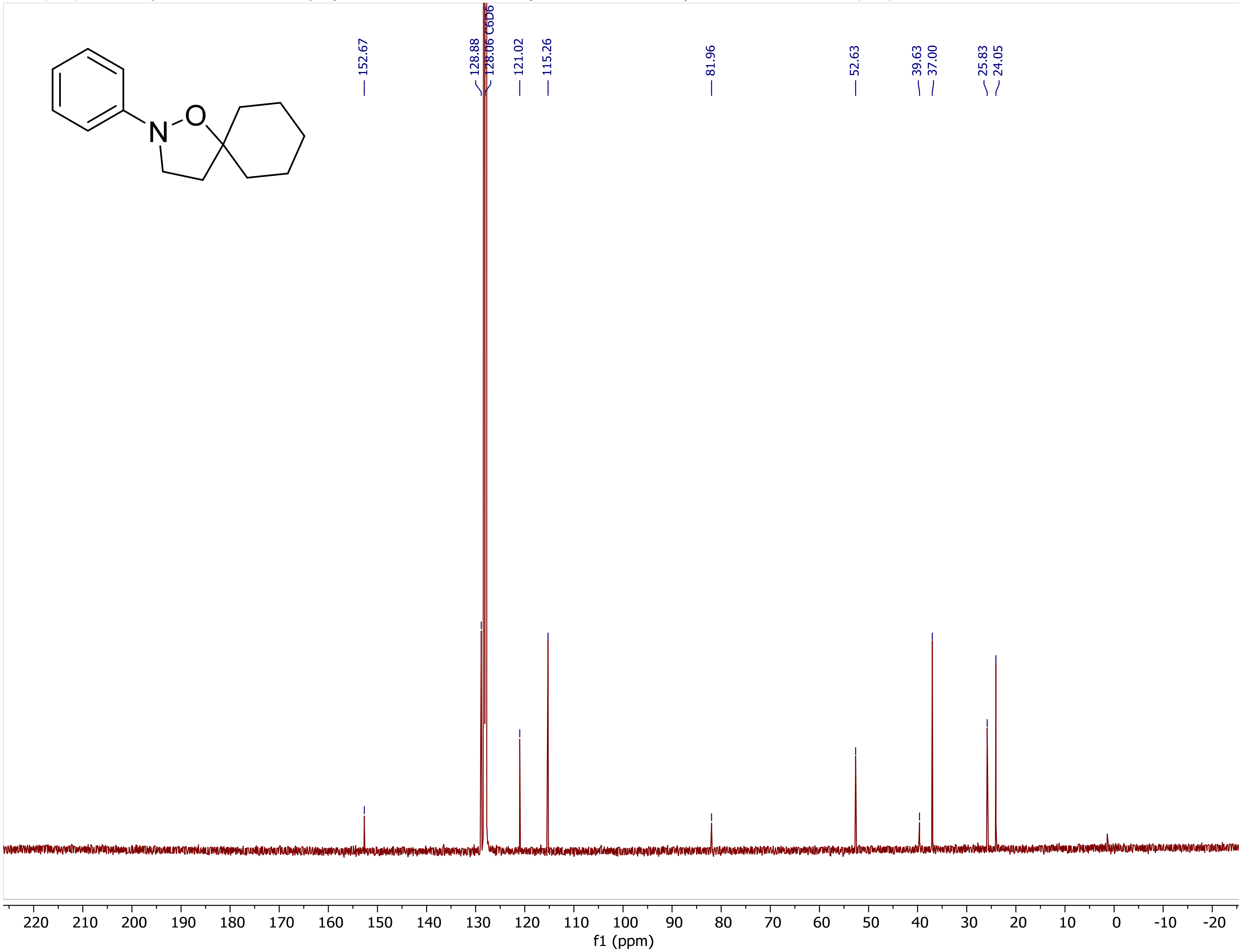

HSQC NMR (500 MHz, C_6D_6) spectrum of 2-Phenyl-1-oxa-2-azaspiro[4.5]decane (**50**)

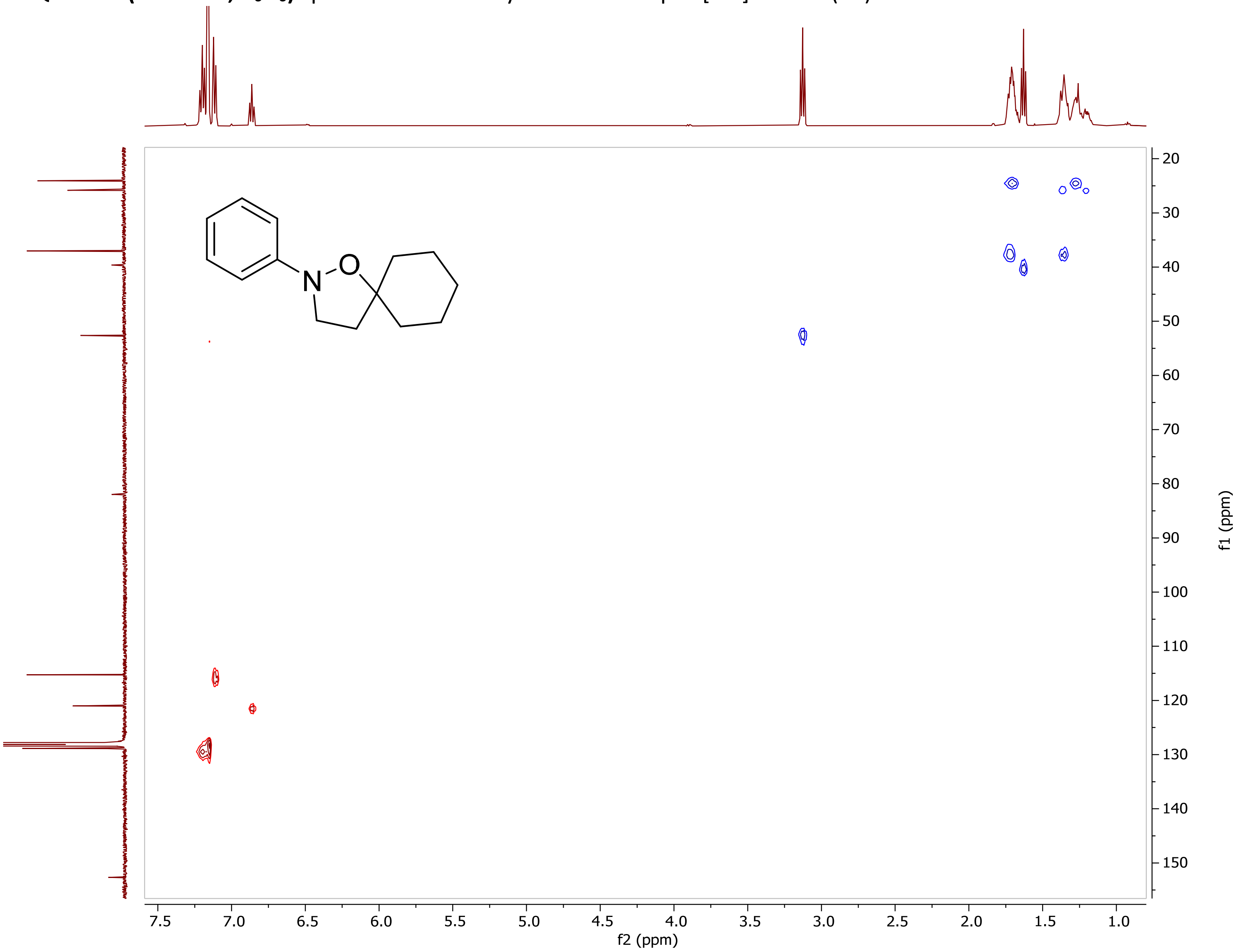

^{1}H NMR (500 MHz, $CDCl_3$) 1-(4-Chlorophenyl)but-3-en-1-ol (**S13**)

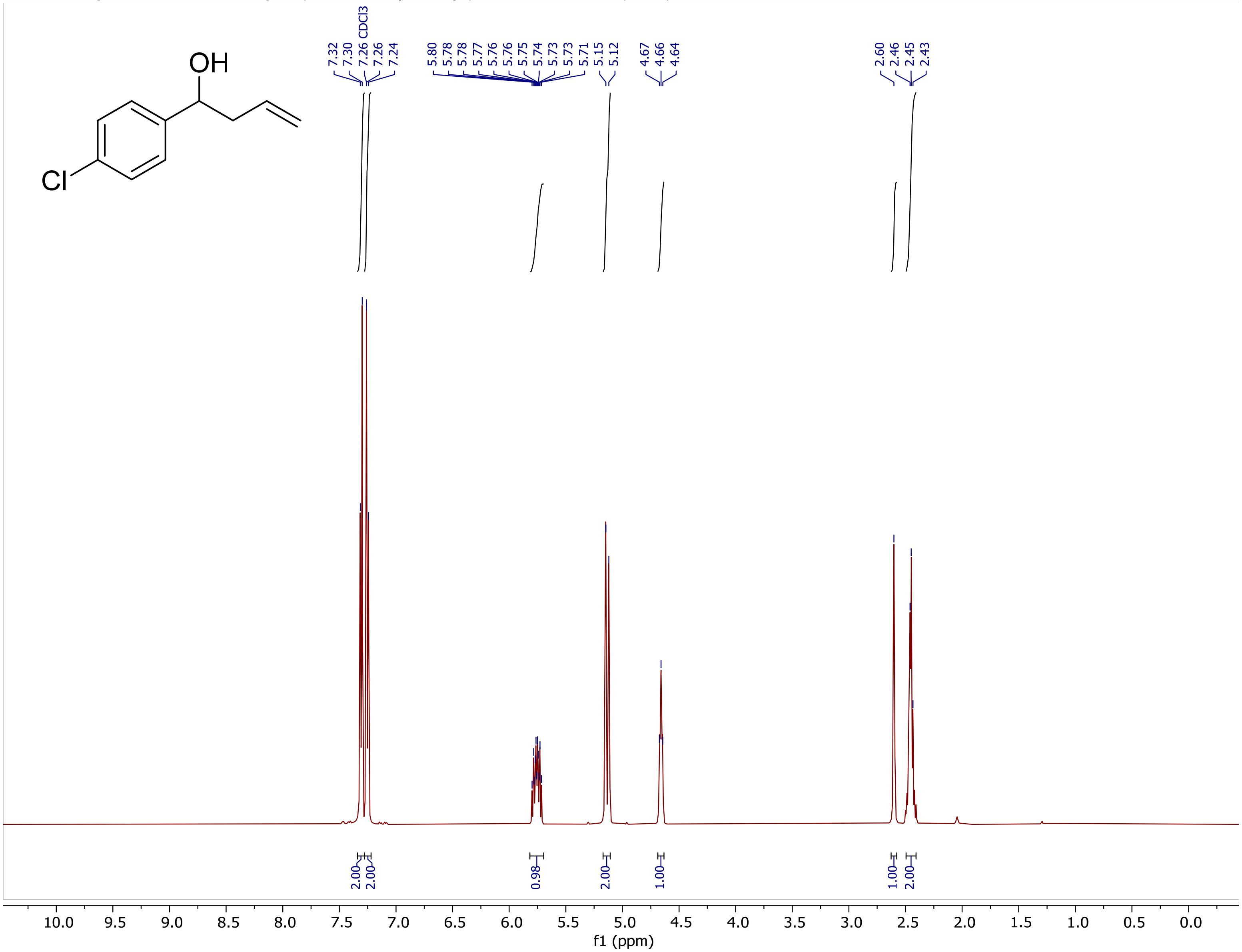

^{13}C {^{1}H} NMR (126 MHz, $CDCl_3$) 1-(4-Chlorophenyl)but-3-en-1-ol (**S13**)

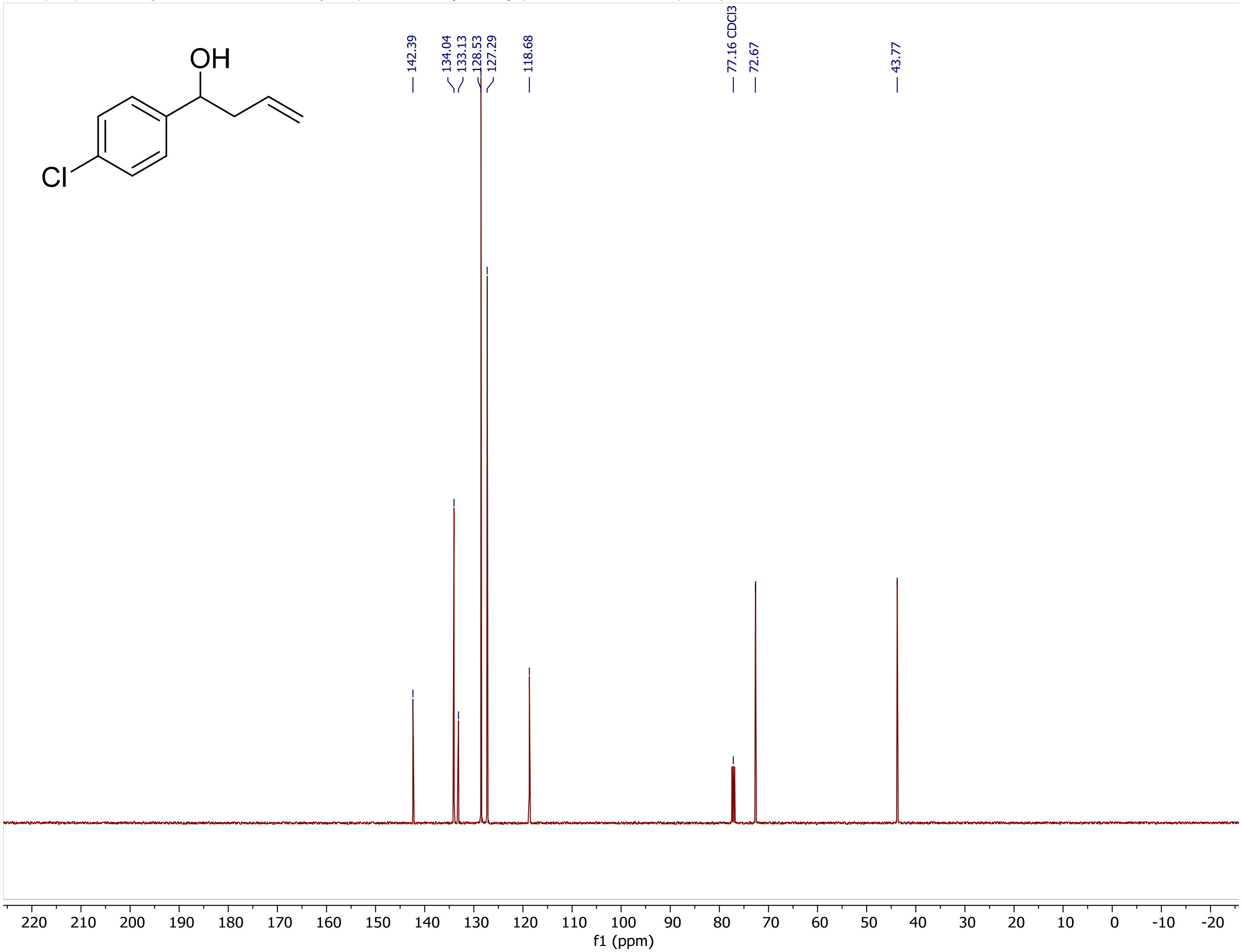

HSQC NMR (500 MHz, $CDCl_3$) 1-(4-Chlorophenyl)but-3-en-1-ol (**S13**)

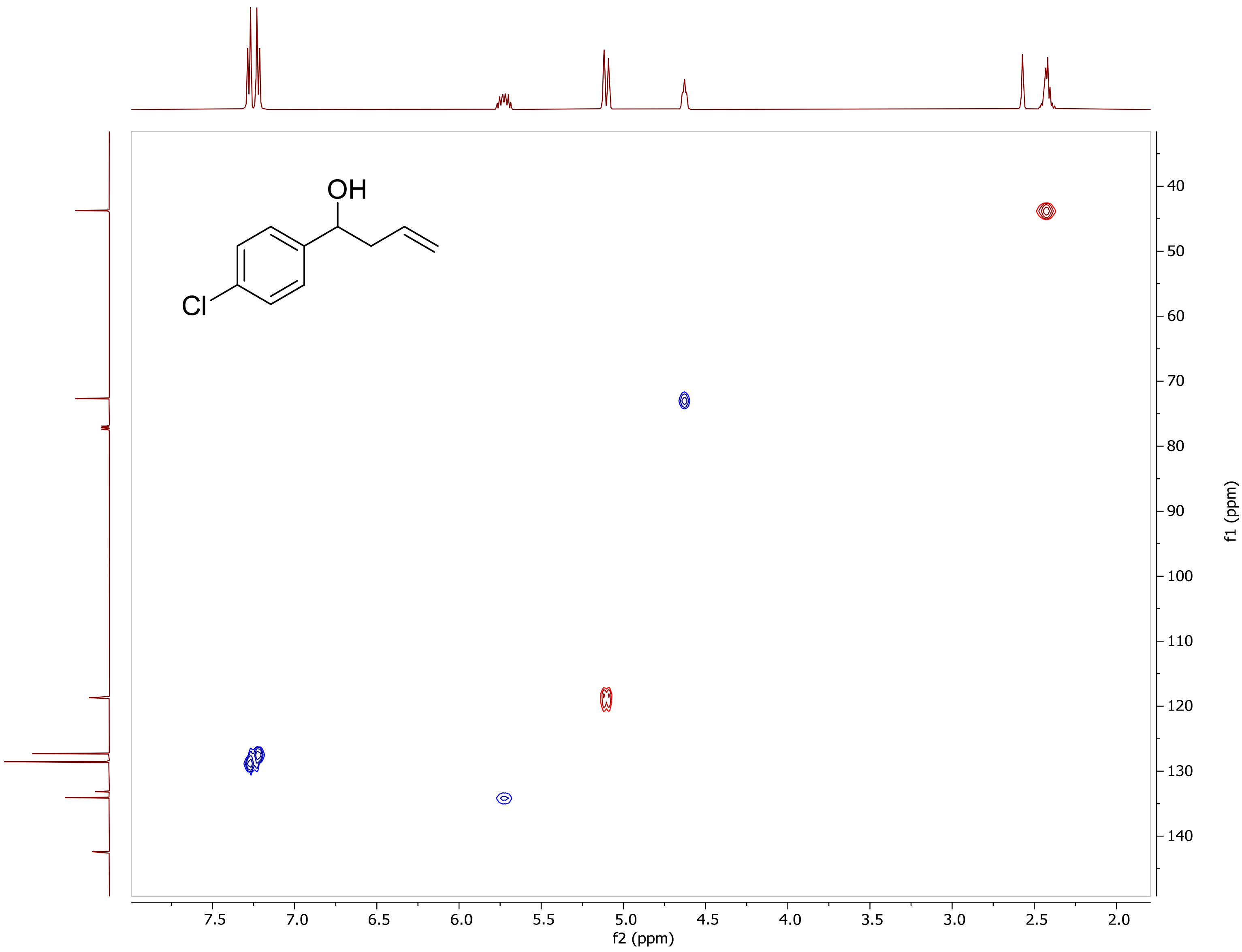

^{1}H NMR (500 MHz, $CDCl_3$) *N*-(1-(4-Chlorophenyl)but-3-en-1-yl)-*N*-(naphthalene-1-ylmethyl)-2-nitro-4-(trifluoromethyl)benzenesulfonamide (**13a**)

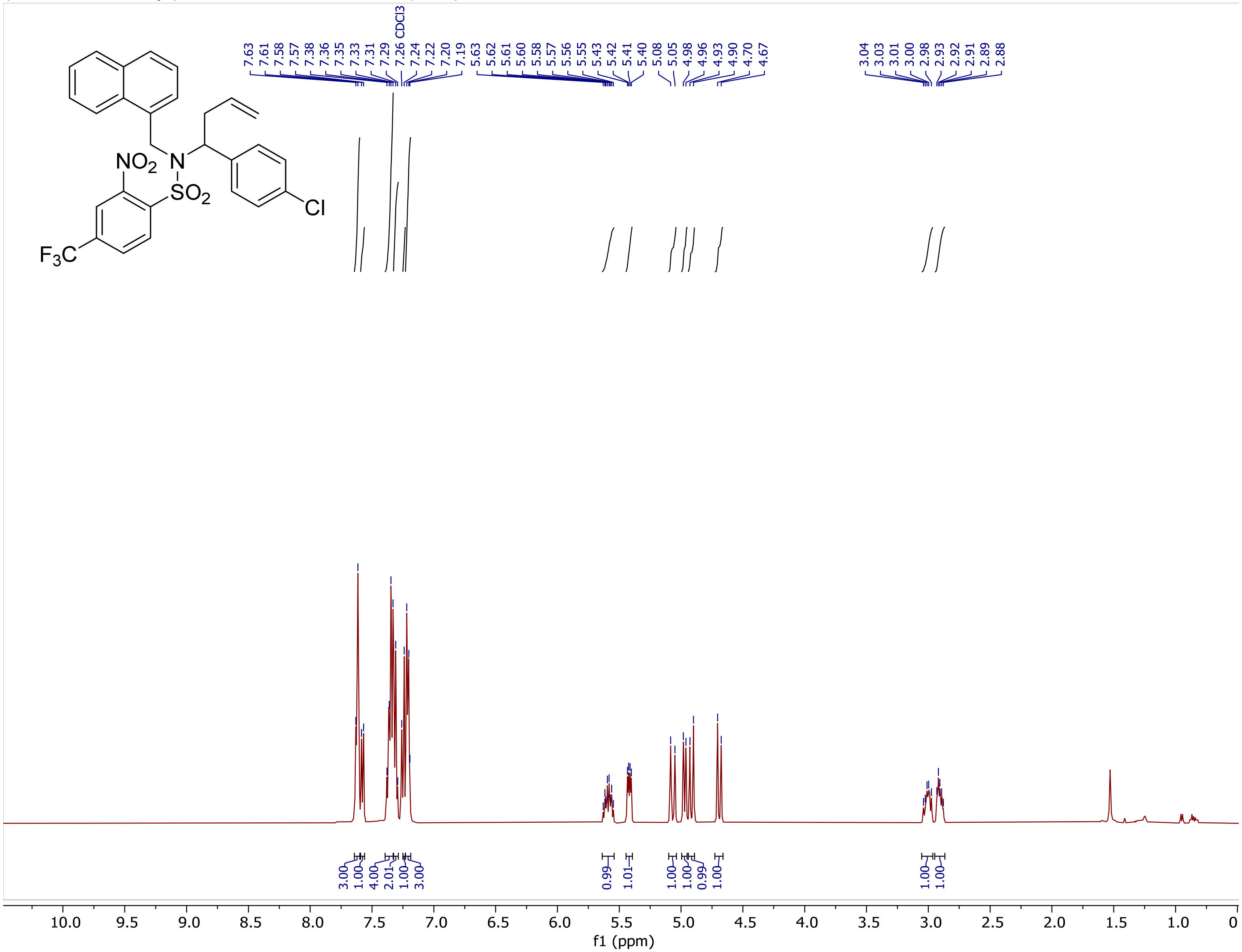

^{13}C {^{1}H, ^{19}F} NMR (126 MHz, $CDCl_3$) *N*-(1-(4-Chlorophenyl)but-3-en-1-yl)-*N*-(naphthalene-1-ylmethyl)-2-nitro-4-(trifluoromethyl)benzenesulfonamide (**13a**)

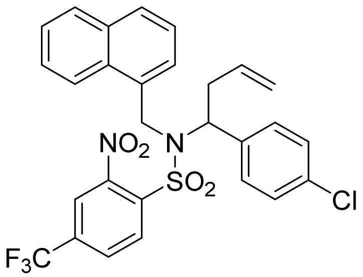

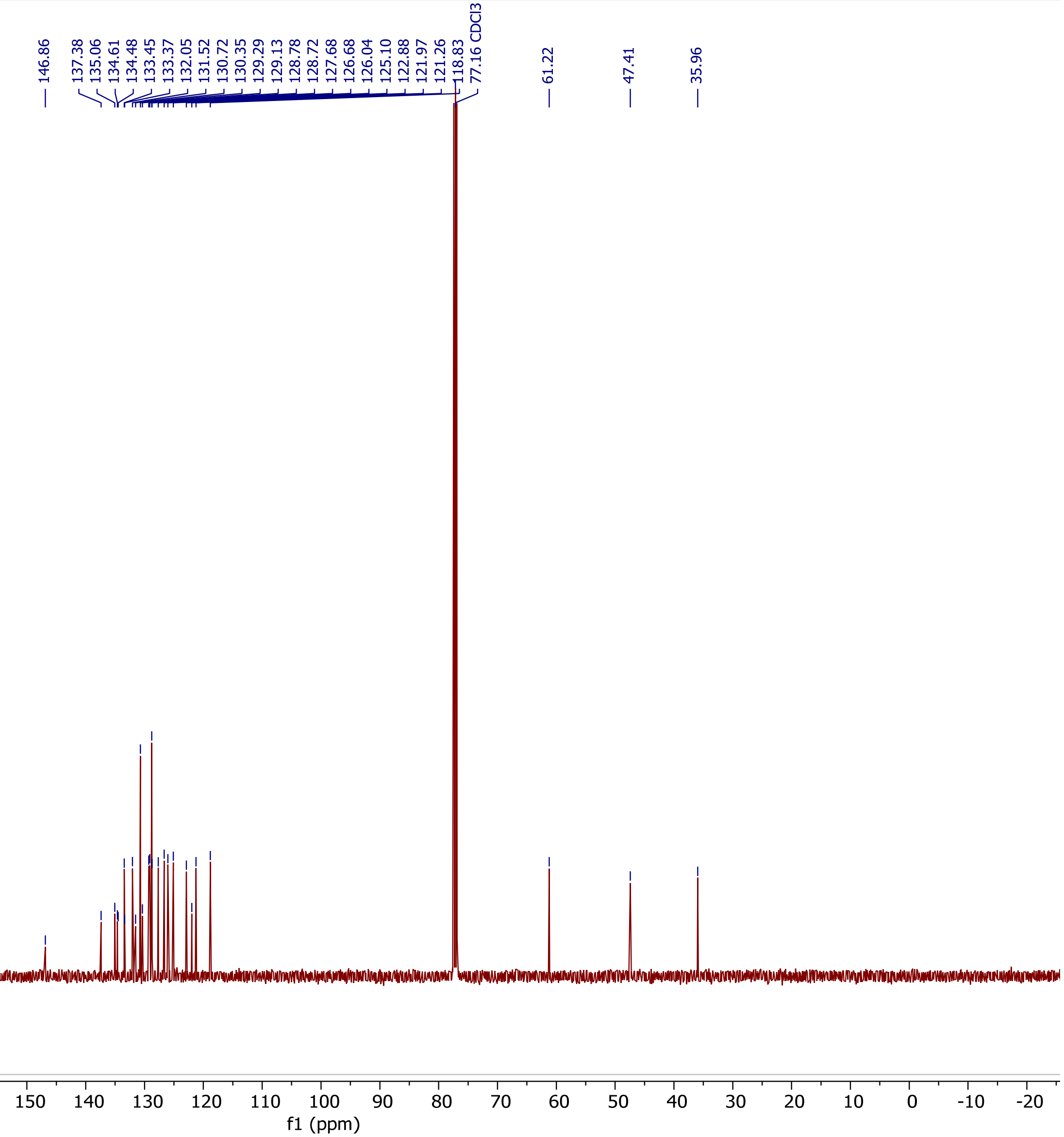

HSQC NMR (500 MHz, CDCl₃) *N*-(1-(4-Chlorophenyl)but-3-en-1-yl)-*N*-(naphthalene-1-ylmethyl)-2-nitro-4-(trifluoromethyl)benzenesulfonamide (**13a**)

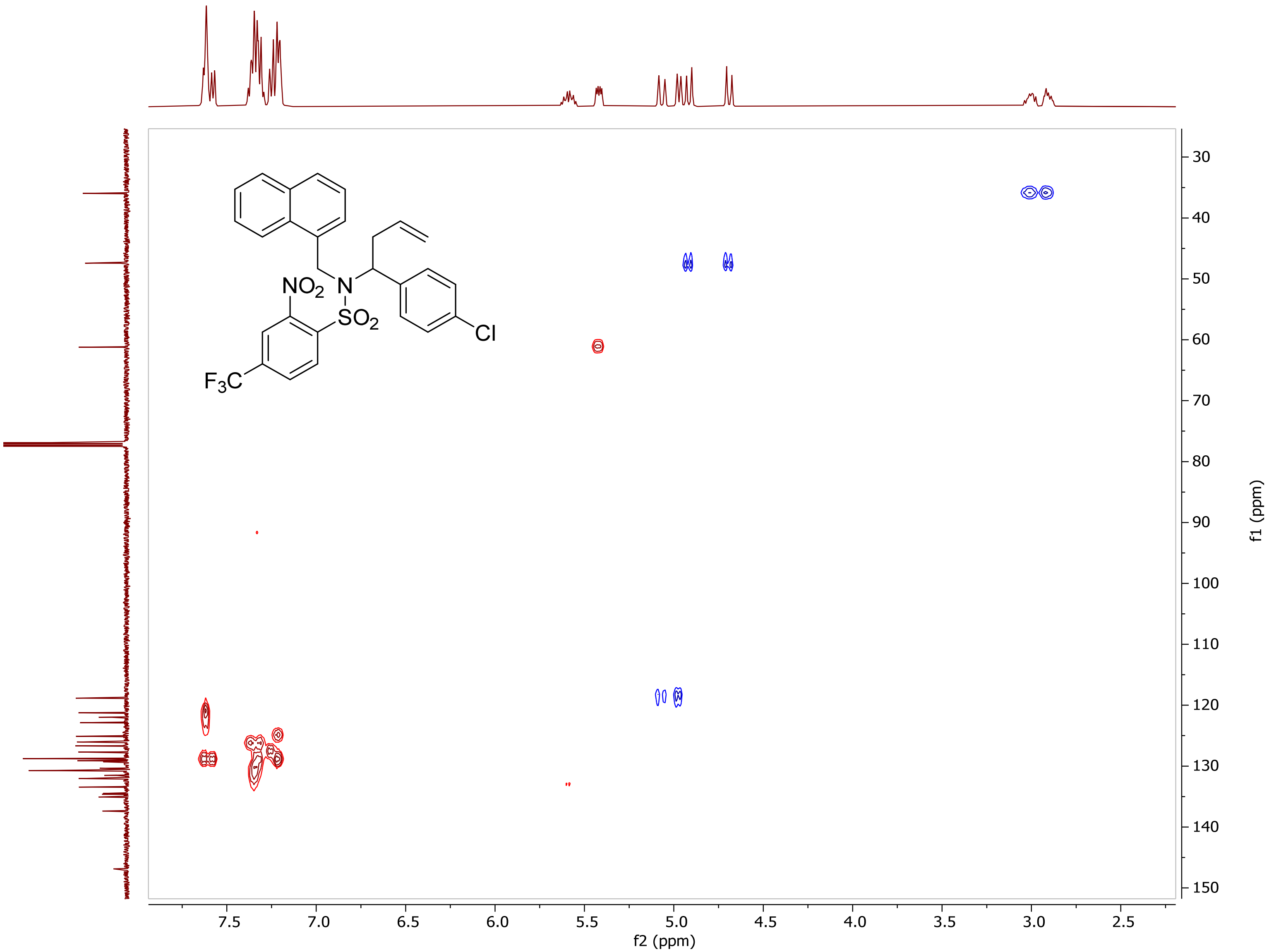

^{1}H NMR (500 MHz, CDCl$_3$) *N*-(1-(4-Chlorophenyl)-3-((triethylsilyl)peroxy)butyl)-*N*-(naphthalene-1-ylmethyl)-2-nitro-4-(trifluoromethyl)benzenesulfonamide (**13b**)

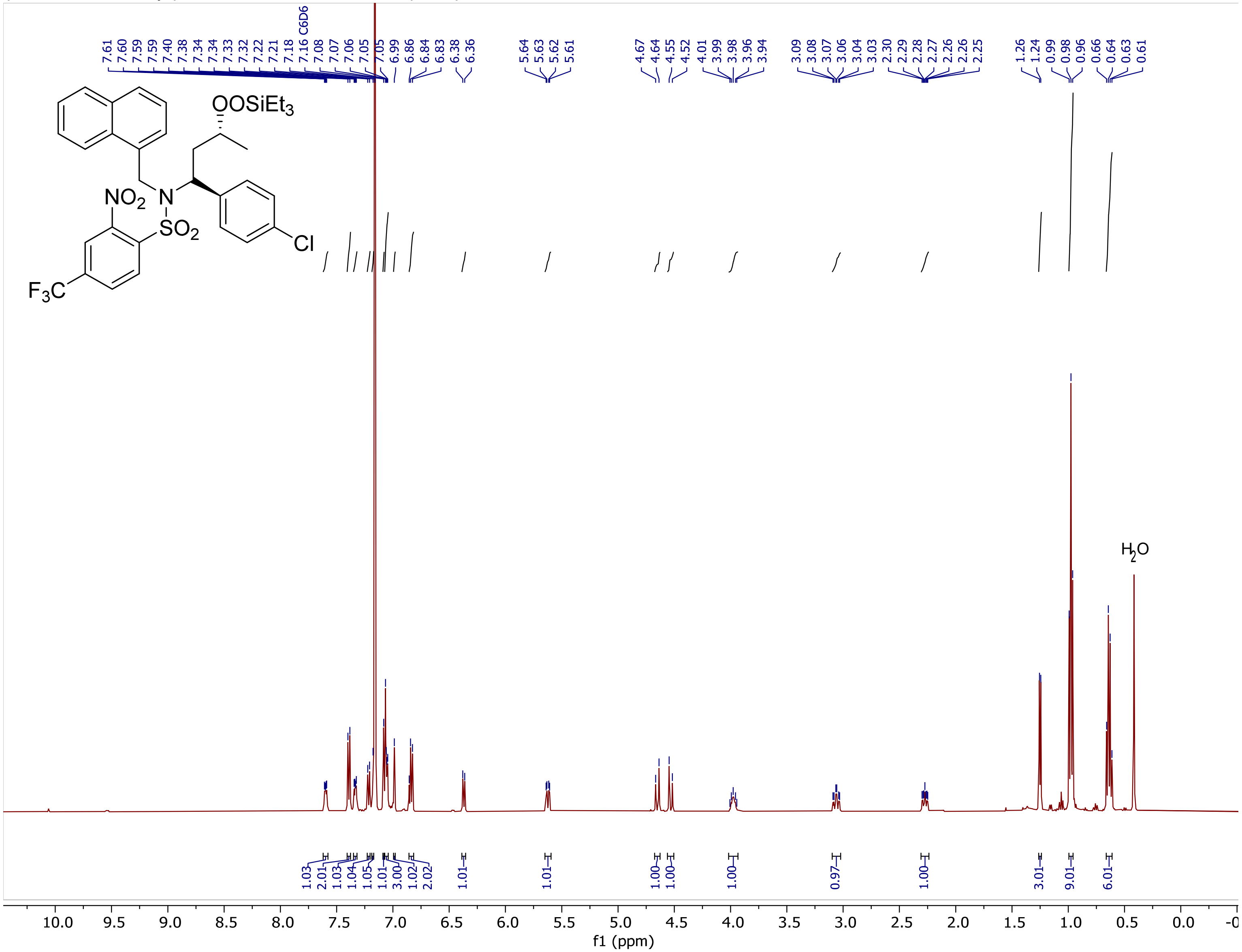

^{13}C {^{1}H, ^{19}F} NMR (126 MHz, $CDCl_3$) *N*-(1-(4-Chlorophenyl)-3-((triethylsilyl)peroxy)butyl)-*N*-(naphthalene-1-ylmethyl)-2-nitro-4-(trifluoromethyl)benzenesulfonamide (**13b**)

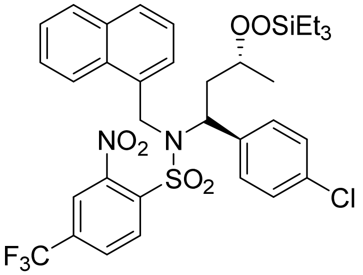

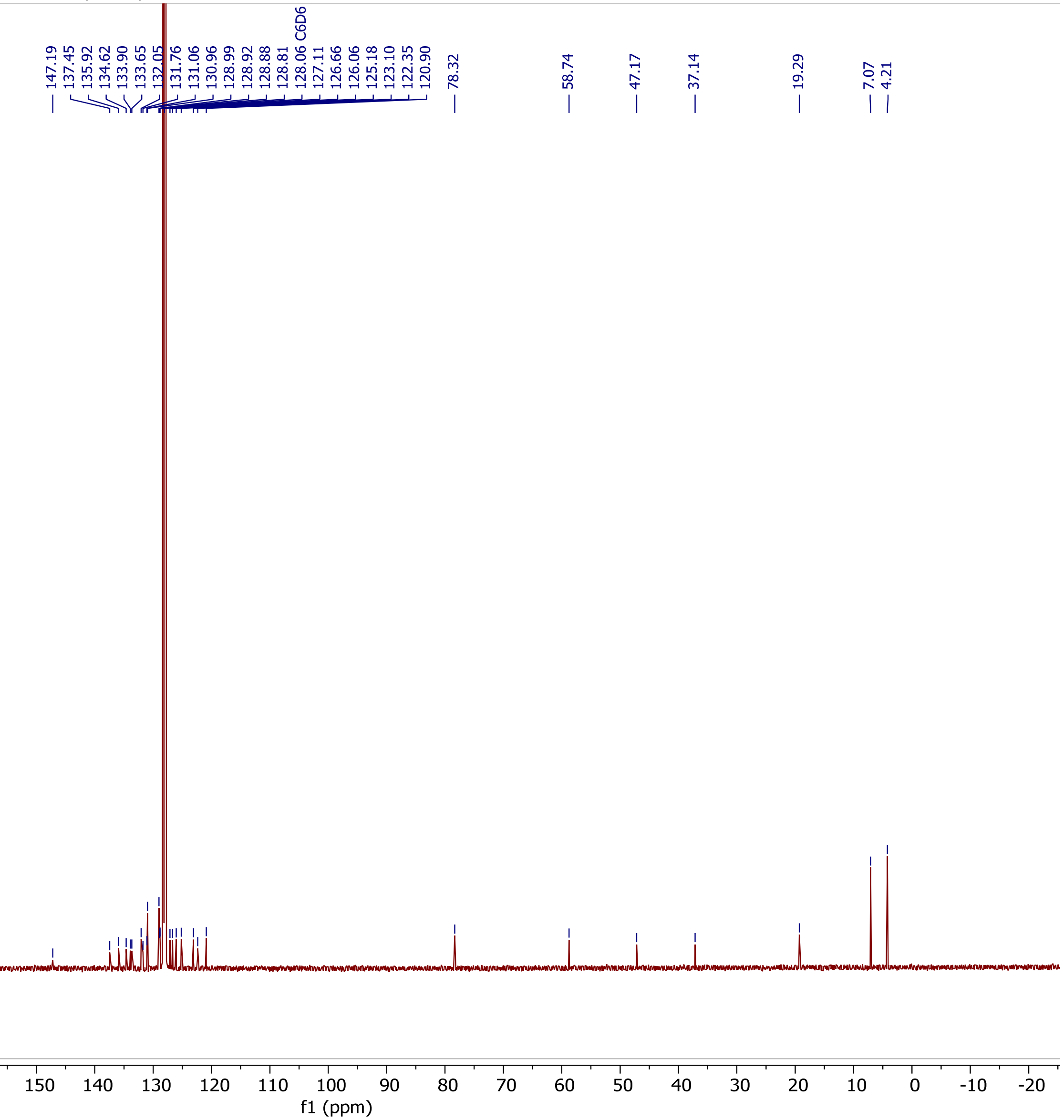

^{1}H NMR (500 MHz, C_6D_6) *N*-(3-((tert-butyldiphenylsilyl)peroxy)-1-(4-Chlorophenyl)butyl)-*N*-(naphthalene-1-ylmethyl)-2-nitro-4-(trifluoromethyl)benzenesulfonamide (**51**)

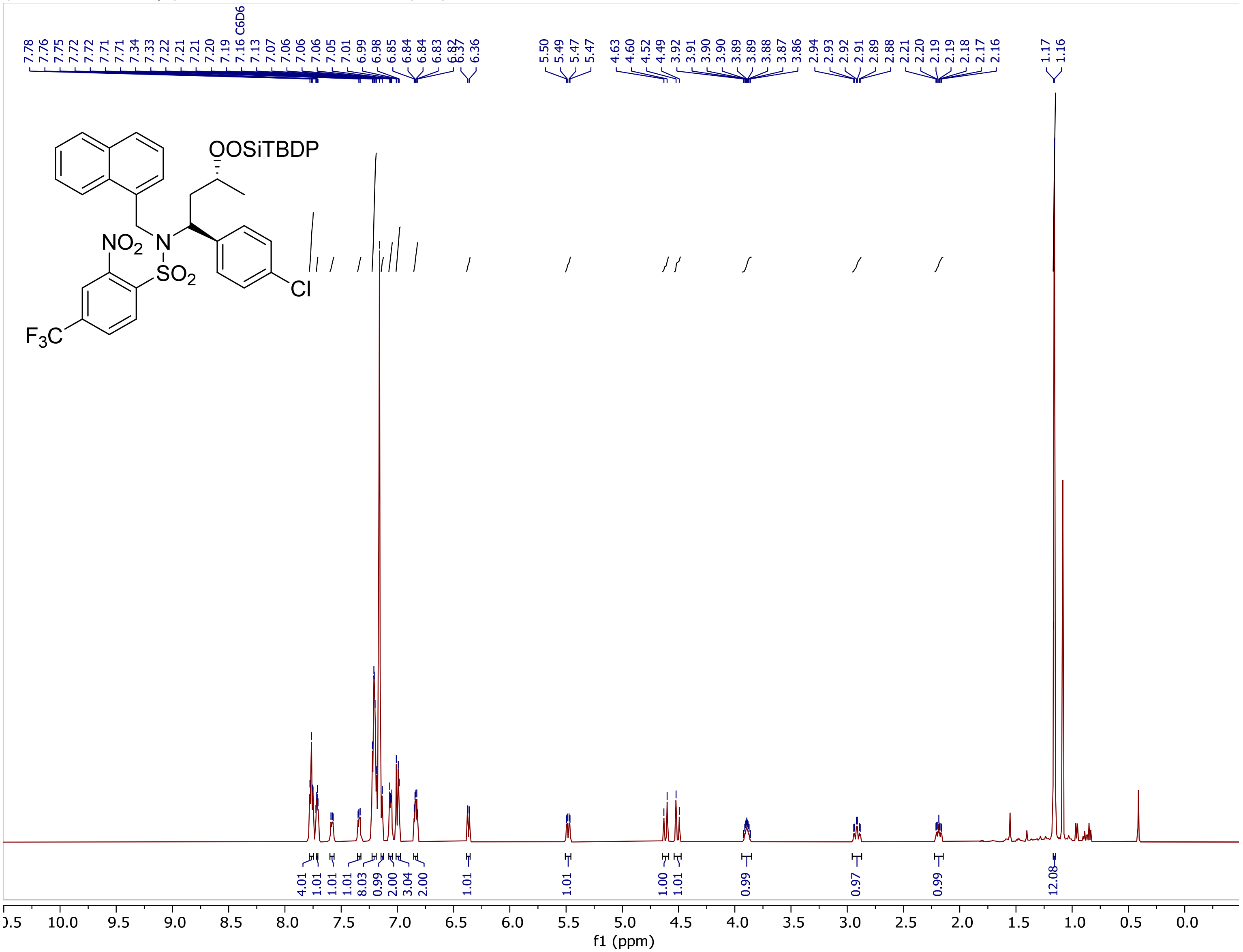

^{13}C {^{1}H, ^{19}F} NMR (126 MHz, C_6D_6) *N*-(3-((tert-butyldiphenylsilyl)peroxy)-1-(4-Chlorophenyl)butyl)-*N*-(naphthalene-1-ylmethyl)-2-nitro-4-(trifluoromethyl)benzenesulfonamide (**51**)

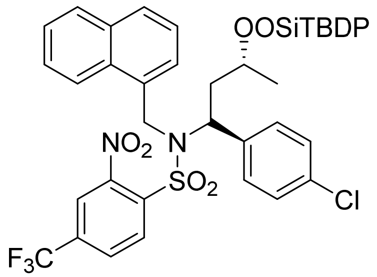

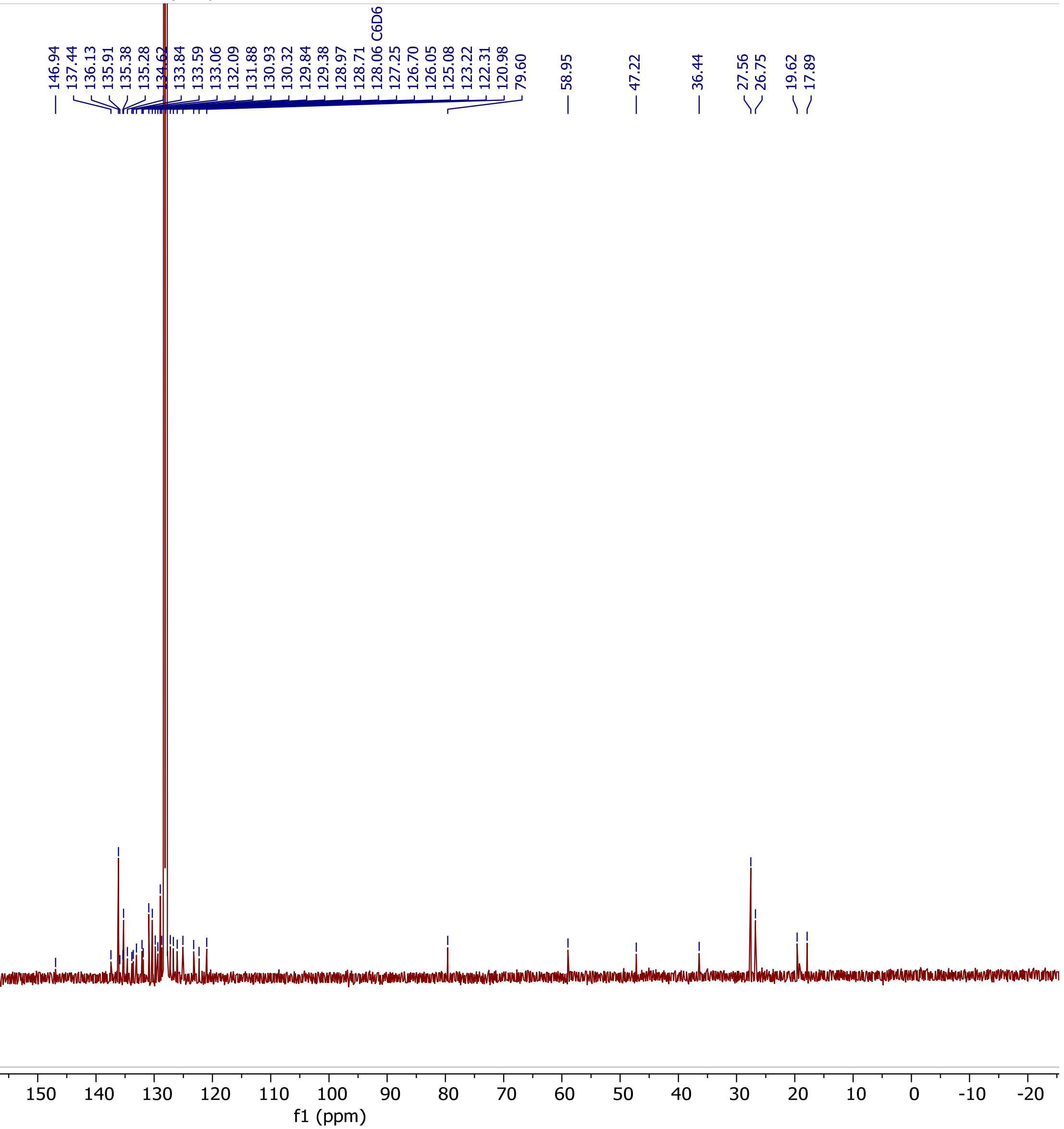

^{1}H NMR (500 MHz, C_6D_6) 3-(4-Chlorophenyl)-5-methyl-2-(naphthalene-1-ylmethyl)isoxazolidine (**53**)

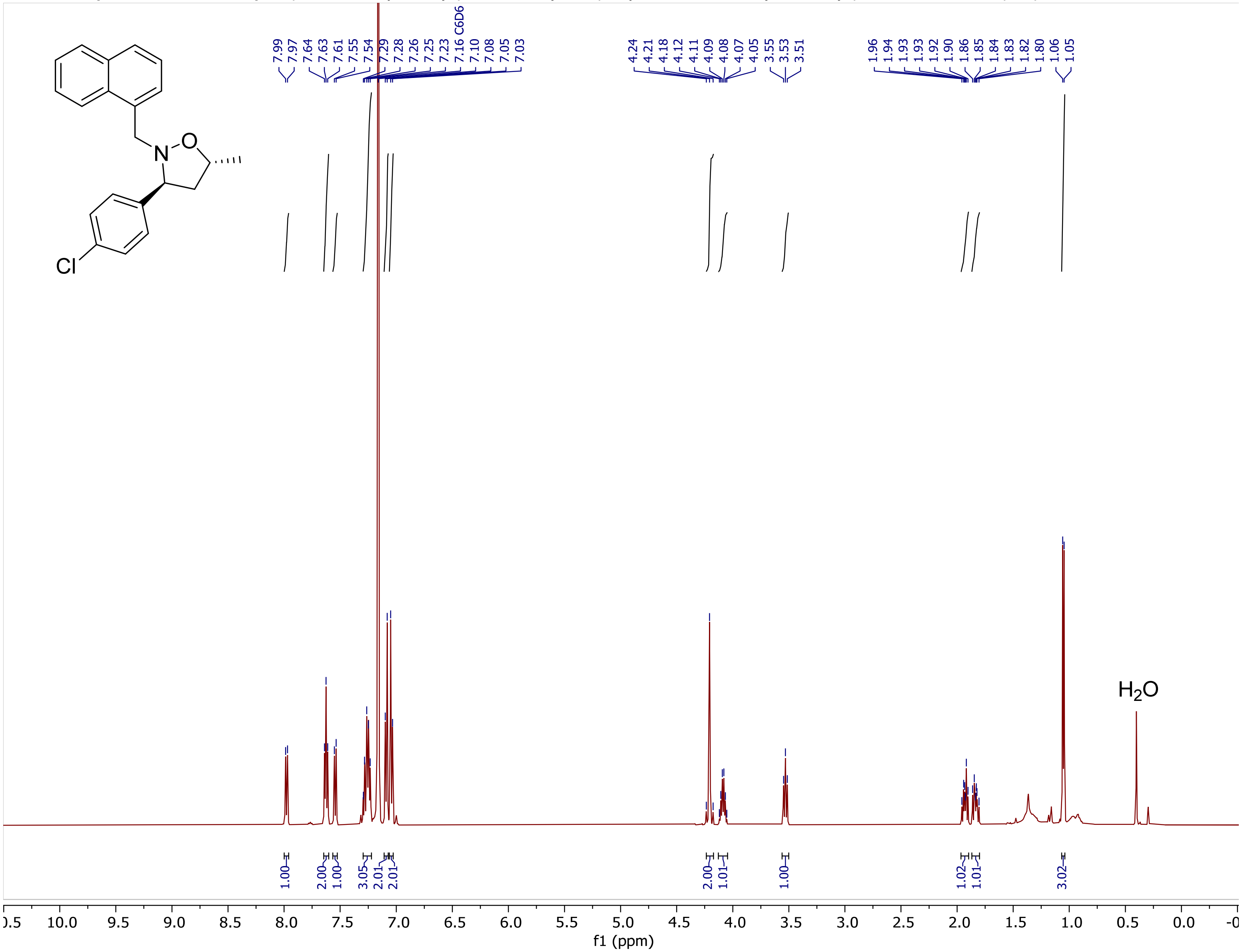

^{13}C {^{1}H} NMR (126 MHz, C_6D_6) 3-(4-Chlorophenyl)-5-methyl-2-(naphthalene-1-ylmethyl)isoxazolidine (**53**)

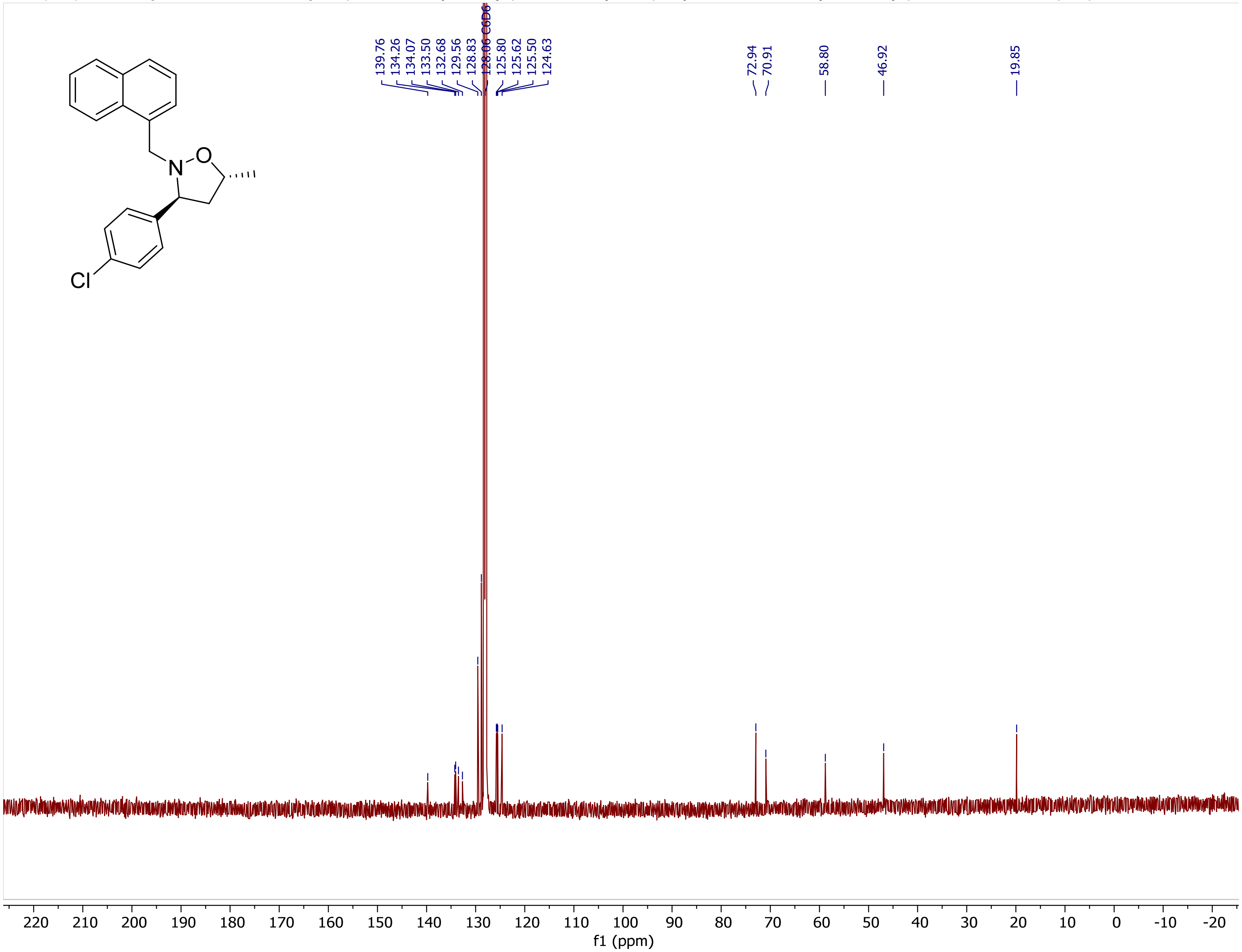

HSQC NMR (500 MHz, C_6D_6) 3-(4-Chlorophenyl)-5-methyl-2-(naphthalene-1-ylmethyl)isoxazolidine (**53**)

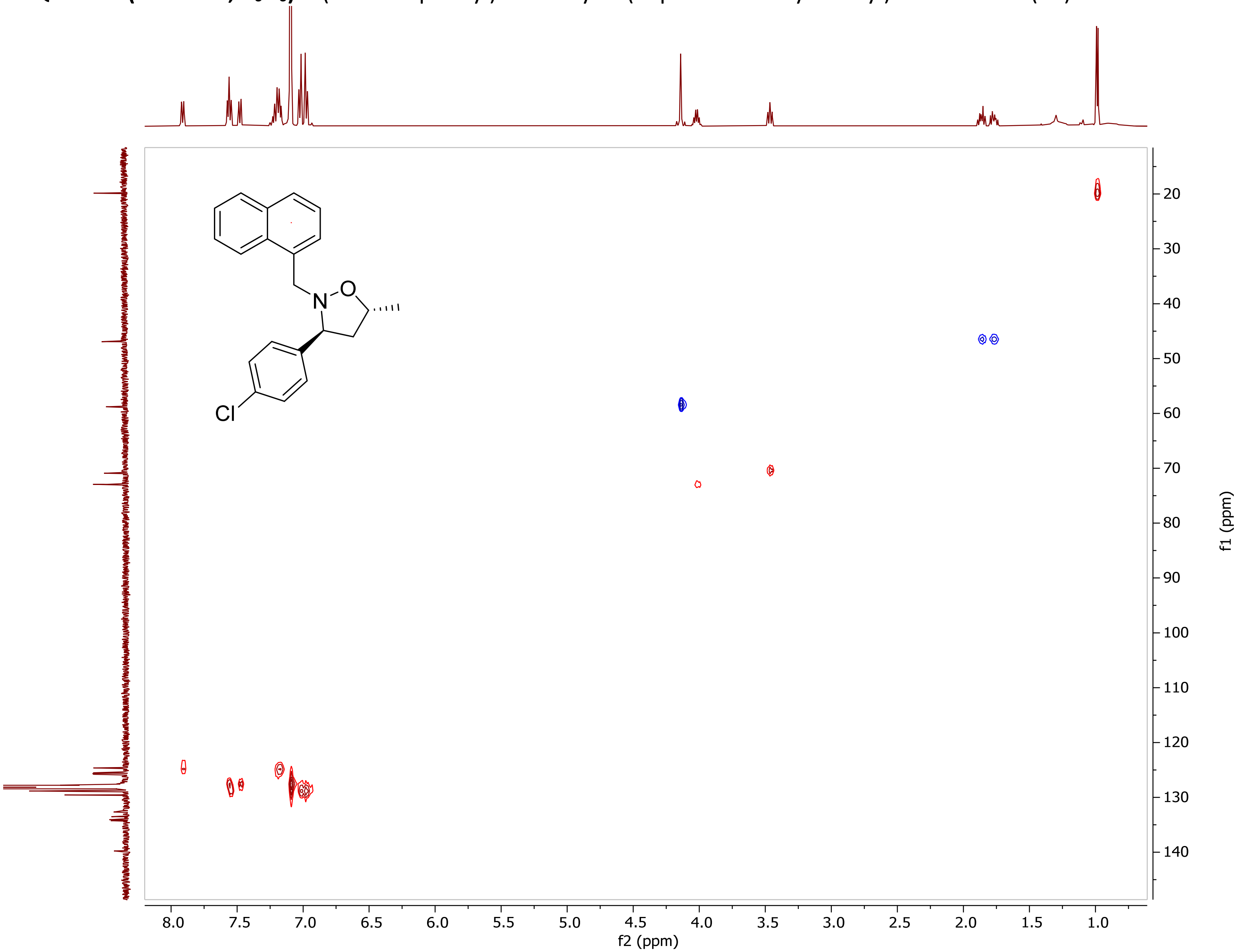

1D NOESY NMR (500 MHz, C_6D_6) 3-(4-Chlorophenyl)-5-methyl-2-(naphthalene-1-ylmethyl)isoxazolidine (**53**)

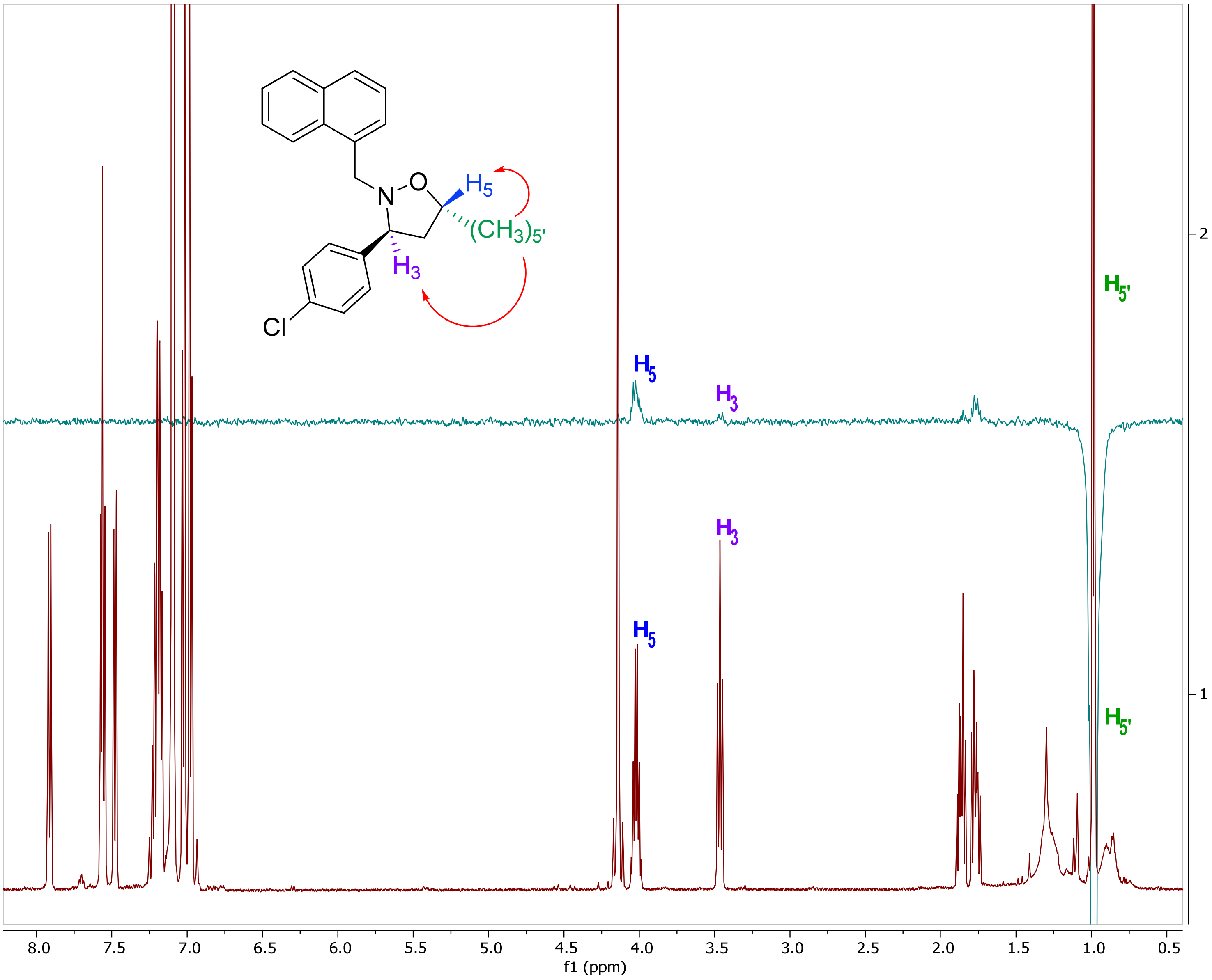

1D NOESY NMR (500 MHz, C_6D_6) 3-(4-Chlorophenyl)-5-methyl-2-(naphthalene-1-ylmethyl)isoxazolidine (**53**)

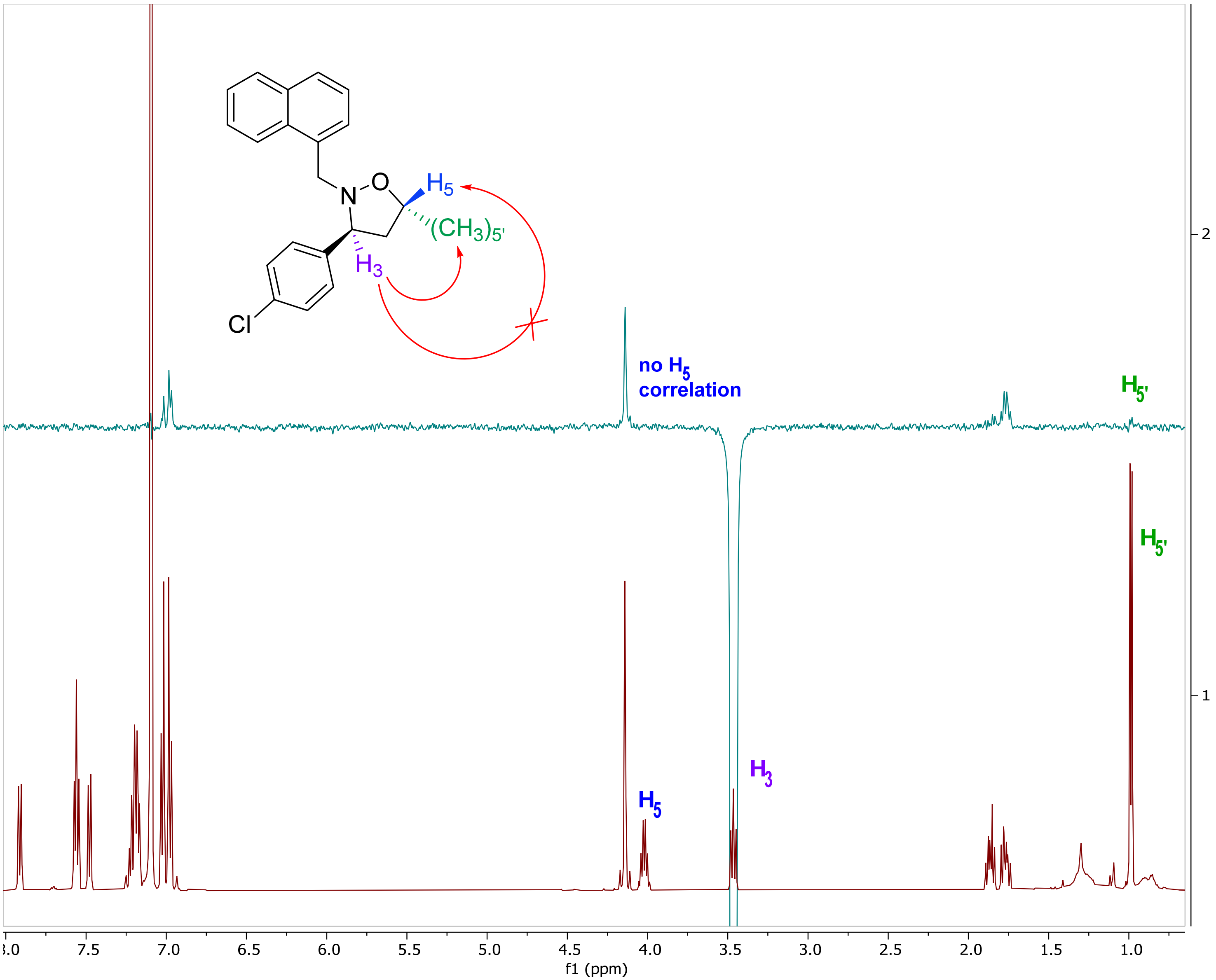

^{1}H NMR (500 MHz, $CDCl_3$) *N*-(1-(4-Chlorophenyl)-3-((triethylsilyl)peroxy)butyl)-*N*-(naphthalene-1-ylmethyl)-2-nitro-4-(trifluoromethyl)benzenesulfonamide (**13c**)

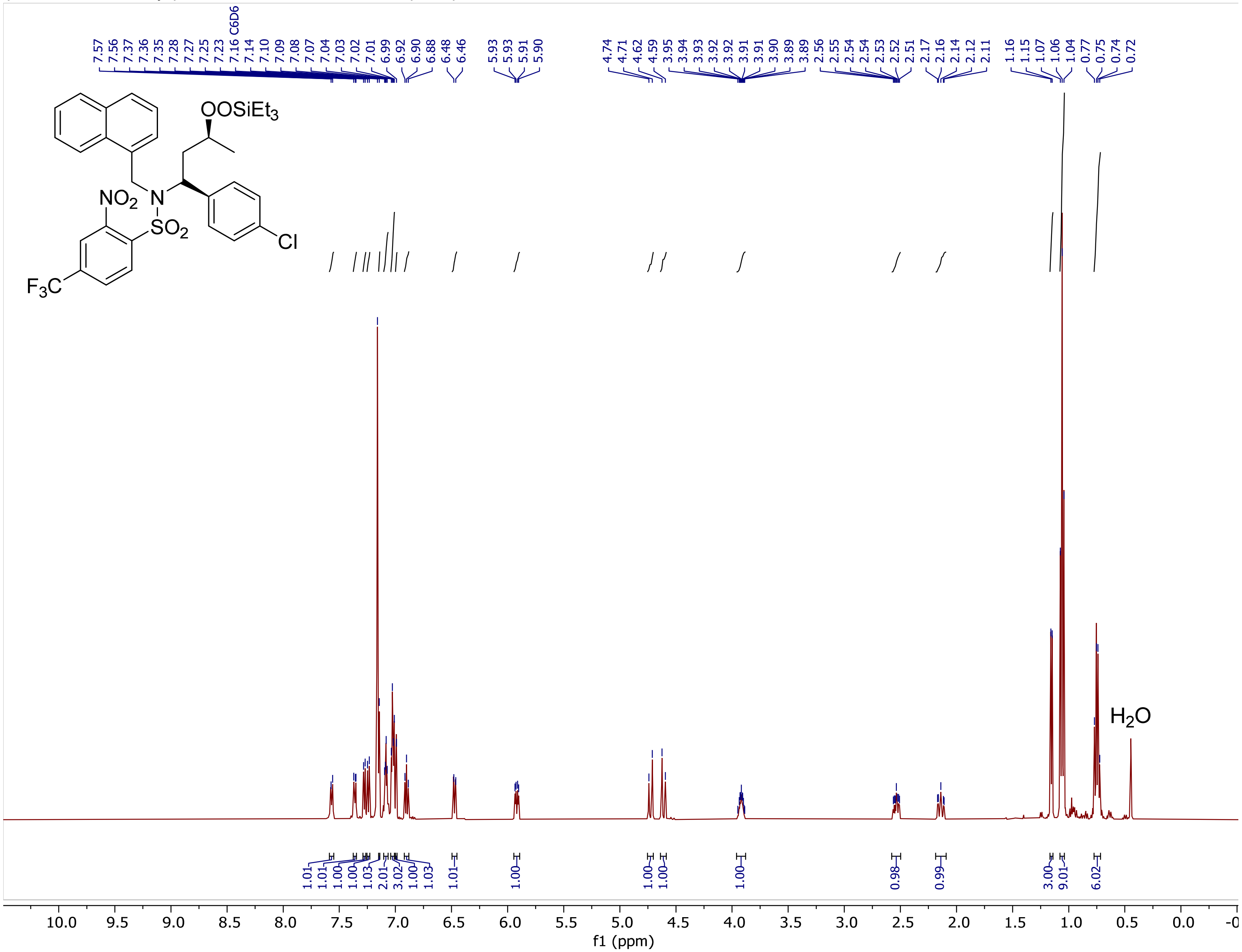

$^{13}C\ \{^{1}H,\ ^{19}F\}$ NMR (126 MHz, $CDCl_3$) *N*-(1-(4-Chlorophenyl)-3-((triethylsilyl)peroxy)butyl)-*N*-(naphthalene-1-ylmethyl)-2-nitro-4-(trifluoromethyl)benzenesulfonamide (**13c**)

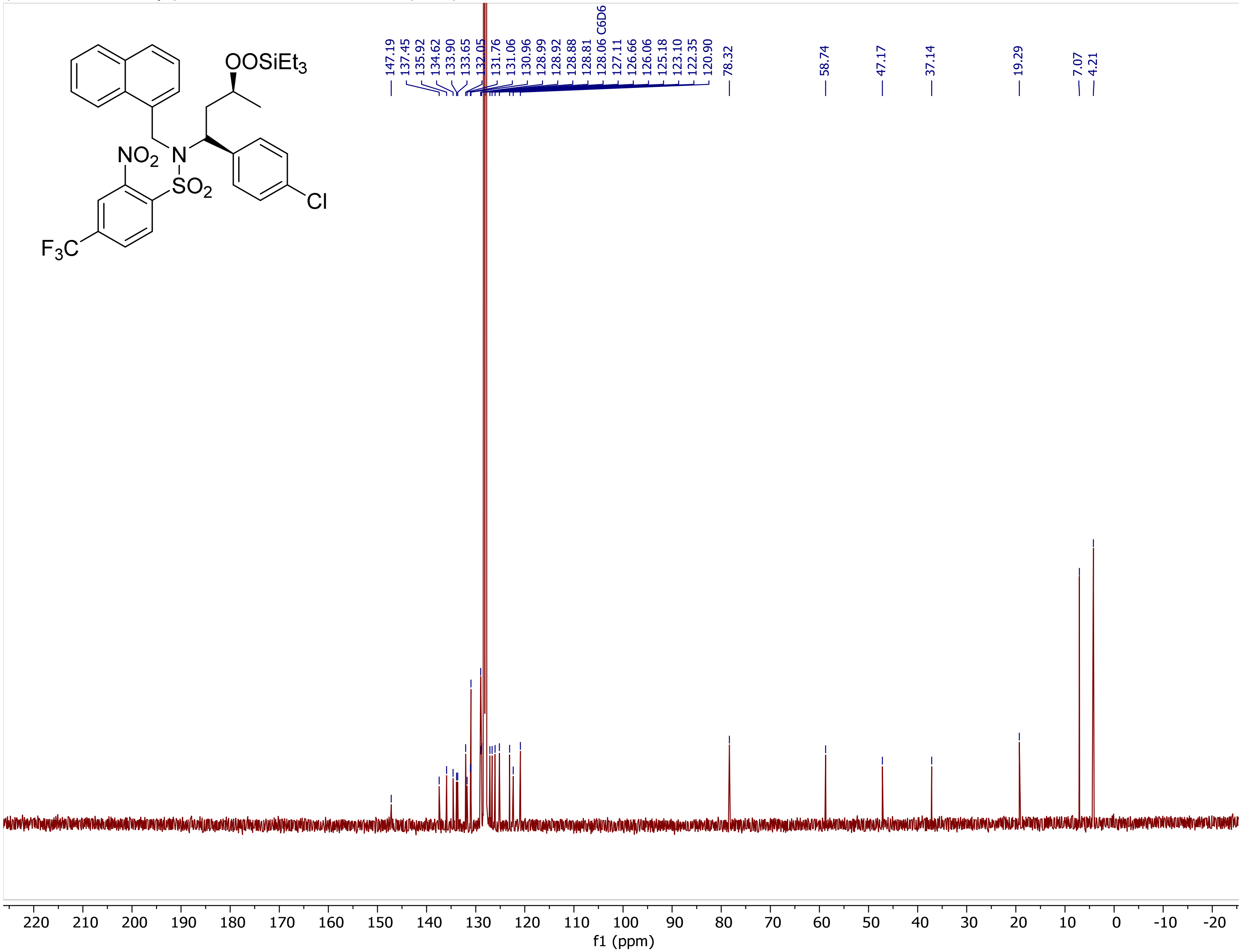

HSQC NMR (500 MHz, $CDCl_3$) *N*-(1-(4-Chlorophenyl)-3-((triethylsilyl)peroxy)butyl)-*N*-(naphthalene-1-ylmethyl)-2-nitro-4-(trifluoromethyl)benzenesulfonamide (**13c**)

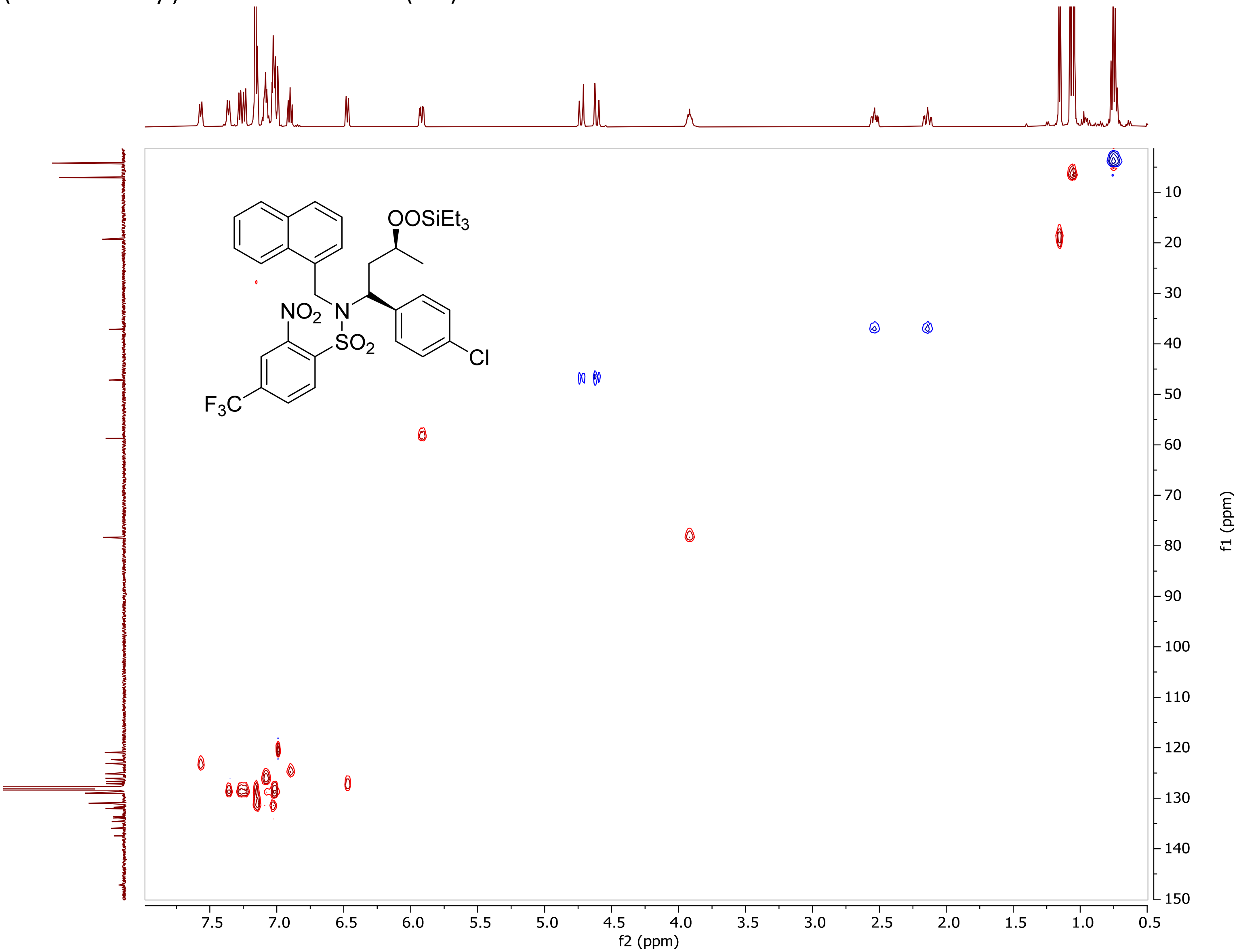

^{1}H NMR (500 MHz, C_6D_6) *N*-(3-((tert-butyldiphenylsilyl)peroxy)-1-(4-Chlorophenyl)butyl)-*N*-(naphthalene-1-ylmethyl)-2-nitro-4-(trifluoromethyl)benzenesulfonamide (**52**)

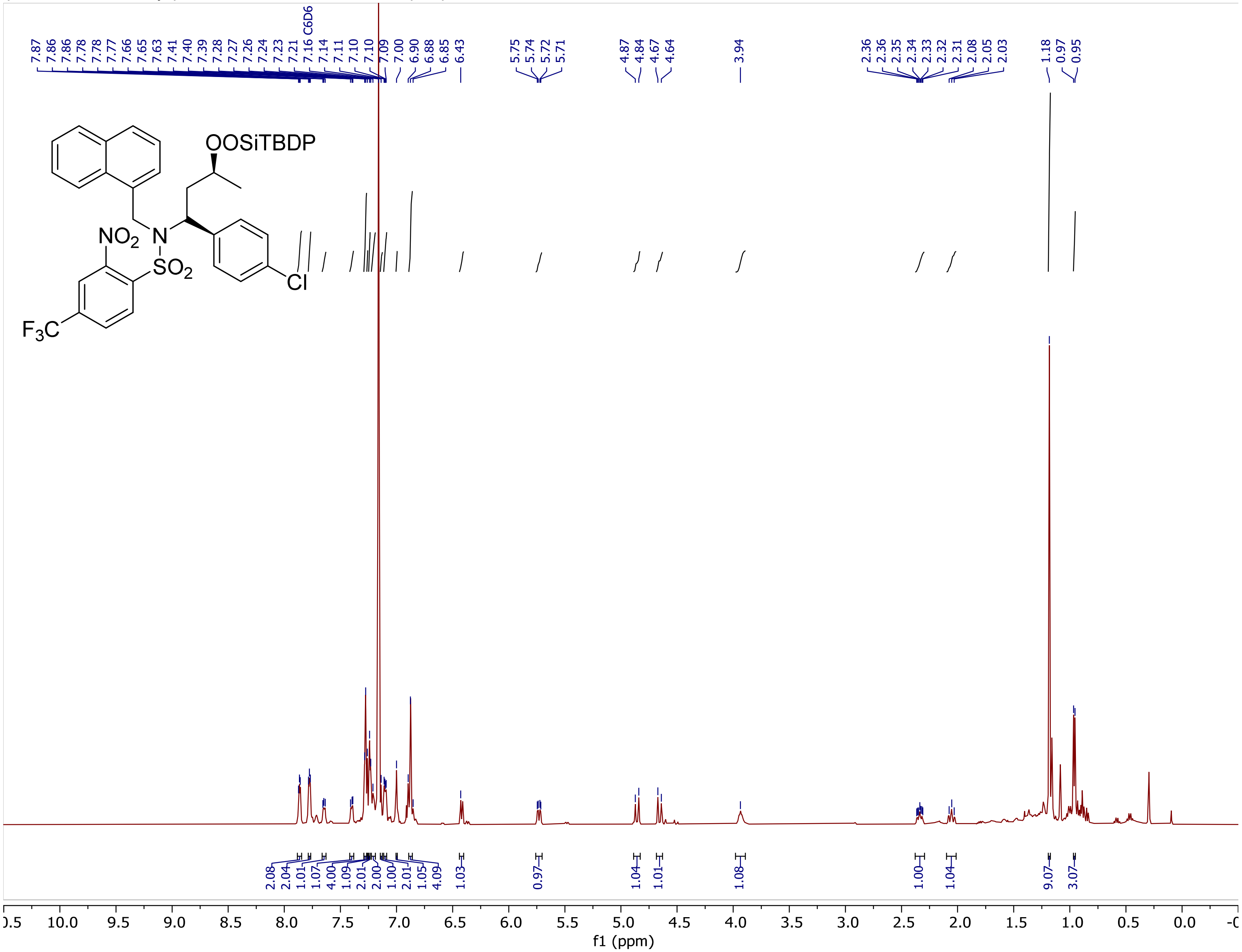

^{13}C {^{1}H, ^{19}F} NMR (126 MHz, C_6D_6) *N*-(3-((tert-butyldiphenylsilyl)peroxy)-1-(4-Chlorophenyl)butyl)-*N*-(naphthalene-1-ylmethyl)-2-nitro-4-(trifluoromethyl)benzenesulfonamide (**52**)

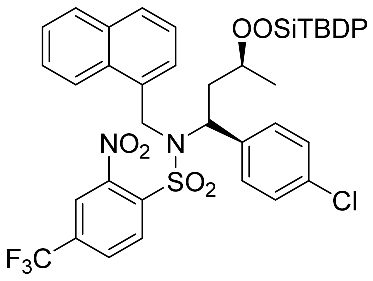

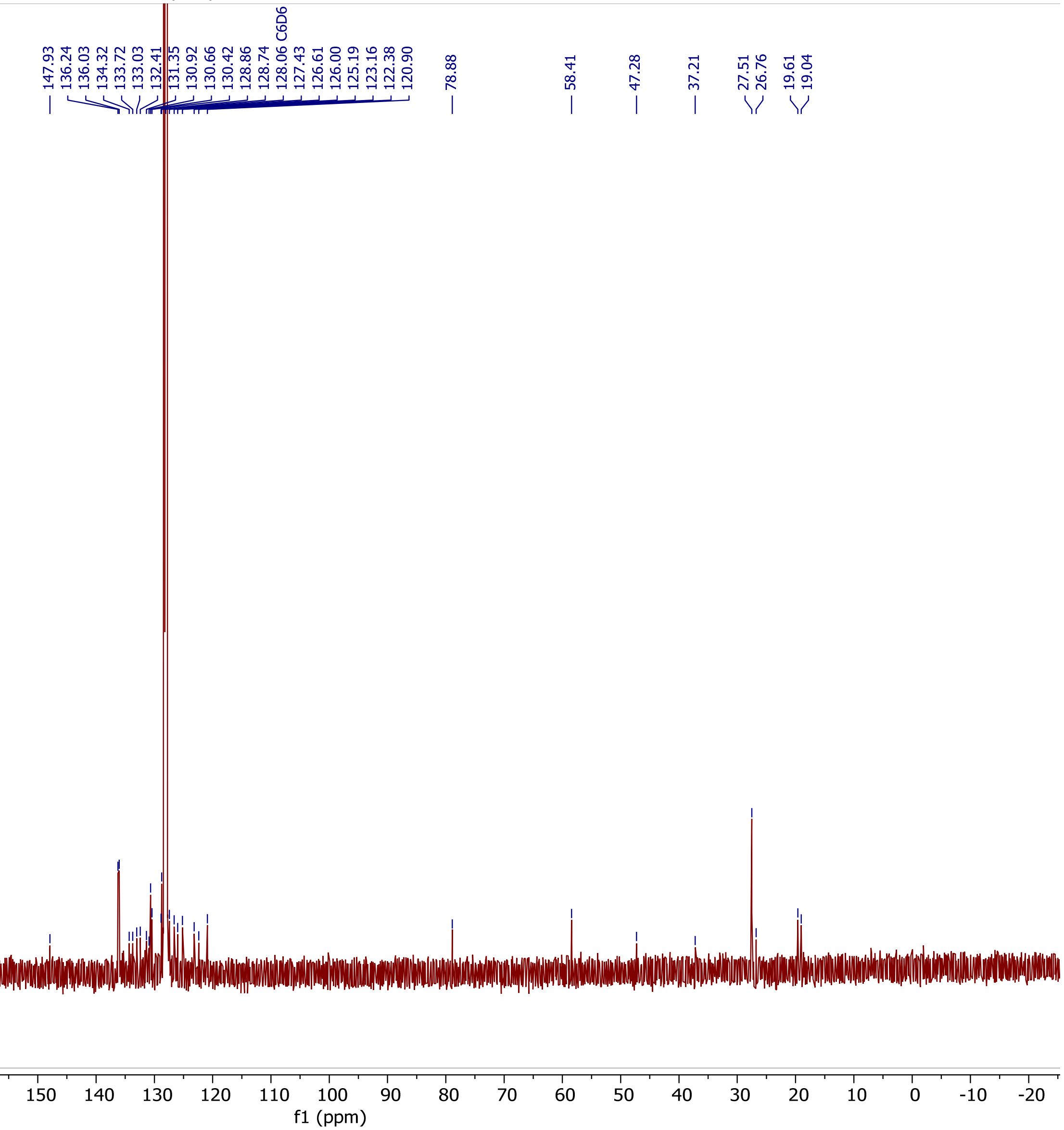

^{1}H NMR (500 MHz, C_6D_6) 3-(4-Chlorophenyl)-5-methyl-2-(naphthalene-1-ylmethyl)isoxazolidine (**54**)

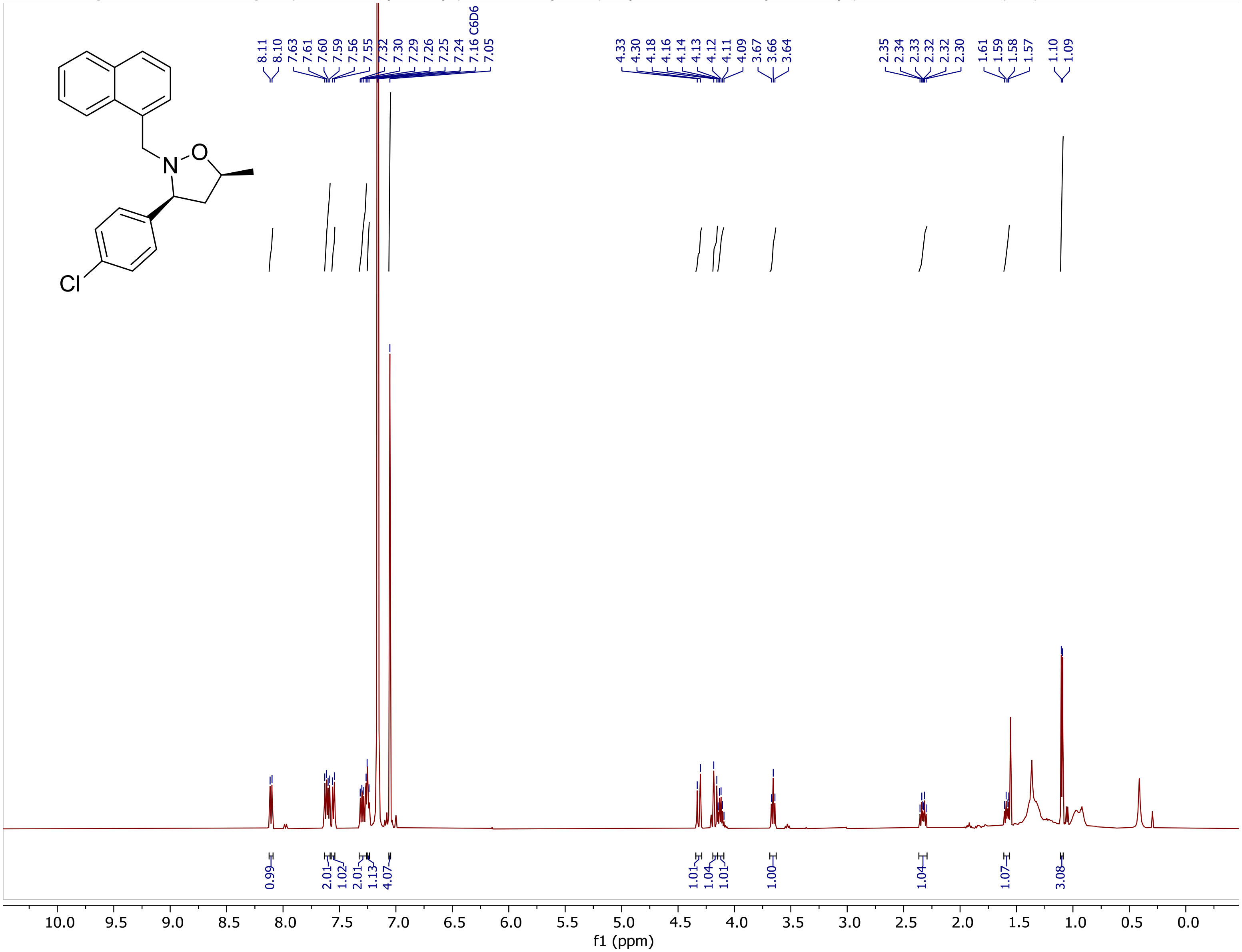

^{13}C {^{1}H} NMR (126 MHz, C_6D_6) 3-(4-Chlorophenyl)-5-methyl-2-(naphthalene-1-ylmethyl)isoxazolidine (**54**)

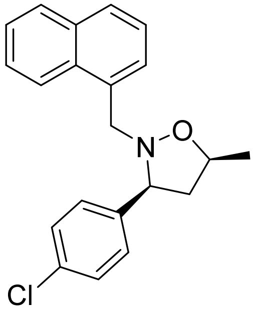

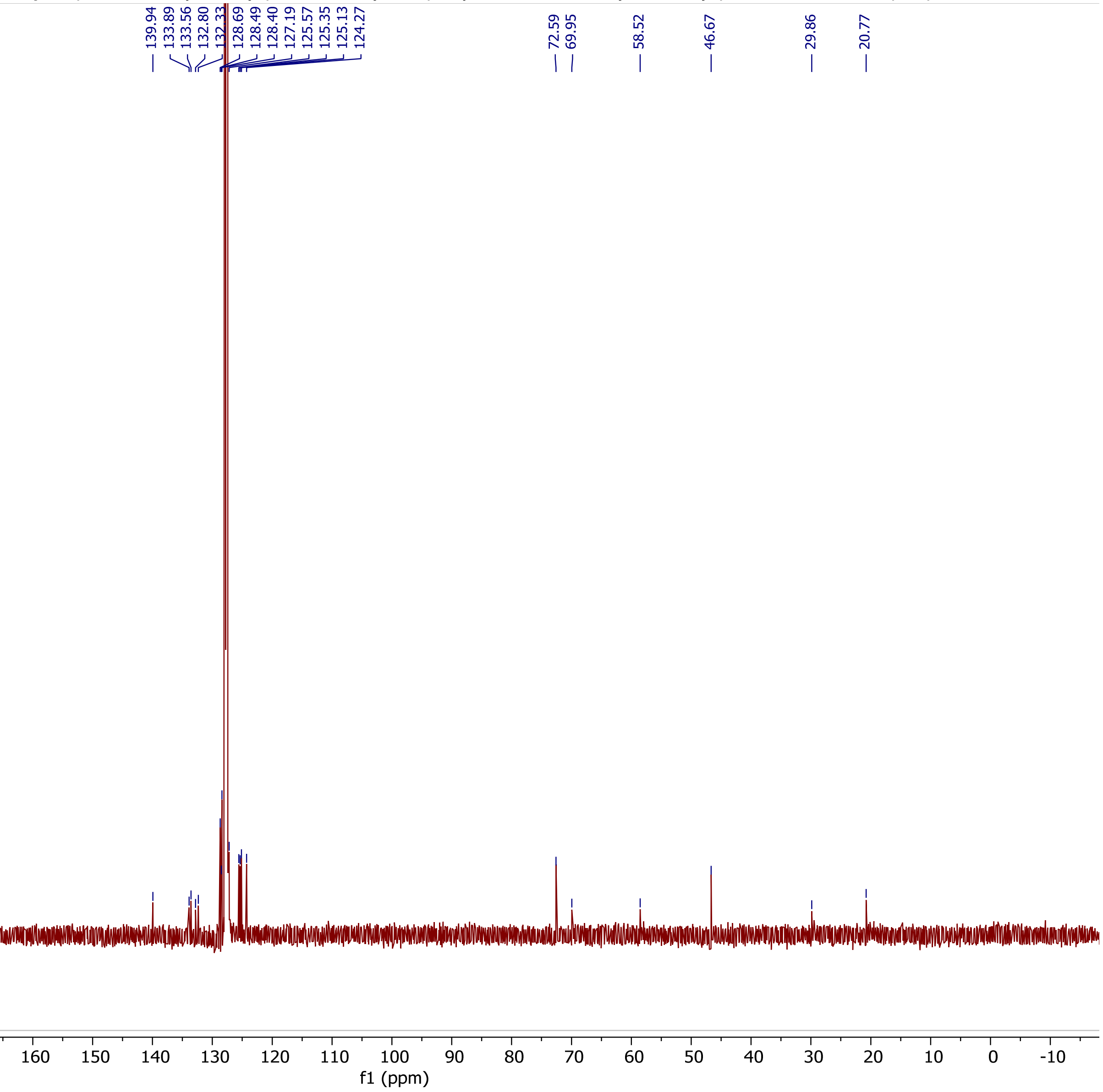

HSQC NMR (500 MHz, C_6D_6) 3-(4-Chlorophenyl)-5-methyl-2-(naphthalene-1-ylmethyl)isoxazolidine (**54**)

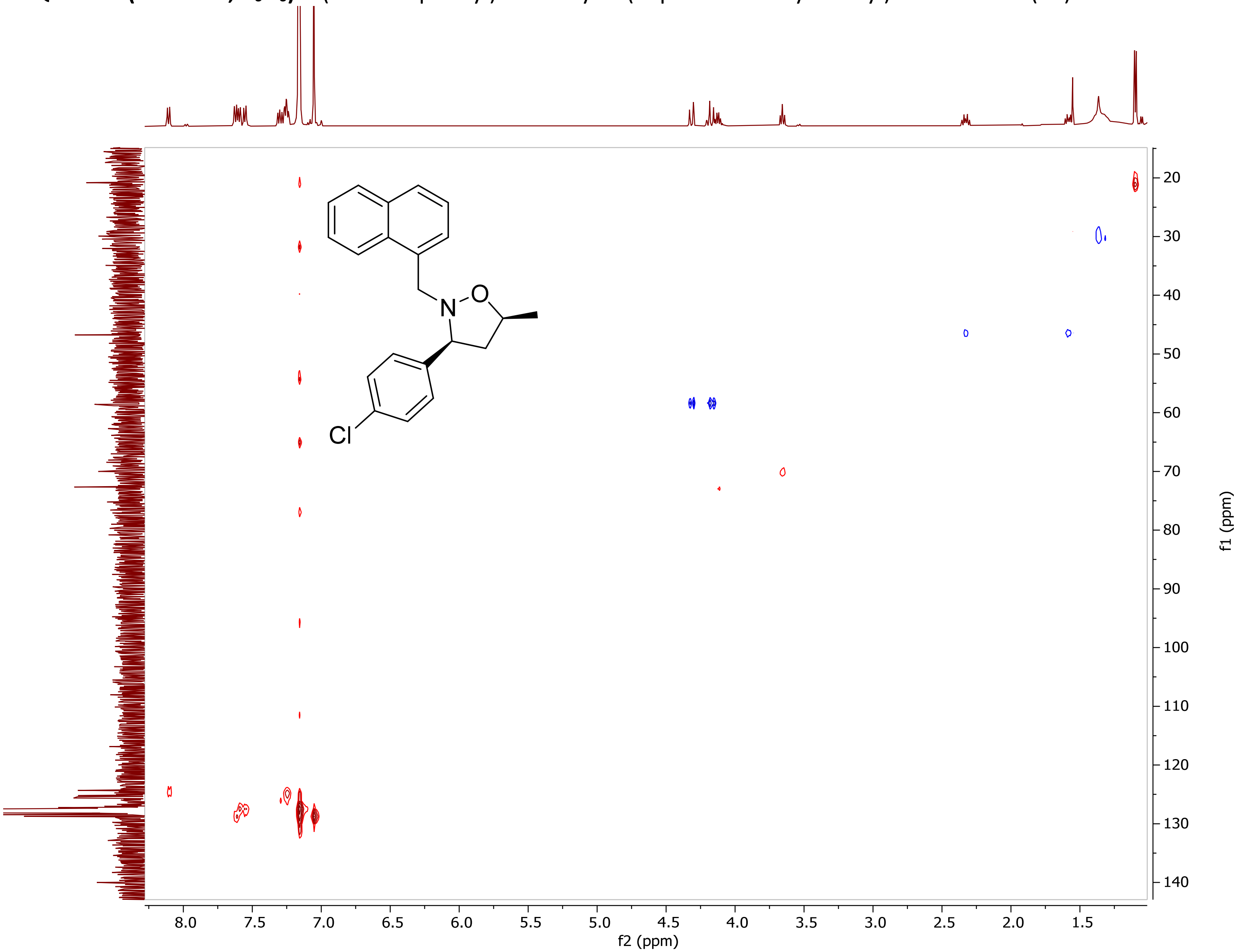

COSY NMR (500 MHz, C_6D_6) 3-(4-Chlorophenyl)-5-methyl-2-(naphthalene-1-ylmethyl)isoxazolidine (**54**)

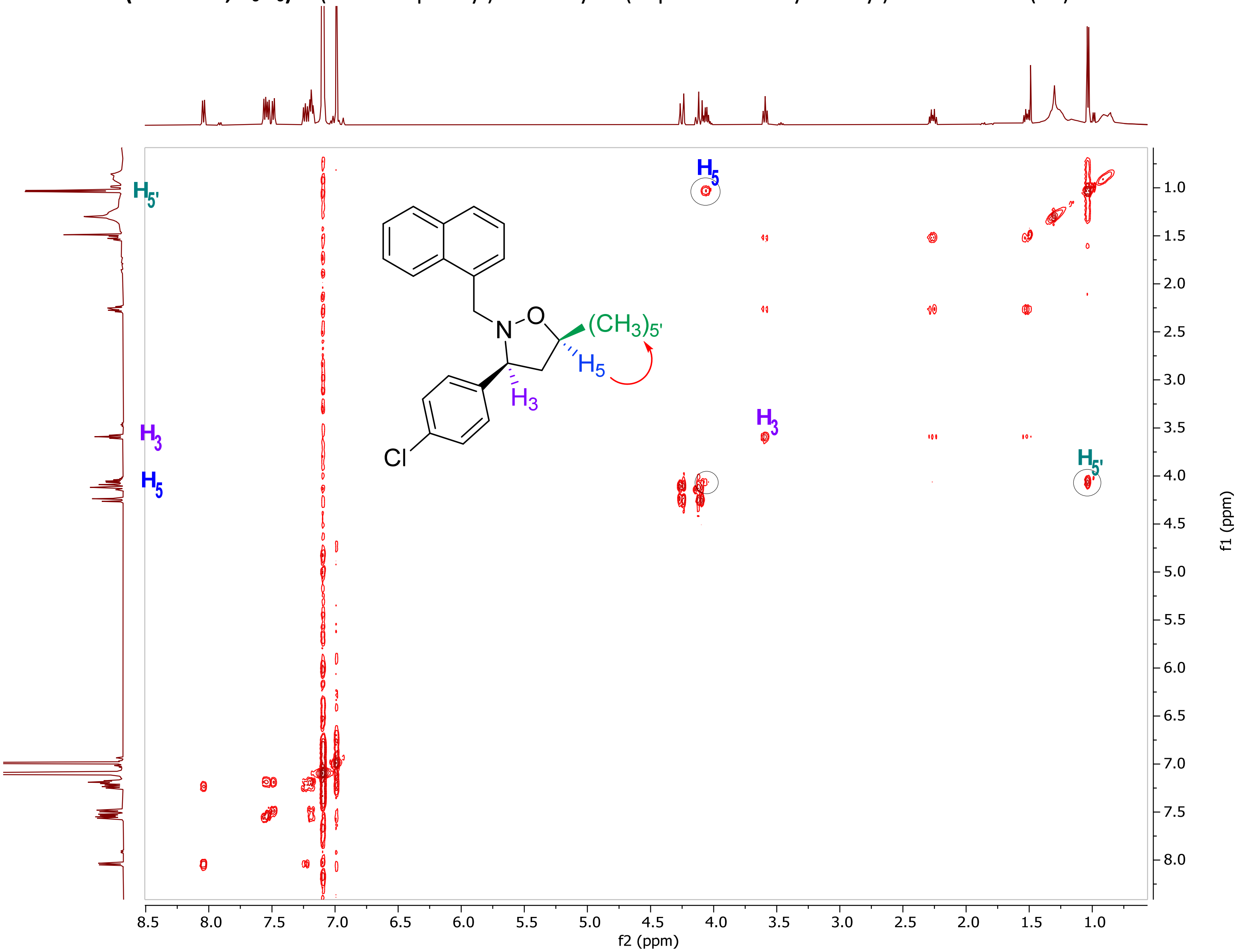

1D NOESY NMR (500 MHz, C_6D_6) 3-(4-Chlorophenyl)-5-methyl-2-(naphthalene-1-ylmethyl)isoxazolidine (**54**)

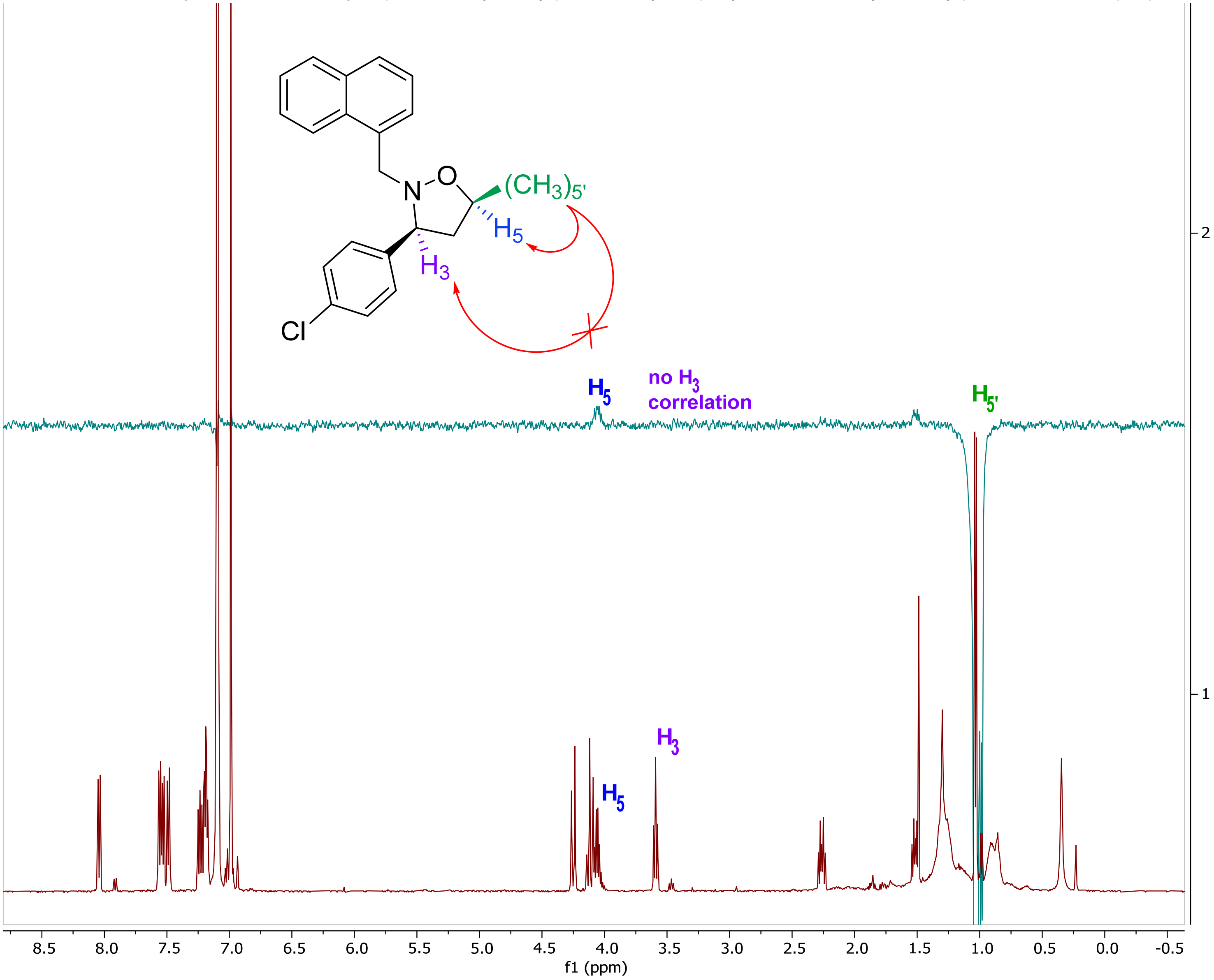

1D NOESY NMR (500 MHz, C_6D_6) 3-(4-Chlorophenyl)-5-methyl-2-(naphthalene-1-ylmethyl)isoxazolidine (**54**)

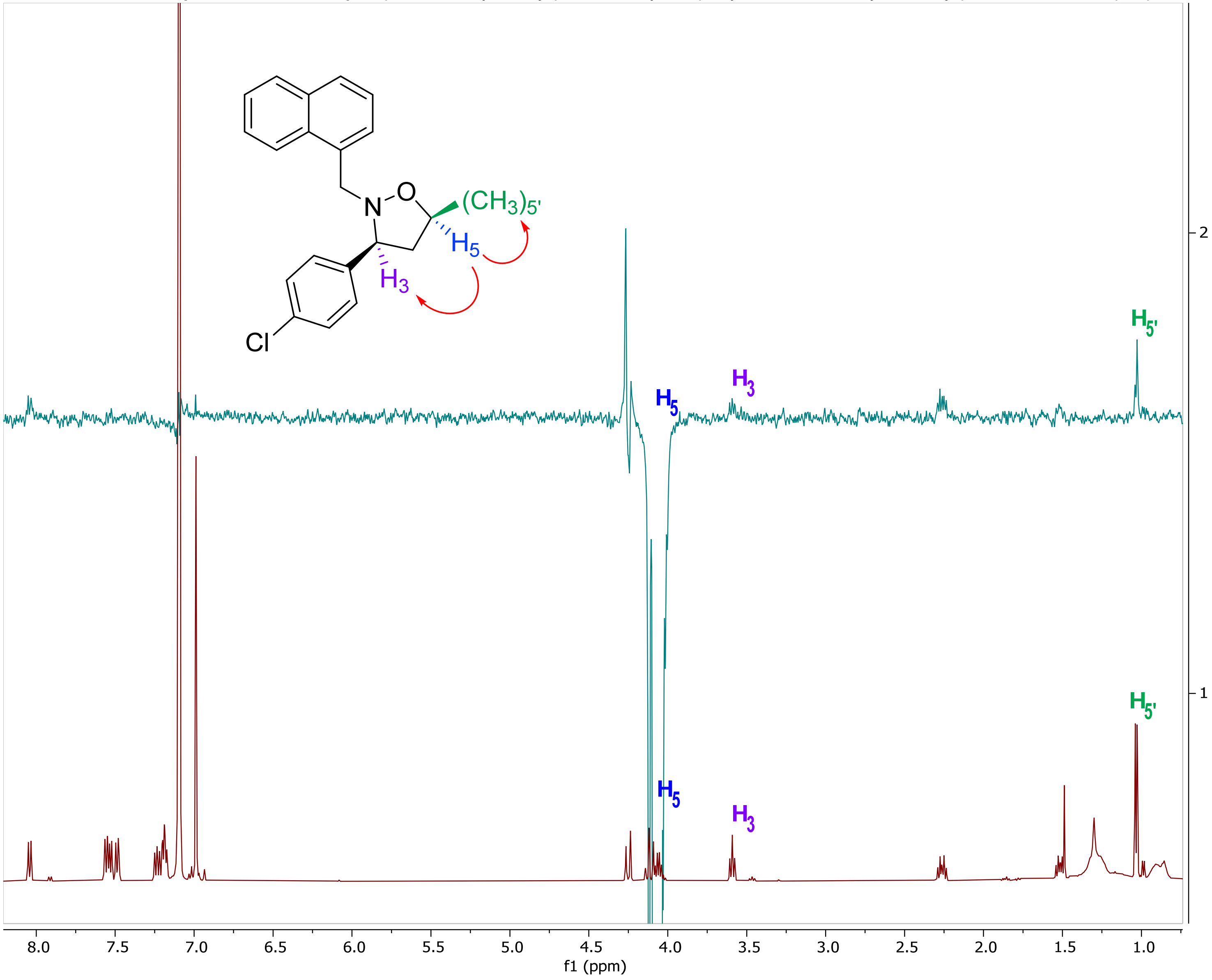

^{1}H NMR (500 MHz, $CDCl_3$) spectrum of 2-Nitro-*N*-(2-(prop-1-en-2-yl)phenyl)-4-(trifluoromethyl)benzenesulfonamide (**S7**)

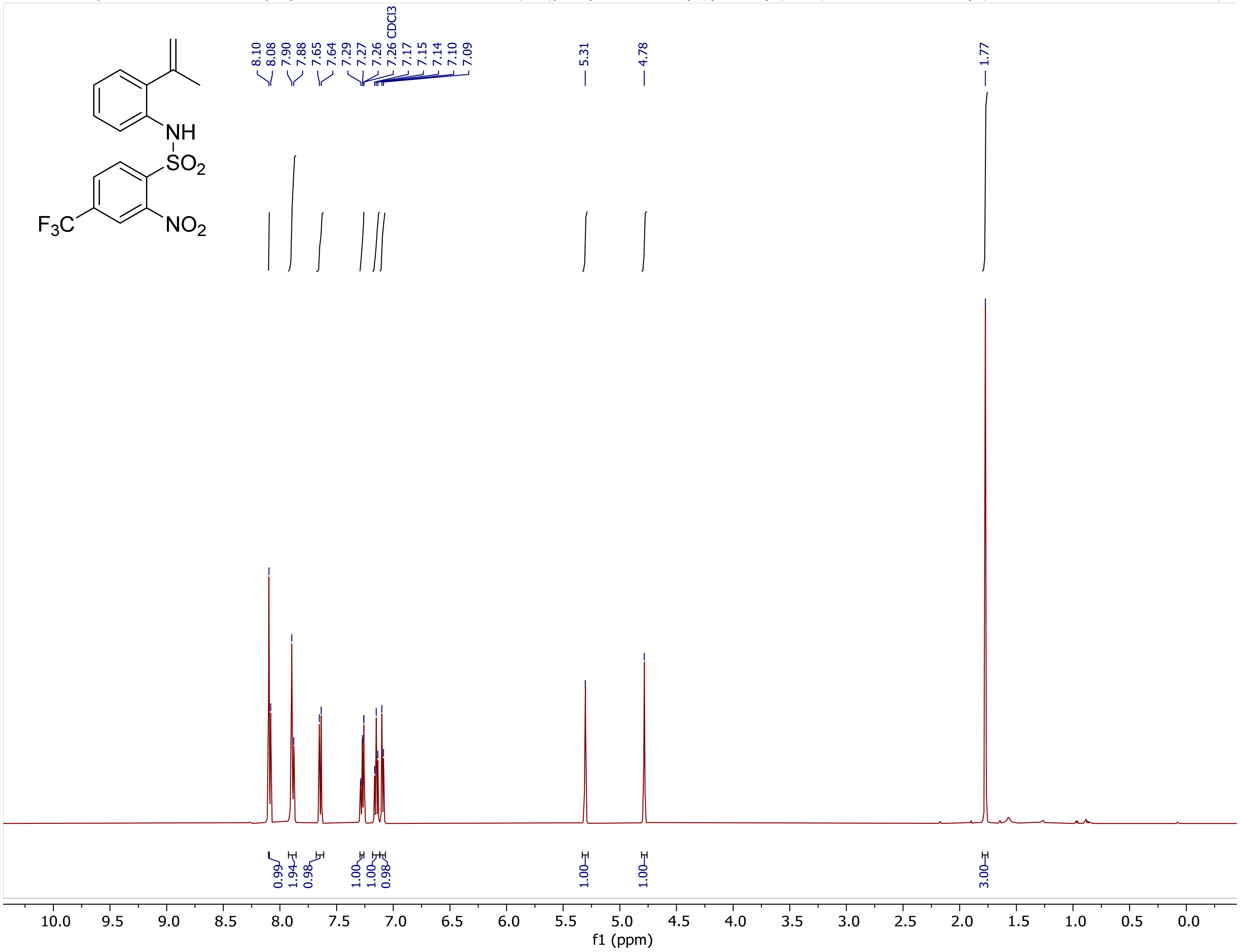

^{13}C {^{1}H} NMR (126 MHz, $CDCl_3$) spectrum of 2-Nitro-*N*-(2-(prop-1-en-2-yl)phenyl)-4-(trifluoromethyl)benzenesulfonamide (**S7**)

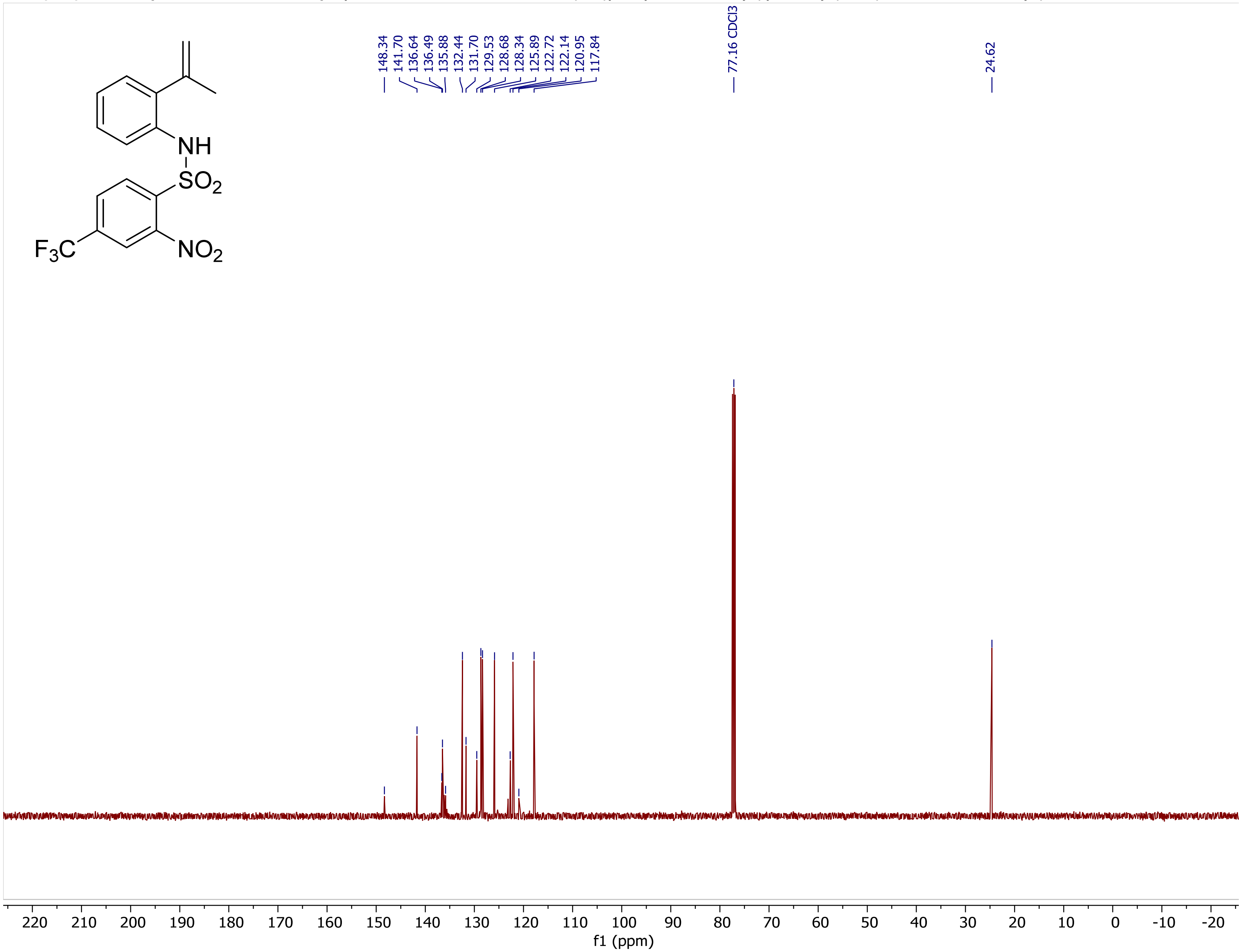

^{1}H NMR (500 MHz, $CDCl_3$) spectrum of *N*-(4-Methoxybenzyl)-2-nitro-*N*-(2-(prop-1-en-2-yl)phenyl)-4-(trifluoromethyl)benzenesulfonamide (**7a**)

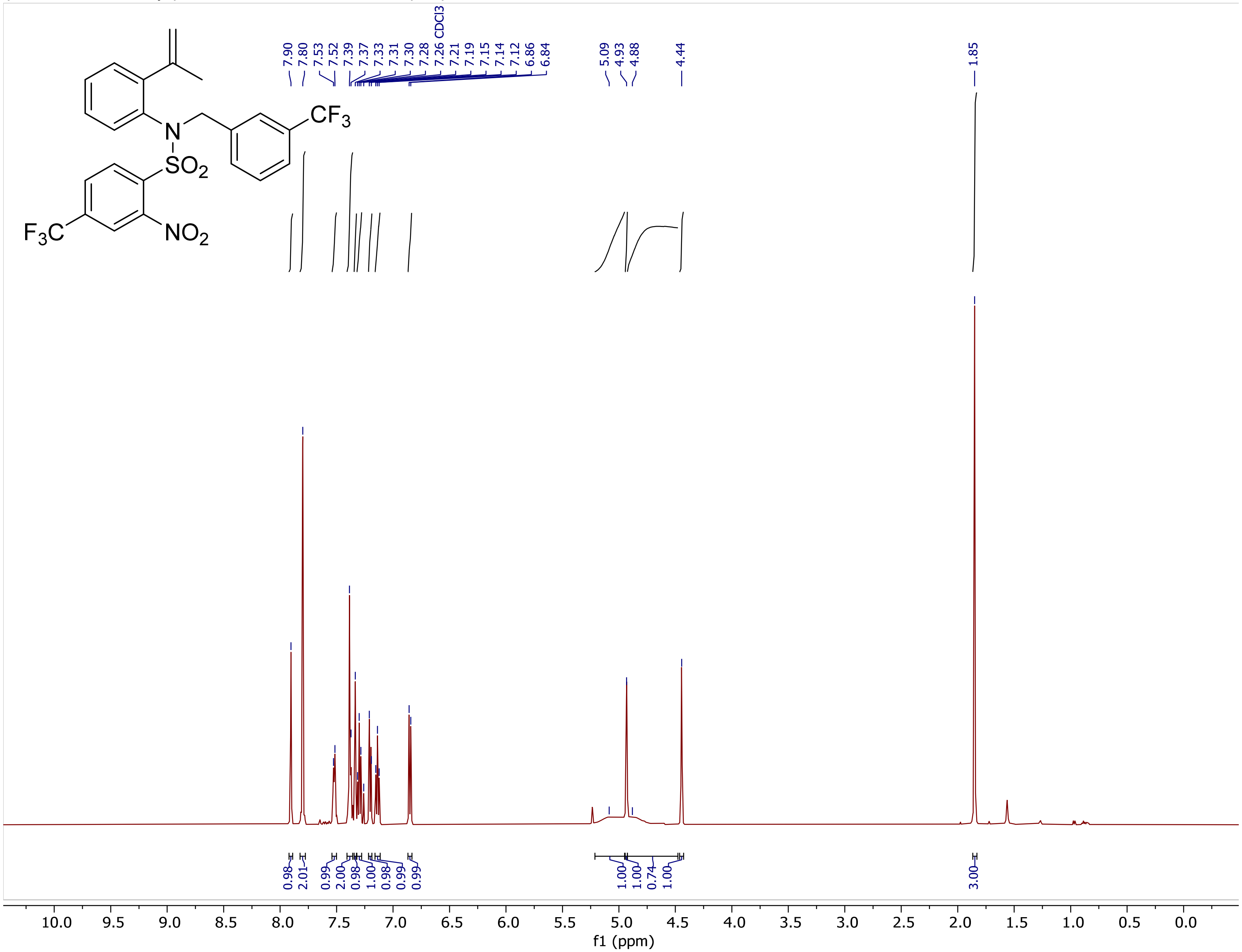

^{13}C {^{1}H} NMR (126 MHz, CDCl$_3$) spectrum of *N*-(4-Methoxybenzyl)-2-nitro-*N*-(2-(prop-1-en-2-yl)phenyl)-4-(trifluoromethyl)benzenesulfonamide (**7a**)

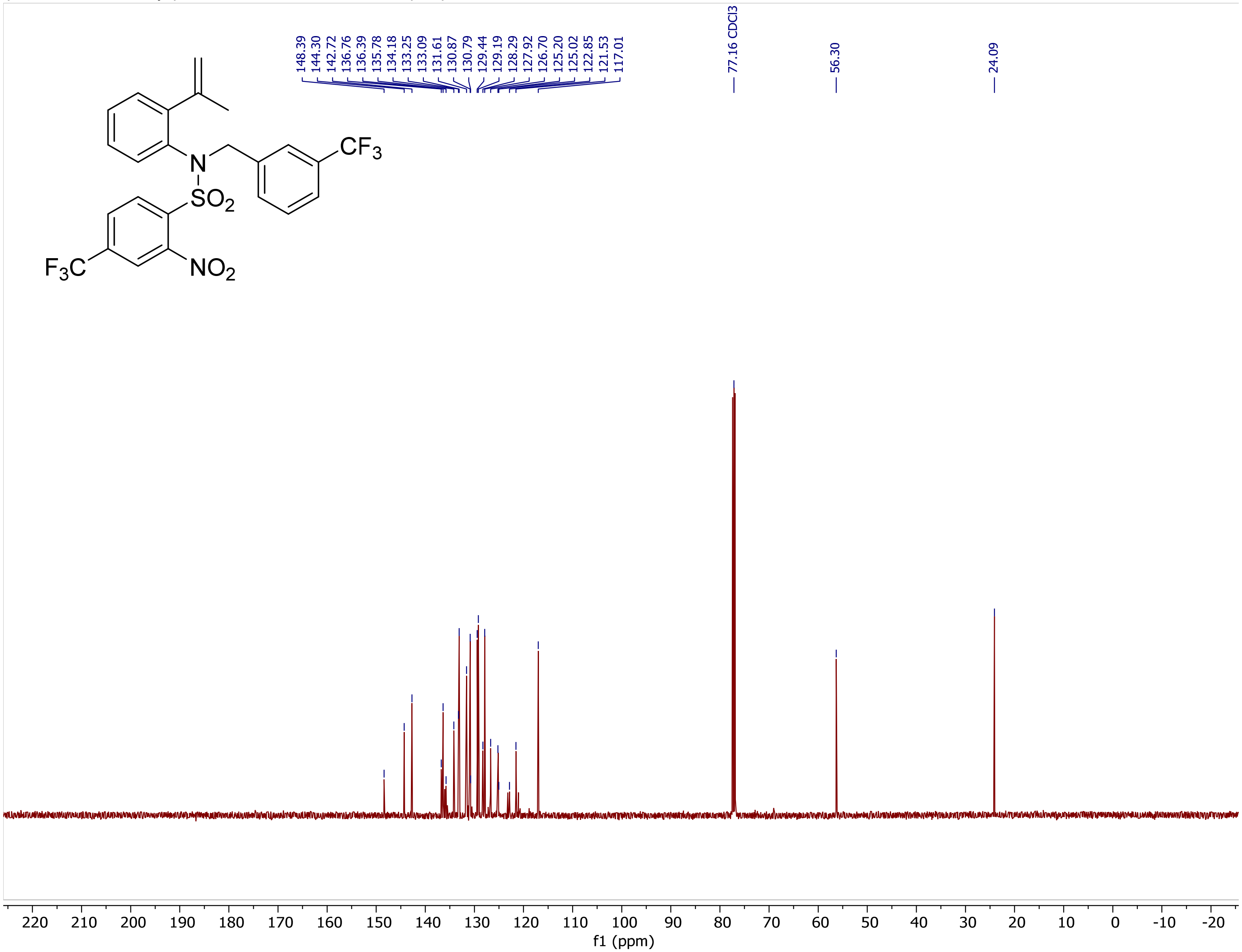

^{1}H NMR (500 MHz, C_6D_6) spectrum of 2-Nitro-*N*-(2-(2-((triethylsilyl)peroxy)propan-2-yl)phenyl)-4-(trifluoromethyl)-*N*-(3-(trifluoromethyl)benzyl)benzenesulfonamide (**55**)

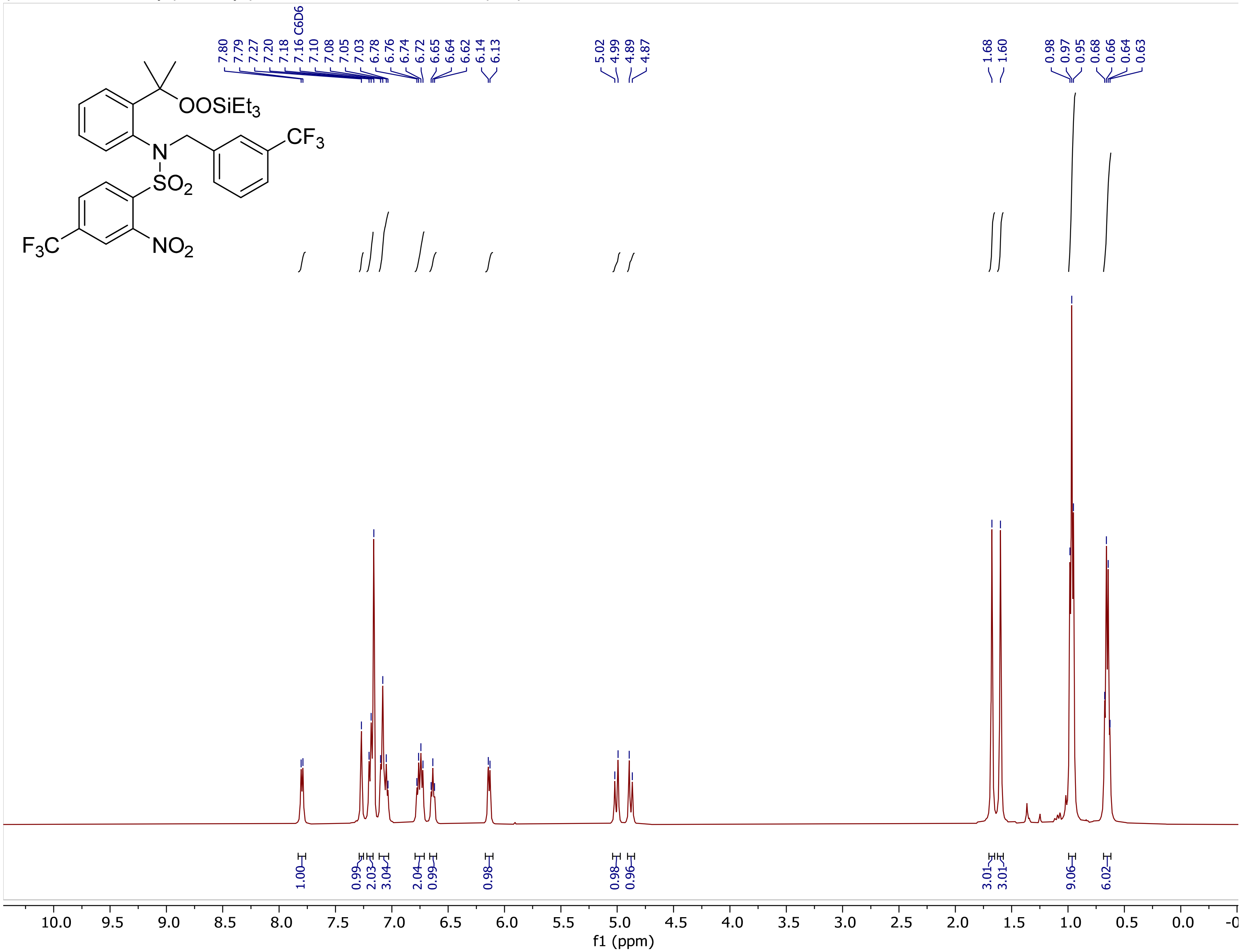

^{13}C {^{1}H} NMR (126 MHz, C_6D_6) spectrum of 2-Nitro-*N*-(2-(2-((triethylsilyl)peroxy)propan-2-yl)phenyl)-4-(trifluoromethyl)-*N*-(3-(trifluoromethyl)benzyl)benzenesulfonamide (**55**)

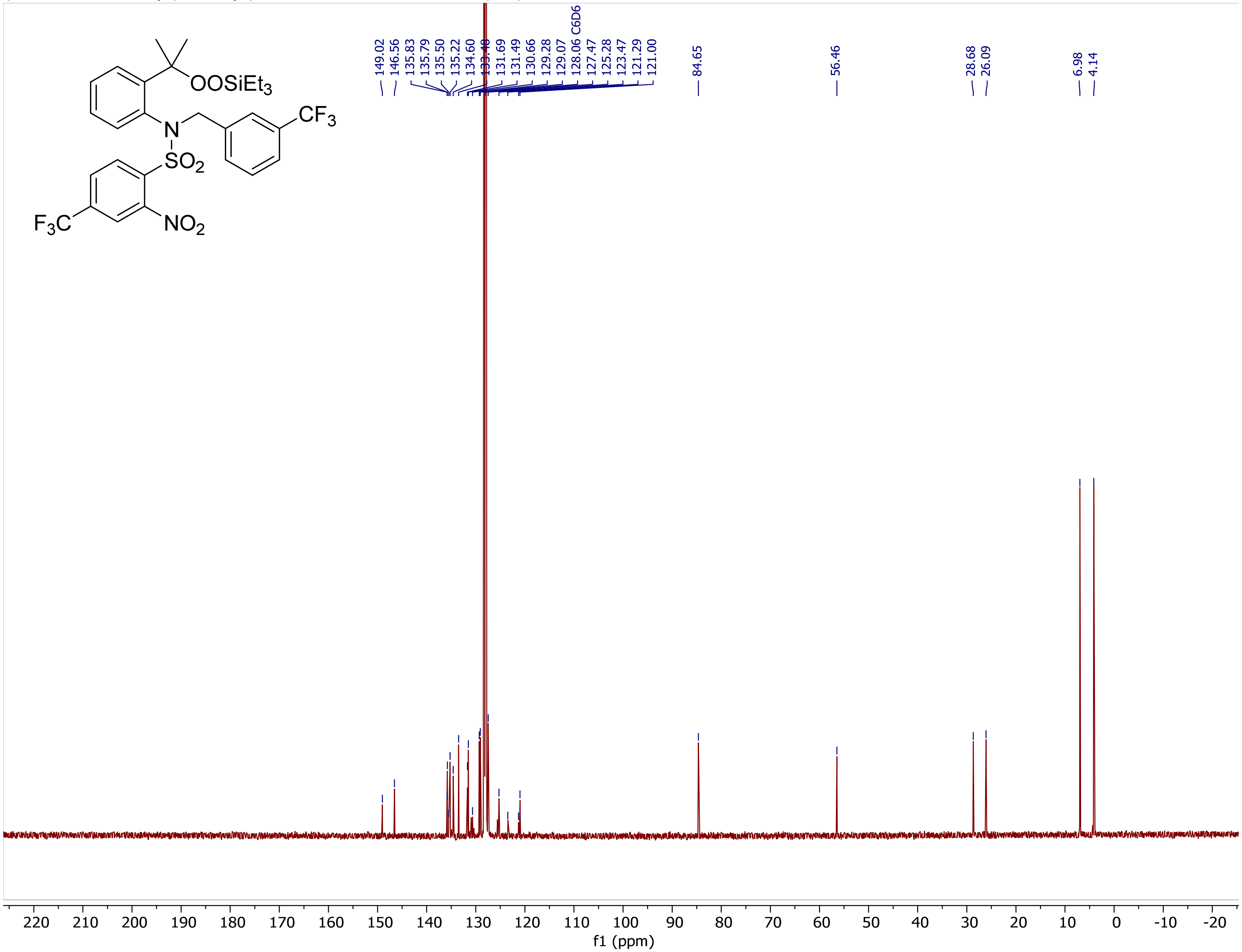

^{1}H NMR (500 MHz, C_6D_6) spectrum of 3,3-Dimethyl-1-(3-trifluoromethyl)benzyl)-1,3-dihydrobenzo[*c*]isoxazole (**56**)

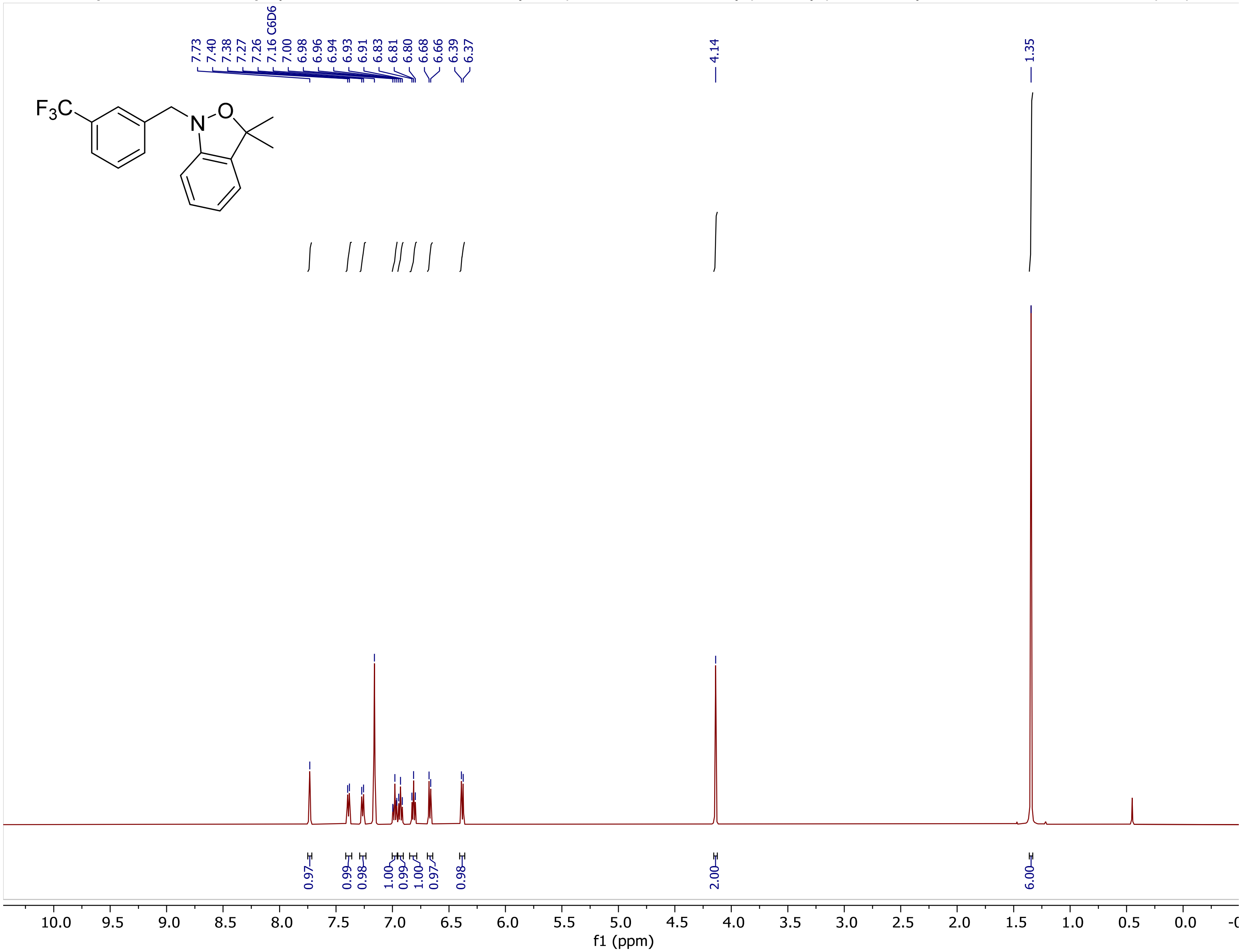

^{13}C {^{1}H, ^{19}F} NMR (126 MHz, C_6D_6) spectrum of 3,3-Dimethyl-1-(3-trifluoromethyl)benzyl)-1,3-dihydrobenzo[*c*]isoxazole (**56**)

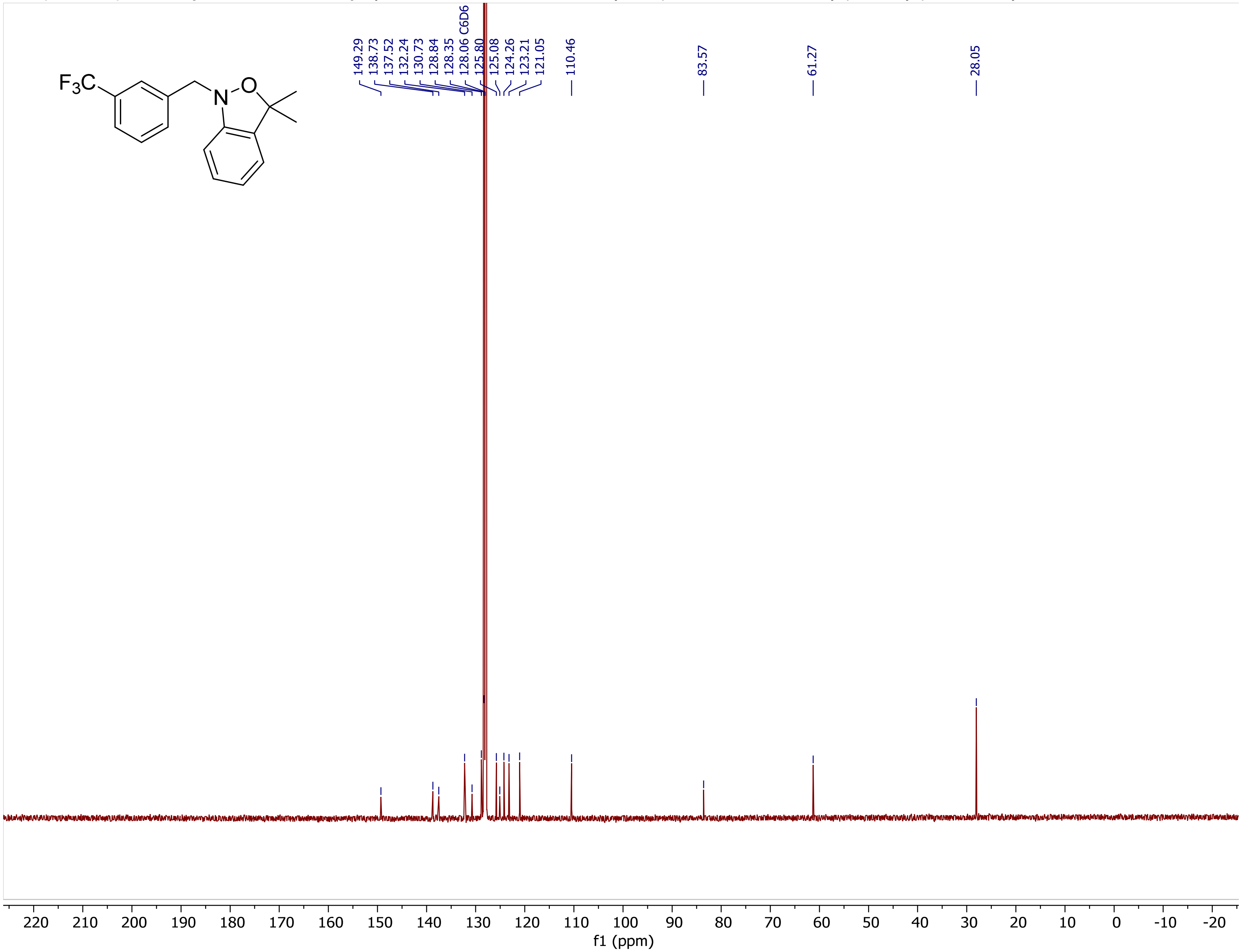

^{19}F NMR (470 MHz, C_6D_6) spectrum of 3,3-Dimethyl-1-(3-trifluoromethyl)benzyl)-1,3-dihydrobenzo[*c*]isoxazole (**56**)

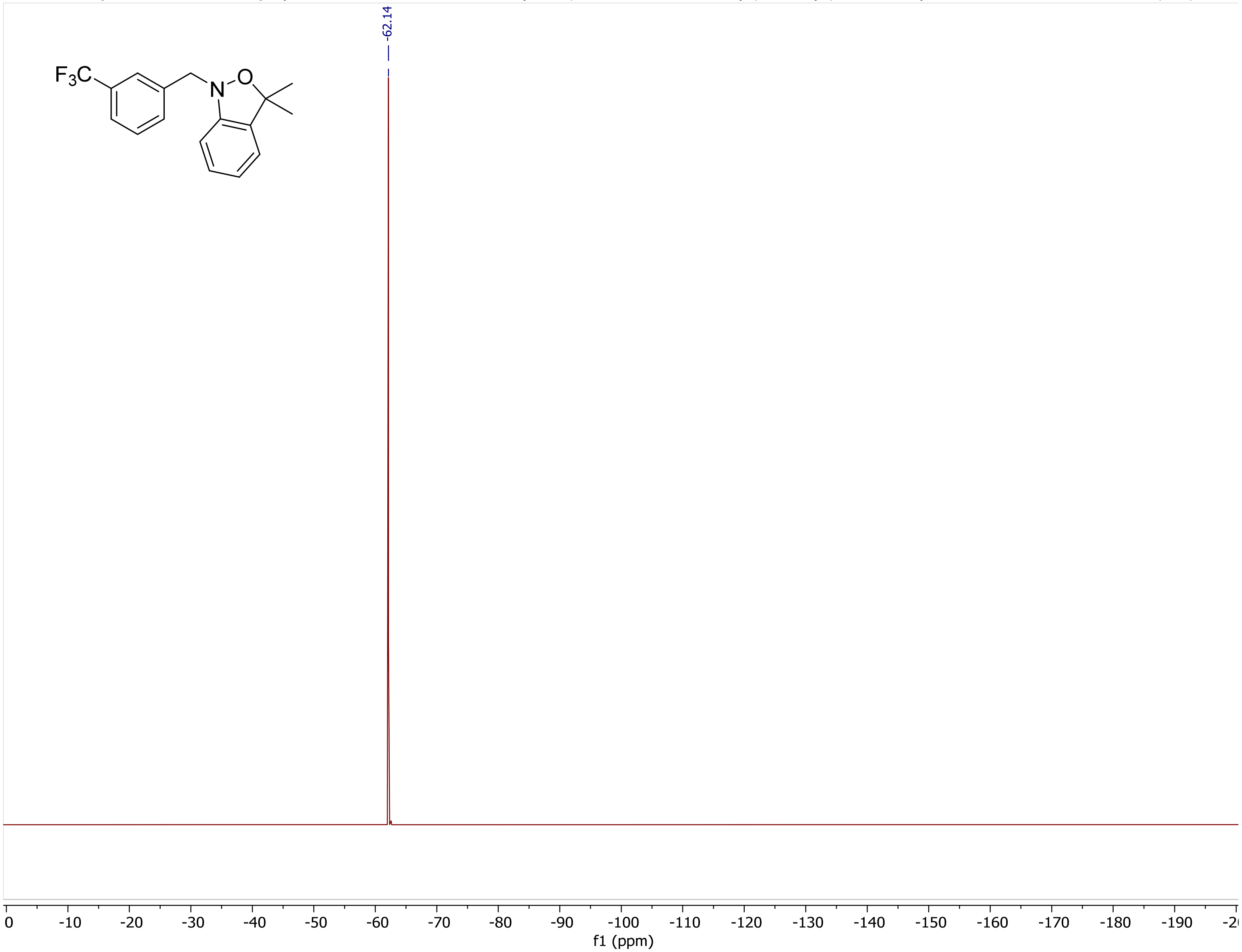

HSQC NMR (500 MHz, C_6D_6) spectrum of 3,3-Dimethyl-1-(3-trifluoromethyl)benzyl)-1,3-dihydrobenzo[*c*]isoxazole (**56**)

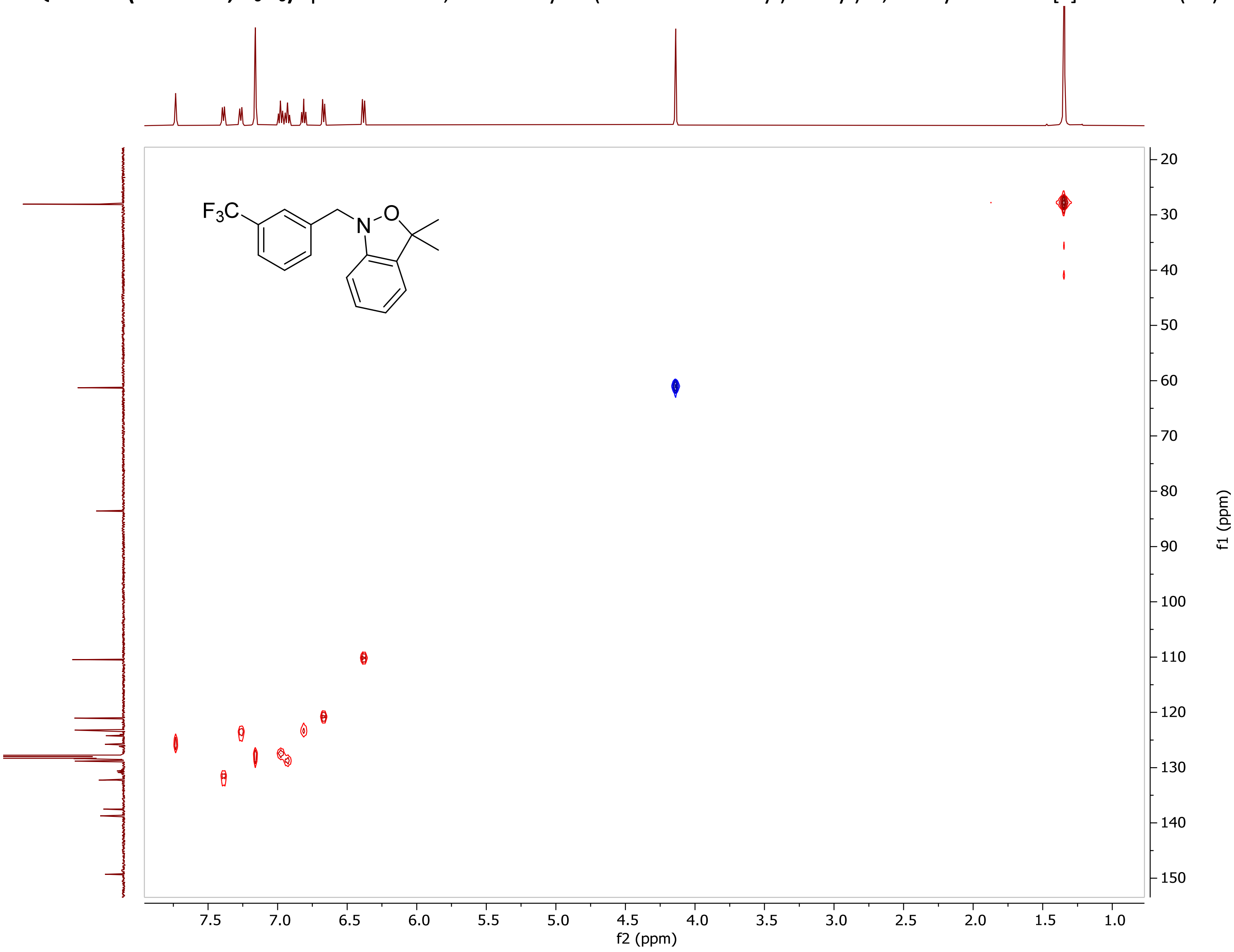

^{1}H NMR (500 MHz, $CDCl_3$) spectrum of *N*-(4-Methoxybenzyl)-2-nitro-*N*-(2-(prop-1-en-2-yl)phenyl)-4-(trifluoromethyl)benzenesulfonamide (**7b**)

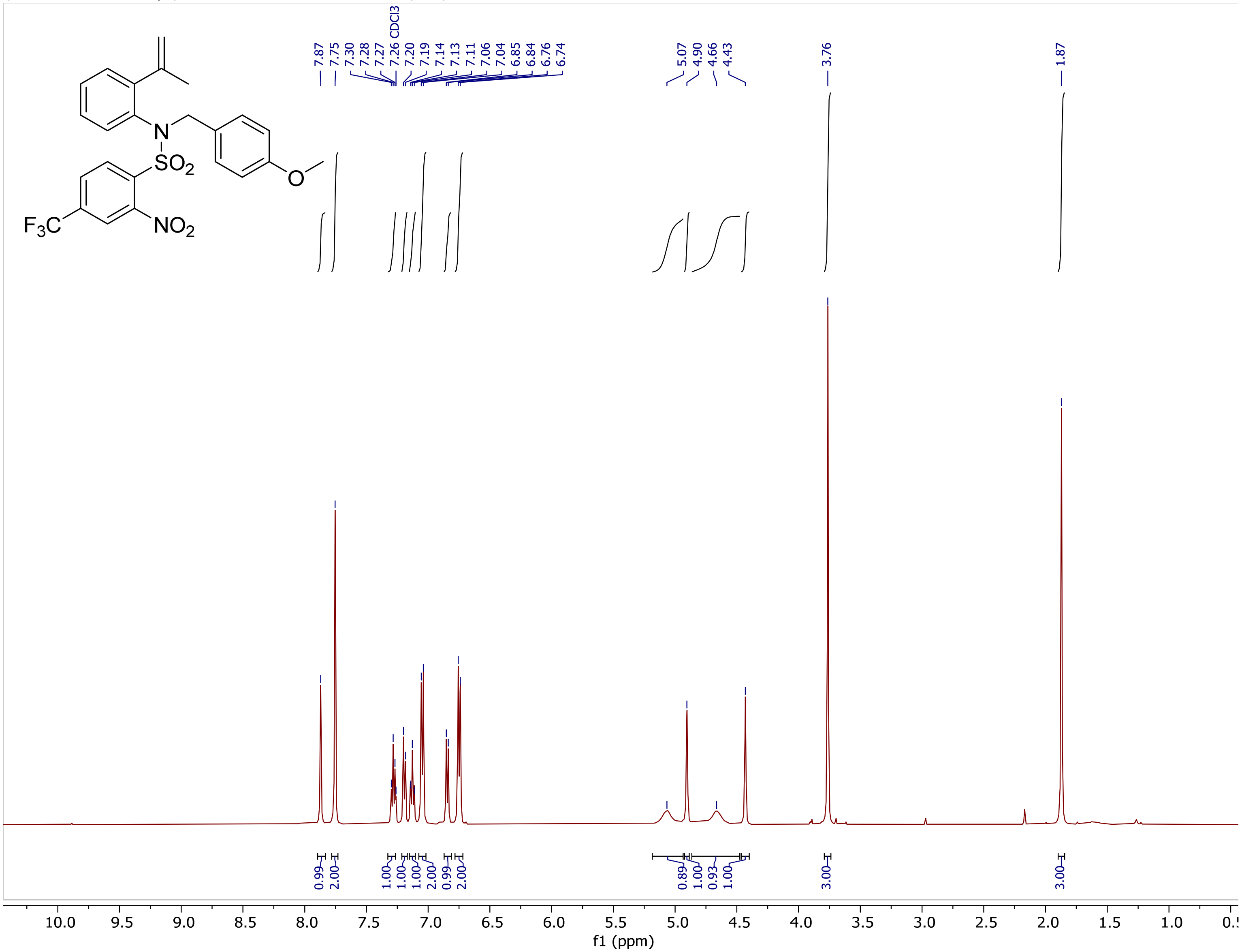

^{13}C {^{1}H} NMR (126 MHz, CDCl$_3$) spectrum of *N*-(4-Methoxybenzyl)-2-nitro-*N*-(2-(prop-1-en-2-yl)phenyl)-4-(trifluoromethyl)benzenesulfonamide (**7b**)

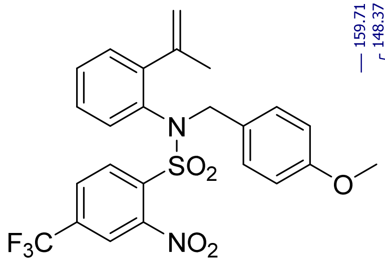

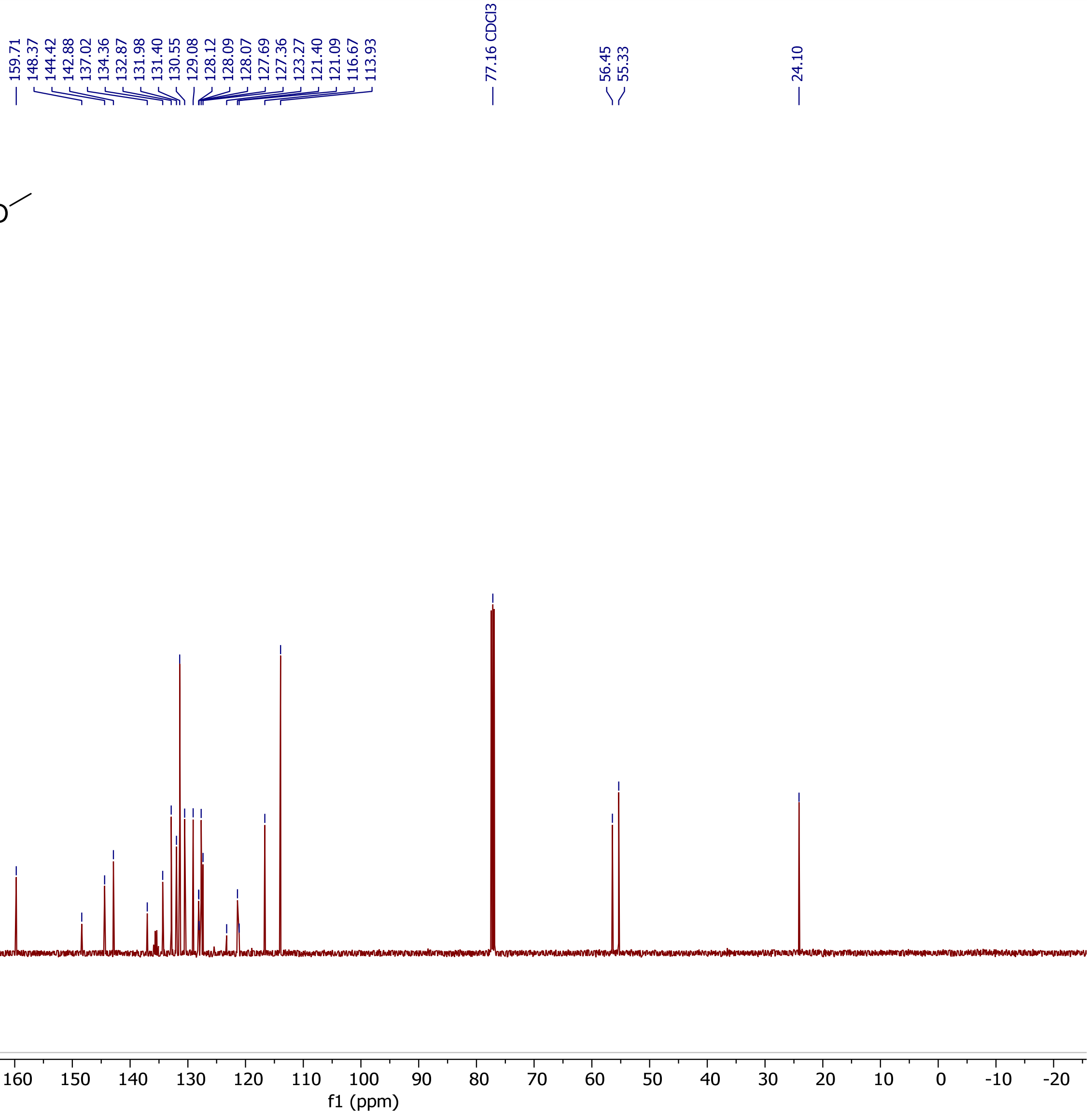

^{1}H NMR (500 MHz, C_6D_6) spectrum of *N*-(4-Methoxybenzyl)-2-nitro-*N*-(2-(2-((triethylsilyl)peroxy)propan-2-yl)phenyl)-4-(trifluoromethyl)benzenesulfonamide (**57**)

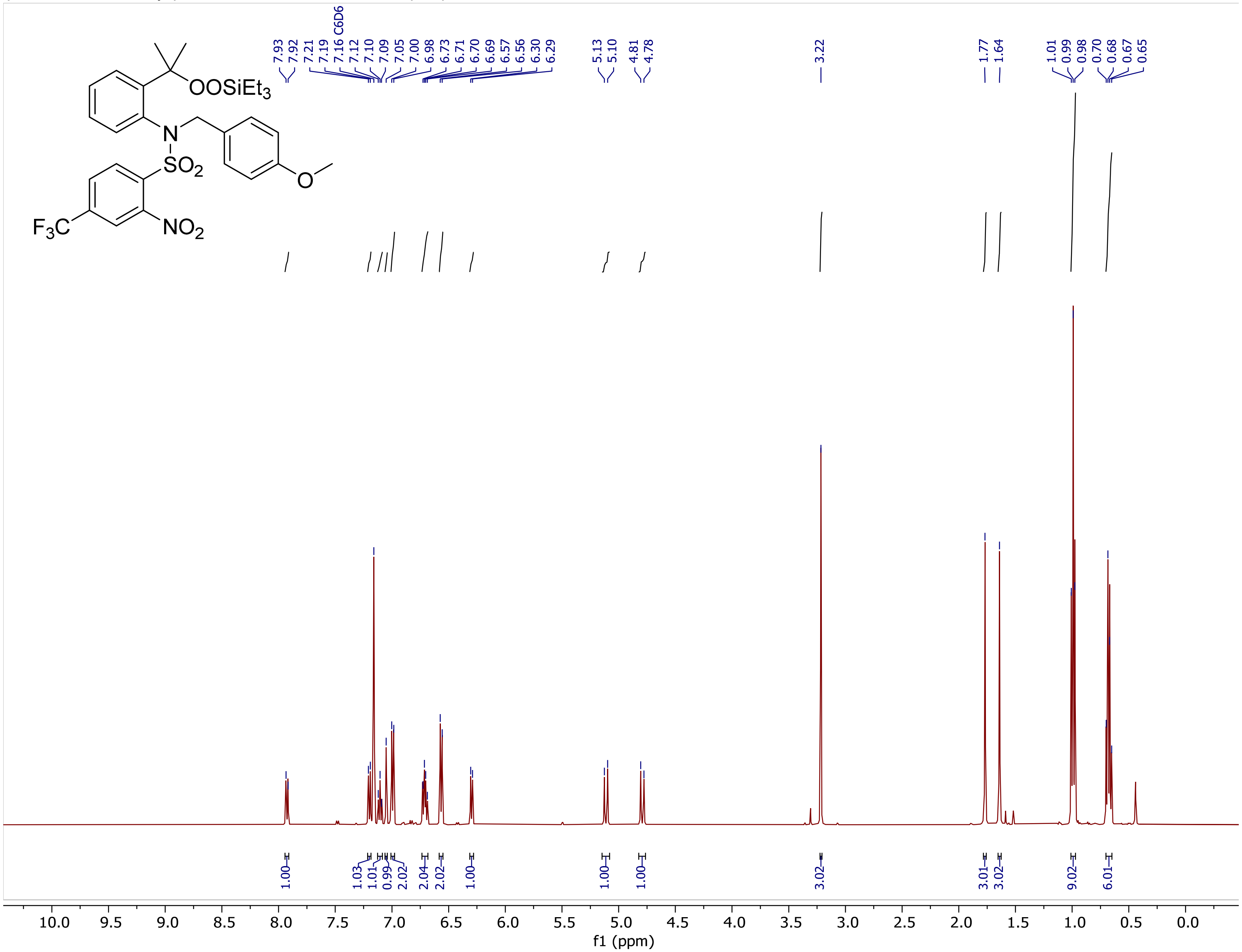

^{13}C {^{1}H, ^{19}F} NMR (126 MHz, C_6D_6) spectrum of *N*-(4-Methoxybenzyl)-2-nitro-*N*-(2-(2-((triethylsilyl)peroxy)propan-2-yl)phenyl)-4-(trifluoromethyl)benzenesulfonamide (**57**)

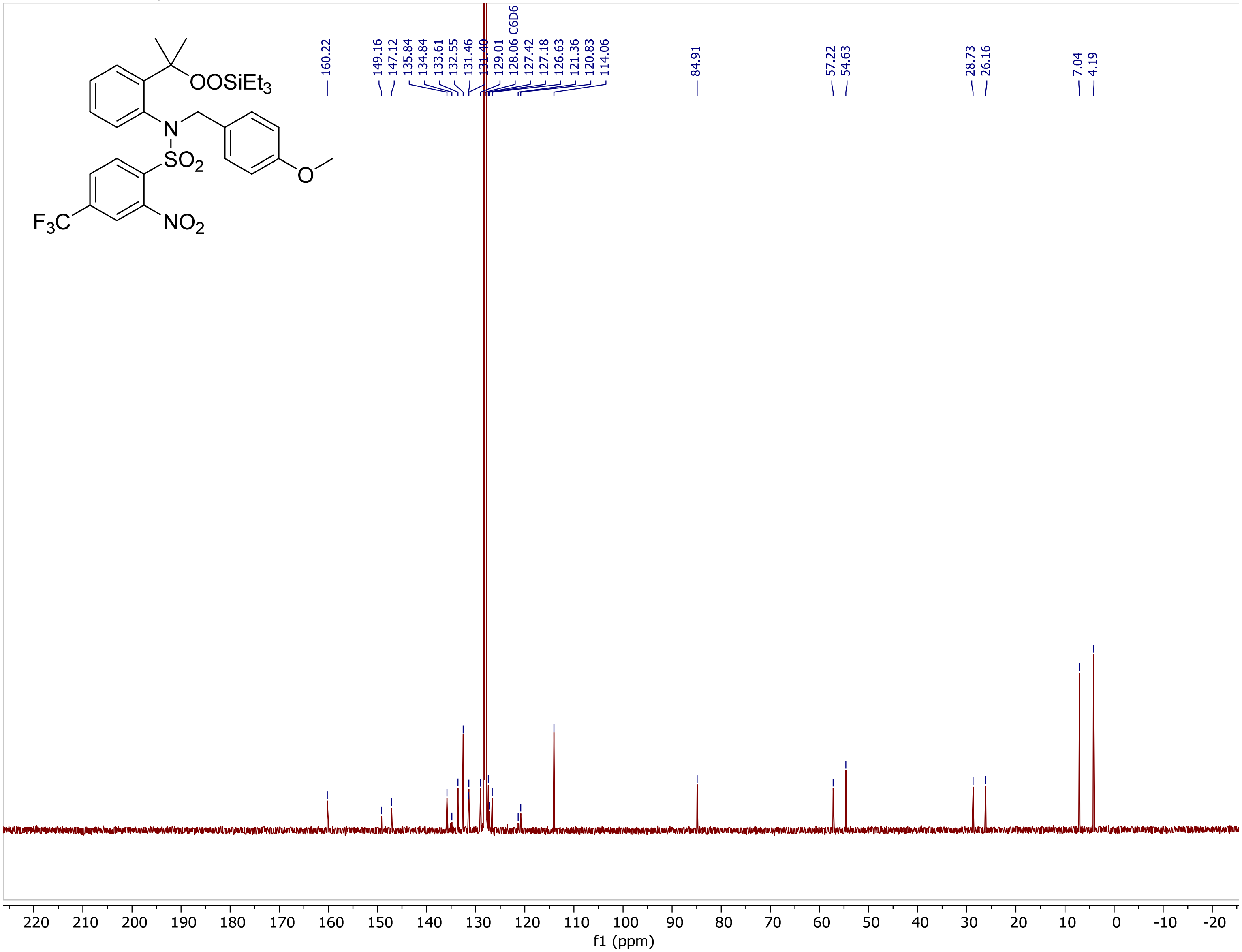

^{1}H NMR (500 MHz, C_6D_6) spectrum of 1-(4-Methoxybenzyl)-3,3-dimethyl-1,3-dihydrobenzo[*c*]isoxazole (**58**)

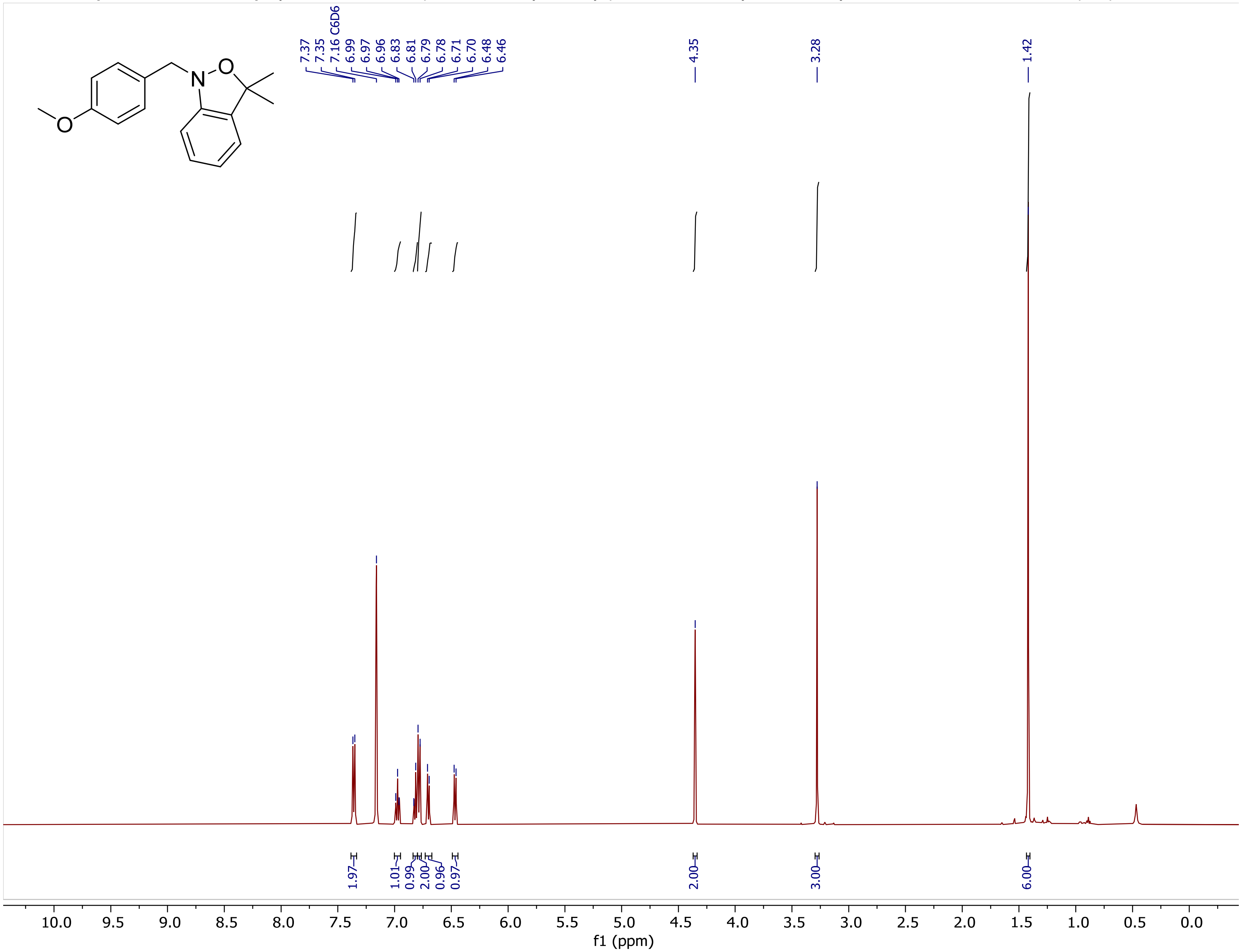

^{13}C {^{1}H} NMR (126 MHz, C_6D_6) spectrum of 1-(4-Methoxybenzyl)-3,3-dimethyl-1,3-dihydrobenzo[*c*]isoxazole (**58**)

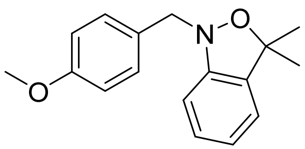

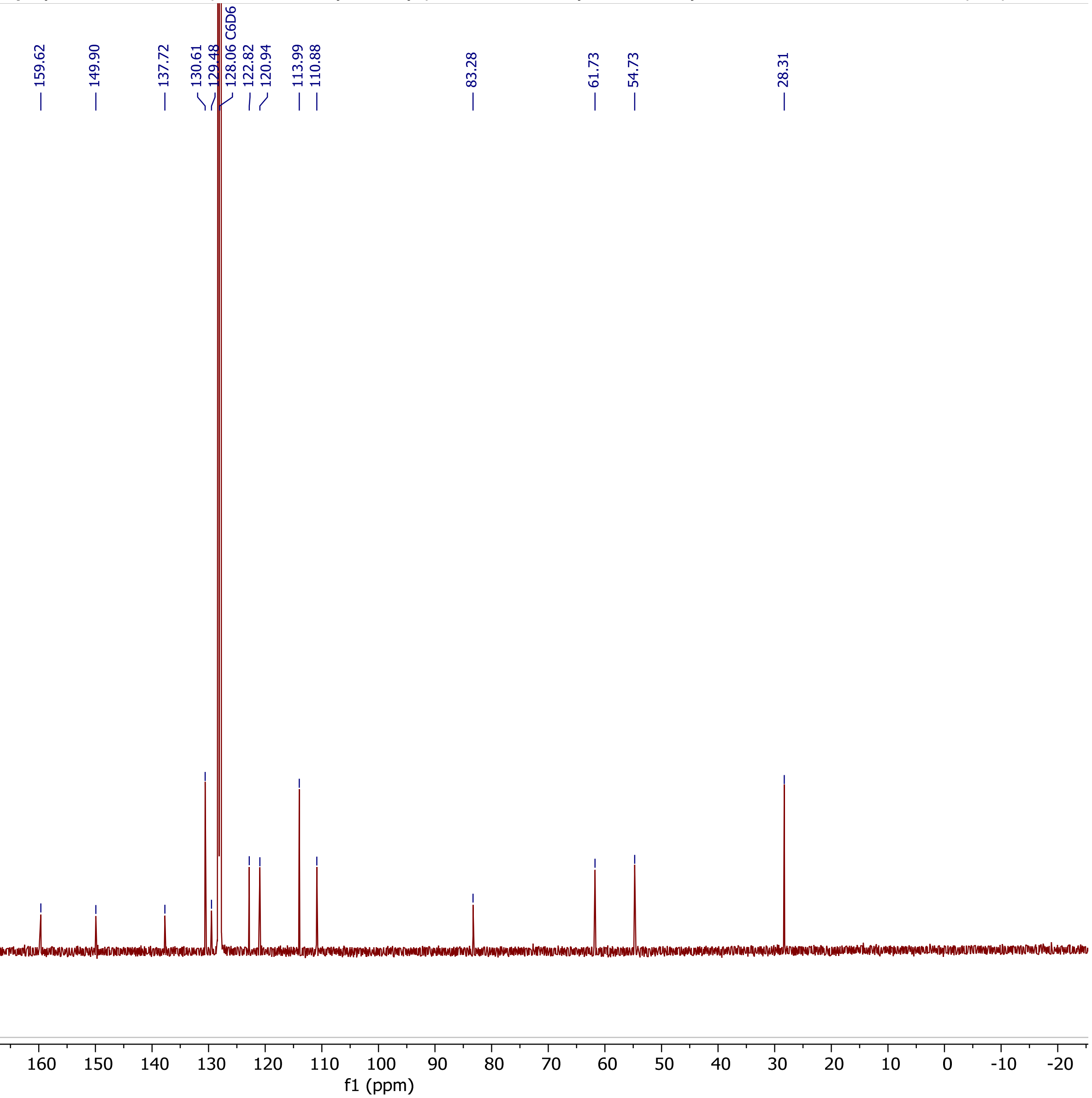

HSQC NMR (500 MHz, C_6D_6) spectrum of 1-(4-Methoxybenzyl)-3,3-dimethyl-1,3-dihydrobenzo[*c*]isoxazole (**58**)

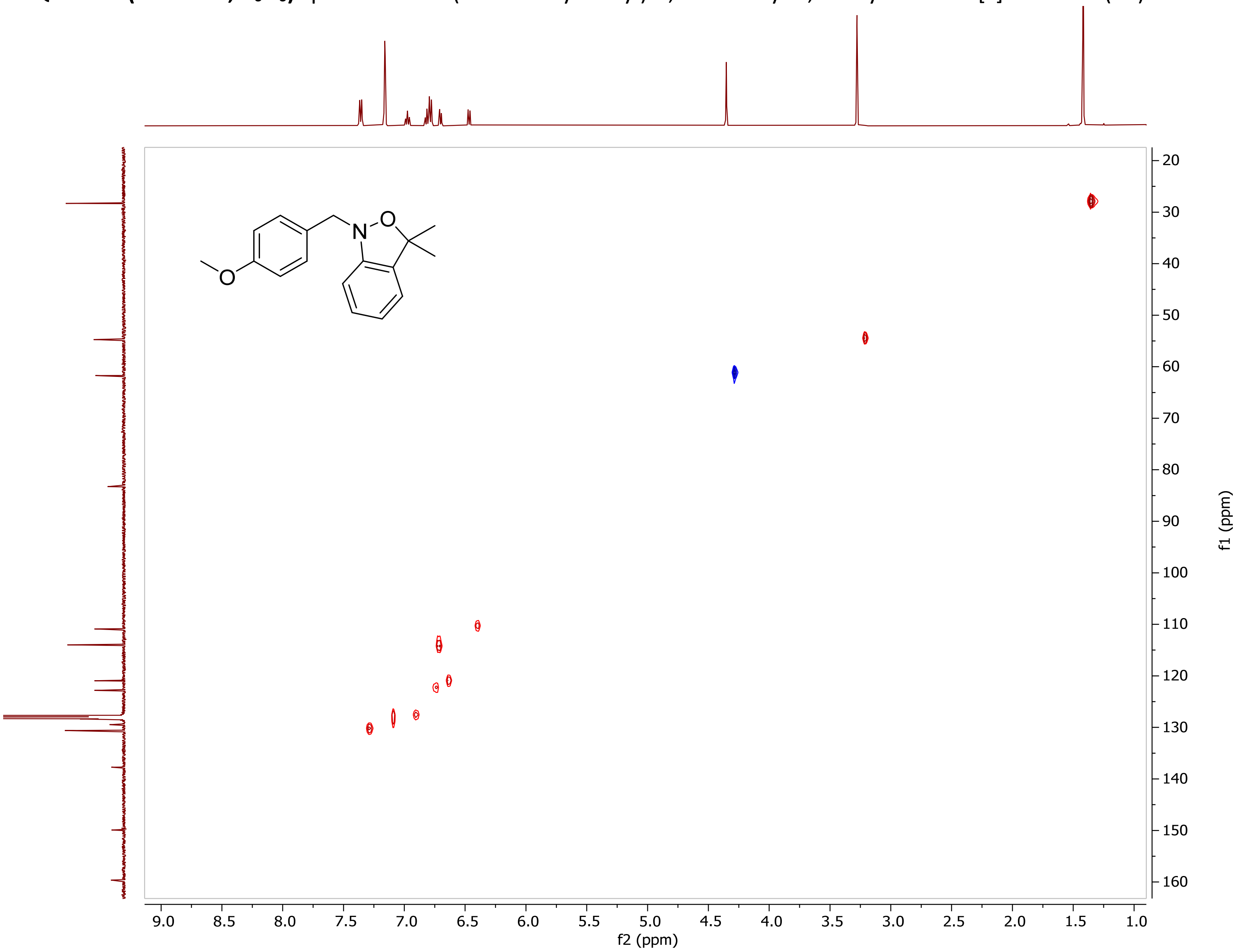

^{1}H NMR (500 MHz, C_6D_6) spectrum of *N*-Benzyl-2-nitro-*N*-(2-(prop-1-en-2-yl)phenyl)-4-(trifluoromethyl)benzenesulfonamide (**7c**)

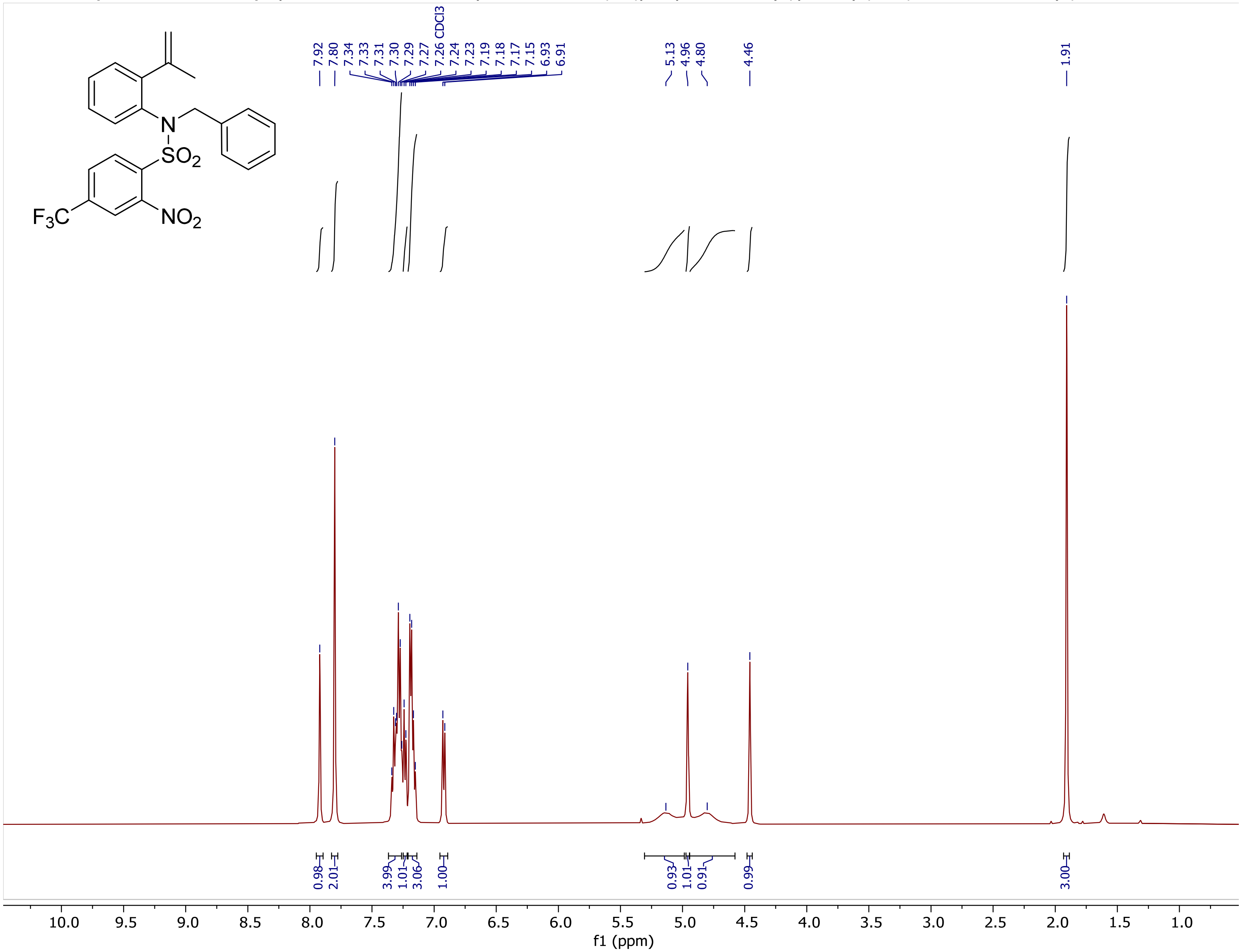

^{13}C {^{1}H, ^{19}F} NMR (126 MHz, C_6D_6) spectrum of *N*-Benzyl-2-nitro-*N*-(2-(prop-1-en-2-yl)phenyl)-4-(trifluoromethyl)benzenesulfonamide (**7c**)

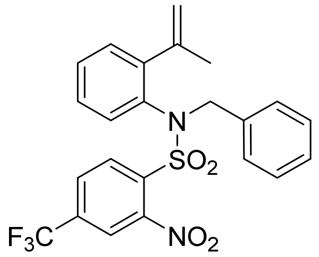

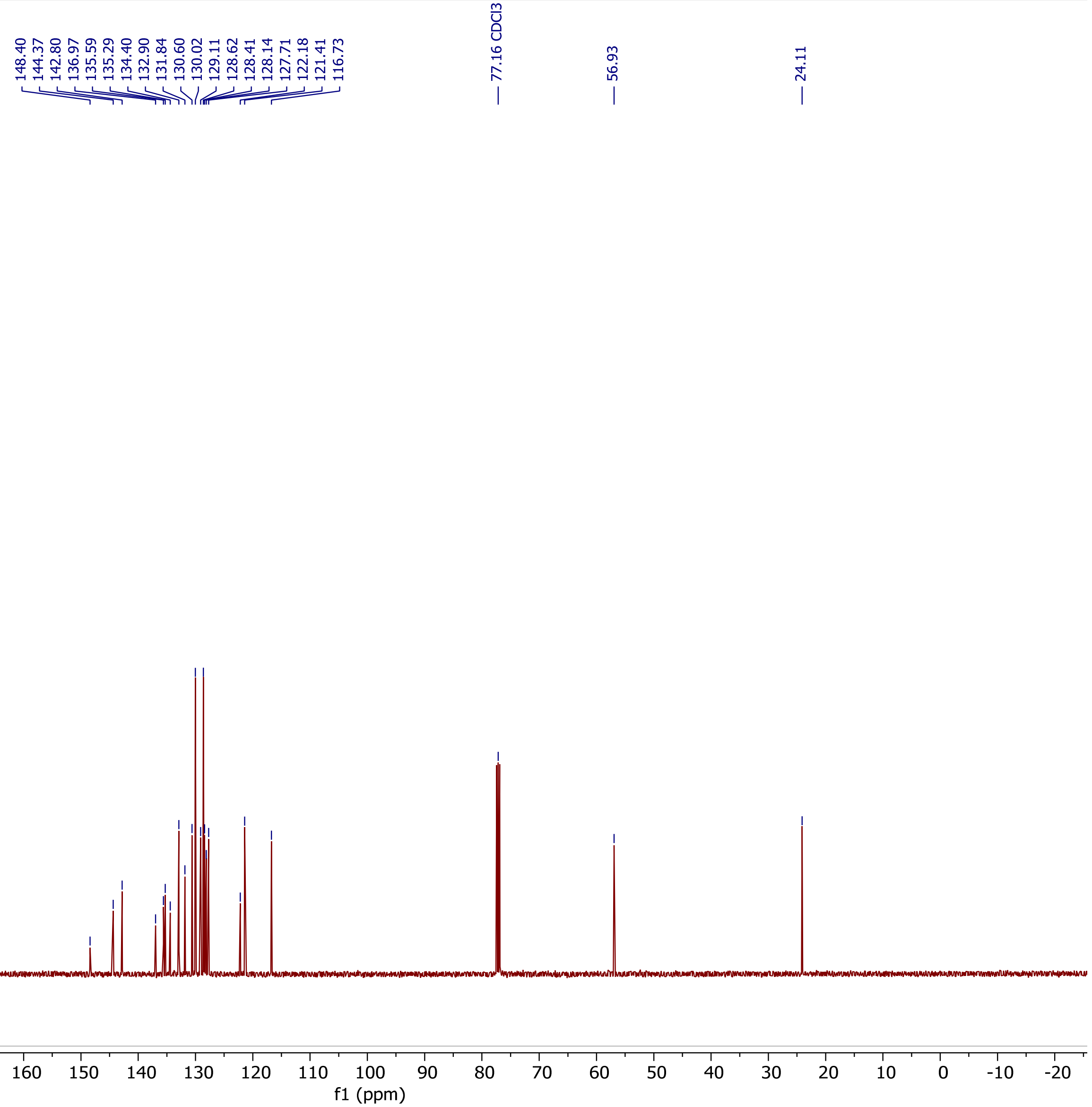

^{1}H NMR (500 MHz, C_6D_6) spectrum of *N*-Benzyl-2-nitro-*N*-(2-(2-((triethylsilyl)peroxy)propan-2-yl)phenyl)-4-(trifluoromethyl)benzenesulfonamide (**59**)

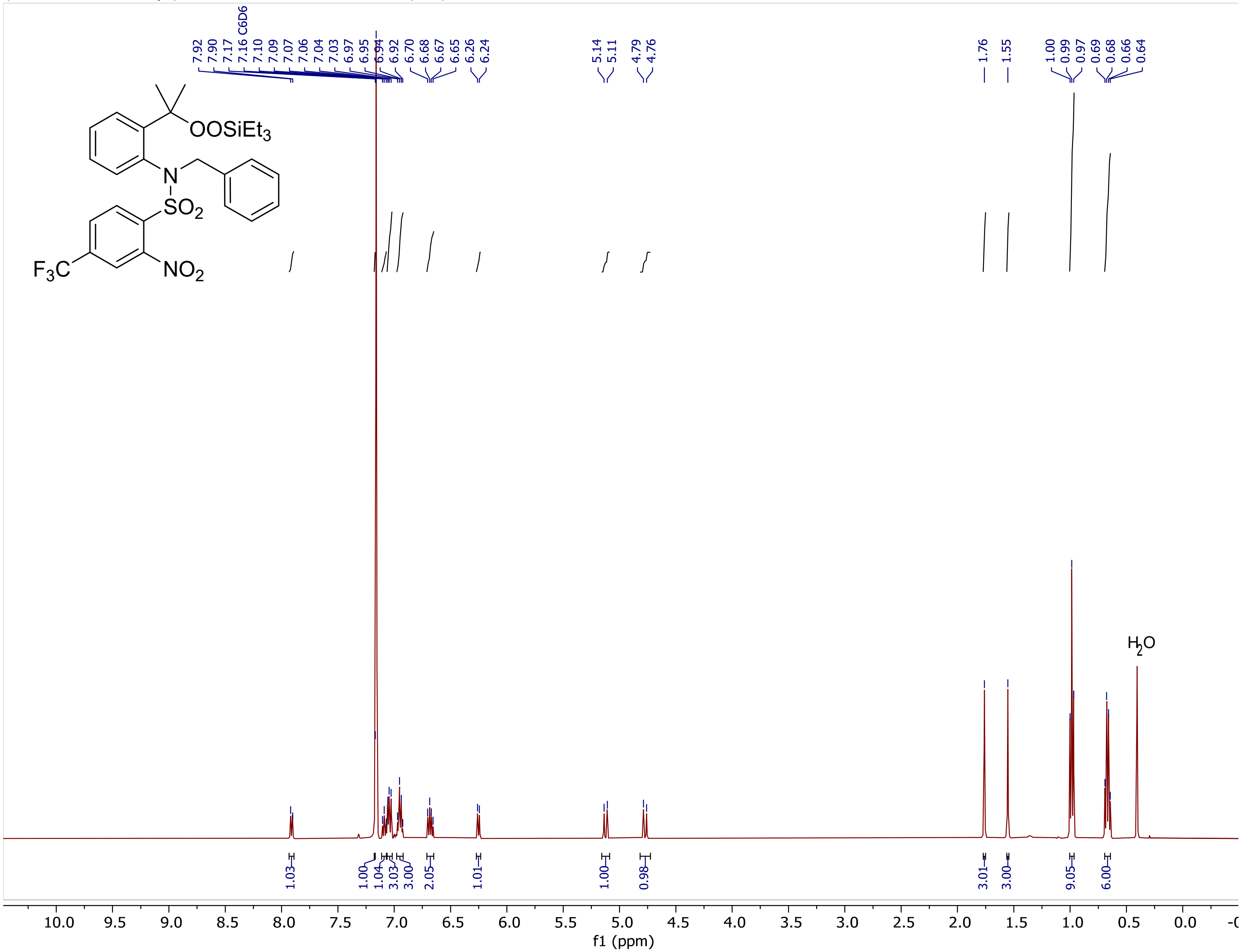

^{13}C {^{1}H, ^{19}F} NMR (126 MHz, C_6D_6) spectrum of *N*-Benzyl-2-nitro-*N*-(2-(2-((triethylsilyl)peroxy)propan-2-yl)phenyl)-4-(trifluoromethyl)benzenesulfonamide (**59**)

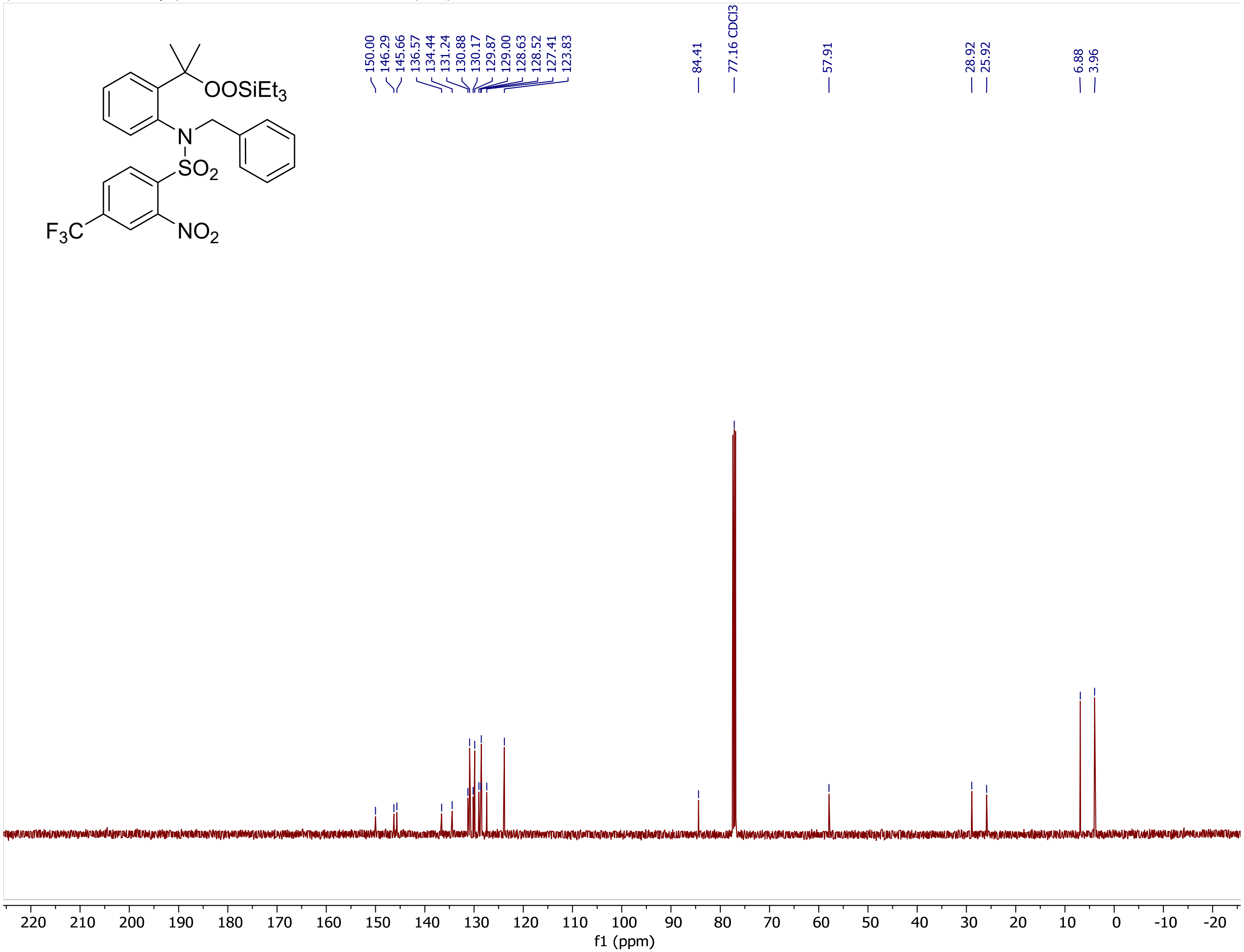

^{1}H NMR (500 MHz, C_6D_6) spectrum of 1-Benzyl-3,3-dimethyl-1,3-dihydrobenzo[*c*]isoxazole (**60**)

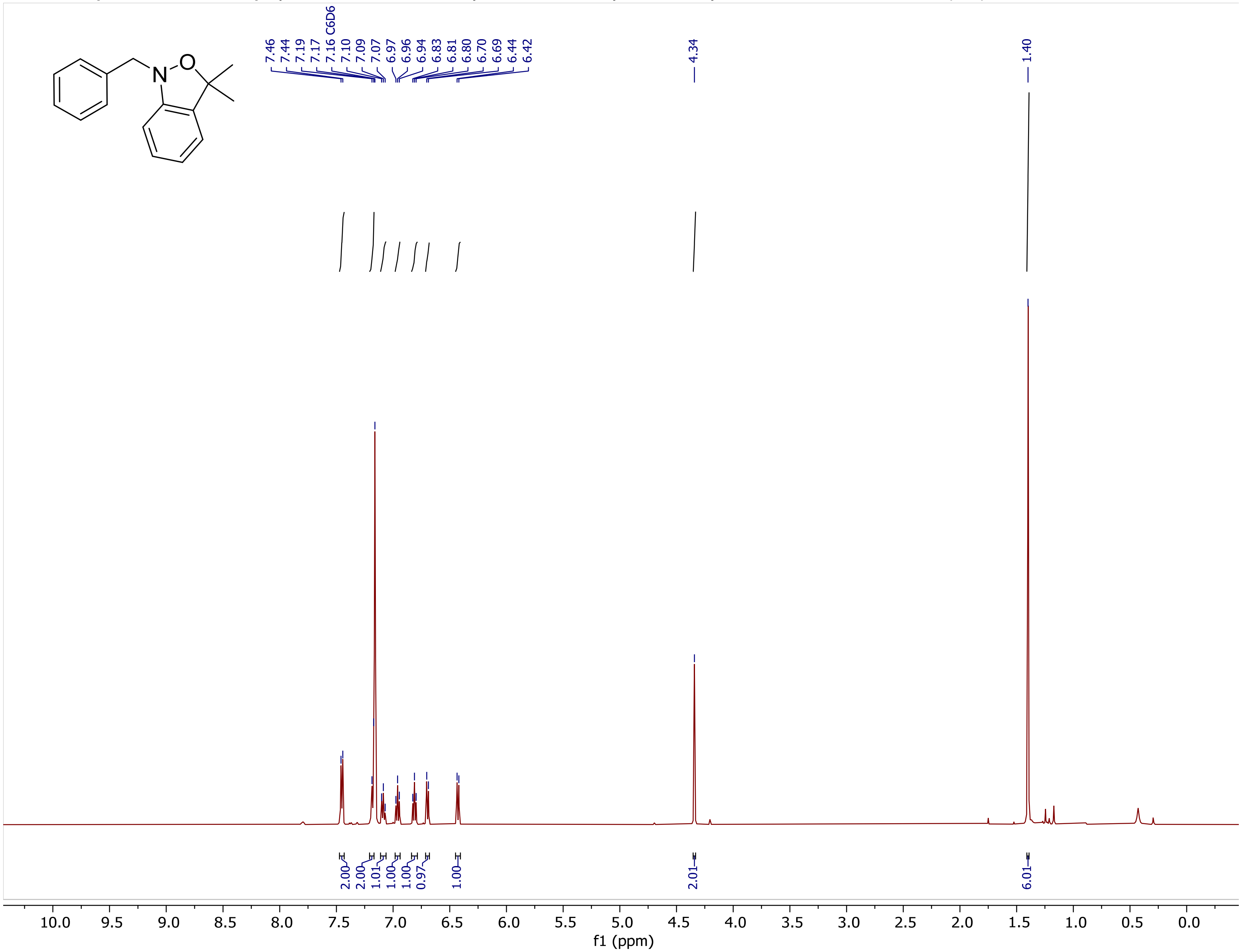

$^{13}C\{^1H\}$ **NMR (126 MHz, C_6D_6)** spectrum of 1-Benzyl-3,3-dimethyl-1,3-dihydrobenzo[*c*]isoxazole (**60**)

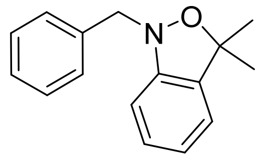

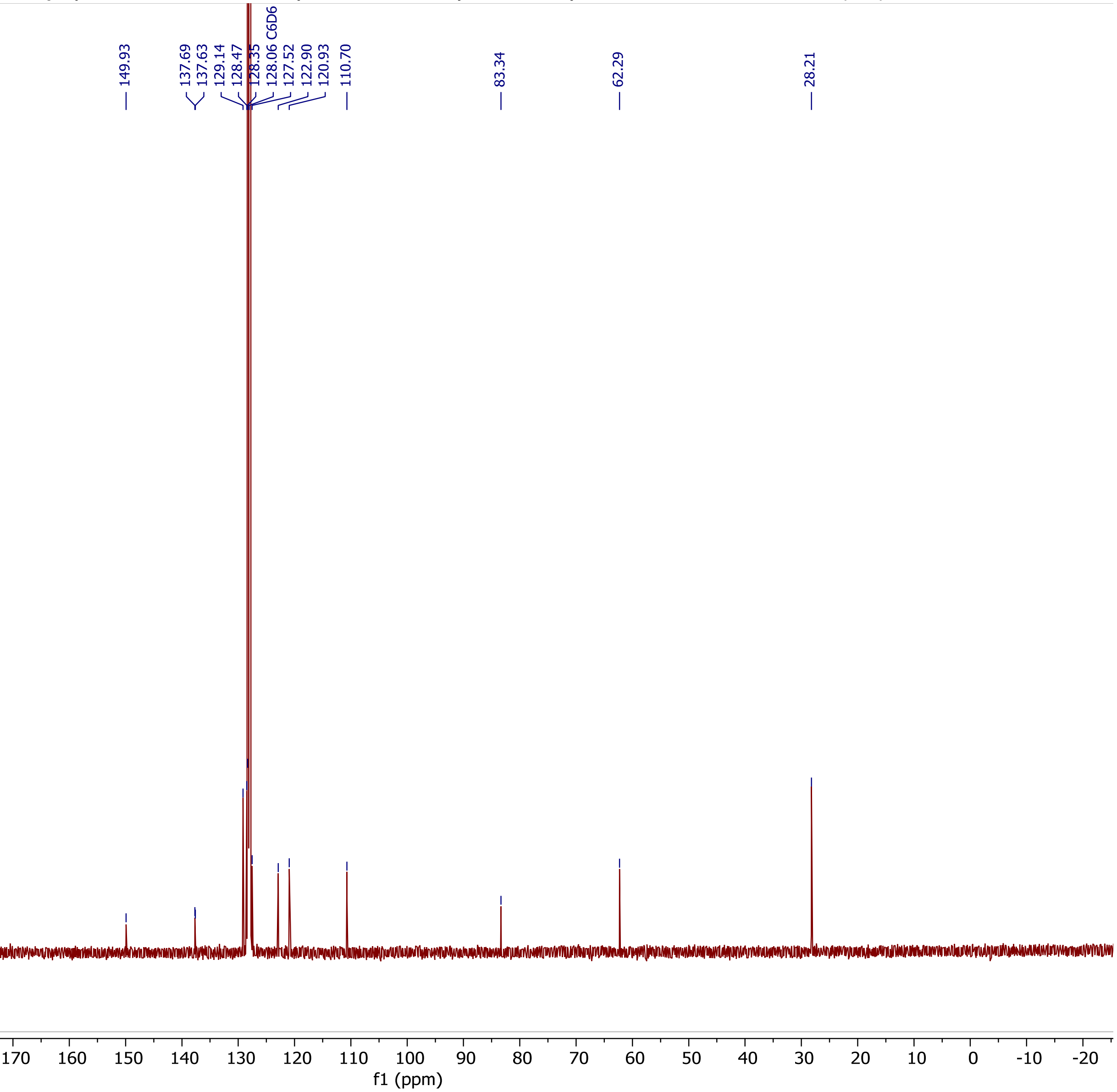

HSQC NMR (500 MHz, C_6D_6) spectrum of 1-Benzyl-3,3-dimethyl-1,3-dihydrobenzo[*c*]isoxazole (**60**)

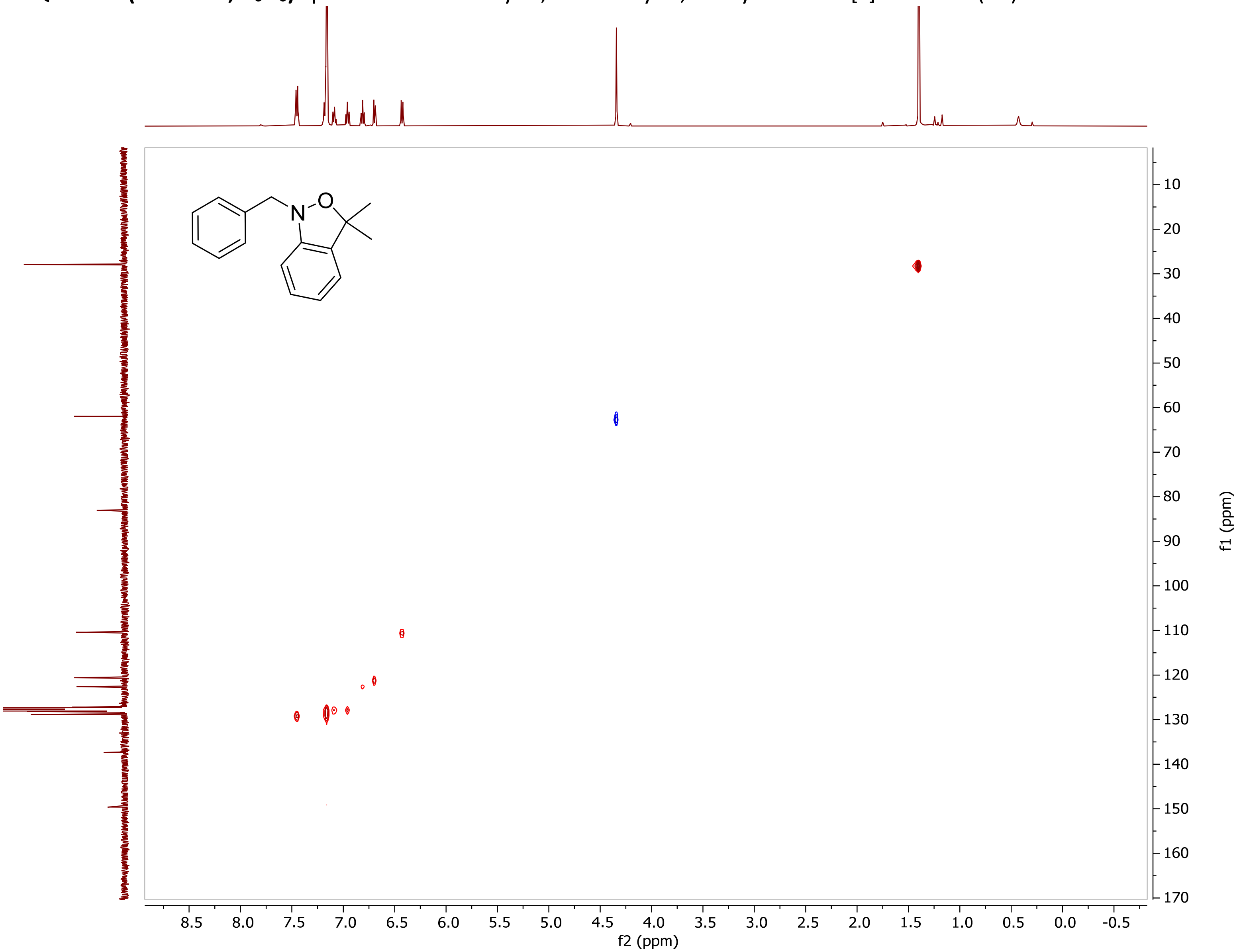

^{1}H NMR (500 MHz, $CDCl_3$) spectrum of 2-Nitro-4-(trifluoromethyl)phenol (**61**)

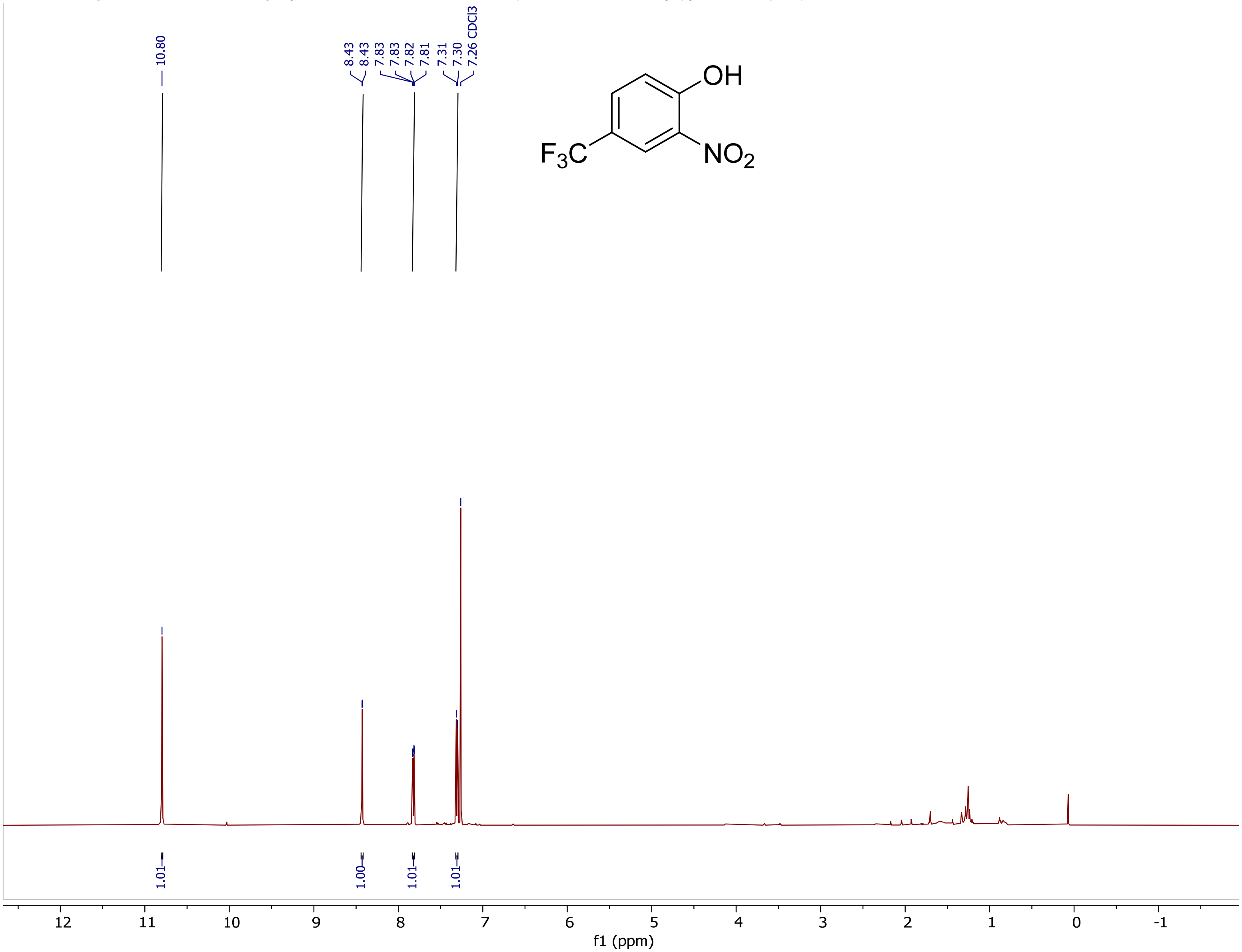

^{13}C {^{1}H, ^{19}F} NMR (126 MHz, $CDCl_3$) spectrum of 2-Nitro-4-(trifluoromethyl)phenol (**61**)

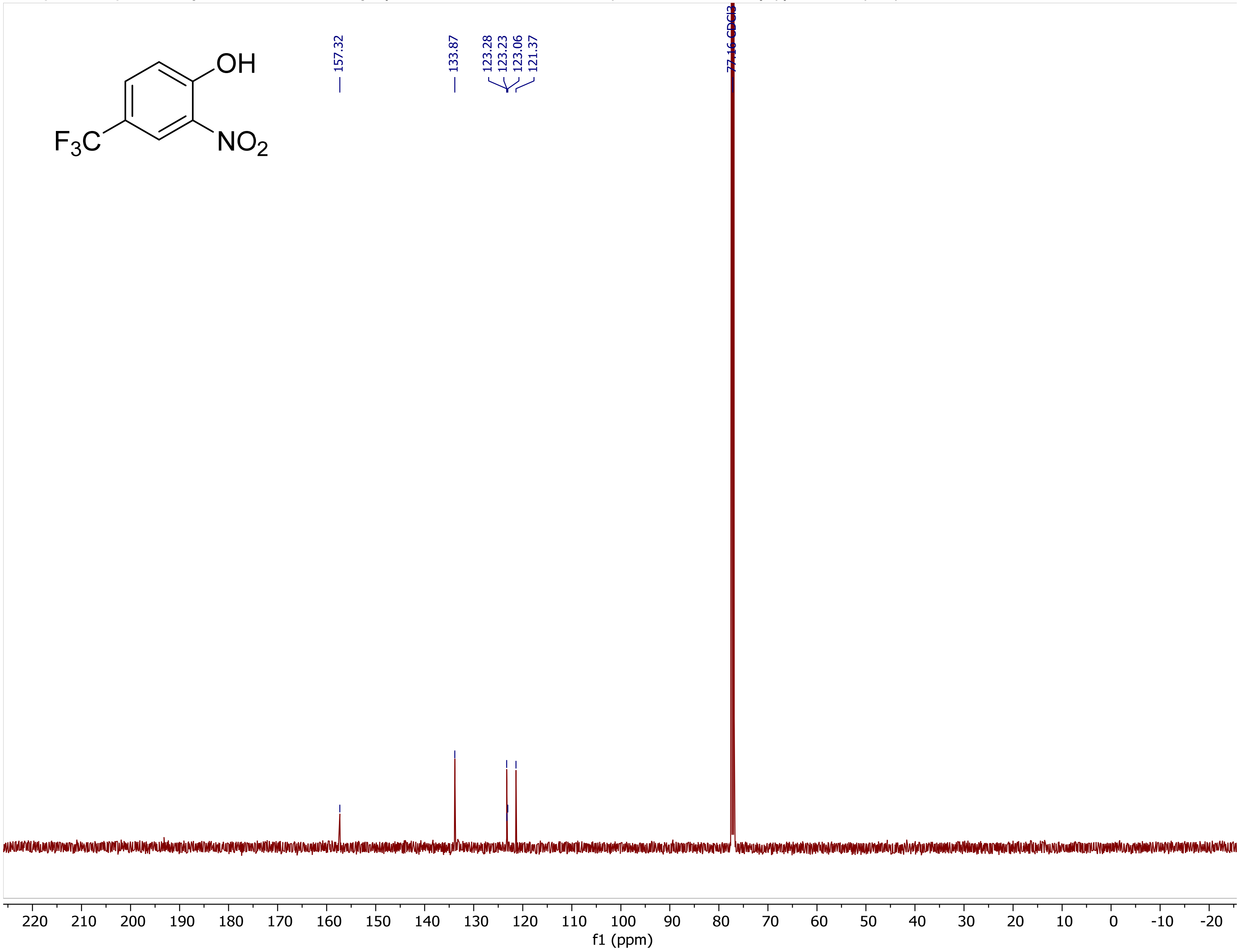

^{13}C {^{1}H} NMR (126 MHz, $CDCl_3$) spectrum of 2-Nitro-4-(trifluoromethyl)phenol (**61**)

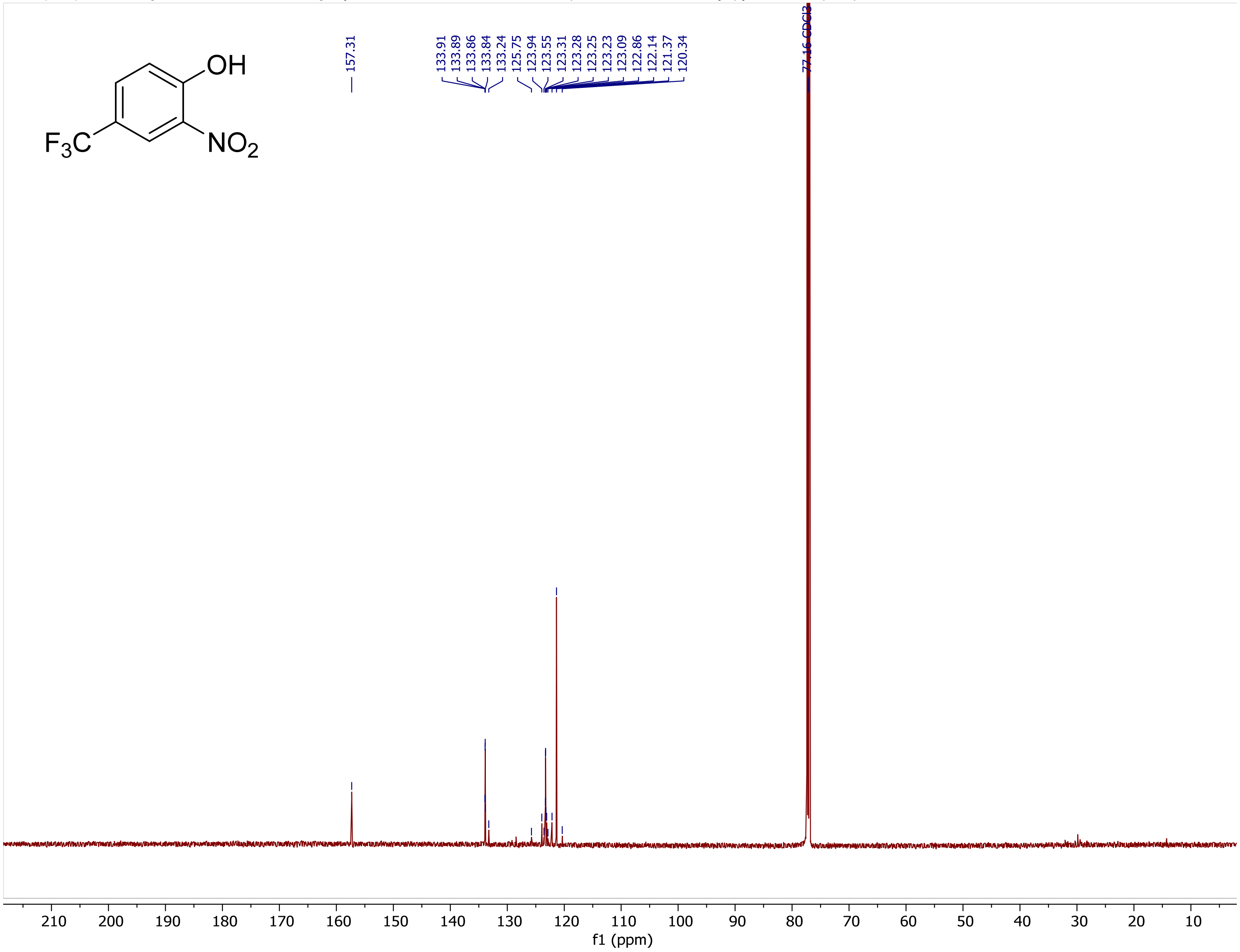

^{19}F NMR (470 MHz, CDCl$_3$) spectrum of 2-Nitro-4-(trifluoromethyl)phenol (**61**)

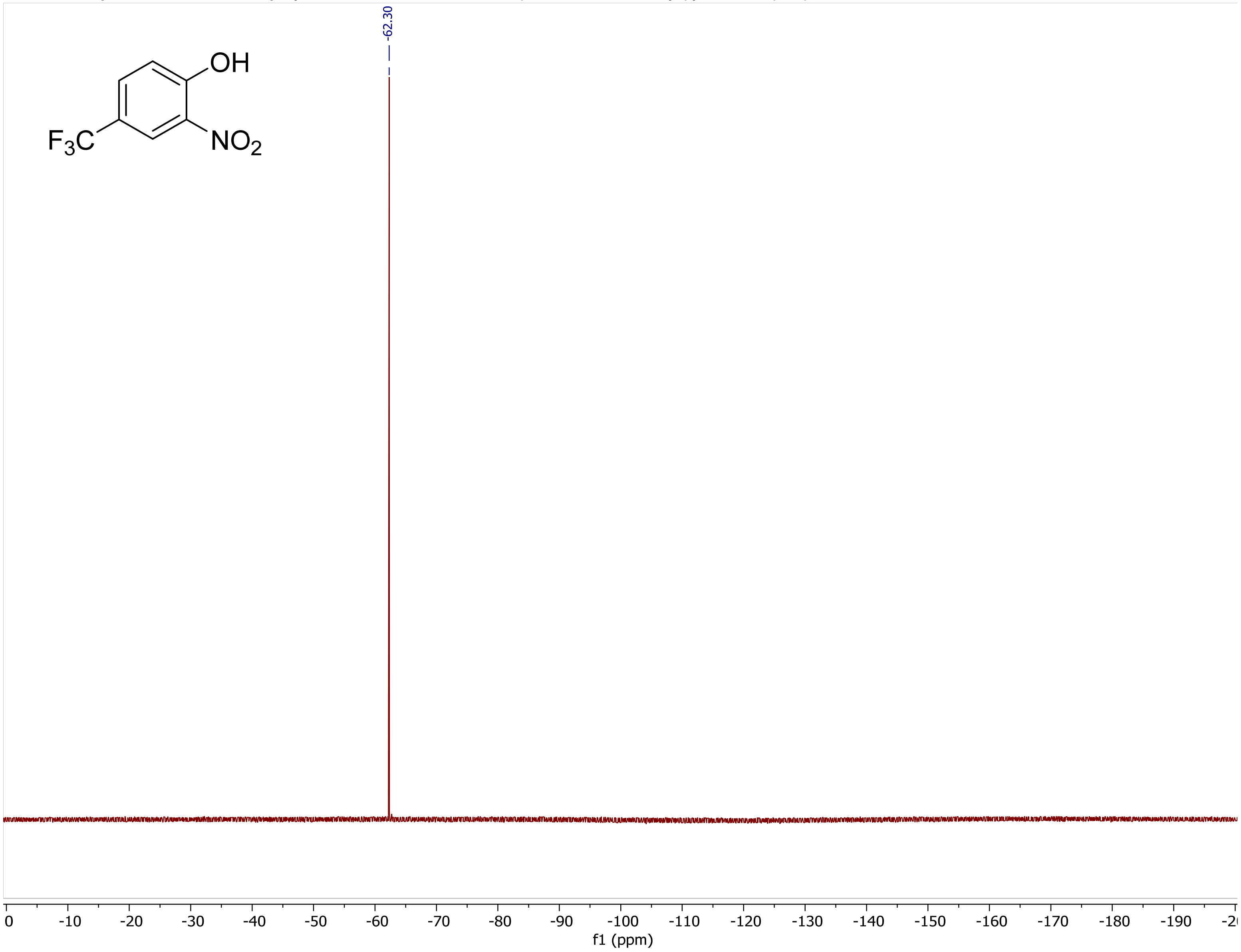

HSQC NMR (500 MHz, CDCl₃) spectrum of 2-Nitro-4-(trifluoromethyl)phenol (**61**)

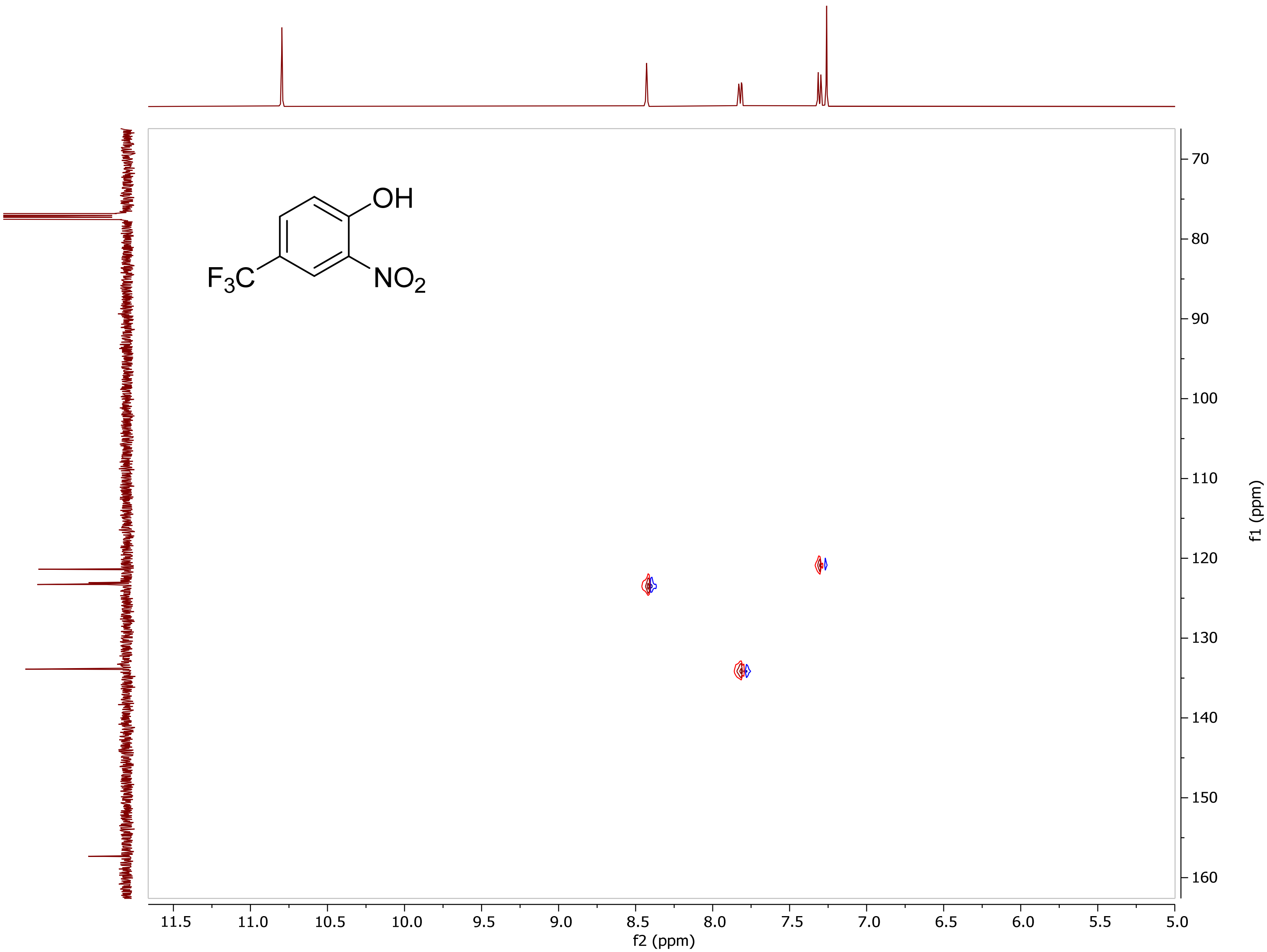

^{1}H NMR (500 MHz, $CDCl_3$) spectrum of *N*-(3-Benzyloxy)propyl)2-nitro-*N*-(2-(prop-1-en-2-yl)phenyl)-4-(trifluoromethyl)benzenesulfonamide (**7d**)

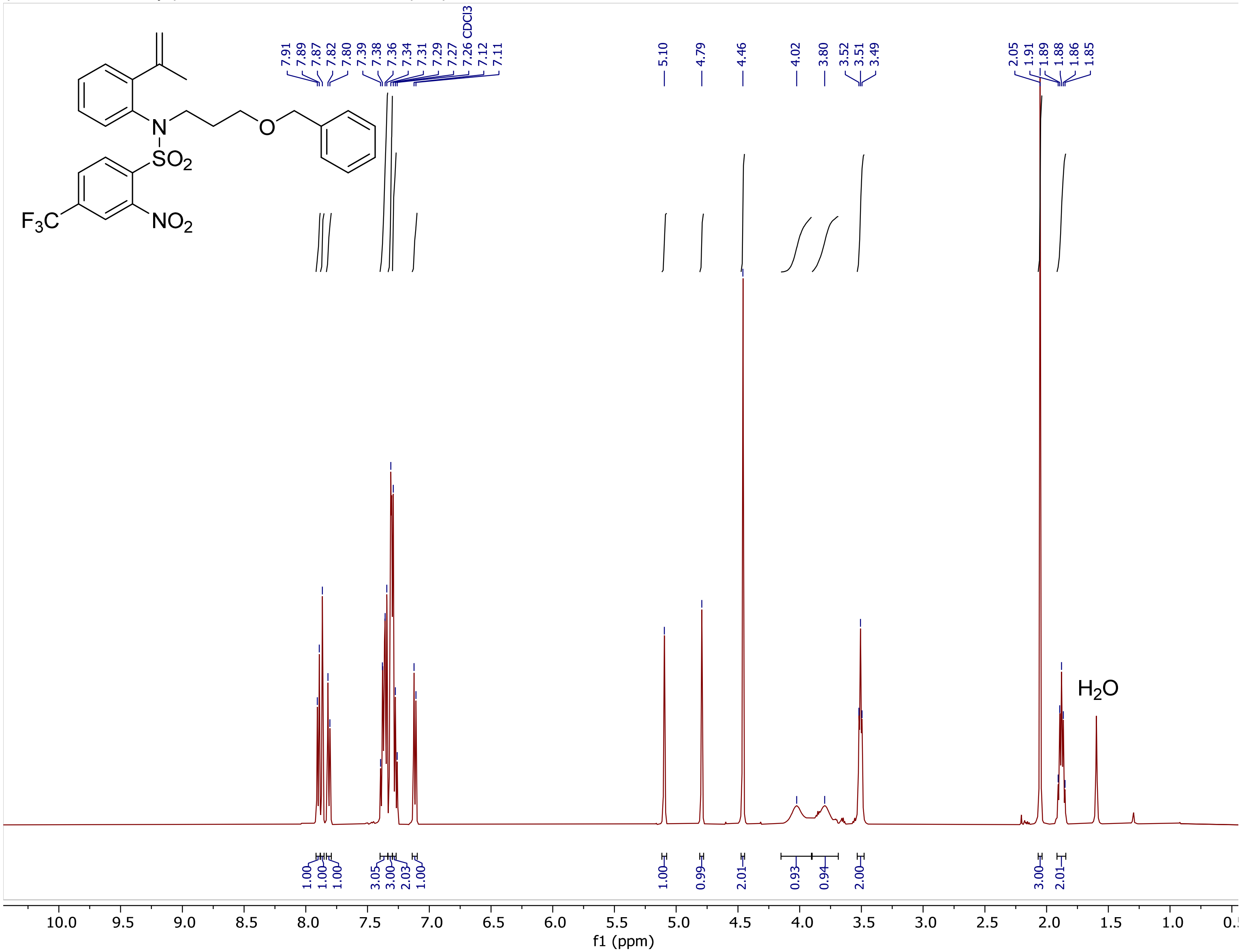

^{13}C {^{1}H} NMR (126 MHz, $CDCl_3$) spectrum of *N*-(3-Benzyloxy)propyl)2-nitro-*N*-(2-(prop-1-en-2-yl)phenyl)-4-(trifluoromethyl)benzenesulfonamide (**7d**)

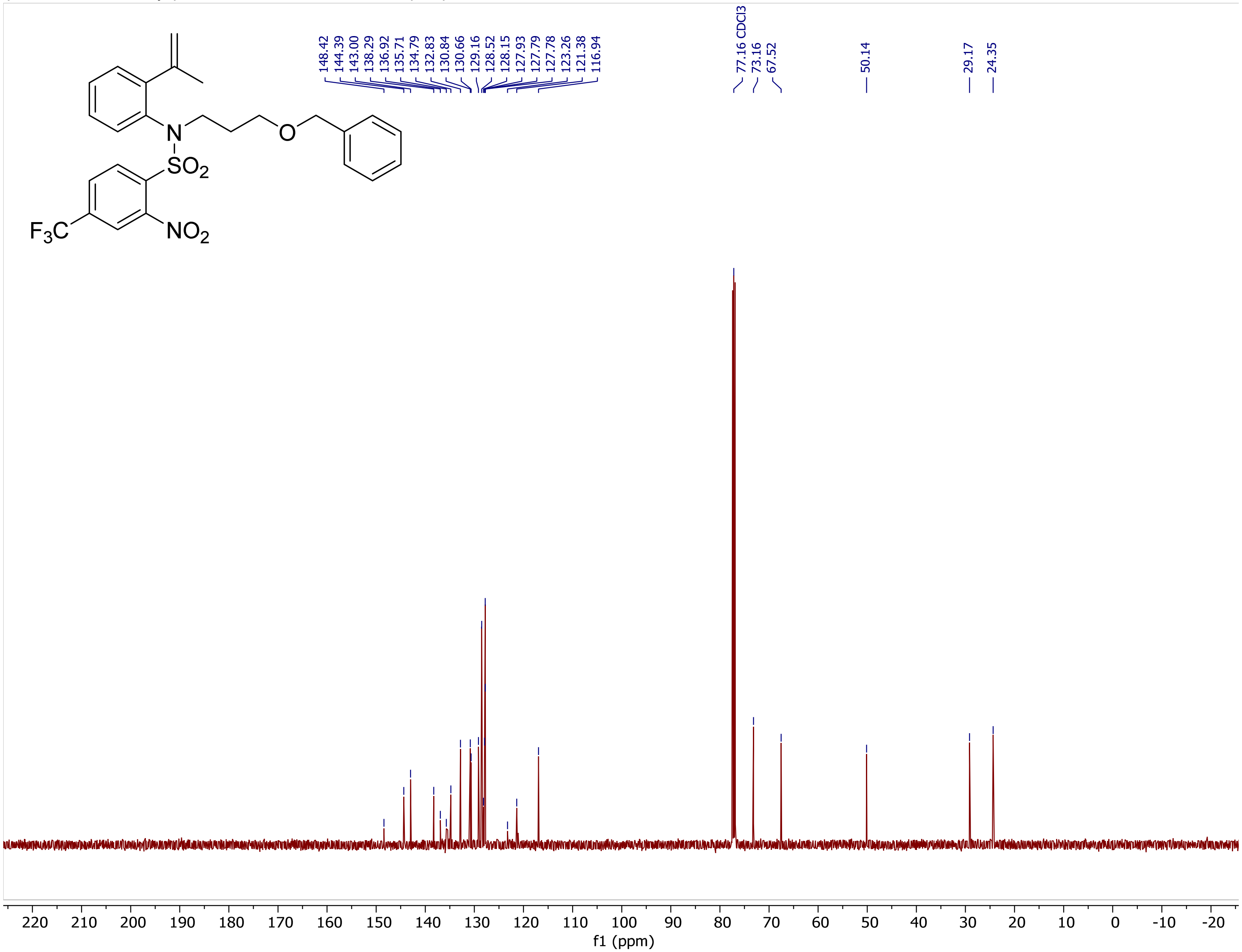

^{1}H NMR (500 MHz, C_6D_6) spectrum of *N*-(3-Benzyloxy)propyl)2-nitro-*N*-(2-(2-((triethylsilyl)peroxy)propan-2-yl)phenyl)-4-(trifluoromethyl)benzenesulfonamide (**65**)

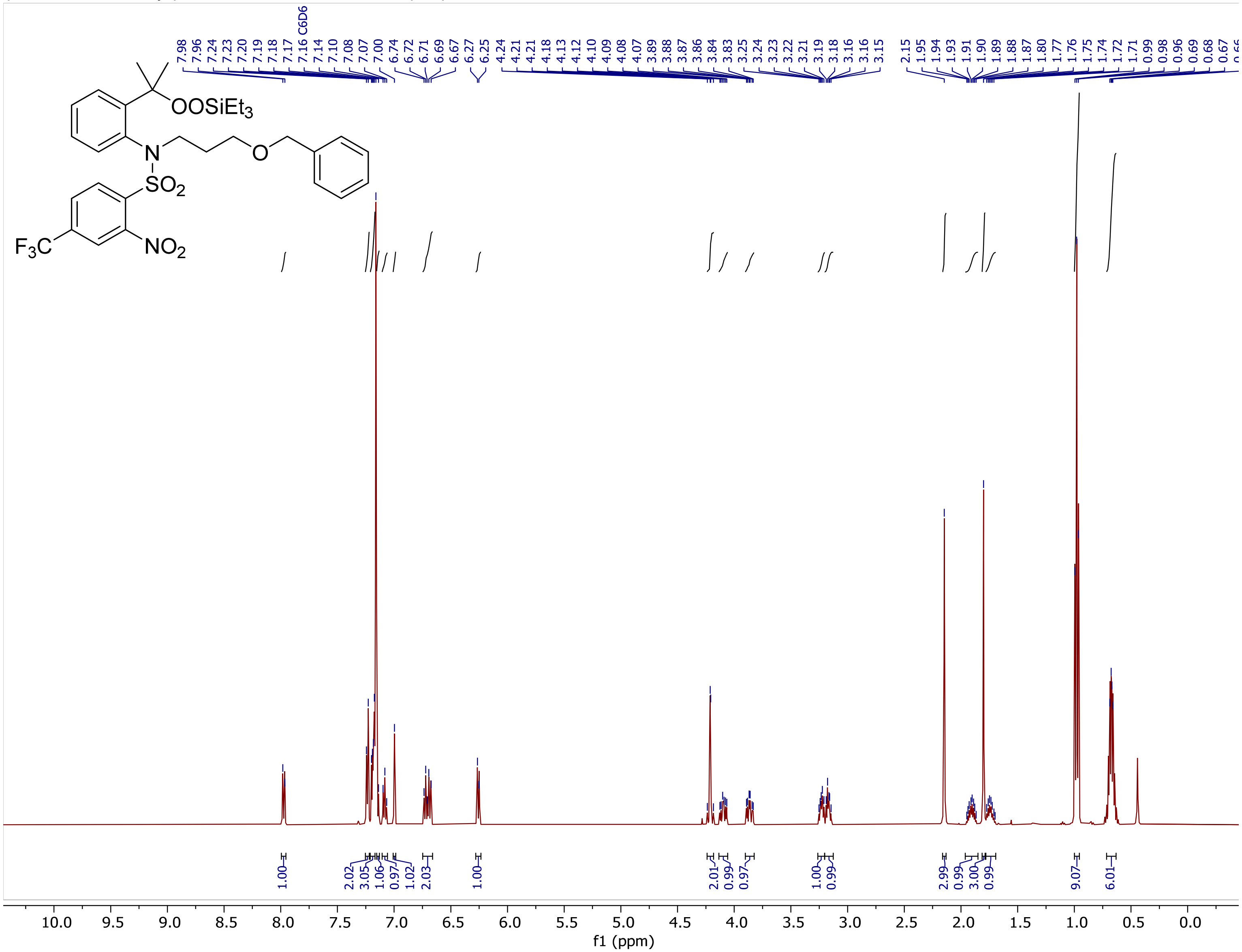

^{13}C {^{1}H, ^{19}F} NMR (126 MHz, C_6D_6) spectrum of *N*-(3-Benzyloxy)propyl)2-nitro-*N*-(2-(2-((triethylsilyl)peroxy)propan-2-yl)phenyl)-4-(trifluoromethyl)benzenesulfonamide (**65**)

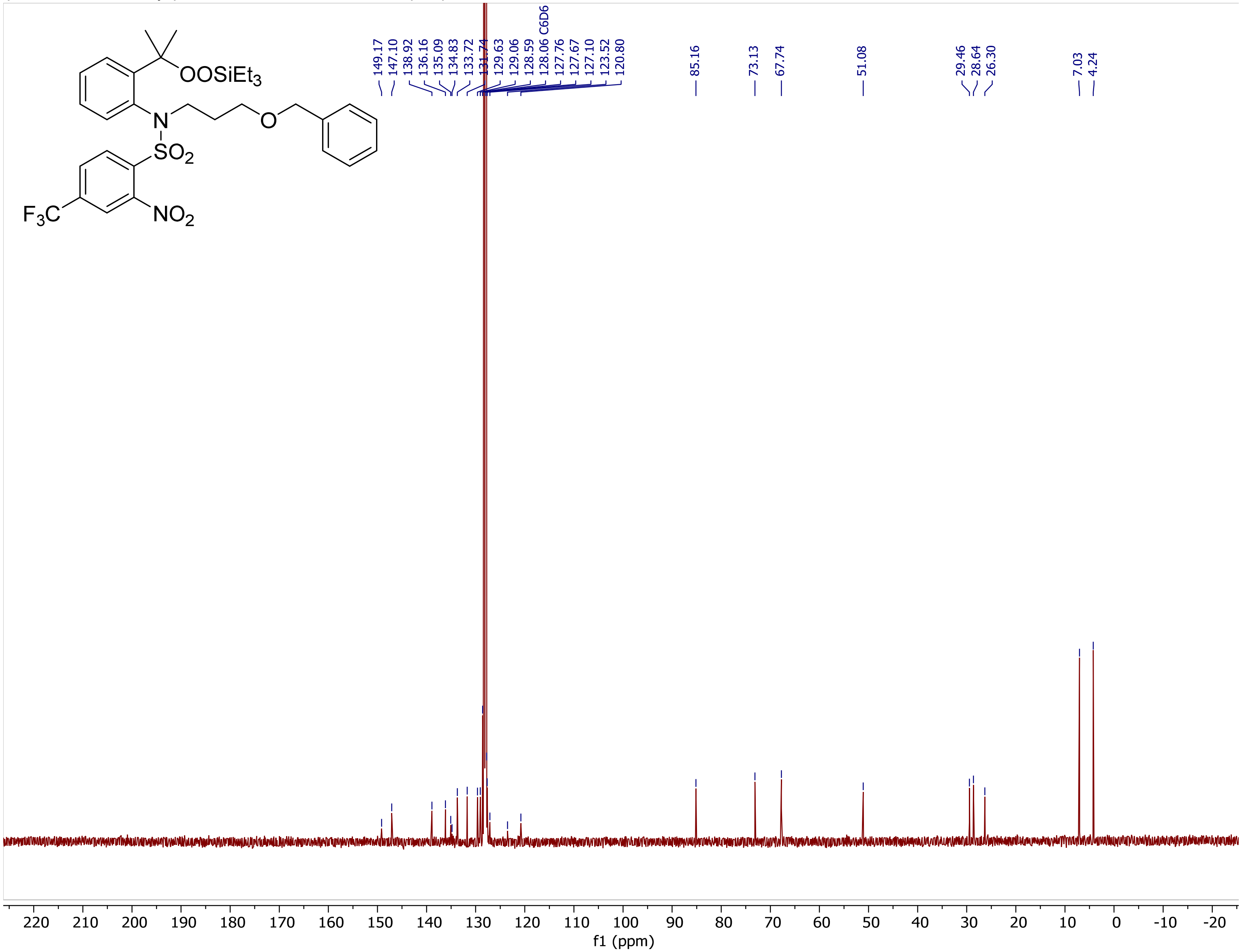

^{1}H NMR (500 MHz, C_6D_6) spectrum of 1-(3-(Benzyloxy)propyl)-3,3-dimethyl-1,3-dihydrobenzo[*c*]isoxazole (**66**)

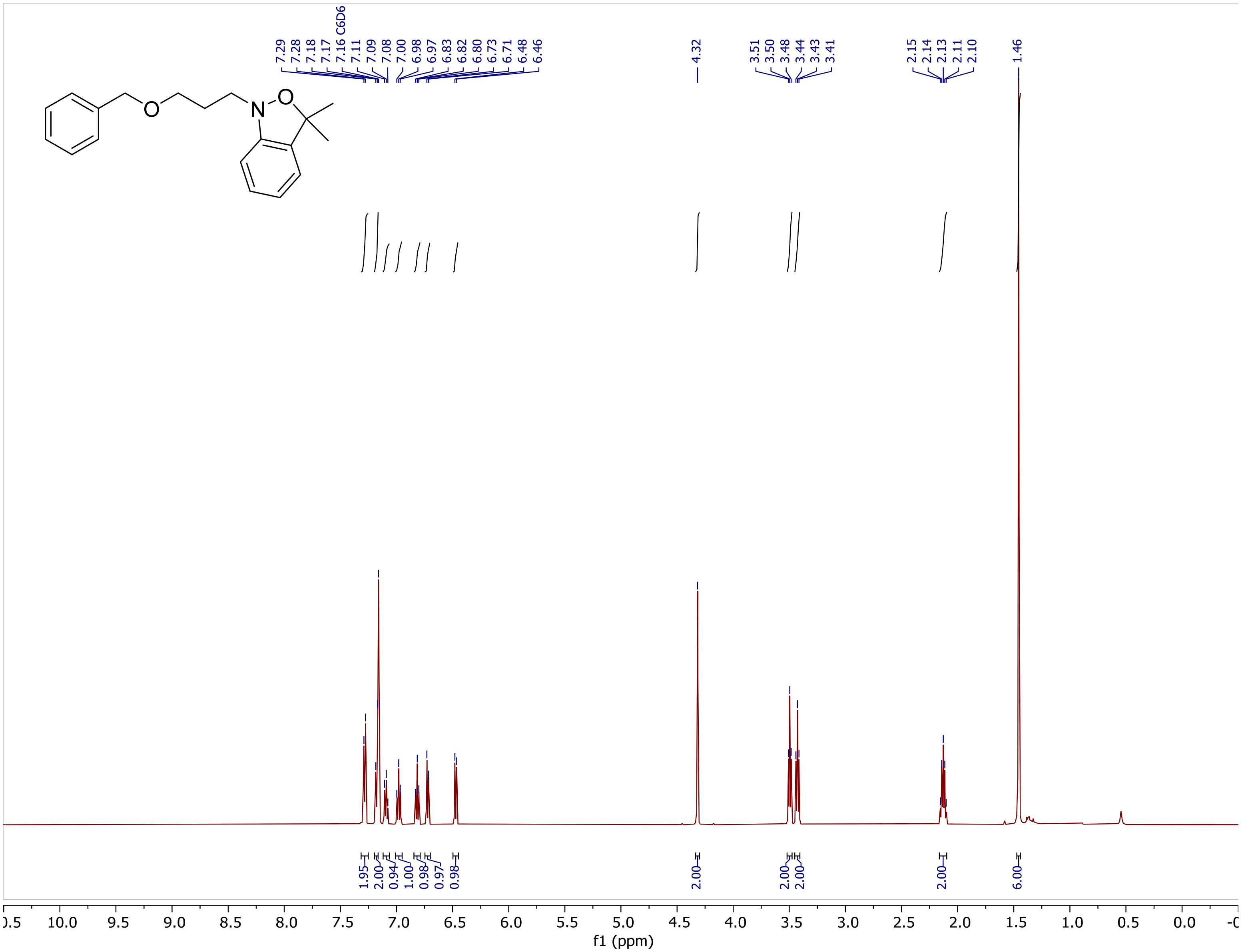

$^{13}C\ \{^{1}H\}$ NMR (126 MHz, C_6D_6) spectrum of 1-(3-(Benzyloxy)propyl)-3,3-dimethyl-1,3-dihydrobenzo[*c*]isoxazole (**66**)

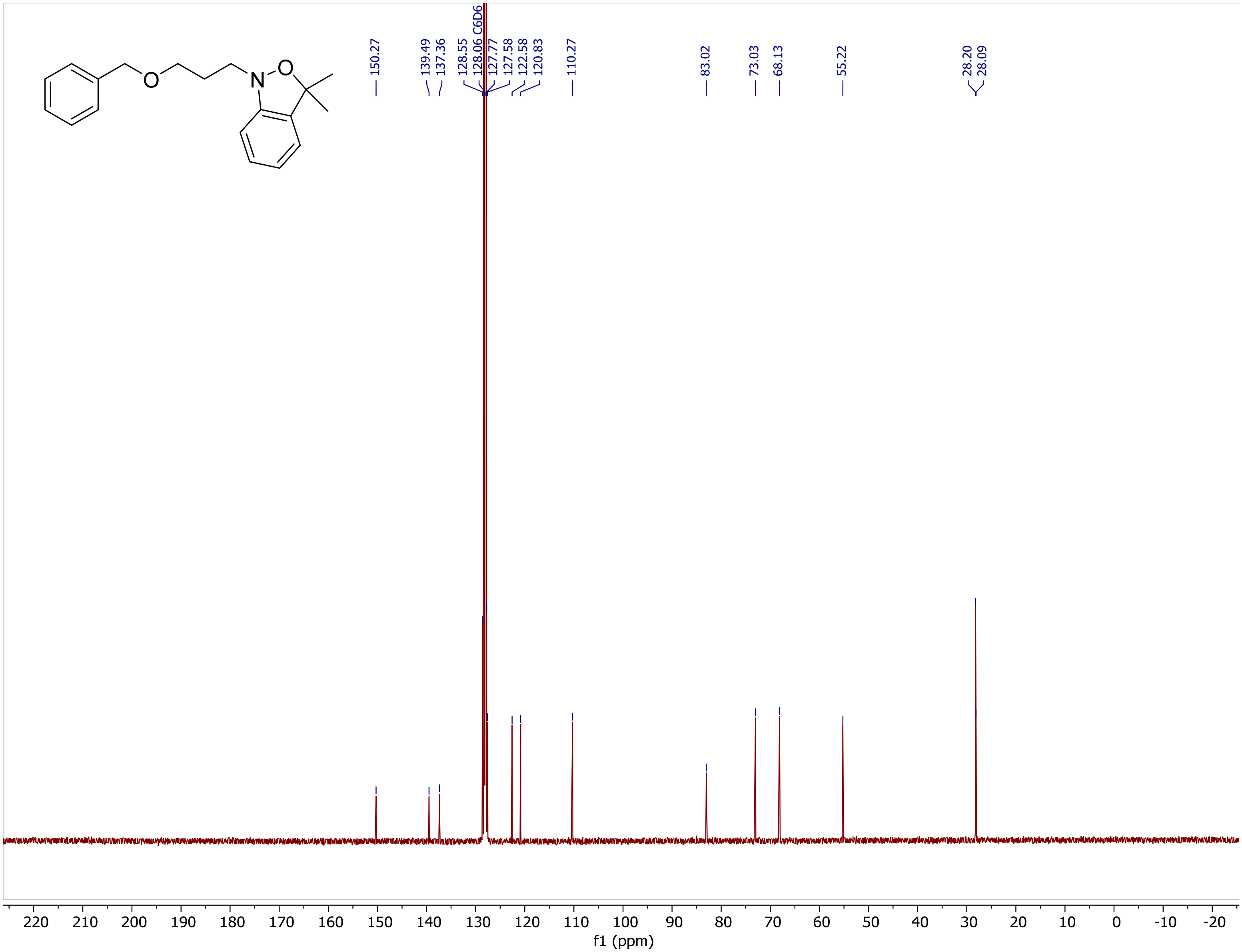

HSQC NMR (500 MHz, C_6D_6) spectrum of 1-(3-(Benzyloxy)propyl)-3,3-dimethyl-1,3-dihydrobenzo[*c*]isoxazole (**66**)

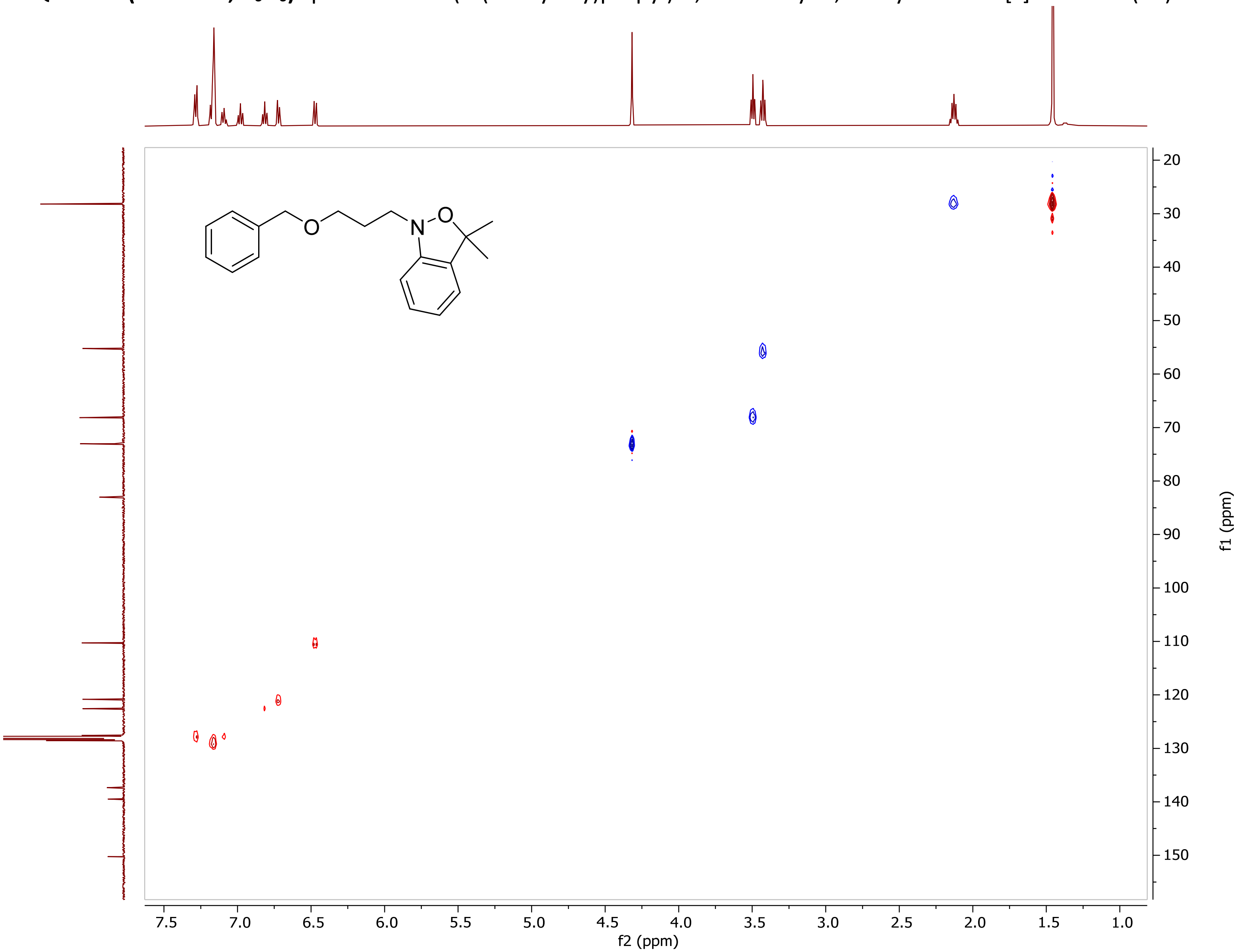

^{1}H NMR (500 MHz, $CDCl_3$) spectrum of 2-Nitro-4-(trifluoromethyl)-*N*-(2-vinylphenyl)benzenesulfonamide (**S8**)

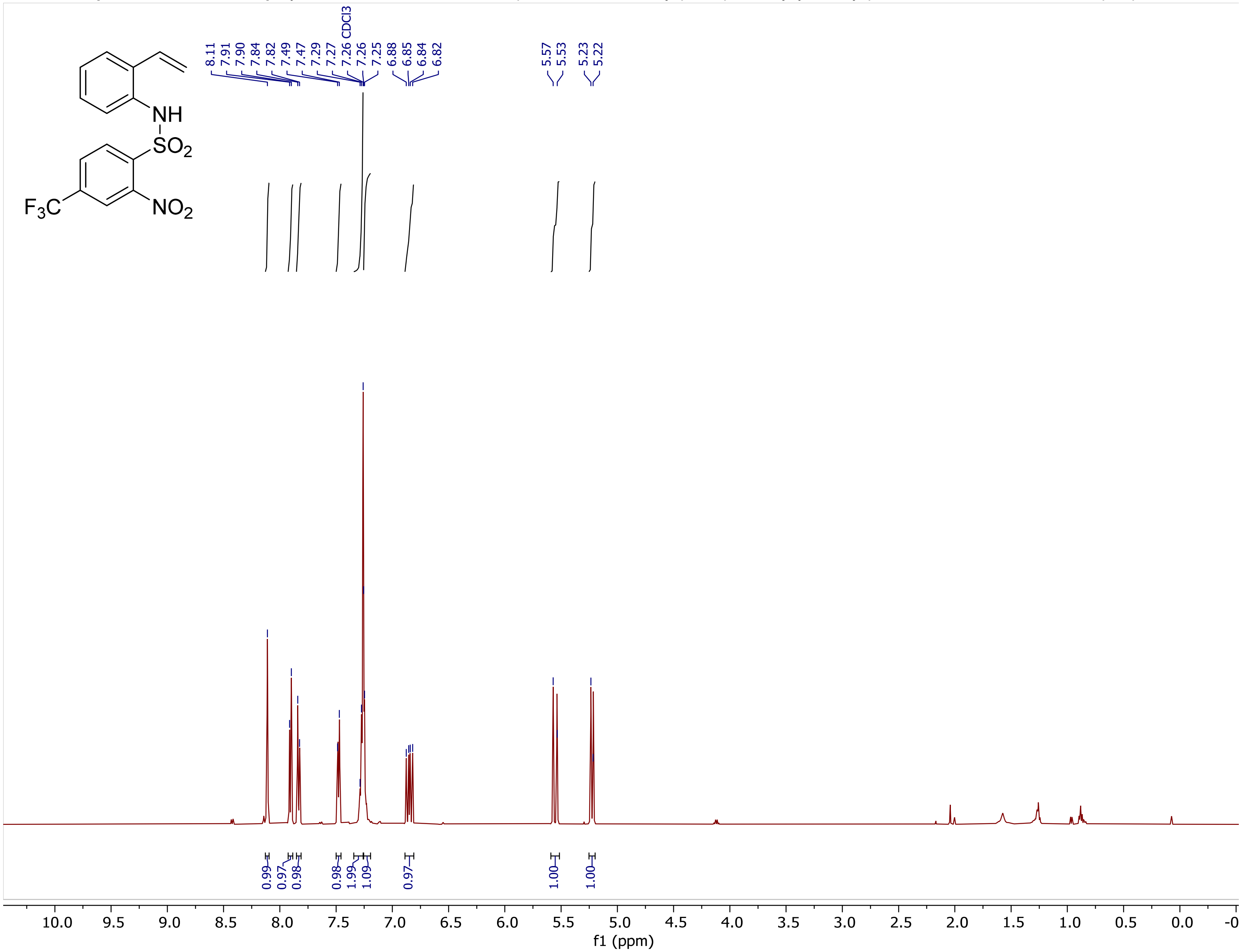

^{13}C {^{1}H} NMR (126 MHz, CDCl$_3$) spectrum of 2-Nitro-4-(trifluoromethyl)-*N*-(2-vinylphenyl)benzenesulfonamide (**S8**)

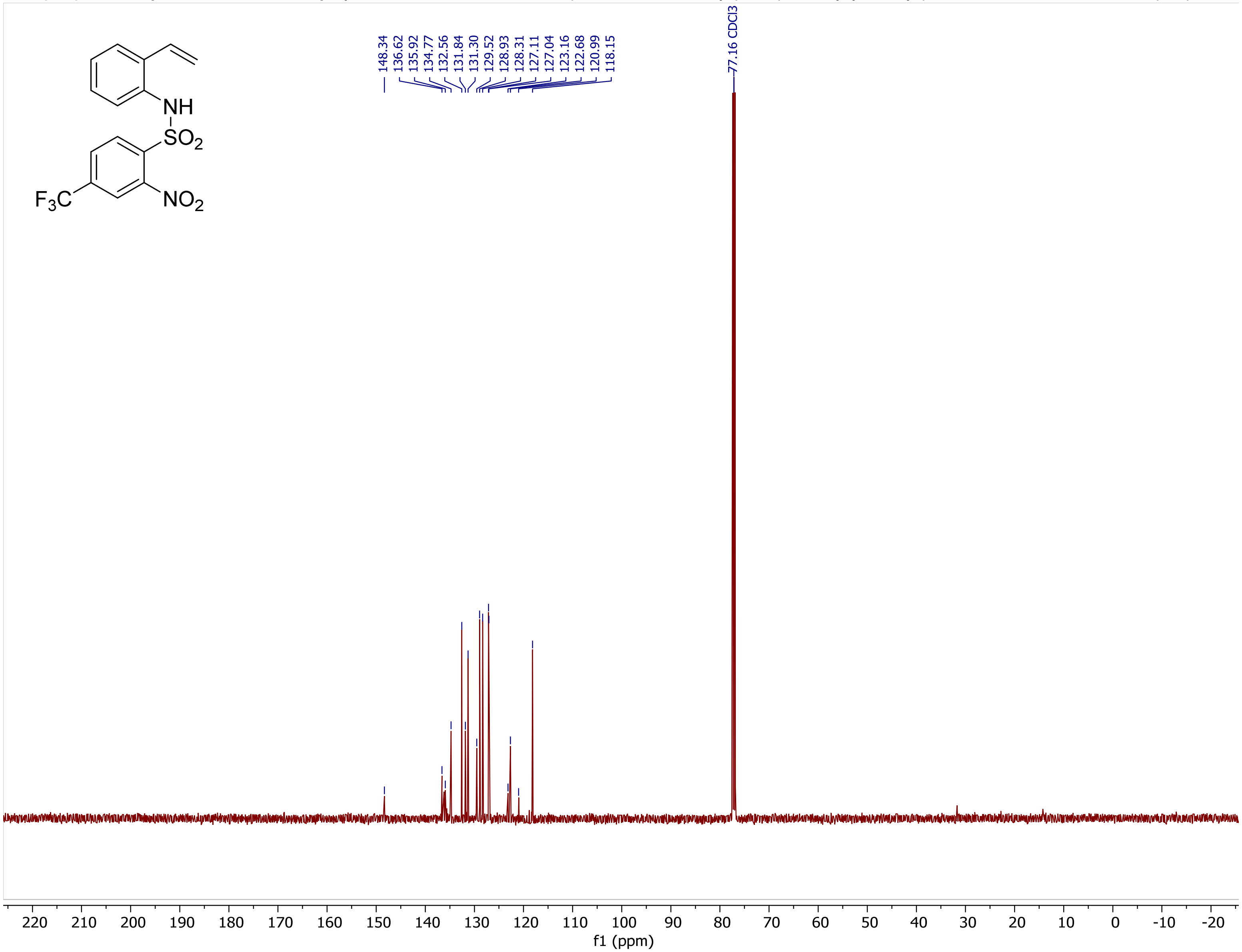

^{1}H NMR (500 MHz, CDCl$_3$) spectrum of *N*-(2-Iodobenzyl)-2-nitro-4-(trifluoromethyl)-*N*-(2-vinylphenyl) benzenesulfonamide (**8a**)

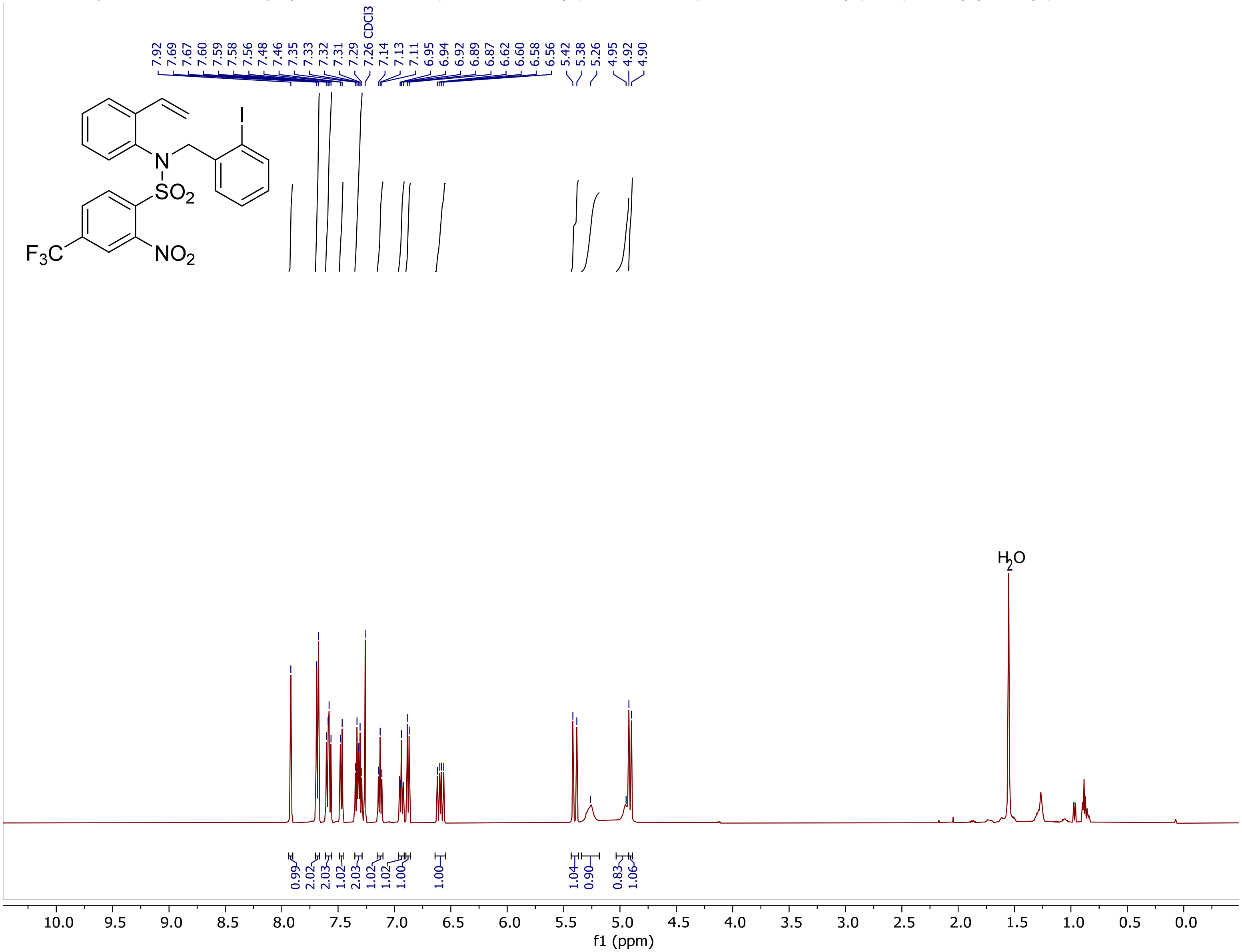

^{13}C {^{1}H} NMR (126 MHz, $CDCl_3$) spectrum of *N*-(2-Iodobenzyl)-2-nitro-4-(trifluoromethyl)-*N*-(2-vinylphenyl) benzenesulfonamide (**8a**)

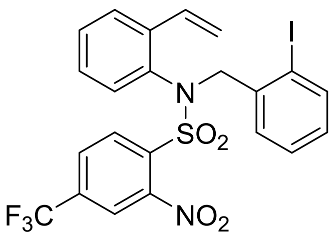

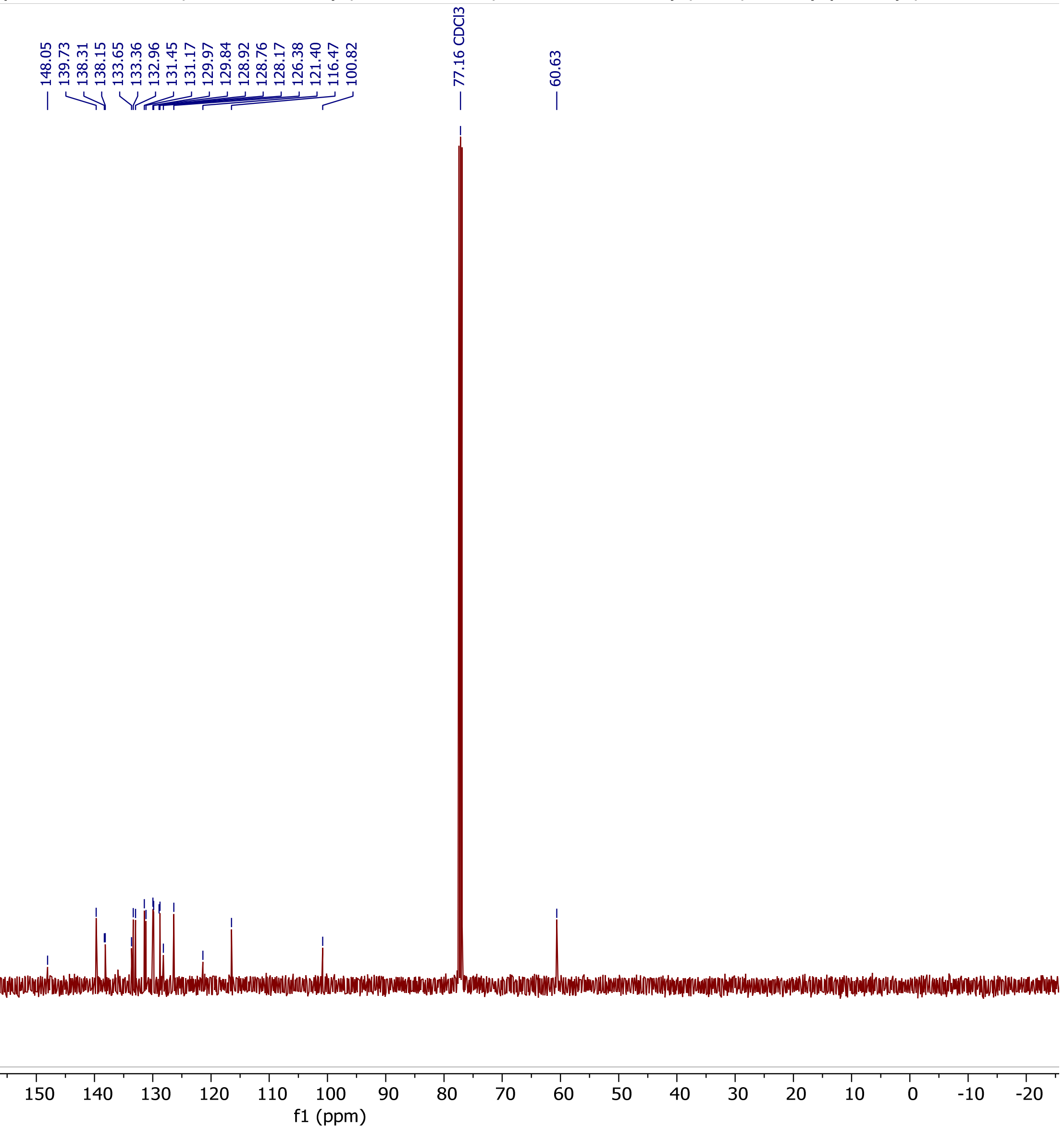

^{1}H NMR (500 MHz, CDCl$_3$) spectrum of *N*-(2-Iodobenzyl)-2-nitro-N-(2-(1-((triethylsilyl)peroxy)ethyl)phenyl)-4-(trifluoromethyl)benzenesulfonamide (**67**)

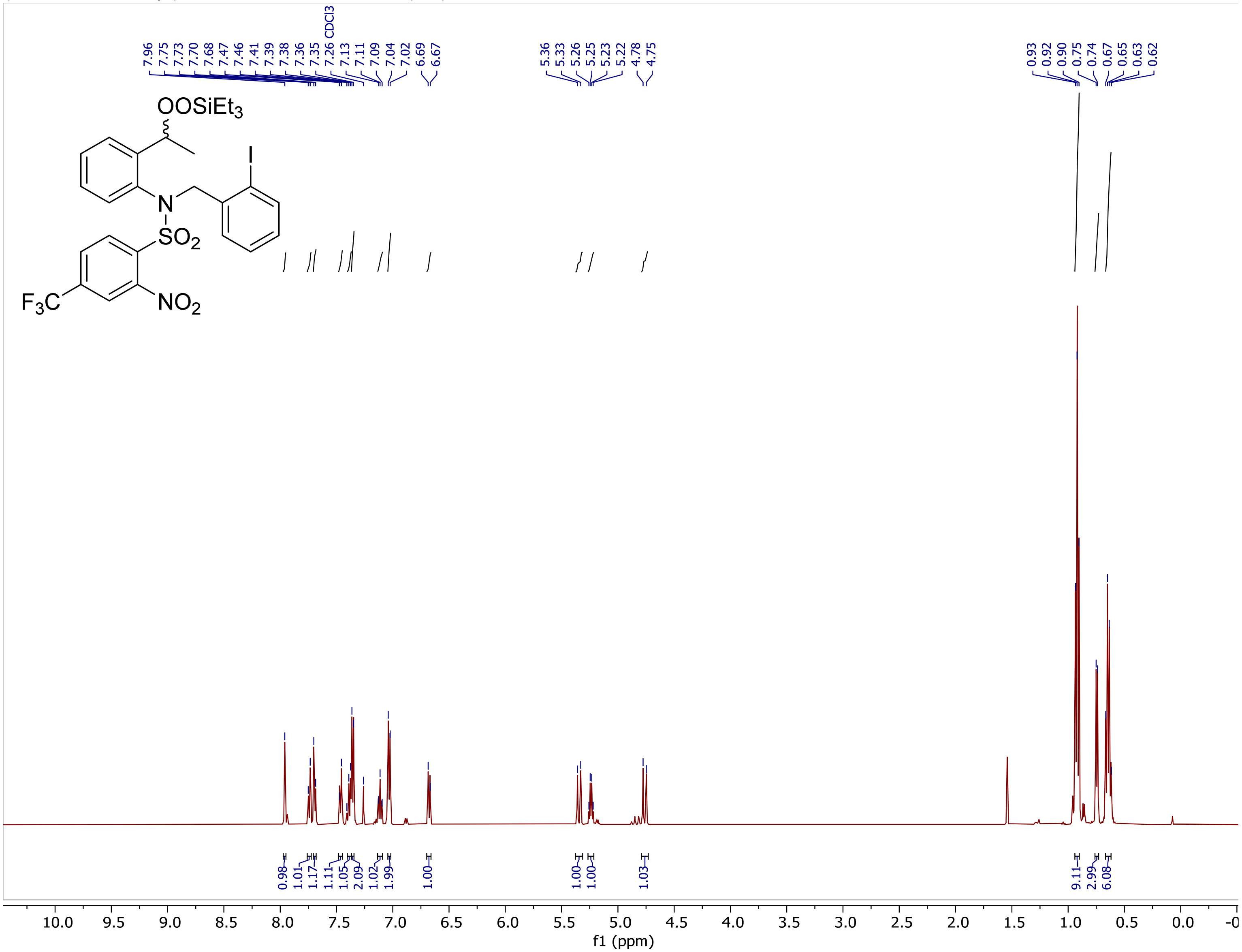

$^{13}C\ \{^{1}H\}$ NMR (126 MHz, $CDCl_3$) spectrum of *N*-(2-Iodobenzyl)-2-nitro-N-(2-(1-((triethylsilyl)peroxy)ethyl)phenyl)-4-(trifluoromethyl)benzenesulfonamide (**67**)

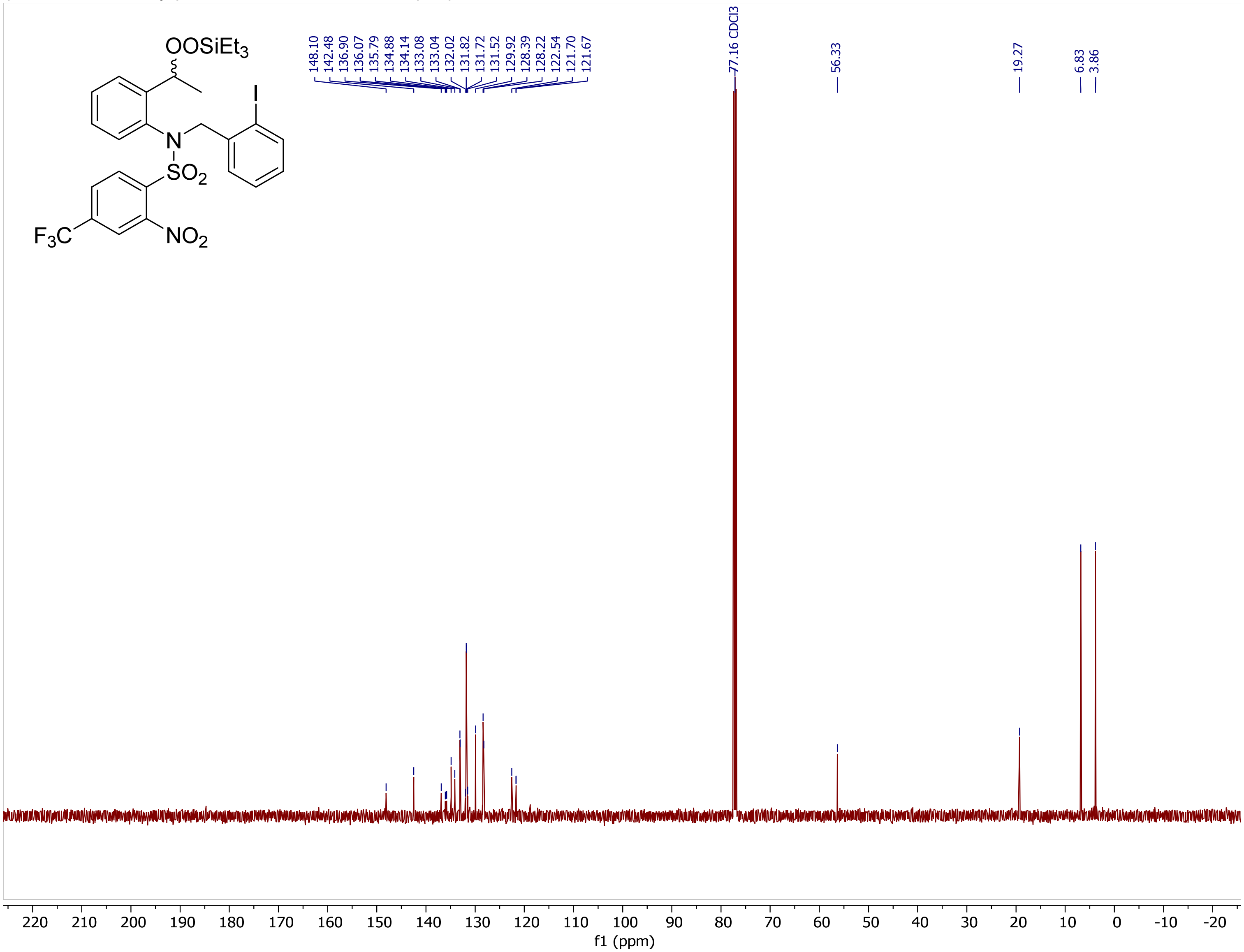

^{1}H NMR (500 MHz, C_6D_6) spectrum of 1-(2-Iodobenzyl)-3-methyl-1,3-dihydrobenzo[*c*]isoxazole (**68**)

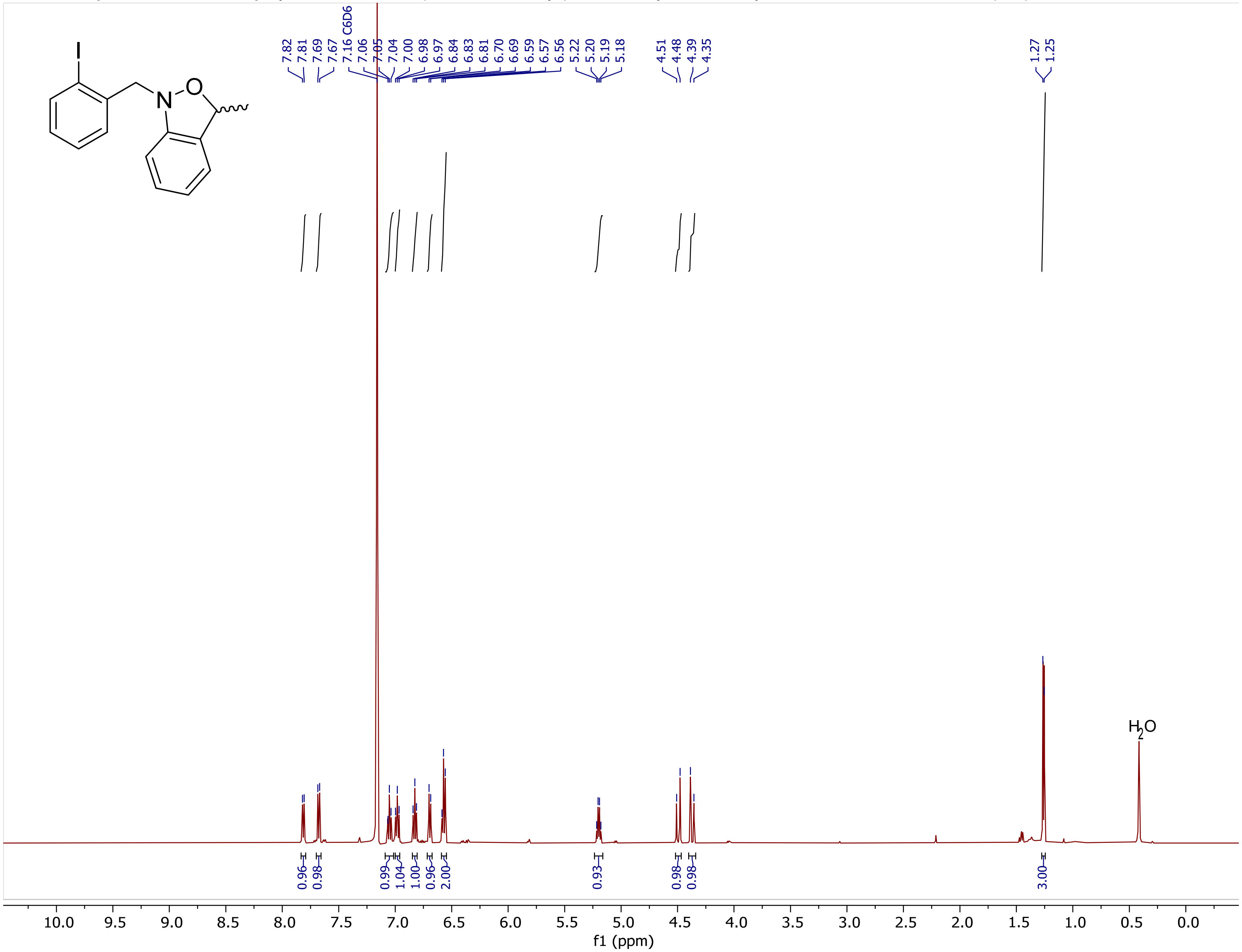

$^{13}C\ \{^1H\}$ NMR (126 MHz, C_6D_6) spectrum of 1-(2-Iodobenzyl)-3-methyl-1,3-dihydrobenzo[*c*]isoxazole (**68**)

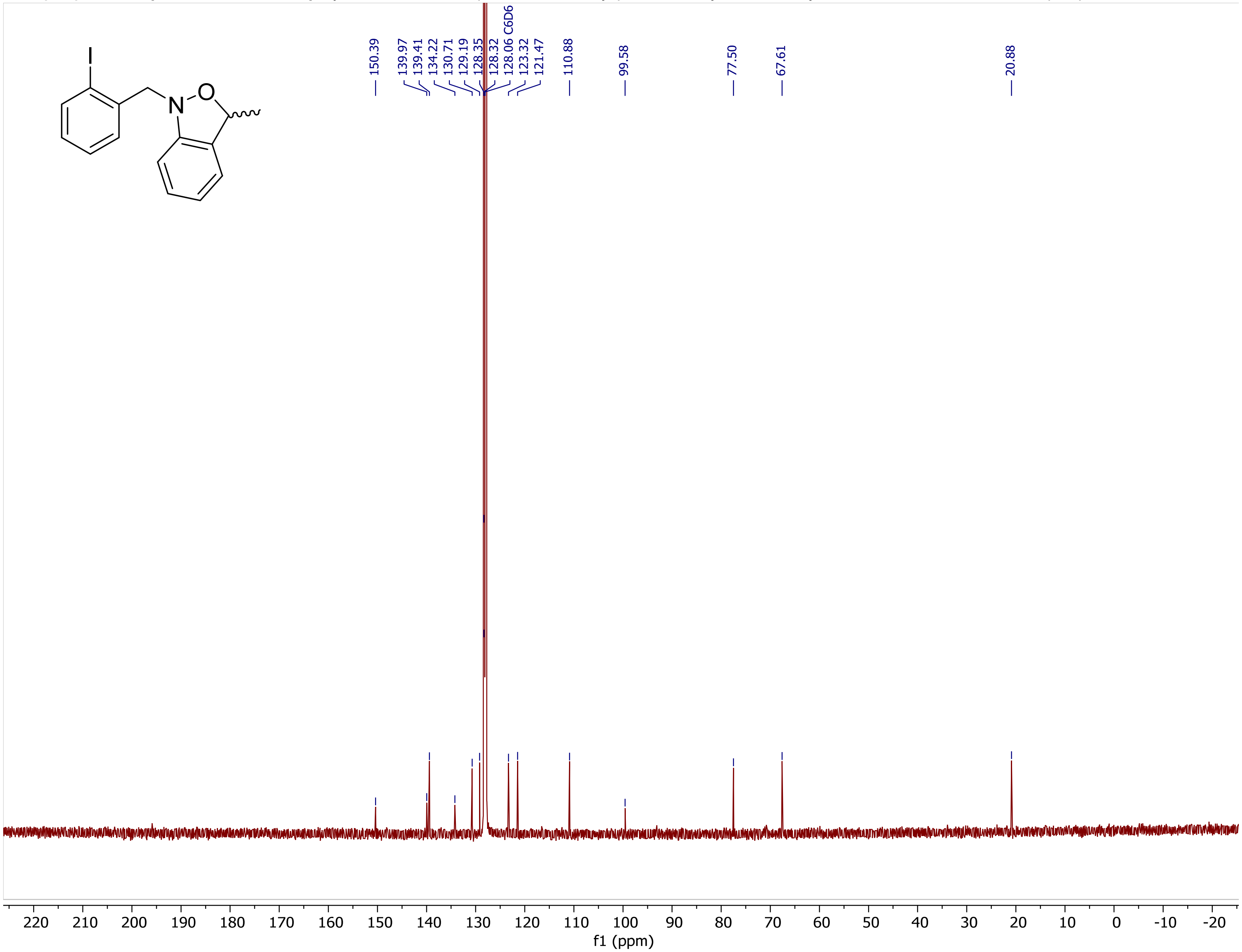

HSQC NMR (500 MHz, C_6D_6) spectrum of 1-(2-Iodobenzyl)-3-methyl-1,3-dihydrobenzo[*c*]isoxazole (**68**)

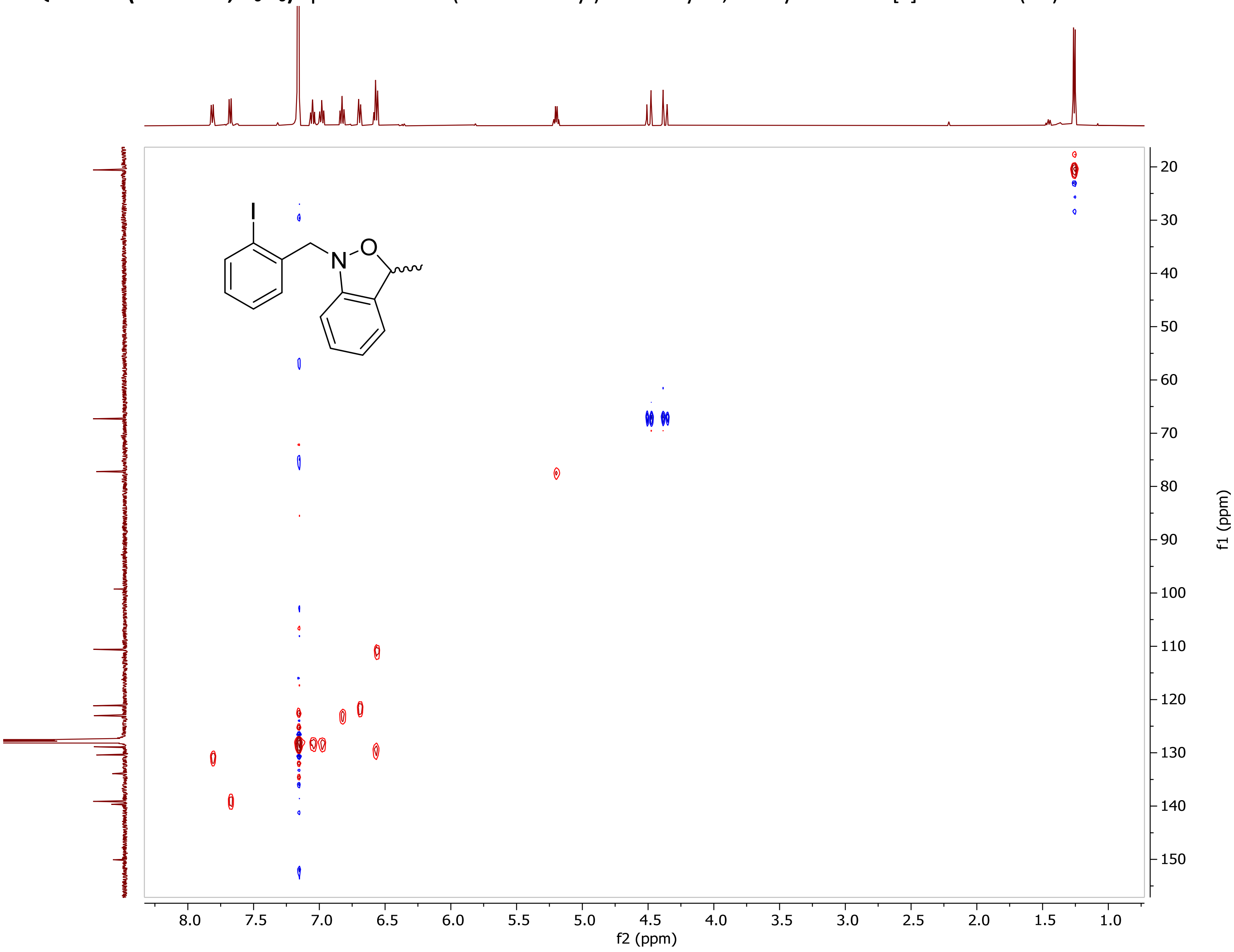

^{1}H NMR (500 MHz, CDCl3) spectrum of 2-Iodobenzylamino-acetophenone (**69**)

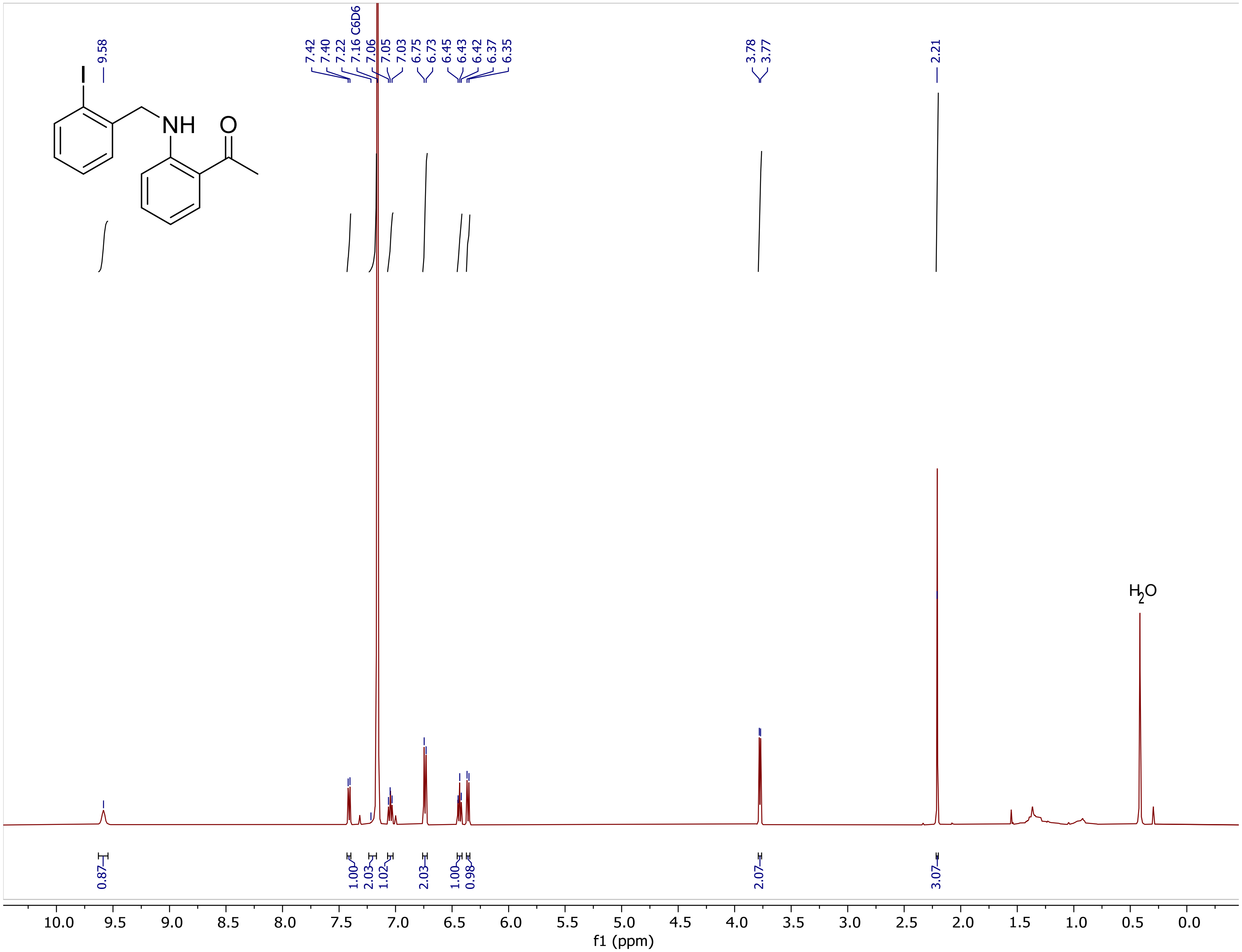

Crude ^{1}H NMR (500 MHz, $CDCl_3$) 2-Allyl-5-bromoindoline (**S9**)

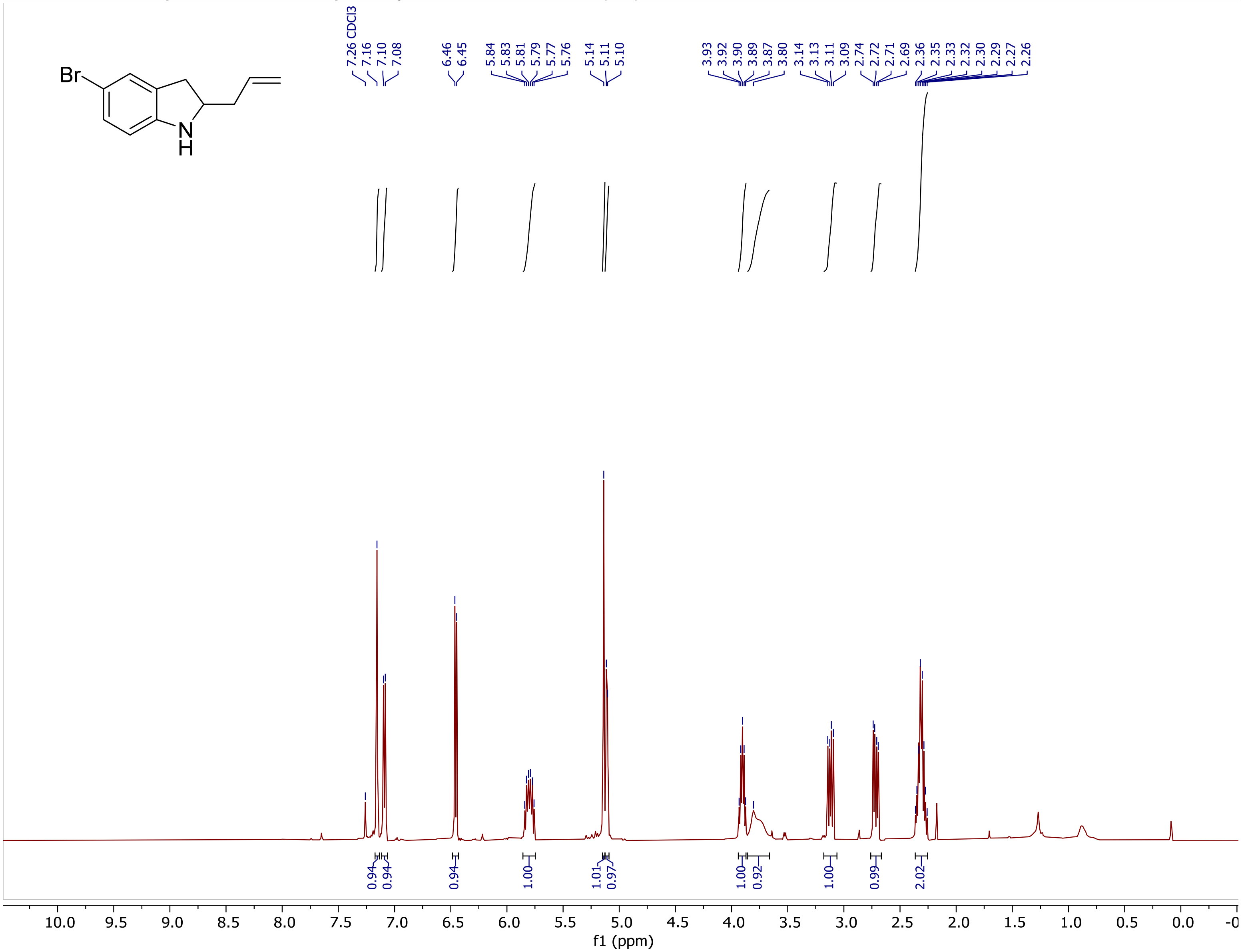

$^{13}C\ \{^{1}H\}$ NMR (126 MHz, $CDCl_3$) 2-Allyl-5-bromoindoline (**S9**)

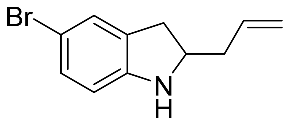

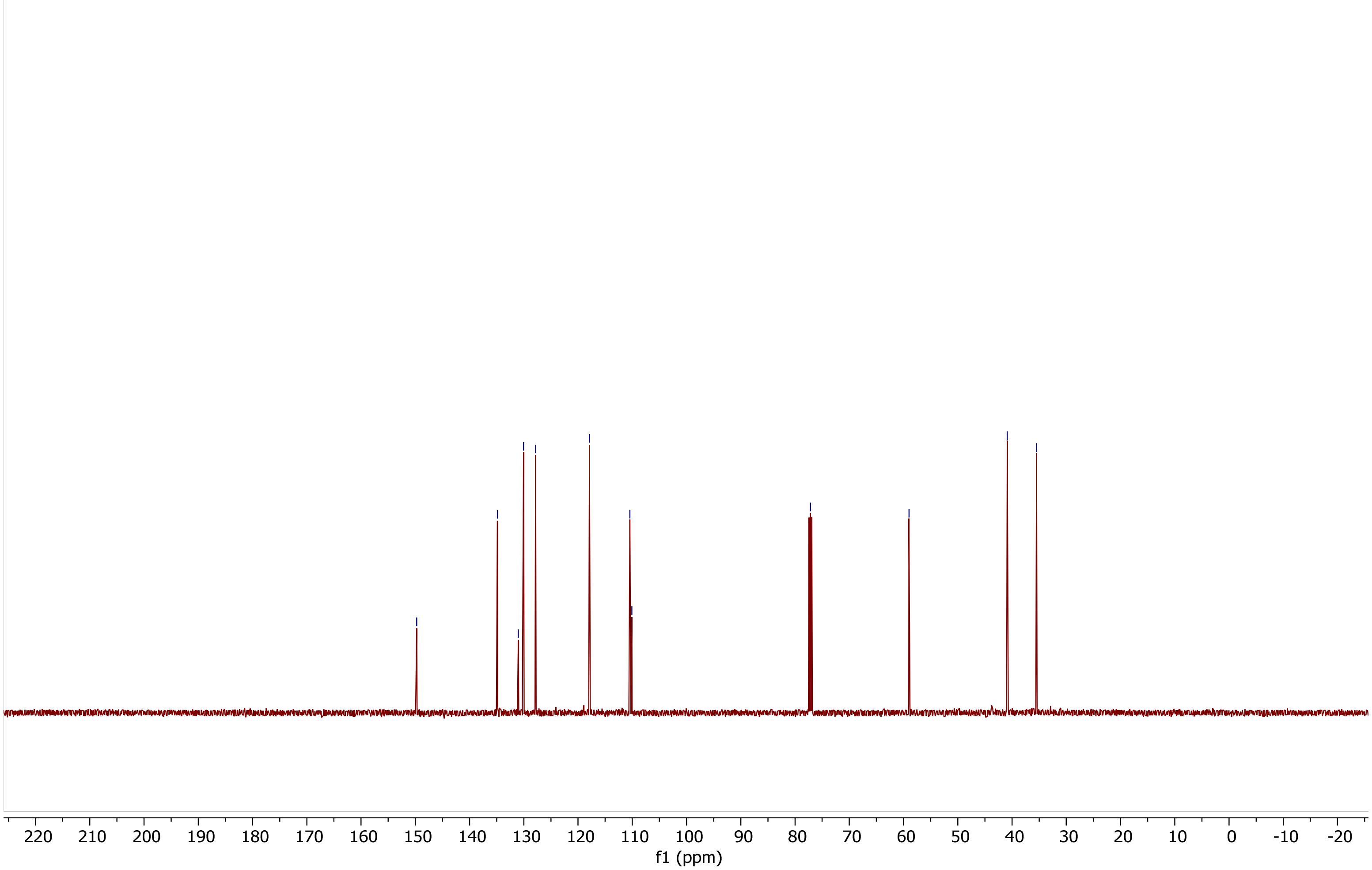

HSQC NMR (500 MHz, $CDCl_3$) 2-Allyl-5-bromoindoline (**S9**)

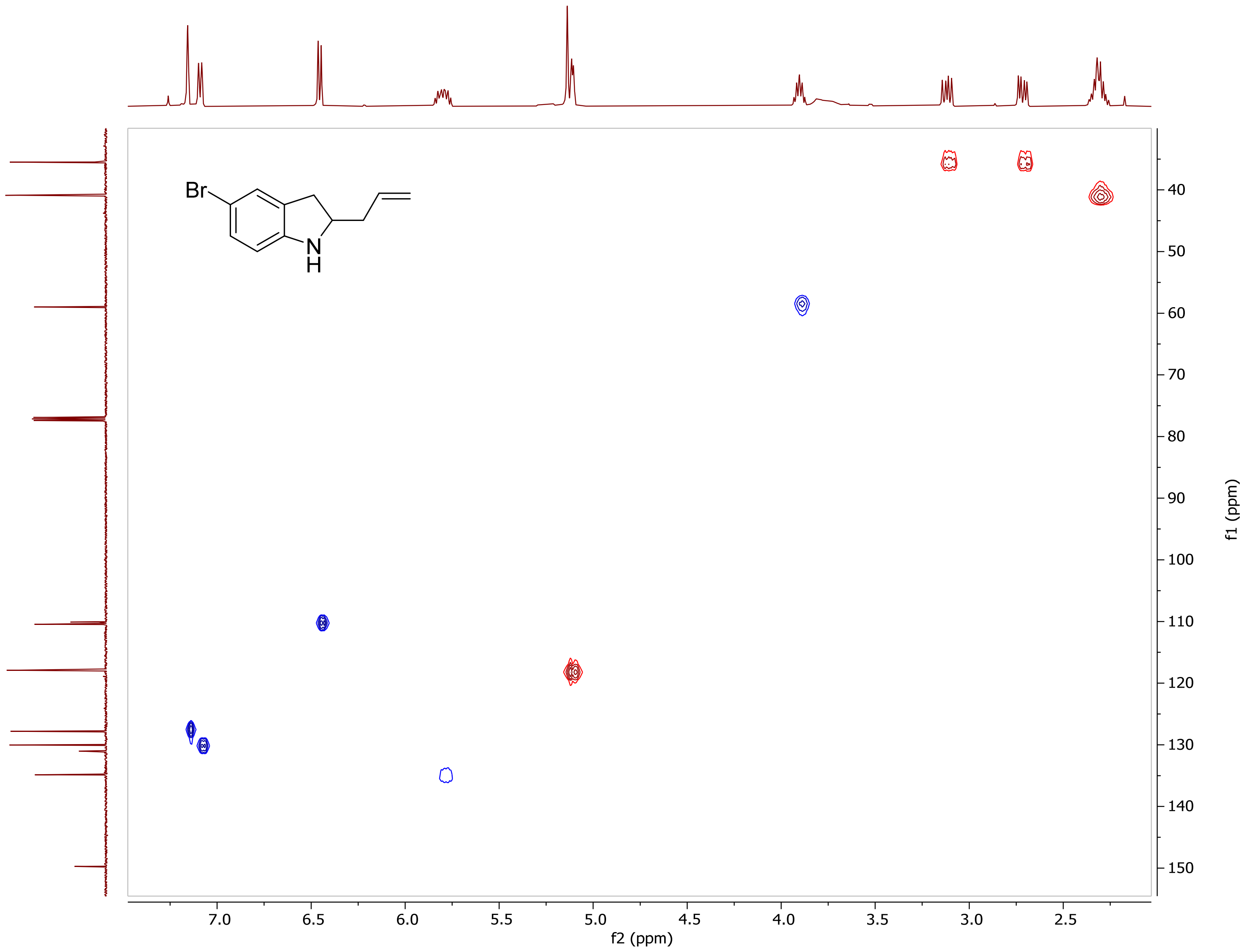

COSY NMR (500 MHz, $CDCl_3$) 2-Allyl-5-bromoindoline (**S9**)

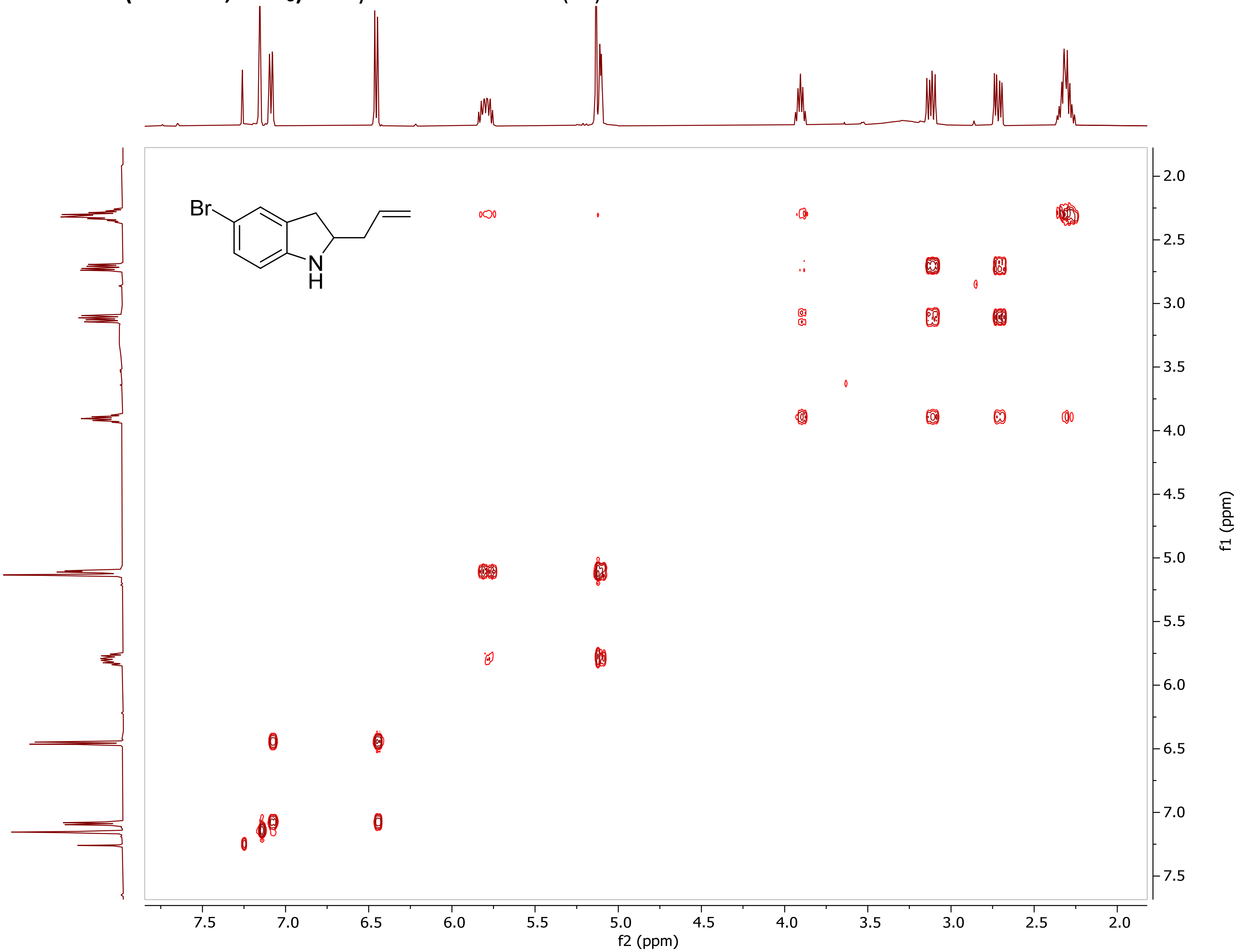

HMBC NMR (500 MHz, $CDCl_3$) 2-Allyl-5-bromoindoline (**S9**)

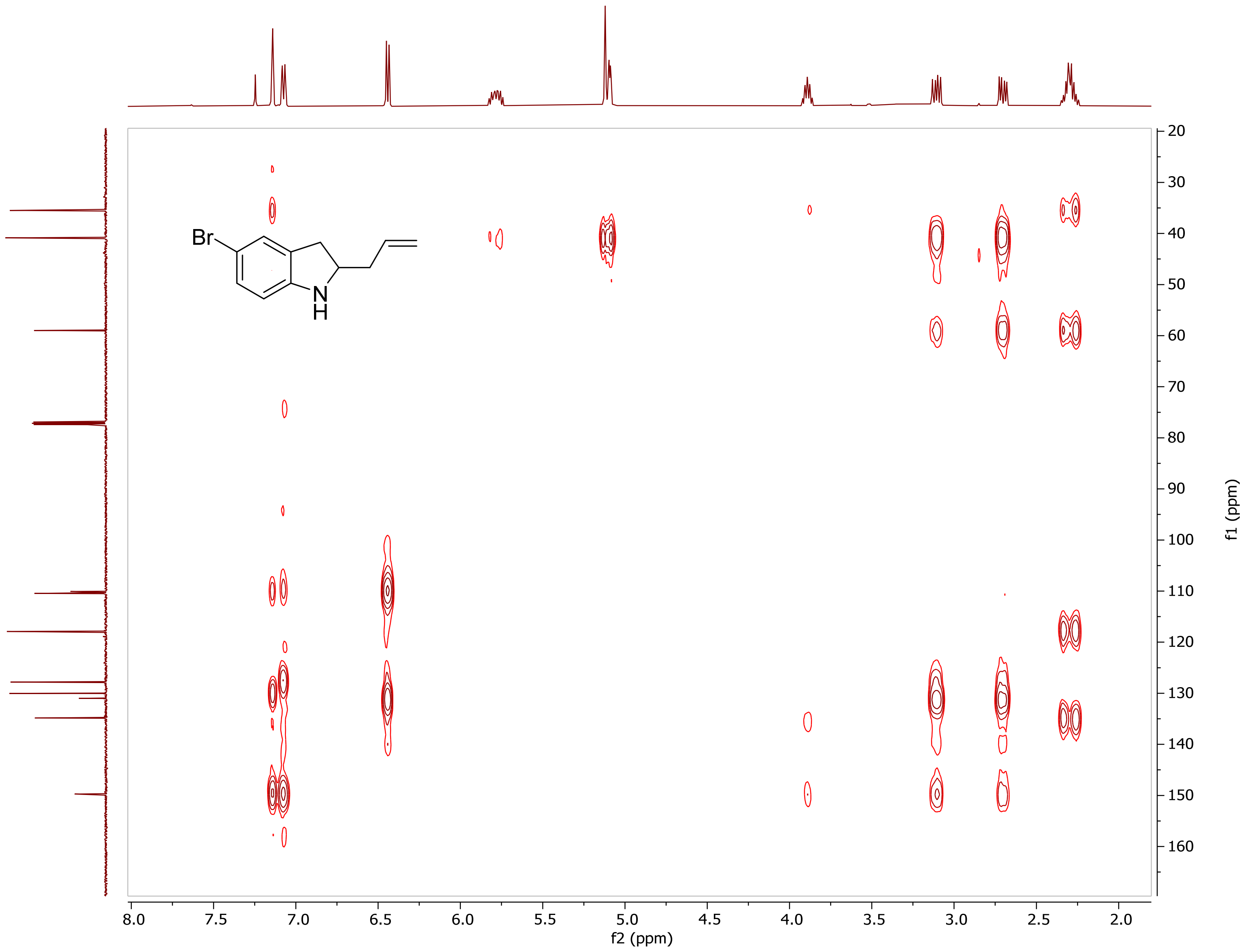

^{1}H NMR (500 MHz, CDCl$_3$) 2-Allyl-5-bromo-1-((2-nitro-4-(trifluoromethyl)phenyl)sulfonyl)indoline (**9a**)

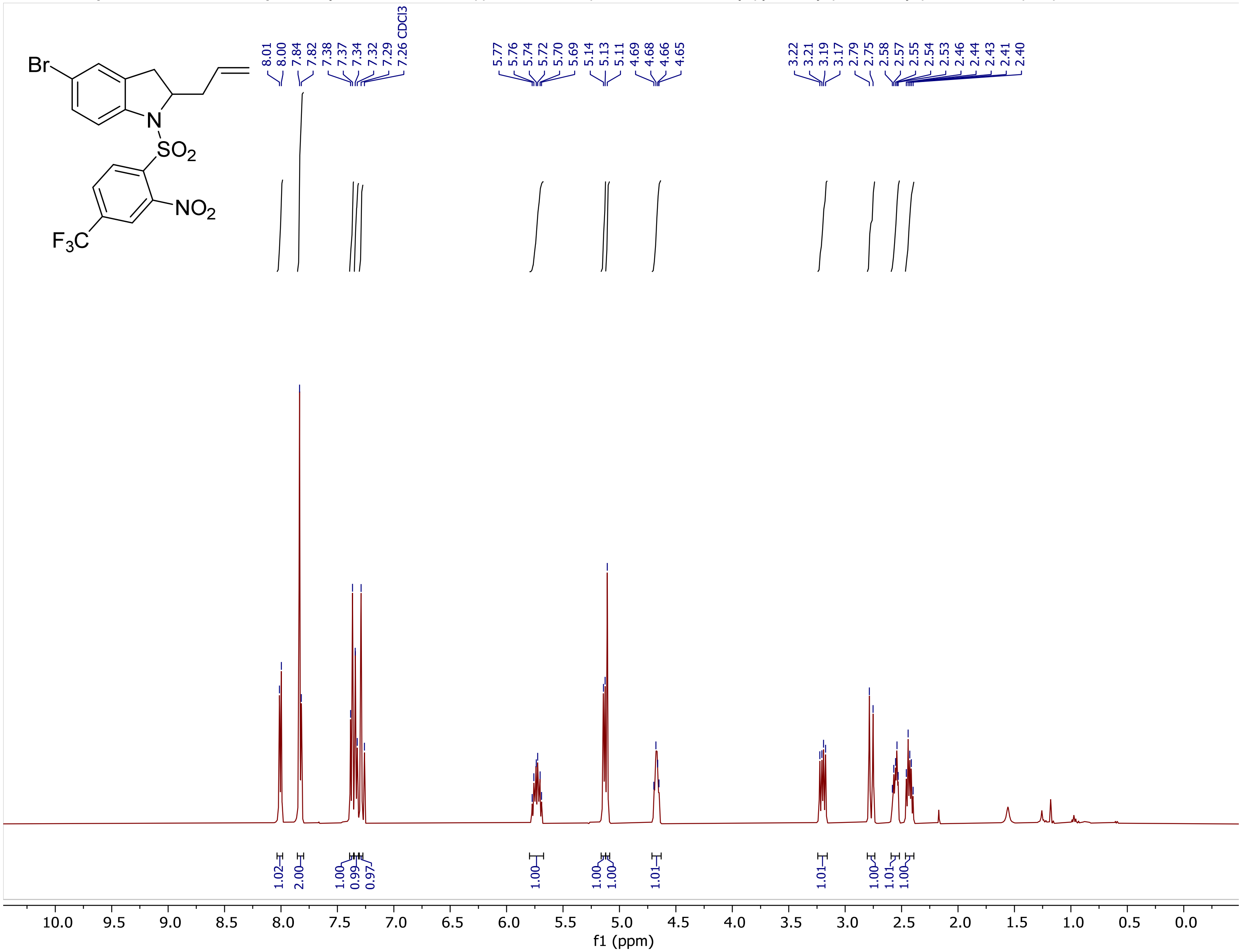

^{13}C {^{1}H, ^{19}F} NMR (126 MHz, $CDCl_3$) 2-Allyl-5-bromo-1-((2-nitro-4-(trifluoromethyl)phenyl)sulfonyl)indoline (**9a**)

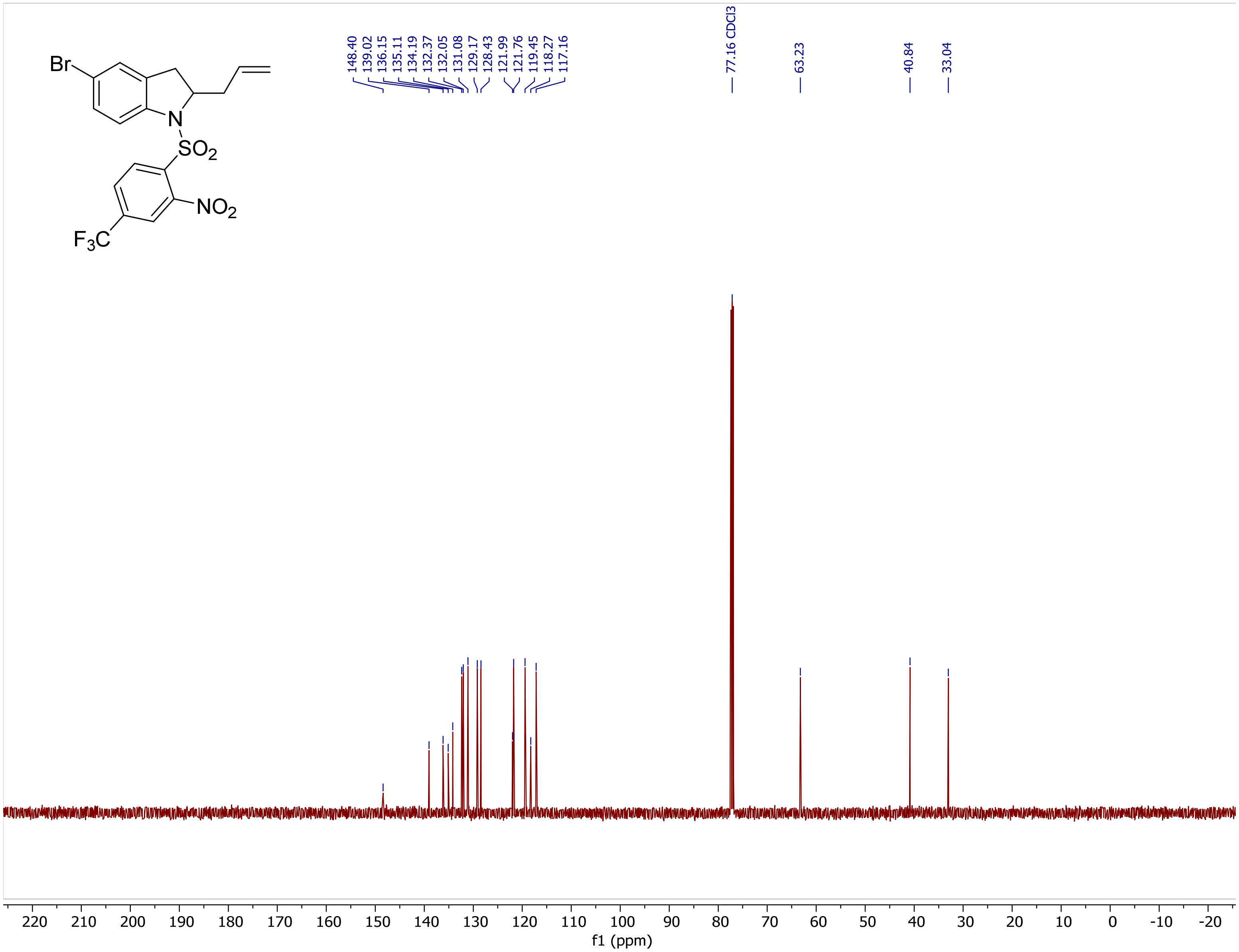

^{1}H NMR (500 MHz, CDCl$_3$) 5-bromo-1-((2-nitro-4-(trifluoromethyl)phenyl)sulfonyl)-2-(2(triethylsilyl)peroxy)propyl)indoline (**9b**)

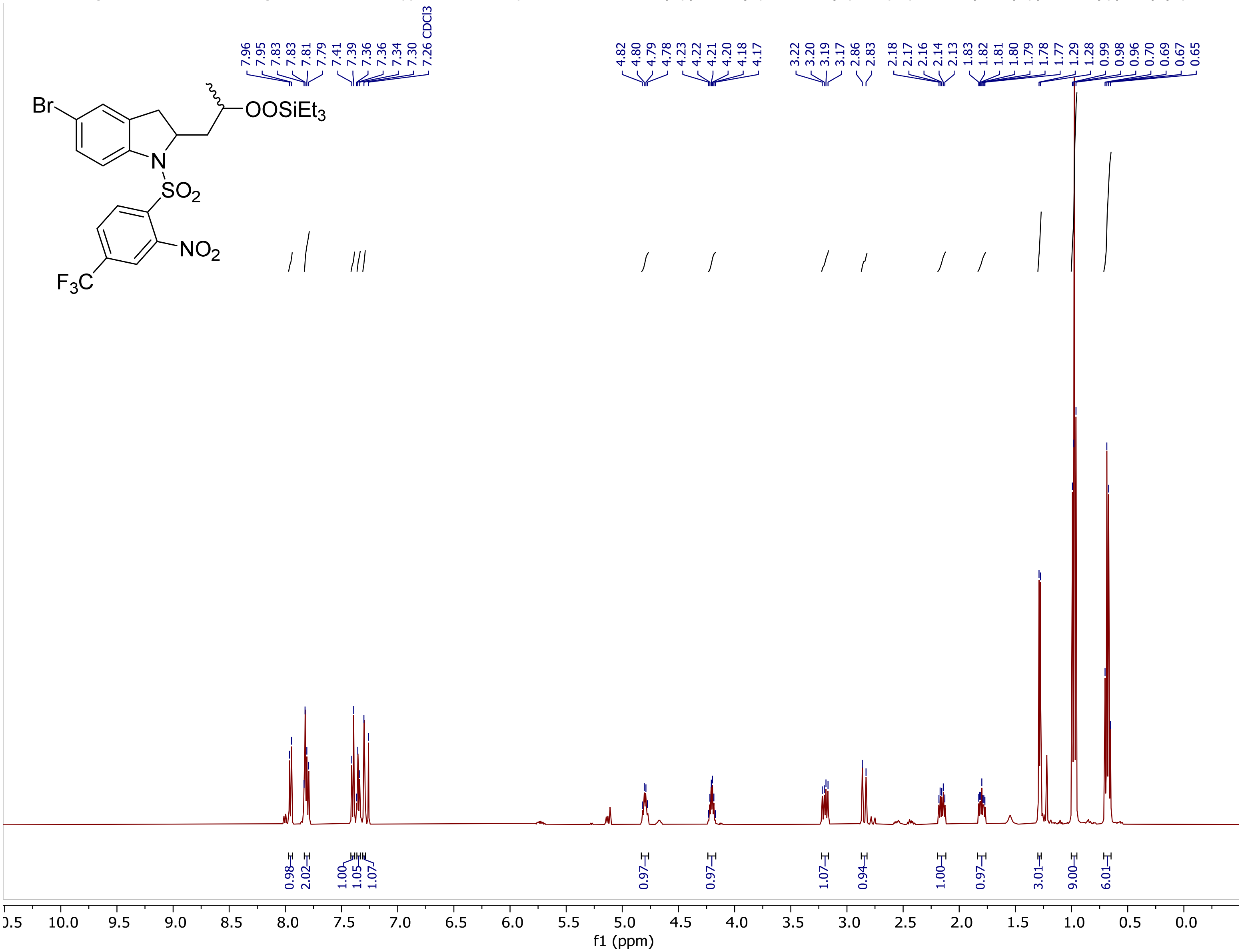

^{13}C {^{1}H, ^{19}F} NMR (126 MHz, $CDCl_3$) 5-bromo-1-((2-nitro-4-(trifluoromethyl)phenyl)sulfonyl)-2-(2(triethylsilyl)peroxy)propyl)indoline (**9b**)

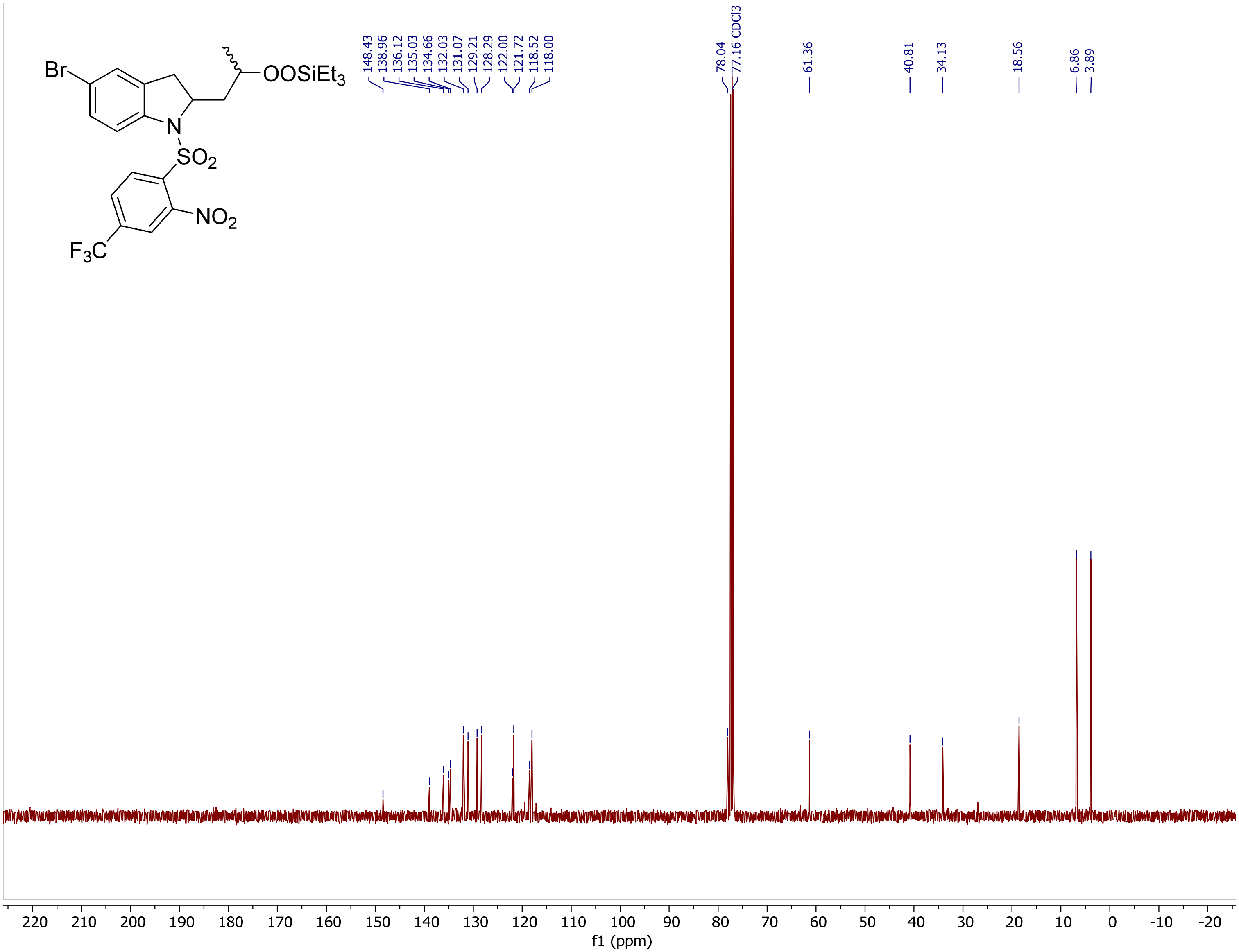

^{1}H NMR (500 MHz, C_6D_6) 5-bromo-2-(2-((tert-butyldiphenylsilyl)peroxy)propyl)-1-((2-nitro-4-(trifluoromethyl)phenyl)sulfonyl)indoline (**70** and **71**)

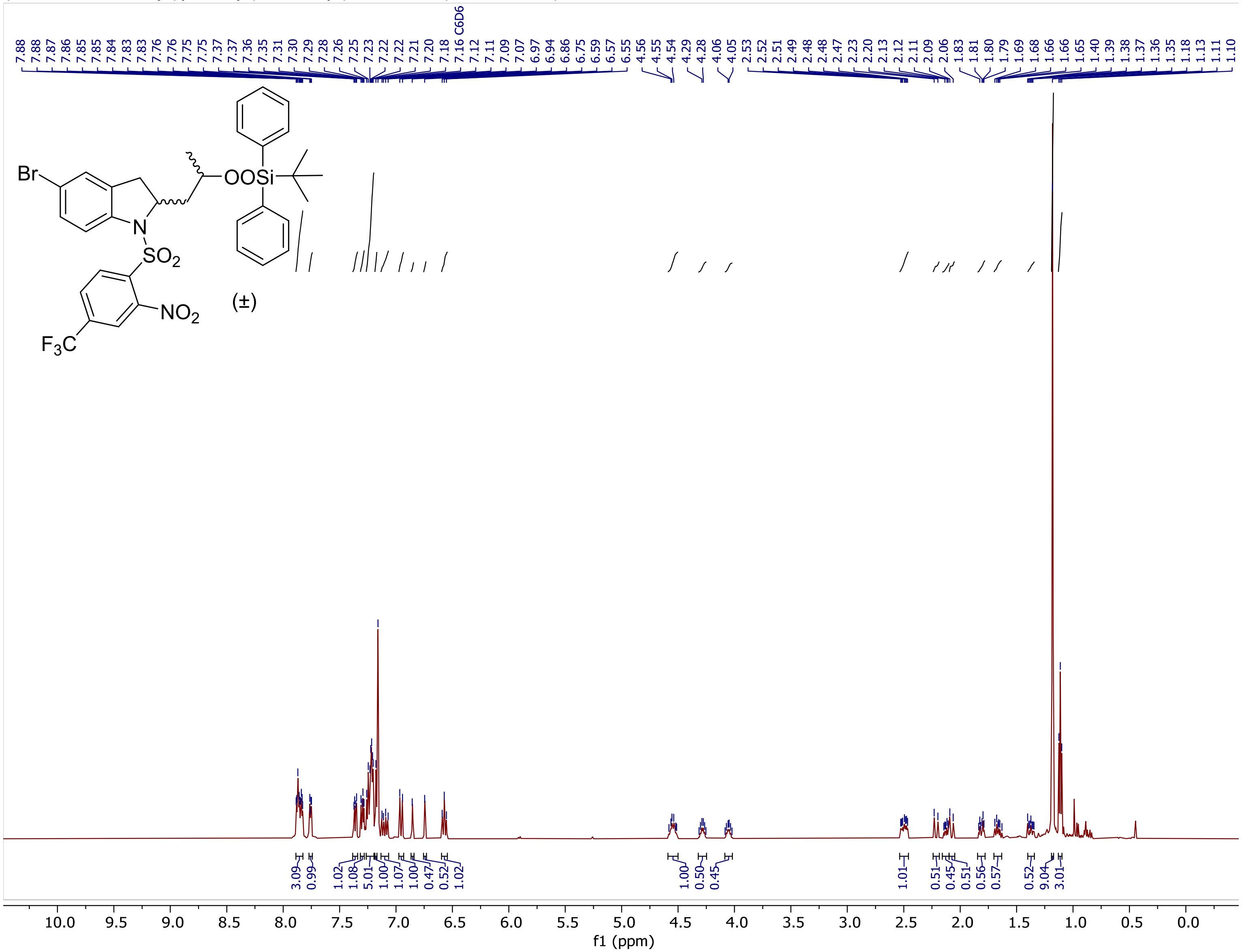

$^{13}C\ \{^1H,\ ^{19}F\}$ NMR (126 MHz, C_6D_6) 5-bromo-2-(2-((tert-butyldiphenylsilyl)peroxy)propyl)-1-((2-nitro-4-(trifluoromethyl)phenyl)sulfonyl)indoline (**70** and **71**)

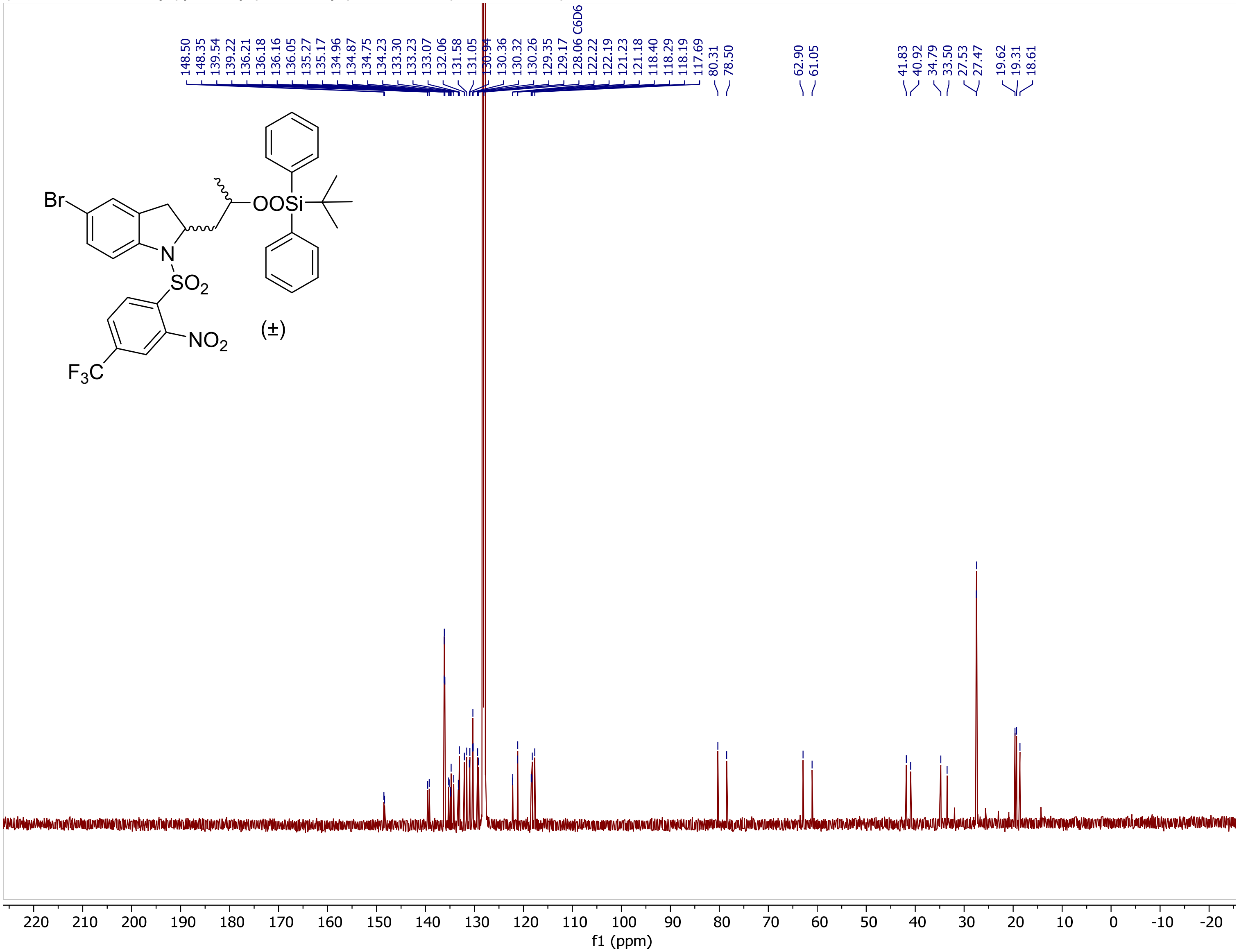

HSQC NMR (500 MHz, C_6D_6) 5-bromo-2-(2-((tert-butyldiphenylsilyl)peroxy)propyl)-1-((2-nitro-4-(trifluoromethyl)phenyl)sulfonyl)indoline (**70** / **71**)

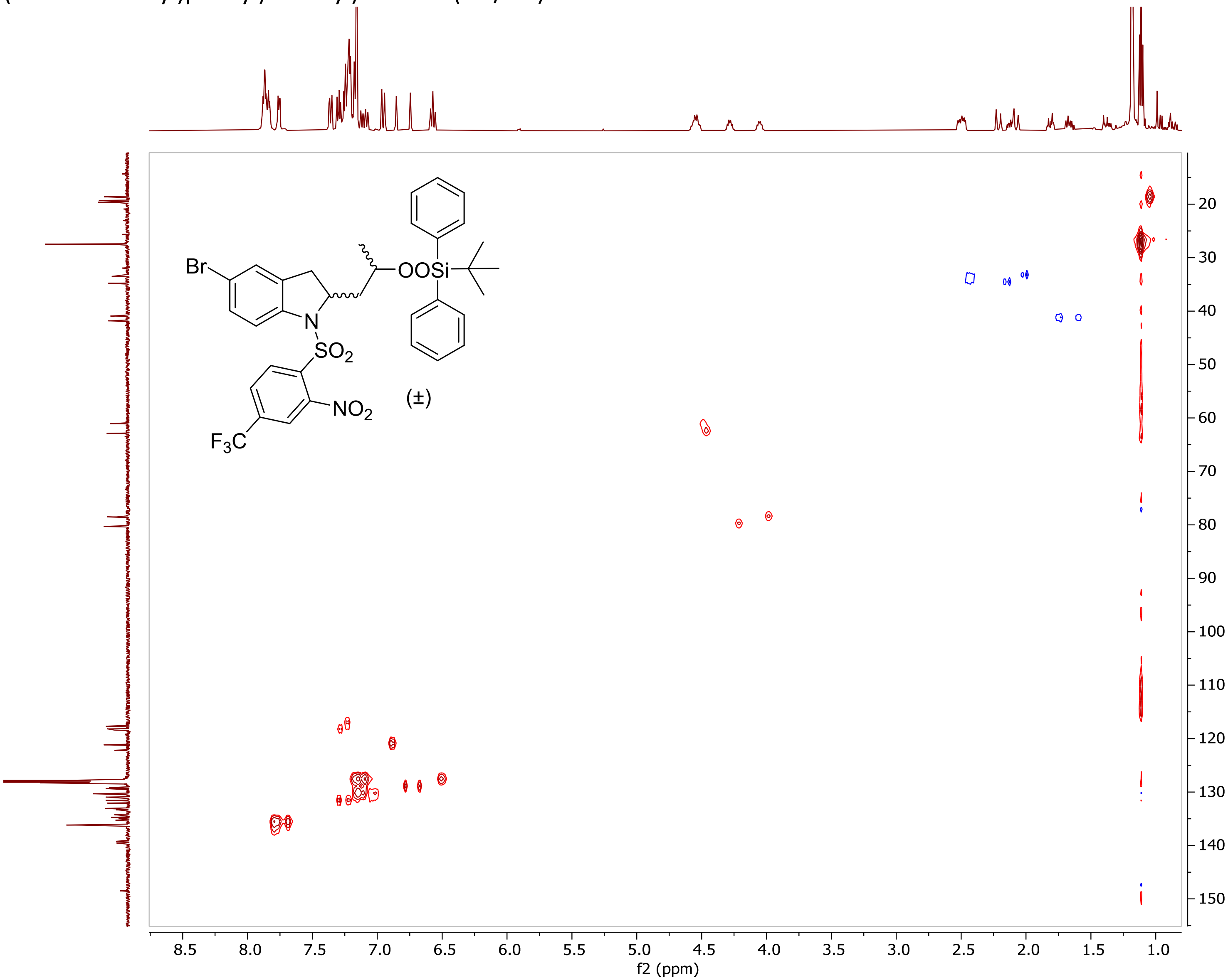

^{1}H NMR (500 MHz, C_6D_6) 2-Methyl-2,3,3a,4-tetrahydroisoxazolo[2,3-*a*]indole (**72**)

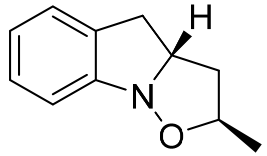

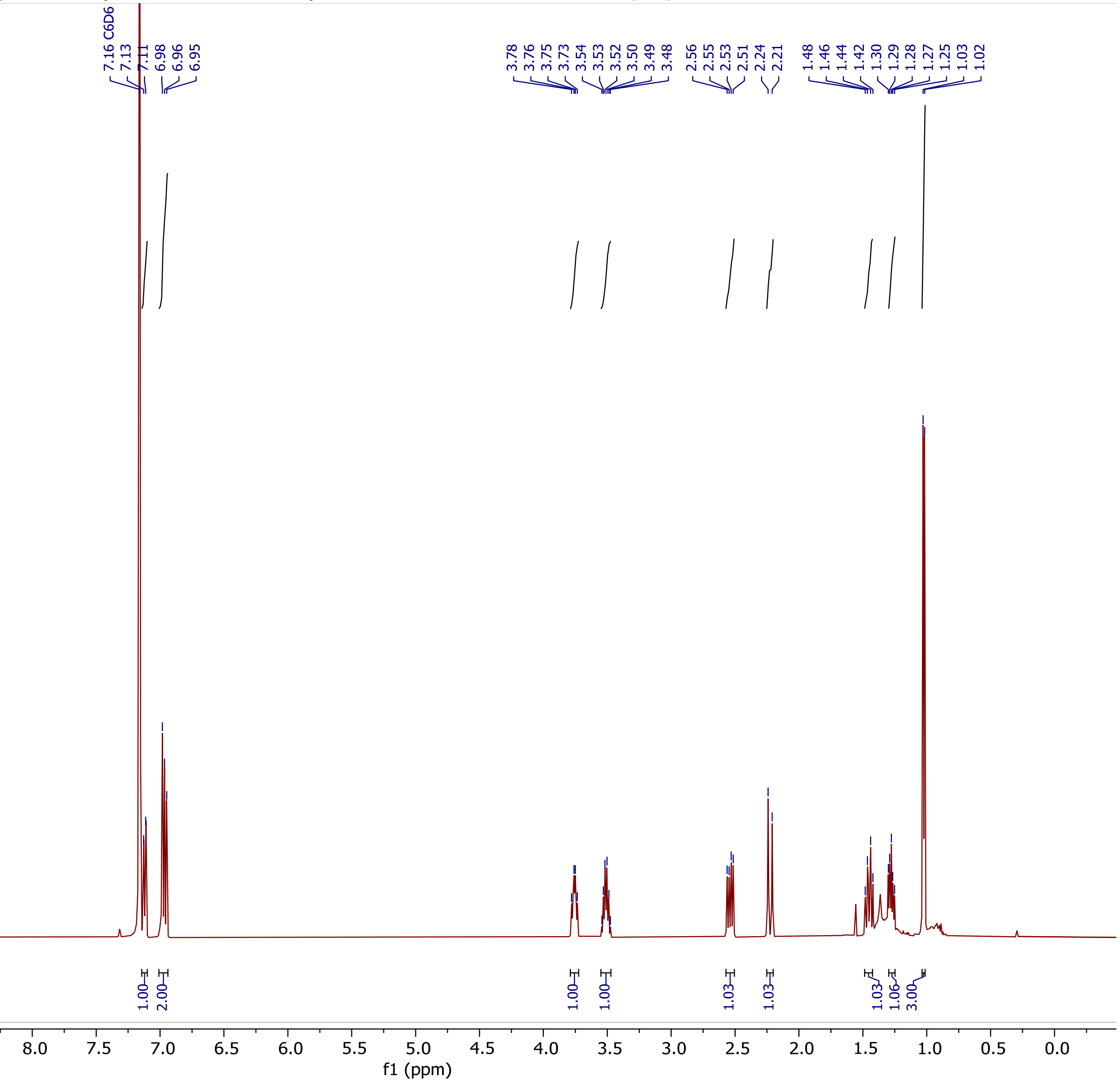

$^{13}C\{^1H\}$ NMR (126 MHz, C_6D_6) 2-Methyl-2,3,3a,4-tetrahydroisoxazolo[2,3-*a*]indole (**72**)

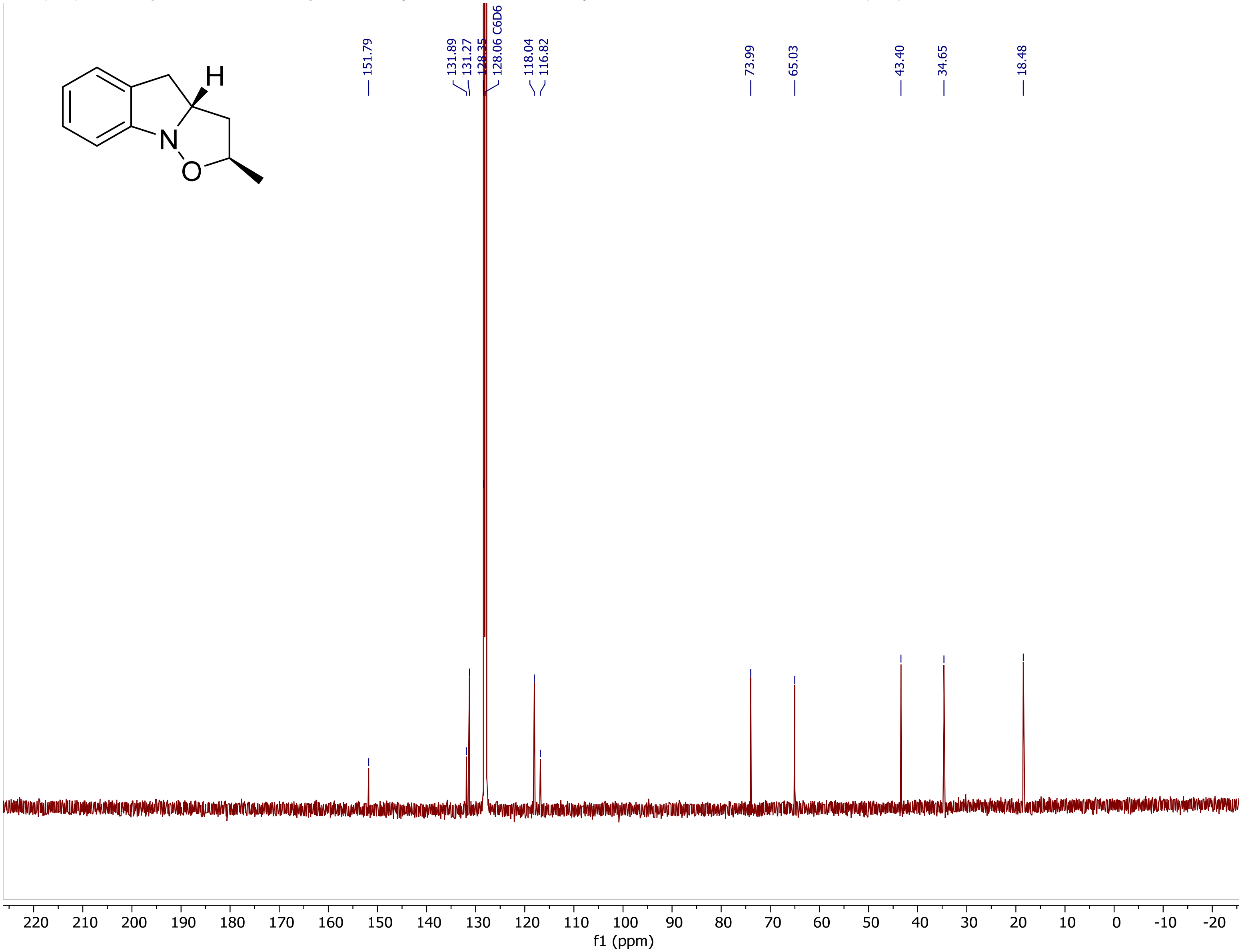

HSQC NMR (500 MHz, C_6D_6) 2-Methyl-2,3,3a,4-tetrahydroisoxazolo[2,3-*a*]indole (**72**)

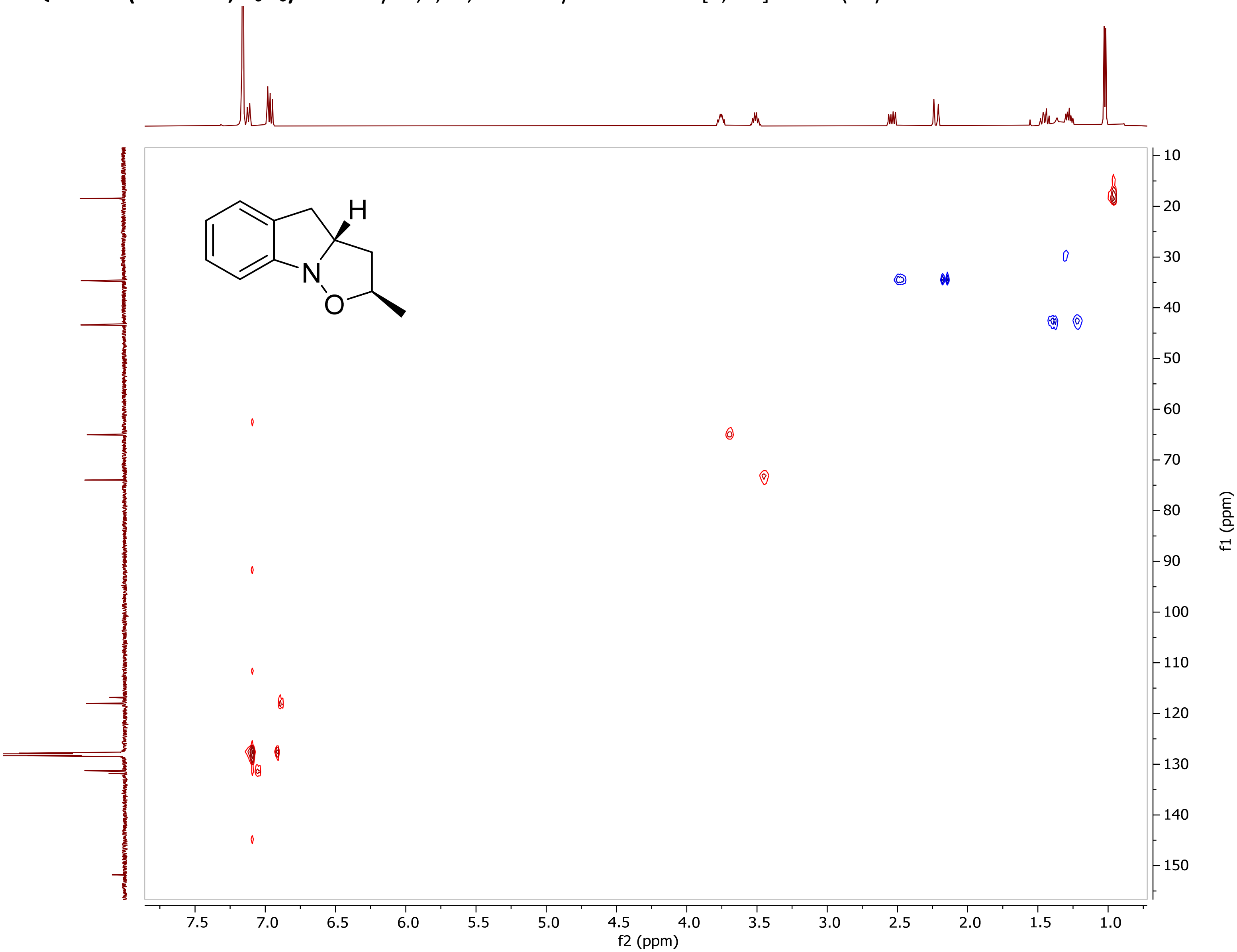

1D NOESY NMR (500 MHz, C_6D_6) 2-Methyl-2,3,3a,4-tetrahydroisoxazolo[2,3-*a*]indole (**72**)

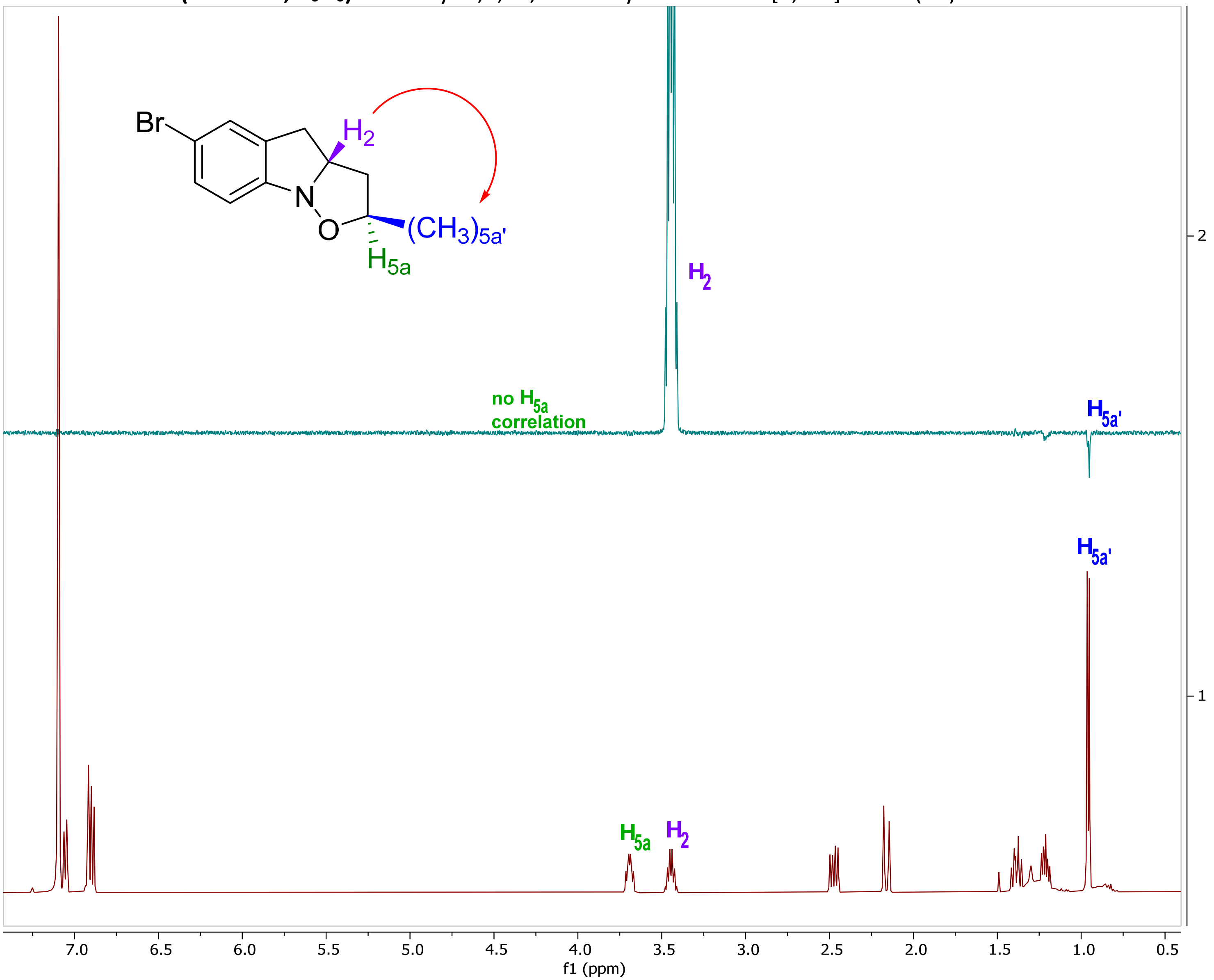

1D NOESY NMR (500 MHz, C_6D_6) 2-Methyl-2,3,3a,4-tetrahydroisoxazolo[2,3-*a*]indole (**72**)

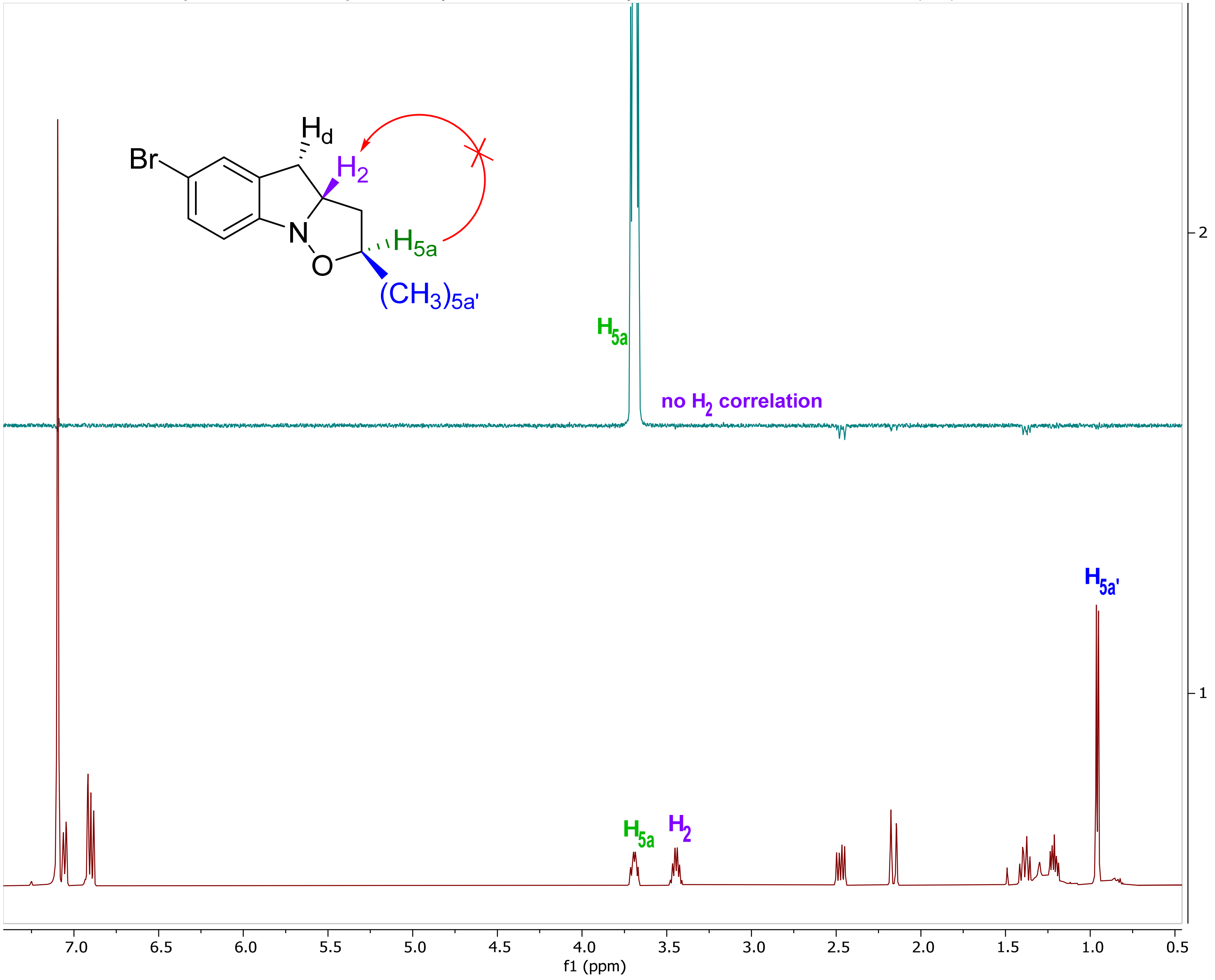

^{1}H NMR (500 MHz, C_6D_6) 2-Methyl-2,3,3a,4-tetrahydroisoxazolo[2,3-*a*]indole (**73**)

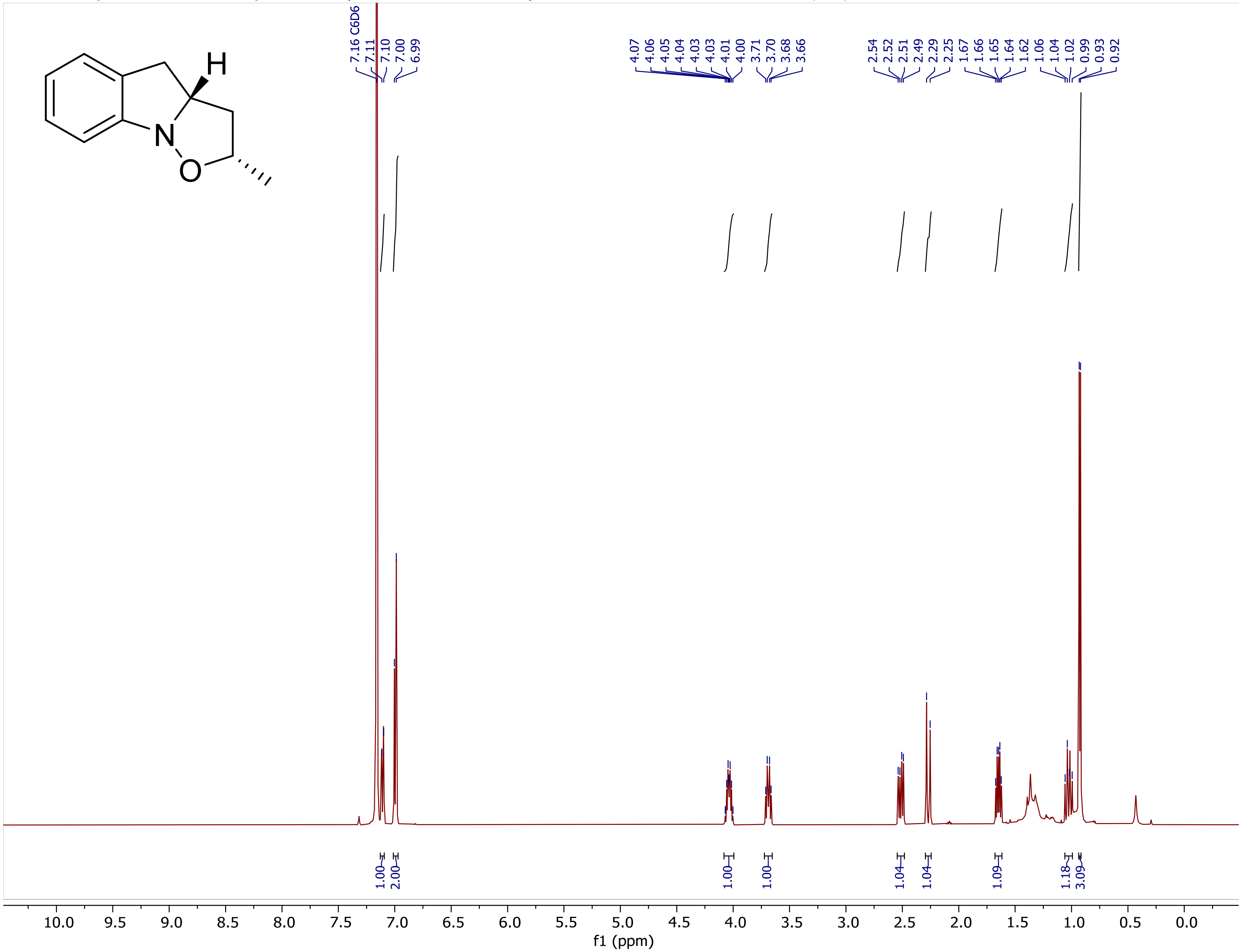

^{13}C {^{1}H} NMR (126 MHz, C_6D_6) 2-Methyl-2,3,3a,4-tetrahydroisoxazolo[2,3-*a*]indole (**73**)

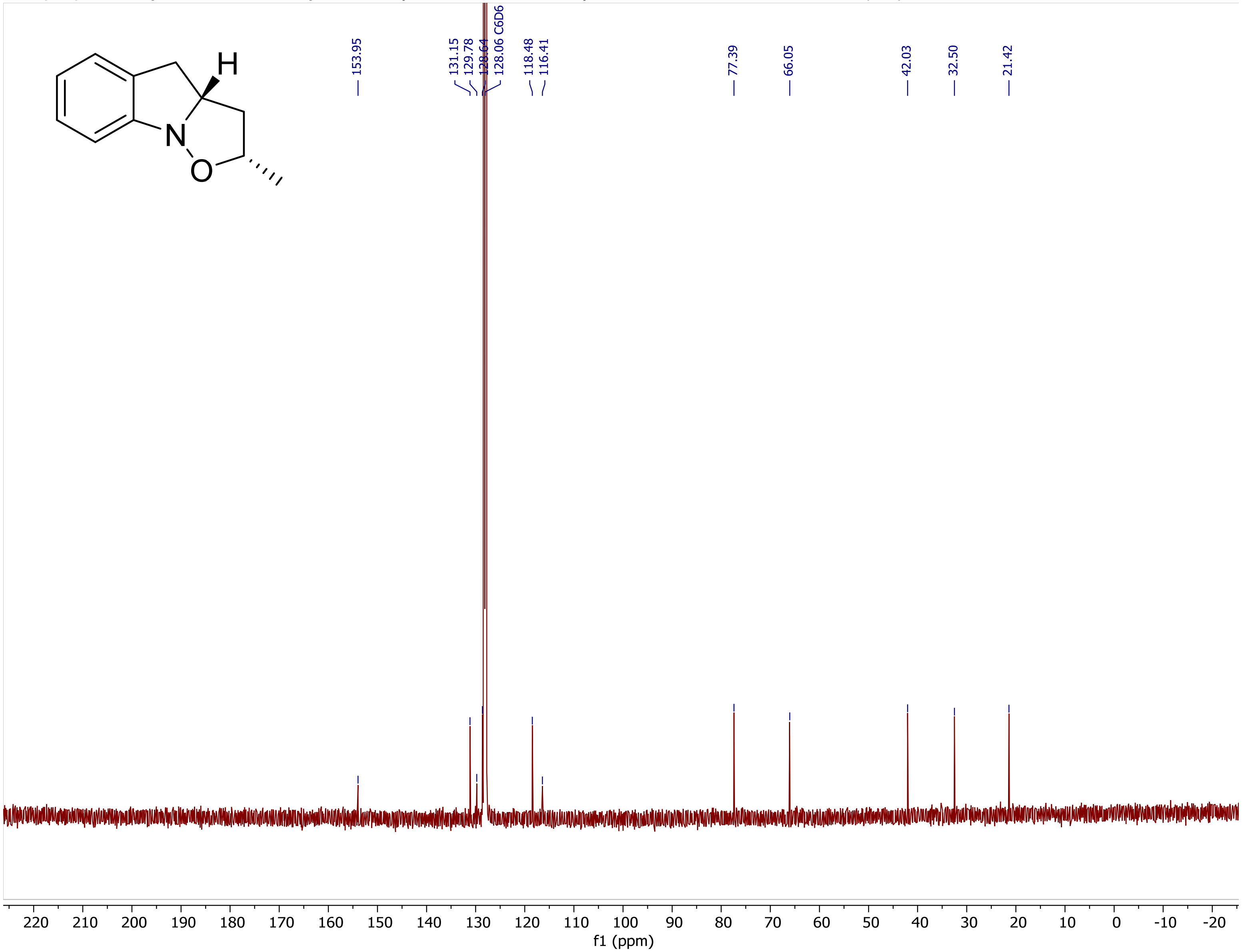

HSQC NMR (500 MHz, C_6D_6) 2-Methyl-2,3,3a,4-tetrahydroisoxazolo[2,3-*a*]indole (**73**)

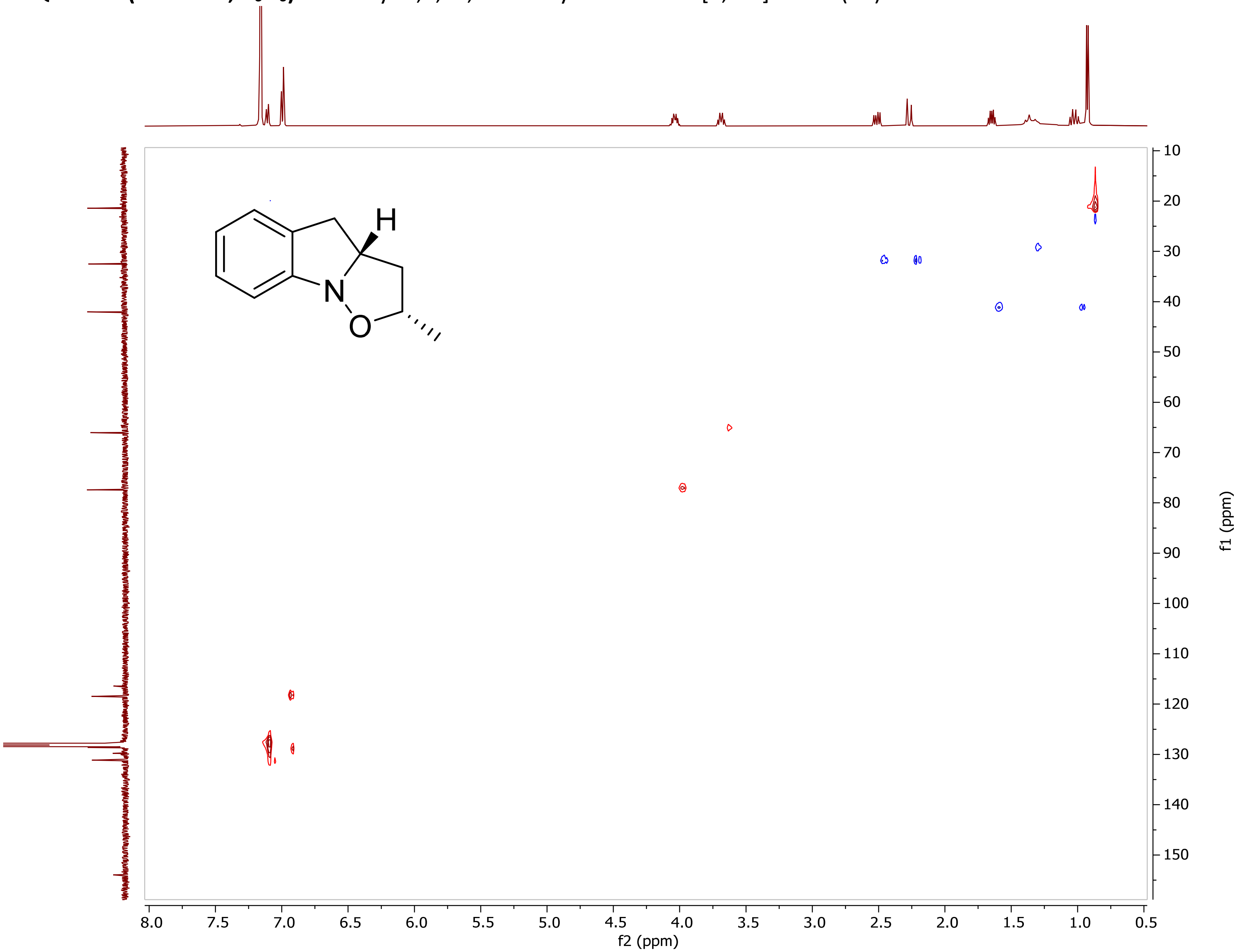

COSY NMR (500 MHz, C_6D_6) 2-Methyl-2,3,3a,4-tetrahydroisoxazolo[2,3-*a*]indole (**73**)

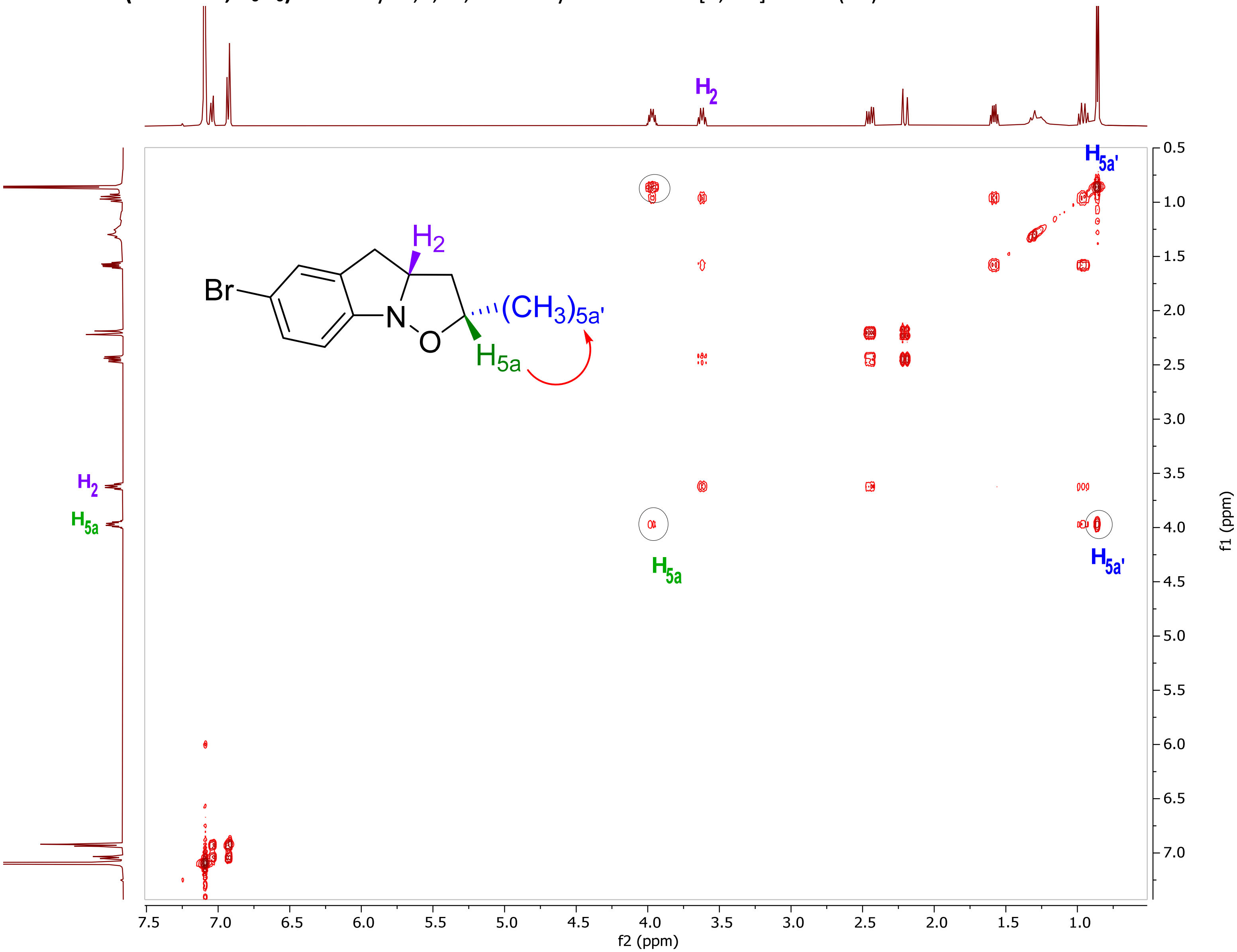

1D NOSEY NMR (500 MHz, C_6D_6) 2-Methyl-2,3,3a,4-tetrahydroisoxazolo[2,3-*a*]indole (**73**)

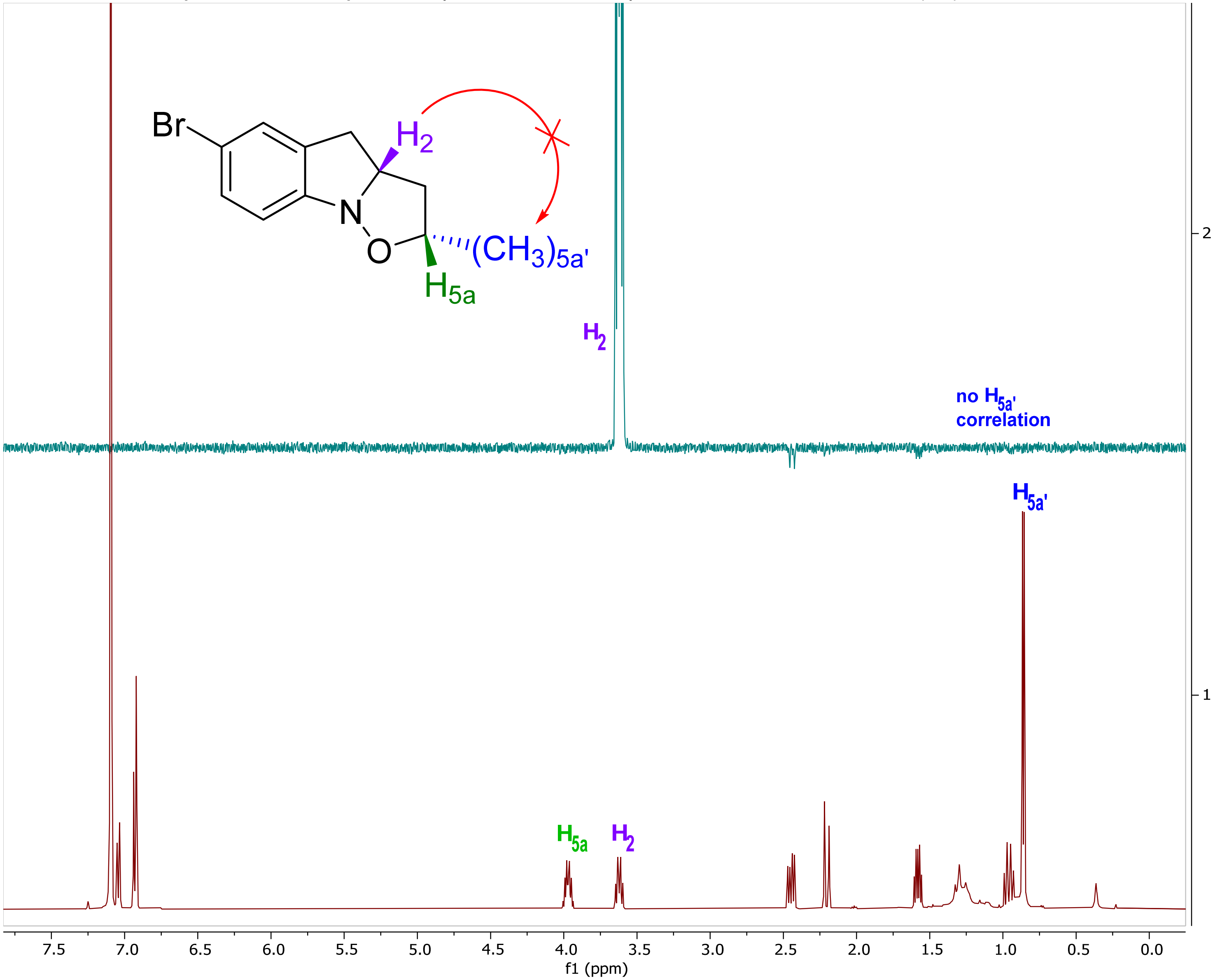

^{1}H NMR (500 MHz, $CDCl_3$) spectrum of *N*-(4-Hydroxybutyl)-*N*-(naphthalen-1-ylmethyl)-2-nitro-4-(trifluoromethyl)benzenesulfonamide (**3l**)

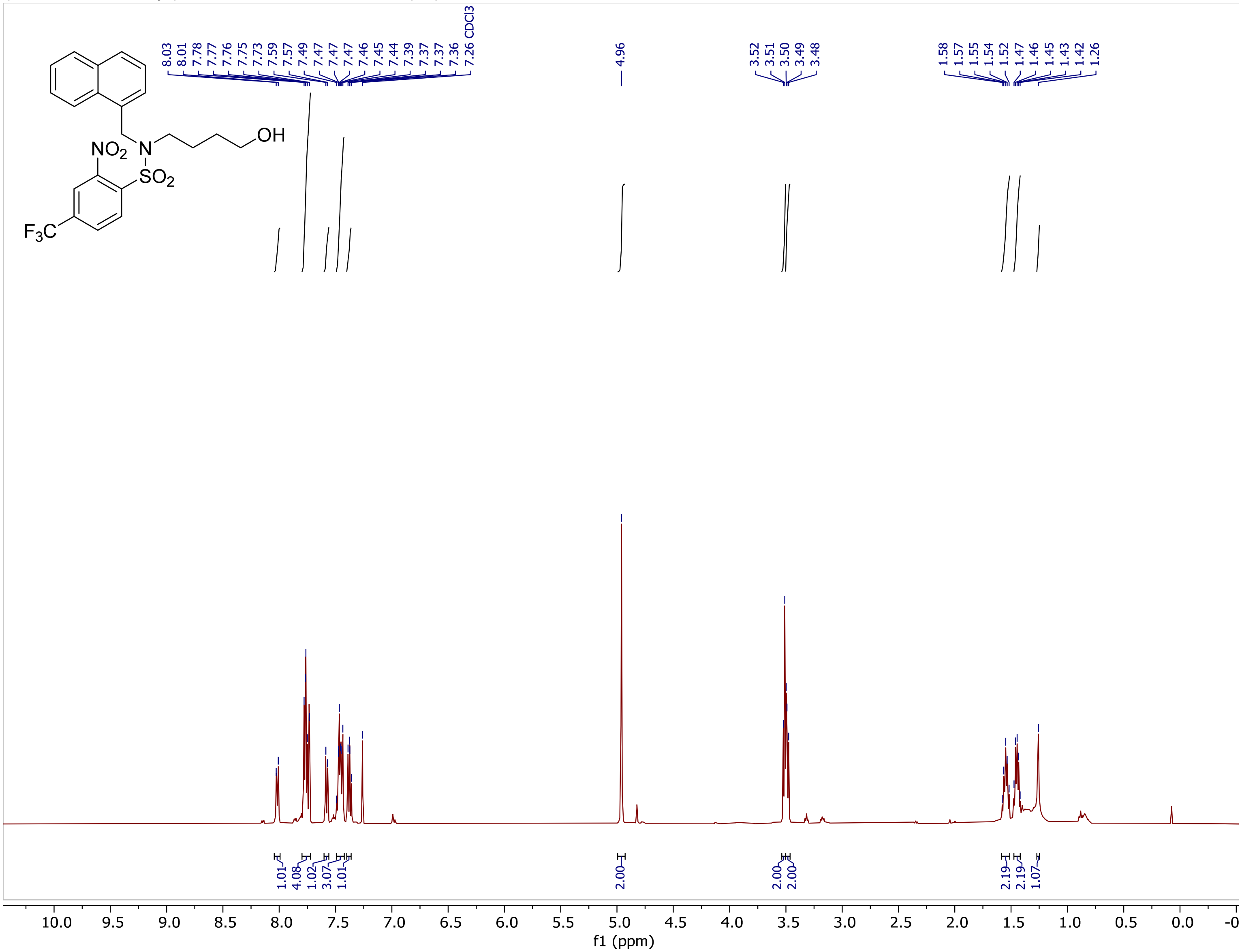

^{13}C {^{1}H, ^{19}F} NMR (126 MHz, $CDCl_3$) spectrum of *N*-(4-Hydroxybutyl)-*N*-(naphthalen-1-ylmethyl)-2-nitro-4-(trifluoromethyl)benzenesulfonamide (**3l**)

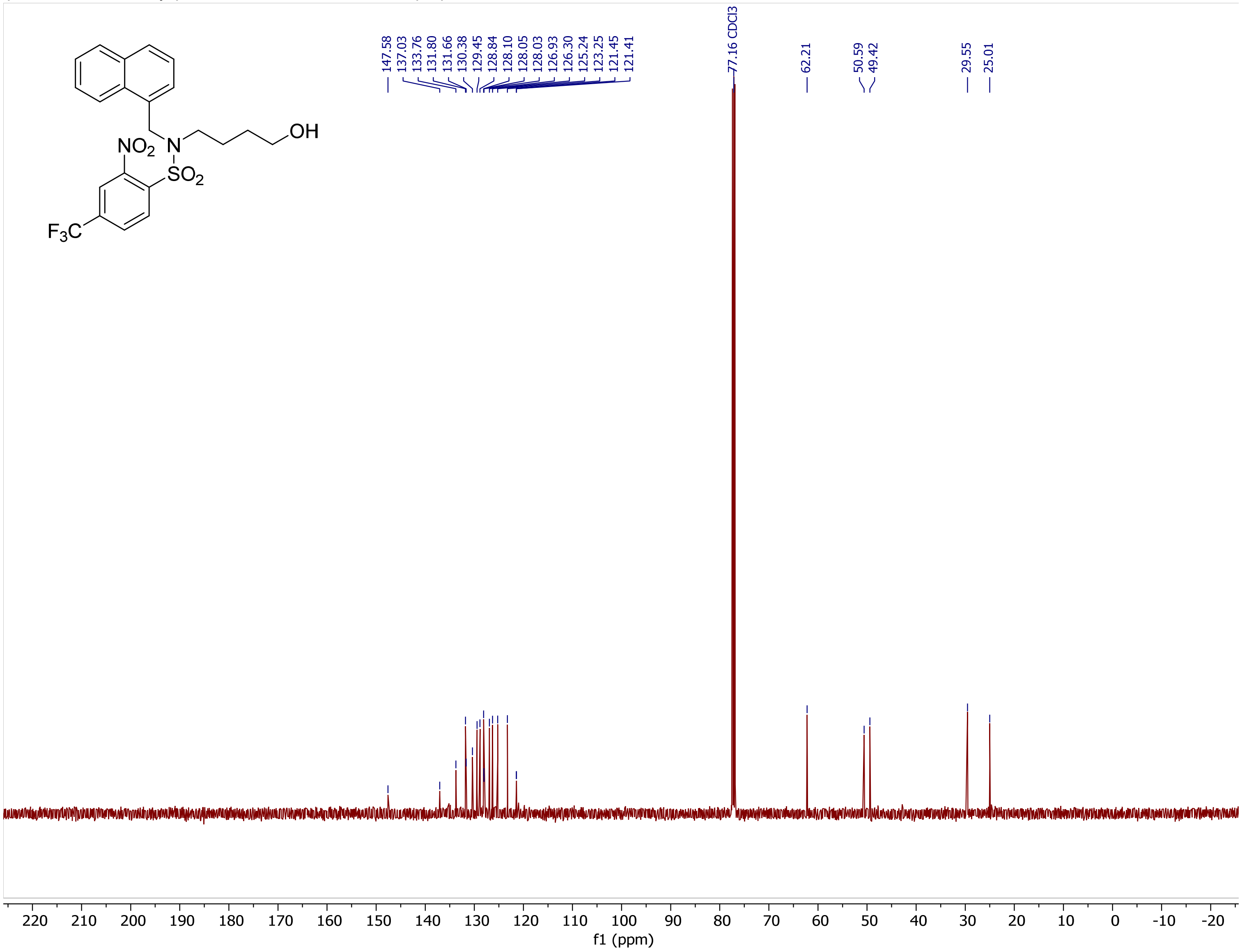

^{1}H NMR (500 MHz, $CDCl_3$) spectrum of *N*-(4-Iodobutyl)-*N*-(naphthalen-1-ylmethyl)-2-nitro-4-(trifluoromethyl)benzenesulfonamide (**3m**)

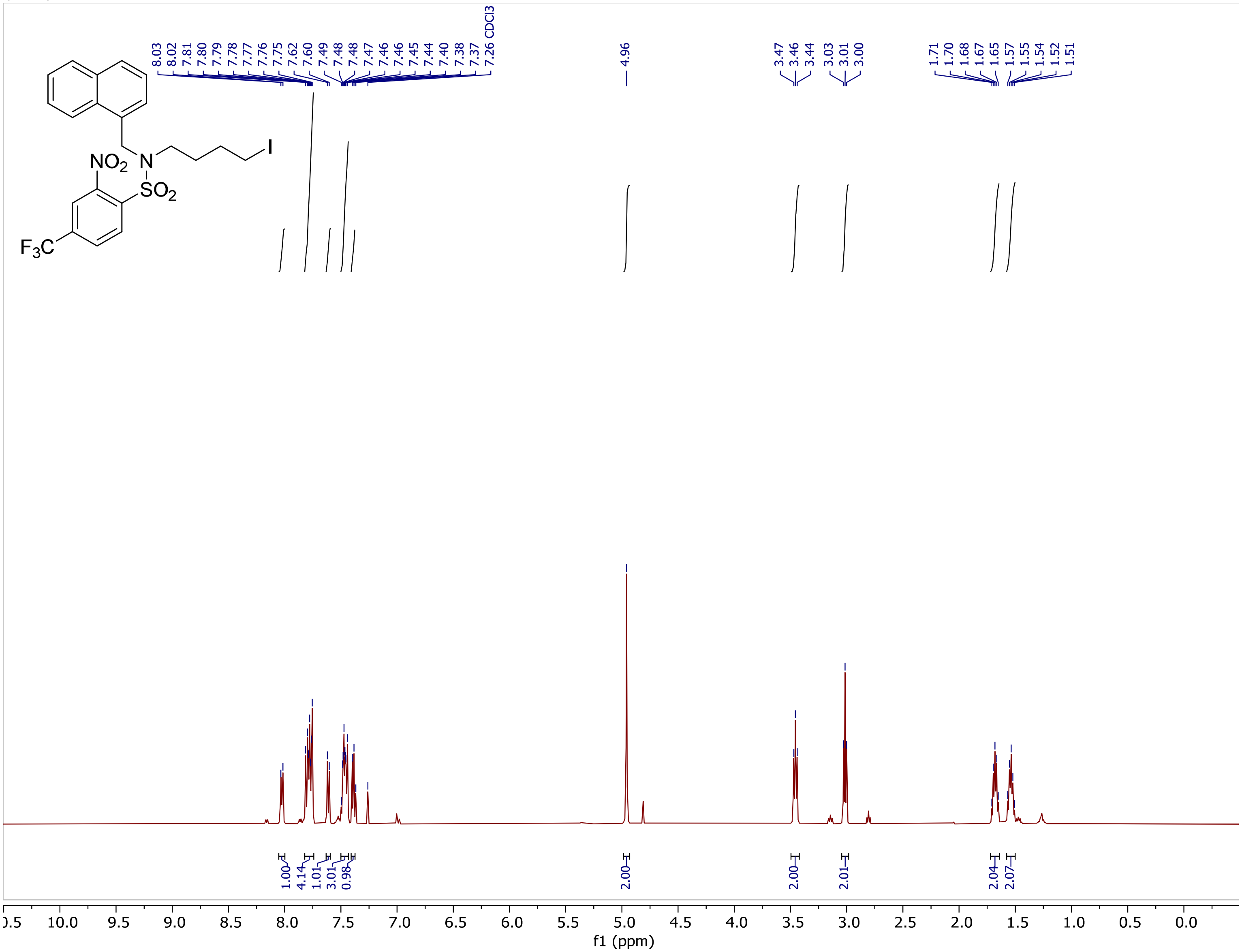

^{13}C {^{1}H} NMR (126 MHz, $CDCl_3$) spectrum of *N*-(4-Iodobutyl)-*N*-(naphthalen-1-ylmethyl)-2-nitro-4-(trifluoromethyl)benzenesulfonamide (**3m**)

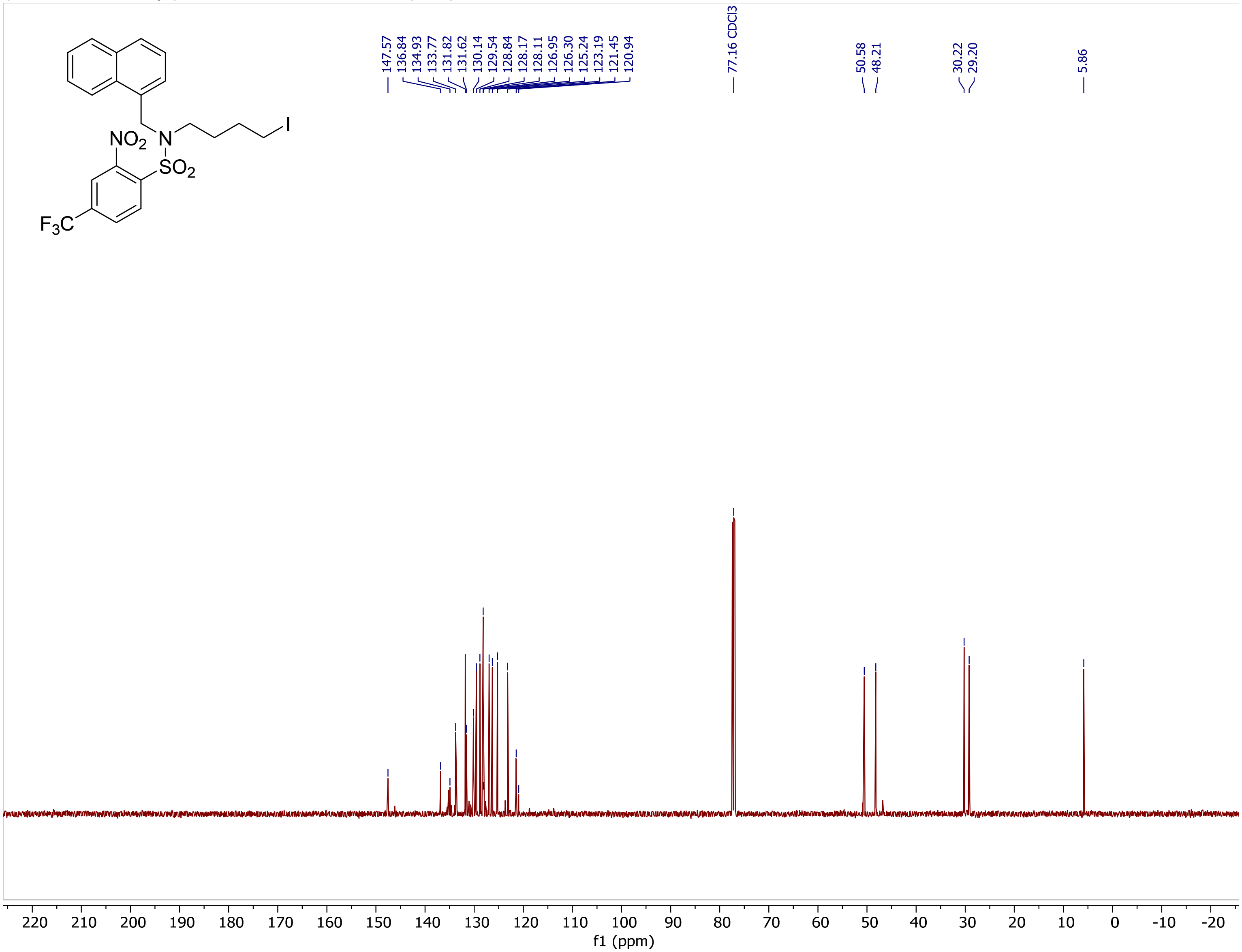

^{1}H NMR (500 MHz, $CDCl_3$) spectrum of *N*-(4-Hydroperoxybutyl)-*N*-(naphthalen-1-ylmethyl)-2-nitro-4-(trifluoromethyl)benzenesulfonamide (**3n**)

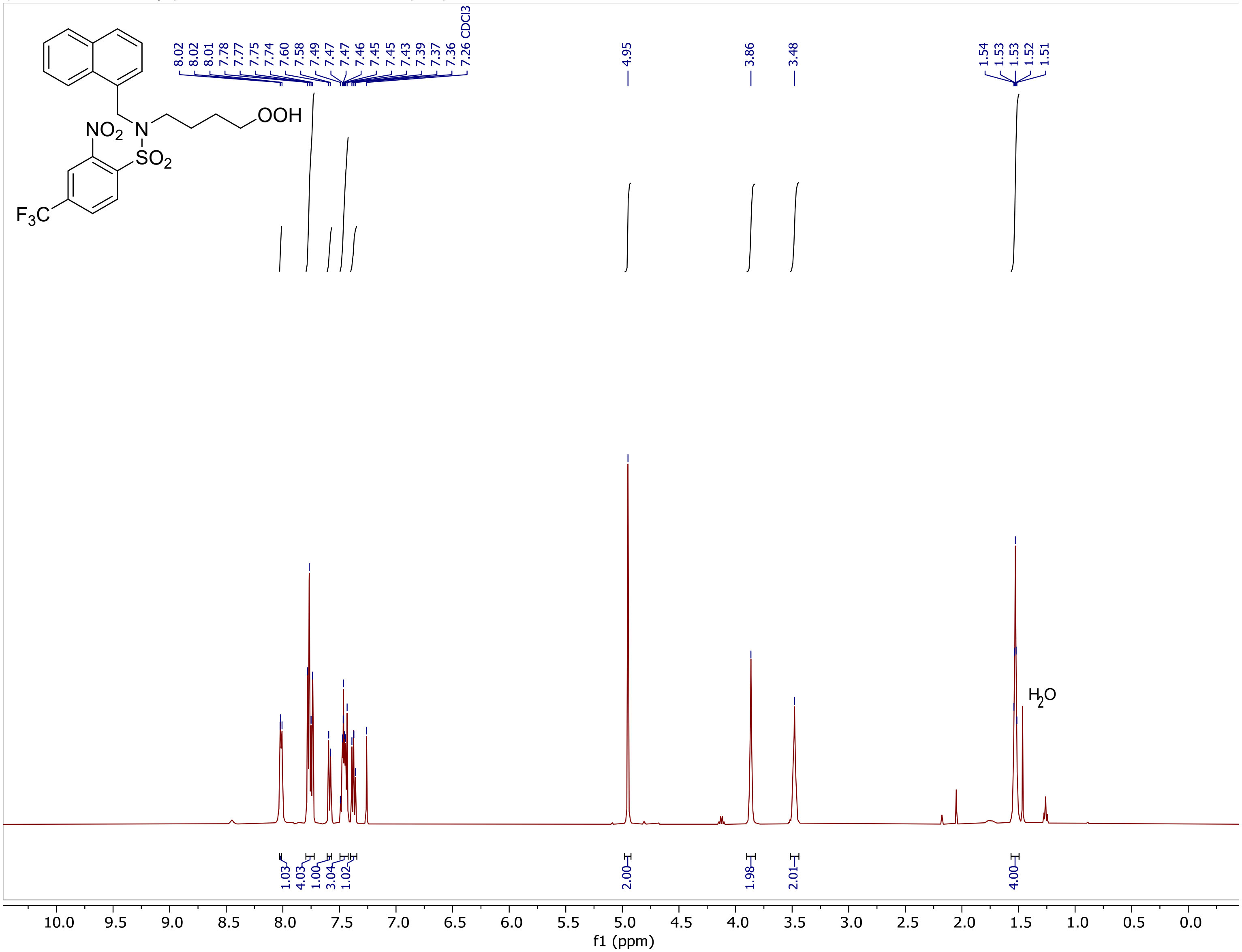

^{13}C {^{1}H} NMR (126 MHz, $CDCl_3$) spectrum of *N*-(4-Hydroperoxybutyl)-*N*-(naphthalen-1-ylmethyl)-2-nitro-4-(trifluoromethyl)benzenesulfonamide (**3n**)

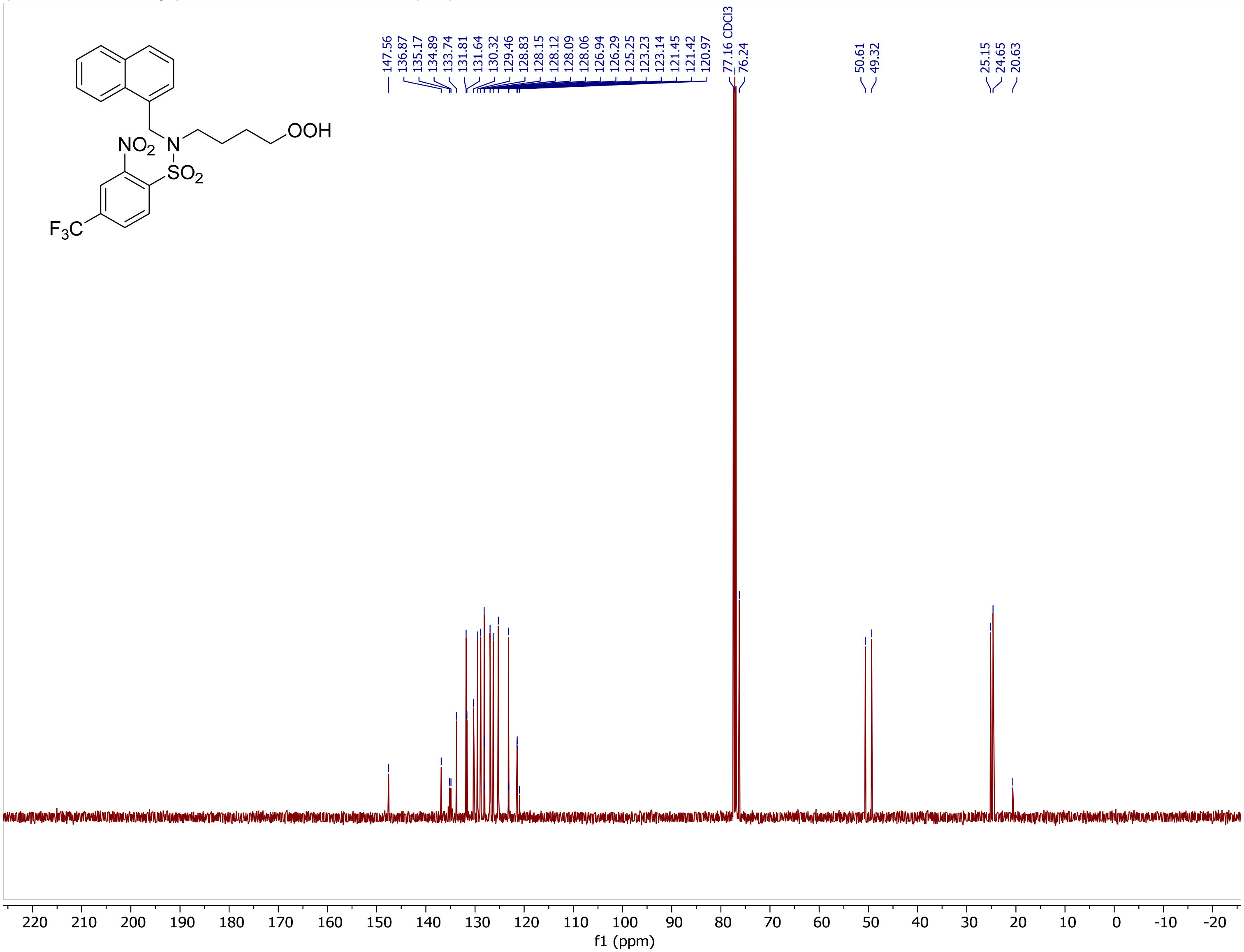

^{1}**H NMR (500 MHz, C_6D_6)** spectrum of *N*-(4-((*tert*-Butyldiphenylsilyl)peroxy)butyl)-*N*-(naphthalen-1-ylmethyl)-2-nitro-4-(trifluoromethyl)benzenesulfonamide (**74**)

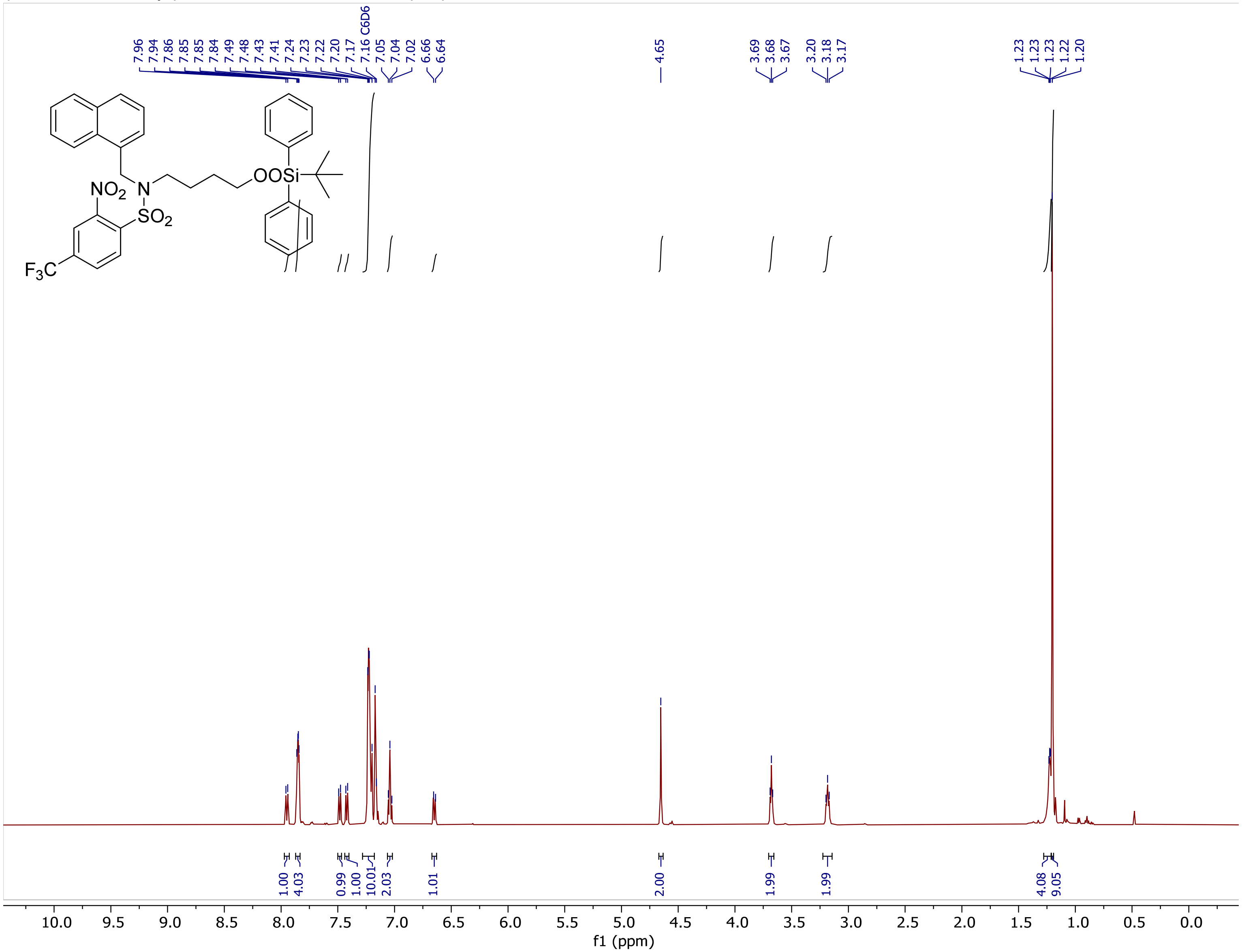

^{13}C {^{1}H} NMR (126 MHz, C_6D_6) spectrum of *N*-(4-((*tert*-Butyldiphenylsilyl)peroxy)butyl)-*N*-(naphthalen-1-ylmethyl)-2-nitro-4-(trifluoromethyl)benzenesulfonamide (**74**)

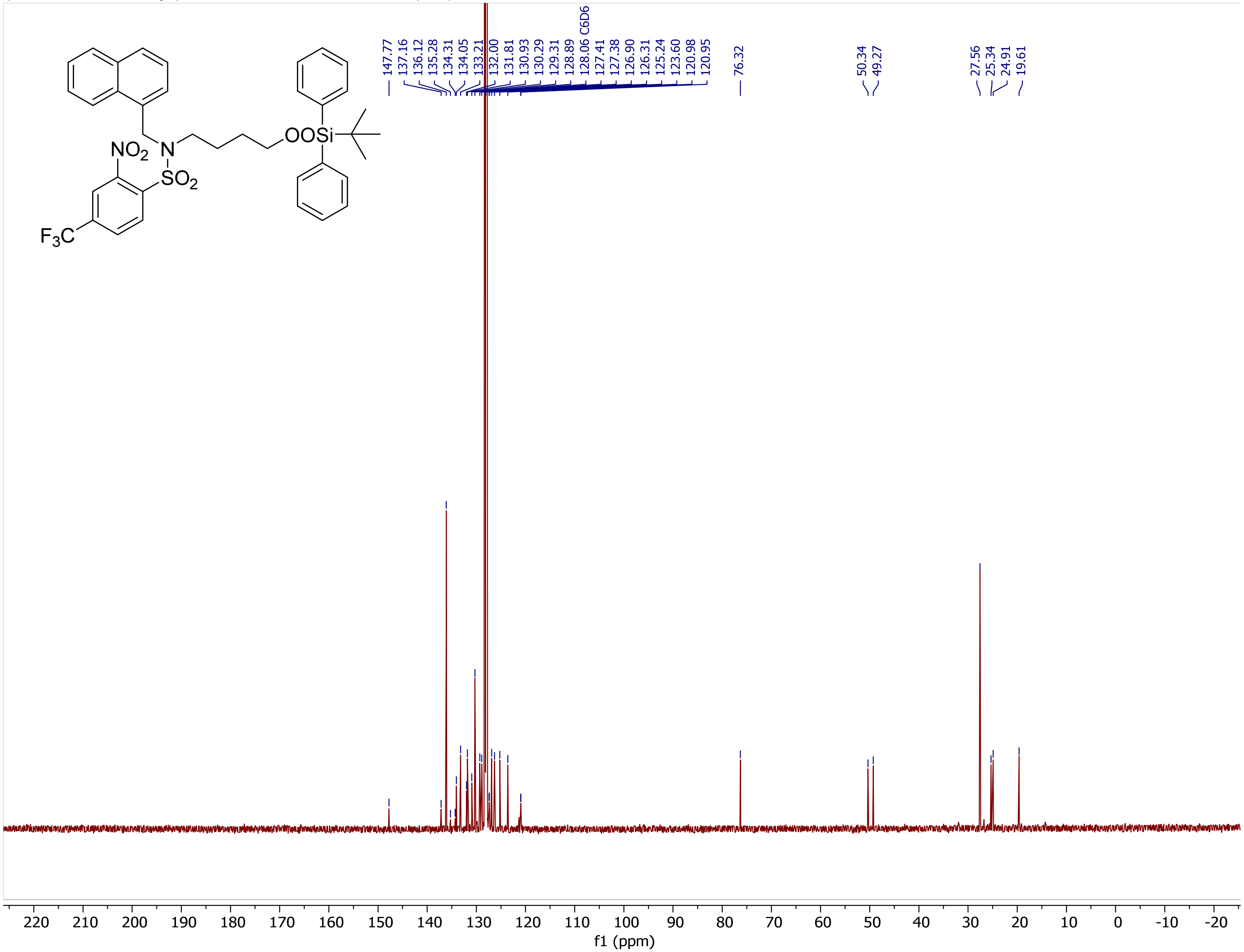

^{1}H NMR (500 MHz, C_6D_6) spectrum of 2-(Naphthalen-1-ylmethyl)-1,2-oxazinane (**76**)

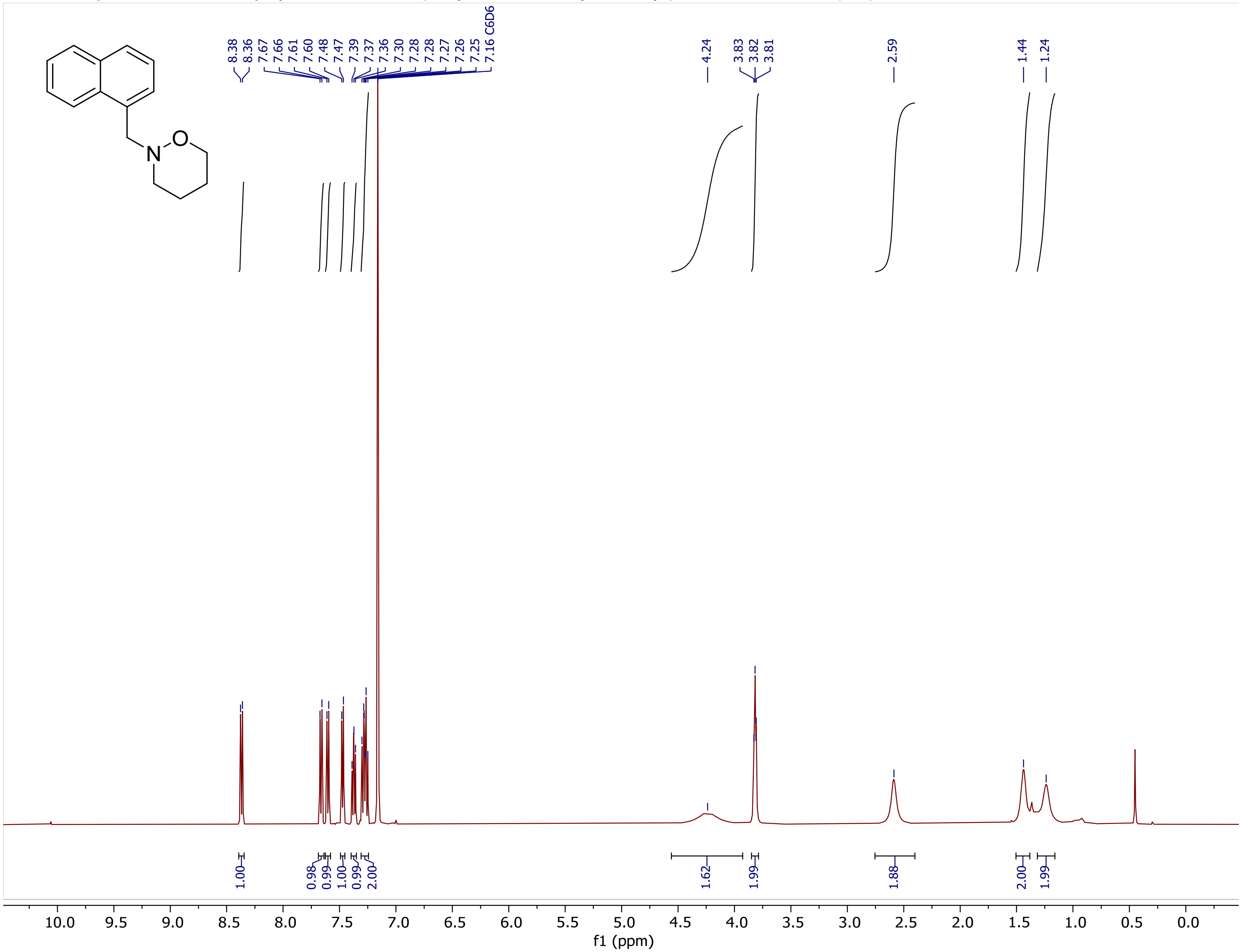

^{1}H NMR (500 MHz, C_6D_6) spectrum of 2-(Naphthalen-1-ylmethyl)-1,2-oxazinane at 65 °C (**76**)

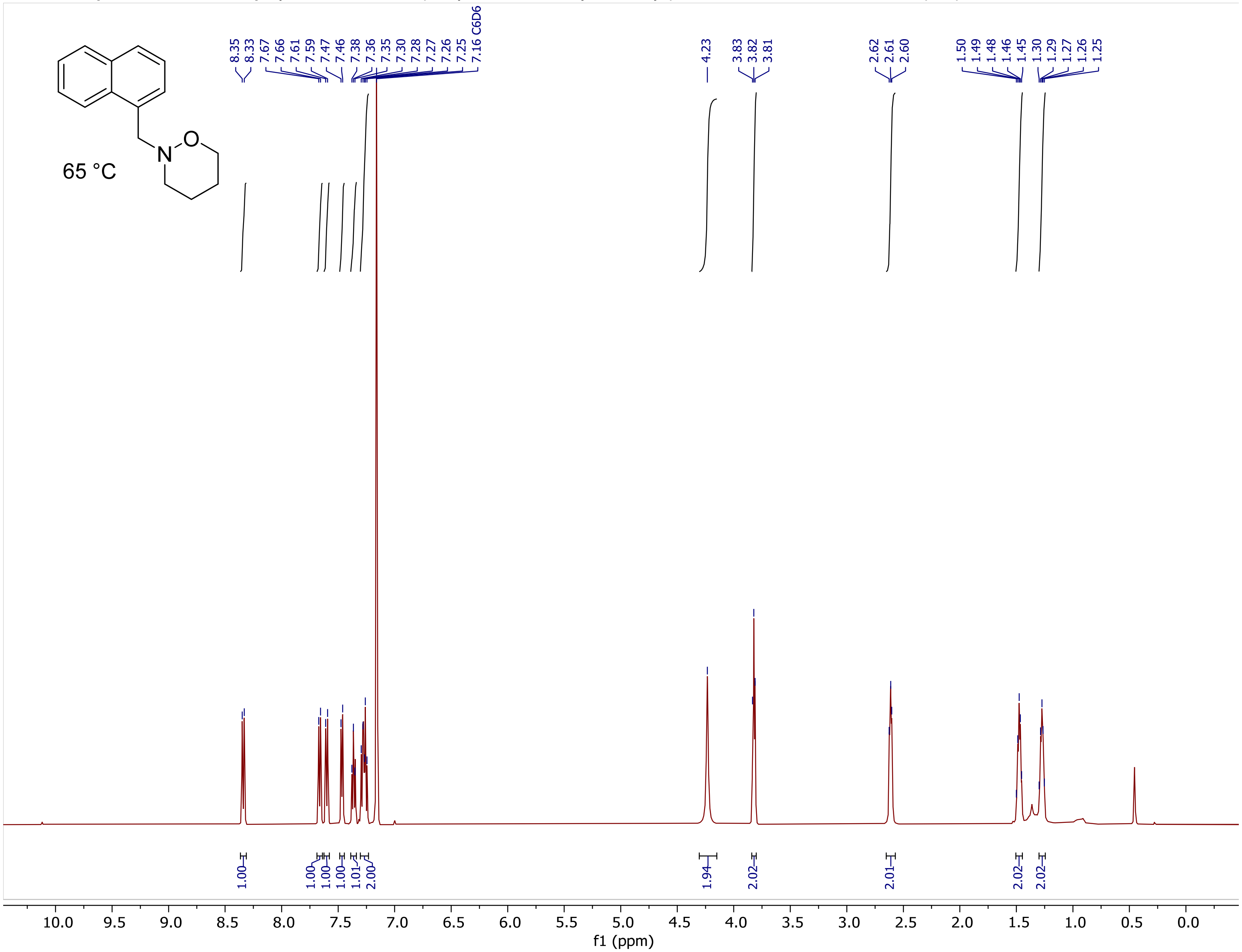

$^{13}C\{^1H\}$ NMR (126 MHz, C_6D_6) spectrum of 2-(Naphthalen-1-ylmethyl)-1,2-oxazinane (**76**)

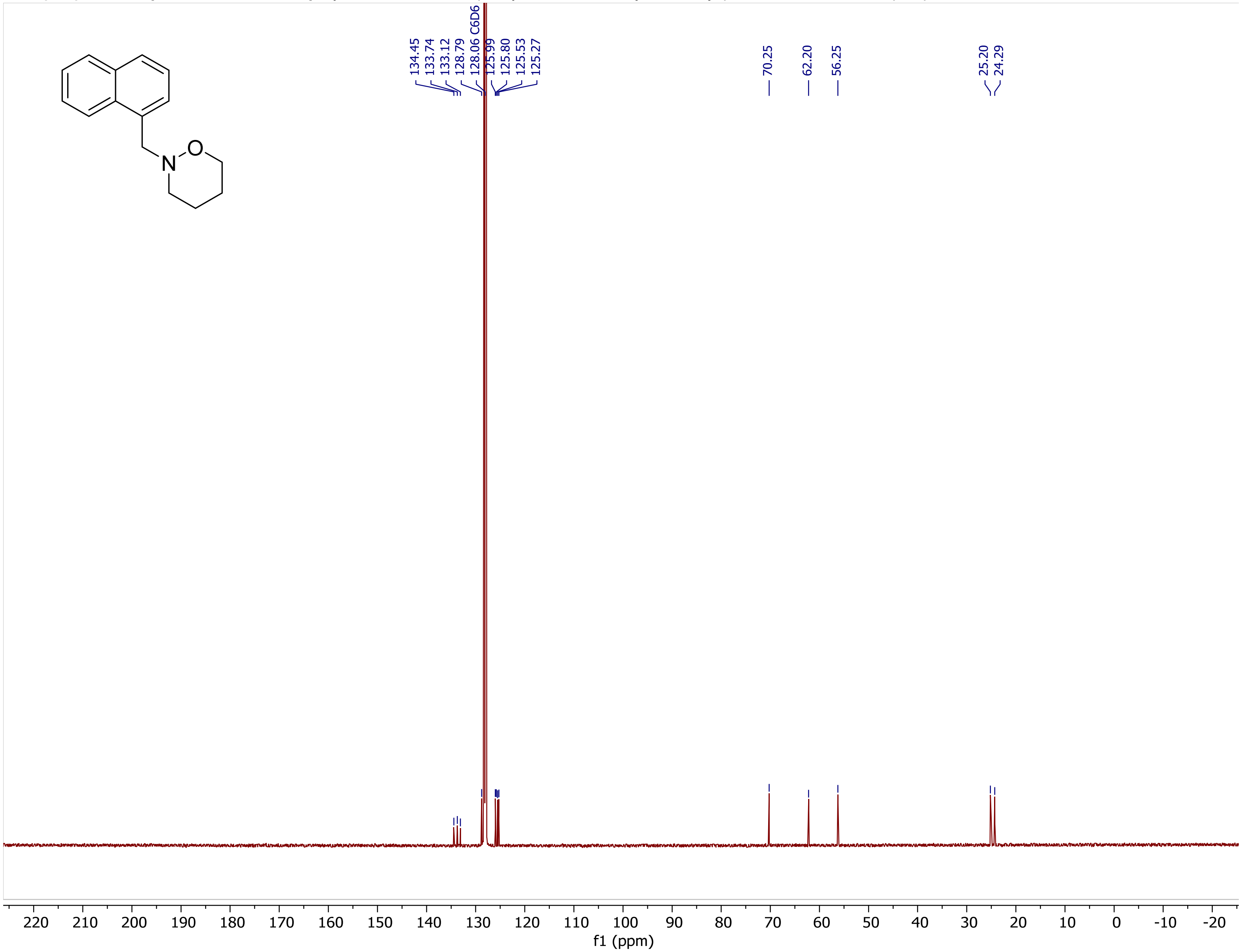

HSQC NMR (500 MHz, C_6D_6) spectrum of 2-(Naphthalen-1-ylmethyl)-1,2-oxazinane at 65 °C (**76**)

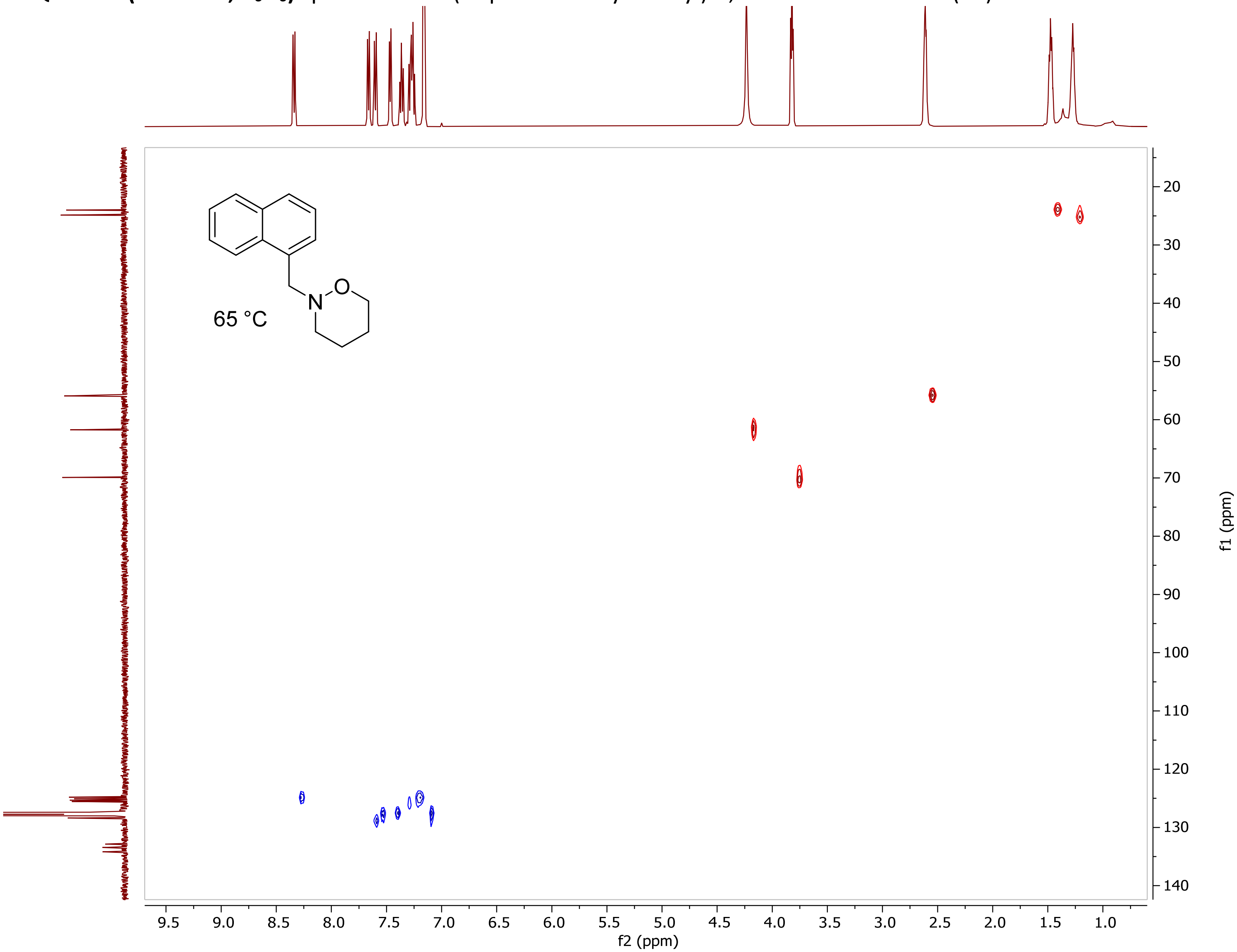

^{1}H NMR (500 MHz, CDCl$_3$) spectrum of *N*-(Naphthalen-1-ylmethyl)-2-nitro-*N*-(pent-4-en-1-yl)-4-(trifluoromethyl)benzenesulfonamide **(3o)**

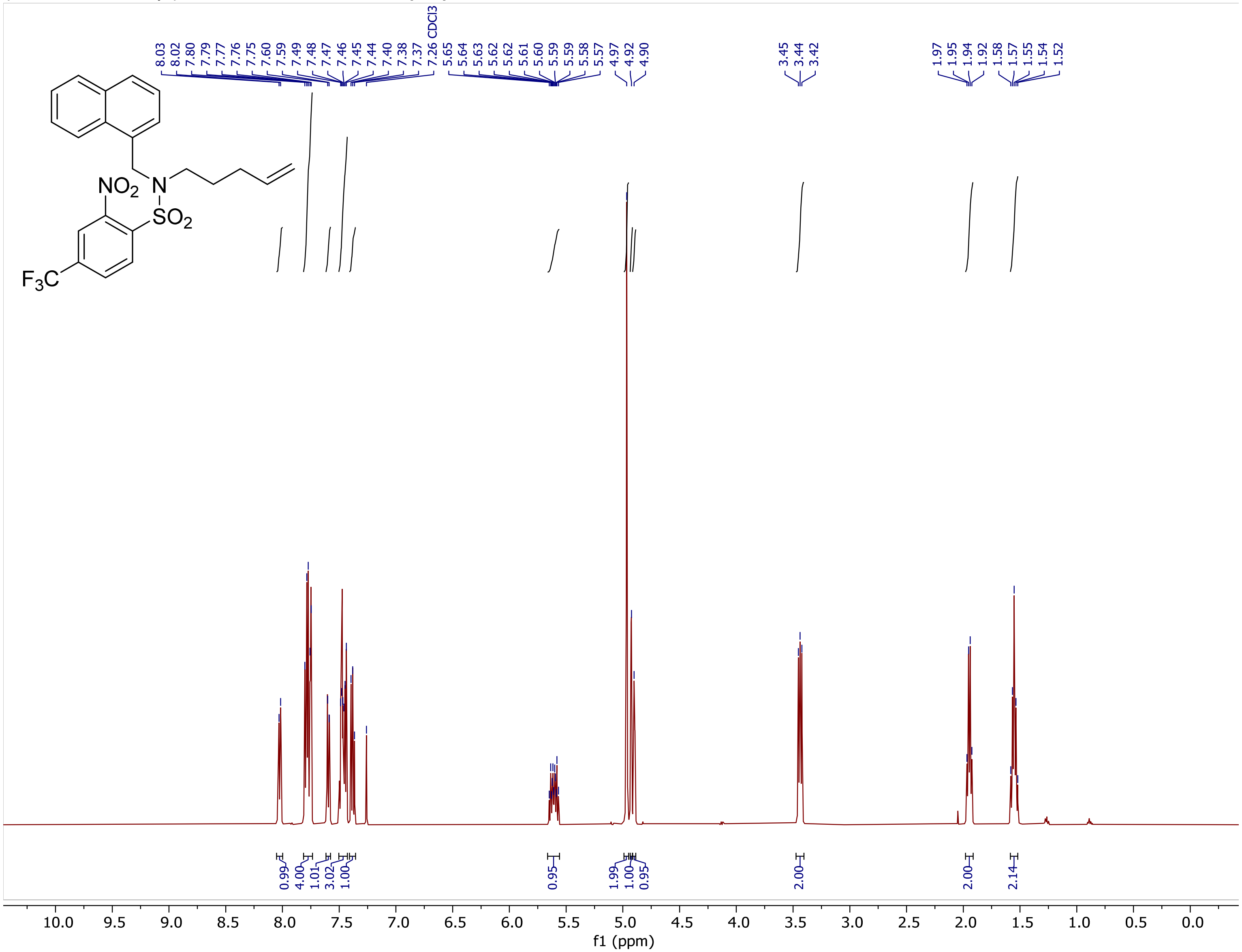

^{13}C {^{1}H} NMR (126 MHz, $CDCl_3$) spectrum of *N*-(Naphthalen-1-ylmethyl)-2-nitro-*N*-(pent-4-en-1-yl)-4-(trifluoromethyl)benzenesulfonamide **(3o)**

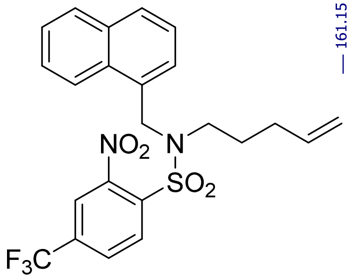

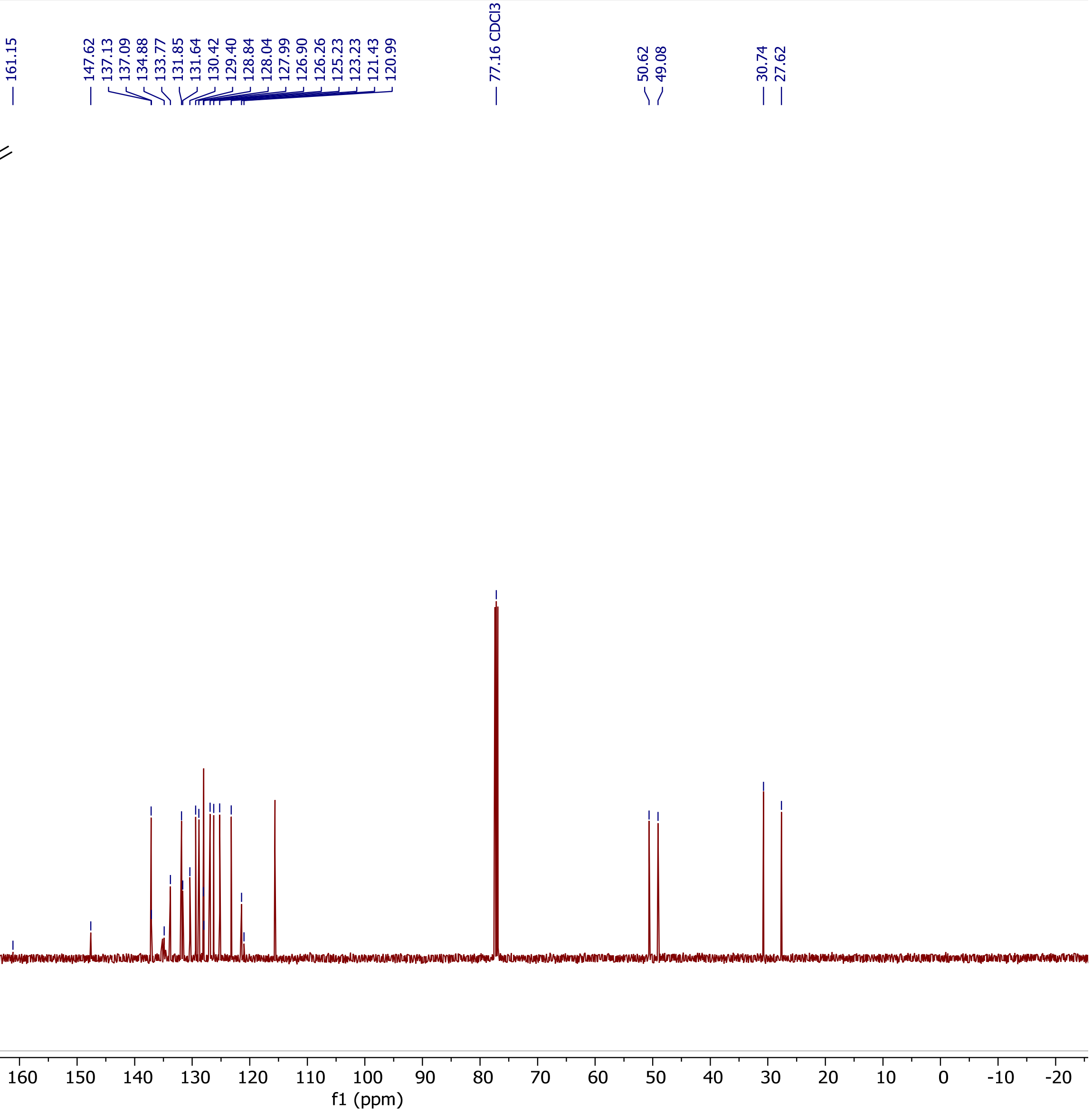

^{1}H NMR (500 MHz, CDCl$_3$) spectrum of *N*-(5-Hydzroxypentyl)-*N*-(naphthalen-1-ylmethyl)-2-nitro-4-(trifluoromethyl)benzenesulfonamide **(3p)**

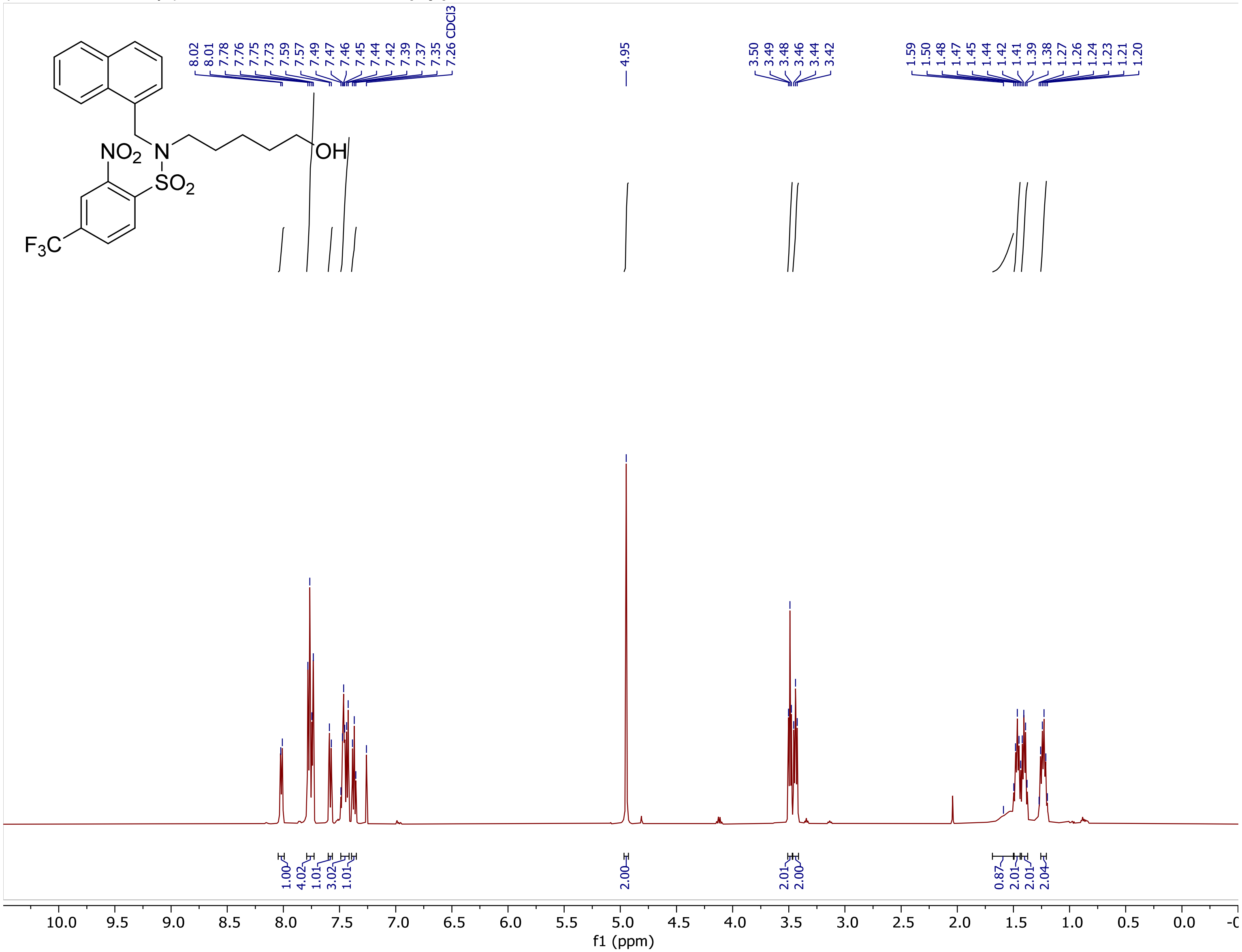

^{13}C {^{1}H, ^{19}F} NMR (126 MHz, $CDCl_3$) spectrum of *N*-(5-Hydzroxypentyl)-*N*-(naphthalen-1-ylmethyl)-2-nitro-4-(trifluoromethyl)benzenesulfonamide **(3p)**

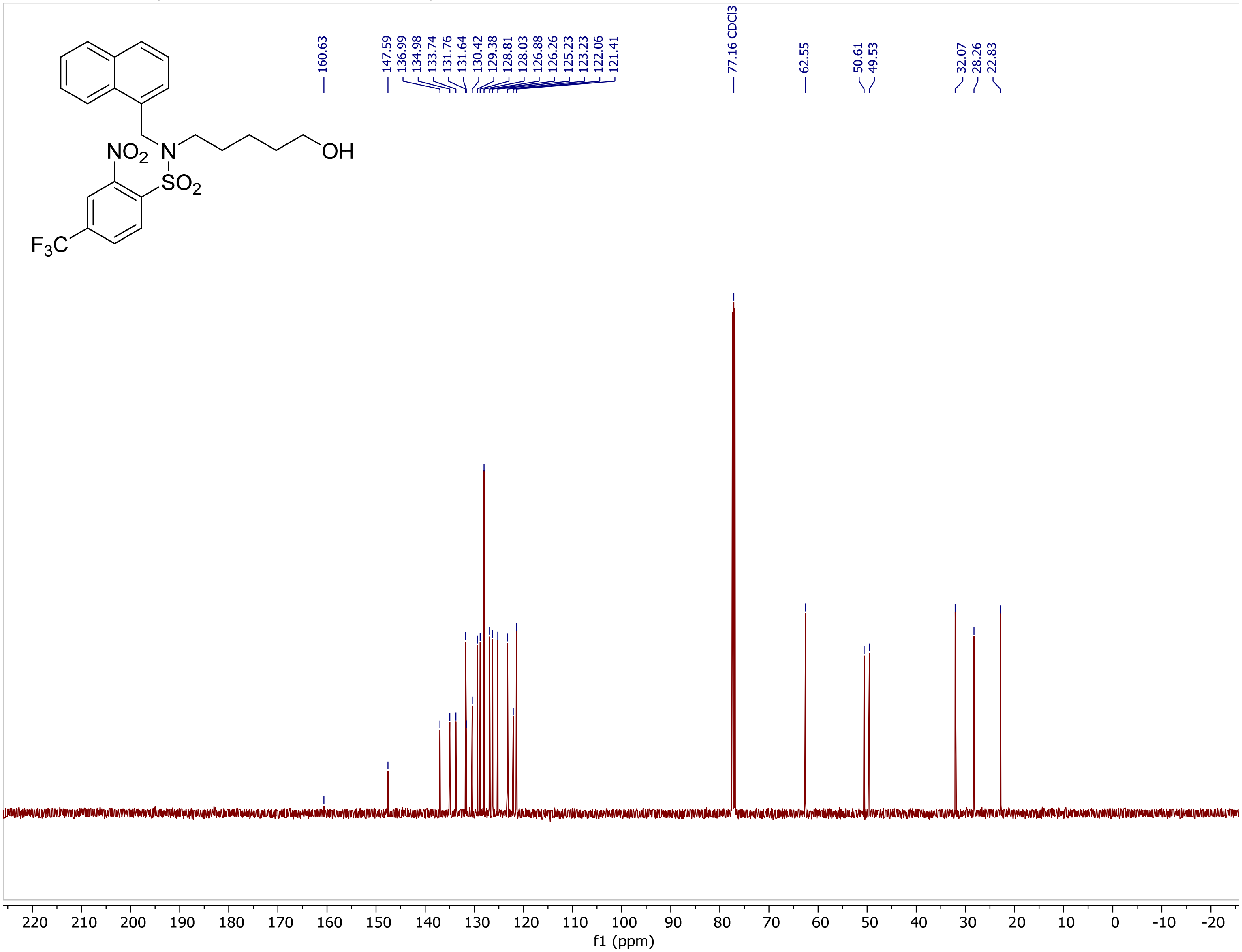

^{1}H NMR (500 MHz, $CDCl_3$) spectrum of *N*-(5-Iodopentyl)-*N*-(naphthalen-1-ylmethyl)-2-nitro-4-(trifluoromethyl)benzenesulfonamide **(3q)**

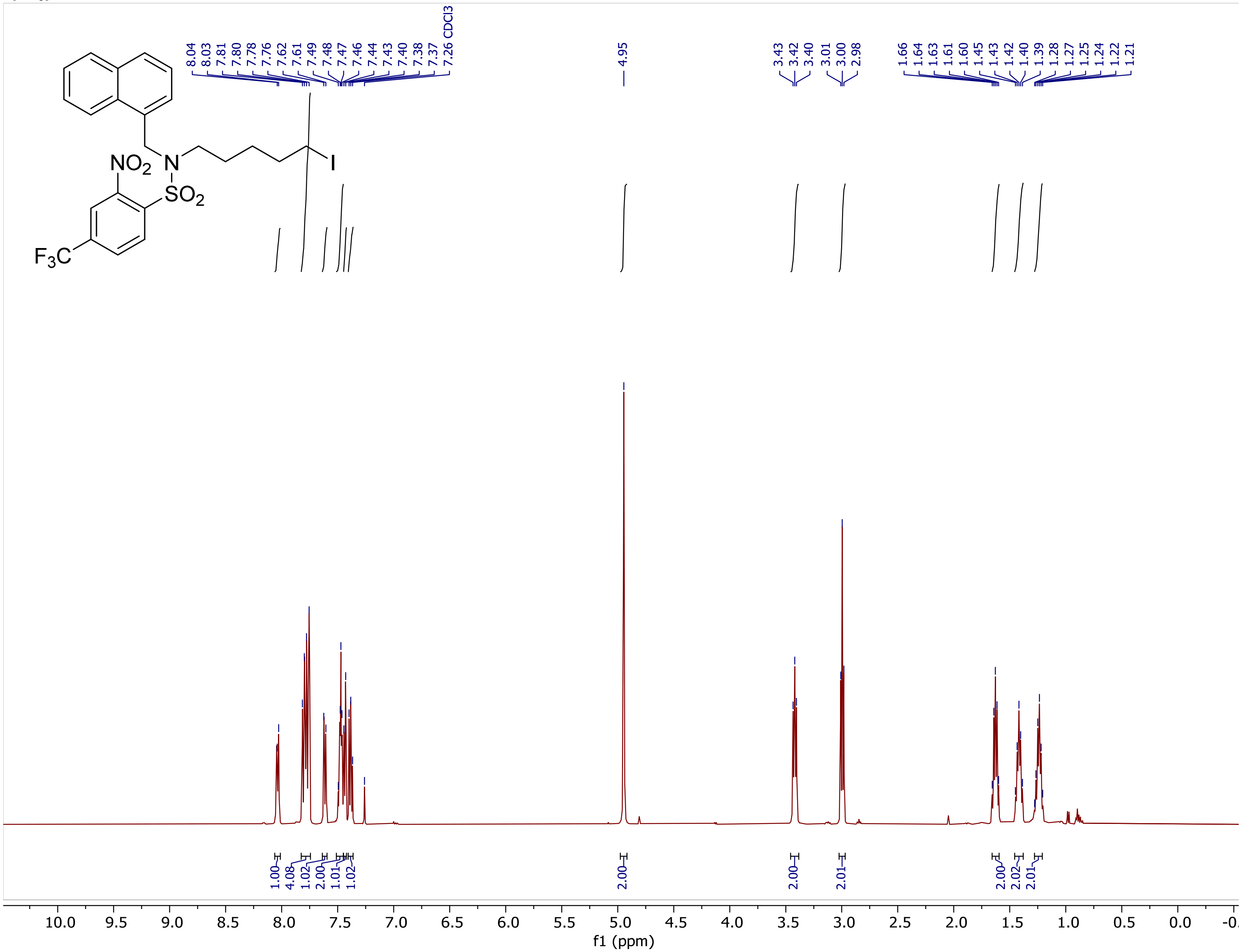

^{13}C {^{1}H, ^{19}F} NMR (126 MHz, $CDCl_3$) spectrum of *N*-(5-Iodopentyl)-*N*-(naphthalen-1-ylmethyl)-2-nitro-4-(trifluoromethyl)benzenesulfonamide **(3q)**

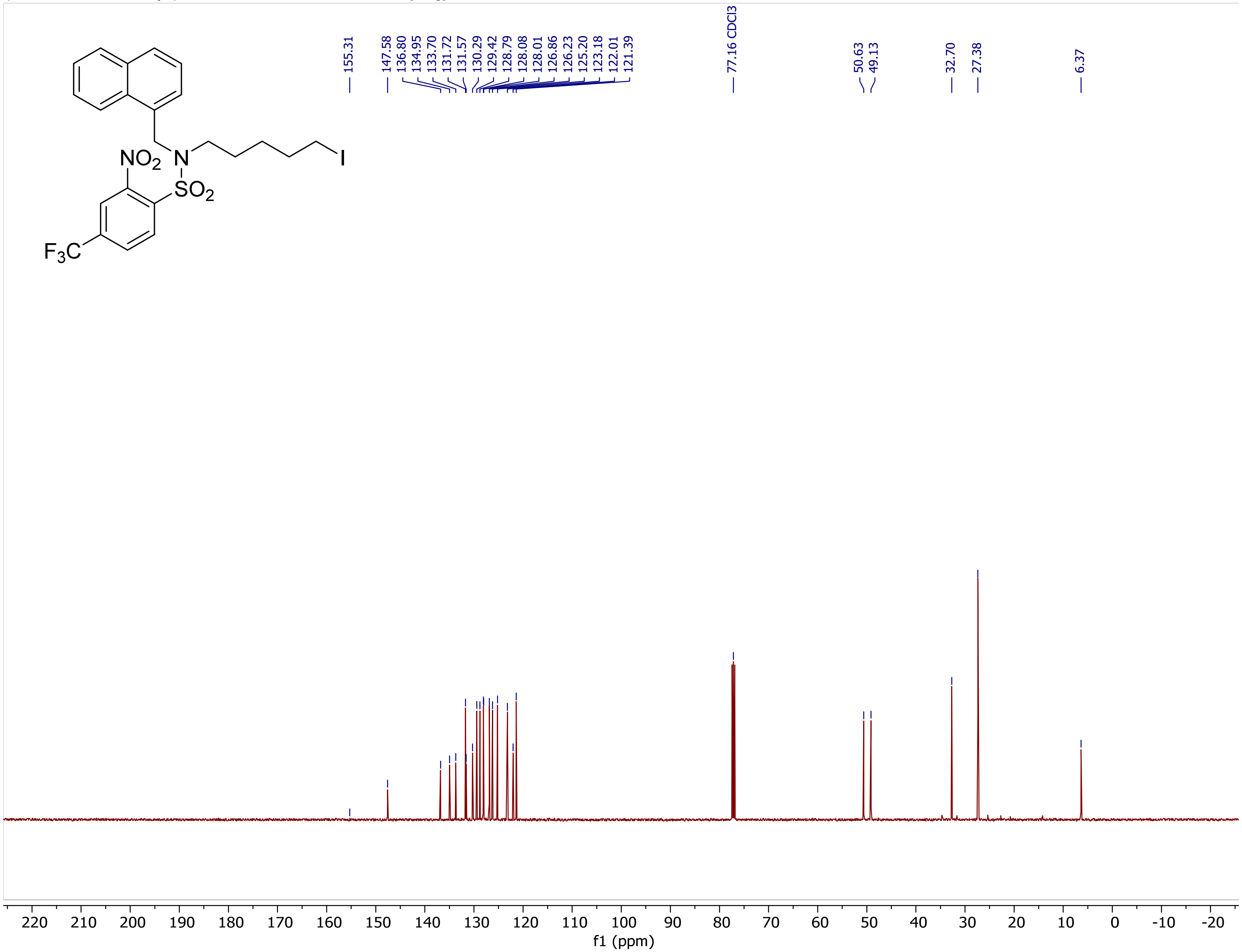

^{1}H NMR (500 MHz, C_6D_6) spectrum of *N*-(5-Hydroperoxypentyl)-*N*-(naphthalen-1-ylmethyl)-2-nitro-4-(trifluoromethyl)benzenesulfonamide **(3r)**

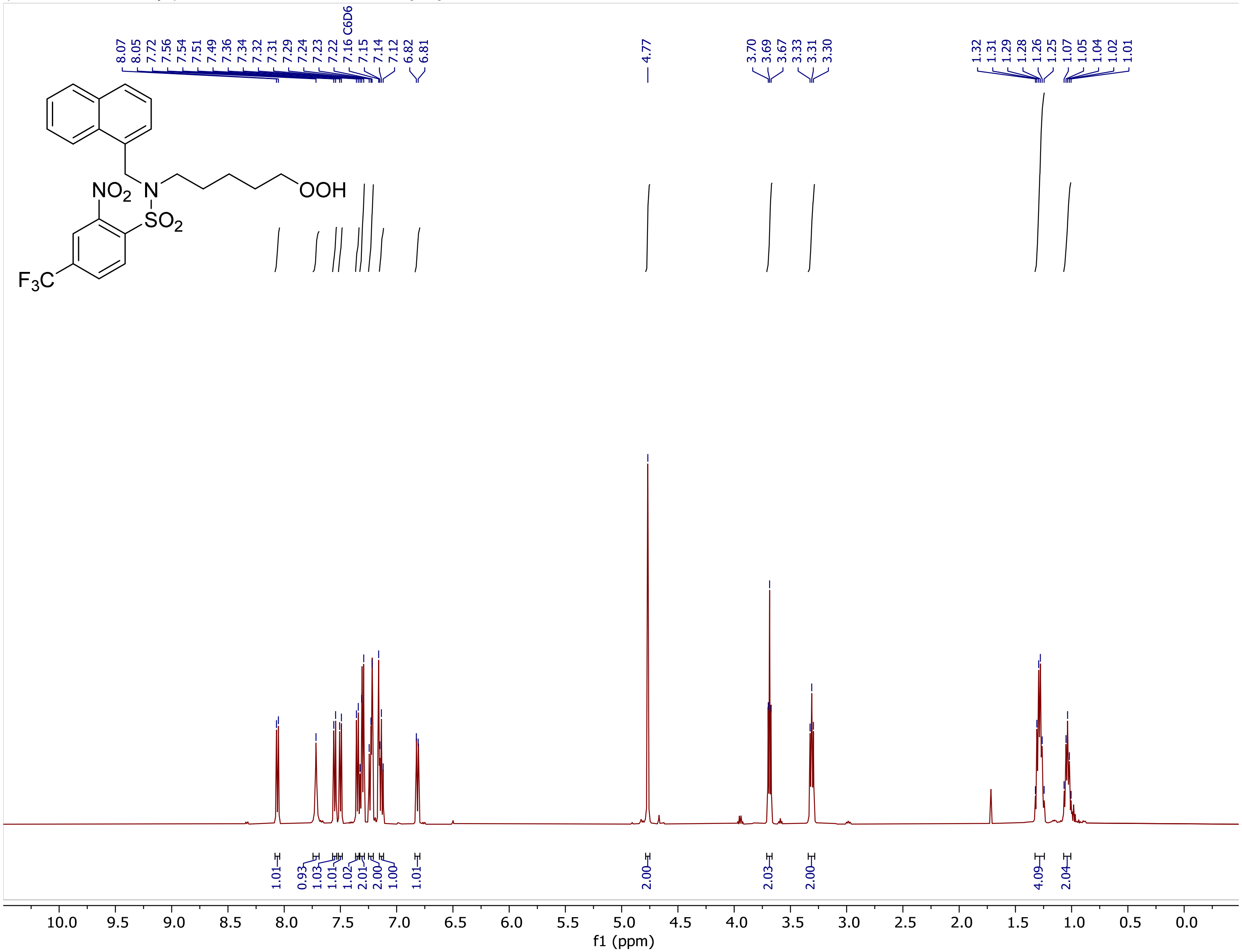

^{13}C {^{1}H, ^{19}F} NMR (126 MHz, C_6D_6) spectrum of *N*-(5-Hydroperoxypentyl)-*N*-(naphthalen-1-ylmethyl)-2-nitro-4-(trifluoromethyl)benzenesulfonamide **(3r)**

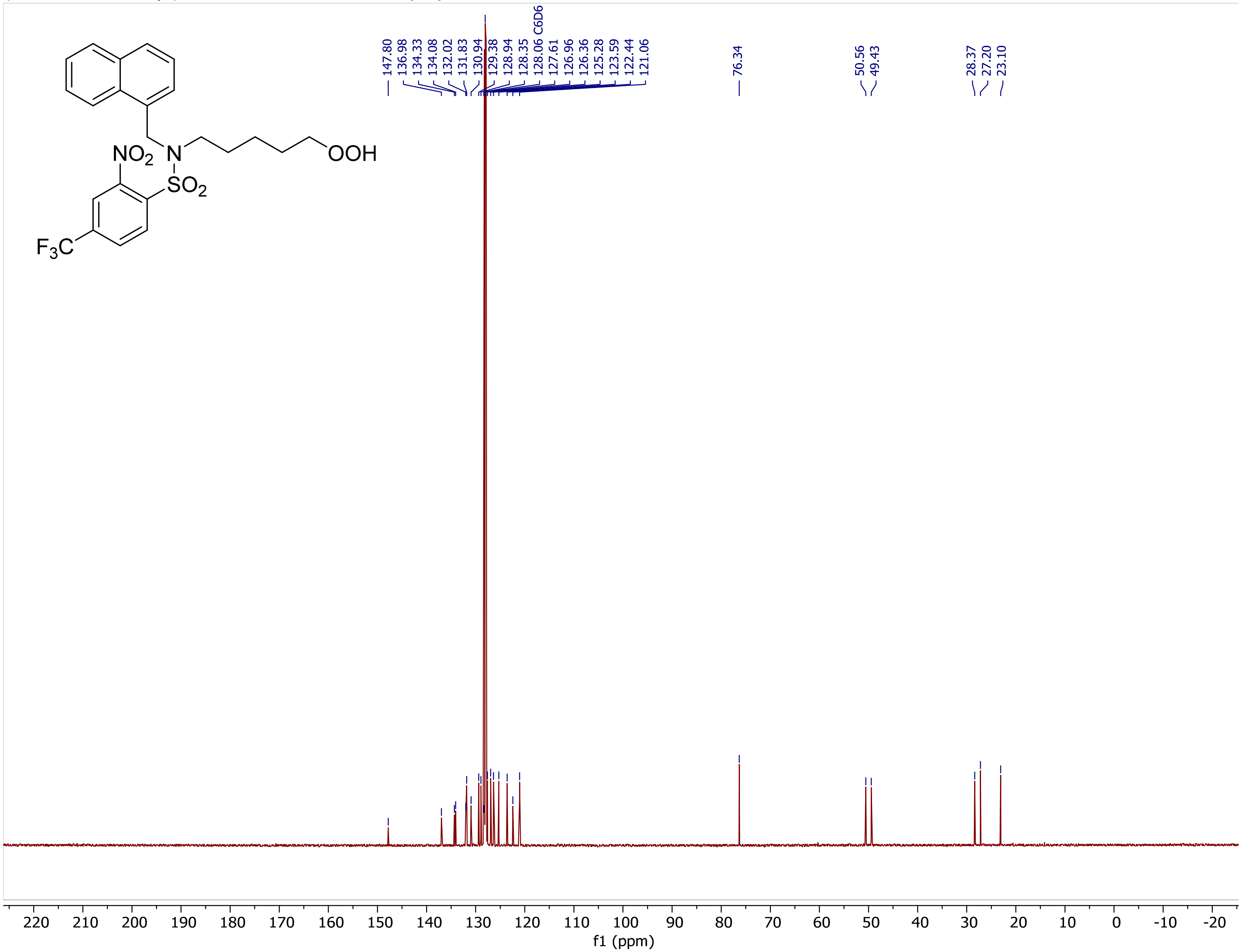

^{1}H NMR (500 MHz, C_6D_6) spectrum of *N*-(5-((*tert*-Butyldiphenylsilyl)peroxy)pentyl)-*N*-(naphthalen-1-ylmethyl)-2-nitro-4-(trifluoromethyl)benzenesulfonamide **(75)**

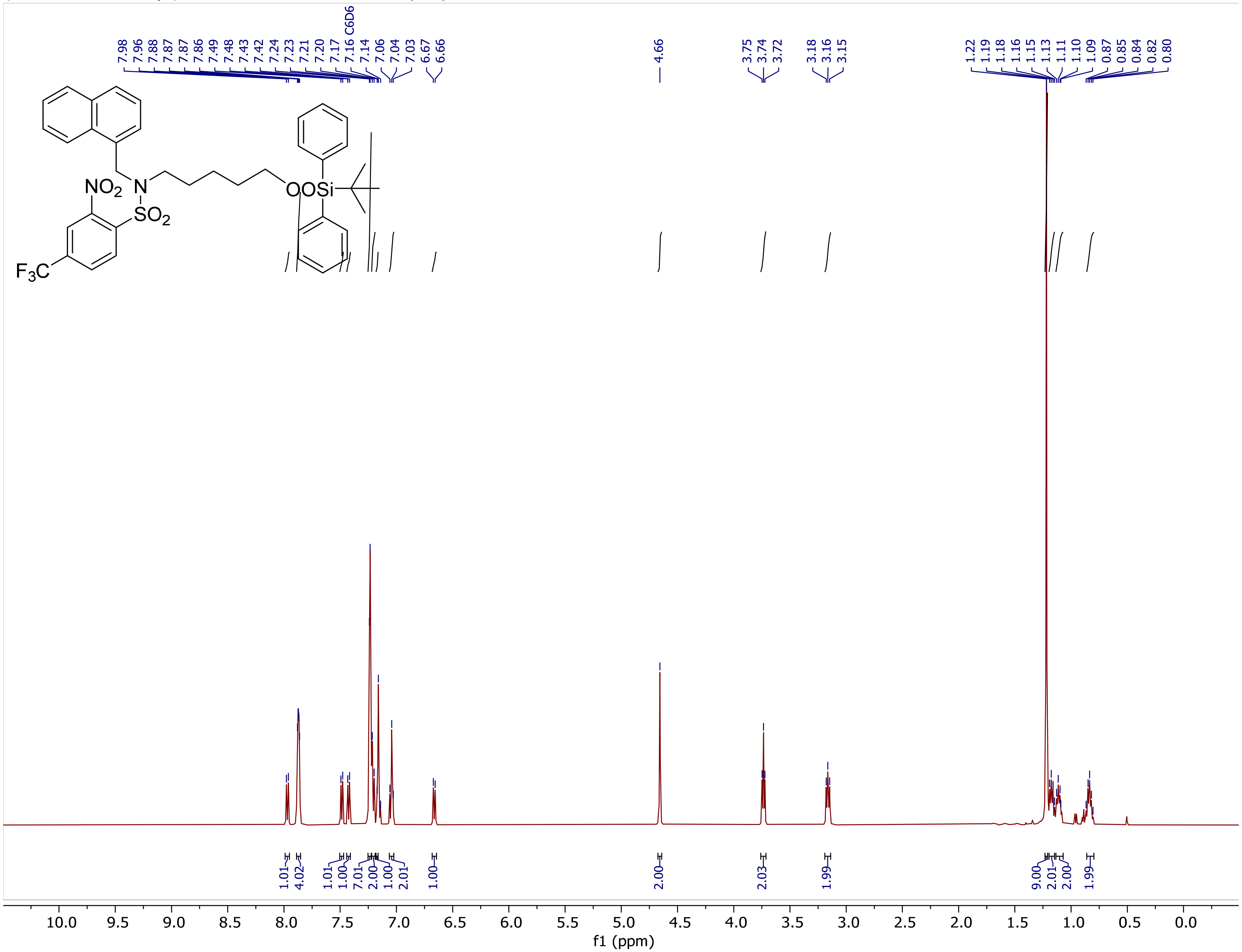

^{13}C {^{1}H, ^{19}F} NMR (126 MHz, C_6D_6) spectrum of *N*-(5-((*tert*-Butyldiphenylsilyl)peroxy)pentyl)-*N*-(naphthalen-1-ylmethyl)-2-nitro-4-(trifluoromethyl)benzenesulfonamide **(75)**

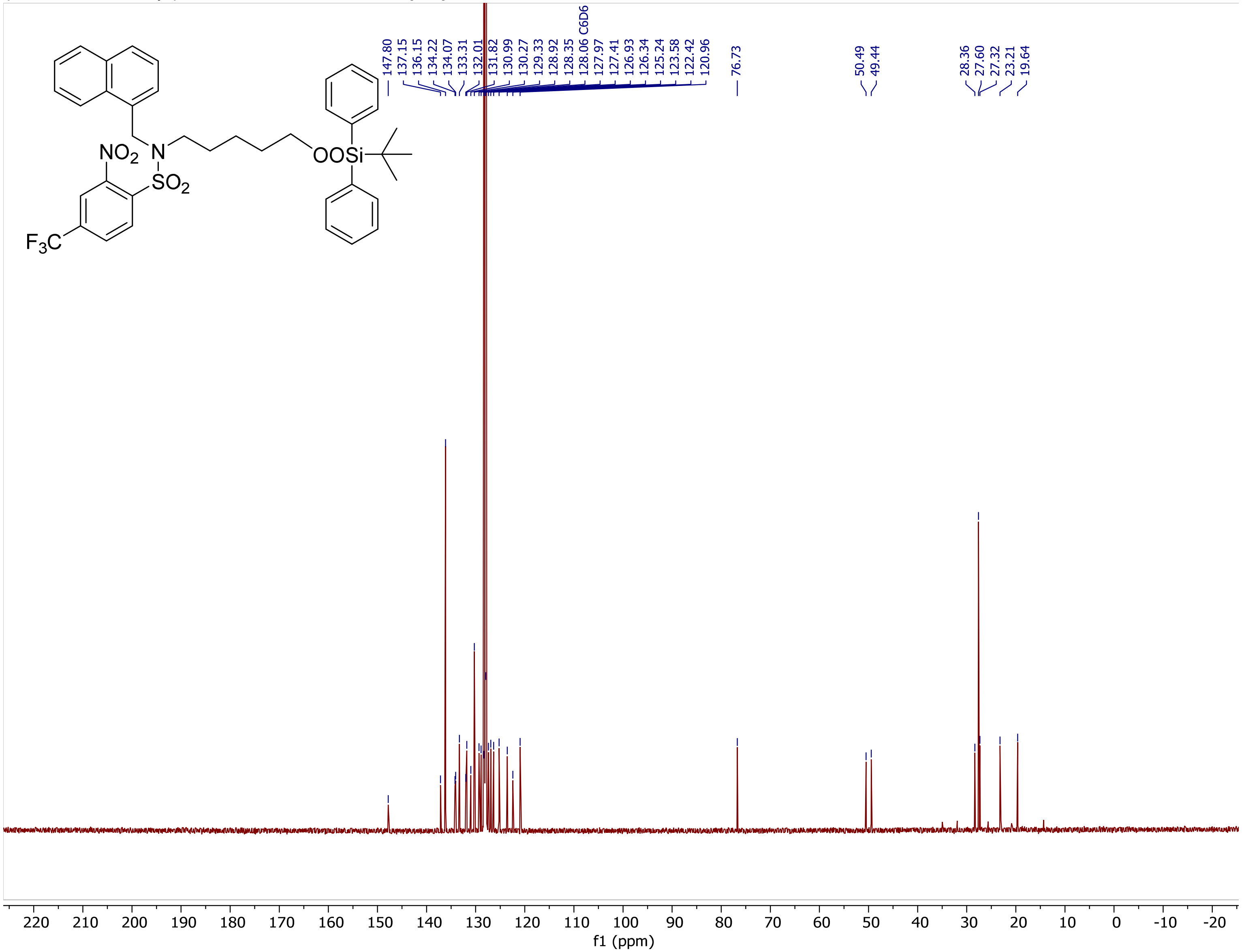

^{1}H NMR (500 MHz, $CDCl_3$) spectrum of 2-((2-Nitro-4-(trifluoromethyl)phenyl)thio)ethan-1-ol (**82**)

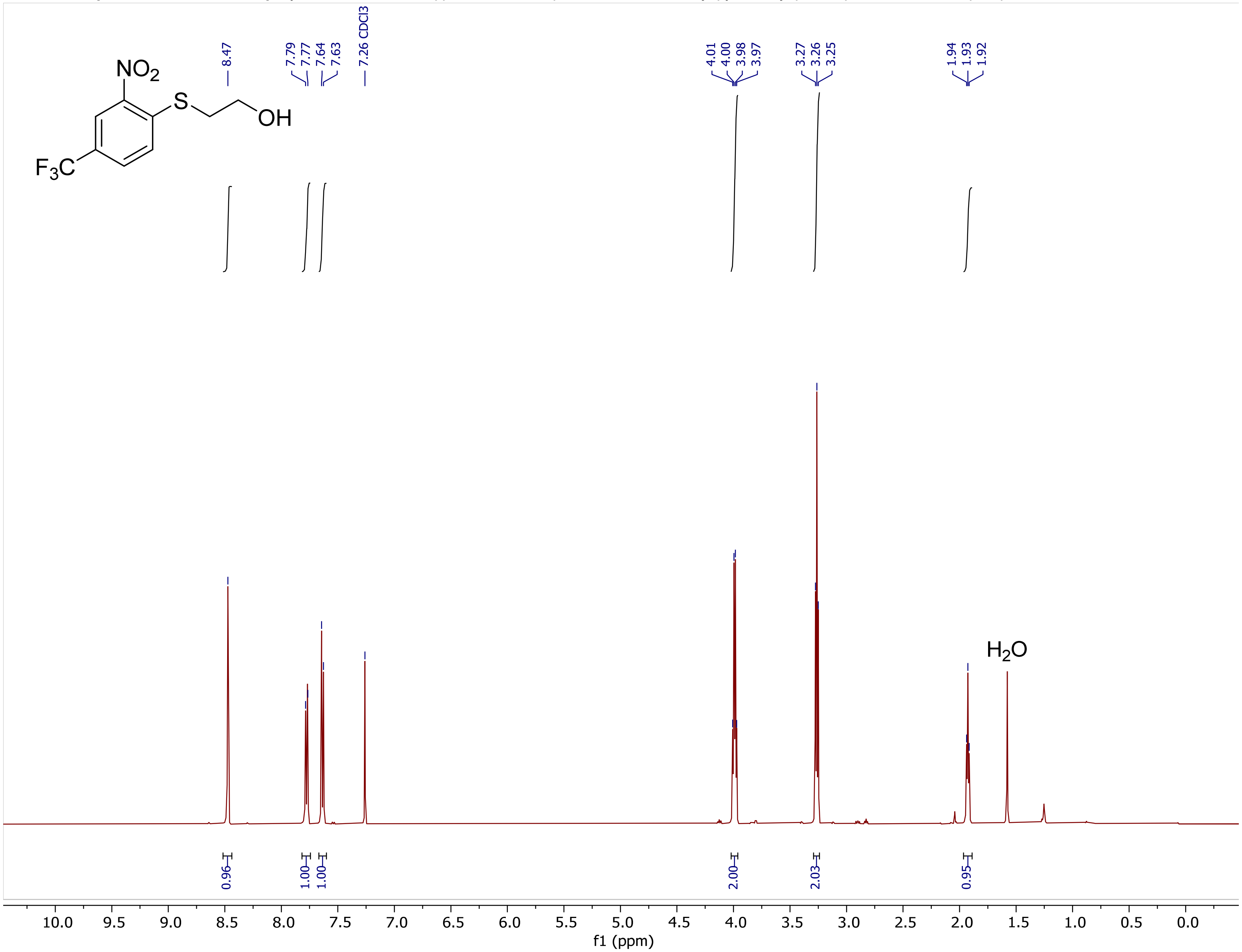

$^{13}C\{^1H\}$ NMR (126 MHz, $CDCl_3$) spectrum of 2-((2-Nitro-4-(trifluoromethyl)phenyl)thio)ethan-1-ol (**82**)

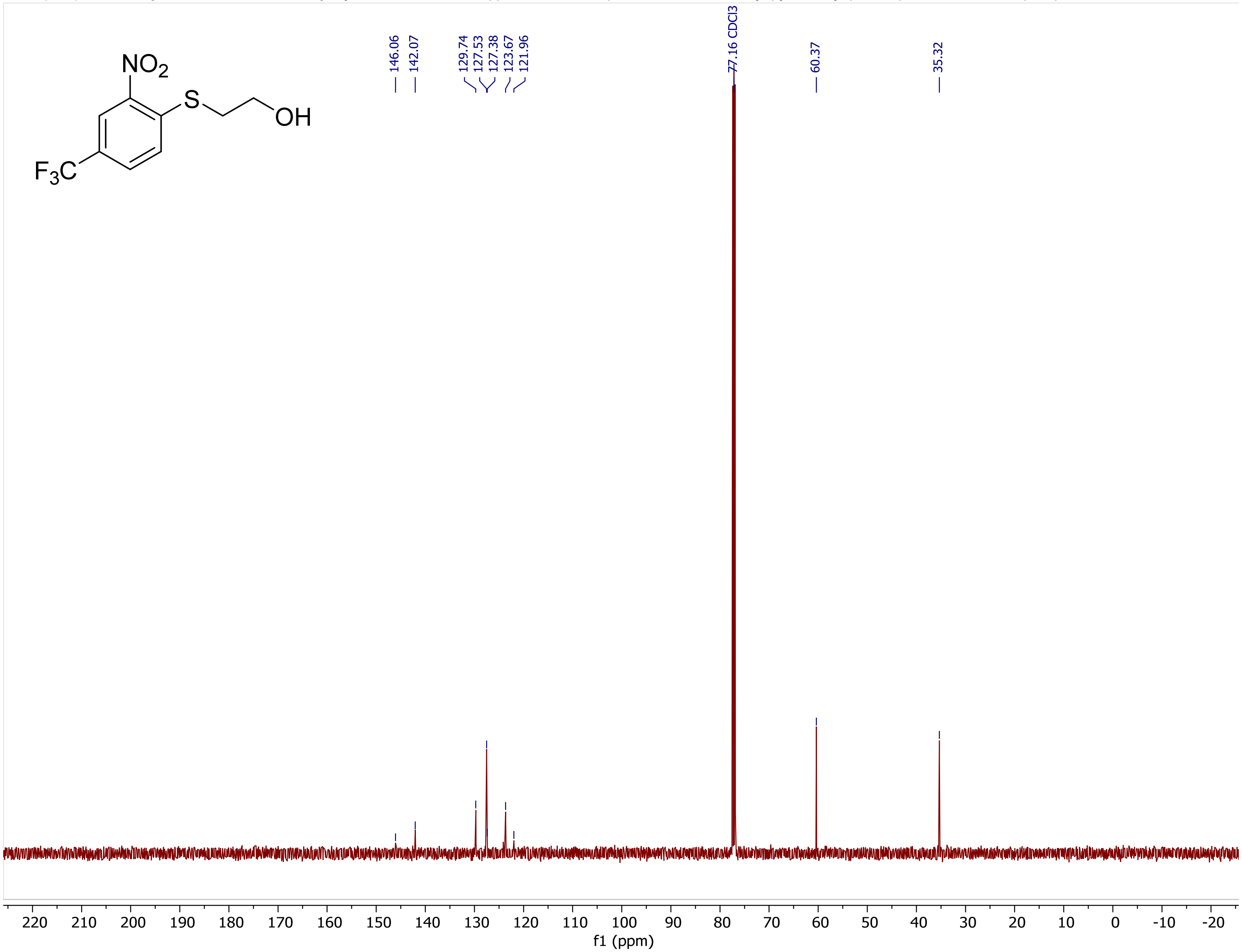

10) ***Barrier to Nitrogen Inversion VT Spectra***

Variable temperature ^{1}H NMR study of Isoxazolidine 25 for the barrier of inversion to Nitrogen

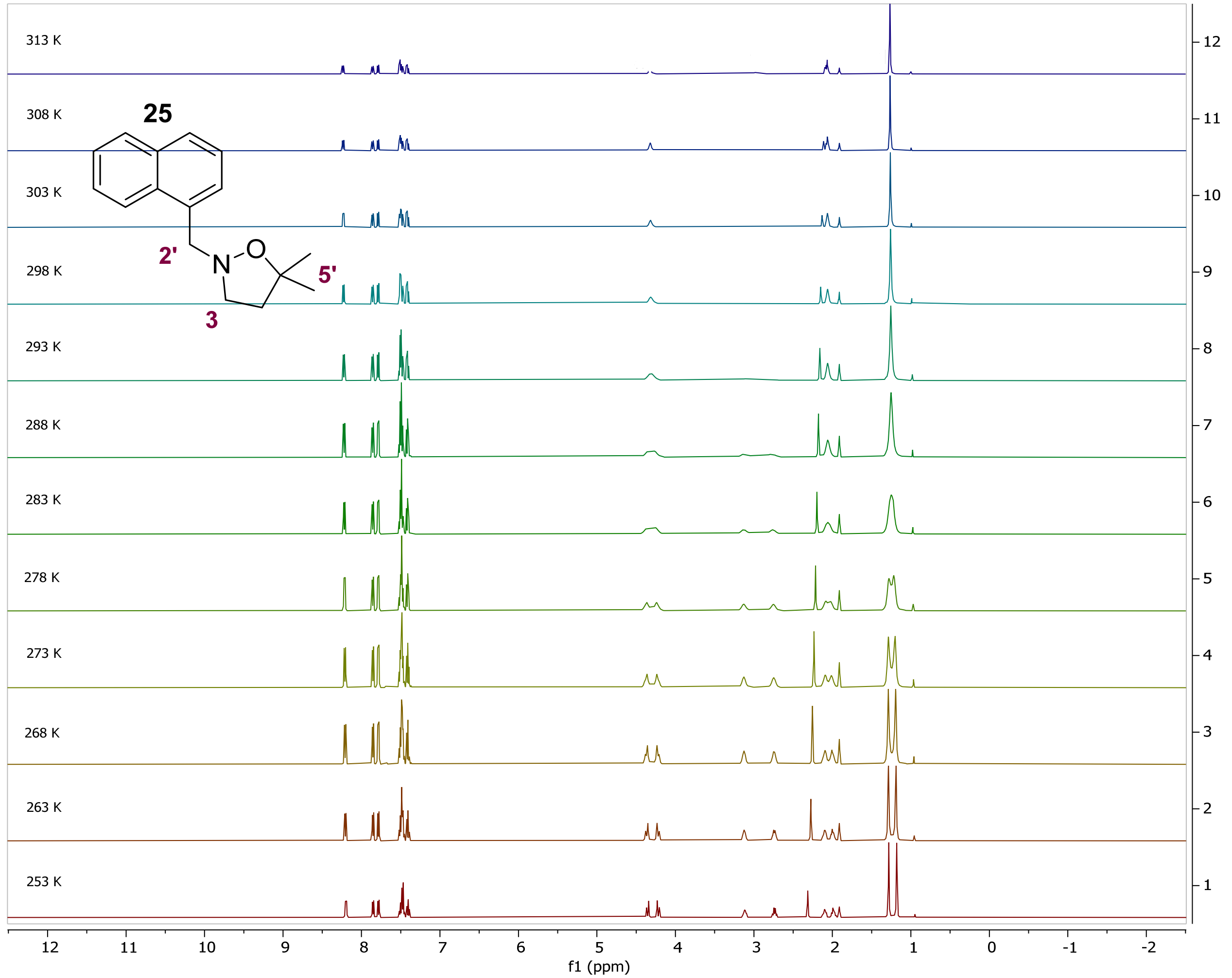

VT figure 1: ^{1}H NMR spectra ([D$_3$] MeCN, 500 MHz) of **25** recorded at different temperatures ranging from −20 °C to 75 °C

The free energy of activation ($\Delta G_c^{\neq}$ (kcal mol^{-1}) for the observed dynamic process at the coalescence temperature (T_c in K) was estimated by measuring the maximum frequency difference between the signals of the two exchanging species at low temperature (Δv in Hz) by application of Equation: $\Delta G_c^{\neq} = 4.575 \times 10^{-3} T_C[9.972 + \log(T_C/\Delta \nu)]$.

Variable temperature ¹H NMR (500 MHz, C_2D_3N) spectrum of 5,5-Dimethyl-2-(naphthalen-1-ylmethyl)isoxazolidine (**25**) at **$H_{2'}$**

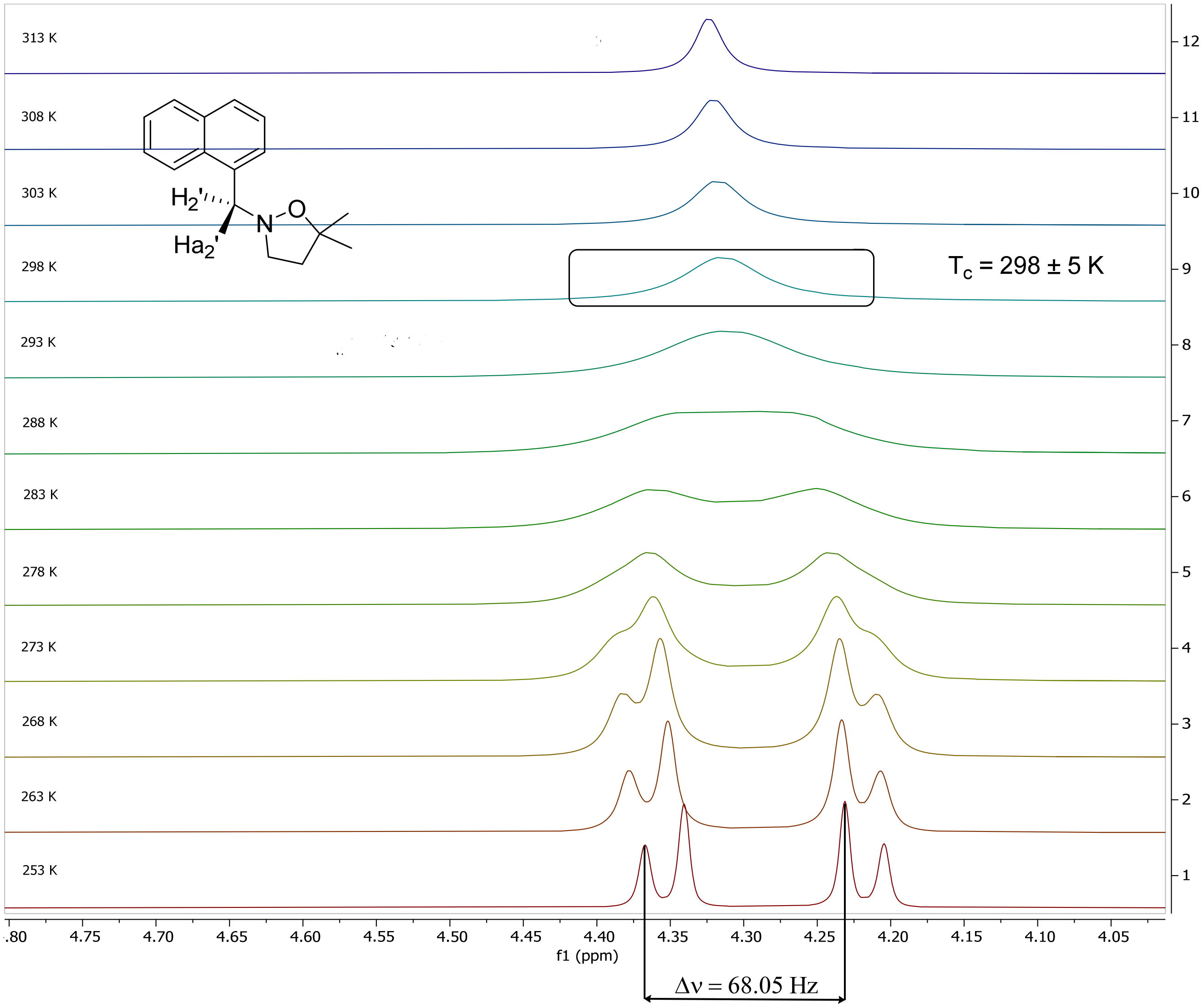

VT figure 2: Selected region of the ¹H NMR spectra ([D₃] MeCN, 500 MHz) of **25** recorded at different temperatures ranging from −20 °C to 40 °C (δ=4.80–4.05 ppm; benzylic protons $H_{2'}$). $\Delta G_c^{\neq}$ = 14.5 kcal mol^{-1}

Variable temperature ^{1}H NMR (500 MHz, C_2D_3N) spectrum of 5,5-Dimethyl-2-(naphthalen-1-ylmethyl)isoxazolidine (**25**) at **H_3**

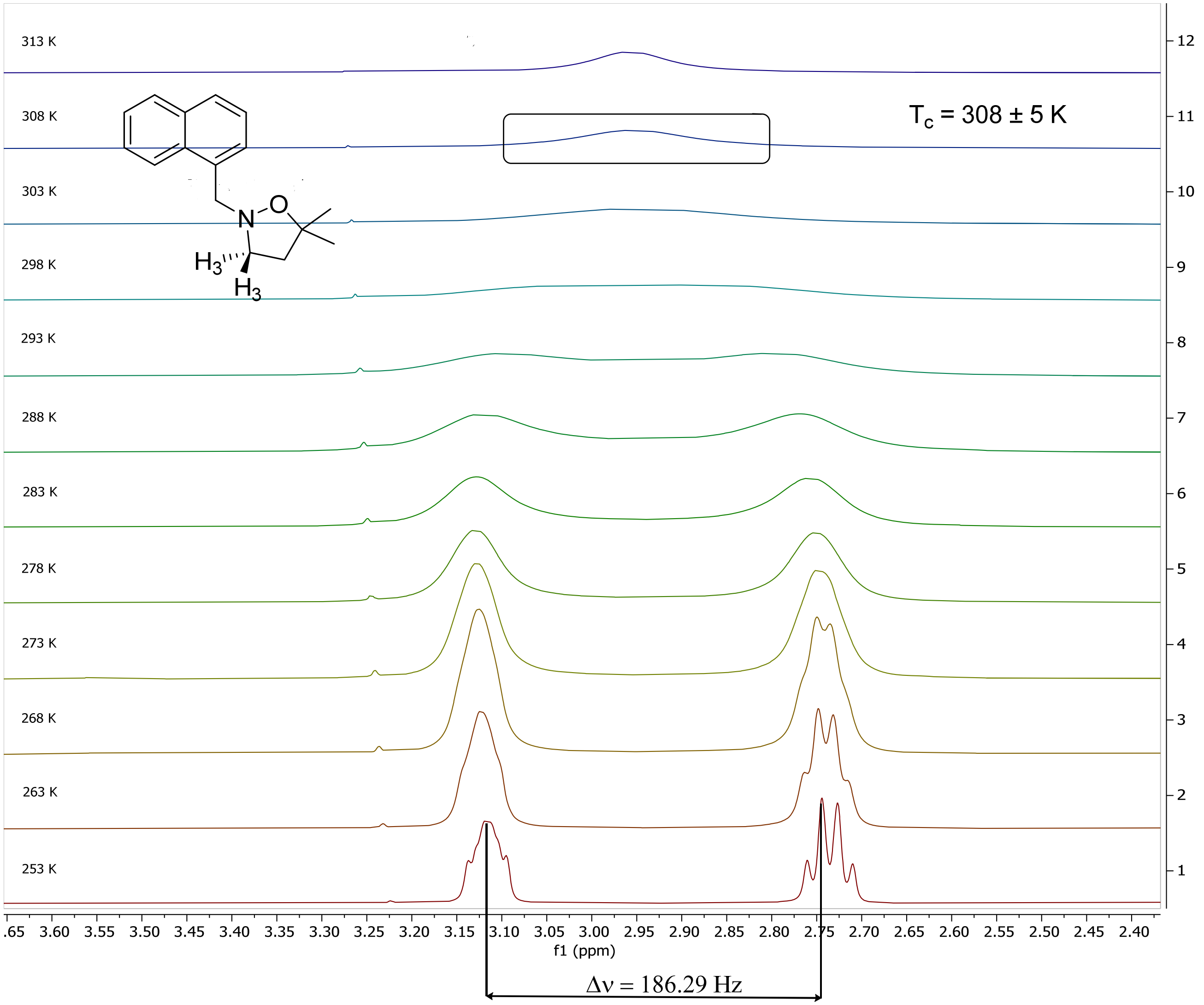

VT figure 3: Selected region of the ^{1}H NMR spectra ([D$_3$] MeCN, 500 MHz) of **25** recorded at different temperatures ranging from −20 °C to 40 °C (δ=3.60–2.40 ppm; cyclic protons H_3). $\Delta G_c^{\neq}$ = 14.4 kcal mol^{-1}

Variable temperature ^{1}H NMR (500 MHz, C_2D_3N) spectrum of 5,5-Dimethyl-2-(naphthalen-1-ylmethyl)isoxazolidine (**25**) at **$H_{5'}$**

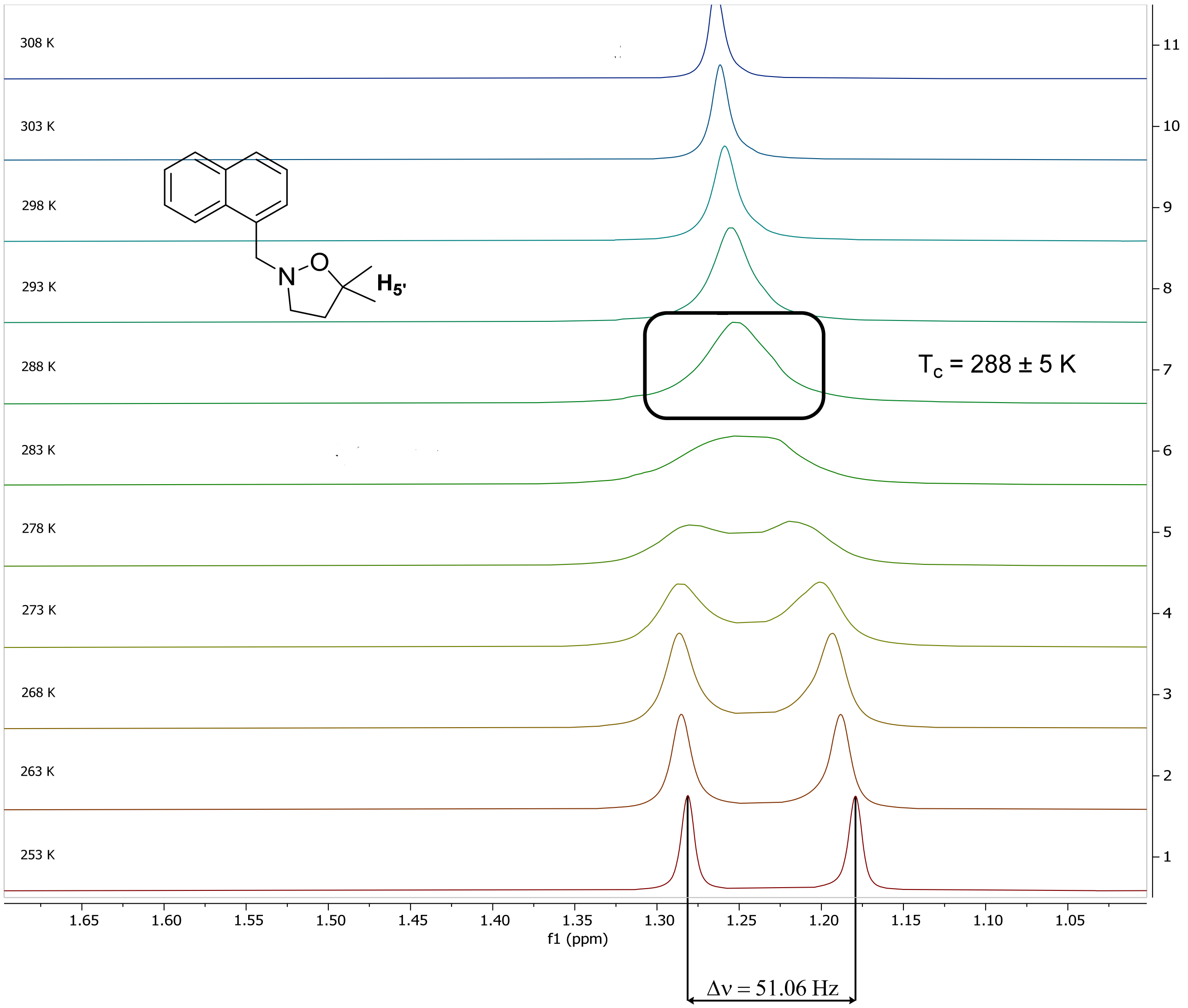

***VT figure 4**:* Selected region of the ^{1}H NMR spectra ([D$_3$] MeCN, 500 MHz) of **25** recorded at different temperatures ranging from −20 °C to 35 °C (δ=1.68–1.03 ppm; methyl protons $H_{5'}$). $\Delta G_c^{\neq}$ = 14.1 kcal mol^{-1}

11) X-Ray Data of 58

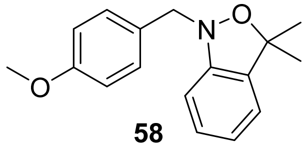

58

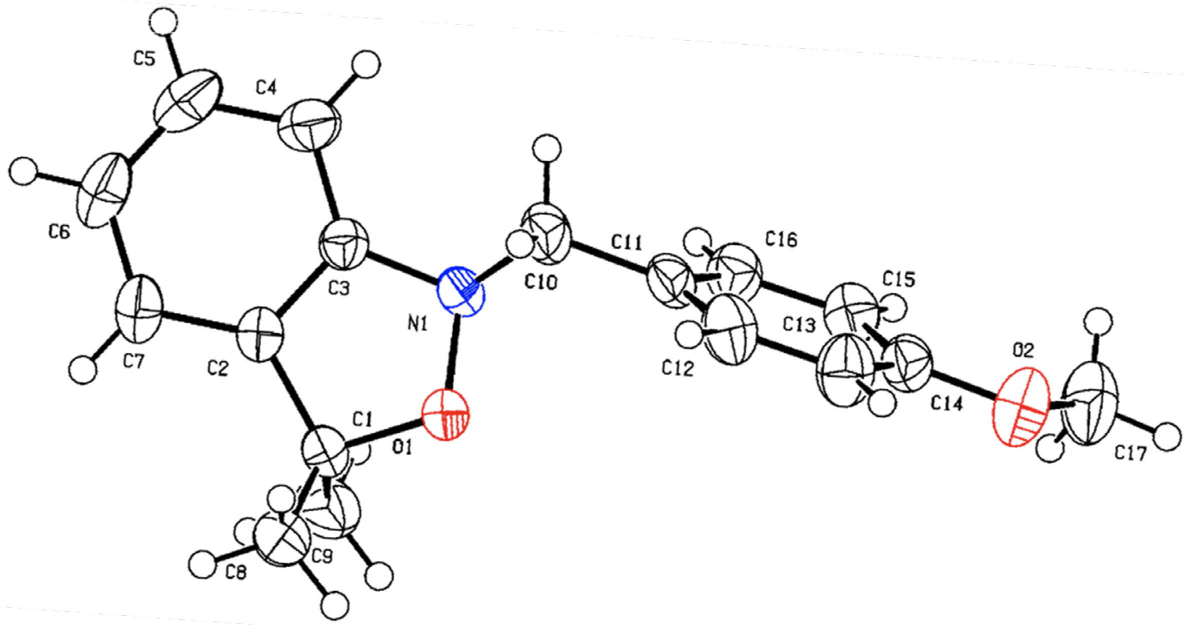

Figure S1. Ortep drawing of **58** with 30% probability for thermal ellipsoids.

Crystal Preparation: Title compound **58** was dissolved in isopropanol by 5 minutes of sonication followed by gentle heating (40 °C). Once fully dissolved, slow evaporation of the solvent over 5 days facilitated recrystallization.

Data collection: A specimen of $C_{17}H_{19}NO_2$, approximate dimensions 0.150 mm x 0.360 mm x 0.420 mm in a colorless crystal was used for the X-ray crystallographic analysis. The X-ray intensity data were measured at room temperature (299 K) on a Bruker D8 Quest PHOTON 100 CMOS X-ray diffractometer system with Incoatec Microfocus Source (IµS) monochromated Mo Ka radiation (l = 0.71073 Å, sealed tube) using phi and omega-scan technique.

The integration of the data using a monoclinic unit cell yielded a total of 69308 reflections to a maximum θ angle of 35.63° (0.61 Å resolution), of which 6795 were independent (average redundancy 10.200, completeness = 99.9%, R_{int} = 6.74%, R_{sig} = 3.89%) and 4167 (61.32%) were greater than 2σ(F^2). The final cell constants of a = 8.7040(4) Å, b = 10.3239(5) Å, c = 16.5399(8) Å, β = 96.819(2)°, volume = 1475.75(12) $Å^3$, are based upon the refinement of the XYZ-centroids of 9770 reflections above 20 σ(I). The data were integrated with the manufacturer's SAINT software and corrected for absorption effects using the Multi-Scan method (SADABS). The calculated minimum and maximum transmission coefficients (based on crystal size) are 0.6082 and 0.7471.

The structure was solved and refined using the Bruker SHELXTL Software Package[17], using the space group P21/c (No. 14), with Z = 4 for the formula unit, $C_{17}H_{19}NO_2$. Non-hydrogen atoms were located from successive difference Fourier map calculations. In the final cycles of each refinement, all the non-hydrogen atoms were refined in anisotropic displacement parameters. All the hydrogen atom positions were calculated and allowed to ride on the carbon to which they are bonded assuming a C–H bond length of m Å (m = 0.930 for Ph-H groups, m = 0.970 for CH_2 groups, m = 0.960 for CH_3 groups). Hydrogen atom temperature factors were fixed at n (n = 1.2 for Ph-H, CH_2 groups, n = 1.5 for CH_3 groups) times the isotropic temperature factors of the C atoms to which they are bonded. The final anisotropic full-matrix least-squares refinement on F^2 with 181 variables converged at R1 = 6.97%, for the observed data and wR2 = 21.35% for all data. The goodness-of-fit was 1.041. The largest peak in the final difference electron density synthesis was 0.321 e-/Å3 and the largest hole was -0.301 e-/$Å^3$ with an RMS deviation of 0.056 e-/$Å^3$. These largest residues are of no chemical significance. On the basis of the final model, the calculated density was 1.212

g/cm^3 and F(000), 576 e-. The asymmetric unit contains one molecule with the formula of $C_{17}H_{19}NO_2$. The efforts have been made to resolve as many alerts as possible generated by CheckCIF. The current highest alerts are at level G.

Table 1. Crystal data and structure refinement for j1_a.

Identification code	j1_a
Empirical formula	C17 H19 N O2
Formula weight	269.33
Temperature	299(2) K
Wavelength	0.71073 A
Crystal system, space group	Monoclinic, P2(1)/c
Unit cell dimensions	a = 8.7040(4) A alpha = 90 deg.<br>b = 10.3239(5) A beta = 96.819(2) deg.<br>c = 16.5399(8) A gamma = 90 deg.
Volume	1475.75(12) A^3
Z, Calculated density	4, 1.212 Mg/m^3
Absorption coefficient	0.079 mm^-1
F(000)	576
Crystal size	0.420 x 0.360 x 0.150 mm
Theta range for data collection	2.480 to 35.629 deg.
Limiting indices	-14<=h<=14, -16<=k<=16, -27<=l<=27
Reflections collected / unique	69308 / 6795 [R(int) = 0.0674]
Completeness to theta = 25.242	99.9 %
Absorption correction	Semi-empirical from equivalents
Max. and min. transmission	0.7471 and 0.6082
Refinement method	Full-matrix least-squares on F^2
Data / restraints / parameters	6795 / 0 / 181

Goodness-of-fit on F^2	1.041
Final R indices [I>2sigma(I)]	R1 = 0.0697, wR2 = 0.1815
R indices (all data)	R1 = 0.1158, wR2 = 0.2135
Extinction coefficient	n/a
Largest diff. peak and hole	0.321 and -0.301 e.A^-3

Table 2. Atomic coordinates (x 10^4) and equivalent isotropic displacement parameters (A^2 x 10^3) for j1_a.
U(eq) is defined as one third of the trace of the orthogonalized Uij tensor.

	x	y	z	U(eq)
N(1)	7946(1)	6558(1)	3392(1)	36(1)
O(1)	6249(1)	6617(1)	3256(1)	43(1)
O(2)	6781(2)	1156(1)	5151(1)	60(1)
C(1)	5850(1)	7533(1)	2595(1)	38(1)
C(2)	7223(1)	8419(1)	2687(1)	34(1)
C(3)	8409(1)	7804(1)	3166(1)	34(1)
C(4)	9852(2)	8378(1)	3332(1)	45(1)
C(5)	10058(2)	9588(2)	3000(1)	57(1)
C(6)	8882(2)	10205(2)	2523(1)	57(1)
C(7)	7441(2)	9625(1)	2358(1)	46(1)
C(8)	4340(2)	8173(2)	2745(1)	51(1)
C(9)	5717(2)	6828(2)	1784(1)	56(1)
C(10)	8373(2)	6228(1)	4246(1)	48(1)
C(11)	7957(2)	4866(1)	4452(1)	40(1)
C(12)	6981(2)	4636(1)	5040(1)	51(1)
C(13)	6614(2)	3396(2)	5260(1)	53(1)
C(14)	7226(2)	2345(1)	4896(1)	42(1)
C(15)	8216(2)	2546(1)	4312(1)	44(1)
C(16)	8563(2)	3802(1)	4094(1)	45(1)
C(17)	7355(3)	39(2)	4806(1)	69(1)

Table 3. Bond lengths [A] and angles [deg] for j1_a.

N(1)-C(3)	1.4120(15)
N(1)-C(10)	1.4573(17)
N(1)-O(1)	1.4688(13)
O(1)-C(1)	1.4566(15)
O(2)-C(14)	1.3697(16)
O(2)-C(17)	1.405(2)
C(1)-C(2)	1.4987(17)
C(1)-C(9)	1.518(2)
C(1)-C(8)	1.5176(19)
C(2)-C(7)	1.3806(16)
C(2)-C(3)	1.3792(17)
C(3)-C(4)	1.3864(17)
C(4)-C(5)	1.385(2)
C(4)-H(4A)	0.9300
C(5)-C(6)	1.373(3)
C(5)-H(5A)	0.9300
C(6)-C(7)	1.388(2)
C(6)-H(6A)	0.9300
C(7)-H(7A)	0.9300
C(8)-H(8A)	0.9600
C(8)-H(8B)	0.9600
C(8)-H(8C)	0.9600
C(9)-H(9A)	0.9600
C(9)-H(9B)	0.9600
C(9)-H(9C)	0.9600
C(10)-C(11)	1.5009(18)
C(10)-H(10A)	0.9700
C(10)-H(10B)	0.9700
C(11)-C(16)	1.381(2)
C(11)-C(12)	1.386(2)
C(12)-C(13)	1.378(2)
C(12)-H(12A)	0.9300
C(13)-C(14)	1.379(2)
C(13)-H(13A)	0.9300
C(14)-C(15)	1.383(2)
C(15)-C(16)	1.3885(19)
C(15)-H(15A)	0.9300
C(16)-H(16A)	0.9300
C(17)-H(17A)	0.9600
C(17)-H(17B)	0.9600
C(17)-H(17C)	0.9600
C(3)-N(1)-C(10)	114.94(10)
C(3)-N(1)-O(1)	103.61(8)
C(10)-N(1)-O(1)	107.09(10)
C(1)-O(1)-N(1)	106.72(9)
C(14)-O(2)-C(17)	118.88(13)
O(1)-C(1)-C(2)	101.75(9)
O(1)-C(1)-C(9)	109.79(11)
C(2)-C(1)-C(9)	111.00(12)

O(1)-C(1)-C(8)	107.05(11)
C(2)-C(1)-C(8)	114.46(11)
C(9)-C(1)-C(8)	112.14(12)
C(7)-C(2)-C(3)	120.89(12)
C(7)-C(2)-C(1)	130.93(12)
C(3)-C(2)-C(1)	108.13(10)
C(4)-C(3)-C(2)	121.04(12)
C(4)-C(3)-N(1)	128.14(11)
C(2)-C(3)-N(1)	110.71(10)
C(3)-C(4)-C(5)	117.64(14)
C(3)-C(4)-H(4A)	121.2
C(5)-C(4)-H(4A)	121.2
C(6)-C(5)-C(4)	121.59(14)
C(6)-C(5)-H(5A)	119.2
C(4)-C(5)-H(5A)	119.2
C(5)-C(6)-C(7)	120.52(13)
C(5)-C(6)-H(6A)	119.7
C(7)-C(6)-H(6A)	119.7
C(2)-C(7)-C(6)	118.32(14)
C(2)-C(7)-H(7A)	120.8
C(6)-C(7)-H(7A)	120.8
C(1)-C(8)-H(8A)	109.5
C(1)-C(8)-H(8B)	109.5
H(8A)-C(8)-H(8B)	109.5
C(1)-C(8)-H(8C)	109.5
H(8A)-C(8)-H(8C)	109.5
H(8B)-C(8)-H(8C)	109.5
C(1)-C(9)-H(9A)	109.5
C(1)-C(9)-H(9B)	109.5
H(9A)-C(9)-H(9B)	109.5
C(1)-C(9)-H(9C)	109.5
H(9A)-C(9)-H(9C)	109.5
H(9B)-C(9)-H(9C)	109.5
N(1)-C(10)-C(11)	113.48(11)
N(1)-C(10)-H(10A)	108.9
C(11)-C(10)-H(10A)	108.9
N(1)-C(10)-H(10B)	108.9
C(11)-C(10)-H(10B)	108.9
H(10A)-C(10)-H(10B)	107.7
C(16)-C(11)-C(12)	117.47(12)
C(16)-C(11)-C(10)	122.25(13)
C(12)-C(11)-C(10)	120.24(13)
C(13)-C(12)-C(11)	121.64(14)
C(13)-C(12)-H(12A)	119.2
C(11)-C(12)-H(12A)	119.2
C(14)-C(13)-C(12)	120.11(14)
C(14)-C(13)-H(13A)	119.9
C(12)-C(13)-H(13A)	119.9
O(2)-C(14)-C(15)	124.88(13)
O(2)-C(14)-C(13)	115.62(13)
C(15)-C(14)-C(13)	119.50(13)
C(14)-C(15)-C(16)	119.54(13)
C(14)-C(15)-H(15A)	120.2
C(16)-C(15)-H(15A)	120.2

C(11)-C(16)-C(15)	121.73(13)
C(11)-C(16)-H(16A)	119.1
C(15)-C(16)-H(16A)	119.1
O(2)-C(17)-H(17A)	109.5
O(2)-C(17)-H(17B)	109.5
H(17A)-C(17)-H(17B)	109.5
O(2)-C(17)-H(17C)	109.5
H(17A)-C(17)-H(17C)	109.5
H(17B)-C(17)-H(17C)	109.5

Symmetry transformations used to generate equivalent atoms:

Table 4. Anisotropic displacement parameters (A^2 x 10^3) for j1_a. The anisotropic displacement factor exponent takes the form: -2 pi^2 [h^2 a*^2 U11 + ... + 2 h k a* b* U12]

	U11	U22	U33	U23	U13	U12
N(1)	36(1)	32(1)	38(1)	5(1)	-5(1)	2(1)
O(1)	36(1)	38(1)	52(1)	16(1)	-3(1)	-4(1)
O(2)	81(1)	41(1)	61(1)	5(1)	20(1)	-7(1)
C(1)	39(1)	32(1)	41(1)	6(1)	-4(1)	2(1)
C(2)	39(1)	30(1)	34(1)	3(1)	6(1)	0(1)
C(3)	35(1)	31(1)	35(1)	0(1)	4(1)	0(1)
C(4)	36(1)	46(1)	55(1)	-5(1)	4(1)	-3(1)
C(5)	49(1)	50(1)	75(1)	-6(1)	21(1)	-17(1)
C(6)	68(1)	39(1)	69(1)	8(1)	28(1)	-9(1)
C(7)	57(1)	35(1)	48(1)	10(1)	15(1)	2(1)
C(8)	38(1)	44(1)	69(1)	8(1)	3(1)	3(1)
C(9)	61(1)	52(1)	49(1)	-6(1)	-12(1)	2(1)
C(10)	63(1)	38(1)	38(1)	6(1)	-12(1)	-2(1)
C(11)	45(1)	38(1)	34(1)	7(1)	-8(1)	2(1)
C(12)	63(1)	42(1)	49(1)	3(1)	10(1)	11(1)
C(13)	63(1)	49(1)	50(1)	5(1)	20(1)	5(1)
C(14)	48(1)	38(1)	38(1)	5(1)	0(1)	-2(1)
C(15)	52(1)	40(1)	39(1)	-1(1)	5(1)	3(1)
C(16)	50(1)	45(1)	38(1)	5(1)	6(1)	-1(1)
C(17)	98(2)	38(1)	70(1)	3(1)	10(1)	0(1)

Table 5. Hydrogen coordinates (x 10^4) and isotropic displacement parameters (A^2 x 10^3) for j1_a.

	x	y	z	U(eq)
H(4A)	10653	7965	3655	55
H(5A)	11017	9992	3103	68
H(6A)	9053	11019	2310	68
H(7A)	6642	10037	2033	55
H(8A)	4038	8786	2320	76
H(8B)	3550	7525	2751	76
H(8C)	4475	8612	3260	76
H(9A)	5454	7436	1350	84
H(9B)	6687	6424	1718	84
H(9C)	4925	6179	1771	84
H(10A)	7860	6822	4581	58
H(10B)	9480	6343	4379	58
H(12A)	6563	5336	5293	61
H(13A)	5952	3269	5655	64
H(15A)	8646	1844	4067	53
H(16A)	9220	3930	3697	53
H(17A)	6947	-716	5043	103
H(17B)	7048	39	4229	103
H(17C)	8464	30	4909	103

Table 6. Torsion angles [deg] for j1_a.

C(3)-N(1)-O(1)-C(1)	29.78(12)
C(10)-N(1)-O(1)-C(1)	151.68(10)
N(1)-O(1)-C(1)-C(2)	-28.44(12)
N(1)-O(1)-C(1)-C(9)	89.20(12)
N(1)-O(1)-C(1)-C(8)	-148.85(11)
O(1)-C(1)-C(2)-C(7)	-165.64(13)
C(9)-C(1)-C(2)-C(7)	77.60(17)
C(8)-C(1)-C(2)-C(7)	-50.58(19)
O(1)-C(1)-C(2)-C(3)	17.10(13)
C(9)-C(1)-C(2)-C(3)	-99.66(13)
C(8)-C(1)-C(2)-C(3)	132.16(12)
C(7)-C(2)-C(3)-C(4)	0.09(19)
C(1)-C(2)-C(3)-C(4)	177.68(12)
C(7)-C(2)-C(3)-N(1)	-176.49(11)
C(1)-C(2)-C(3)-N(1)	1.10(14)
C(10)-N(1)-C(3)-C(4)	48.43(18)
O(1)-N(1)-C(3)-C(4)	164.92(13)
C(10)-N(1)-C(3)-C(2)	-135.30(12)
O(1)-N(1)-C(3)-C(2)	-18.80(13)
C(2)-C(3)-C(4)-C(5)	0.0(2)
N(1)-C(3)-C(4)-C(5)	175.93(13)
C(3)-C(4)-C(5)-C(6)	0.1(2)
C(4)-C(5)-C(6)-C(7)	-0.2(3)
C(3)-C(2)-C(7)-C(6)	-0.2(2)
C(1)-C(2)-C(7)-C(6)	-177.20(14)
C(5)-C(6)-C(7)-C(2)	0.3(2)
C(3)-N(1)-C(10)-C(11)	-176.11(11)
O(1)-N(1)-C(10)-C(11)	69.41(15)
N(1)-C(10)-C(11)-C(16)	61.11(18)
N(1)-C(10)-C(11)-C(12)	-121.38(15)
C(16)-C(11)-C(12)-C(13)	-0.4(2)
C(10)-C(11)-C(12)-C(13)	-178.04(14)
C(11)-C(12)-C(13)-C(14)	0.3(3)
C(17)-O(2)-C(14)-C(15)	0.0(2)
C(17)-O(2)-C(14)-C(13)	179.84(16)
C(12)-C(13)-C(14)-O(2)	-179.49(15)
C(12)-C(13)-C(14)-C(15)	0.4(2)
O(2)-C(14)-C(15)-C(16)	179.01(14)
C(13)-C(14)-C(15)-C(16)	-0.8(2)
C(12)-C(11)-C(16)-C(15)	-0.1(2)
C(10)-C(11)-C(16)-C(15)	177.50(13)
C(14)-C(15)-C(16)-C(11)	0.7(2)

Symmetry transformations used to generate equivalent atoms:

12) References

1) D. Swern (Ed.), Organic Peroxides, Vol. 3, John Wiley & Sons, New York (1972), pp. 341-364.
2) Kropf, H.; Bernert, C. Organische Peroxide, VII. Verhalten von Phenyl- Und Benzyl-tert.-butylhydroperoxiden Bei Thermischer Zersetzung, Hock- Und Criegee-Umlagerung. *Justus Liebigs Ann. Chem.* **1971**, *751* (1), 109–120.
3) Kan, T.; Fukuyama, T. Ns strategies: A highly Versatile Synthetic Method for Amines. *Chem. Commun*. **2003**, 353–359.
4) Olsen, C. A.; Witt, M.; Hansen, S. H.; Jaroszewski, J. W.; Franzyk, H. Fukuyama–Mitsunobu Alkylation in Amine Synthesis on Solid Phase Revisited: N-Alkylation with Secondary Alcohols and Synthesis of Curtatoxins. *Tetrahedron* **2005**, *61* (25), 6046–6055.
5) Groendyke, B. J.; Modak, A.; Cook, S. P. Fenton-Inspired C–H Functionalization: Peroxide-Directed C–H Thioetherification. *J. Org. Chem.* **2019**, *84* (20), 13073–13091.
6) Isayama, S.; Mukaiyama, T. Novel Method for the Preparation of Triethylsilyl Peroxides from Olefins by the Reaction with Molecular Oxygen and Triethylsilane Catalyzed by Bis(1,3-Diketonato)Cobalt(II). *Chem. Lett.* **2006**, *18* (4), 573–576.
7) O'Neill, P. M.; Hindley, S.; Pugh, M. D.; Davies, J.; Bray, P. G.; Park, B. K.; Kapu, D. S.; Ward, S. A.; Stocks, P. A. Co(Thd)2: A Superior Catalyst for Aerobic Epoxidation and Hydroperoxysilylation of Unactivated Alkenes: Application to the Synthesis of Spiro-1,2,4-Trioxanes. *Tetrahedron Lett.* **2003**, *44* (44), 8135–8138.

8) Feng, Y.; Holte, D.; Zoller, J.; Umemiya, S.; Simke, L. R.; Baran, P. S. Total Synthesis of Verruculogen and Fumitremorgin A Enabled by Ligand-Controlled C–H Borylation. *J. Am. Chem. Soc.* **2015**, *137* (32), 10160–10163.

9) Olson, A; Jameson, A; Kyasa, S; Evans, B; Dussault, P. Reductive Cleavage of Organic Peroxides by Iron Salts and Thiols. *ACS Omega* **2018**, *3* (10), 14054-14063.

10) Siu, J; Parry, J; Lin, S. Aminoxyl-Catalyzed Electrochemical Diazidation of Alkenes Mediated by a Metastable Charge-Transfer Complex. *J. Am. Chem Soc.* **2019** *141* (7), 2825-2831.

11) Kaga, A; Lin Tnay, Y; Chiba, S. Synthesis of Fasicularin. *Org. Lett.* **2016** *18* (14), 3506-3508.

12) Dong, XY., Zhang, YF., Ma, CL; Gu, QS; Wang, FL; Li, ZL; Jiang, SP; Liu, XY. A general asymmetric copper-catalyzed Sonogashira C(*sp*^{3})–C(*sp*) coupling. *Nat. Chem.* **2019** *11* (12), 1158–1166.

13) Farhad, N; Batey, N. Regio- and Stereoselective Allylation and Crotylation of Indoles at C2 Through the Use of Potassium Organotrifluoroborate Salts. *Angew. Chem., Int. Ed.* **2013**, *52* (3), 892-895.

14) Zheng, Y; Hu, Q; Huang, Q; Xie, Y. Late-Stage C–H Nitration of Unactivated Arenes by $Fe(NO_3)_3\cdot 9H_2O$ in Hexafluoroisopropanol. *Org. Lett.* **2024** *26* (15), 3316-3320.

15) Guastavino, J; Rossi, R. Synthesis of Benzo-fused Heterocycles by Intramolecular α-Arylation of Ketone Enolate Anions. *J. Org Chem.* **2012** *77* (1), 460-472.

16) Fan, Y; Saiki, Y; Nakaoka, K; Ema, T. One-Pot Synthesis of Aldehydes or Alcohols from CO2 via Formamides or Silyl Formates. *Adv. Synth. Catal.* **2023** *365* (6), 877-883.

17) (a) Bruker AXS Inc. *APEX3 Crystallography Software Suite*, 2016, 5465 East Cheryl Parkway, Madison, WI 53711,USA; (b) Sheldrick, G.M. *A Short History of SHELX*, Acta Cryst. 2008, A64, 112-122; (c) Sheldrick, G.M. *Crystal structure refinement with SHELXL*, Acta Cryst. 2015, C71, 3-8.
